# A visible-light mediated ring opening reaction of alkylidenecyclopropanes for the generation of homopropargyl radicals†

**DOI:** 10.1039/d1sc01889b

**Published:** 2021-05-28

**Authors:** Xiao-Yu Zhang, Chao Ning, Ben Mao, Yin Wei, Min Shi

**Affiliations:** Key Laboratory for Advanced Materials and Institute of Fine Chemicals, Key Laboratory for Advanced Materials and Feringa Nobel Prize Scientist Joint Research Center, School of Chemistry & Molecular Engineering, East China University of Science and Technology Meilong Road No. 130 Shanghai 200237 China; State Key Laboratory of Organometallic Chemistry, Shanghai Institute of Organic Chemistry, Chinese Academy of Sciences 345 Lingling Road Shanghai 200032 China weiyin@sioc.ac.cn mshi@mail.sioc.ac.cn

## Abstract

Classical cyclopropylcarbinyl radical clock reactions have been widely applied to conduct mechanistic studies for probing radical processes for a long time; however, alkylidenecyclopropanes, which have a similar molecular structure to methylcyclopropanes, surprisingly have not yet attracted researcher's attention for similar ring opening radical clock processes. In recent years, photocatalytic NHPI ester activation chemistry has witnessed significant blooming developments and provided new synthetic routes for cross-coupling reactions. Herein, we wish to report a non-classical ring opening radical clock reaction using innovative NHPI esters bearing alkylidenecyclopropanes upon photoredox catalysis, providing a brand-new synthetic approach for the direct preparation of a variety of alkynyl derivatives. The potential synthetic utility of this protocol is demonstrated in the diverse transformations and facile synthesis of bioactive molecules or their derivatives and medicinal substances.

## Abstract 2

A non-classical ring opening radical clock reaction using the innovative NHPI esters bearing alkylidenecyclopropanes upon photoredox catalysis has been demonstrated, providing a brand-new synthetic approach to access a variety of alkynyl derivatives.

## Graphical abstract

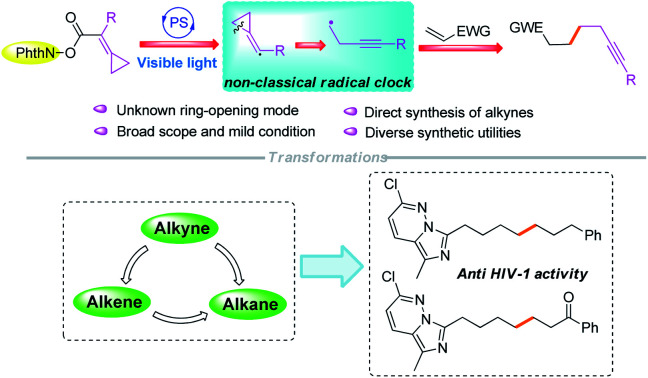

## Introduction

**Scheme 1:**
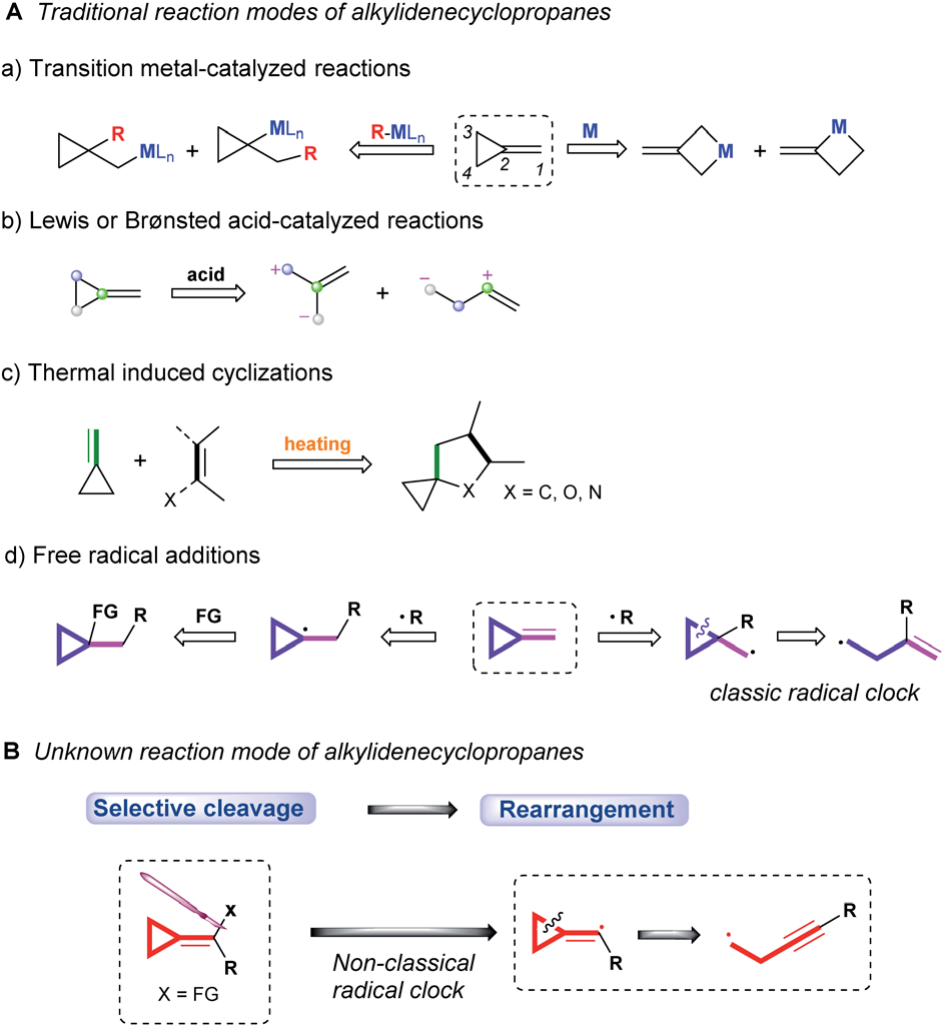
Traditional reaction modes of alkylidenecyclopropanes and unknown reaction mode of alkylidenecyclopropanes.

Over the past few decades, the ring-opening reactions of alkylidenecyclopropanes (ACPs) have been vigorously explored, due to their high reactivity endowed by ring strain.^1^ In general, the reaction modes of alkylidenecyclopropanes can be mainly classified into the following: transition metal-catalyzed reactions,^2^ Lewis or Brønsted acid-catalyzed/mediated reactions,^3^ thermal induced cyclizations^4^ and free radical additions (Scheme 1A).^5^ Since the 1970s, the research on the chemistry of ACPs upon transition metal catalysis has been in progress, and four different reaction patterns have been disclosed thus far (Scheme 1A, a). The distal bond (C3–C4) and proximal bond (C2–C3 or C2–C4) of ACPs can be inserted by transition metal **M** to undergo the subsequent reactions. On the other hand, the organo-transition metal reagent R–**M**L_*n*_ can be added into alkylidenecyclopropanes *via anti*-Markovnikov or Markovnikov addition, leading to the ring opening of cyclopropane or other transformations. In the reaction of ACPs with Lewis or Brønsted acids, the cyclopropyl ring can be activated to undergo ring opening to form two different zwitterionic intermediates, which can undergo further transformations (Scheme 1A, b). As for thermally induced cyclizations, heating would facilitate the double bond of ACPs to react with a dienophile or dipole to afford spirocyclic derivatives (Scheme 1A, c).

Furthermore, the application of alkylidenecyclopropanes in radical C–C bond forming reactions has also received much attention. The addition of radicals into alkylidenecyclopropanes could generate cyclopropyl radicals or cyclopropylcarbinyl radicals. The former can be further transformed to achieve the difunctionalization of alkenes, and the latter can undergo β-scission in the cyclopropyl ring to afford the homoallyl radical known as a *classical radical clock* (Scheme 1A, d). These synthetic methods provide an efficient access to the rapid generation of molecular complexity. However, with increasing exploration on research means and technologies, nearly no breakthrough on the chemical transformation of alkylidenecyclopropanes has been made for a long time. More efforts to develop new reaction modes of alkylidenecyclopropanes are highly desired in current research.

**Scheme 2:**
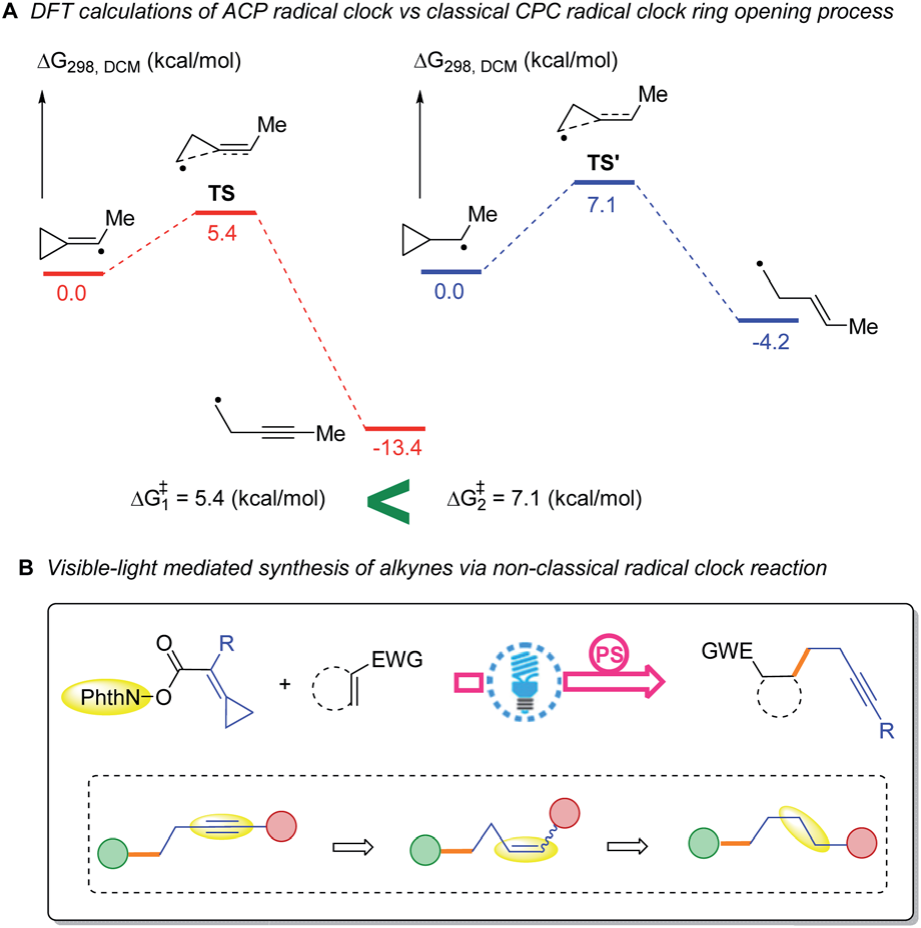
DFT calculations of the ACP radical clock *vs.* classical CPC ring opening radical clock process. Alkylidenecyclopropanes and this work.

The ring opening of the cyclopropylcarbinyl (CPC) radical to the 3-butenyl radical is a fast radical rearrangement that holds a position of distinction in mechanistic studies involving “radical clocks” and “mechanistic probes”. Numerous research studies have adopted potential precursors of this radical or its analogues in attempts to implicate radical intermediates in a reaction pathway by the detection of the products from the radical ring opening process.^6^ At the same time, it is found that this methodology and the final detected products bearing alkenyl groups can be applied to diverse organic synthesis.^7^ Since alkylidenecyclopropanes (ACPs) have a similar molecular structure to methylcyclopropane and the release of ring strain can provide a potent thermodynamic driving force, we designed an unknown radical reaction mode, in which the C(sp^2^)–X bond of alkylidenecyclopropane can be selectively cleaved to afford the alkylidenecyclopropane radical (Scheme 1B). This radical can further undergo the same ring opening rearrangement process to generate the corresponding radical species containing an alkynyl moiety and subsequently play a vital role in mechanistic studies and diverse organic synthesis. Thus, we conducted preliminary density functional theory (DFT) calculations to predict whether the envisaged process is kinetically feasible (Scheme 2A) (see the ESI† for the details). The calculation results showed that the energy barrier for the ring-opening process of the alkylidenecyclopropane radical 
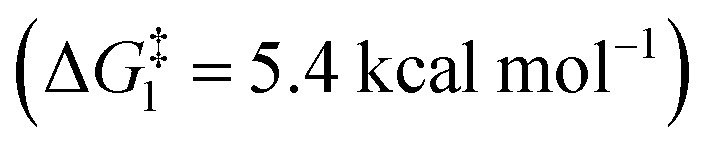
 is lower than that for the ring-opening process of the CPC radical 
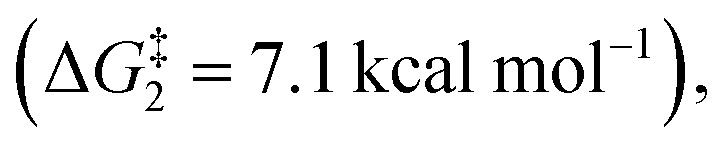
 indicating that the ring-opening process of the alkylidenecyclopropane radical is kinetically favorable. In addition, the ring-opening process of the alkylidenecyclopropane radical is an exergonic process (Δ*G*_1_ = −13.4 kcal mol^−1^), suggesting that this is a thermodynamically favorable process. Based on these calculation results, developing reactions involving the ring opening process of the alkylidenecyclopropane radical is feasible.

Recently, NHPI (*N*-hydroxyphthalimide) esters, as stable and readily accessible compounds, have emerged as a novel and efficient synthon to undergo a number of interesting and practical transformations.^8^ Due to their redox reactivity, NHPI esters can serve as a radical precursor to accept an electron to trigger a consecutive domino of events that release phthalimidyl anions, CO_2_ and the corresponding radicals applied for a variety of reactions.^9^ Photoredox catalysis induced by visible light, owing to the advantages of ease of handling, application safety and natural abundance, is utilized as one of the accesses fulfilling the described process.^10^ Visible-light induced carbon–carbon bond-forming reactions involving NHPI esters were identified as an ideal strategy to construct various interesting compounds. Such reactions can be categorized as follows: (i) C(sp^3^)–C(sp^3^) bond formation; (ii) C(sp^3^)–C(sp^2^) bond formation; (iii) C(sp^3^)–C(sp) bond formation; (iv) C(sp^3^)–B or Se/S bond formation.^8^ However, in these reactions, NHPI esters directly release the C(sp^3^) radicals applied for the bond formation. No examples of releasing the highly reactive vinyl radicals as C(sp^2^) radicals have been reported to date. With regard to this aspect, we skillfully take advantage of the high reactivity of C(sp^2^) radicals to fulfill our design. Building on the widely investigated field of classical ring opening radical clock reactions and the successes of photocatalytic NHPI esters in decarboxylative activity chemistry, we are inspired by the possibility of an innovative non-classical ring opening radical clock reaction to provide both terminal and internal alkynes (Scheme 2B). Notably, it is convinced that different levels of saturation of small molecules can cause a broad spectrum of transformations, especially applicable in the areas of pharmaceutical and agrochemical production. Therefore, the transformations of alkynes into alkenes and alkanes fulfilled by our envisaged method would provide a practical synthetic route (Scheme 2B).

## Results and discussion

### Experimental investigations

We began our investigation with easily available NHPI ester **1b** (0.075 mmol, 1.5 equiv.) and phenyl vinyl ketone **2a** (0.05 mmol, 1.0 equiv.) as the model substrates, Ru(bpy)_3_(PF_6_)_2_ as the photosensitizer, and Hantzsch ester and ^*i*^Pr_2_NEt as additives in dichloromethane (0.75 mL) at room temperature upon the irradiation of a 15 W blue LED for 3 h, giving the desired alkyne **3ba** in 87% yield (Table 1, entry 1). The optimization of reaction conditions revealed that the photosensitizer, light and additive were indispensable in this reaction (Table 1, entries 2–4). Other metal-polypyridyl complex photosensitizers were also screened, and the results showed that Ru(bpz)_3_(PF_6_)_2_ was less effective as compared with Ru(bpy)_3_(PF_6_)_2_, but the use of *fac*-Ir(ppy)_3_ did not afford the desired product (Table 1, entries 5-6). Instead of *i*Pr_2_NEt, other additives such as DBU and DABCO were tested, which resulted in decreased yields (Table 1, entries 7-8). We further investigated other solvents, such as THF and DCE, providing **3ba** in 71% and 57% yields (Table 1, entries 9-10), respectively, identifying DCM as the most suitable solvent. Using 1.0 equiv. of **1b** or prolonging the reaction time to 12 h could not effectively improve the yield of **3ba** (Table 1, entries 11-12).

**Table 1:** Optimization of the reaction conditions^a^

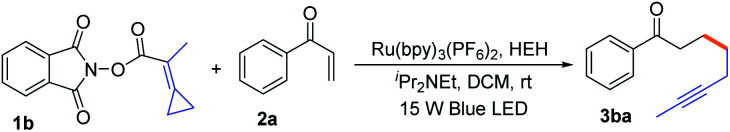		
Entry	Condition	Yield^b^ (%)
1	Standard condition	87
2	Without Ru(bpy)_3_(PF_6_)_2_	NR
3	Without blue LED	NR
4	Without ^*i*^Pr_2_NEt	15
5	Ru(bpz)_3_(PF_6_)_2_ instead of Ru(bpy)_3_(PF_6_)_2_	34
6	*fac*-lr(ppy)_3_ instead of Ru(bpy)_3_(PF_6_)_2_	NR
7	DBU instead of ^*i*^Pr_2_NEt	21
8	DABCO instead of ^*i*^Pr_2_NEt	13
9	THF instead of DCM	71
10	DCE instead of DCM	57
11	1.0 equiv. **1b**	66
12	12 h instead of 3 h	84

a Reaction conditions: **1b** (0.075 mmol, 1.5 equiv.), **2a** (0.05 mmol, 1.0 equiv.), Hantzsch ester (HEH) (0.075 mmol, 1.5 equiv.), ^*i*^Pr_2_NEt (0.11 mmol, 2.2 equiv.), Ru(bpy)_3_(PF_6_)_2_ (2 mol%), DCM (0.75 mL), rt, 3 h, Ar, and 15 W blue LED. All the reactions were carried out on a 0.05 mmol scale in solvent (0.75 mL) at rt for 3 h unless otherwise specified.

b Isolated yield.

With the optimal reaction conditions in hand, we attempted to investigate the substrate scope of various NHPI esters bearing alkylidenecyclopropanes and the results are shown in Table 2. When the substituent R was a hydrogen atom, the reaction could proceed smoothly to provide the terminal alkyne **3aa** in 75% yield. For substrates **1c** and **1d**, ethyl group and propyl group substituted NHPI esters were compatible in this reaction, generating the corresponding internal alkynes **3ca** and **3da** in 89% and 92% yields, respectively. When the R group was changed into the benzyl group bearing different substituents, such as 4-H, 4-OMe, 4-Cl and 3,5-Br (**1e–1h**), the desired products **3ea–3ha** were obtained in 89–93% yields. Moreover, we attempted to introduce featured substituents, such as allyl, propargyl and cinnamyl groups, into the substrates, and found that the reactions also proceeded smoothly to afford the corresponding internal alkynes **3ia**, **3ja** and **3ka** in good yields ranging from 80–94%. Notably, the substrate **1la** having an aromatic heterocycle was also suitable for this reaction, affording the desired product **3la** in 85% yield.

**Table 2:** Substrate scope of NHPI esters bearing alkylidenecyclopropanes^a^

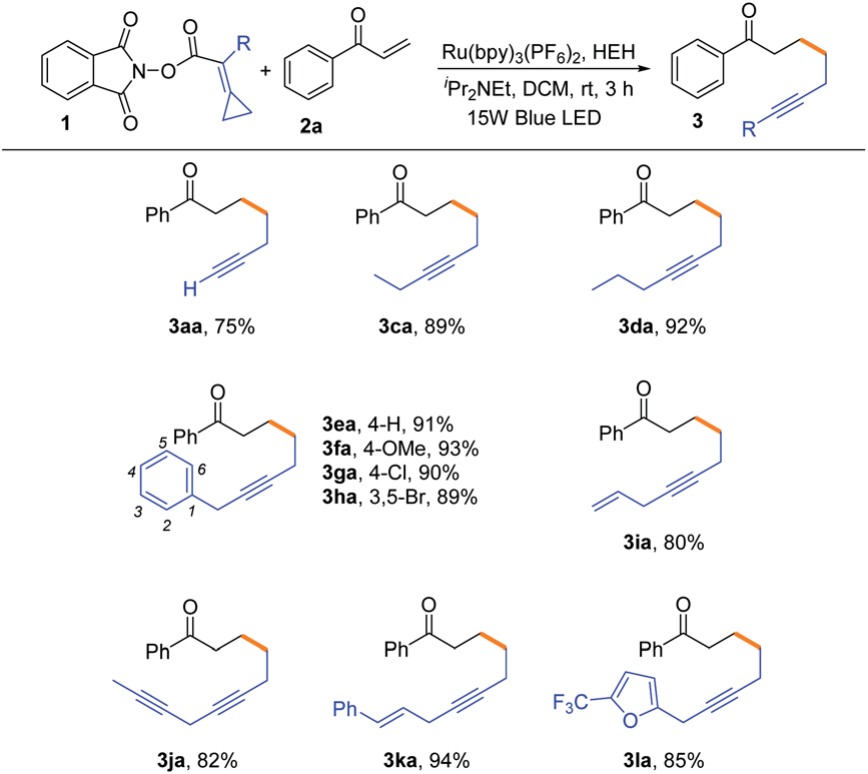

a Reaction conditions: **1** (0.3 mmol, 1.5 equiv.), **2a** (0.2 mmol, 1.0 equiv.), Hantzsch ester (HEH) (0.3 mmol, 1.5 equiv.), ^*i*^Pr_2_NEt (0.44 mmol, 2.2 equiv.), Ru(bpy)_3_(PF_6_)_2_ (2 mol%), DCM (3 mL), rt, 3 h, Ar, and 15 W blue LED. Yields were determined from isolated products.

Next, we focused on the generality of this protocol regarding α,β-unsaturated carbonyl compounds. As outlined in Table 3, most of the reactions proceeded smoothly under well-established conditions to generate the expected products in moderate to good yields. First, we examined aryl substituted α,β-unsaturated ketones (**2b–2i**). The mild conditions enabled the tolerance to important functional groups, such as halide, cyano, ester, alkoxy and so on, affording the corresponding alkynes **3bb–3bi**, in the yields ranging from 75% to 92%. Moreover, alkyl substituted α,β-unsaturated ketones (**2j–2n**) were also compatible in this reaction, affording the desired alkynes **3bj–3bn**, albeit with moderate yields (36–43%). For the α,β-unsaturated amide **2o**, the reaction could also proceed smoothly to produce the desired product **3bo** in 33% yield. When the *α* position of α,β-unsaturated ketones was substituted by ester and phenyl groups, the reaction occurred smoothly to give the expected products **3bp** and **3bq** in 87% and 81% yields, respectively. For conjugated diene substrate **2r**, in which the *δ* position was substituted by a methyl group, the corresponding product **3br** was obtained in 92% yield, probably due to the radical addition taking place at the *δ* position and the relative stability of key radical intermediates. It is well known that aromatic heterocycles play an important role in medicinal chemistry; therefore, α,β-unsaturated ketones bearing furyl, thienyl, pyrrole, indolyl, benzofuryl and imidazolyl groups were applied to this reaction to generate alkynes having aromatic heterocycles. To our delight, these reactions also proceeded very well to provide the corresponding alkynes **3bs–3bx** in moderate to good yields. For cyclic *exo*-methylene ketones **2y** and **2z**, the desired products **3by** and **3bz** were obtained in both 78% yields. Notably, when the alkenyl sulfone **2aa** was utilized as the substrate, the desulfonylative cross-coupling reaction occurred, resulting in the formation of product **3baa** in 97% yield. Finally, we illustrated the utility of this novel non-classical radical clock reaction using the core structures of several bioactive molecules, such as geraniol (**2ab**), estrone (**2ad**) and vanillin (**2ae**), and a mesogenic compound (**2ac**). All the desired alkynylated products were obtained in good yields (78–88%), irrespective of existing complex molecular structures. In addition, acrylesters, acrylamides, *exo*-cyclic lactone and simple crotonyl or cinnamoyl substrates were also attempted to use as the radical acceptors to proceed this reaction; however, no desired products were obtained (for unsuccessful examples, see Page S3 in the ESI†). Unfortunately, we did not succeed in obtaining an aryl-substituted substrate after several attempts.

**Table 3:** Substrate scope of α,β-unsaturated ketones^a^

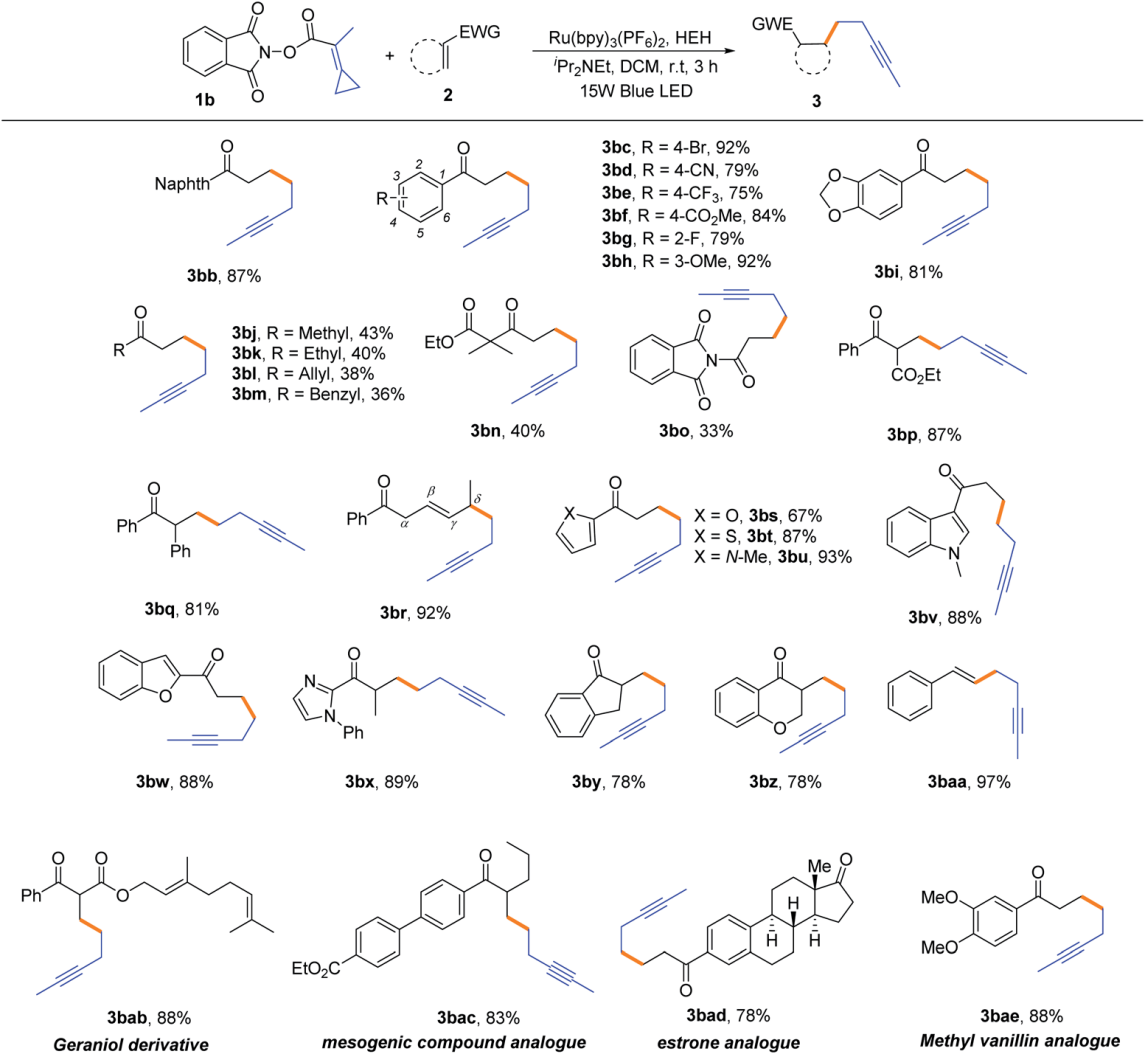

a Reaction conditions: **1b** (0.3 mmol, 1.5 equiv.), **2** (0.2 mmol, 1.0 equiv.), Hantzsch ester (HEH) (0.3 mmol, 1.5 equiv.), ^*i*^Pr_2_NEt (0.44 mmol, 2.2 equiv.), Ru(bpy)_3_(PF_6_)_2_ (2 mol%), DCM (3 mL), rt, 3 h, Ar, and 15 W blue LED. Yields were determined from isolated products.

### Synthetic applications

**Scheme 3:**
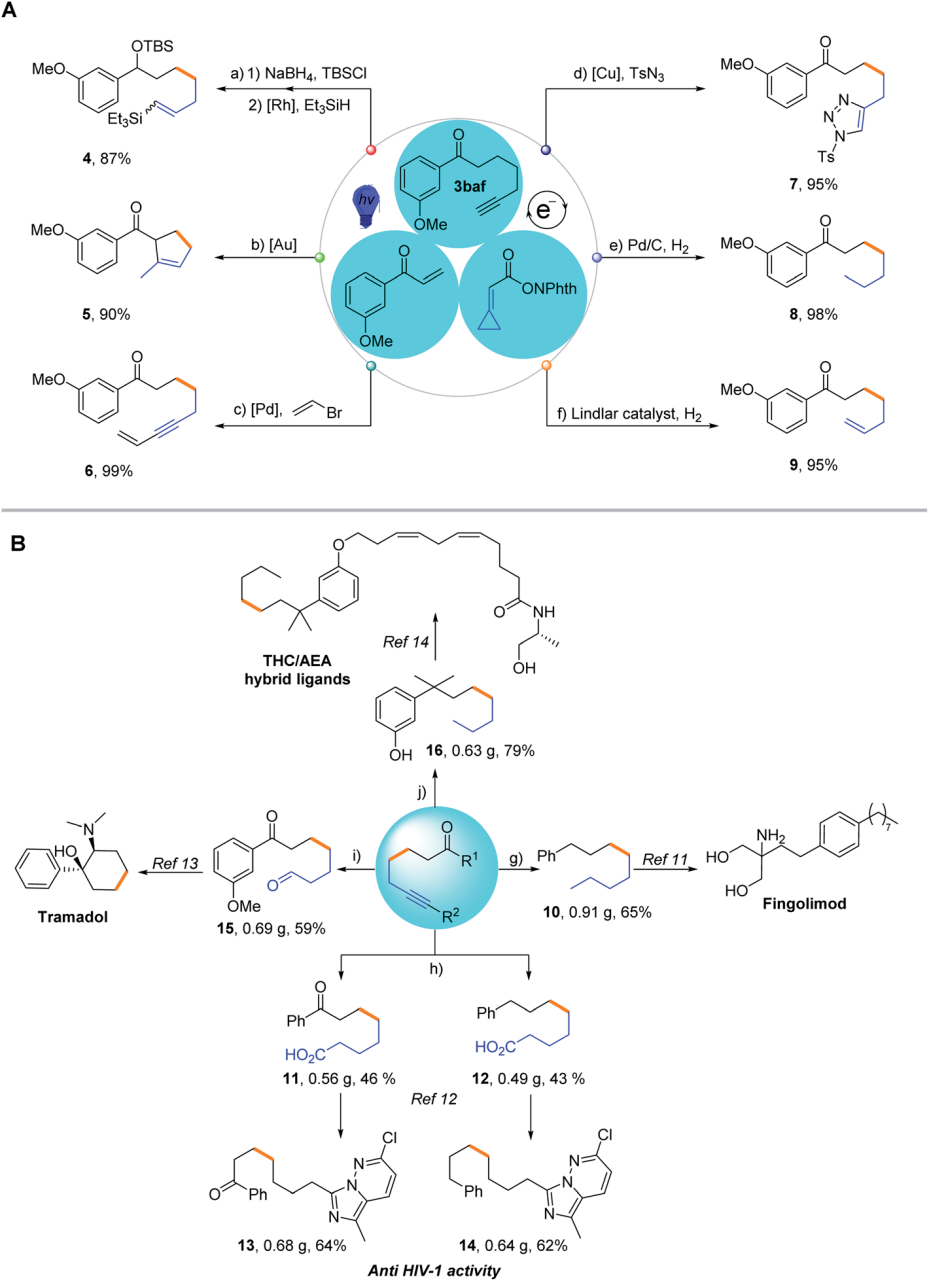
Synthetic applications of the non-classical ring opening radical clock reaction. (A) Product transformations of **3baf**. (a) NaBH_4_, MeOH, 0 °C – rt; DMAP, imidazole, TBSCl, DCM, 0 °C – rt, and 12 h; Rh(COD)_2_BF_4_, PPh_3_, Et_3_SiH, acetone, rt, and 20 h. (b) AuCl_3_, AgOTf, toluene, rt, and 8 h. (c) Pd(PPh_3_)_2_Cl_2_, CuI, Et_3_N, THF, rt, 12 h. (d) CuTc, toluene, rt, and 8 h. (e) Pd/C, MeOH, H_2_, rt, and 4 h. (f) Lindlar catalyst, MeOH, H_2_, rt, and 8 h. (B) Synthesis of drugs applying our methodology. (g) Pd/C, MeOH, H_2_, rt, and 4 h; NaBH_4_, AlCl_3_, THF, and 0 °C - 65 °C. (h) ethylene glycol, *p*-TsOH, toluene, and 110 °C; *n*-BuLi, propyl chloroformate, THF, and −78 °C; Pd/C, MeOH, H_2_, rt, and overnight; LiOH, THF, H_2_O, and rt; DMAP, DCC, Et_3_N, DCM, DMF, and 0 °C – rt; 6-(1-aminoethyl)pyridazin-3(2*H*)-one **S6**, POCl_3_, DCE, reflux, and 3 h. (i) BH_3_·THF, cyclohexene, THF, 0 °C, and 2 h; NaBO_3_·4H_2_O, H_2_O, and 2 h. (j) Pd/C, MeOH, H_2_, rt, and 4 h; TiCl_4_, (CH_3_)_2_Zn, DCM, −50 °C, and 2 h; BBr_3_, DCM, 0 °C, and 12 h.

To demonstrate the preparative utility of our methodology, product transformations were conducted (Scheme 3A). The resulting alkynes can act as versatile forging blocks for diversity-oriented synthesis. A rhodium-catalyzed hydrosilylation reaction and gold-catalyzed cyclization of **3baf** were conducted to afford the corresponding products, silylated compound **4** and cyclized product **5** in 87% and 90% yields, respectively. The Sonogashira cross-coupling reaction of **3baf** with vinyl bromide also proceeded smoothly, giving the enyne **6** in 99% yield, which could be utilized for the construction of valuable building blocks, such as pyrroles. The click reaction of **3baf** with tosyl azide *via* copper catalysis afforded the [3 + 2] cycloaddition product **7** in 95% yield, demonstrating the potential practicability of the resulting alkynes in medicinal chemistry. Hydrogenation or partial hydrogenation of the obtained alkyne **3baf** effectively generated the corresponding alkane **8** and alkene **9**. As for the immediate potential in biochemistry and medicinal chemistry, we have easily synthesized five different synthetic precursors **10**, **11**, **12**, **15** and **16** on a gram scale through this methodology (Scheme 3B). Compound **10** was synthesized from **3ba** through a two-step reduction procedure in 65% yield (0.91 g), which was regarded as the key intermediate for the synthesis of Fingolimod, mainly available for the treatment of Multiple Sclerosis (MS).^11^ Starting from **3aa**, the two vital precursors **11** and **12** were synthesized in 46% (0.56 g) and 43% (0.49 g) yields, respectively. Precursors **11** and **12** could be further transformed into substituted imidazo [1,5-*b*] pyridazine **13** and its analogue **14**, which are proved to be exceptionally active against the reverse transcriptase of HIV-1, in 64% (0.68 g) yield and 62% (0.64 g) yield, respectively^12^ (for details on the formal synthesis or total synthesis of bioactive molecules, see Pages S13 and S14 in the ESI†). Since 1977, Tramadol has been used as a painkiller by serving as a weak μ-opioid receptor agonist;^13^ therefore, we synthesized the vital precursor **15** through a one-pot selective hydroboration and oxidation of alkyne **3baf** in 59% yield (0.69 g). Furthermore, on the basis of Razdan's protocol,^14^ we prepared the building block **16** in 79% yield (0.63 g) through the reduction and demethylation of alkyne **3baf**, which could be applied to assemble the novel tetrahydrocannabinol/anandamide (THC/AEA) hybrid ligand. This modified ligand could significantly improve its binding affinity to the CB1 receptor in biological research. In summary, these diverse transformations exhibit the strong capability of our non-classical radical clock reaction coupled with various subsequent manipulations for generating terminal or internal alkynes and their derivatives, which would be synthetically very useful along with excellent functional group tolerance.

### Mechanistic studies

**Fig. 1:**
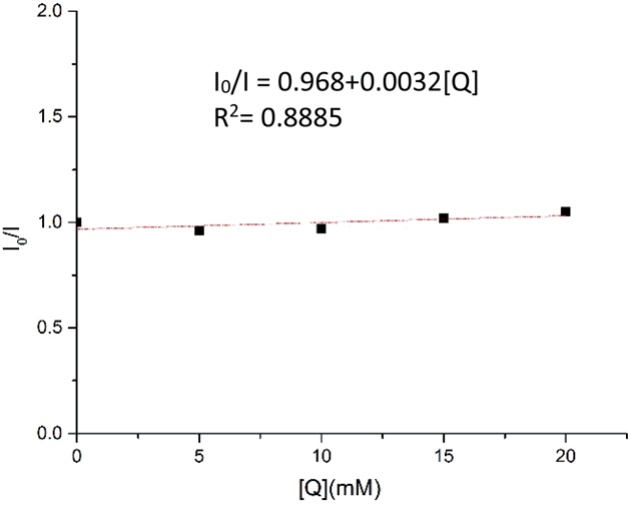
Stern–Volmer quenching experiment.

**Scheme 4:**
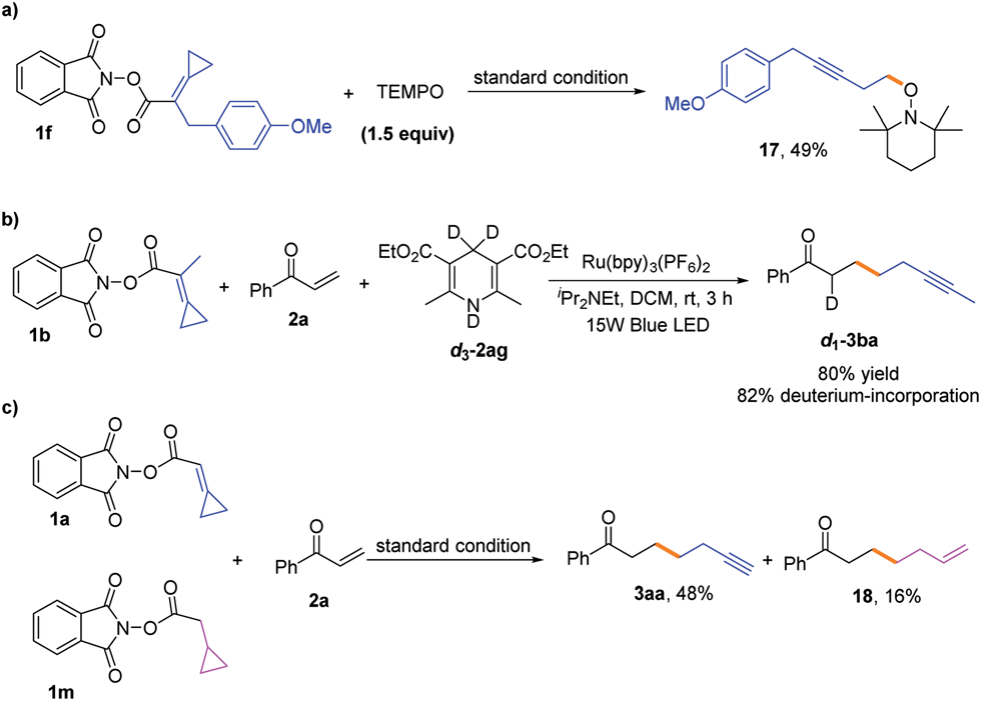
Mechanistic investigations.

To gain more insights into the reaction mechanism, the fluorescence quenching experiments of Ru(bpy)_3_(PF_6_)_2_ with **1b** were performed and its Stern–Volmer analysis is depicted in Fig. 1, indicating that **1b** could not be photo-excited by the photocatalyst effectively. In addition, we carried out several control experiments (Scheme 4). First, when TEMPO as a radical scavenger was subjected to our model reaction, the non-classical radical clock reaction was inhibited completely and a new product **17** generated from the resultant alkyl radical trapped by TEMPO was obtained in 49% yield (Scheme 4, a). Subsequently, a reaction using deuterium-labeled Hantzsch ester *d*_3_-**2af** was conducted, and the deuterium atom was incorporated exclusively at the *α* position of alkyne *d*_1_-**3ba**, showing that SET and HAT processes were both involved in the last step^15^ (Scheme 4, b). Substrate **1a** (*E*_1/2_ = −1.18 V *vs.* SCE, see Page S18 in the ESI†) and radical clock substrate **1m** (*E*_1/2_ = −1.24 V *vs.* SCE, see Page S18 in the ESI†) were mixed together to undergo the reaction, affording the corresponding products **3aa** and **18** in 48% and 16% yield, respectively (Scheme 4, c). This reaction outcome basically agrees with the prediction of DFT calculations, which indicate that the ring-opening process of the alkylidenecyclopropane radical is kinetically more favorable.

**Scheme 5:**
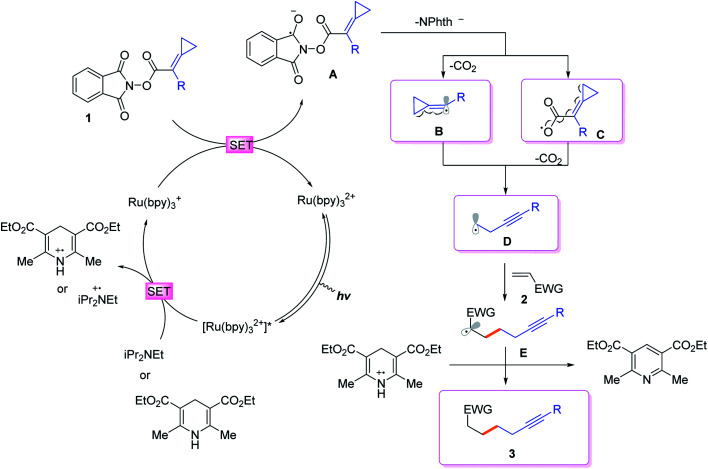
Proposed reaction mechanisms.

On the basis of generally accepted mechanisms for NHPI ester's decarboxylative activity,^8,15^ a plausible catalytic cycle is proposed in Scheme 5. The initial excitation of Ru(bpy)_3_(PF_6_)_2_ produces the excited state 
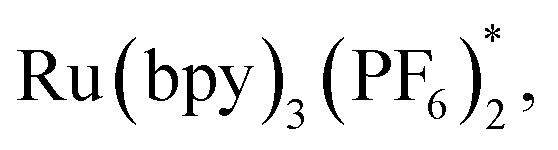
 which undergoes SET with ^*i*^Pr_2_NEt or Hantzsch ester to generate the Ru(i), amine radical cation or Hantzsch ester radical cation. The *N*-(acyloxy)phthalimide **1** receives an electron from Ru(i) (*E*_1/2_ = −1.33 V *vs.* SCE), to transiently form radical anionic intermediate **A**. Next, an entropically favored decarboxylation can proceed *via* two plausible pathways to generate the desired radical intermediate **D**: (i) rapid homolytic fragmentation and decarboxylation from **A** to release the phthalimide anion, CO_2_ and the radical intermediate **B**. As a central design element, we postulated that **B** would undergo the ring-opening process to produce the nucleophilic carbon-centered radical bearing alkynyl **D**, or (ii) homolytic fragmentation of the N–O bond to furnish intermediate **C**, which can further undergo decarboxylation and radical rearrangement to **D**. The addition of this nucleophilic radical to a conjugate acceptor **2** generates a stabilized radical **E**, which then undergoes SET and HAT processes to successfully form the functionalized alkyne **3**. Notably, the radical intermediate **E** was not engaged in a fast 5-*exo*-dig-cyclization to afford the cyclized product; therefore, we assumed that the cyclization is reversible and reduction only occurred on the ring-opened isomer.

## Conclusions

In conclusion, we have successfully established a robust methodology to access alkynes *via* the versatile and practical non-classical ring-opening radical clock reactions of NHPI esters bearing ACPs inspired by conventional photocatalytic NHPI ester's activation and classical radical clock reactions. Compared with the traditional reaction modes of alkylidenecyclopropanes, this method is totally new and its prospect will be prosperous for the rapid generation of molecular complexity in organic synthesis. Most importantly, since the carbon–carbon triple bond can serve as a versatile functional group for diverse organic synthesis, the resulting alkynes can be transformed into a variety of bioactive molecules and natural products. Further studies for discovering detailed mechanisms and novel reaction types to synthesize other useful compounds are underway.

## Author contributions

X.-Y. Z., C.N. and B. M. contributed to the experimental work; Y. W. contributed to the computational work. X.-Y. Z., Y. W. and M. S. contributed to ideation and writing of the paper.

## Conflicts of interest

There are no conflicts to declare.

## Supplementary Material

SC-012-D1SC01889B-s001
