# Supplementary material for: A visible-light mediated ring opening reaction of alkylidenecyclopropanes for the generation of homopropargyl radicals
Source: Chem Sci. 2021 May 28;12(26):9088–95. doi: 10.1039/d1sc01889b (PMC8261759; doi:10.1039/d1sc01889b)
Supplement: SC-012-D1SC01889B-s001 [file SC-012-D1SC01889B-s001.pdf]

Electronic Supplementary Material (ESI) for Chemical Science.
This journal is © The Royal Society of Chemistry 2021

Supporting Information

Visible-light Mediated Ring Opening Reaction of Alkylidenecyclopropanes for the Generation of Homopropargyl Radical

Xiao-Yu Zhang,[a] Chao Ning,[a] Ben Mao,[a] Yin Wei*[b] and Min Shi*[a,b]

[a] Laboratory for Advanced Materials & Institute of Fine Chemicals, Key Laboratory for Advanced Materials and Feringa Nobel Prize Scientist Joint Research Center, School of Chemistry & Molecular Engineering, East China University of Science and Technology, 130 Meilong Road, Shanghai 200237, P. R. China, [b] State Key Laboratory of Organometallic Chemistry, Center for Excellence in Molecular Synthesis, University of Chinese Academy of Sciences, Shanghai Institute of Organic Chemistry, Chinese Academy of Sciences, 345 Lingling Road, Shanghai 200032, P. R. China. weiyin@sioc.ac.cn, mshi@mail.sioc.ac.cn

CONTENTS

1. General Remarks S2
2. General Procedure for the Synthesis of Substrates S3
   2.1 Synthesis of Alkenes S3
   2.2 Synthesis of *N*-Hydroxyphthalimide Esters (NHP esters) S5
   2.3 Synthesis of Deuterated Hantzsch Esters S6
3. General Procedure for the Synthesis of Products S8
4. Synthetic Application of the Reaction S9
   4.1 Product Transformations S9
   4.2 Synthesis of Drugs S12
5. Mechanistic Studies S18
   5.1 Cyclic Voltammetry Experiments S18
   5.2 Stern-Volmer Fluorescence Quenching Experiments S19
   5.3 Radical Trapping Experiment S20
   5.4 Deuterium Labeling Study S20
   5.5 DFT Calculations S21
6. The Characterization Data of Substrates S26
7. The Characterization Data of Products S57
8. References S191

1. General Remarks

Melting points were determined on a digital melting point apparatus and temperatures were uncorrected. NMR spectra were recorded with a Bruker spectrometer at 400 MHz (^{1}H NMR), 600 MHz (^{1}H NMR), 100 MHz (^{13}C NMR), 150 MHz (^{13}C NMR) and 376 MHz (^{19}F NMR) in $CDCl_3$, respectively. Chemical shift was reported in ppm down field from internal TMS. Organic solvents used were dried by standard methods when necessary. Commercially available reagents were used without further purification. All reactions were monitored by TLC with Huanghai GF_{254} silica gel coated plates. Flash column chromatography was carried out using 300-400 mesh silica gel at increased pressure. All reactions were performed under argon using standard Schlenk techniques if necessary. Infrared spectra were recorded on a Perkin-Elmer PE-983 spectrometer with absorption in cm^{-1}. Mass spectra were recorded by ESI and HRMS was measured on a HP-5989 instrument. The 15 W Blue LED (Manufacturer: Liangyuan-Light Factory, Model: PAR 38, Wavelength: 425 nm) was directly got from the supermarket.

2. General Procedure for the Synthesis of Substrates

2.1 Synthesis of Alkenes

Alkenes **2a-2i**, **2l-2z** and **2ae** were prepared according to the previously reported work.[1-8] Alkenes **2j-2k** and **2aa** were commercially available. Alkenes **2ab-2ad** were prepared by the following procedures.

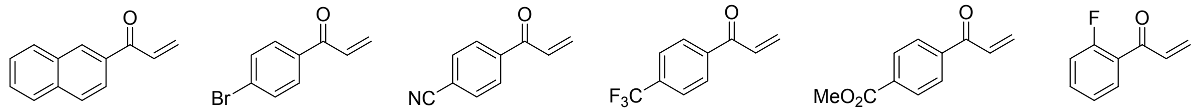

2b **2c** **2d** **2e** **2f** **2g**

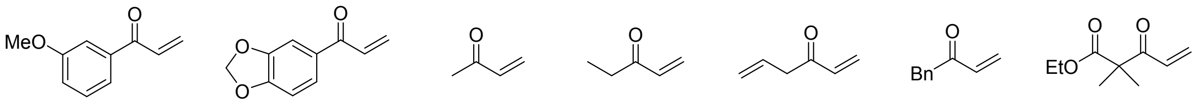

2h **2i** **2j** **2k** **2l** **2m** **2n**

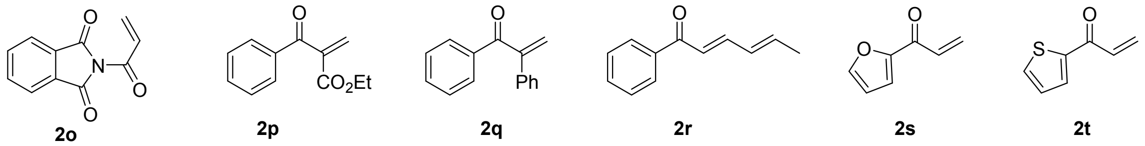

2o **2p** **2q** **2r** **2s** **2t**

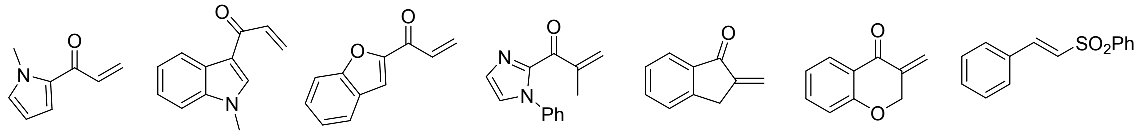

2u **2v** **2w** **2x** **2y** **2z** **2aa**

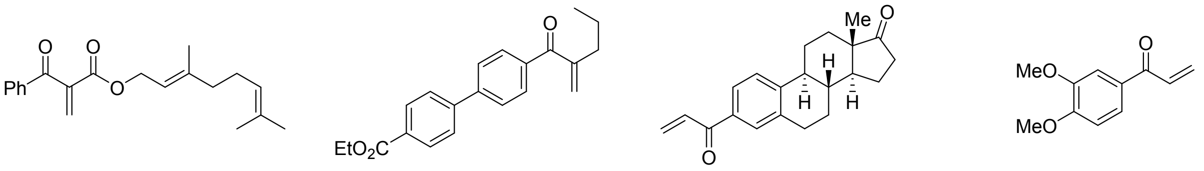

2ab **2ac** **2ad** **2ae**

Geraniol derivative **Mesogenic compound analogue** **Estrone analogue** **Methyl vanillin analogue**

Unsuccessful examples are shown as followings.

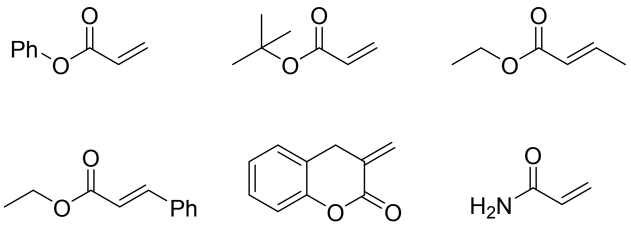

Synthesis of Geraniol Derivative 2ab

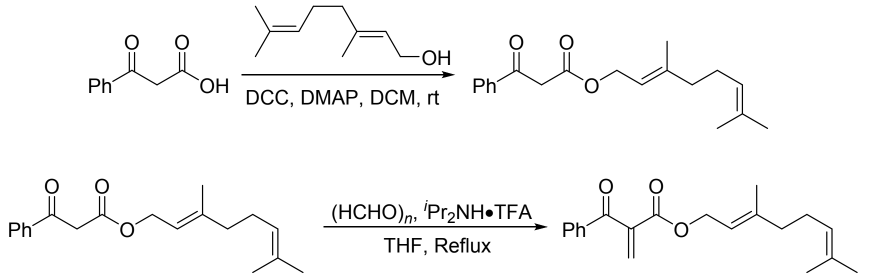

Geraniol derivative

To a solution of 3-oxo-3-phenylpropanoic acid (1.92 g, 10.0 mmol, 1.0 equiv) in a mixture of CH_2Cl_2 (10.0 mL) were added Geraniol (1.54 g, 10.0 mmol, 1.0 equiv), dicyclohexylcarbodiimide (2.20 g, 11.0 mmol, 1.1 equiv), and DMAP (122.0 mg, 1.0 mmol, 0.1 equiv) sequentially at room temperature. The reaction mixture was stirred overnight. Water was added and the reaction mixture was extracted with EtOAc three times. The combined organic layer was filtered through a short pad of silica gel and rinsed with elution (petroleum ether:ethyl acetate = 2:1). The filtrate was evaporated under reduced pressure to afford the crude product as colorless oil, which was directly used without further purification.

To a mixture of the crude product (2.0 mmol, 1.0 equiv) and paraformaldehyde (4.0 mmol, 2.0 equiv) in dry THF (10 mL) was added the corresponding diisopropylammonium 2,2,2-trifluoroacetate[8] (2.0 mmol, 1.0 equiv). The reaction mixture is stirred under reflux with oil bath for 2 h. Then, a second addition of paraformaldehyde (4.0 mmol, 2.0 equiv) is added to the reaction mixture. Next, the reaction mixture is stirred under reflux for an additional 6 h. The reaction mixture is cooled down and filtered. The filtrate was concentrated under vacuum and purified by a silica gel column chromatography (petroleum ether:ethyl acetate = 10:1) to afford the product **2ab**.

Synthesis of Mesogenic Compound Analogue 2ac

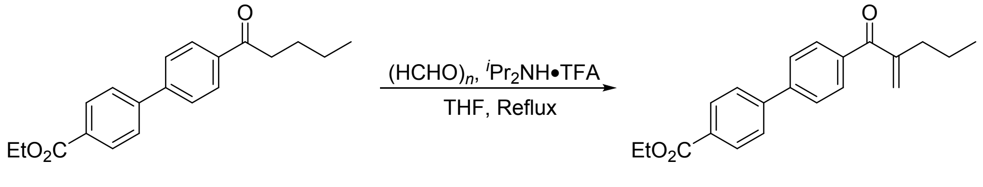

Mesogenic compound analogue

To a mixture of substrate[9] (2.0 mmol, 1.0 equiv) and paraformaldehyde (4.0 mmol, 2.0 equiv) in dry THF (10 mL) was added the corresponding diisopropylammonium 2,2,2-trifluoroacetate (2.0 mmol, 1.0 equiv). The reaction mixture was stirred under reflux with oil bath for 2 h. Then, a second portion of paraformaldehyde (4.0 mmol, 2.0 equiv) was added to the reaction mixture. Next, the

reaction mixture was stirred under reflux with oil bath for an additional 6 h. The reaction mixture was cooled down and filtered. The filtrate was concentrated under vacuum and purified by a silica gel column chromatography (petroleum ether:ethyl acetate = 10:1) to afford the product **2ac**.

Synthesis of Estrone Analogue 2ad

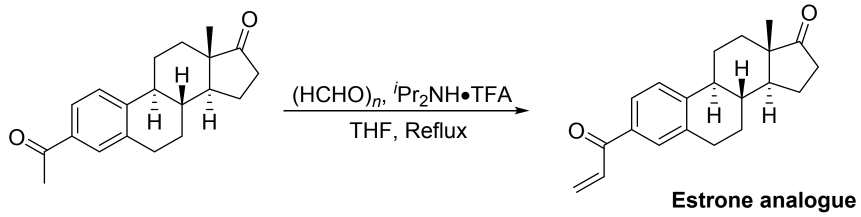

Estrone analogue

To a mixture of substrate[10] (2.0 mmol, 1.0 equiv) and paraformaldehyde (4.0 mmol, 2.0 equiv) in dry THF (10 mL) was added the corresponding diisopropylammonium 2,2,2-trifluoroacetate (2.0 mmol, 1.0 equiv). The reaction mixture was stirred under reflux with oil bath for 2 h. Then, a second portion of paraformaldehyde (4.0 mmol, 2.0 equiv) was added to the reaction mixture. Next, the reaction mixture was stirred under reflux with oil bath for an additional 6 h. The reaction mixture was cooled down and filtered. The filtrate was concentrated under vacuum and the crude product was purified by a silica gel column chromatography (petroleum ether:ethyl acetate = 30:1) to afford the final product **2ad**.

2.2 Synthesis of *N*-Hydroxyphthalimide Esters (NHP esters)

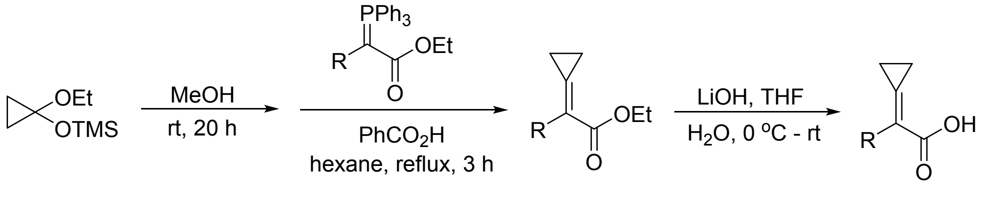

S1

This procedure was modified from the previous literature.[11-12] In a round bottom flask equipped with a magnetic stir bar 1-ethoxycyclopropoxy)trimethylsilane (1.74 g, 10 mmol, 1.0 equiv) was dissolved in methanol (4.9 mL). After stirred for 20 hours at room temperature, methanol was removed in *vacuo*. The residue was dissolved in hexane (50 mL), and the benzoic acid (244 mg, 2 mmol, 0.2 equiv) was added. This flask was brought to gentle reflux in an oil bath. To the refluxing solution was added the corresponding ylide[13] (12 mmol, 1.2 equiv) at a rate so as to maintain reflux. After complete the addition, the reaction mixture was allowed to reflux for an additional hour. After

cooling to room temperature, hexane was removed *in vacuo* and the residue was washed with consecutive petroleum ether solution until no further precipitation of triphenylphosphine oxide. Washings were then concentrated *in vacuum* and purified *via* a column chromatography (petroleum ether:ethyl acetate = 10:1) to give cyclopropylidene ethyl ester as crude colourless oil in the yields ranging from 40% to 60%.

Lithium hydroxide (2.0 equiv) dissolved in distilled water (20 mL) was added to the THF solution (20 mL) of the cyclopropylidene ethyl ester (1.0 equiv) at 0 °C with an ice bath. This mixture was stirred at room temperature and monitored by TLC analysis. After the ester was totally consumed, the reaction mixture was acidified with an aqueous solution of 20% hydrochloric acid. The aqueous solution was extracted with ether and the combined organics were dried over anhydrous sodium sulfate. This crude mixture was concentrated *in vacuo* to give the crude product as a yellow oil, which could be directly used for the next step without further purification.

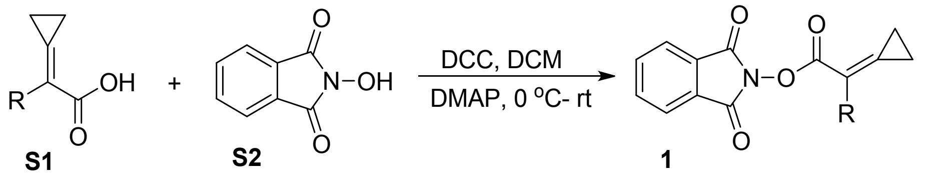

An oven-dried 100 mL round bottom flask containing a teflon-coated magnetic stir bar was charged with **S1** (1.0 equiv), *N*-hydroxyphthalimide **S2** (1.2 equiv), DMAP (0.1 equiv) and DCM under nitrogen atmosphere. The flask was cooled to 0 °C with an ice bath and DCC (1.2 equiv) in DCM was added dropwise for 15 minutes. The resulted mixture was stirred at room temperature and monitored by TLC analysis. After the **S1** was completely consumed, the heterogeneous mixture was filtered and the filtrate was concentrated under reduced pressure. The residue was purified with a flash column chromatography (petroleum ether:ethyl acetate = 10:1) to afford substrates **1** in moderate to good yields.

2.3 Synthesis of Deuterated Hantzsch Esters

4,4-dideuterio-2,6-dimethyl-3,5-dicarbethoxy-1,4-dihydropyridine

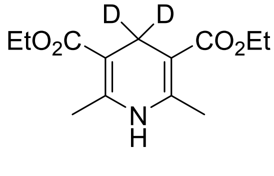

Compound **2af** was synthesized according to the previously reported literature procedure.[14] An

oven-dried round bottom flask was charged with ethyl acetoacetate (1.6 mL, 12.48 mmol, 4.0 equiv), d_2-paraformaldehyde (0.1 g, 3.12 mmol, 1.0 equiv), ammonium acetate (0.48 g, 6.24 mmol, 2.0 equiv) and water (6.5 mL), then the mixture was heated at 86 ^{o}C in an oil bath. After 3 hours, the reaction mixture was allowed to cool down to room temperature and filtered. The precipitate was dried in *vacuo* to afford the desired compound **2af** as a yellow solid.

1,4,4-trideuterio-2,6-dimethyl-3,5-dicarbethoxy-1,4-dihydropyridine

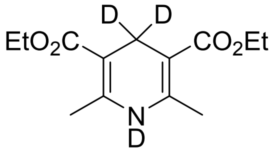

Compound **2ag** was synthesized by following the previously reported literature procedure.[15] In an oven dried round bottom flask compound **2ag** (0.1g, 0.39 mmol) and CD_3OD (1 mL) were mixed and stirred for overnight under argon atmosphere. The solvent was evaporated and the desired compound **2ag** was obtained as a pale green solid.

3. General Procedure for the Synthesis of Products

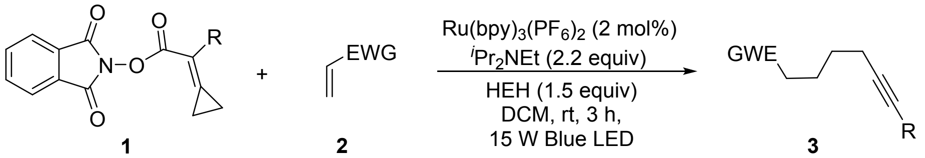

An oven-dried Schlenk tube (20 mL) was equipped with a magnetic stir bar, and substrate **1** (0.3 mmol, 1.5 equiv), **2** (0.2 mmol, 1.0 equiv), $Ru(bpy)_3(PF_6)_2$ (3.4 mg, 2 mol%), $^i Pr_2NEt$ (0.44 mmol, 2.2 equiv), Hantzsch ester (diethyl 2,6-dimethyl-1,4-dihydropyridine-3,5-dicarboxylate) (0.3 mmol, 1.5 equiv) and DCM (3.0 mL) were added. The tube was degassed by alternating vacuum evacuation (10 min) and argon backfill three times. The tube was placed at a distance (app. 5 cm) from 15 W Blue LED, and the resulting yellow solution was stirred at ambient temperature for 3 hours under visible-light irradiation. When the reaction was finished up, the mixture was evaporated in *vacuo*. The crude products were directly purified by a flash chromatography on silica gel (petroleum ether:ethyl acetate = 20:1) to give the desired products.

Reaction Setup

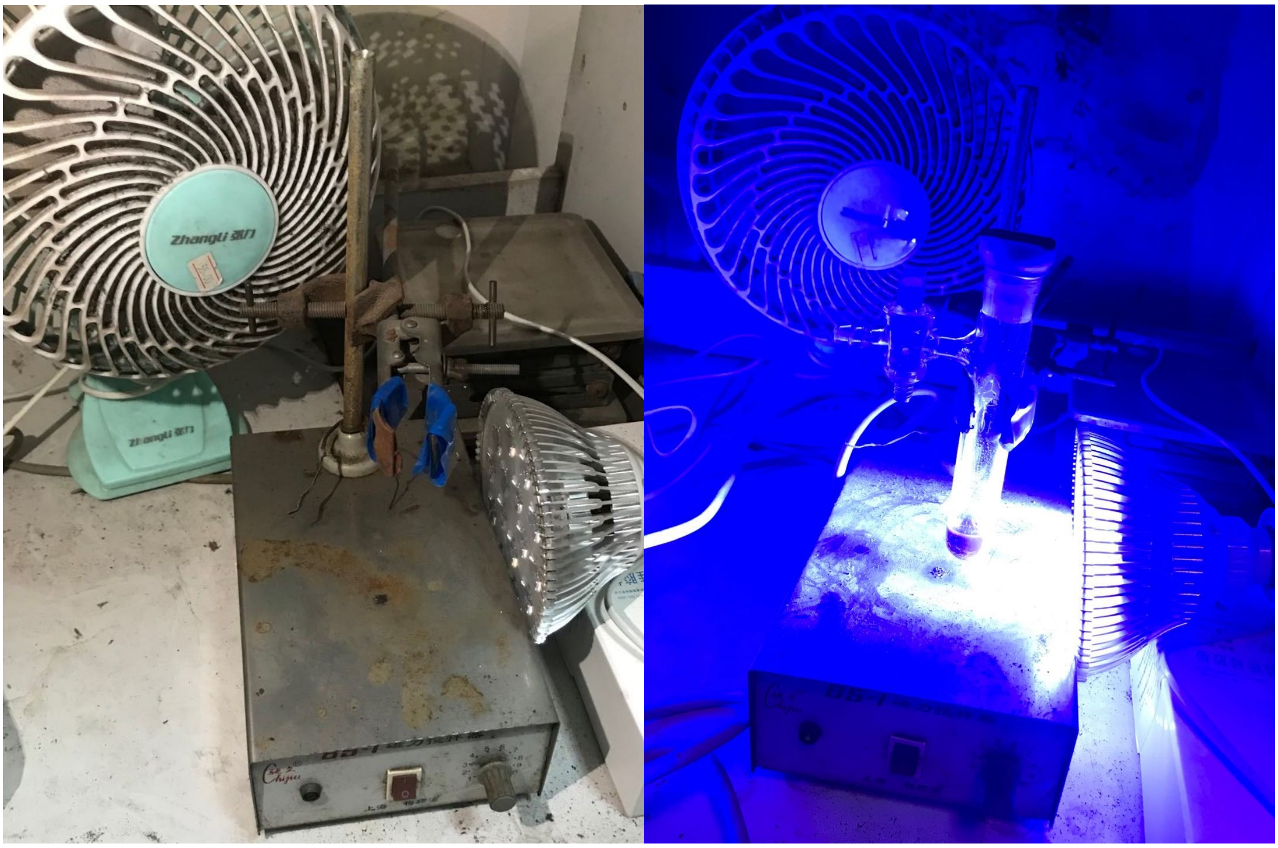

4. Synthetic Application of the Reaction

4.1 Product Transformations

Synthesis of Compound 4

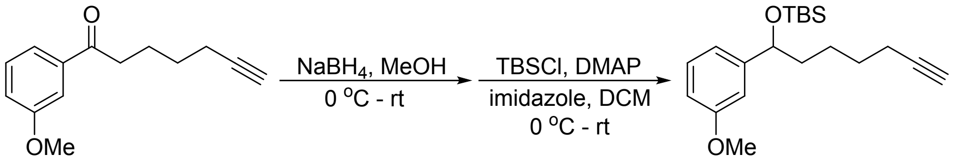

3baf **S3**

An over-dried 50 mL round bottom flask containing a teflon-coated magnetic stir bar was charged with **3baf** (216.3 mg, 1.0 mmol, 1.0 equiv) and MeOH under air. The flask was cooled to 0 °C with an ice bath and $NaBH_4$ (45.4 mg, 1.2 mmol, 1.2 equiv) was added in portions. The resulted mixture was stirred at room temperature and monitored by TLC analysis. After the substrate was completely consumed, the heterogeneous mixture was filtered and the filtrate was concentrated under reduced pressure. The residue was treated with water and DCM. The layers were separated and the water phase was extracted with DCM (10 mL) for three times. The combined organic layers were dried over anhydrous Na_2SO_4 and concentrated under reduced pressure. The crude product could be directly used for the next step without further purification.

A 50 mL round bottom flask containing a teflon-coated magnetic stir bar was charged with alcohol (174.6 mg, 0.8 mmol, 1.0 equiv), DMAP (9.8 mg, 0.08 mmol, 0.1 equiv), imidazole (59.9 mg, 0.88 mmol, 1.1 equiv) and DCM (10 mL) under argon atmosphere. The flask was cooled to 0 °C with ice bath and TBSCl (132.6 mg, 0.88 mmol, 1.1 equiv) was added dropwise. The resulted mixture was stirred at room temperature for 12 h. The heterogeneous mixture was filtered and the filtrate was concentrated under reduced pressure. The residue was purified with a flash column chromatography (petroleum ether:ethyl acetate = 10:1) to afford product **S3** in 90% yield.

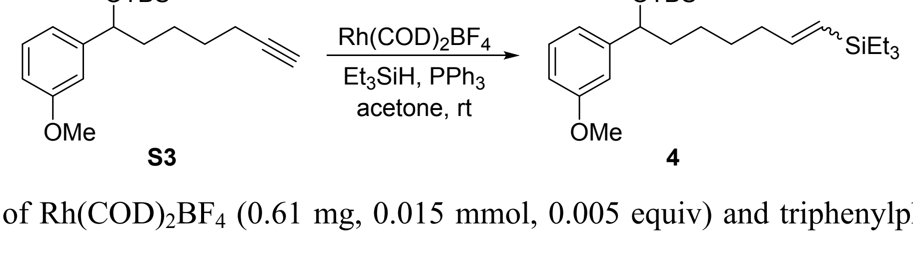

S3 **4**

To a solution of $Rh(COD)_2BF_4$ (0.61 mg, 0.015 mmol, 0.005 equiv) and triphenylphosphine (0.79 mg, 0.03 mmol, 0.01 equiv) in acetone (2 mL) was added successively **S3** (99.8 mg, 0.3 mmol, 1.0 equiv) and Et_3SiH (52.4 mg, 0.45 mmol, 1.5 equiv) at room temperature. After being stirred for 20 h, the solvent was evaporated and the residue was purified by a column chromatography on silica

gel (petroleum ether:ethyl acetate = 20:1) to afford product **4** in 87% yield.

Synthesis of Compound 5

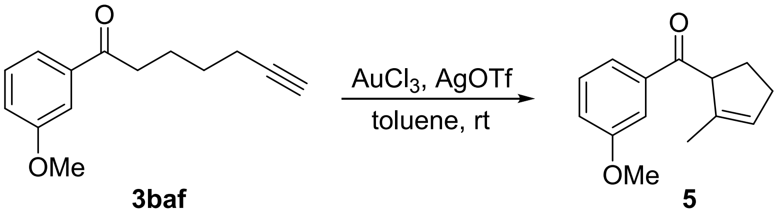

In an oven-dried 10 mL Schlenk tube was equipped with a magnetic stir bar, substrate **3baf** (64.9 mg, 0.3 mmol, 1.0 equiv), $AuCl_3$ (4.5 mg, 0.015 mmol, 0.05 equiv), AgOTf (11.6 mg, 0.045 mmol, 0.15 equiv) and toluene (2 mL) were added. The resulting mixture was stirred vigorously at room temperature for 8 h. After that, the mixture was concentrated under reduced pressure. The residue was purified by a flash column chromatography (petroleum ether:ethyl acetate = 20:1) on silica gel to afford **5** as a pale oil (90% yield).

Synthesis of Compound 6

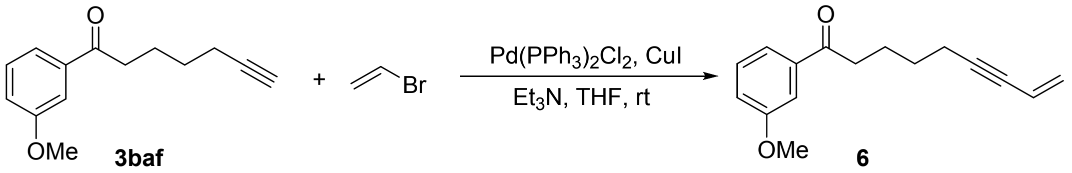

In an oven-dried 20 mL Schlenk tube was equipped with a magnetic stir bar, $Pd(PPh_3)_2Cl_2$ (2.1 mg, 0.003 mmol, 0.01 equiv), CuI (2.6 mg, 0.012 mmol, 0.04 equiv) were added. The mixture was evacuated and backfilled with argon for three time, and the flask was equipped with a balloon filled with argon. A solution of substrate **3baf** (64.9 mg, 0.3 mmol, 1.0 equiv) in THF (2.5 mL) was added *via* a syringe, followed by the addition of 2-vinylbromide (0.54 mL, 0.54 mmol, 1.0 M solution in THF, 1.8 equiv), anhydrous degassed Et_3N (0.1 mL, 0.69 mmol, 2.3 equiv). The Schlenk tube was sealed with a screw-cap and stirred at room temperature. After stirring for 12 h, the reaction mixture was cooled to room temperature and concentrated. The residue was purified by a flash column chromatography (petroleum ether:ethyl acetate = 10:1) on silica gel to give product **6** as colorless oil (99% yield).

Synthesis of Compound 7

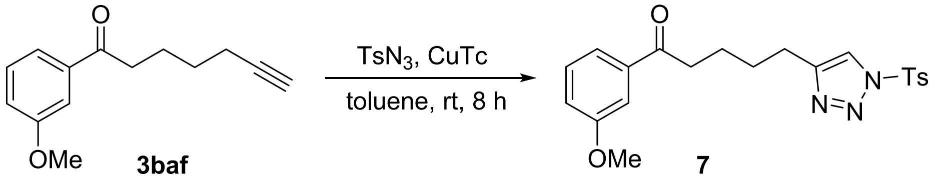

In an oven-dried 10 mL Schlenk tube was equipped with a magnetic stir bar, substrate **3baf** (108.2 mg, 0.5 mmol, 1.0 equiv), CuTc (9.54 mg, 0.05 mmol, 0.1 equiv) and toluene (5 mL) were added. After 10 minutes, TsN_3 (118.4 mg, 0.6 mmol, 1.2 equiv) was added in one portion. The resulting mixture was stirred vigorously at room temperature for 8 hours. After that, the mixture was concentrated under reduced pressure. The residue was purified by a flash column chromatography (petroleum ether:ethyl acetate = 4:1) on silica gel to afford **7** as white solid (95% yield).

Synthesis of Compound 8

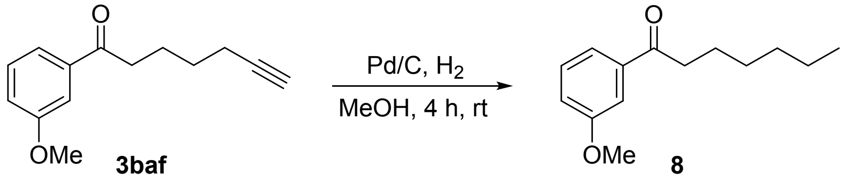

To a flask was added compound **3baf** (64.9 mg, 0.3 mmol, 1.0 equiv), Pd/C (32 mg) and menthol (8 mL). After the reaction mixture was stirred at room temperature for 4 hours under 1 atm H_2, the resulting mixture was filtered with a pad of celite. The filtrate was concentrated. The residue was purified by flash column chromatography (petroleum ether) on silica gel to give product **8** as a colorless oil (98% yield).

Synthesis of Compound 9

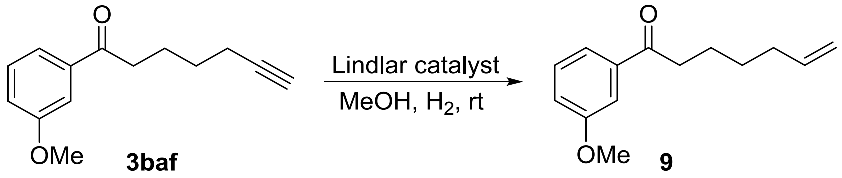

To a flask was added substrate **3baf** (64.9 mg, 0.3 mmol, 1.0 equiv), Lindlar catalyst (32 mg) and MeOH (8 mL). After the reaction mixture was stirred at room temperature for 8 h under 1.0 atm H_2, the resulting mixture was filtered with a pad of celite. The filtrate was concentrated under reduced pressure and the residue was purified with a silica gel chromatography (petroleum ether:ethyl acetate = 20:1) to give product **9** as a colorless oil (95% yield).

4.2 Synthesis of Drugs

Synthesis of 10 (Precursor of Fingolimod)

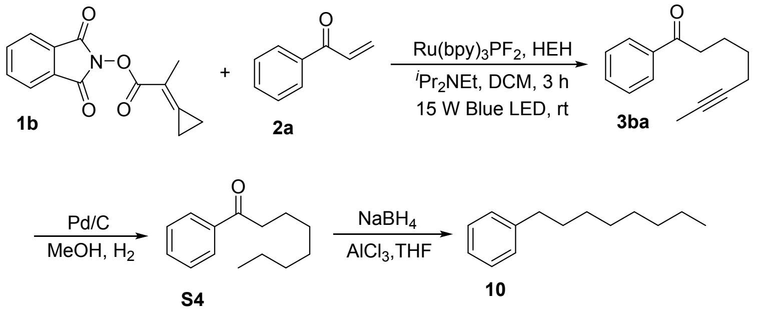

1b 2a 3ba S4 10

An oven-dried Schlenk tube (200 mL) was equipped with a magnetic stir bar, and substrates **1b** (3.09 g, 12.0 mmol, 1.5 equiv), **2a** (1.06 g, 8.0 mmol, 1.0 equiv), $Ru(bpy)_3(PF_6)_2$ (136.0 mg, 2 mol%), ^{i}Pr$_2$NEt (2.23 g, 17.6 mmol, 2.2 equiv), Hantzsch ester (diethyl 2,6-dimethyl-1,4-dihydropyridine-3,5-dicarboxylate) (3.02 g, 12.0 mmol, 1.5 equiv) and DCM (120 mL) were added. The tube was degassed by alternating vacuum evacuation (10 min) and argon backfill three times. The tube was placed at a distance (app. 5 cm) from 15 W Blue LED, and the resulting yellow solution was stirred at ambient temperature under visible-light irradiation. When the reaction finished, the mixture was evaporated in *vacuo*. The crude products were directly purified by flash chromatography on silica gel (petroleum ether:ethyl acetate = 20:1) to give the desired products **3ba** (75% yield).

To a flask was added compound **3ba** (1.2 g, 6.0 mmol, 1.0 equiv), Pd/C (600 mg) and menthol (100 mL). After the reaction mixture was stirred at room temperature for 4 h under 1 atm H_2, the resulting mixture was filtered. The filtrate was concentrated. The residue was purified by flash column chromatography (petroleum ether) on silica gel to give product **S4** as a colorless oil (98% yield).

An over-dried 20 mL round bottom flask containing a teflon-coated magnetic stir bar was charged with product **S4** (1.02 g, 5.0 mmol, 1.0 equiv), $AlCl_3$ (1.0 g, 15.0 mmol, 3.0 equiv) and THF (100 mL) under air. The flask was cooled to 0 ^{o}C with ice bath and $NaBH_4$ (0.57 g, 15.0 mmol, 3.0 equiv) was added within 1 hour. The reaction mixture was stirred for 2 h at 0 °C with ice bath and continued to be stirred for another 16 h at 65 °C in oil bath. After completion of reaction, the heterogeneous mixture was concentrated under reduced pressure. The residue was quenched in mixture of water (150 mL) and hydrochloric acid (25 mL) at room temperature. The layers were

separated and the water phase was extracted with toluene (50 mL) for three times. The combined organic layers were dried over anhydrous Na_2SO_4 and concentrated under reduced pressure. The residue was purified by flash column chromatography (petroleum ether) on silica gel to give product **10** as a colorless liquid (65% yield).

Synthesis of 13 and 14 (Anti-HIV-1 Activity)

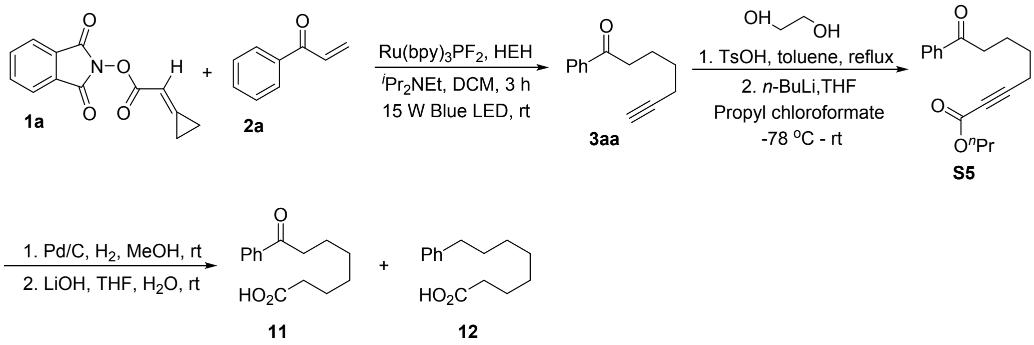

An oven-dried Schlenk tube (200 mL) was equipped with a magnetic stir bar, and substrate **1b** (2.90 g, 12.0 mmol, 1.5 equiv), **2a** (1.06 g, 8.0 mmol, 1.0 equiv), $Ru(bpy)_3(PF_6)_2$ (136.0 mg, 2 mol%), $^iPr_2NEt$ (2.28 g, 17.6 mmol, 2.2 equiv), Hantzsch ester (diethyl 2,6-dimethyl-1,4-dihydropyridine-3,5-dicarboxylate) (3.02 g, 12.0 mmol, 1.5 equiv) and DCM (120 mL) were added. The tube was degassed by alternating vacuum evacuation (10 min) and argon backfill three times. The tube was placed at a distance (app. 5 cm) from 15 W Blue LED, and the resulting yellow solution was stirred at ambient temperature under visible-light irradiation. When the reaction was finished up, the mixture was evaporated in *vacuo*. The crude product was directly purified by a flash chromatography on silica gel (petroleum ether:ethyl acetate = 20:1) to give the desired product **3aa** (60% yield).

To a flask was added compound **3aa** (838.2 mg, 4.5 mmol, 1.0 equiv), ethylene glycol (297.3 mg, 4.95 mmol, 1.1 equiv), *p*-toluenesulfonic acid (77.4 mg, 0.45 mmol, 0.1 equiv) and toluene (60 mL). After the reaction mixture was stirred at 110 °C in an oil bath and monitored by TLC analysis. The resulting mixture was concentrated in vacuum and washed with sodium bicarbonates. The mixture was extracted with EtOAc and the combined organics was dried over anhydrous Na_2SO_4. The solvent was evaporated and the residue was concentrated in vacuum to afford crude product as a colorless oil. To a solution of alkyne (4.5 mmol, 1.0 equiv) in THF (20 mL) was added dropwise, at -78 °C, a 1.0 M solution of *n*-BuLi (5.4 mL, 5.4 mmol, 1.2 equiv) in hexane. The reaction

mixture was stirred at -78 °C during 30 min and then the solution of propyl chloroformate (1.66 g, 13.5 mmol, 3.0 equiv) in THF (15 mL) was added in one portion. After being stirred for 30 min at -78 °C and overnight at room temperature, the reaction mixture was hydrolyzed with saturated solution of NH_4Cl (30 mL) and extracted with Et_2O (3 x 20 mL). The combined organic phases were washed with brine (50 mL), dried over anhydrous Na_2SO_4, filtered and concentrated under reduced pressure to afford crude product. The crude products were directly purified by a flash chromatography on silica gel (petroleum ether:ethyl acetate = 20:1) to give the desired product **S5** (91% yield).

To a solution of the product **S5** (1.73 g) in THF (80 mL) and water (80 mL) was added 1.0 M HCl (60 mL) and the solution was stirred at room temperature for 4 h. The reaction mixture was extracted with Et_2O (3 x 40 mL). The combined organic phases were washed with brine (20 mL), dried over anhydrous Na_2SO_4, filtered and concentrated under reduced pressure to afford the crude product. To a flask was added the crude product, Pd/C (864 mg) and methanol (60 mL). After the reaction mixture was stirred at room temperature for overnight under 1.0 atm H_2, the resulting mixture was filtered. The filtrate was concentrated under reduced pressure and the residue was dissolved in THF (50 mL), and lithium hydroxide (2.0 equiv) dissolved in distilled water (50 mL) was added to the THF solution. This mixture in the flask was stirred at room temperature and monitored by TLC analysis. After the ester was totally consumed, the reaction mixture was acidified with 20% hydrochloric acid aqueous solution. The aqueous solution was extracted with ether and the combined organics were dried over anhydrous Na_2SO_4. The solvent was evaporated and the residue was purified by a column chromatography on silica gel (petroleum ether:ethyl acetate = 1:1) to afford the corresponding carboxylic acids **11** and **12**.

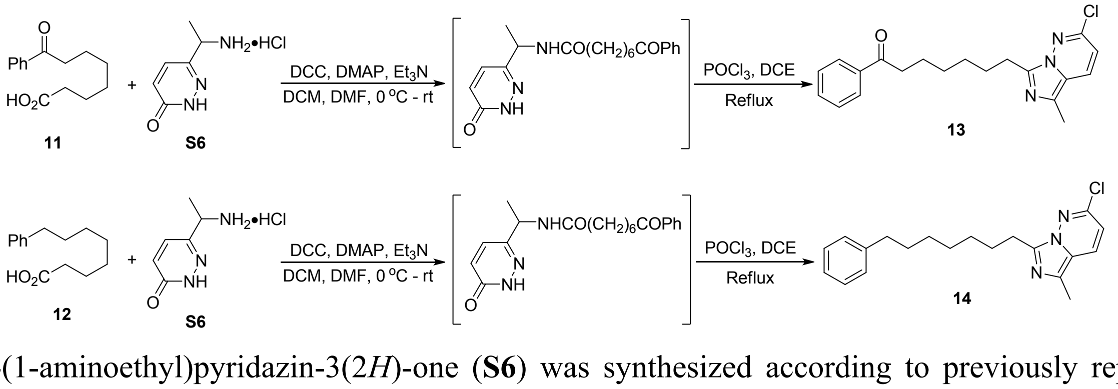

The 6-(1-aminoethyl)pyridazin-3(2*H*)-one (**S6**) was synthesized according to previously reported literature procedures and the analytic data were consistent with those in literature.[9]

An oven-dried 25 round bottom flask containing a teflon-coated magnetic stir bar was charged with the corresponding carboxylic acid (3.0 mmol, 1.0 equiv), **S6** (3.6 mmol, 1.2 equiv), DMAP (0.3 mmol, 0.1 equiv), Et_3N (3.6 mmol, 1.2 equiv), DCM and DMF under nitrogen atmosphere. The flask was cooled to 0 ^oC with an ice bath and DCC (3.6 mmol, 1.2 equiv) in DCM was added dropwise. The resulted mixture was stirred at room temperature and monitored by TLC analysis. After the carboxylic acid was completely consumed, the heterogeneous mixture was filtered and the filtrate was washed with water and extracted with DCM (3 x 20 mL). The organic solutions were combined and dried over anhydrous Na_2SO_4. The solvent was concentrated under reduced pressure to afford the crude product. An oven-dried Schlenk tube (25 mL) was charged with a suspension of crude product in 1,2-dichloroethane (40 mL) and phosphoryl chloride (12.0 mmol, 4.0 equiv). The tube was heated to reflux with stirring for 3 h and then left at room temperature for 18 h. The resulting solution was evaporated, and the residual oil was suspended in ethyl acetate (50 mL). This suspension was cooled to 0 °C with an ice bath and quenched by sodium bicarbonate saturated solution. The aqueous phase was separated and extracted with ethyl acetate (2 x 20 mL). The organic solutions were combined and dried over anhydrous Na_2SO_4. The solvent was concentrated in vacuum and the residue was purified by a column chromatography on silica gel (petroleum ether:ethyl acetate = 1:1) to afford the corresponding products **13** and **14** in 64% and 62% yields.

Synthesis of 25 (Precursor of Tramadol)

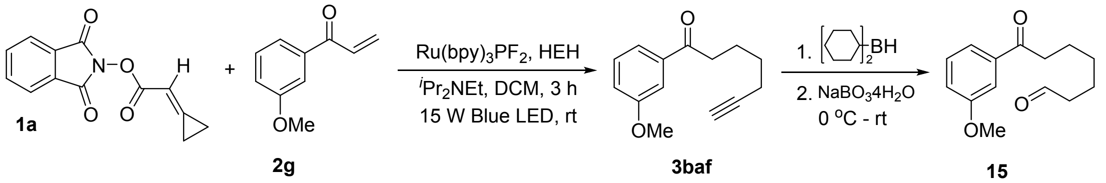

An oven-dried Schlenk tube (200 mL) was equipped with a magnetic stir bar, and substrate **1a** (2.91 g, 12.0 mmol, 1.5 equiv), alkene **2g** (1.30 g, 8.0 mmol, 1.0 equiv), $Ru(bpy)_3(PF_6)_2$ (136.0 mg, 2 mol%), $^iPr_2NEt$ (2.28 g, 17.6 mmol, 2.2 equiv), Hantzsch ester (diethyl 2,6-dimethyl-1,4-dihydropyridine-3,5-dicarboxylate) (3.02 g, 12.0 mmol, 1.5 equiv) and DCM (120 mL) were added. The tube was degassed by alternating vacuum evacuation (10 min) and argon backfill three times. The tube was placed at a distance (app. 5 cm) from 15 W Blue LED, and the resulting yellow solution was stirred at ambient temperature under visible-light irradiation. When the reaction was finished up, the mixture was evaporated in *vacuo*. The crude product was directly purified by a flash

chromatography on silica gel (petroleum ether:ethyl acetate = 20:1) to give the desired product **3baf** (68% yield).

$BH_3 \cdot THF$ (5.0 mL of a 1.0 M solution in THF, 5.0 mmol, 1.0 equiv) was placed in a dry, argon-flushed flask, which was then immersed in an ice-water bath. Cyclohexene (0.85 g, 10.0 mmol, 1.05 mL, 2.0 equiv) was added dropwise and the mixture was stirred at 0 °C with an ice bath for 1 h. Substrate **3baf** (811.0 mg, 5.0 mmol, 1.0 equiv) was then added to the slurry of dicyclohexylborane in THF. The cooling bath was removed and the reaction mixture was stirred for 1 h at room temperature. $NaBO_3 \cdot 4H_2O$ (1.38 g, 15.0 mmol, 3.0 equiv) and water (10 mL) was added to the mixture and stirred for 2 h at room temperature. The reaction mixture was extracted with ether (2 x 20 mL). The organic solutions were combined and dried over anhydrous Na_2SO_4. The solvent was concentrated in vacuum and the residue was purified by a column chromatography on silica gel (petroleum ether:ethyl acetate = 10:1) to afford the desired product **15** in 76% yield.

Synthesis of 16 (Precursor of THC/AEA Hybrid Ligands)

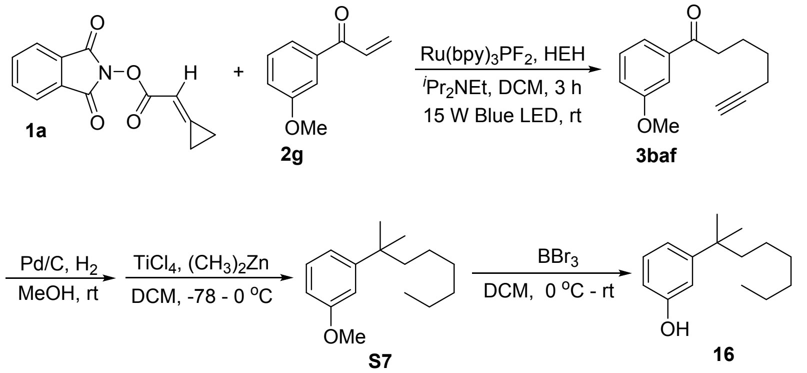

An oven-dried Schlenk tube (200 mL) was equipped with a magnetic stir bar, and substrate **1a** (2.91 g, 12.0 mmol, 1.5 equiv), alkene **2g** (1.30 g, 8.0 mmol, 1.0 equiv), $Ru(bpy)_3(PF_6)_2$ (136.0 mg, 2 mol%), $^iPr_2NEt$ (2.28 g, 17.6 mmol, 2.2 equiv), Hantzsch ester (diethyl 2,6-dimethyl-1,4-dihydropyridine-3,5-dicarboxylate) (3.02 g, 12.0 mmol, 1.5 equiv) and DCM (120 mL) were added. The tube was degassed by alternating vacuum evacuation (10 min) and argon backfill three times. The tube was placed at a distance (app. 5 cm) from 15 W Blue LED, and the resulting yellow solution was stirred at ambient temperature under visible-light irradiation. When the reaction was finished up, the mixture was evaporated in *vacuo*. The crude product was directly purified by a flash chromatography on silica gel (petroleum ether:ethyl acetate = 20:1) to give the desired product **3baf** (67% yield).

To a flask was added the substrate **3baf** (1.08 g, 5.0 mmol, 1.0 equiv), Pd/C (550 mg) and methanol (60 mL). After the reaction mixture was stirred at room temperature for 4 h under 1.0 atm H_2, the resulting mixture was filtered. The filtrate was concentrated under reduced pressure to afford the crude product. A 1.0 M solution of $TiCl_4$ (10.5 mL, 2.1 equiv) in CH_2Cl_2 was added to CH_2Cl_2 (60 mL) placed in a round bottom flask maintaining the temperature at -50 ^oC under N_2. This reaction vessel was followed by addition of 1.0 M $(CH_3)_2Zn$ (10.5 mL, 2.1 equiv) slowly. The orange-brown solution obtained was stirred vigorously at -50 ^oC for 1 hour after which a solution of the crude product (5.0 mmol, 1.0 equiv) in dry CH_2Cl_2 (50 mL) was added dropwise. The reaction solution was allowed to stir for 2 h at -50 ^oC, then allowed to reach -10 ^oC before quenching by addition of ice-cold saturated NH_4Cl solution dropwise. The reaction mixture was extracted with DCM (2 x 30 mL). The organic solutions were combined and dried over anhydrous Na_2SO_4. The solvent was concentrated in vacuum and the residue was purified by a column chromatography on silica gel (petroleum ether:ethyl acetate = 10:1) to afford product **S7** in 90% yield.

To a solution of BBr_3 (1.0 M in CH_2Cl_2, 3.3 mL, 3.3 mmol, 1.1 equiv) cooled to 0 ^oC with an ice bath was added a solution of substrate **S7** (3.0 mmol, 1.0 equiv) in CH_2Cl_2 (25 mL) dropwise. The reaction was allowed to stir 12 h at room temperature. The reaction solution was cooled to 0 ^oC with an ice bath and water (10 mL) was added dropwise. The aqueous layer was then extracted with EtOAc, and the organic extracts were combined, dried and concentrated in *vacuo*. The residue was purified by a column chromatography on silica gel (petroleum ether:ethyl acetate = 4:1) to afford product **16** in 79% yield.

5. Mechanistic Studies

5.1 Cyclic Voltammetry Experiments

Cyclic Voltammetry was performed on a CH Instrument with an Electrochemical Workstation model CHI1220B. A solution of the sample in MeCN (0.001 M) was tested with 0.1 M Bu_4NPF_6 as the supporting electrolyte, using a glassy carbon as the working electrode, a Pt as the counter electrode, and a saturated calomel electrode reference electrode. Scan rate = 0.1 V/s, 2 sweep segments, a sample interval of 0.001 V.

5.1.1 Reductive Potential of Substrate

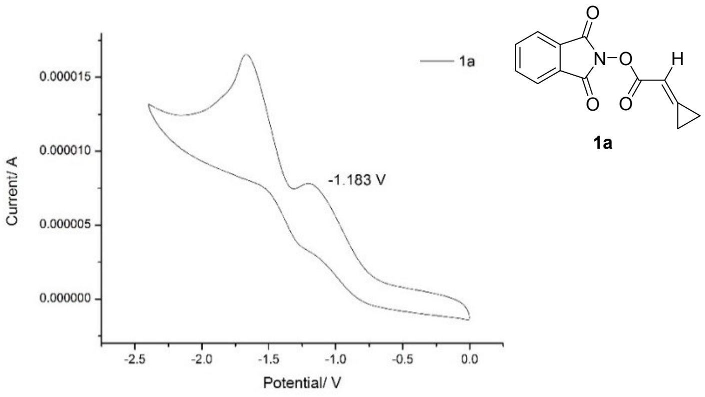

Figure S1. Cyclic Voltammogram of 1a

$E_p^{0/-1}$ (**1a**) = -1.18V (vs SCE)

5.1.2 Reductive Potential of Radical Clock Substrate

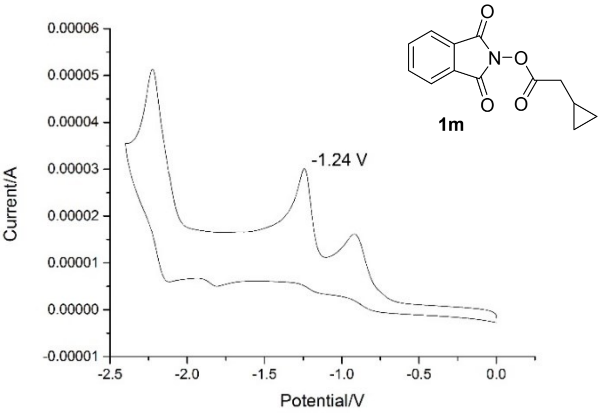

Figure S2. Cyclic Voltammogram of Radical Clock

$E_p^{0/-1}$ (**1m**) = -1.24V (vs SCE)

5.2 Stern-Volmer Fluorescence Quenching Experiments

Emission intensities were recorded using Varian Cary Eclipse spectrometer for all experiments. All $Ru(bpy)_3(PF_6)_2$ solutions were excited at 460 nm and the emission intensity was collected at 600 nm. In a typical experiment, the solution of $Ru(bpy)_3(PF_6)_2$ in DCM (15 μM) was added the appropriate amount of quencher in a screw-top 1.0 cm quartz cuvette. After degassing with nitrogen for 10 min, the emission spectra of the samples were collected.

The fluorescence intensity was measured in the presence of different four concentrations (5 mM, 10 mM, 15 mM, 20 mM of **1b**) to obtain a set of corresponding concentration fluorescence quenching spectra.

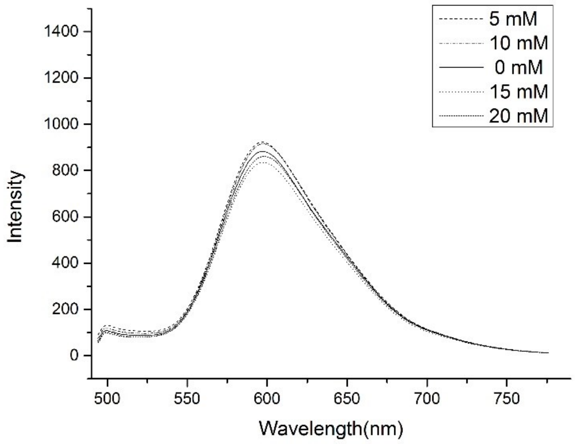

Figure S3. Fluorescence quenching of $Ru(bpy)_3(PF_6)_2$ by various concentrations of 1b.

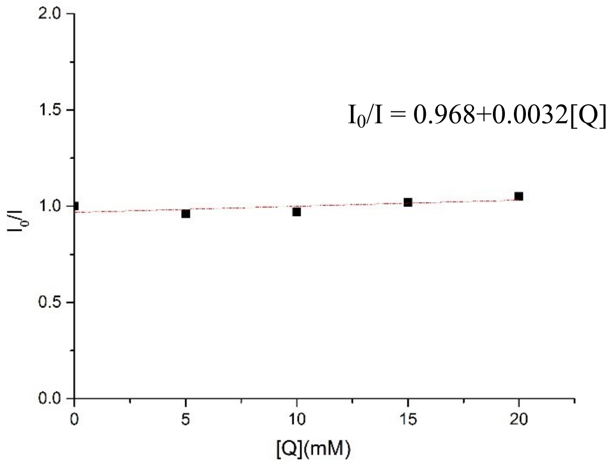

Figure S4. Stern-Volmer plots for the fluorescence quenching of $Ru(bpy)_3(PF_6)_2$ by various concentrations of 1b.

5.3 Radical Trapping Experiment

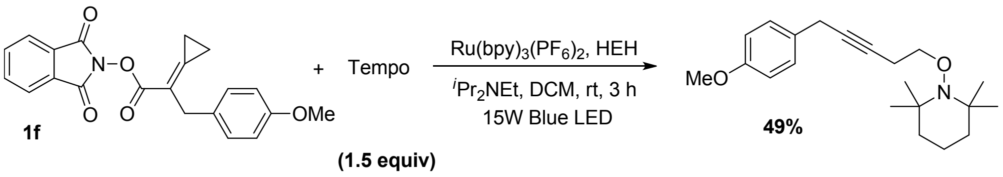

An oven-dried Schlenk tube (10 mL) was equipped with a magnetic stir bar, and substrate **1f** (0.1 mmol, 1.0 equiv), TEMPO (0.15 mmol, 1.5 equiv), $Ru(bpy)_3(PF_6)_2$ (1.7 mg, 2 mol%), $^{i}Pr_2NEt$ (0.22 mmol, 2.2 equiv), Hantzsch ester (0.15 mmol, 1.5 equiv) and DCM (1.5 mL) were added. The tube was degassed by alternating vacuum evacuation (10 min) and argon backfill three times. The tube was placed at a distance (app. 5 cm) from 15 W Blue LED, and the resulting yellow solution was stirred at ambient temperature for 3 hours under visible-light irradiation. When the reaction was finished up, the mixture was evaporated in *vacuo* and the crude product was directly purified by a flash chromatography on silica gel (petroleum ether:ethyl acetate = 20:1) to give the desired product **17** in 49% yield.

5.4 Deuterium Labeling Study

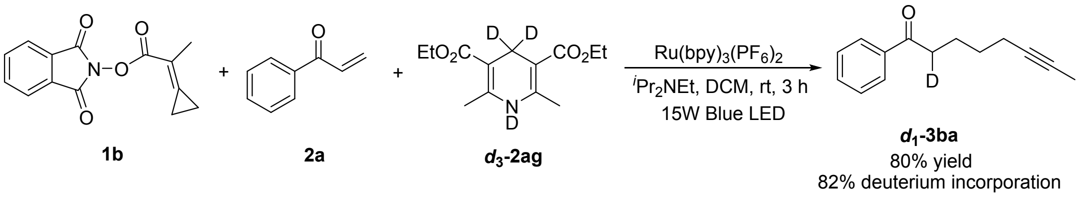

An oven-dried Schlenk tube (10 mL) was equipped with a magnetic stir bar, and substrate **1b** (0.15 mmol, 1.5 equiv), **2a** (0.1 mmol, 1.0 equiv), $Ru(bpy)_3(PF_6)_2$ (1.7 mg, 2 mol%), $^{i}Pr_2NEt$ (0.22 mmol, 2.2 equiv), 1,4,4-trideuterio-2,6-dimethyl-3,5-dicarbethoxy-1,4-dihydropyridine (0.15 mmol, 1.5 equiv) and DCM (1.5 mL) were added. The reaction tube was degassed by alternating vacuum evacuation (10 mins) and argon backfill three times. The tube was placed at a distance (app. 5 cm) from 15 W Blue LED, and the resulting yellow solution was stirred at ambient temperature for 3 hours under visible-light irradiation. When the reaction was finished up, the mixture was evaporated in *vacuo*. The crude product was directly purified by a flash chromatography on silica gel (petroleum ether:ethyl acetate = 20:1) to give the desired product in 80% yield with 82% deuterium incorporation.

5.5 Rate Measuring Experiment

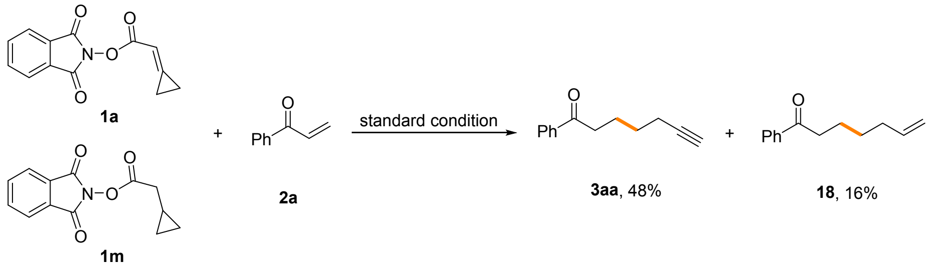

An oven-dried Schlenk tube (10 mL) was equipped with a magnetic stir bar, and substrate **1a** (0.15 mmol, 1.5 equiv), radical clock substrate **1m** (0.15 mmol, 1.5 equiv), **2a** (0.1 mmol, 1.0 equiv), $Ru(bpy)_3(PF_6)_2$ (1.7 mg, 2 mol%), $^i Pr_2NEt$ (0.22 mmol, 2.2 equiv), Hantzsch ester (0.15 mmol, 1.5 equiv) and DCM (1.5 mL) were added. The reaction tube was degassed by alternating vacuum evacuation (10 min) and argon backfill three times. The tube was placed at a distance (app. 5 cm) from 15 W Blue LED, and the resulting yellow solution was stirred at ambient temperature for 3 hours under visible-light irradiation. When the reaction was finished up, the mixture was evaporated in *vacuo*. The crude product was directly purified by a flash chromatography on silica gel (petroleum ether:ethyl acetate = 20:1) to give the corresponding products **3aa** and **18** in 48% and 16% yield, respectively.

5.6 DFT Calculation

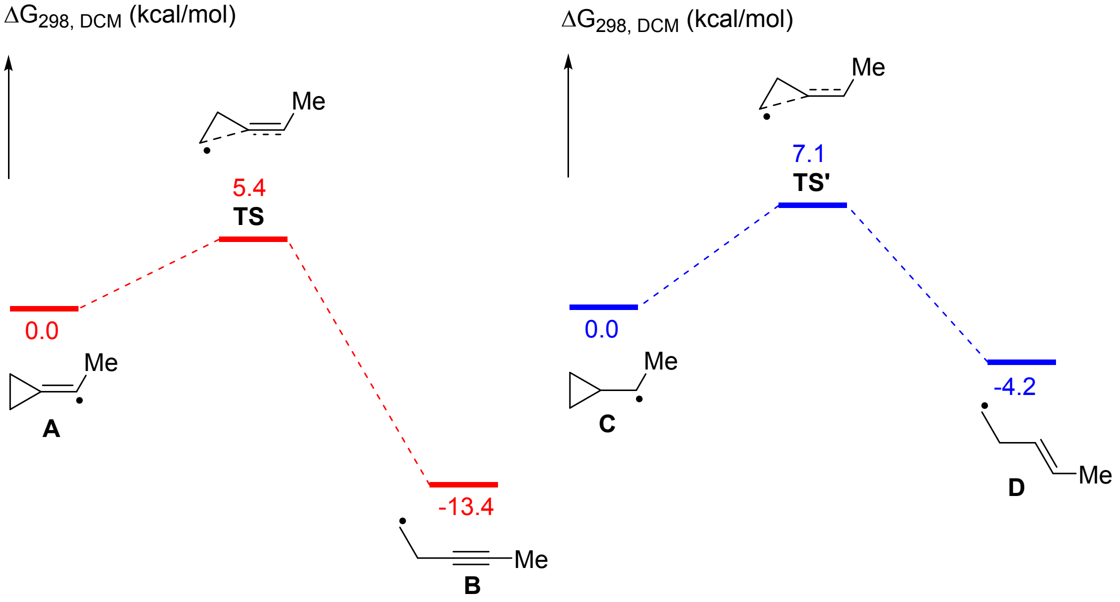

Calculated at SMD/B3LYP/6-311+G(d,p)//B3LYP/6-31G(d) level

Computational methods

All DFT calculations were performed with Gaussian 16 program. The geometries of all minima and transition states have been optimized at B3LYP/6-31G(d) level of theory. The subsequent frequency calculations on the stationary points were carried out at the same level of theory to ascertain the nature of the stationary points as minima or first-order saddle points on the respective potential energy surfaces. All transition states were characterized by one and only one imaginary frequency pertaining to the desired reaction coordinate. Thermochemical corrections to 298.15 K have been calculated for all minima from unscaled vibrational frequencies obtained at this same level. The solvent effect was estimated by the IEFPCM method with radii and nonelectrostatic terms for SMD salvation model in DCM ($\varepsilon = 8.93$). Solution-phase single point energy calculations were performed at B3LYP/6-311+G(d,p) level based on the gas phase optimized structures.

Table S1.[a]

	E_{tot} (E_h)	$H_{298, DCM}$	$G_{298, DCM}$
**A**	-194.6709033	-194.5633859	-194.5997509
**TS**	-194.6593820	-194.5539814	-194.5911144
**B**	-194.6860918	-194.5797843	-194.6210893
**C**	-195.9284328	-195.7973513	-195.8351513
**TS'**	-195.9163422	-195.7872011	-195.8237891
**D**	-195.9330182	-195.8029705	-195.8417785

a. Calculated at SMD/B3LYP/6-311+G(d,p)//B3LYP/6-31G(d)

Archive Entries

A

```
1\1\GINC-OM103\SP\UB3LYP\6-311+G(d,p)\C5H7(2)\WEIY\25-Apr-2020\0\\#p b
 3lyp/6-311+g(d,p) geom=check scrf=(iefpcm,smd,solvent=dichloromethane)
 \\Title Card Required\\0,2\C,0,-1.8015103967,-0.6361833559,0.064258800
 8\C,0,-2.2365467733,-1.5471127388,0.8740878151\C,0,-3.1497441186,-1.94
 65619175,1.9950045905\C,0,-2.1833857468,-2.942699694,1.3651627155\H,0,
 -4.2121917862,-2.0519010189,1.7807282703\H,0,-2.9288125988,-1.57491281
 47,2.9944698109\H,0,-1.3159285465,-3.2532021148,1.9464339871\H,0,-2.59
 79285148,-3.7280184829,0.7340630175\C,0,-1.9898352882,0.7825796741,-0.
 2887698836\H,0,-1.0611639168,1.3533400601,-0.1609574771\H,0,-2.2975005
 751,0.8973786478,-1.3359395216\H,0,-2.7621436281,1.2545423554,0.340494
 0444\\Version=ES64L-G16RevA.03\State=2-A\HF=-194.6709033\S2=0.758941\S
 2-1=0.\S2A=0.750053\RMSD=1.356e-09\Dipole=-0.3607419,0.1665972,0.31750
 58\Quadrupole=-1.2548098,1.9123359,-0.6575261,0.3350373,0.4613617,-1.3
 649166\PG=C01 [X(C5H7)]\\@
```

TS

```
1\1\GINC-OM103\SP\UB3LYP\6-311+G(d,p)\C5H7(2)\WEIY\25-Apr-2020\0\\#p b
 3lyp/6-311+g(d,p) geom=check scrf=(iefpcm,smd,solvent=dichloromethane)
 \\Title Card Required\\0,2\C,0,-0.1820869968,-0.0147538332,0.006615427
 4\C,0,0.0102837667,-0.0104361982,1.2383666907\C,0,1.4357259174,0.02299
 895,2.4053925037\C,0,-0.0356710622,-0.0117805547,2.6876159398\H,0,1.96
 53957617,0.9674985942,2.3910890209\H,0,2.0093314989,-0.8954760824,2.39
 07043686\H,0,-0.4205599243,-0.922366571,3.1555320551\H,0,-0.4630501867
 ,0.8795171572,3.1557877765\C,0,0.1543835021,-0.0066244787,-1.417916646
 1\H,0,-0.2367350485,-0.8983019254,-1.9241015681\H,0,-0.2776732425,0.86
 61879864,-1.9236949111\H,0,1.242841954,0.0186601433,-1.5829089357\\Ver
 sion=ES64L-G16RevA.03\State=2-A\HF=-194.659382\S2=0.770218\S2-1=0.\S2A
```

=0.75015\RMSD=4.287e-09\Dipole=0.2643472,0.0062114,0.0409627\Quadrupol
e=-2.2483136,-0.8233953,3.0717089,-0.0333946,-0.8983769,-0.0217909\PG=
C01 [X(C5H7)]\\@

B

1\1\GINC-OM103\SP\UB3LYP\6-311+G(d,p)\C5H7(2)\WEIY\25-Apr-2020\0\\#p b
3lyp/6-311+g(d,p) geom=check scrf=(iefpcm,smd,solvent=dichloromethane)
\\Title Card Required\\0,2\C,0,-1.1643686701,-0.0753328436,-0.00007669
1\C,0,0.0222967662,-0.3077481647,0.0056919704\C,0,1.4636863148,-0.5768
17536,0.0165875041\C,0,2.3183874938,0.6544390063,-0.0240100589\H,0,1.7
21815078,-1.2350159531,-0.8299942084\H,0,1.7109710847,-1.1783998195,0.
9120739576\H,0,1.9036541945,1.630286331,0.1975654719\H,0,3.3902928363,
0.5588625887,-0.1641975235\C,0,-2.5991452202,0.2019577226,-0.009312162
5\H,0,-3.0759066488,-0.1207913743,0.9244847688\H,0,-2.7965865805,1.274
5398386,-0.1273061246\H,0,-3.1014221687,-0.318375336,-0.8341760439\\Ve
rsion=ES64L-G16RevA.03\State=2-A\HF=-194.6860918\S2=0.753957\S2-1=0.\S
2A=0.75001\RMSD=9.466e-09\Dipole=-0.1079042,-0.118712,0.0556939\Quadru
pole=4.3900889,-1.5254448,-2.8646441,-1.8588216,0.074398,0.2454757\PG=
C01 [X(C5H7)]\\@

C

1\1\GINC-OM103\SP\UB3LYP\6-311+G(d,p)\C5H9(2)\WEIY\26-Apr-2020\0\\#p b
3lyp/6-311+g(d,p) geom=check scrf=(iefpcm,smd,solvent=dichloromethane)
\\Title Card Required\\0,2\C,0,0.134629033,-0.359371202,0.1604717795\C
,0,0.1472921956,0.4900231341,1.3526759139\C,0,0.6474061571,-0.03953600
43,2.706916505\C,0,-0.8117926108,0.2354038238,2.5091073215\H,0,1.22787
18034,0.6368409424,3.3295398524\H,0,0.9767560044,-1.0745148427,2.74159
18378\H,0,-1.4856741009,-0.6124236676,2.4135376472\H,0,-1.2518843482,1
.0983997723,3.0020364349\C,0,0.7687006648,0.051625622,-1.127770087\H,0
,0.303297006,-0.4488019278,-1.9860368934\H,0,0.6915026756,1.1350541209
,-1.2880999351\H,0,1.8458086512,-0.1942476509,-1.1693462076\H,0,-0.171
6523224,-1.3975583165,0.2810378559\H,0,0.3822619095,1.5372062879,1.165
8380581\\Version=ES64L-G16RevA.03\State=2-A\HF=-195.9284328\S2=0.75478
4\S2-1=0.\S2A=0.750018\RMSD=8.669e-09\Dipole=0.1254539,0.0744258,0.093
37\Quadrupole=-1.0348619,1.2617762,-0.2269144,0.3560136,-0.3938121,0.5
948102\PG=C01 [X(C5H9)]\\@

TS’

1\1\GINC-OM103\SP\UB3LYP\6-311+G(d,p)\C5H9(2)\WEIY\26-Apr-2020\0\\#p b
3lyp/6-311+g(d,p) geom=check scrf=(iefpcm,smd,solvent=dichloromethane)
\\ts_radical_me\\0,2\C,0,-0.000426809,0.0004848037,0.0000011586\C,0,-0
.0049308643,-0.0036794386,1.387354265\C,0,1.6591060229,-0.0043214638,2
.3383145535\C,0,0.617637137,-1.0560228752,2.2477084607\H,0,1.656187103
4,0.7195812521,3.144747514\H,0,2.501300969,-0.0098816115,1.6581538589\
H,0,0.9076723422,-1.9648994576,1.7164986619\H,0,0.0836440952,-1.294715

6434,3.1695492469\C,0,-0.5527654532,1.1131434462,-0.8374385516\H,0,-1.
2500417803,0.7403977887,-1.6013312583\H,0,-1.0899425079,1.8470254245,-
0.225567247\H,0,0.2383492218,1.6547409312,-1.3803312464\H,0,0.50194708
1,-0.8146716256,-0.521435829\H,0,-0.5912871061,0.7527765463,1.90474127
99\\Version=ES64L-G16RevA.03\State=2-A\HF=-195.9163422\S2=0.775498\S2-
1=0.\S2A=0.750138\RMSD=6.678e-09\Dipole=-0.0337968,0.0171762,0.071487\
Quadrupole=-1.1893625,0.1495133,1.0398492,-0.7730911,-1.2218927,0.3701
099\PG=C01 [X(C5H9)]\\@

D

1\1\GINC-OM103\SP\UB3LYP\6-311+G(d,p)\C5H9(2)\WEIY\26-Apr-2020\0\\#p b
3lyp/6-311+g(d,p) geom=check scrf=(iefpcm,smd,solvent=dichloromethane)
\\Title Card Required\\0,2\C,0,0.0837105821,-0.2239641791,0.0916425154
\C,0,0.1606587526,-0.3268969716,1.420931399\C,0,1.9450267421,0.1526069
475,3.12094967\C,0,1.3532927157,-0.8530786394,2.1832805365\H,0,2.42793
54431,-0.1549121075,4.0432509136\H,0,2.0234944791,1.1948155289,2.82725
73204\H,0,2.1126184262,-1.1983528924,1.4571948546\H,0,1.0649410226,-1.
750196927,2.7525617669\C,0,-1.0974599097,0.3052492949,-0.6707735845\H,
0,-1.4961381507,-0.4488695491,-1.3631349271\H,0,-1.90727827,0.60824058
05,0.0016829034\H,0,-0.8206931471,1.1752841273,-1.2818766902\H,0,0.936
6145977,-0.5475985035,-0.5088887592\H,0,-0.6837363585,0.0010499748,2.0
299937023\\Version=ES64L-G16RevA.03\State=2-A\HF=-195.9330182\S2=0.753
821\S2-1=0.\S2A=0.75001\RMSD=8.986e-09\Dipole=-0.0021368,-0.105179,-0.
1681409\Quadrupole=-0.0636097,-0.735881,0.7994907,-0.8724525,0.3570413
,-0.7953268\PG=C01 [X(C5H9)]\\@

6. The Characterization Data of Substrates

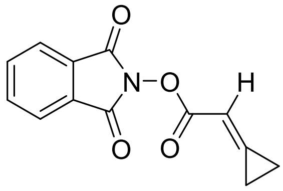

1,3-dioxoisoindolin-2-yl 2-cyclopropylideneacetate (1a): white solid, m.p. 185-186 °C, 531.6 mg, 63%, Eluent: PE/EA = 10/1.

^{1}H NMR (400 MHz, $CDCl_3$, TMS): δ 7.91 (dd, *J* = 5.6, 3.2 Hz, 2H), 7.80 (dd, *J* = 5.6, 3.2 Hz, 2H), 6.51 (p, *J* = 2.0 Hz, 1H), 1.64-1.58 (m, 2H), 1.45-1.39 (m, 2H). **^{13}C NMR** (100 MHz, $CDCl_3$, TMS): δ 3.4, 5.4, 106.0, 123.9, 129.0, 134.7, 153.2, 161.4, 162.2. **IR** (EtOH): ν 3092, 3000, 1798, 1732, 1718, 1489, 1356, 1283, 1185, 1131, 1029, 998, 875, 797, 694, 590 cm^{-1}. **HRMS** (ESI) m/z: $[M+Na]^+$ Calcd. for $C_{13}H_9NNaO_4$ 266.0424; found 266.0429.

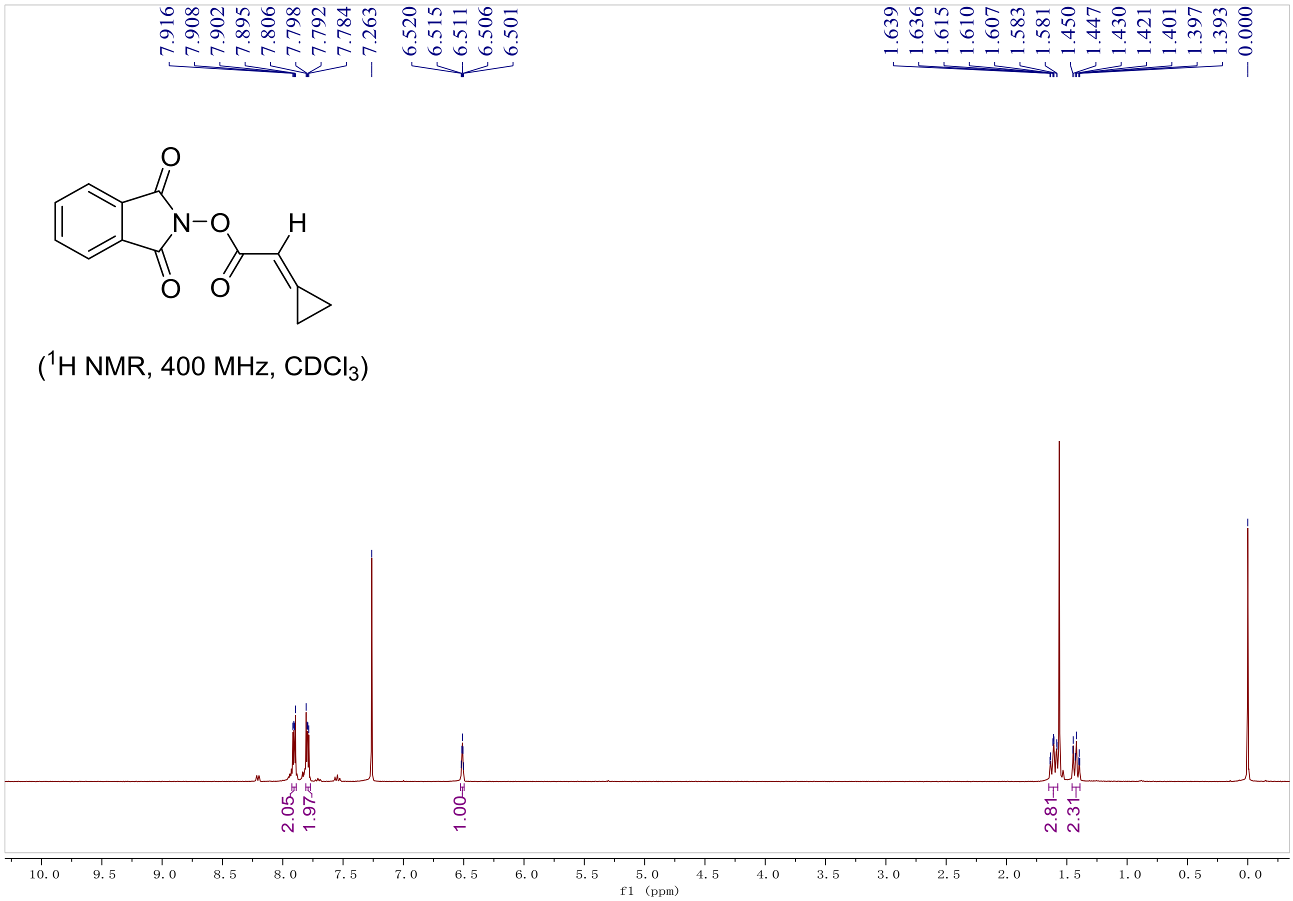

7.916 7.908 7.902 7.895 7.806 7.798 7.792 7.784 7.263 6.520 6.515 6.511 6.506 6.501 1.639 1.636 1.615 1.610 1.607 1.583 1.581 1.450 1.447 1.430 1.421 1.401 1.397 1.393 0.000
(1H NMR, 400 MHz, CDCl3)
2.05 1.97 1.00 2.81 2.31
f1 (ppm)

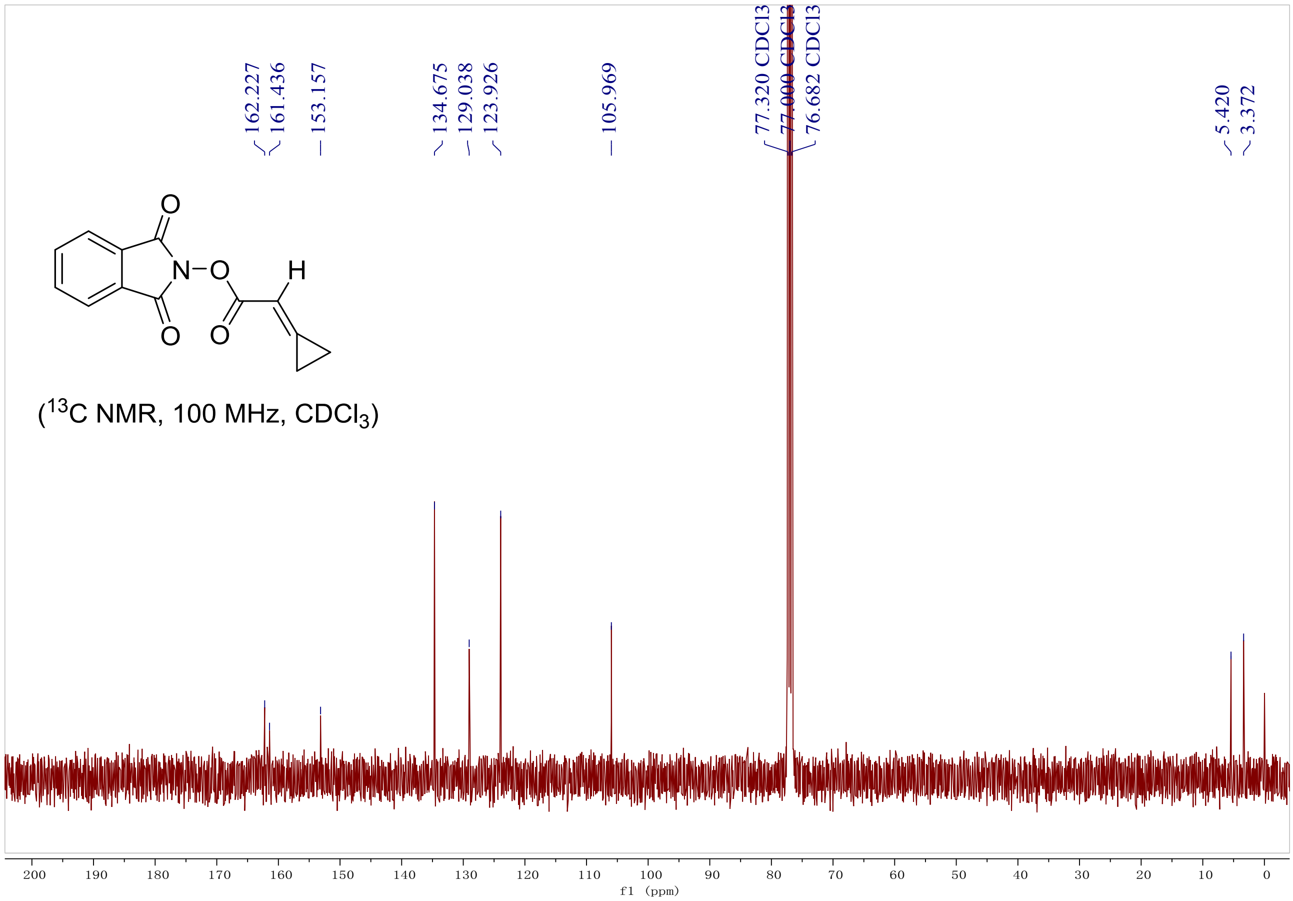

162.227 161.436 153.157 134.675 129.038 123.926 105.969 77.320 CDCl3 77.000 CDCl3 76.682 CDCl3 5.420 3.372
(13C NMR, 100 MHz, CDCl3)
f1 (ppm)

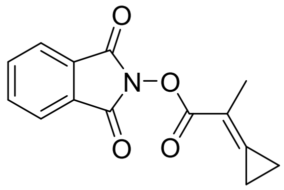

1,3-Dioxoisoindolin-2-yl 2-cyclopropylidenepropanoate (1b): white solid, m.p. 190-192 °C, 561.8 mg, 73%, Eluent: PE/EA = 10/1.

^{1}H NMR (400 MHz, $CDCl_3$, TMS): δ 7.90 (dd, *J* = 5.6, 3.2 Hz, 2H), 7.79 (dd, *J* = 5.6, 3.2 Hz, 2H), 2.15 (s, 3H), 1.70-1.59 (m, 2H), 1.38-1.30 (m, 2H). **^{13}C NMR** (100 MHz, $CDCl_3$, TMS): δ 3.4, 6.8, 16.7, 114.1, 123.9, 129.1, 134.6, 145.0, 162.4, 162.9. **IR** (EtOH): ν 2988, 2965, 1763, 1742, 1554, 1370, 1249, 1185, 1079, 1003, 879, 837 cm^{-1}. **HRMS** (ESI) m/z: $[M+Na]^+$ Calcd. for $C_{14}H_{11}NNaO_4$ 280.0580; found 280.0586.

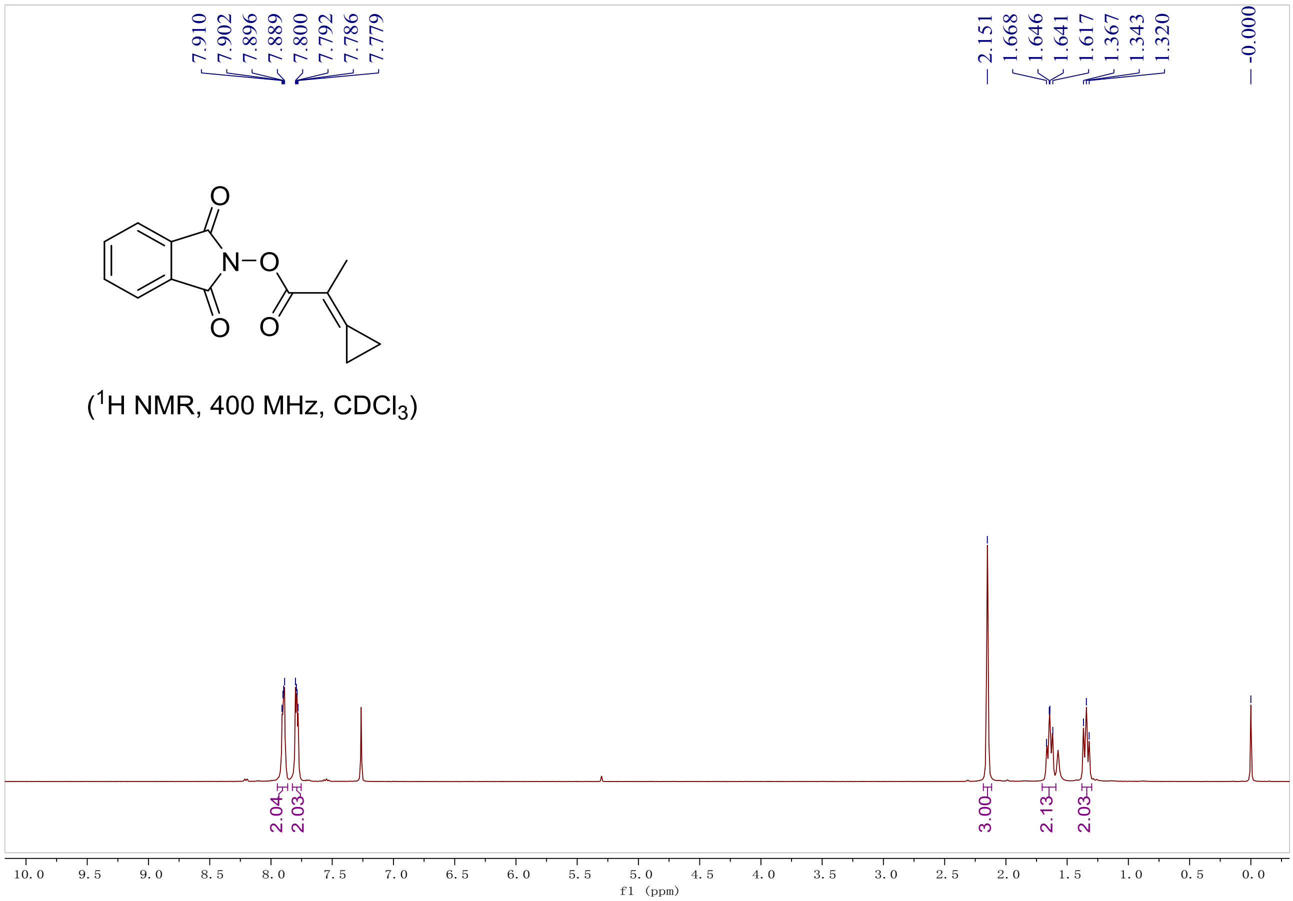
7.910
7.902
7.896
7.889
7.800
7.792
7.786
7.779
2.151
1.668
1.646
1.641
1.617
1.367
1.343
1.320
-0.000
(1H NMR, 400 MHz, CDCl3)
2.04
2.03
3.00
2.13
2.03
10.0 9.5 9.0 8.5 8.0 7.5 7.0 6.5 6.0 5.5 5.0 4.5 4.0 3.5 3.0 2.5 2.0 1.5 1.0 0.5 0.0
f1 (ppm)

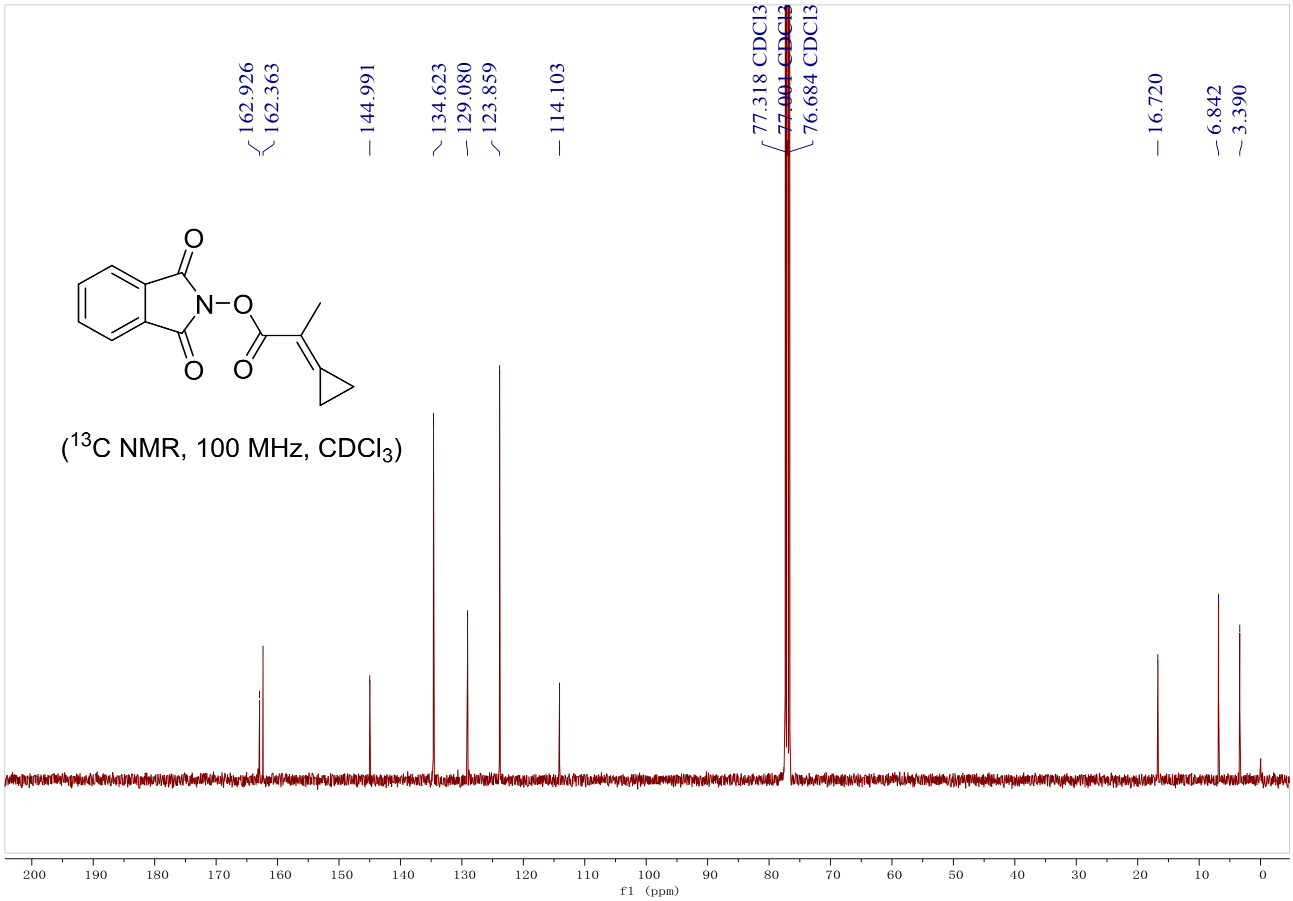
162.926
162.363
144.991
134.623
129.080
123.859
114.103
77.318 CDCl3
77.001 CDCl3
76.684 CDCl3
16.720
6.842
3.390
(13C NMR, 100 MHz, CDCl3)
200 190 180 170 160 150 140 130 120 110 100 90 80 70 60 50 40 30 20 10 0
f1 (ppm)

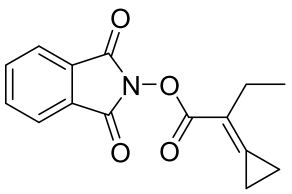

1,3-Dioxoisoindolin-2-yl 2-cyclopropylidenebutanoate (1c): white solid, m.p. 179-180 °C, 463.5 mg, 85%, Eluent:PE/EA = 10/1.

^{1}H NMR (400 MHz, $CDCl_3$, TMS): δ 7.90 (dd, *J* = 5.6, 3.2 Hz, 2H), 7.79 (dd, *J* = 5.6, 3.2 Hz, 2H), 2.57 (q, *J* = 7.6 Hz, 2H), 1.62-1.55 (m, 2H), 1.45-1.36 (m, 2H), 1.20 (t, *J* = 7.6 Hz, 3H). **^{13}C NMR** (100 MHz, $CDCl_3$, TMS): δ 3.7, 5.6, 12.8, 24.7, 119.7, 123.8, 129.1, 134.6, 143.9, 162.4, 162.7. **IR** (EtOH): ν 2959, 2846, 1801, 1730, 1511, 1365, 1243, 1138, 1026, 998, 879, 700 cm^{-1}. **HRMS** (ESI) m/z: $[M+Na]^+$ Calcd. for $C_{15}H_{13}NNaO_4$ 294.0737; found 294.0744.

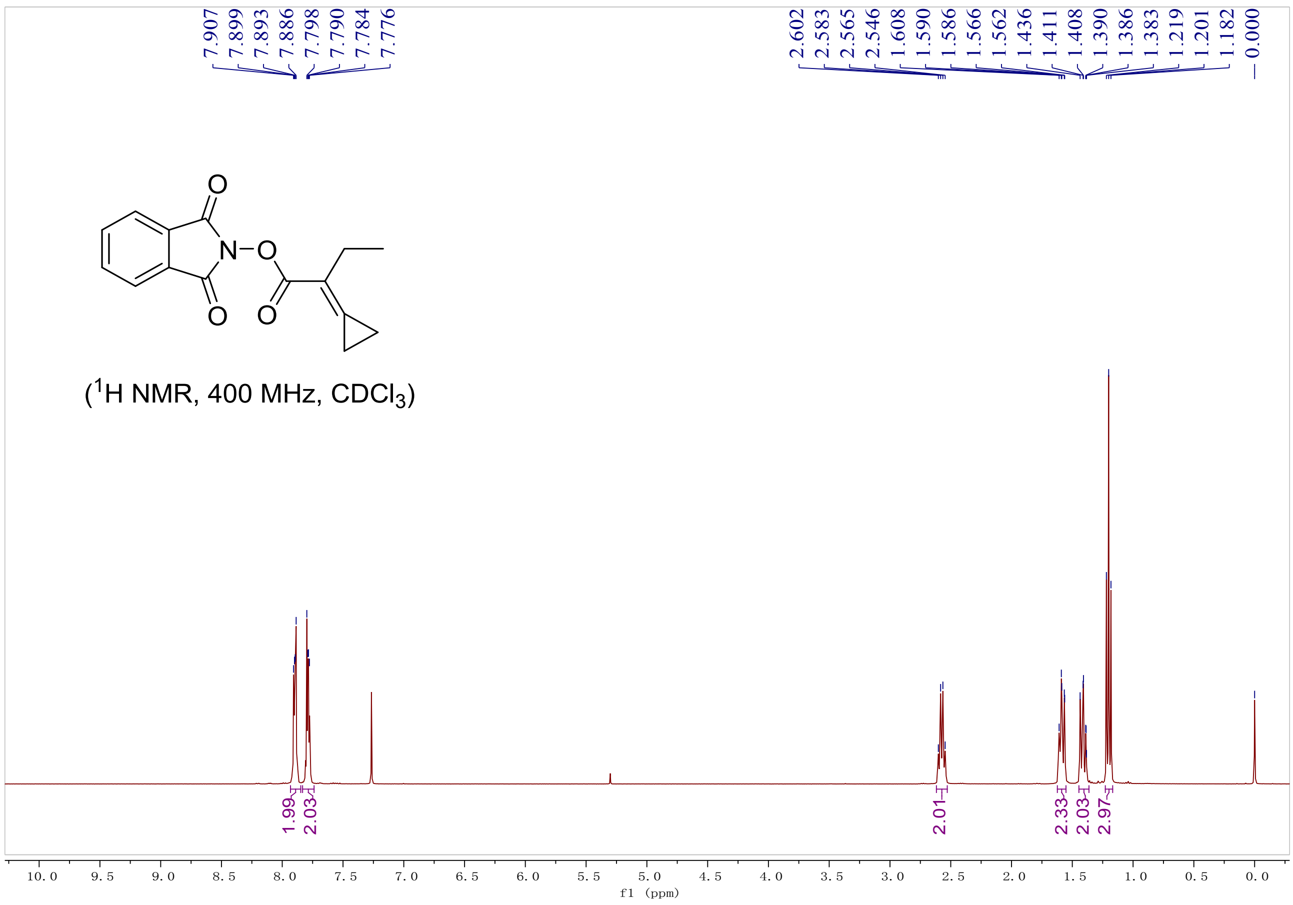
7.907
7.899
7.893
7.886
7.798
7.790
7.784
7.776
2.602
2.583
2.565
2.546
1.608
1.590
1.586
1.566
1.562
1.436
1.411
1.408
1.390
1.386
1.383
1.219
1.201
1.182
0.000
(1H NMR, 400 MHz, CDCl3)
1.99
2.03
2.01
2.33
2.03
2.97
f1 (ppm)

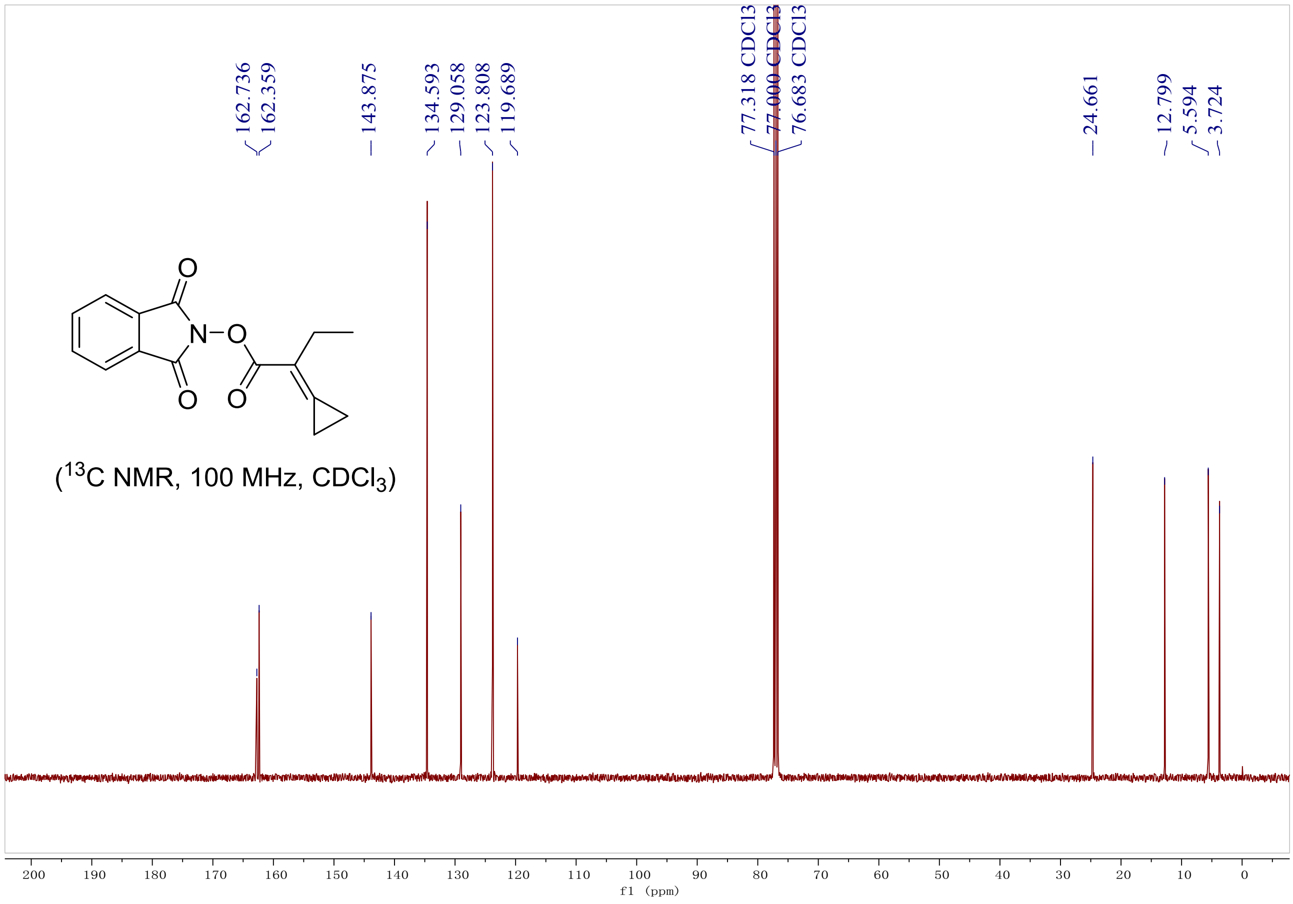
162.736
162.359
143.875
134.593
129.058
123.808
119.689
77.318 CDCl3
77.000 CDCl3
76.683 CDCl3
24.661
12.799
5.594
3.724
(13C NMR, 100 MHz, CDCl3)
f1 (ppm)

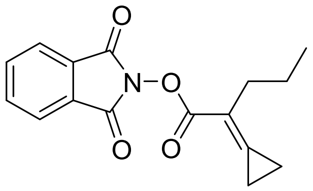

1,3-Dioxoisoindolin-2-yl 2-cyclopropylidenepentanoate (1d): white solid, m.p. 184-186 °C, 389.5 mg, 68%, Eluent: PE/EA = 10/1.

^{1}H NMR (400 MHz, $CDCl_3$, TMS): δ 7.89 (dd, *J* = 5.6, 3.2 Hz, 2H), 7.79 (dd, *J* = 5.6, 3.2 Hz, 2H), 2.51 (t, *J* = 7.6 Hz, 2H), 1.69-1.57 (m, 4H), 1.42-1.35 (m, 2H), 0.94 (t, *J* = 7.6 Hz, 3H). **^{13}C NMR** (100 MHz, $CDCl_3$, TMS): δ 3.8, 6.1, 13.8, 21.6, 33.2, 118.4, 123.8, 129.1, 134.6, 144.7, 162.4, 162.8. **IR** (EtOH): ν 3081, 2988, 2898, 1802, 1763, 1744, 1467, 1371, 1222, 1138, 1090, 983, 876, 787, 706 cm^{-1}. **HRMS** (ESI) m/z: $[M+Na]^+$ Calcd. for $C_{16}H_{15}NNaO_4$ 308.0893; found 308.0900.

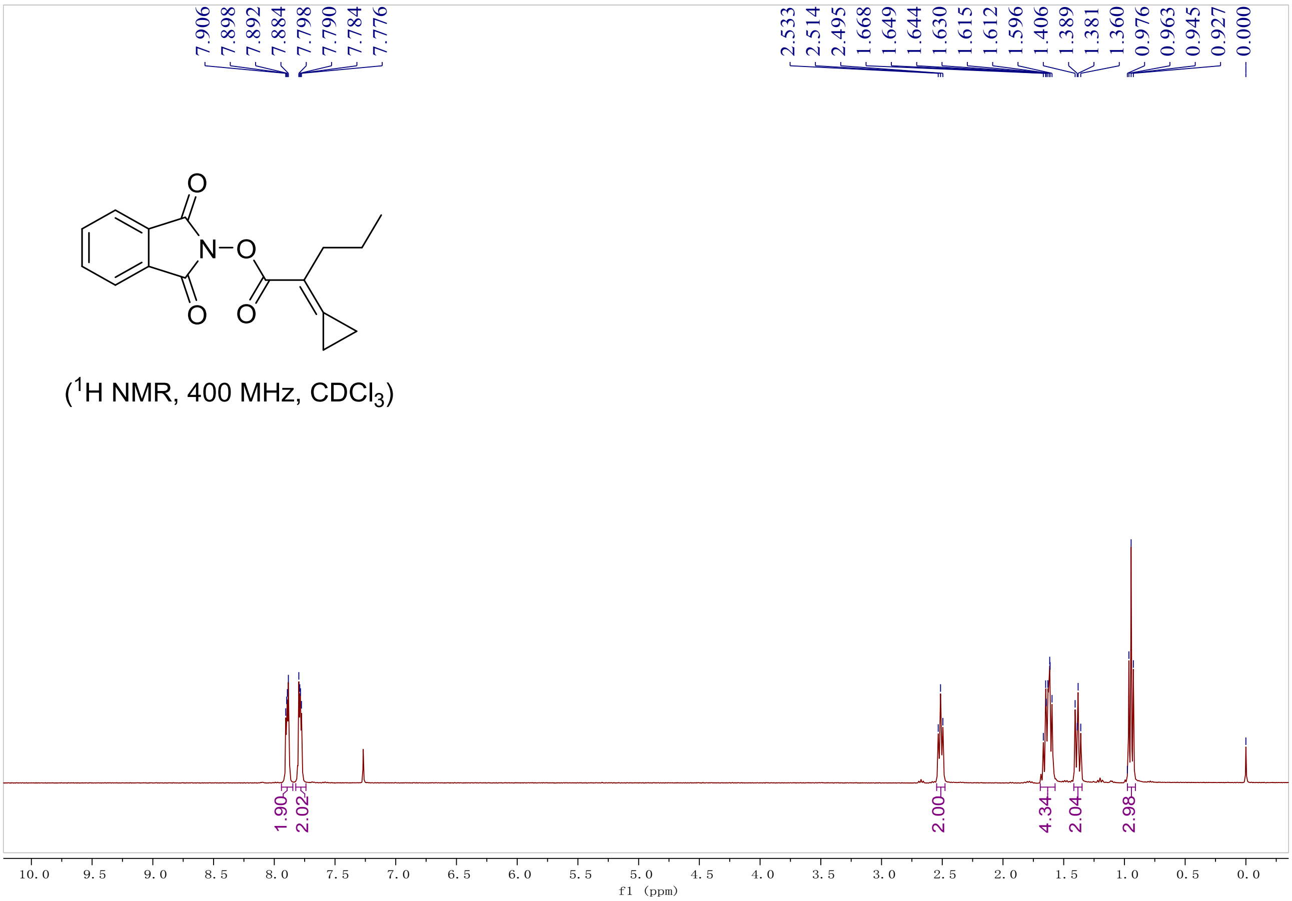
7.906
7.898
7.892
7.884
7.798
7.790
7.784
7.776
2.533
2.514
2.495
1.668
1.649
1.644
1.630
1.615
1.612
1.596
1.406
1.389
1.381
1.360
0.976
0.963
0.945
0.927
0.000
O
N–O
O
O
(¹H NMR, 400 MHz, CDCl3)
1.90
2.02
2.00
4.34
2.04
2.98
10.0 9.5 9.0 8.5 8.0 7.5 7.0 6.5 6.0 5.5 5.0 4.5 4.0 3.5 3.0 2.5 2.0 1.5 1.0 0.5 0.0
f1 (ppm)

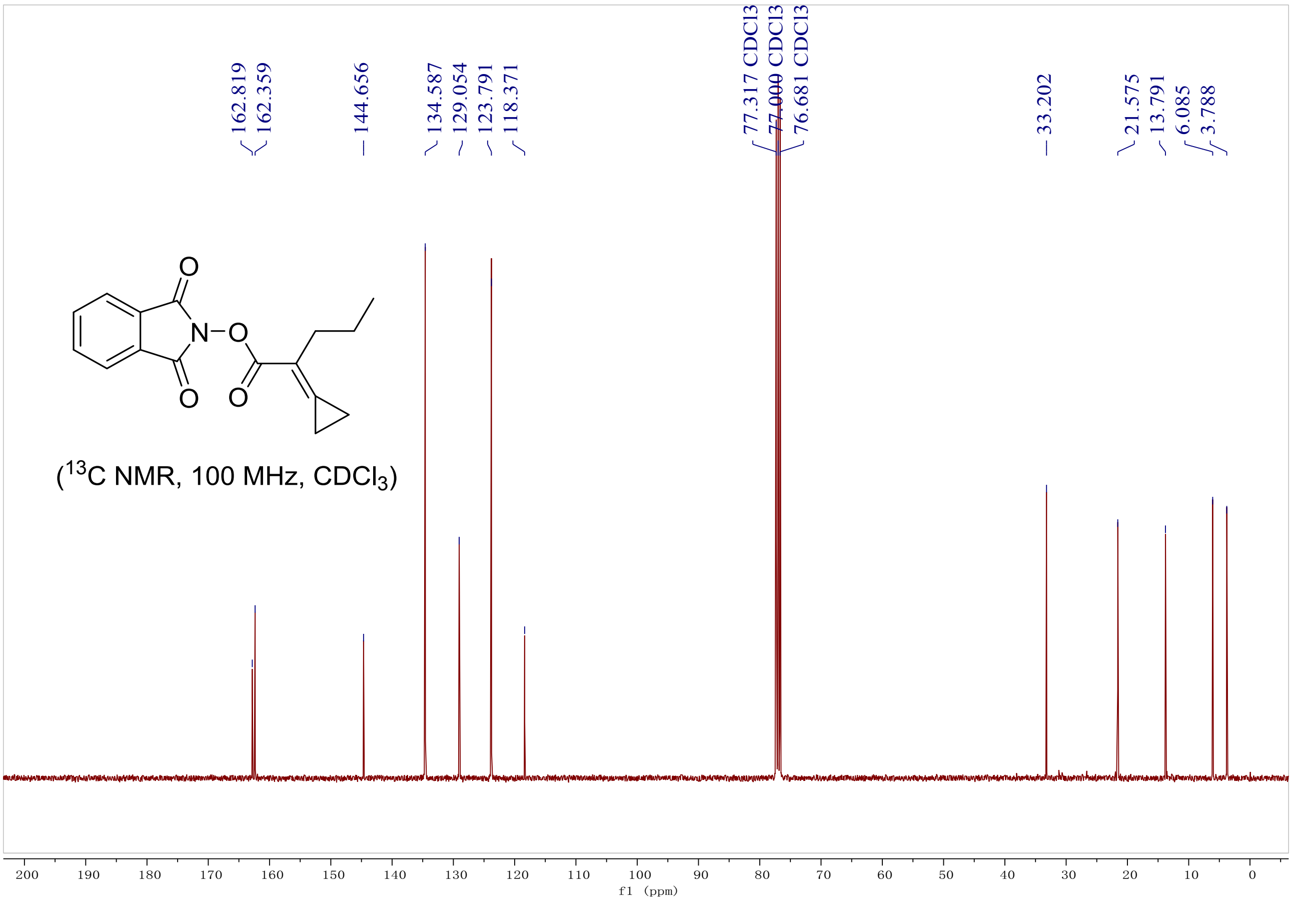
162.819
162.359
144.656
134.587
129.054
123.791
118.371
77.317 CDCl3
77.000 CDCl3
76.681 CDCl3
33.202
21.575
13.791
6.085
3.788
O
N–O
O
O
(¹³C NMR, 100 MHz, CDCl3)
200 190 180 170 160 150 140 130 120 110 100 90 80 70 60 50 40 30 20 10 0
f1 (ppm)

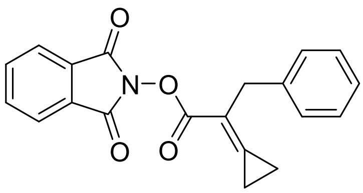

1,3-Dioxoisoindolin-2-yl 2-cyclopropylidene-3-phenylpropanoate (1e): white solid, m.p. 182-183 °C, 539.5 mg, 81%, Eluent: PE/EA = 10/1.

^{1}H NMR (400 MHz, $CDCl_3$, TMS): δ 7.88 (dd, *J* = 5.6, 3.2 Hz, 2H), 7.78 (dd, *J* = 5.6, 3.2 Hz, 2H), 7.32-7.17 (m, 5H), 3.86 (s, 2H), 1.67-1.57 (m, 2H), 1.28-1.18 (m, 2H). **^{13}C NMR** (150 MHz, $CDCl_3$, TMS): δ 4.1, 6.2, 37.1, 117.8, 123.8, 126.4, 128.4, 129.0, 129.1, 134.6, 138.7, 146.6, 162.3, 162.7. **IR** (EtOH): ν 2950, 2923, 2837, 1801, 1745, 1373, 1273, 1165, 1132, 1079, 1024, 996, 877, 843, 789, 694, 683 cm^{-1}. **HRMS** (ESI) m/z: $[M+Na]^+$ Calcd. for $C_{20}H_{15}NNaO_4$ 356.0893; found 356.0902.

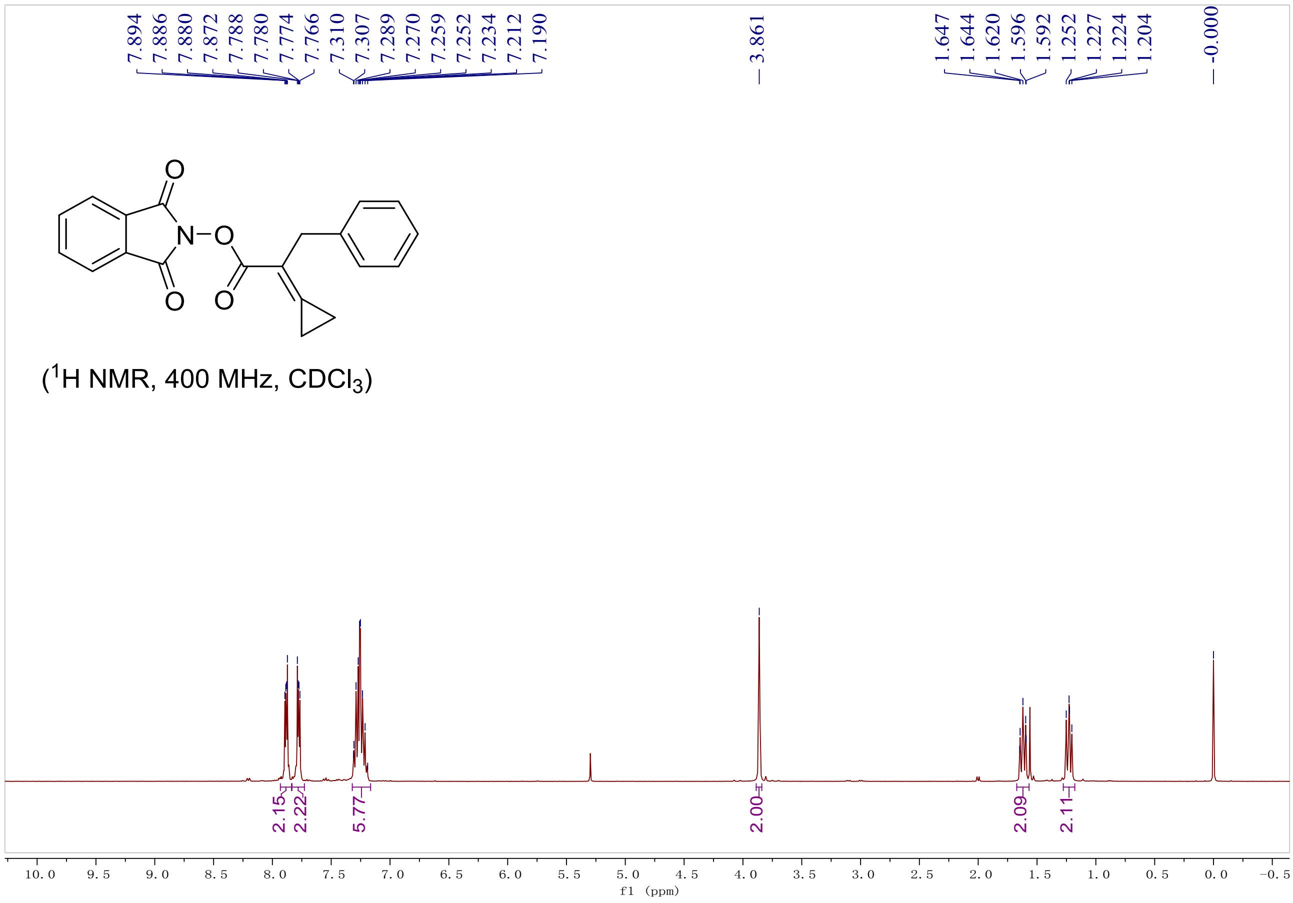
7.894
7.886
7.880
7.872
7.788
7.780
7.774
7.766
7.310
7.307
7.289
7.270
7.259
7.252
7.234
7.212
7.190
3.861
1.647
1.644
1.620
1.596
1.592
1.252
1.227
1.224
1.204
-0.000
O
N
O
O
O
(1H NMR, 400 MHz, CDCl3)
2.15
2.22
5.77
2.00
2.09
2.11
f1 (ppm)

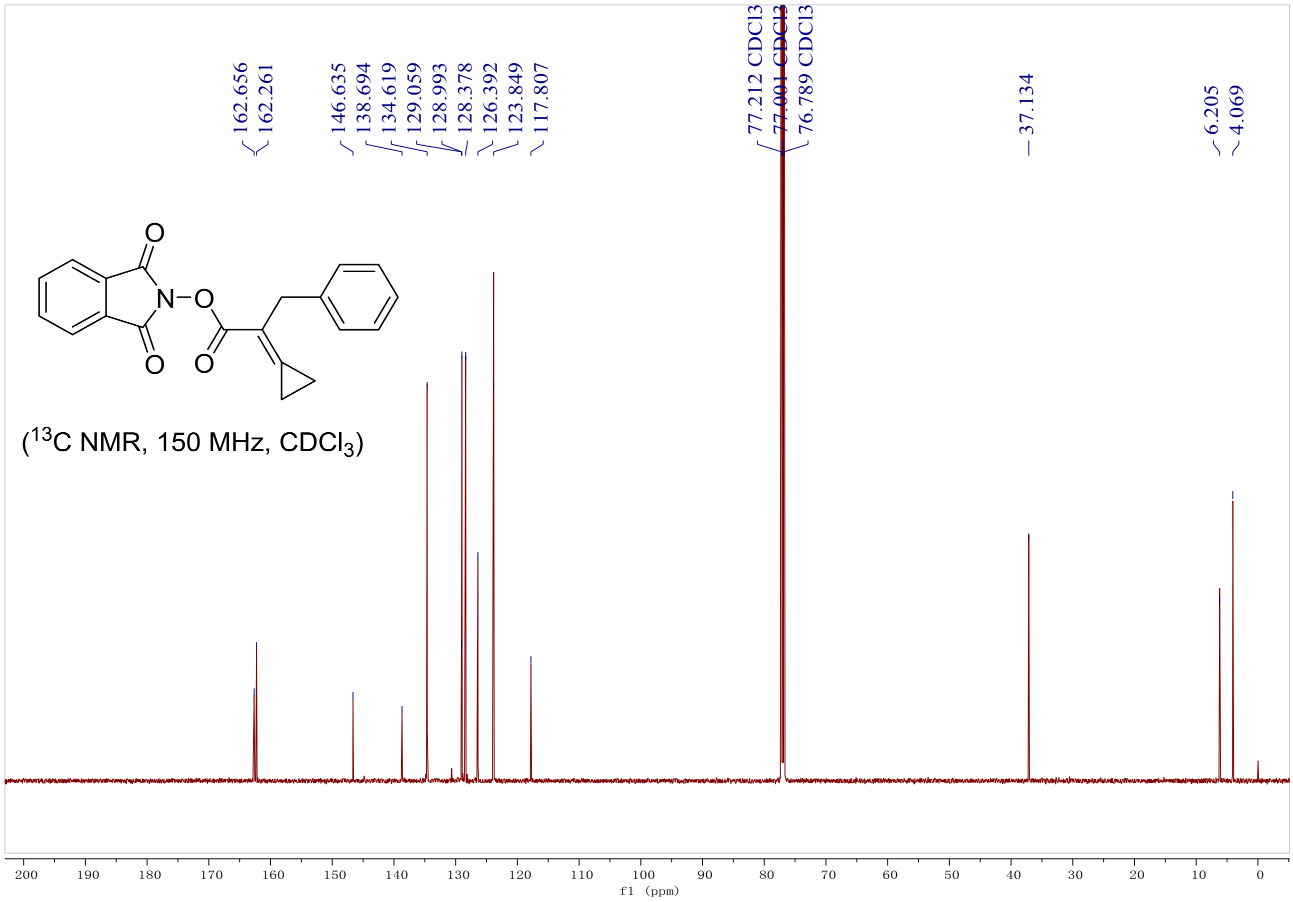
162.656
162.261
146.635
138.694
134.619
129.059
128.993
128.378
126.392
123.849
117.807
77.212 CDCl3
77.001 CDCl3
76.789 CDCl3
37.134
6.205
4.069
O
N
O
O
O
(13C NMR, 150 MHz, CDCl3)
f1 (ppm)

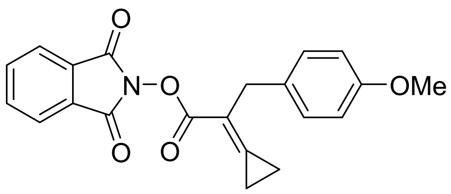

1,3-Dioxoisoindolin-2-yl 2-cyclopropylidene-3-(4-methoxyphenyl)propanoate (1f): white solid, m.p. 182-184 °C, 321.1 mg, 45%, Eluent: PE/EA = 10/1.

^{1}H NMR (400 MHz, $CDCl_3$, TMS): δ 7.89 (dd, J = 5.6, 3.2 Hz, 2H), 7.78 (dd, J = 5.6, 3.2 Hz, 2H), 7.16 (d, J = 8.6 Hz, 2H), 6.83 (d, J = 8.6 Hz, 2H), 3.82-3.76 (m, 5H), 1.63-1.59 (m, 2H), 1.26-1.19 (m, 2H). **^{13}C NMR** (150 MHz, $CDCl_3$, TMS): δ 4.1, 6.1, 36.3, 55.2, 113.8, 118.1, 123.9, 129.1, 130.0, 130.8, 134.6, 146.3, 158.2, 162.3, 162.7. **IR** (EtOH): ν 2922, 2905, 1796, 1741, 1611, 1511, 1466, 1365, 1278, 1243, 1184, 1135, 1081, 1026, 999, 832, 700 cm^{-1}. **HRMS** (ESI) m/z: $[M+Na]^+$ Calcd. for $C_{21}H_{17}NNaO_5$ 386.0999; found 386.1000.

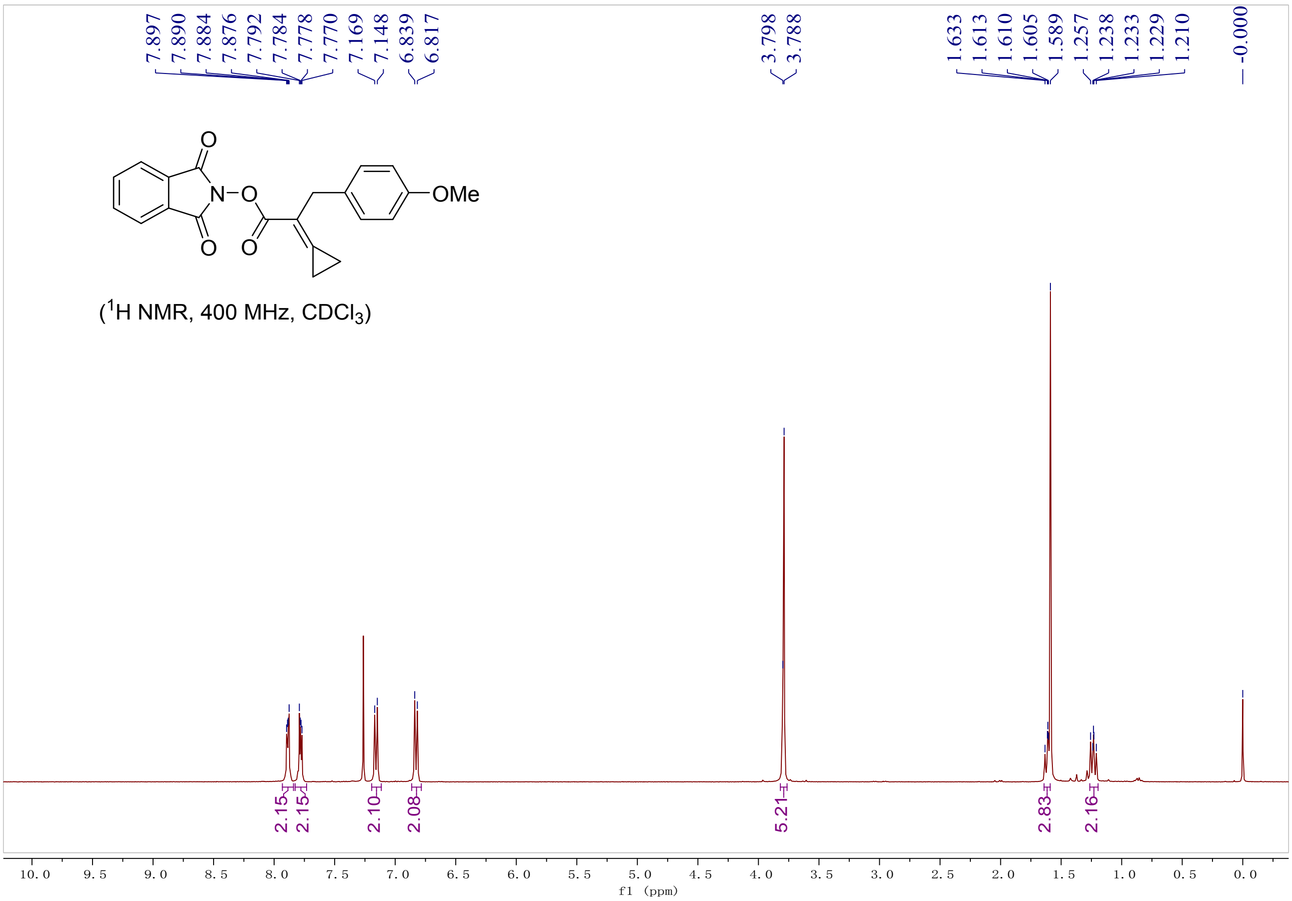
7.897
7.890
7.884
7.876
7.792
7.784
7.778
7.770
7.169
7.148
6.839
6.817
3.798
3.788
1.633
1.613
1.610
1.605
1.589
1.257
1.238
1.233
1.229
1.210
-0.000
OMe
(^{1}H NMR, 400 MHz, CDCl$_3$)
2.15
2.15
2.10
2.08
5.21
2.83
2.16
f1 (ppm)

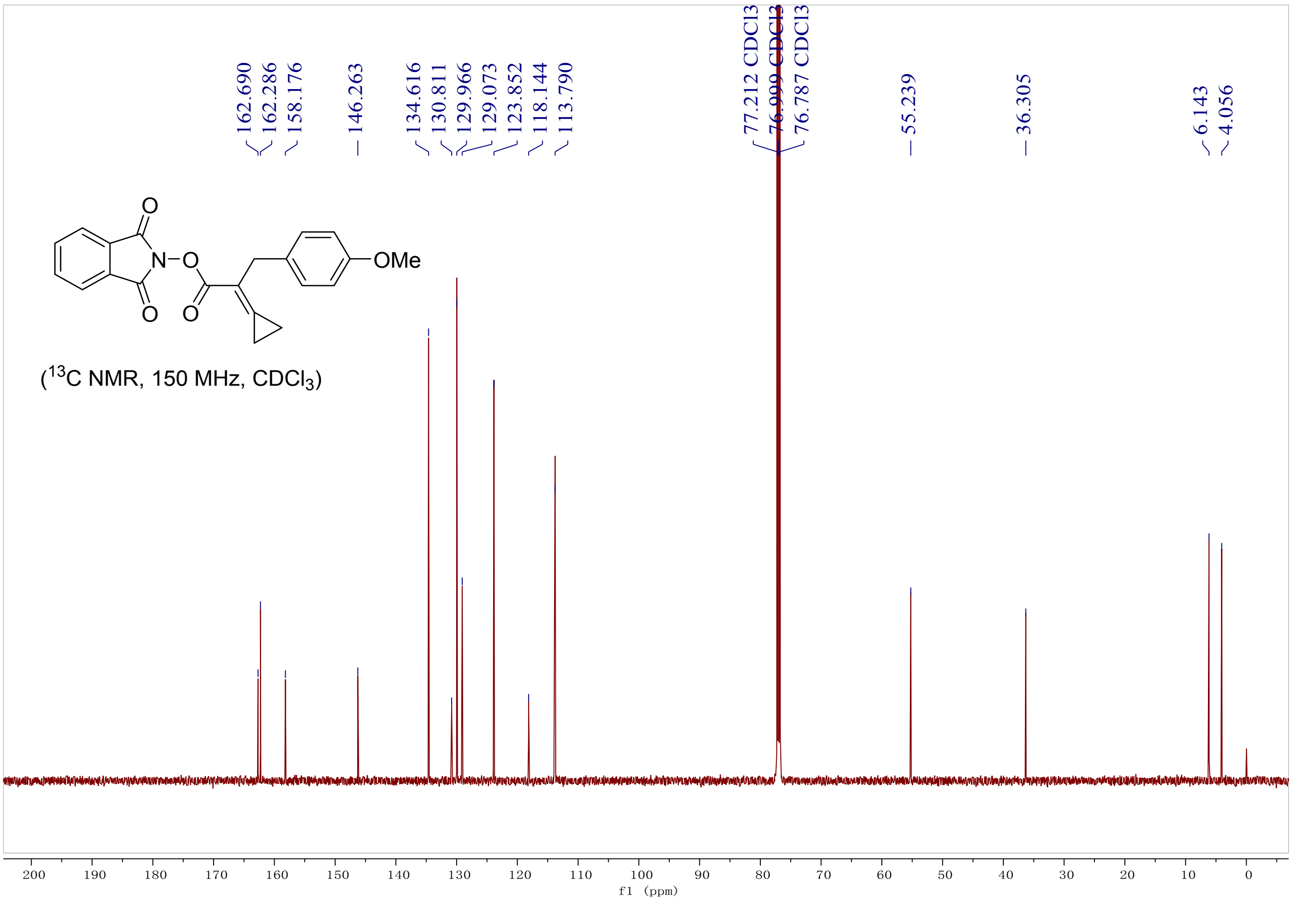
162.690
162.286
158.176
146.263
134.616
130.811
129.966
129.073
123.852
118.144
113.790
77.212 CDCl3
76.999 CDCl3
76.787 CDCl3
55.239
36.305
6.143
4.056
OMe
(^{13}C NMR, 150 MHz, CDCl$_3$)
f1 (ppm)

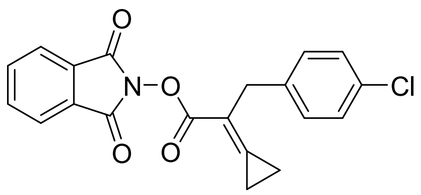

1,3-Dioxoisoindolin-2-yl 3-(4-chlorophenyl)-2-cyclopropylidenepropanoate (1g): white solid, m.p. 183-184 °C, 389.4 mg, 53%, Eluent: PE/EA = 10/1.

^{1}H NMR (400 MHz, $CDCl_3$, TMS): δ 7.89 (dd, J = 5.6, 3.2 Hz, 2H), 7.78 (dd, J = 5.6, 3.2 Hz, 2H), 7.27-7.24 (m, 2H), 7.17 (d, J = 8.4 Hz, 2H), 3.82 (s, 2H), 1.69-1.59 (m, 2H), 1.30-1.20 (m, 2H). **^{13}C NMR** (100 MHz, $CDCl_3$, TMS): δ 4.1, 6.3, 36.5, 117.4, 123.9, 128.5, 129.0, 130.3, 132.2, 134.7, 137.1, 146.9, 162.2, 162.5. **IR** (EtOH): ν 3058, 2966, 1796, 1735, 1607, 1489, 1468, 1356, 1283, 1185, 1131, 1084, 1029, 998, 964, 921, 857, 727, 687 cm^{-1}. **HRMS** (ESI) m/z: $[M+Na]^+$ Calcd. for $C_{20}H_{14}NNaClO_4$ 390.0504; found 390.0502.

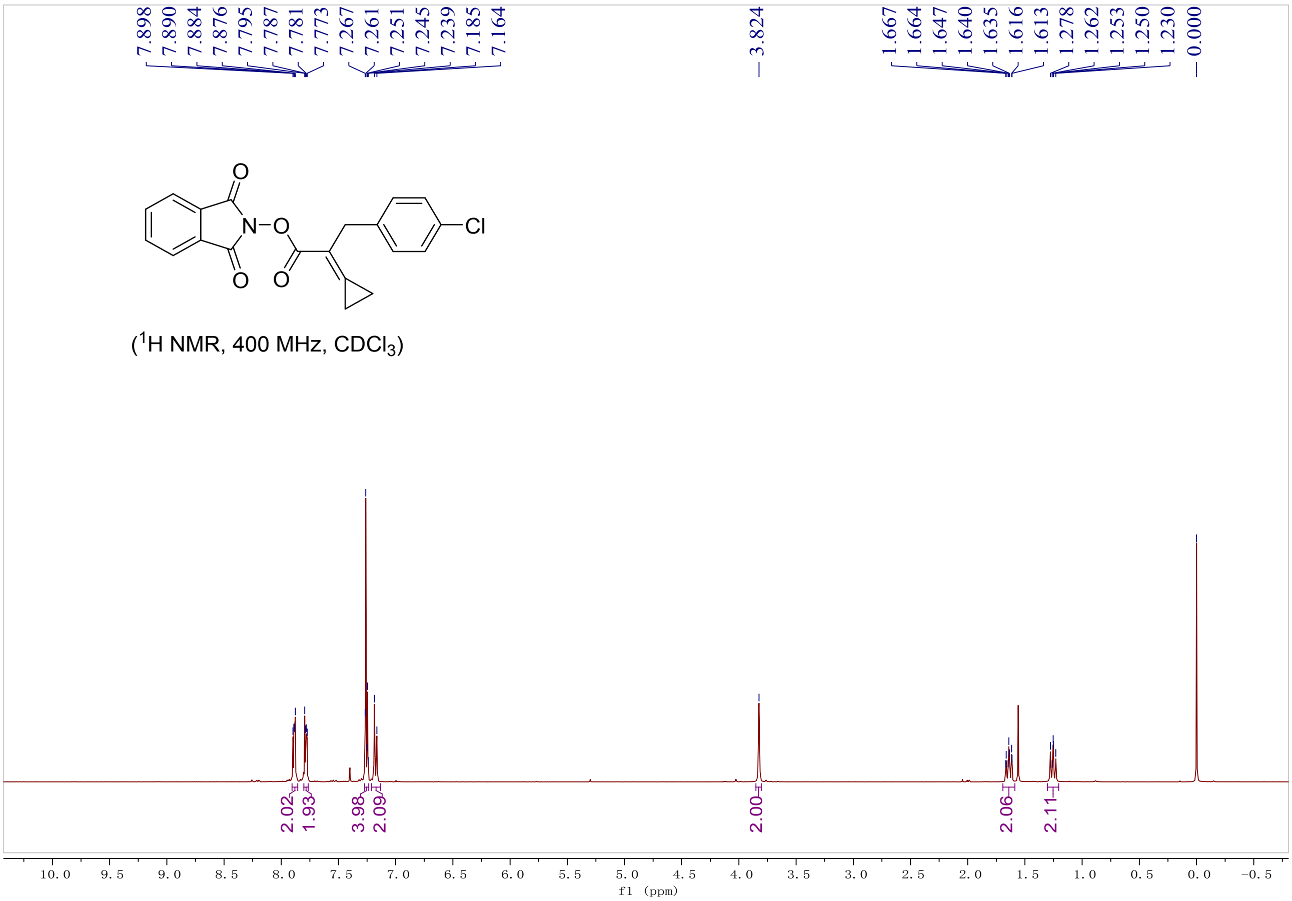
7.898
7.890
7.884
7.876
7.795
7.787
7.781
7.773
7.267
7.261
7.251
7.245
7.239
7.185
7.164
3.824
1.667
1.664
1.647
1.640
1.635
1.616
1.613
1.278
1.262
1.253
1.250
1.230
0.000
Cl
(1H NMR, 400 MHz, CDCl3)
2.02
1.93
3.98
2.09
2.00
2.06
2.11
f1 (ppm)

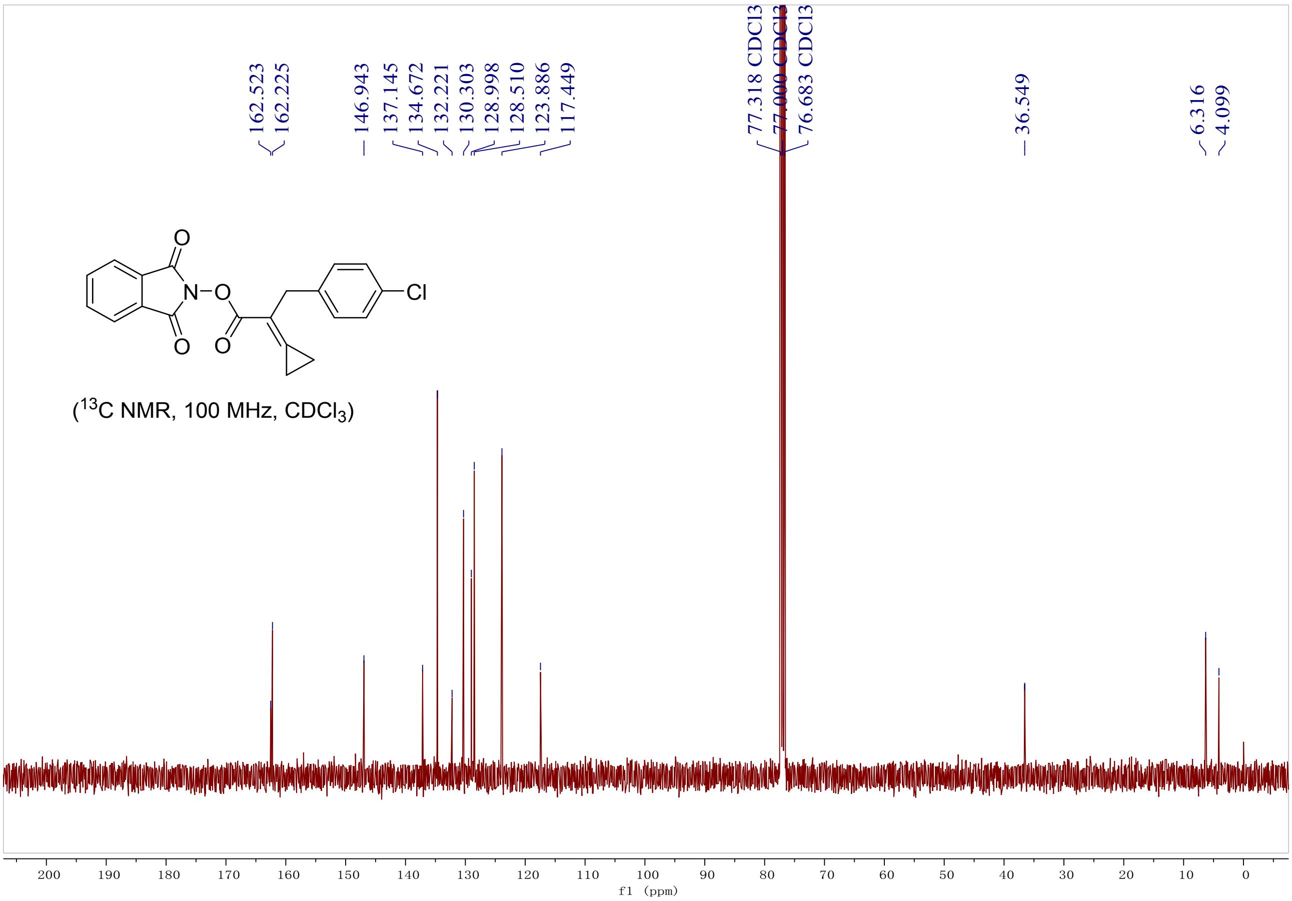
162.523
162.225
146.943
137.145
134.672
132.221
130.303
128.998
128.510
123.886
117.449
77.318 CDCl3
77.000 CDCl3
76.683 CDCl3
36.549
6.316
4.099
Cl
(13C NMR, 100 MHz, CDCl3)
f1 (ppm)

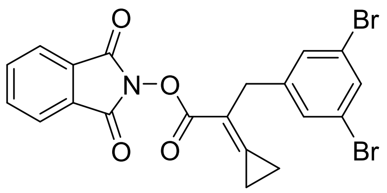

1,3-dioxoisoindolin-2-yl 2-cyclopropylidene-3-(3,5-dibromophenyl)propanoate (1h): white solid, m.p. 193-194 °C, 618.8 mg, 63%, Eluent: PE/EA = 10/1.

^{1}H NMR (400 MHz, $CDCl_3$, TMS): δ 7.90 (dd, *J* = 5.6, 3.2 Hz, 2H), 7.79 (dd, *J* = 5.6, 3.2 Hz, 2H), 7.53 (t, *J* = 1.8 Hz, 1H), 7.33 (d, *J* = 1.8 Hz, 2H), 3.80 (s, 2H), 1.72-1.66 (m, 2H), 1.36-1.26 (m, 2H). **^{13}C NMR** (150 MHz, $CDCl_3$, TMS): δ 4.2, 6.5, 36.6, 116.7, 123.1, 123.9, 129.0, 130.3, 132.2, 134.7, 142.5, 147.8, 162.1, 162.3. **IR** (EtOH): ν 2988, 2901, 1756, 1742, 1550, 1374, 1250, 1185, 1078, 1057, 1003, 879, 740, 693 cm^{-1}. **HRMS** (ESI) m/z: $[M+Na]^+$ Calcd. for $C_{20}H_{13}NBr_2O_4Na$ 511.9104; found 511.9096.

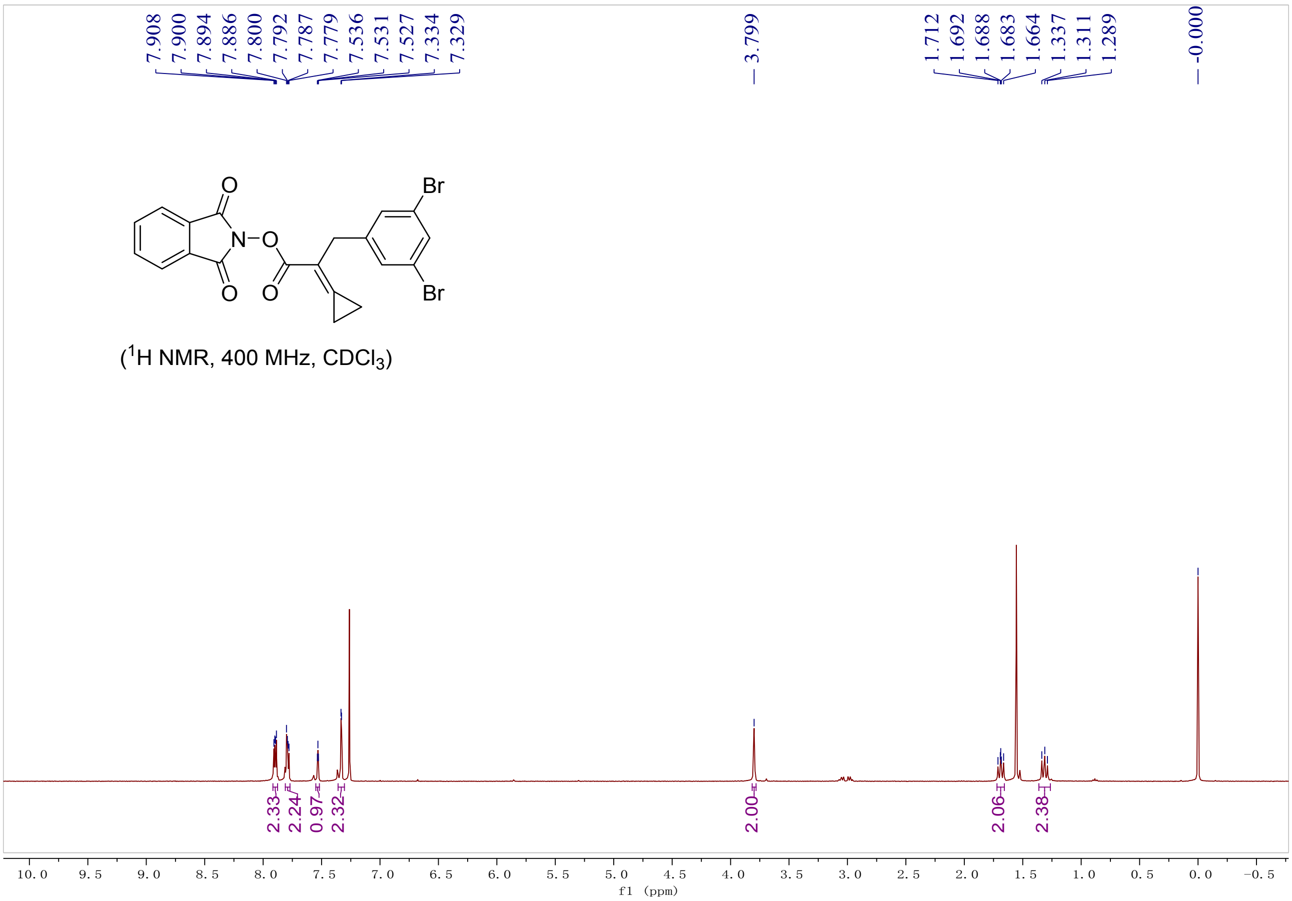
7.908
7.900
7.894
7.886
7.800
7.792
7.787
7.779
7.536
7.531
7.527
7.334
7.329
3.799
1.712
1.692
1.688
1.683
1.664
1.337
1.311
1.289
-0.000
Br
O
N–O
O
O
Br
(1H NMR, 400 MHz, CDCl3)
2.33
2.24
0.97
2.32
2.00
2.06
2.38
f1 (ppm)

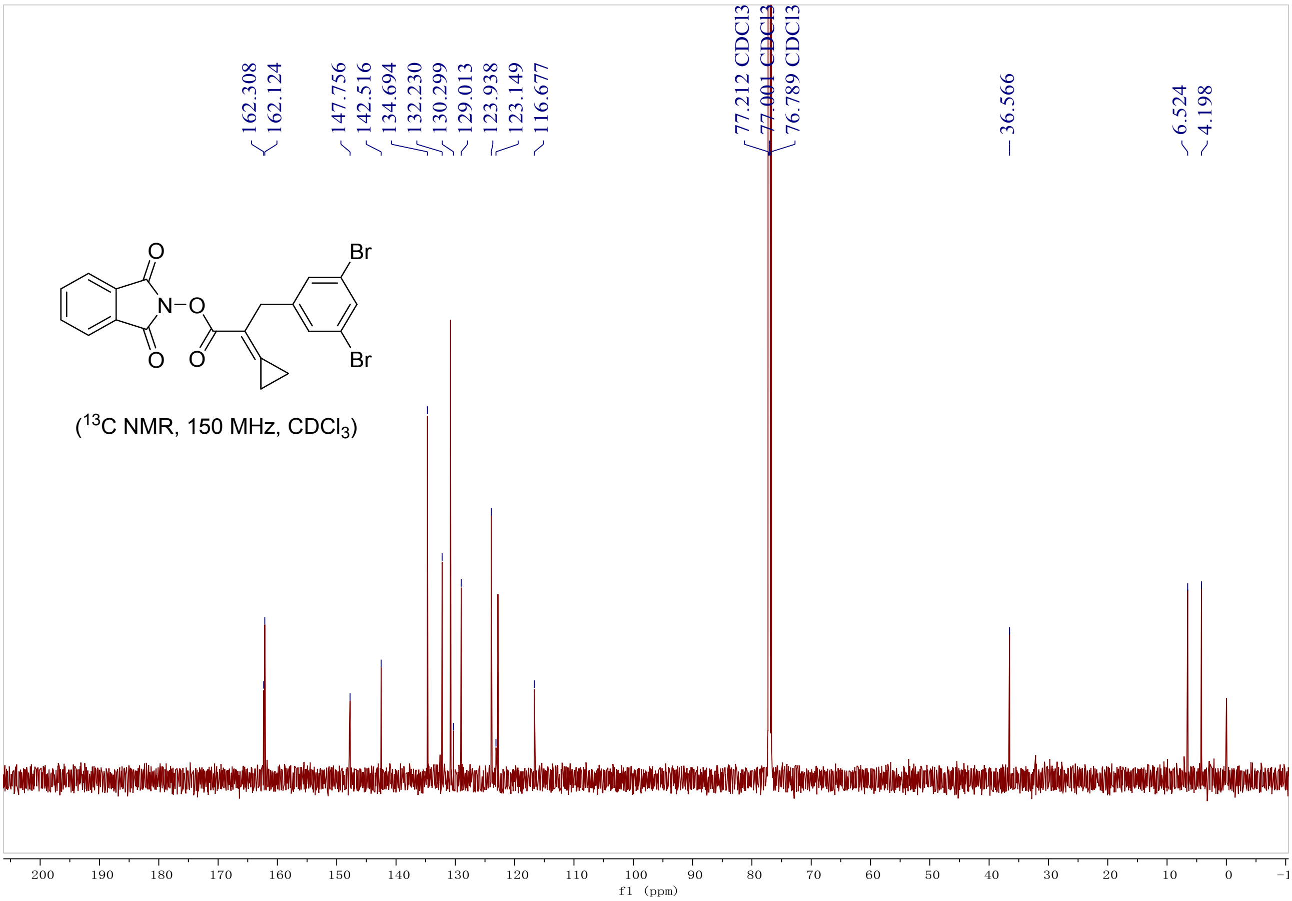
162.308
162.124
147.756
142.516
134.694
132.230
130.299
129.013
123.938
123.149
116.677
77.212 CDCl3
77.001 CDCl3
76.789 CDCl3
36.566
6.524
4.198
Br
O
N–O
O
O
Br
(13C NMR, 150 MHz, CDCl3)
f1 (ppm)

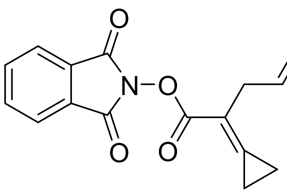

1,3-Dioxoisoindolin-2-yl 2-cyclopropylidenepent-4-enoate (1i): white solid, m.p. 185-186 °C, 461.8 mg, 82%, Eluent: PE/EA = 10/1.

^{1}H NMR (400 MHz, $CDCl_3$, TMS): δ 7.89 (dd, *J* = 5.6, 3.2 Hz, 2H), 7.79 (dd, *J* = 5.6, 3.2 Hz, 2H), 5.96 (ddt, *J* = 16.8, 10.2, 6.6 Hz, 1H), 5.19-5.03 (m, 2H), 3.32-3.27 (m, 2H), 1.69-1.58 (m, 2H), 1.46-1.36 (m, 2H). **^{13}C NMR** (150 MHz, $CDCl_3$, TMS): δ 4.0, 6.1, 35.5, 116.4, 116.6, 123.8, 129.1, 134.5, 134.6, 145.9, 162.3, 162.5. **IR** (EtOH): ν 3099, 3069, 3045, 3024, 1804, 1760, 1751, 1602, 1467, 1371, 1222, 1185, 1084, 1016, 986, 968, 879, 748, 691 cm^{-1}. **HRMS** (ESI) m/z: $[M+Na]^+$ Calcd. for $C_{16}H_{13}NNaO_4$ 306.0737; found 306.0743.

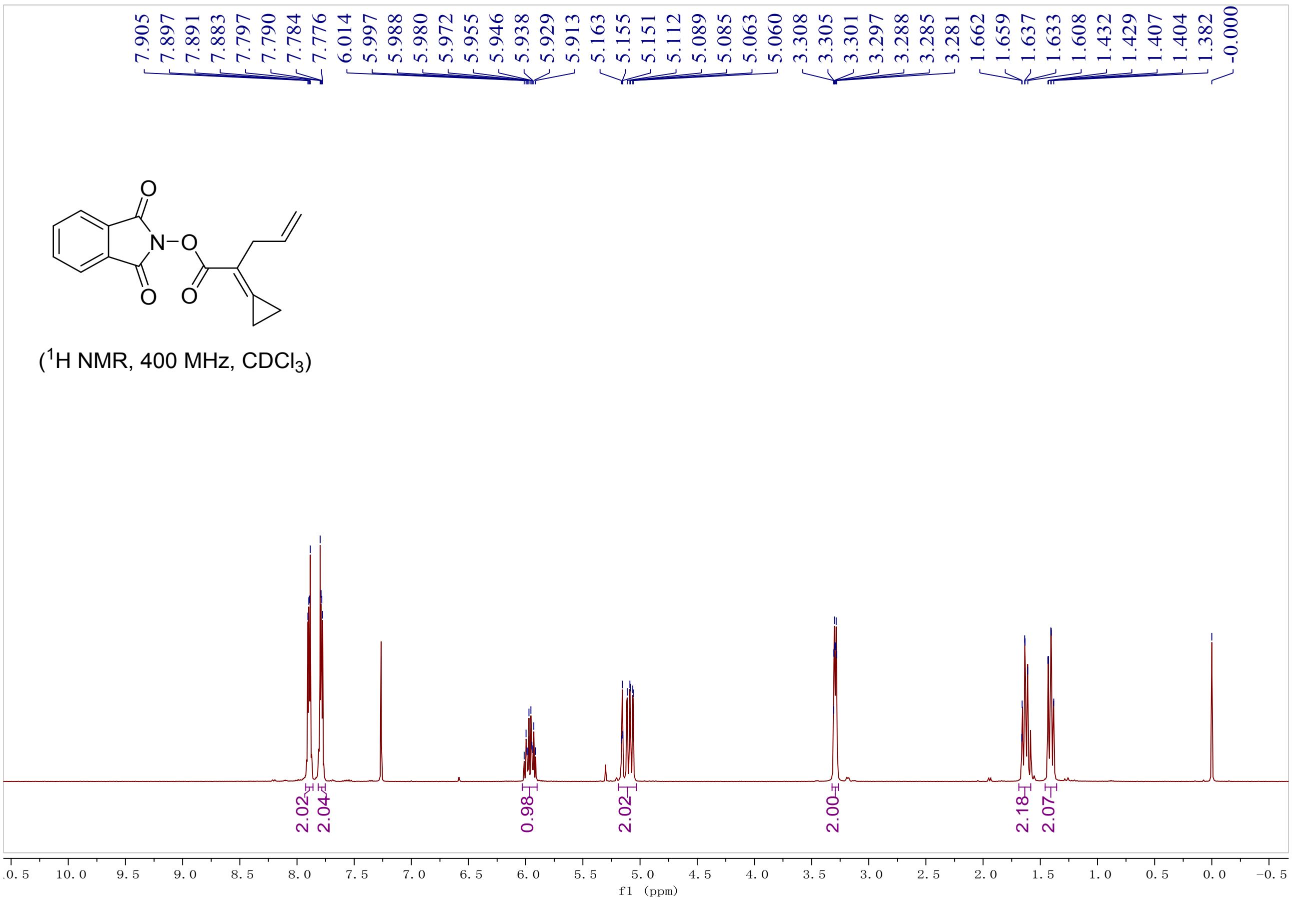
7.905 7.897 7.891 7.883 7.797 7.790 7.784 7.776 6.014 5.997 5.988 5.980 5.972 5.955 5.946 5.938 5.929 5.913 5.163 5.155 5.151 5.112 5.089 5.085 5.063 5.060 3.308 3.305 3.301 3.297 3.288 3.285 3.281 1.662 1.659 1.637 1.633 1.608 1.432 1.429 1.407 1.404 1.382 -0.000
(1H NMR, 400 MHz, CDCl3)
2.02 2.04 0.98 2.02 2.00 2.18 2.07
f1 (ppm)

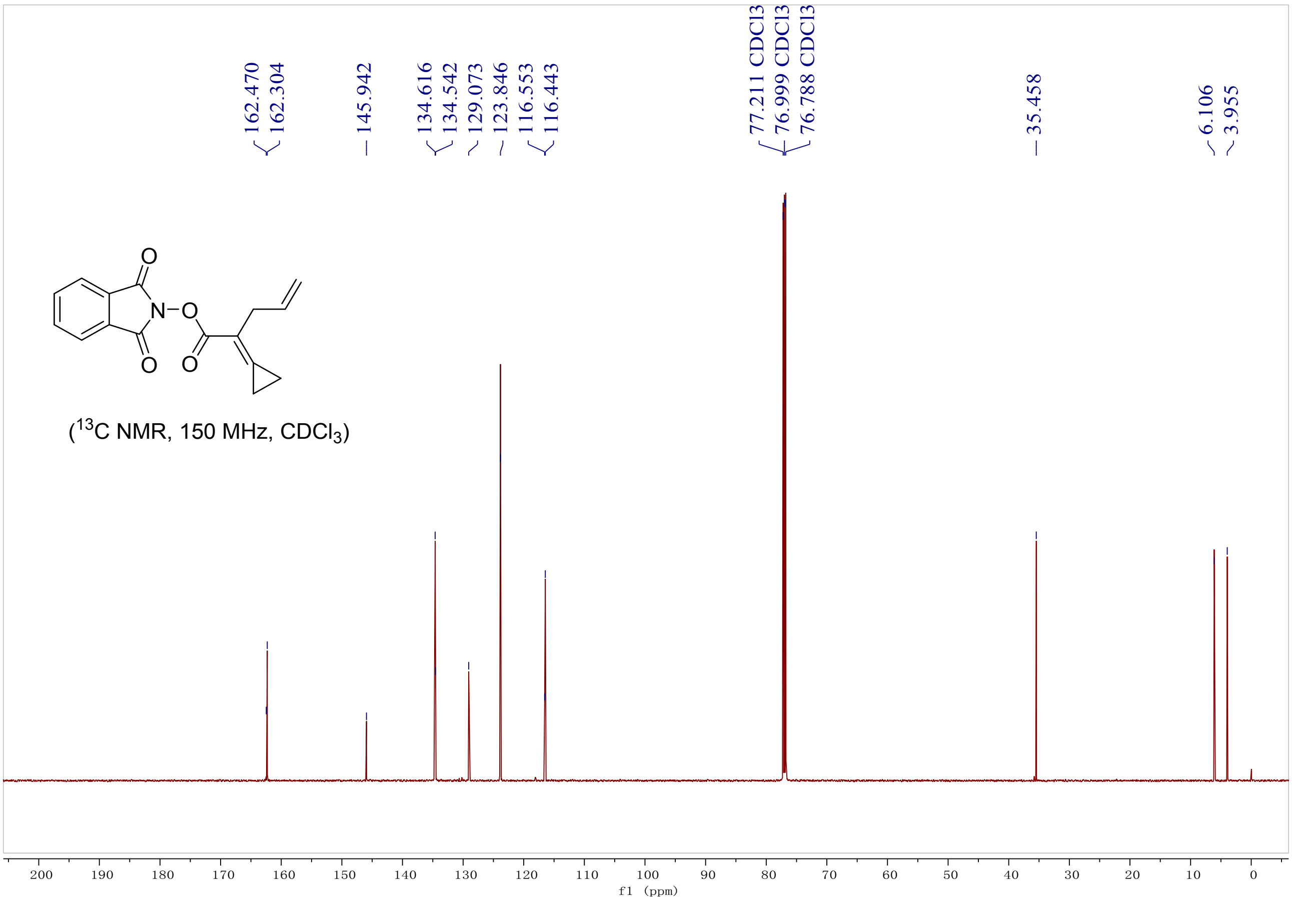
162.470 162.304 145.942 134.616 134.542 129.073 123.846 116.553 116.443 77.211 CDCl3 76.999 CDCl3 76.788 CDCl3 35.458 6.106 3.955
(13C NMR, 150 MHz, CDCl3)
f1 (ppm)

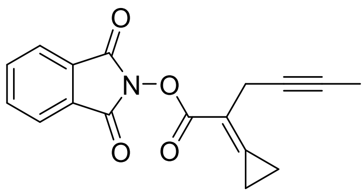

1,3-Dioxoisoindolin-2-yl 2-cyclopropylidenehex-4-ynoate (1j): white solid, m.p. 176-178 °C, 356.3 mg, 60%, Eluent: PE/EA = 10/1.

^{1}H NMR (400 MHz, $CDCl_3$, TMS): δ 7.90 (dd, *J* = 5.6, 3.2 Hz, 2H), 7.79 (dd, *J* = 5.6, 3.2 Hz, 2H), 3.40 (qt, *J* = 2.8, 1.4 Hz, 2H), 1.80 (t, *J* = 2.8 Hz, 3H), 1.66-1.59 (m, 2H), 1.58-1.50 (m, 2H). **^{13}C NMR** (100 MHz, $CDCl_3$, TMS): δ 3.5, 4.5, 5.8, 21.3, 74.6, 77.9, 114.3, 123.9, 129.0, 134.7, 146.7, 162.0, 162.2. **IR** (EtOH): ν 2956, 2922, 2219, 1840, 1798, 1746, 1605, 1420, 1358, 1273, 1214, 1187, 1132, 996, 962 cm^{-1}. **HRMS** (ESI) m/z: $[M+Na]^+$ Calcd. for $C_{17}H_{13}NNaO_4$ 318.0737; found 318.0741.

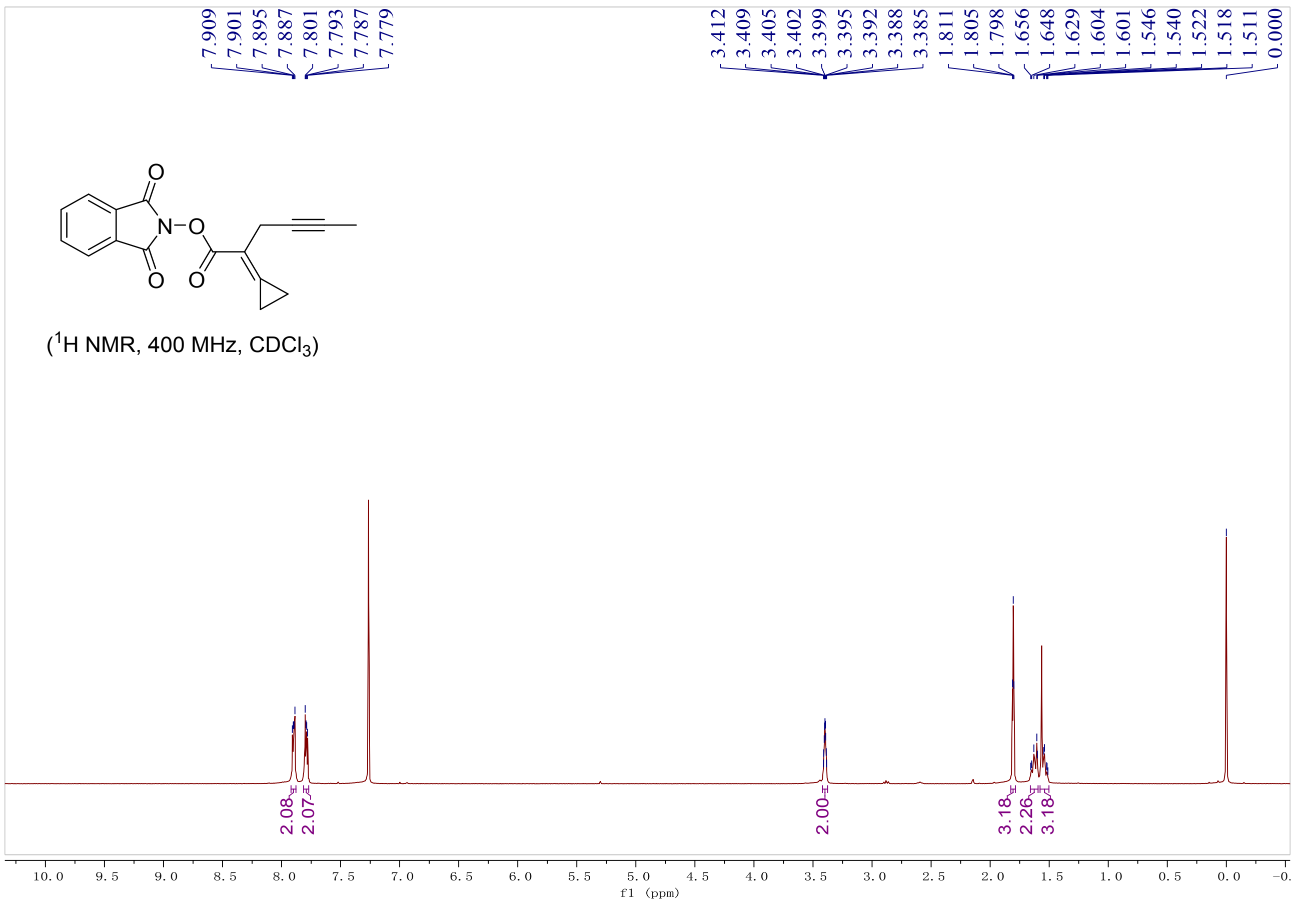

7.909
7.901
7.895
7.887
7.801
7.793
7.787
7.779
3.412
3.409
3.405
3.402
3.399
3.395
3.392
3.388
3.385
1.811
1.805
1.798
1.656
1.648
1.629
1.604
1.601
1.546
1.540
1.522
1.518
1.511
0.000
(¹H NMR, 400 MHz, CDCl3)
2.08
2.07
2.00
3.18
2.26
3.18
f1 (ppm)

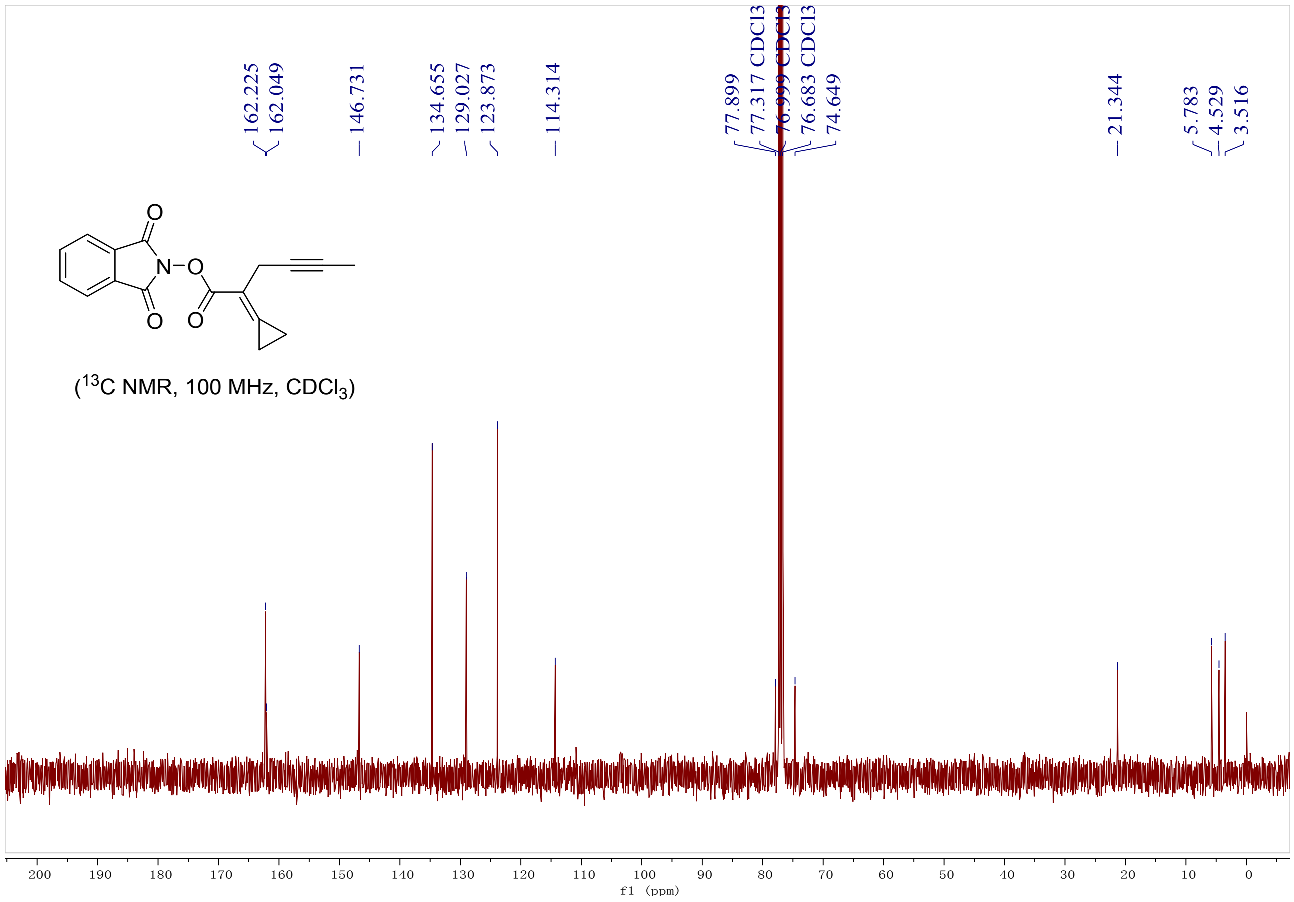

162.225
162.049
146.731
134.655
129.027
123.873
114.314
77.899
77.317 CDCl3
76.999 CDCl3
76.683 CDCl3
74.649
21.344
5.783
4.529
3.516
(¹³C NMR, 100 MHz, CDCl3)
f1 (ppm)

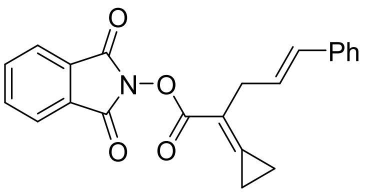

1,3-Dioxoisoindolin-2-yl (*E*)-2-cyclopropylidene-5-phenylpent-4-enoate (1k): white solid, m.p. 189-190 °C, 549.8 mg, 76%, Eluent: PE/EA = 10/1.

^{1}H NMR (400 MHz, $CDCl_3$, TMS): δ 7.90 (dd, *J* = 5.6, 3.2 Hz, 2H), 7.79 (dd, *J* = 5.6, 3.2 Hz, 2H), 7.40-7.34 (m, 2H), 7.30 (t, *J* = 7.6 Hz, 2H), 7.25-7.16 (m, 1H), 6.49 (d, *J* = 15.8 Hz, 1H), 6.35 (dt, *J* = 15.8, 6.8 Hz, 1H), 3.49-3.41 (m, 2H), 1.68-1.61 (m, 2H), 1.47-1.39 (m, 2H). **^{13}C NMR** (100 MHz, $CDCl_3$, TMS): δ 4.1, 6.1, 34.8, 116.8, 123.9, 126.1, 126.3, 127.2, 128.5, 129.0, 131.6, 134.6, 137.3, 146.1, 162.3, 162.5. **IR** (EtOH): ν 3069, 3024, 2920, 1796, 1744, 1602, 1496, 1467, 1371, 1222, 1185, 1159, 1138, 1090, 986, 847, 773 cm^{-1}. **HRMS** (ESI) m/z: $[M+Na]^+$ Calcd. for $C_{22}H_{17}NNaO_4$ 382.1050; found 382.1051.

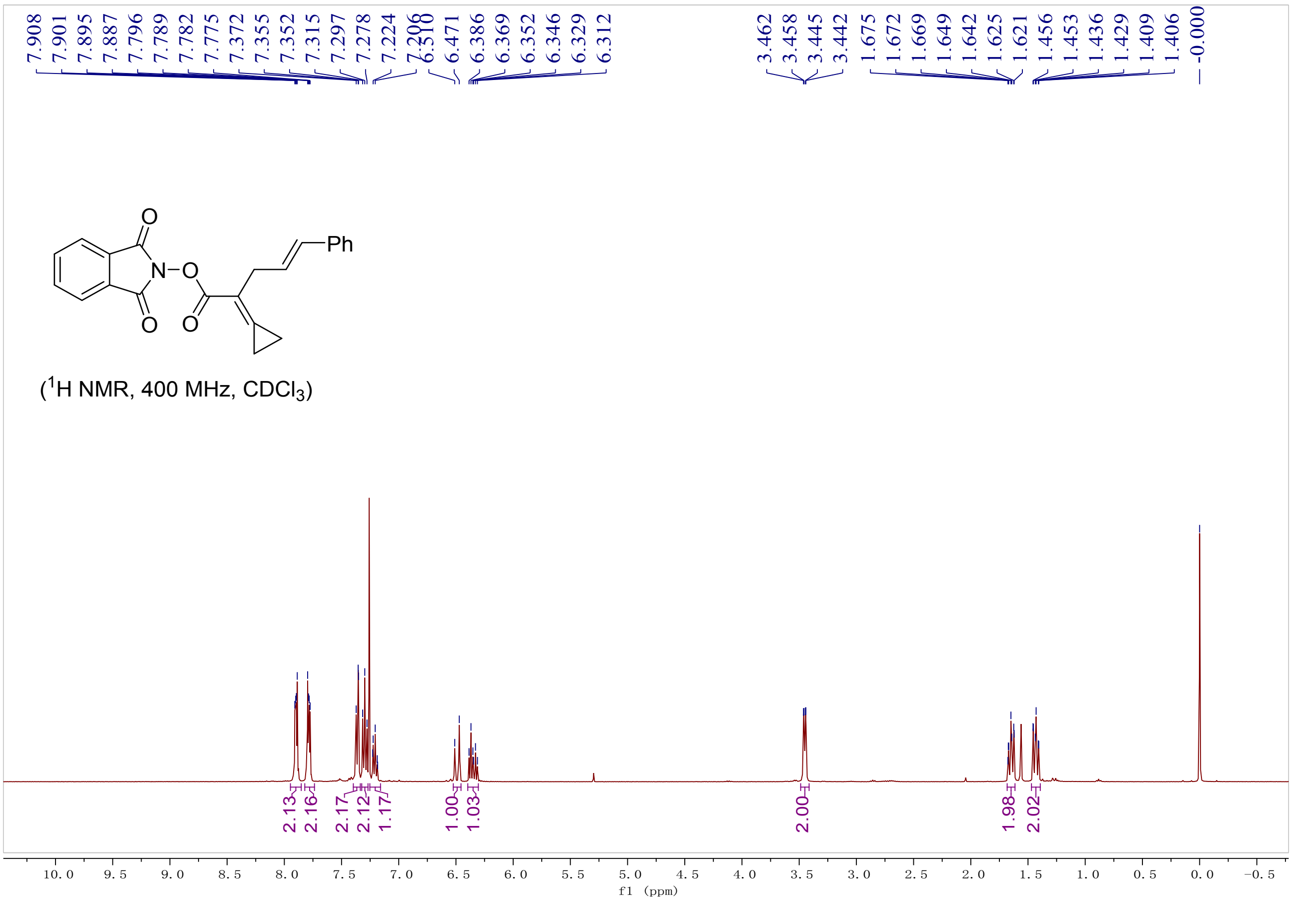

7.908
7.901
7.895
7.887
7.796
7.789
7.782
7.775
7.372
7.355
7.352
7.315
7.297
7.278
7.224
7.206
6.510
6.471
6.386
6.369
6.352
6.346
6.329
6.312
3.462
3.458
3.445
3.442
1.675
1.672
1.669
1.649
1.642
1.625
1.621
1.456
1.453
1.436
1.429
1.409
1.406
-0.000
O
N
O
O
O
Ph
(^{1}H NMR, 400 MHz, $CDCl_3$)
2.13
2.16
2.17
2.12
1.17
1.00
1.03
2.00
1.98
2.02
f1 (ppm)

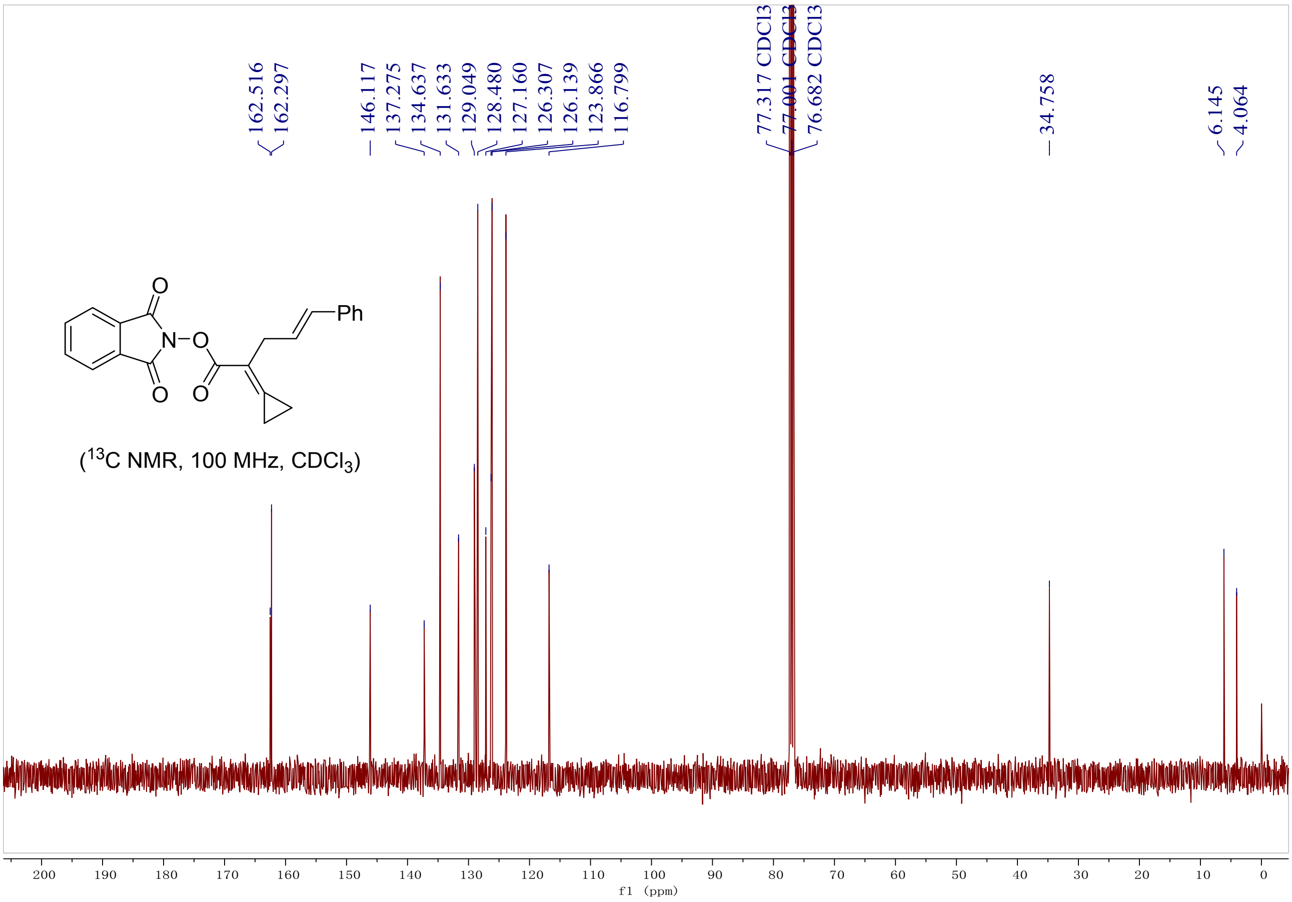

162.516
162.297
146.117
137.275
134.637
131.633
129.049
128.480
127.160
126.307
126.139
123.866
116.799
77.317 CDCl3
77.001 CDCl3
76.682 CDCl3
34.758
6.145
4.064
O
N
O
O
O
Ph
(^{13}C NMR, 100 MHz, $CDCl_3$)
f1 (ppm)

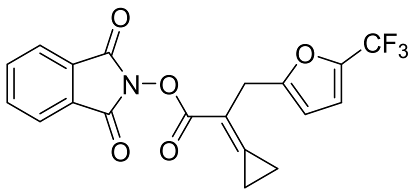

1,3-Dioxoisoindolin-2-yl 2-cyclopropylidene-3-(5-(trifluoromethyl)furan-2-yl)propanoate (1l): white solid, m.p. 185-186 ºC, 493.3 mg, 63%, Eluent: PE/EA = 10/1.

^{1}H NMR (400 MHz, $CDCl_3$, TMS): δ 7.82 (dd, *J* = 5.6, 3.2 Hz, 2H), 7.72 (dd, *J* = 5.6, 3.2 Hz, 2H), 6.64 (dd, *J* = 3.2, 1.4 Hz, 1H), 6.13 (d, *J* = 3.2 Hz, 1H), 3.83 (s, 2H), 1.64-1.53 (m, 2H), 1.24-1.14 (m, 2H). **^{13}C NMR** (100 MHz, $CDCl_3$, TMS): δ 3.8, 6.3, 29.6, 108.0, 112.5, 114.3, 119.1 (q, *J* = 264.7 Hz), 123.9, 128.9, 134.7, 140.7 (q, *J* = 42.5 Hz), 148.1, 155.0 (q, *J* = 1.5 Hz), 162.08, 162.13. ^{19}F NMR (376 MHz, $CDCl_3$): δ -63.9; **IR** (EtOH): ν 3023, 2922, 1796, 1768, 1744, 1467, 1371, 1322, 1185, 1138, 1090, 1016, 852, 767, 699 cm^{-1}. **HRMS** (ESI) m/z: $[M+Na]^+$ Calcd. for $C_{19}H_{12}F_3NNaO_5$ 414.0560; found 414.0562.

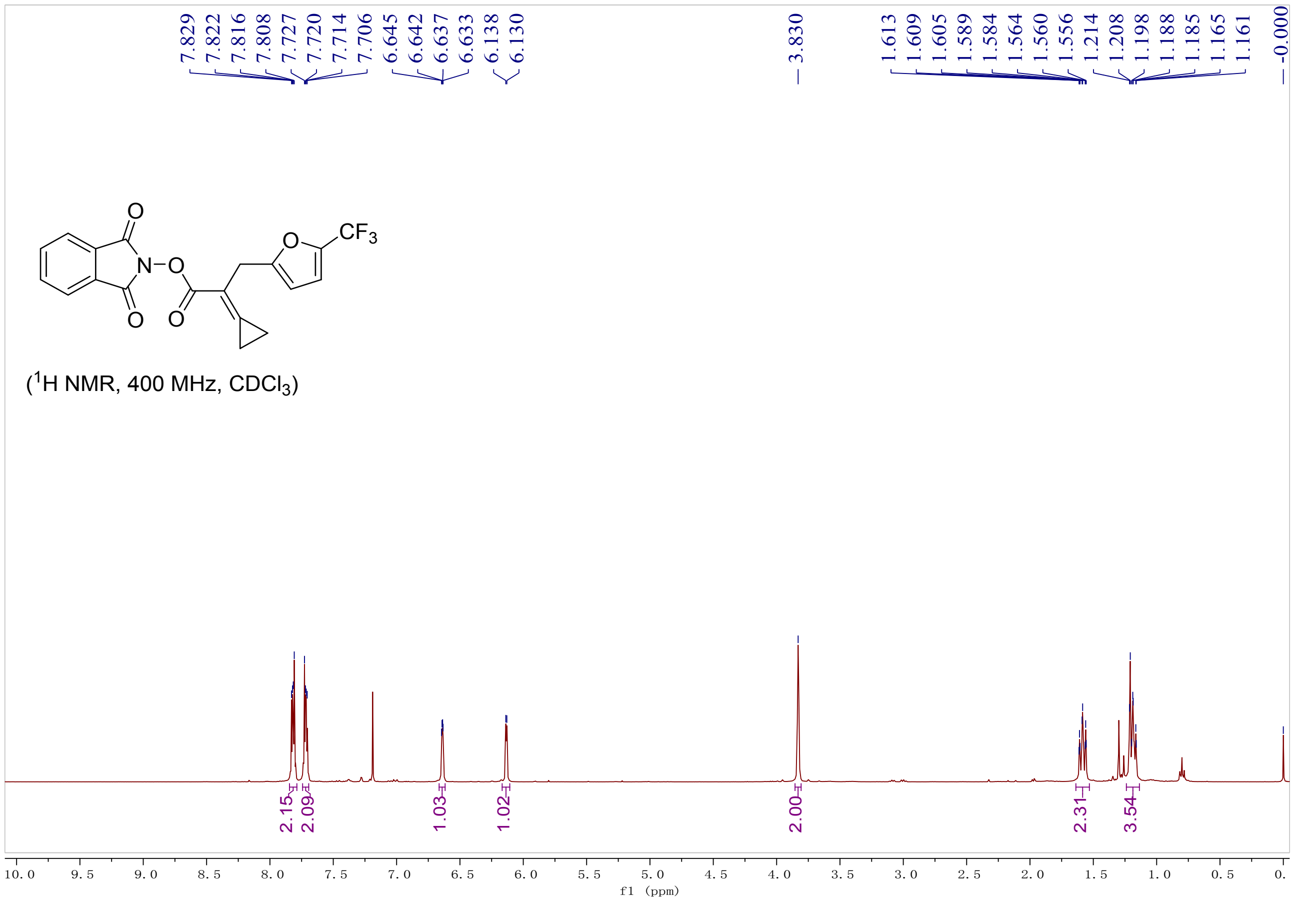
7.829
7.822
7.816
7.808
7.727
7.720
7.714
7.706
6.645
6.642
6.637
6.633
6.138
6.130
3.830
1.613
1.609
1.605
1.589
1.584
1.564
1.560
1.556
1.214
1.208
1.198
1.188
1.185
1.165
1.161
-0.000
O
N–O
O
O
O
CF3
(1H NMR, 400 MHz, CDCl3)
2.15
2.09
1.03
1.02
2.00
2.31
3.54
f1 (ppm)

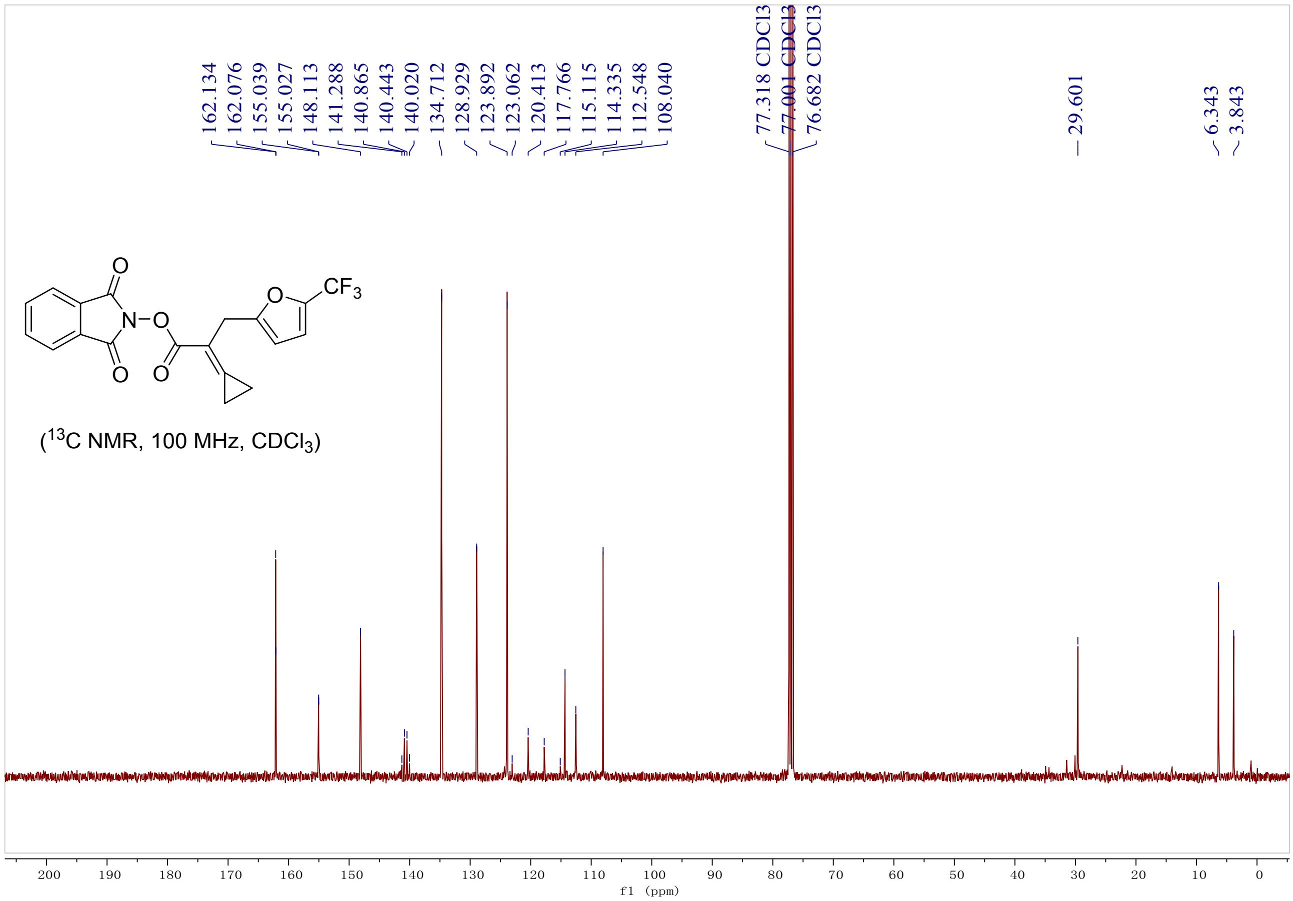
162.134
162.076
155.039
155.027
148.113
141.288
140.865
140.443
140.020
134.712
128.929
123.892
123.062
120.413
117.766
115.115
114.335
112.548
108.040
77.318 CDCl3
77.001 CDCl3
76.682 CDCl3
29.601
6.343
3.843
O
N–O
O
O
O
CF3
(13C NMR, 100 MHz, CDCl3)
f1 (ppm)

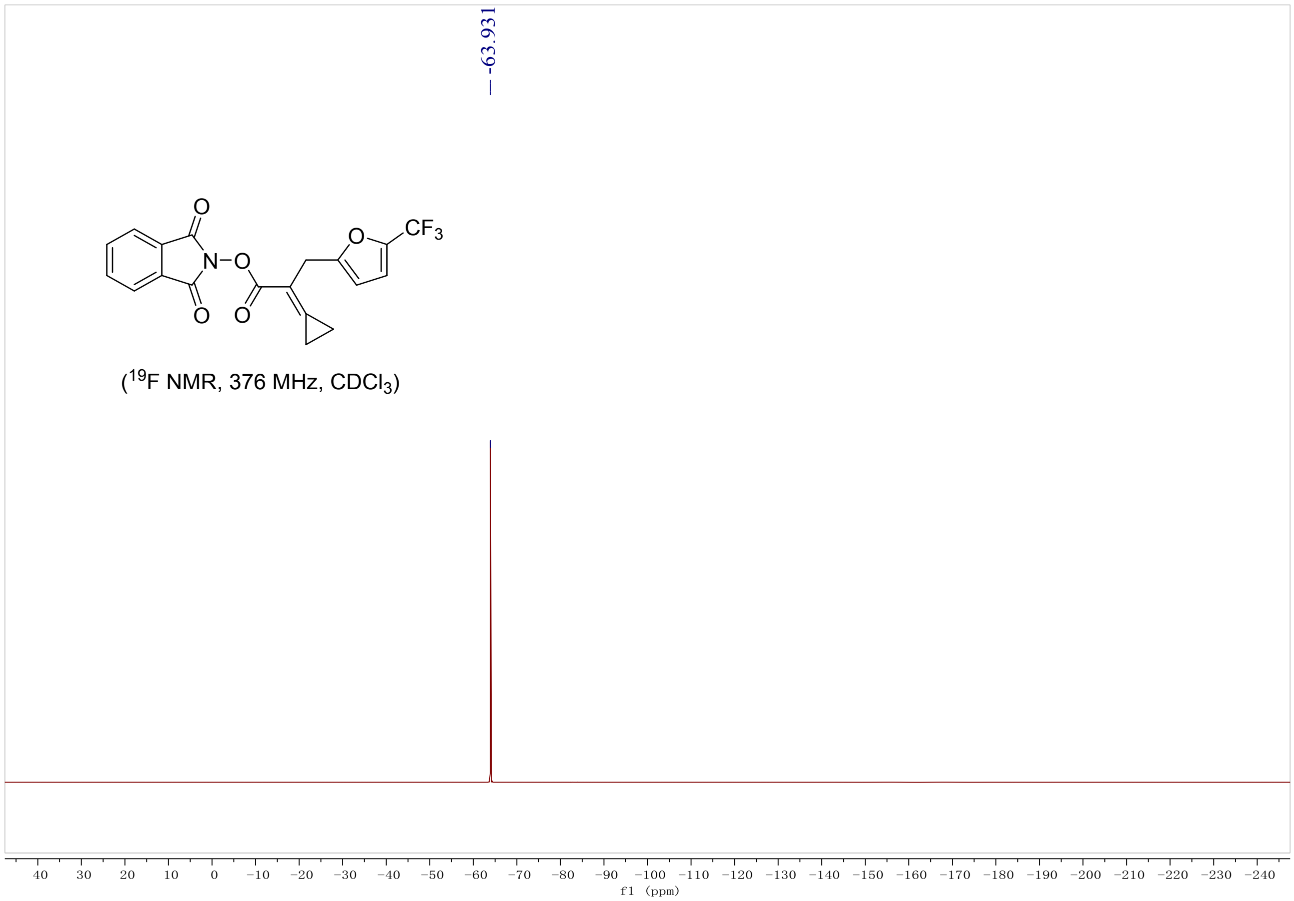

-63.931
O
N
O
O
O
O
CF3
(19F NMR, 376 MHz, CDCl3)
40 30 20 10 0 -10 -20 -30 -40 -50 -60 -70 -80 -90 -100 -110 -120 -130 -140 -150 -160 -170 -180 -190 -200 -210 -220 -230 -240
f1 (ppm)

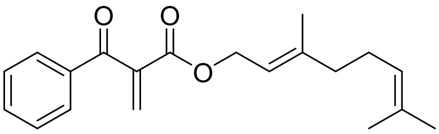

(*E*)-3,7-dimethylocta-2,6-dien-1-yl 2-benzoylacrylate (2ab): colorless oil, 512 mg, 82%, Eluent: PE/EA = 10/1.

^{1}H NMR (600 MHz, $CDCl_3$, TMS): δ 7.88-7.83 (m, 2H), 7.61-7.56 (m, 1H), 7.49-7.43 (m, 2H), 6.68 (s, 1H), 6.07 (s, 1H), 5.27-5.21 (m, 1H), 5.07-5.02 (m, 1H), 4.68 (d, J = 7.2 Hz, 2H), 2.08-2.01 (m, 2H), 2.02-1.96 (m, 2H), 1.67 (s, 3H), 1.62 (s, 3H), 1.59 (s, 3H). **^{13}C NMR** (150 MHz, $CDCl_3$, TMS): δ 16.4, 17.6, 25.6, 26.2, 39.4, 62.3, 117.5, 123.6, 128.5, 129.4, 131.4, 131.8, 133.5, 136.3, 141.5, 143.0, 164.3, 193.1. **IR** (EtOH): ν 2970, 2921, 2896, 2872, 1730, 1679, 1633, 1446, 1379, 1351, 1244, 1180, 1139, 1109, 944, 851, 757, 689 cm^{-1}. **HRMS** (ESI) m/z: $[M+H]^+$ Calcd. for $C_{20}H_{25}O_3$ 313.1798; found 313.1799.

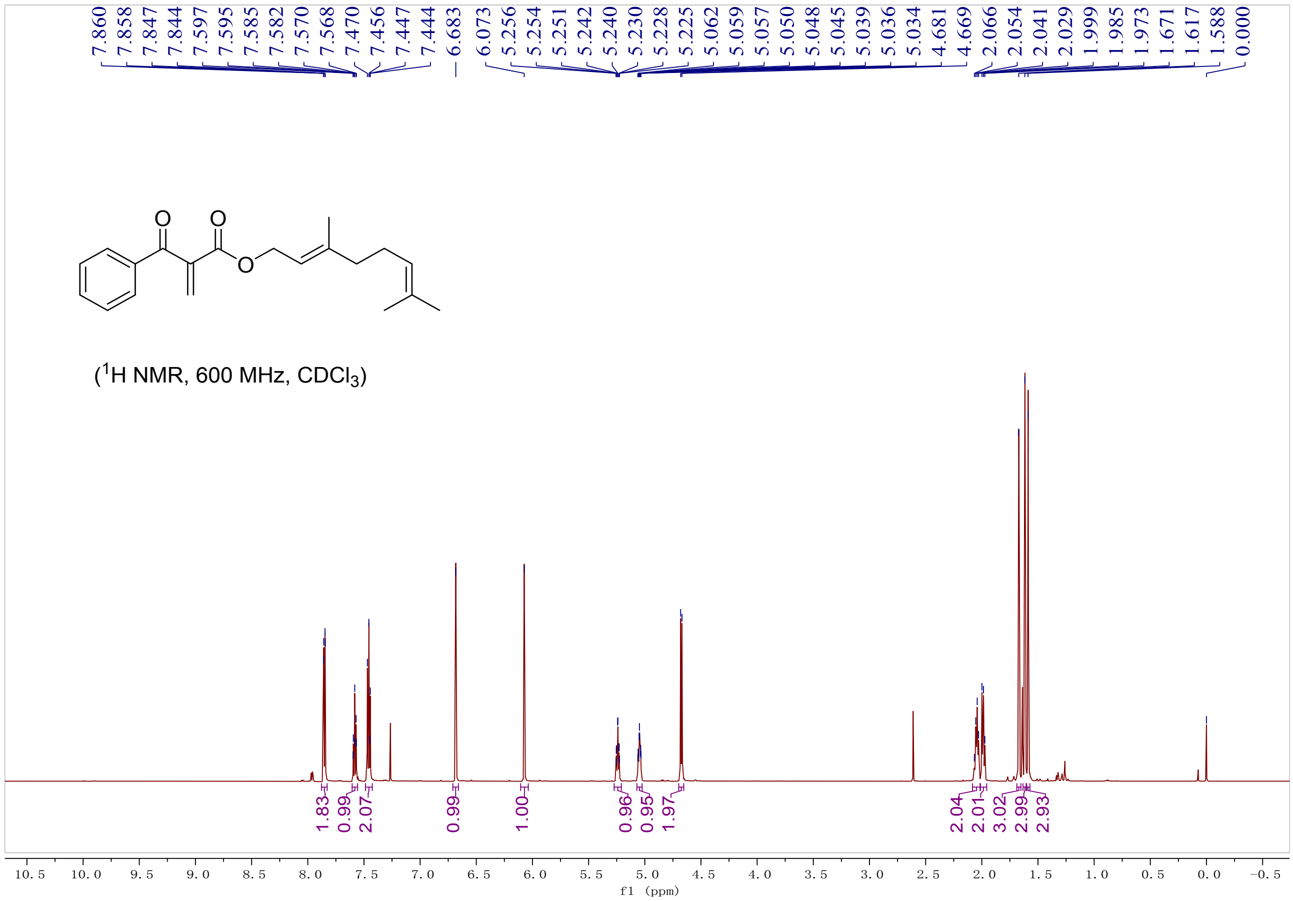
7.860 7.858 7.847 7.844 7.597 7.595 7.585 7.582 7.570 7.568 7.470 7.456 7.447 7.444 6.683 6.073 5.256 5.254 5.251 5.242 5.240 5.230 5.228 5.225 5.062 5.059 5.057 5.050 5.048 5.045 5.039 5.036 5.034 4.681 4.669 2.066 2.054 2.041 2.029 1.999 1.985 1.973 1.671 1.617 1.588 0.000
O
O
O
(1H NMR, 600 MHz, CDCl3)
1.83 0.99 2.07 0.99 1.00 0.96 0.95 1.97 2.04 2.01 3.02 2.99 2.93
f1 (ppm)

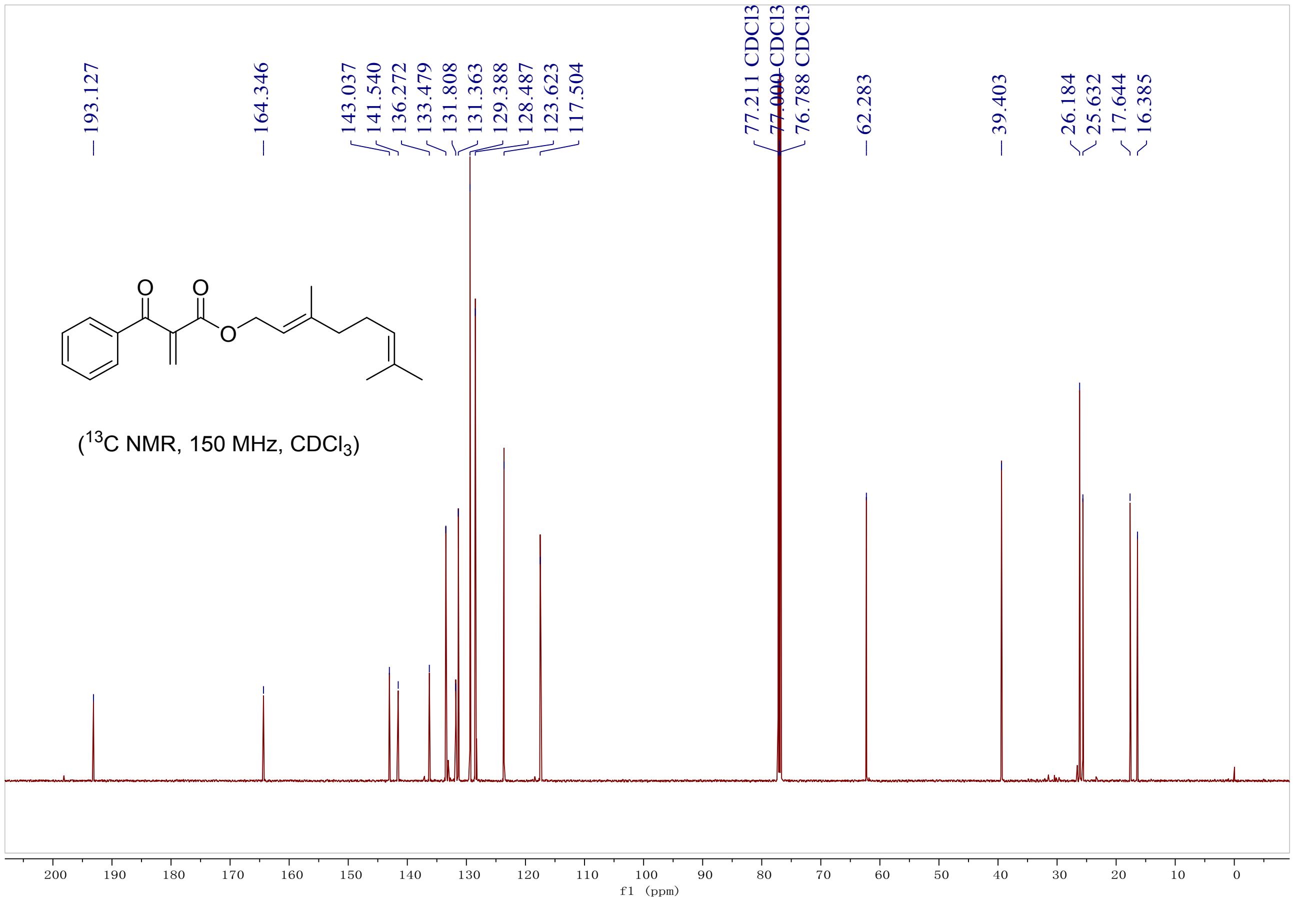
193.127 164.346 143.037 141.540 136.272 133.479 131.808 131.363 129.388 128.487 123.623 117.504 77.211 CDCl3 77.000 CDCl3 76.788 CDCl3 62.283 39.403 26.184 25.632 17.644 16.385
O
O
O
(13C NMR, 150 MHz, CDCl3)
f1 (ppm)

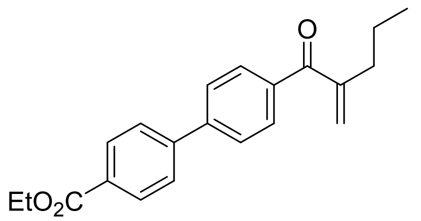

Ethyl 4'-(2-methylenepentanoyl)-[1,1'-biphenyl]-4-carboxylate (2ac): colorless oil, 206 mg, 32%, Eluent: PE/EA = 10/1.

^{1}H NMR (600 MHz, $CDCl_3$, TMS): δ 8.14 (d, *J* = 8.4 Hz, 2H), 7.87 (d, *J* = 8.4 Hz, 2H), 7.69 (d, *J* = 8.4 Hz, 4H), 5.87-5.84 (m, 1H), 5.63 (s, 1H), 4.41 (q, *J* = 7.2 Hz, 2H), 2.50-2.46 (m, 2H), 1.59-1.52 (m, 2H), 1.42 (t, *J* = 7.2 Hz, 3H), 0.99 (t, *J* = 7.4 Hz, 3H). **^{13}C NMR** (150 MHz, $CDCl_3$, TMS): δ 13.8, 14.3, 21.4, 34.4, 61.1, 125.2, 127.06, 127.15, 130.16, 130.20, 137.3, 143.7, 144.3, 148.3, 166.3, 197.9. **IR** (EtOH): ν 2988, 2959, 1707, 1642, 1601, 1332, 1274, 1171, 1102, 984, 849, 767, 699 cm^{-1}. **HRMS** (ESI) m/z: $[M]^+$ Calcd. for $C_{21}H_{22}O_3$ 322.1563; found 322.1559.

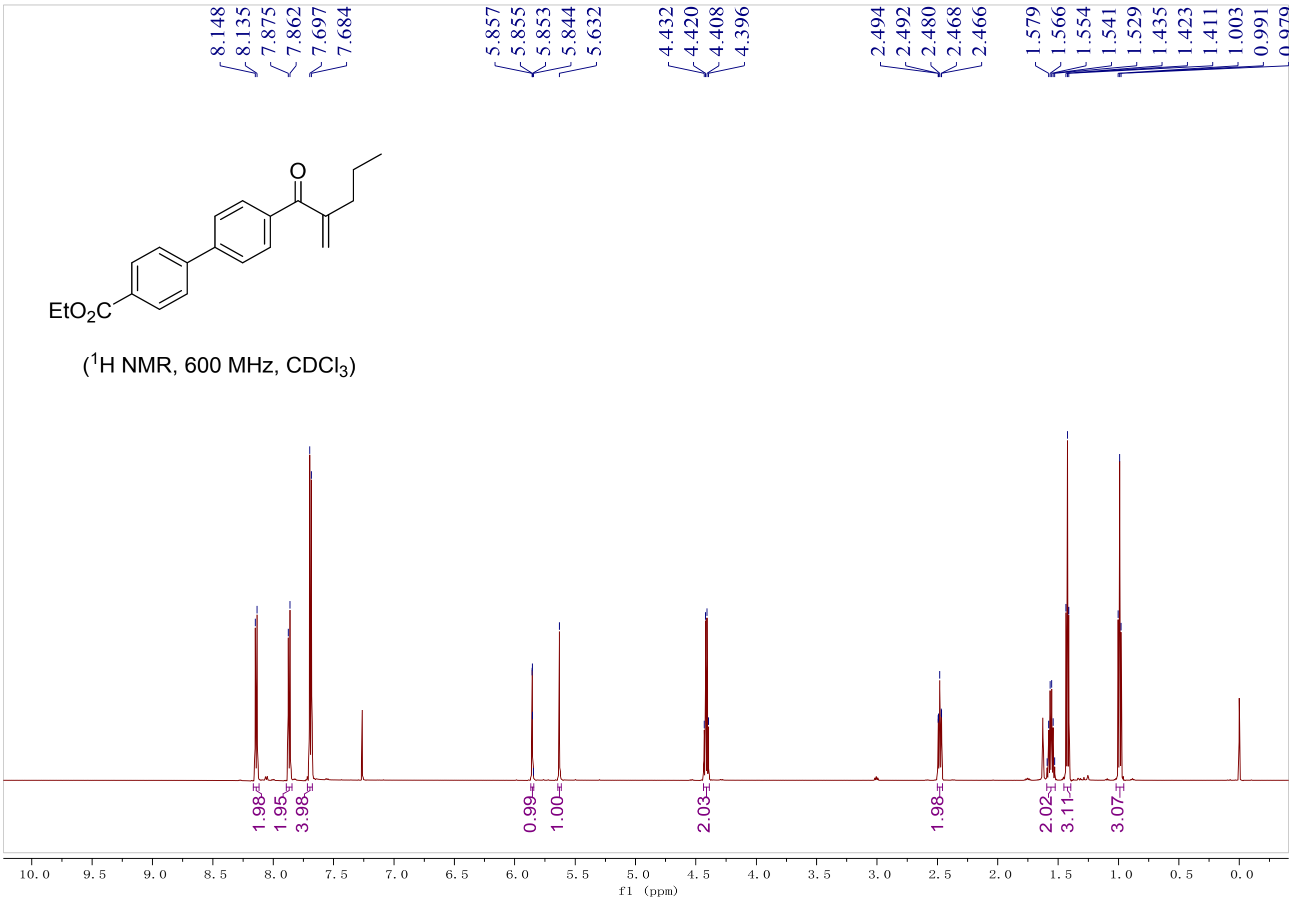
8.148
8.135
7.875
7.862
7.697
7.684
5.857
5.855
5.853
5.844
5.632
4.432
4.420
4.408
4.396
2.494
2.492
2.480
2.468
2.466
1.579
1.566
1.554
1.541
1.529
1.435
1.423
1.411
1.003
0.991
EtO2C
O
(1H NMR, 600 MHz, CDCl3)
1.98
1.95
3.98
0.99
1.00
2.03
1.98
2.02
3.11
3.07
f1 (ppm)

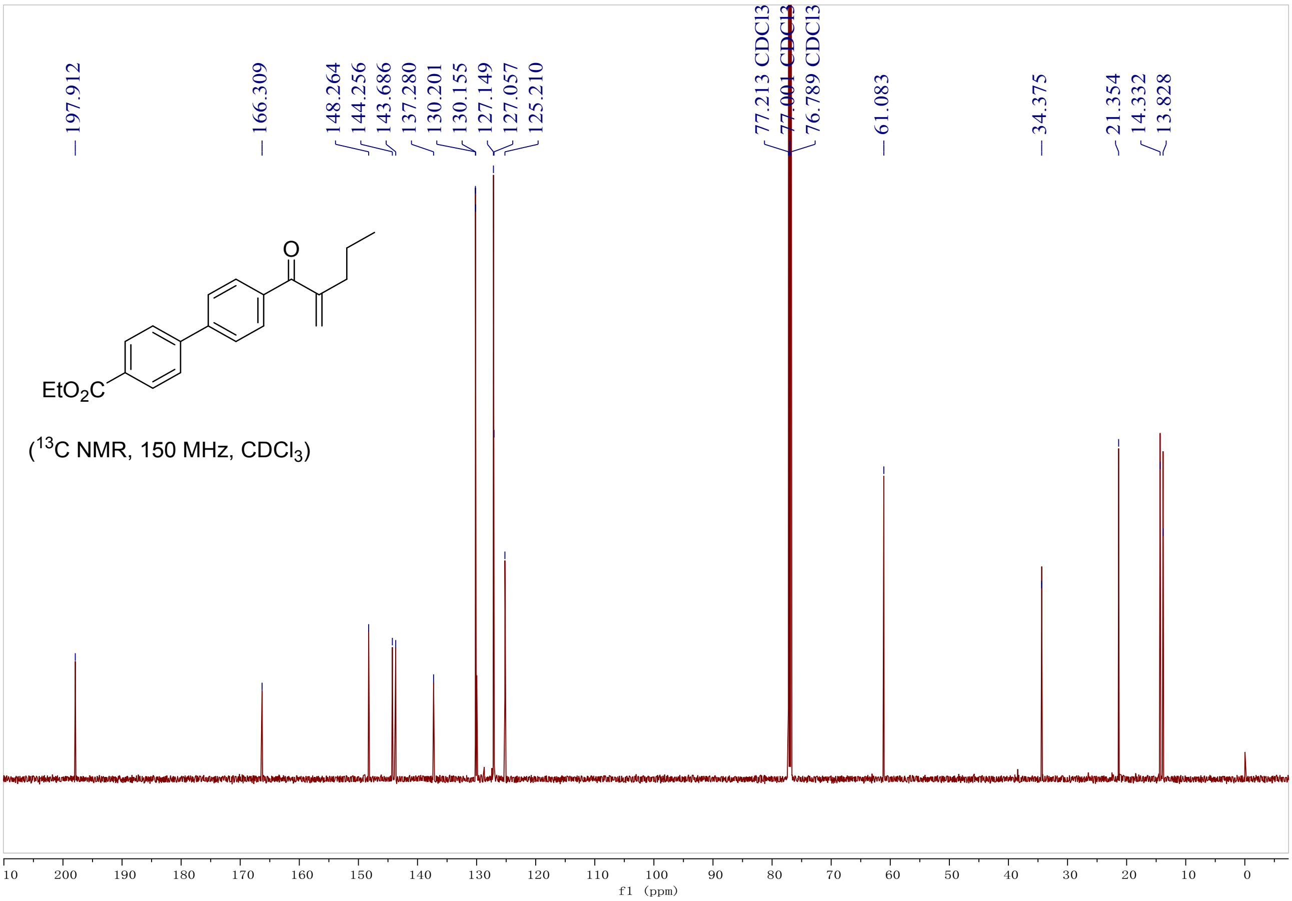
197.912
166.309
148.264
144.256
143.686
137.280
130.201
130.155
127.149
127.057
125.210
77.213 CDCl3
76.789 CDCl3
61.083
34.375
21.354
14.332
13.828
EtO2C
O
(13C NMR, 150 MHz, CDCl3)
f1 (ppm)

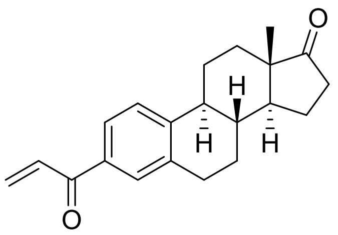

(8*R*,9*S*,13*S*,14*S*)-3-acryloyl-13-methyl-6,7,8,9,11,12,13,14,15,16-decahydro-17*H*-cyclopenta[*a*]phenanthren-17-one (2ad): colorless solid, m.p. 205-206 °C, 259 mg, 42%, Eluent: PE/EA = 30/1.

^{1}H NMR (400 MHz, $CDCl_3$, TMS): δ 7.76-7.69 (m, 2H), 7.42-7.40 (m, 1H), 7.17 (dd, *J* = 17.2, 10.4 Hz, 1H), 6.44 (dd, *J* = 17.2, 1.8 Hz, 1H), 5.91 (dd, *J* = 10.4, 1.8 Hz, 1H), 3.02-2.96 (m, 2H), 2.58-2.45 (m, 2H), 2.36 (td, *J* = 10.8, 4.2 Hz, 1H), 2.21-2.14 (m, 1H), 2.11-2.05 (m, 2H), 2.02-1.97 (m, 1H), 1.73-1.46 (m, 6H), 0.93 (s, 3H). **^{13}C NMR** (150 MHz, $CDCl_3$, TMS): δ 13.8, 21.6, 25.6, 26.3, 31.5, 35.8, 37.8, 44.7, 47.9, 50.5, 125.6, 126.2, 129.4, 129.7, 132.4, 134.9, 137.1, 145.4, 190.7, 220.5. **IR** (EtOH): ν 3002, 2959, 2899, 2863, 1707, 1643, 1553, 1274, 1202, 1171, 1103, 1004, 984, 944, 850,736 cm^{-1}. **HRMS** (EI) m/z: $[M]^+$ Calcd. for $C_{21}H_{24}O_2$ 308.1771; found 308.1766.

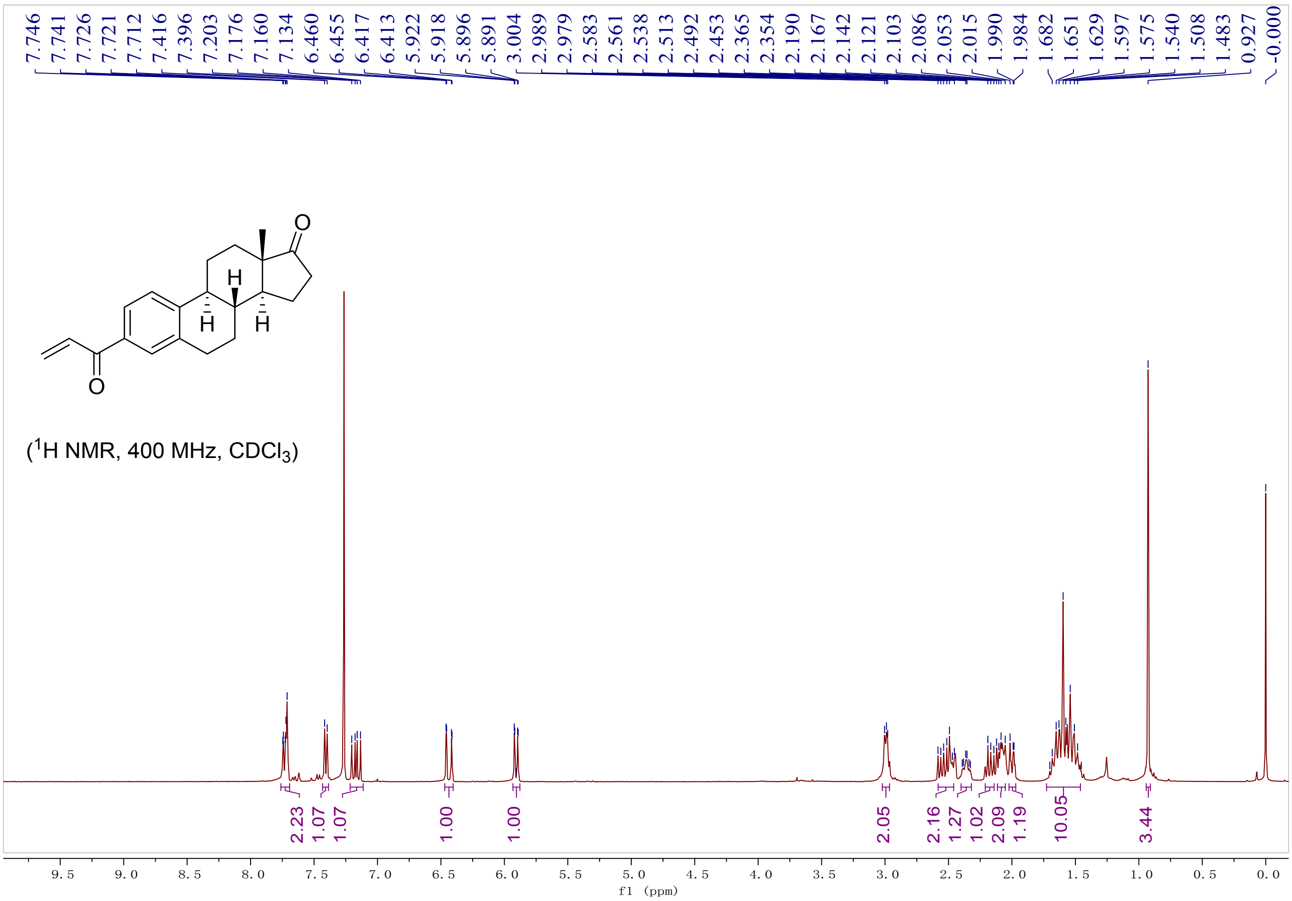

7.746
7.741
7.726
7.721
7.712
7.416
7.396
7.203
7.176
7.160
7.134
6.460
6.455
6.417
6.413
5.922
5.918
5.896
5.891
3.004
2.989
2.979
2.583
2.561
2.538
2.513
2.492
2.453
2.365
2.354
2.190
2.167
2.142
2.121
2.103
2.086
2.053
2.015
1.990
1.984
1.682
1.651
1.629
1.597
1.575
1.540
1.508
1.483
0.927
-0.000
(¹H NMR, 400 MHz, CDCl3)
2.23
1.07
1.07
1.00
1.00
2.05
2.16
1.27
1.02
2.09
1.19
10.05
3.44
f1 (ppm)

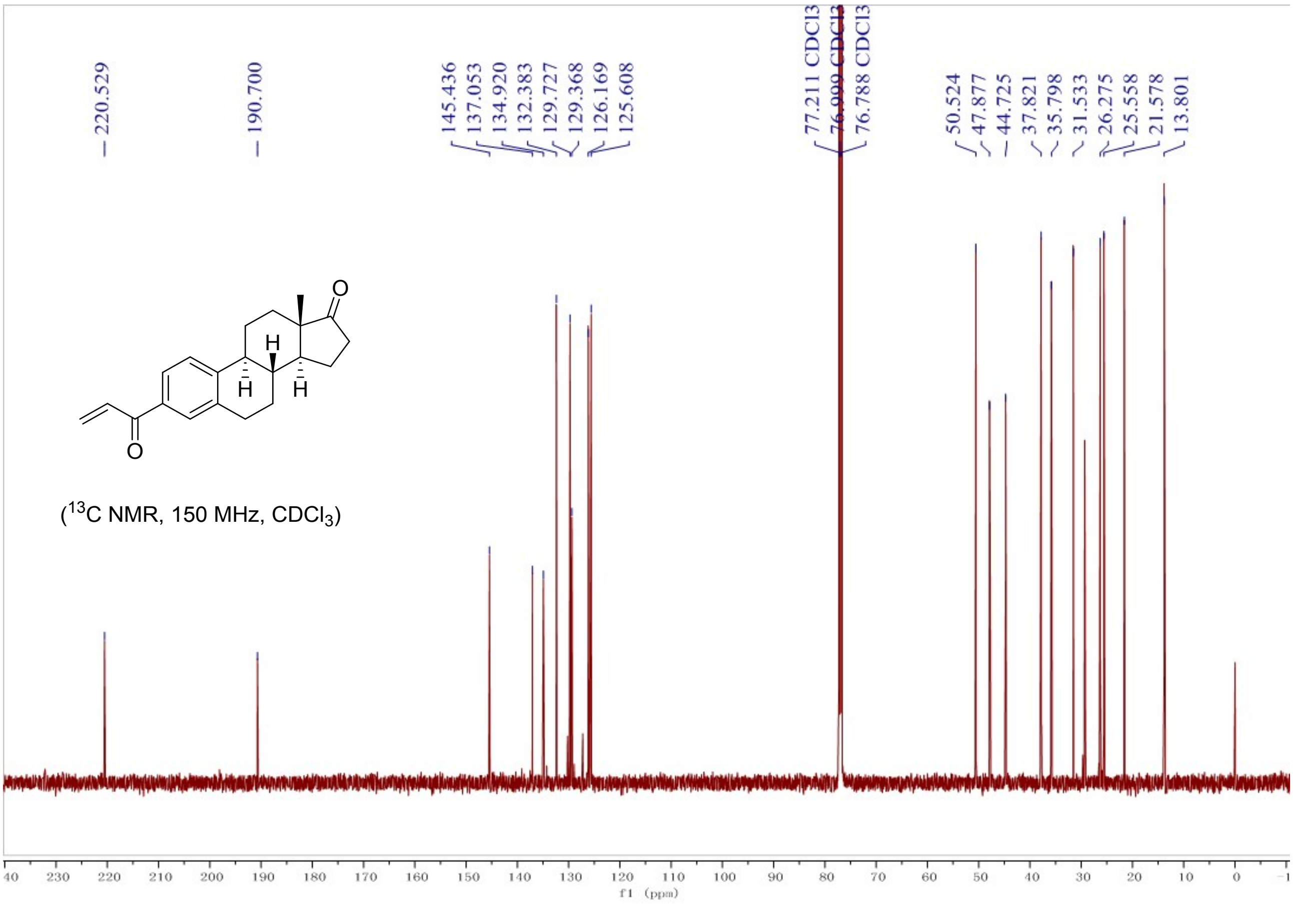

220.529
190.700
145.436
137.053
134.920
132.383
129.727
129.368
126.169
125.608
77.211 CDCl3
76.999 CDCl3
76.788 CDCl3
50.524
47.877
44.725
37.821
35.798
31.533
26.275
25.558
21.578
13.801
(¹³C NMR, 150 MHz, CDCl3)
f1 (ppm)

7. The Characterization Data of Products

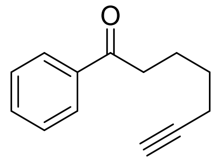

1-phenylhept-6-yn-1-one (3aa): colorless oil, 28 mg, 75%, Eluent: PE/EA = 20/1.

^{1}H NMR (400 MHz, $CDCl_3$, TMS): δ 8.01-7.93 (m, 2H), 7.61-7.52 (m, 1H), 7.51-7.42 (m, 2H), 3.01 (t, J = 7.2 Hz, 2H), 2.26 (td, J = 7.2, 2.6 Hz, 2H), 1.96 (t, J = 2.6 Hz, 1H), 1.91-1.84 (m, 2H), 1.68-1.58 (m, 2H). **^{13}C NMR** (100 MHz, $CDCl_3$, TMS): δ 18.3, 23.3, 28.0, 37.9, 68.6, 84.1, 128.0, 128.6, 133.0, 136.9, 200.0. **IR** (EtOH): ν 3296, 2939, 2872, 2210, 1682, 1597, 1448, 1355, 1223, 1180, 1075, 974, 751, 689 cm^{-1}. **HRMS** (ESI) m/z: $[M]^+$ Calcd. for $C_{13}H_{14}O$ 186.1039; found 186.1037.

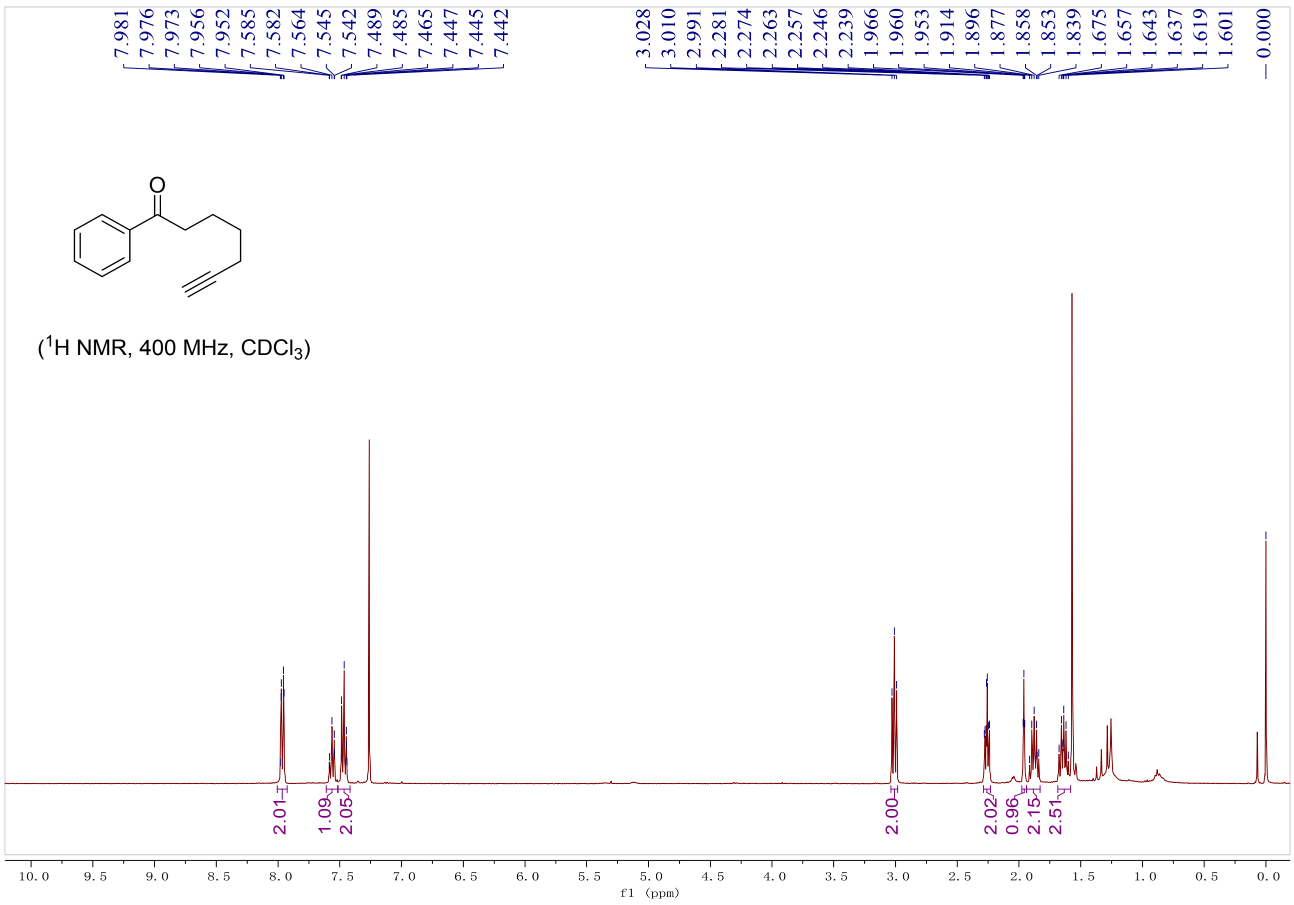
7.981
7.976
7.973
7.956
7.952
7.585
7.582
7.564
7.545
7.542
7.489
7.485
7.465
7.447
7.445
7.442
3.028
3.010
2.991
2.281
2.274
2.263
2.257
2.246
2.239
1.966
1.960
1.953
1.914
1.896
1.877
1.858
1.853
1.839
1.675
1.657
1.643
1.637
1.619
1.601
0.000
O
(^{1}H NMR, 400 MHz, $CDCl_3$)
2.01
1.09
2.05
2.00
2.02
0.96
2.15
2.51
f1 (ppm)

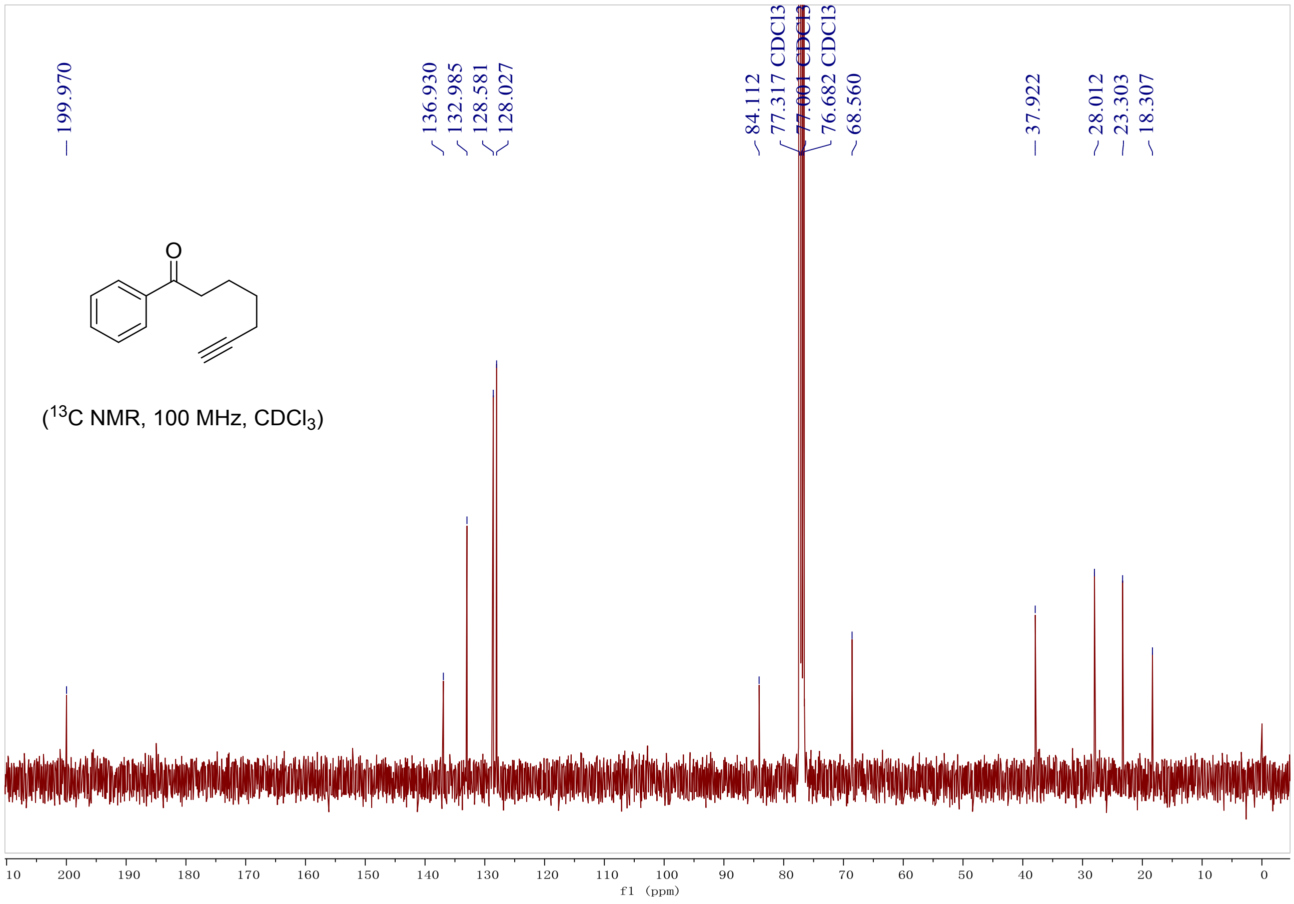
199.970
136.930
132.985
128.581
128.027
84.112
77.317 CDCl3
77.001 CDCl3
76.682 CDCl3
68.560
37.922
28.012
23.303
18.307
O
(^{13}C NMR, 100 MHz, $CDCl_3$)
f1 (ppm)

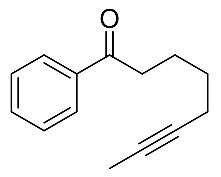

1-phenyloct-6-yn-1-one (3ba): colorless oil, 34 mg, 85%, Eluent: PE/EA = 20/1.

^{1}H NMR (400 MHz, $CDCl_3$, TMS): δ 7.99-7.94 (m, 2H), 7.59-7.53 (m, 1H), 7.51-7.42 (m, 2H), 3.00 (t, *J* = 7.4 Hz, 2H), 2.19 (tq, *J* = 7.2, 2.6 Hz, 2H), 1.90-1.80 (m, 2H), 1.77 (t, *J* = 2.6 Hz, 3H), 1.62-1.56 (m, 2H). **^{13}C NMR** (100 MHz, $CDCl_3$, TMS): δ 3.5, 18.6, 23.5, 28.6, 38.0, 75.9, 78.7, 128.0, 128.5, 132.9, 137.0, 200.2. **IR** (EtOH): ν 2939, 2866, 2129, 1682, 1597, 1448, 1278, 1223, 1179, 1002, 752, 737, 690 cm^{-1}. **HRMS** (ESI) m/z: $[M+H]^+$ Calcd. for $C_{14}H_{17}O$ 201.1274; found 201.1275.

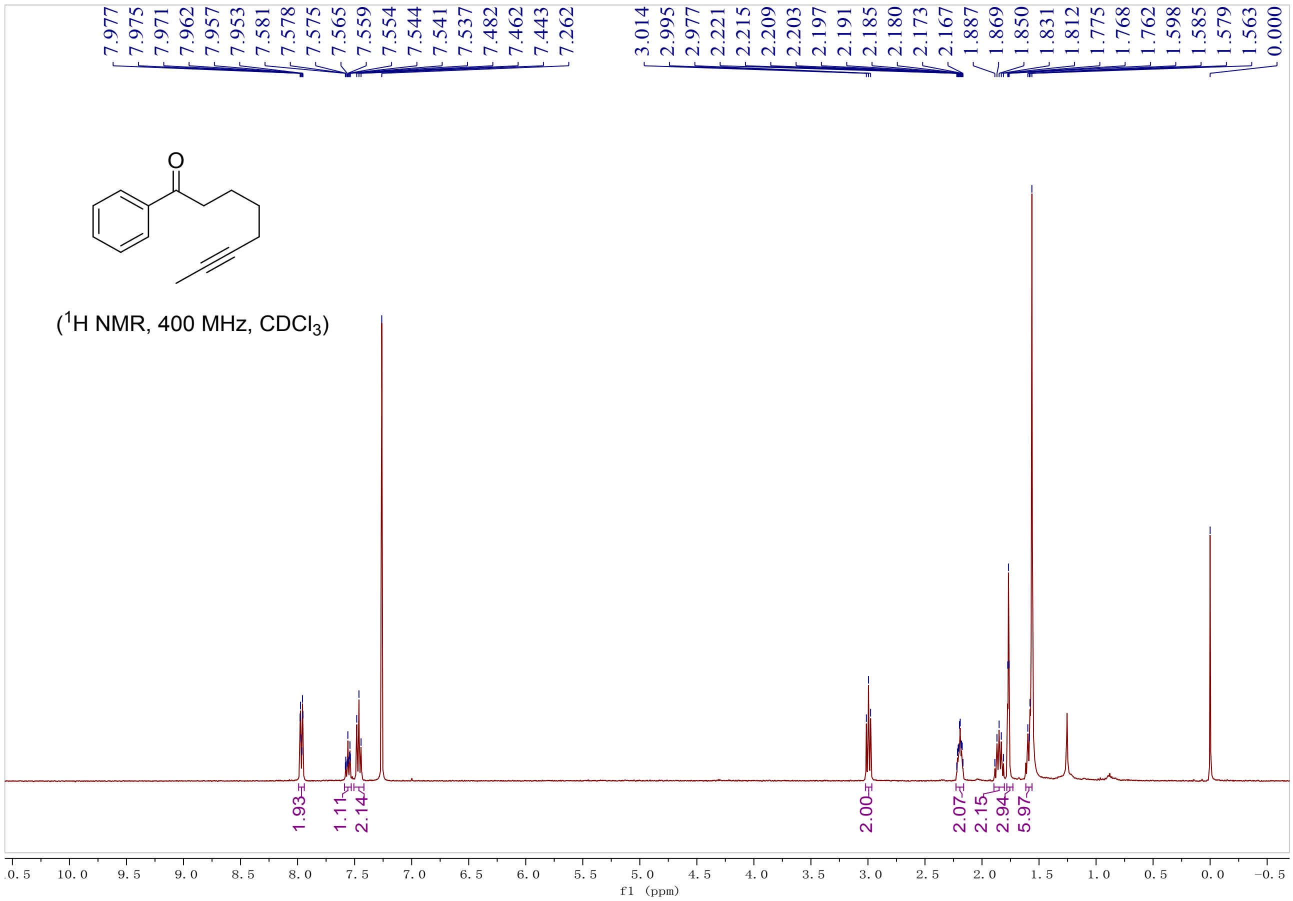
7.977
7.975
7.971
7.962
7.957
7.953
7.581
7.578
7.575
7.565
7.559
7.554
7.544
7.541
7.537
7.482
7.462
7.443
7.262
3.014
2.995
2.977
2.221
2.215
2.209
2.203
2.197
2.191
2.185
2.180
2.173
2.167
1.887
1.869
1.850
1.831
1.812
1.775
1.768
1.762
1.598
1.585
1.579
1.563
0.000
O
(1H NMR, 400 MHz, CDCl3)
1.93
1.11
2.14
2.00
2.07
2.15
2.94
5.97
f1 (ppm)

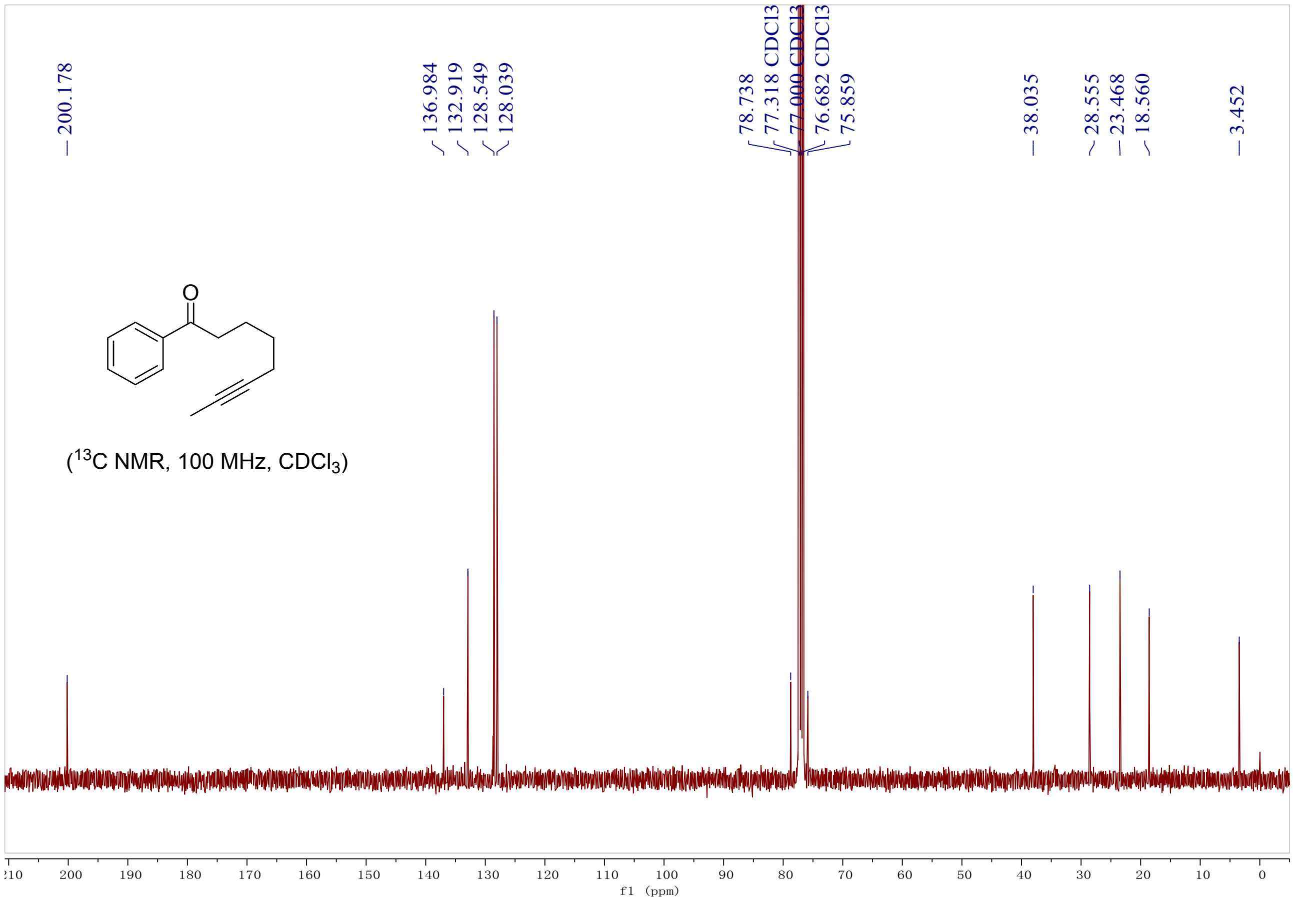
200.178
136.984
132.919
128.549
128.039
78.738
77.318 CDCl3
77.000 CDCl3
76.682 CDCl3
75.859
38.035
28.555
23.468
18.560
3.452
O
(13C NMR, 100 MHz, CDCl3)
f1 (ppm)

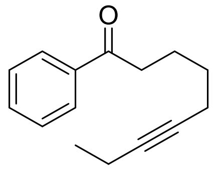

1-phenylnon-6-yn-1-one (3ca): colorless oil, 38 mg, 89%, Eluent: PE/EA = 20/1.

^{1}H NMR (400 MHz, $CDCl_3$, TMS): δ 8.00-7.93 (m, 2H), 7.61-7.51 (m, 1H), 7.51-7.42 (m, 2H), 3.00 (t, J = 7.4 Hz, 2H), 2.25-2.18 (m, 2H), 2.18-2.11 (m, 2H), 1.89-1.81 (m, 2H), 1.65-1.52 (m, 2H), 1.10 (t, J = 7.4 Hz, 3H). **^{13}C NMR** (100 MHz, $CDCl_3$, TMS): δ 12.4, 14.3, 18.6, 23.5, 28.6, 38.1, 78.9, 82.1, 128.0, 128.6, 132.9, 137.0, 200.2. **IR** (EtOH): ν 2941, 2925, 2201, 1685, 1599, 1580, 1448, 1356, 1285, 1226, 1002, 974, 769, 732, 683 cm^{-1}. **HRMS** (ESI) m/z: $[M+Na]^+$ Calcd. for $C_{15}H_{18}ONa$ 237.1250; found 237.1251.

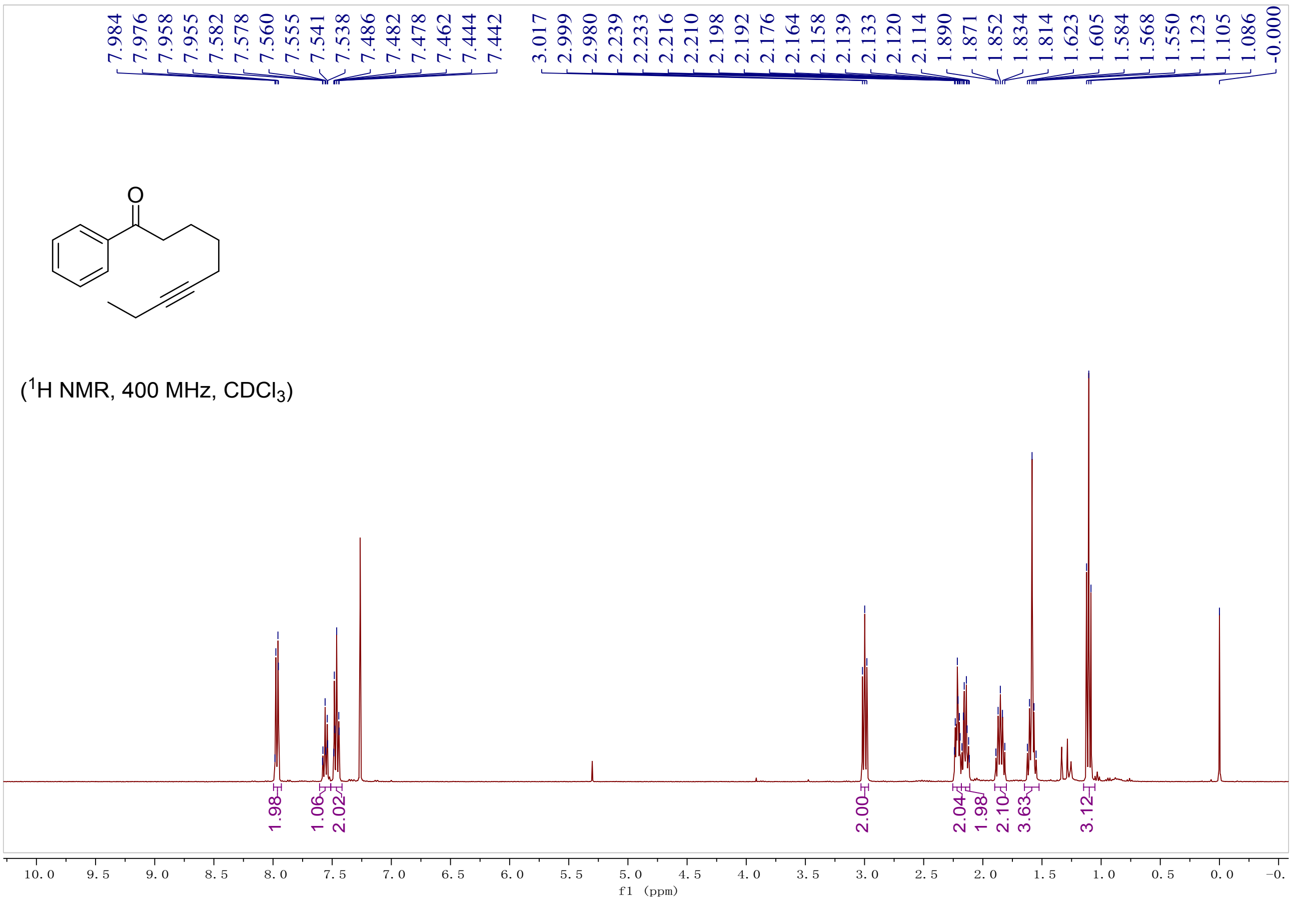
O
7.984 7.976 7.958 7.955 7.582 7.578 7.560 7.555 7.541 7.538 7.486 7.482 7.478 7.462 7.444 7.442
3.017 2.999 2.980 2.239 2.233 2.216 2.210 2.198 2.192 2.176 2.164 2.158 2.139 2.133 2.120 2.114 1.890 1.871 1.852 1.834 1.814 1.623 1.605 1.584 1.568 1.550 1.123 1.105 1.086 -0.000
(¹H NMR, 400 MHz, CDCl3)
1.98 1.06 2.02 2.00 2.04 1.98 2.10 3.63 3.12
f1 (ppm)

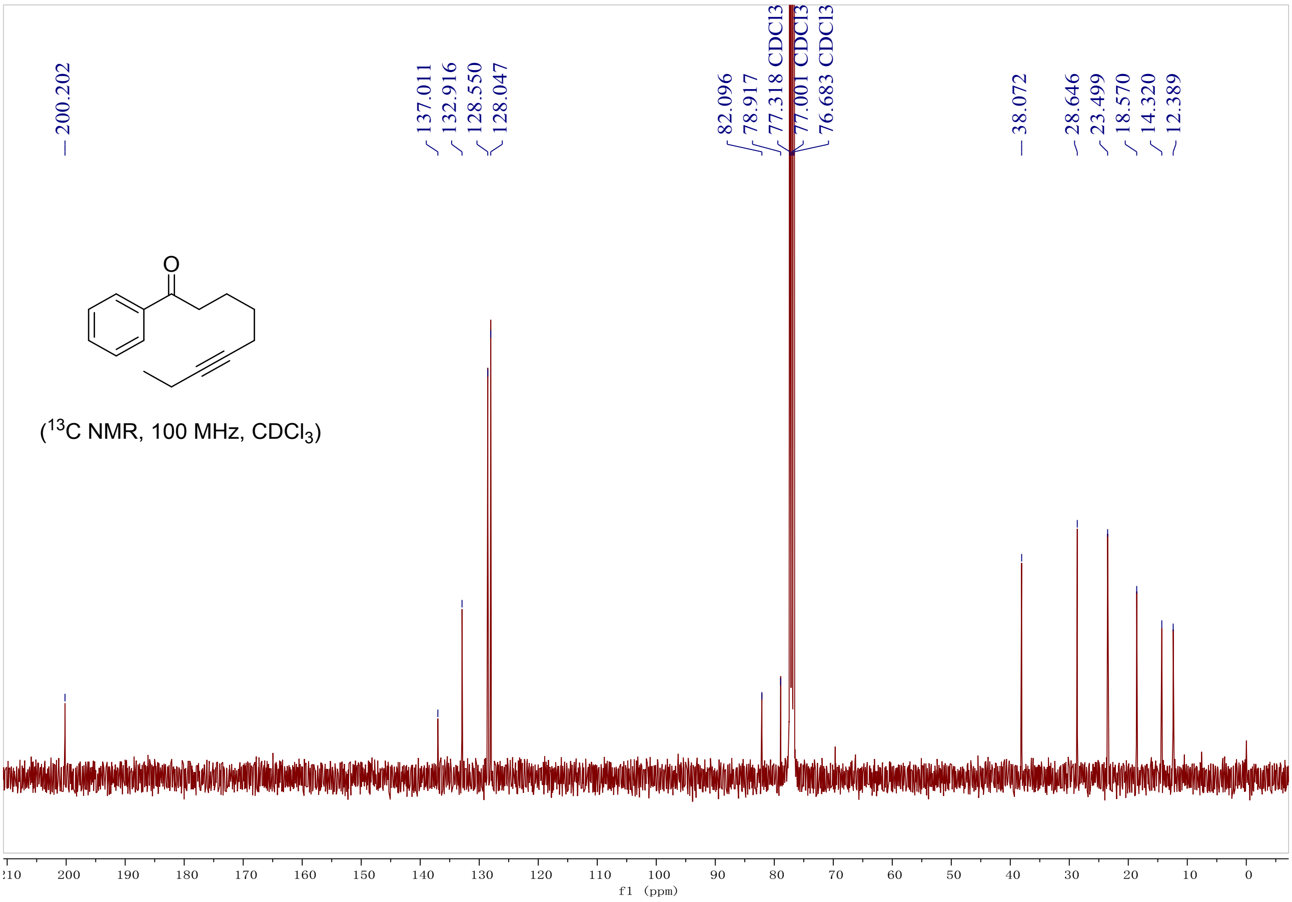
200.202
137.011 132.916 128.550 128.047
82.096 78.917 77.318 CDCl3 77.001 CDCl3 76.683 CDCl3
38.072
28.646 23.499 18.570 14.320 12.389
O
(¹³C NMR, 100 MHz, CDCl3)
f1 (ppm)

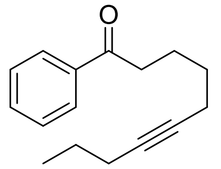

1-phenyldec-6-yn-1-one (3da): colorless oil, 42 mg, 92%, Eluent: PE/EA = 20/1.

^{1}H NMR (400 MHz, $CDCl_3$, TMS): δ 8.01-7.93 (m, 2H), 7.61-7.51 (m, 1H), 7.51-7.42 (m, 2H), 3.00 (t, *J* = 7.4 Hz, 2H), 2.22 (tt, *J* = 7.0, 2.4 Hz, 2H), 2.11 (tt, *J* = 7.0, 2.4 Hz, 2H), 1.92-1.79 (m, 2H), 1.63-1.54 (m, 2H), 1.49 (h, *J* = 7.2 Hz, 2H), 0.96 (t, *J* = 7.4 Hz, 3H). **^{13}C NMR** (100 MHz, $CDCl_3$, TMS): δ 13.5, 18.6, 20.7, 22.5, 23.5, 28.7, 38.1, 79.7, 80.5, 128.0, 128.5, 132.9, 137.0, 200.2. **IR** (EtOH): ν 2991, 2960, 2941, 2221, 1679, 1602, 1524, 1449, 1356, 1227, 1154, 1085, 1009, 851, 777, 693, 656 cm^{-1}. **HRMS** (ESI) m/z: $[M+H]^+$ Calcd. for $C_{16}H_{21}O$ 229.1587; found 229.1590.

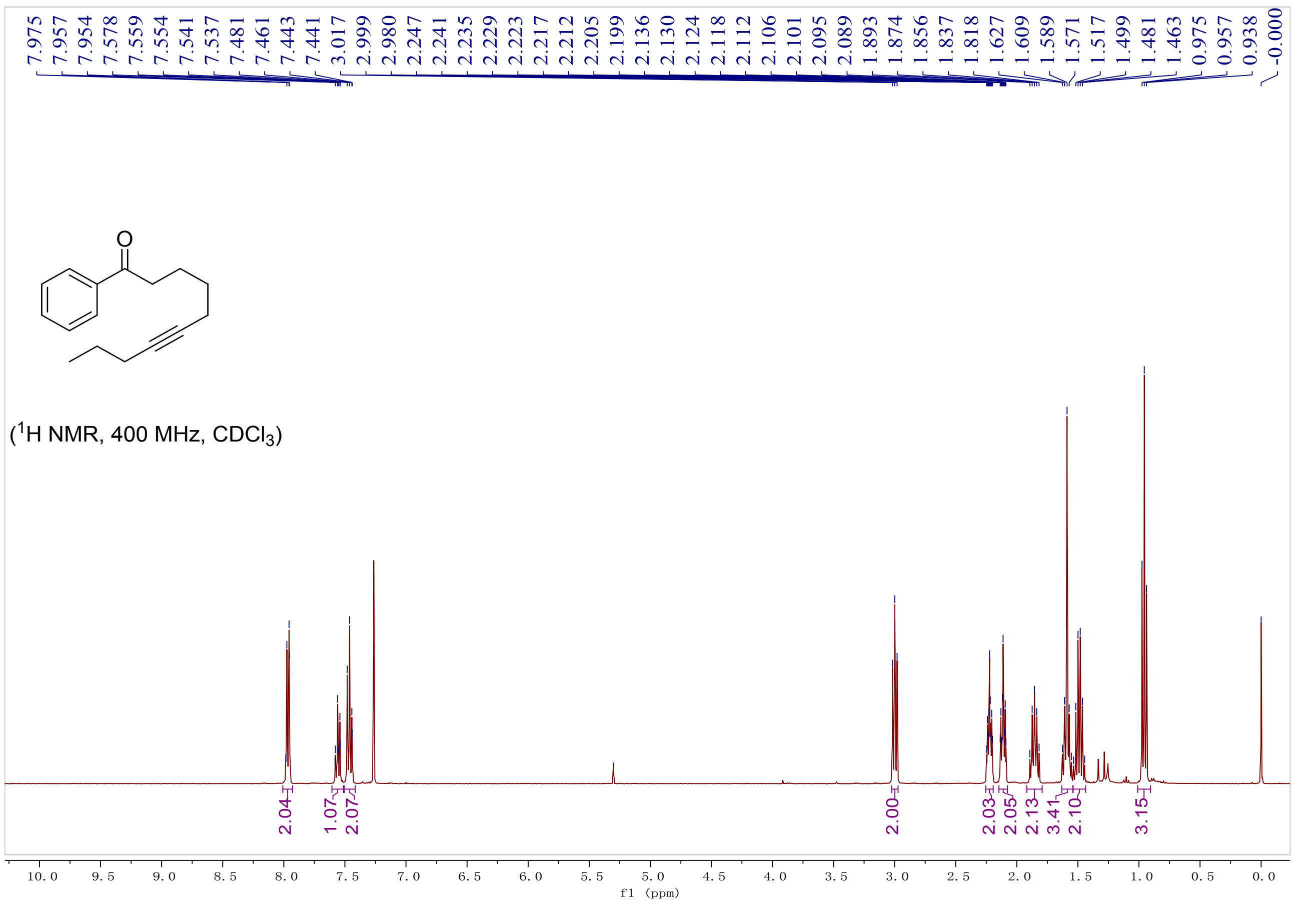

7.975
7.957
7.954
7.578
7.559
7.554
7.541
7.537
7.481
7.461
7.443
7.441
3.017
2.999
2.980
2.247
2.241
2.235
2.229
2.223
2.217
2.212
2.205
2.199
2.136
2.130
2.124
2.118
2.112
2.106
2.101
2.095
2.089
1.893
1.874
1.856
1.837
1.818
1.627
1.609
1.589
1.571
1.517
1.499
1.481
1.463
0.975
0.957
0.938
-0.000
O
(1H NMR, 400 MHz, CDCl3)
2.04
1.07
2.07
2.00
2.03
2.05
2.13
3.41
2.10
3.15
10.0 9.5 9.0 8.5 8.0 7.5 7.0 6.5 6.0 5.5 5.0 4.5 4.0 3.5 3.0 2.5 2.0 1.5 1.0 0.5 0.0
f1 (ppm)

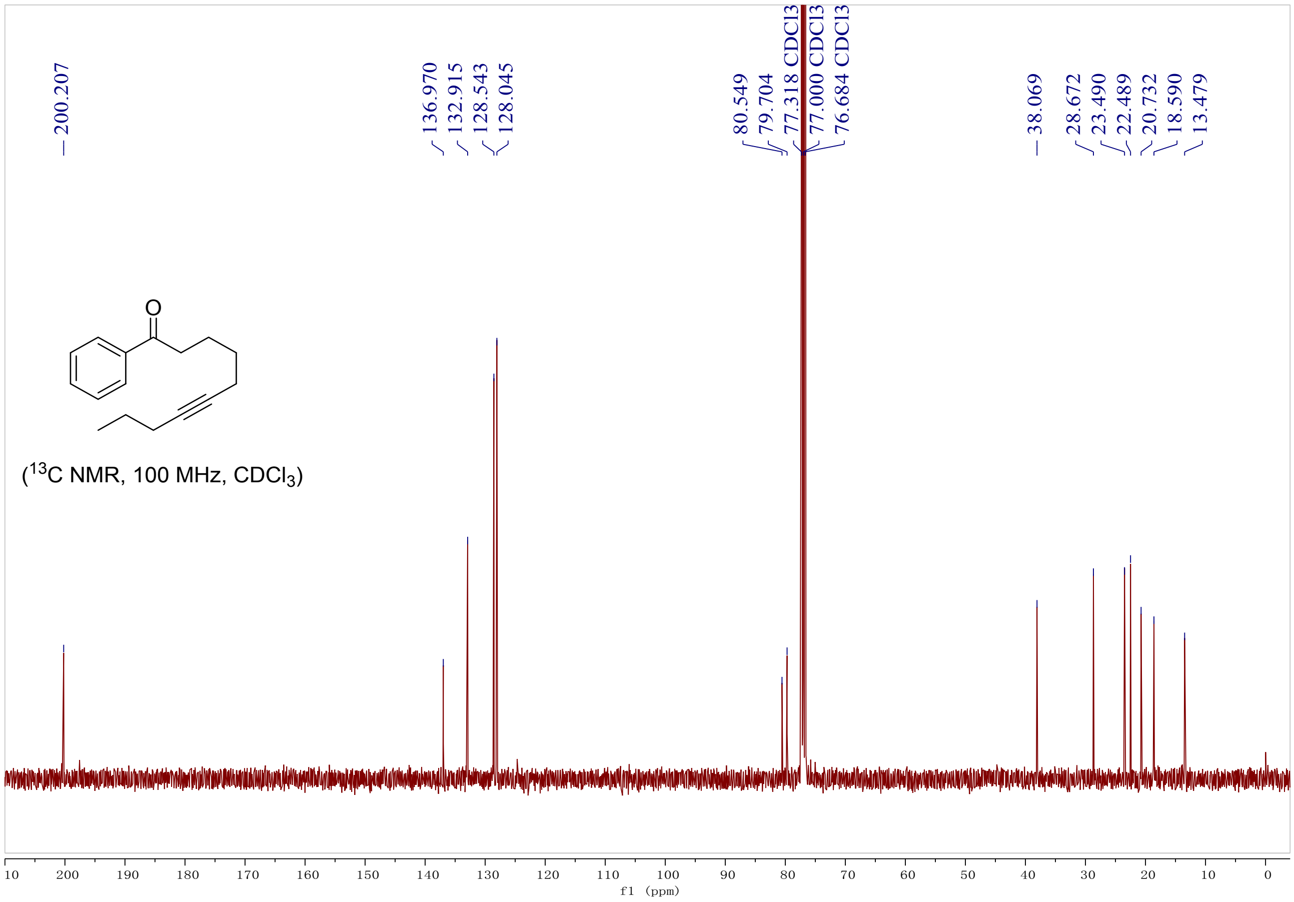

200.207
136.970
132.915
128.543
128.045
80.549
79.704
77.318 CDCl3
77.000 CDCl3
76.684 CDCl3
38.069
28.672
23.490
22.489
20.732
18.590
13.479
O
(13C NMR, 100 MHz, CDCl3)
10 200 190 180 170 160 150 140 130 120 110 100 90 80 70 60 50 40 30 20 10 0
f1 (ppm)

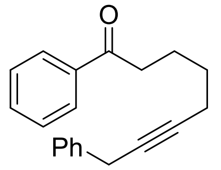

1,8-diphenyloct-6-yn-1-one (3ea): colorless oil, 50 mg, 91%, Eluent: PE/EA = 20/1.

^{1}H NMR (600 MHz, $CDCl_3$, TMS): δ 7.99-7.93 (m, 2H), 7.57-7.53 (m, 1H), 7.48-7.41 (m, 2H), 7.33 (d, *J* = 7.1 Hz, 2H), 7.32-7.28 (m, 2H), 7.23-7.19 (m, 1H), 3.57 (t, *J* = 2.4 Hz, 2H), 3.01 (t, *J* = 7.4 Hz, 2H), 2.29 (tt, *J* = 7.2, 2.4 Hz, 2H), 1.91-1.85 (m, 2H), 1.67-1.60 (m, 2H). **^{13}C NMR** (150 MHz, $CDCl_3$, TMS): δ 18.7, 23.5, 25.1, 28.5, 38.0, 78.0, 82.0, 126.4, 127.8, 128.0, 128.4, 128.6, 132.9, 137.0, 137.5, 200.1. **IR** (EtOH): ν 2973, 2882, 2218, 1659, 1379, 1340, 1267, 1154, 1087, 1045, 861, 823, 732, 693, 680 cm^{-1}. **HRMS** (EI) m/z: $[M]^+$ Calcd. for $C_{20}H_{19}O$ 275.1430; found 275.1424.

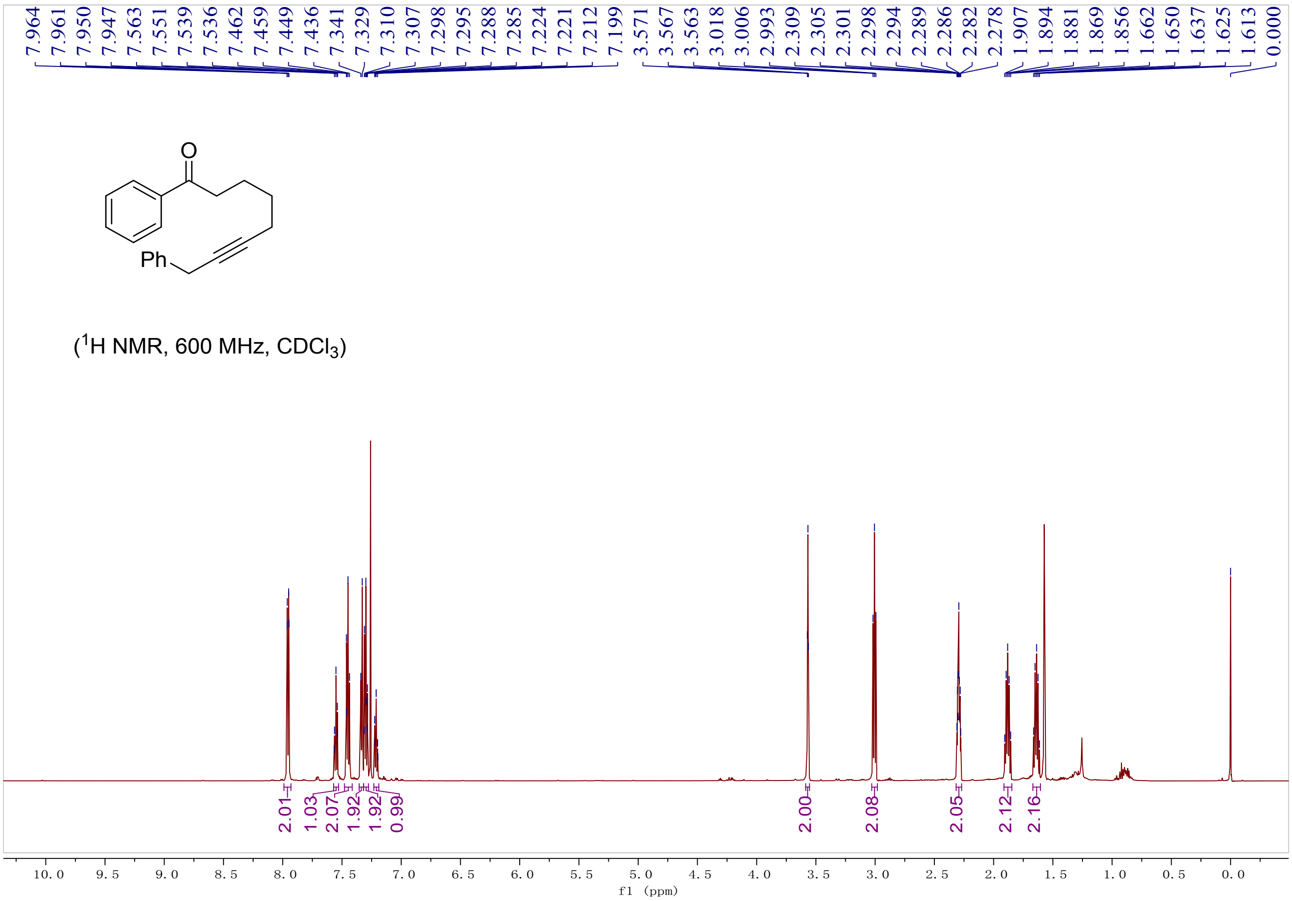
7.964 7.961 7.950 7.947 7.563 7.551 7.539 7.536 7.462 7.459 7.449 7.436 7.341 7.329 7.310 7.307 7.298 7.295 7.288 7.285 7.224 7.221 7.212 7.199 3.571 3.567 3.563 3.018 3.006 2.993 2.309 2.305 2.301 2.298 2.294 2.289 2.286 2.282 2.278 1.907 1.894 1.881 1.869 1.856 1.662 1.650 1.637 1.625 1.613 0.000
O
Ph
(1H NMR, 600 MHz, CDCl3)
2.01 1.03 2.07 1.92 1.92 0.99 2.00 2.08 2.05 2.12 2.16
10.0 9.5 9.0 8.5 8.0 7.5 7.0 6.5 6.0 5.5 5.0 4.5 4.0 3.5 3.0 2.5 2.0 1.5 1.0 0.5 0.0
f1 (ppm)

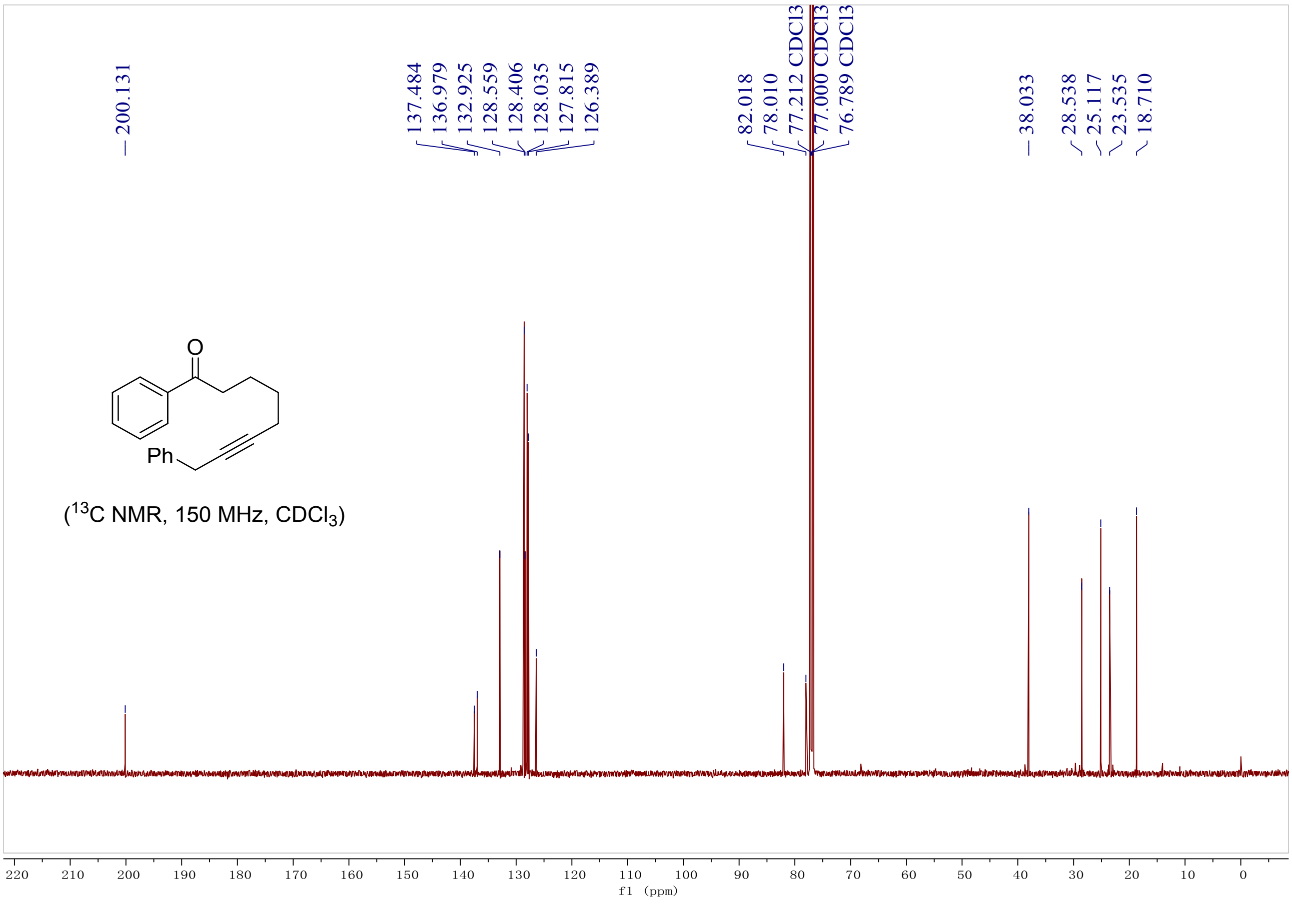
200.131
137.484 136.979 132.925 128.559 128.406 128.035 127.815 126.389
82.018 78.010 77.212 CDCl3 77.000 CDCl3 76.789 CDCl3
38.033 28.538 25.117 23.535 18.710
O
Ph
(13C NMR, 150 MHz, CDCl3)
220 210 200 190 180 170 160 150 140 130 120 110 100 90 80 70 60 50 40 30 20 10 0
f1 (ppm)

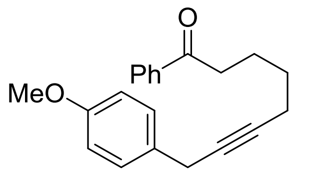

8-(4-methoxyphenyl)-1-phenyloct-6-yn-1-one (3fa): colorless oil, 57 mg, 93%, Eluent: PE/EA = 20/1.

^{1}H NMR (600 MHz, $CDCl_3$, TMS): δ 7.98-7.92 (m, 2H), 7.58-7.52 (m, 1H), 7.48-7.42 (m, 2H), 7.24 (d, J = 8.8 Hz, 2H), 6.84 (d, J = 8.8 Hz, 2H), 3.79 (s, 3H), 3.50 (t, J = 2.4 Hz, 2H), 3.00 (t, J = 7.4 Hz, 2H), 2.28 (tt, J = 7.2, 2.4 Hz, 2H), 1.91-1.84 (m, 2H), 1.66-1.60 (m, 2H). **^{13}C NMR** (150 MHz, $CDCl_3$, TMS): δ 18.7, 23.5, 24.2, 28.5, 38.0, 55.3, 78.5, 81.7, 113.8, 128.0, 128.6, 128.8, 129.6, 132.9, 137.0, 158.2, 200.1. **IR** (EtOH): ν 2972, 2899, 2881, 2219, 1682, 1424, 1399, 1379, 1345, 1327, 1209, 1084, 1002, 861, 729, 693 cm^{-1}. **HRMS** (EI) m/z: $[M]^+$ Calcd. for $C_{21}H_{22}O_2$ 306.1614; found 306.1610.

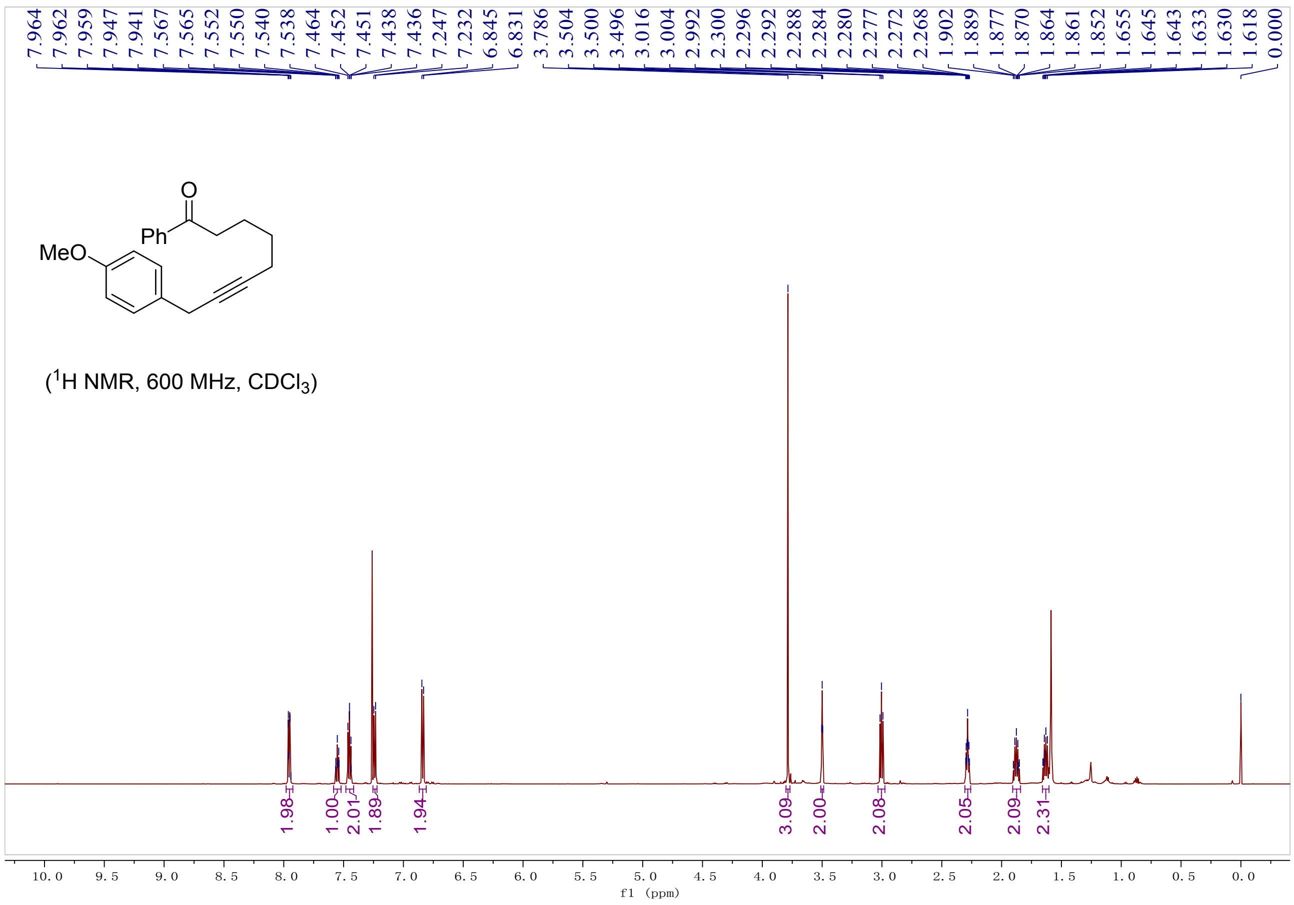
7.964
7.962
7.959
7.947
7.941
7.567
7.565
7.552
7.550
7.540
7.538
7.464
7.452
7.451
7.438
7.436
7.247
7.232
6.845
6.831
3.786
3.504
3.500
3.496
3.016
3.004
2.992
2.300
2.296
2.292
2.288
2.284
2.280
2.277
2.272
2.268
1.902
1.889
1.877
1.870
1.864
1.861
1.852
1.655
1.645
1.643
1.633
1.630
1.618
0.000
Ph
MeO
(1H NMR, 600 MHz, CDCl3)
1.98
1.00
2.01
1.89
1.94
3.09
2.00
2.08
2.05
2.09
2.31
f1 (ppm)

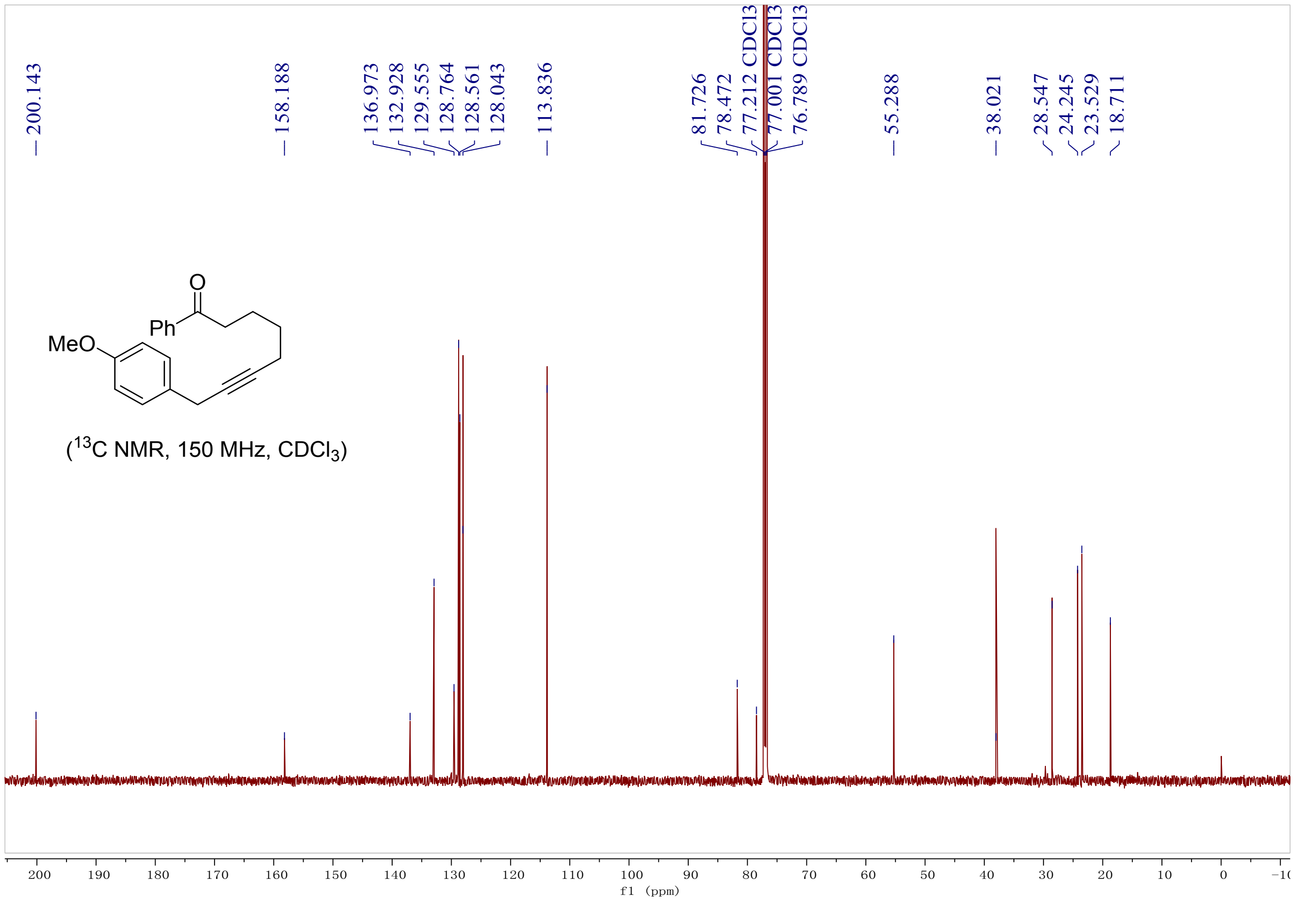
200.143
158.188
136.973
132.928
129.555
128.764
128.561
128.043
113.836
81.726
78.472
77.212 CDCl3
77.001 CDCl3
76.789 CDCl3
55.288
38.021
28.547
24.245
23.529
18.711
Ph
MeO
(13C NMR, 150 MHz, CDCl3)
f1 (ppm)

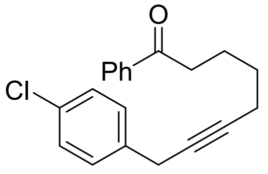

8-(4-chlorophenyl)-1-phenyloct-6-yn-1-one (3ga): colorless oil, 56 mg, 90%, Eluent: PE/EA = 20/1.

^{1}H NMR (400 MHz, $CDCl_3$, TMS): δ 7.99-7.92 (m, 2H), 7.61-7.51 (m, 1H), 7.49-7.42 (m, 2H), 7.26 (s, 4H), 3.53 (t, J = 2.4 Hz, 2H), 3.01 (t, J = 7.2 Hz, 2H), 2.29 (tt, J = 7.0, 2.4 Hz, 2H), 1.92-1.83 (m, 2H), 1.67-1.58 (m, 2H). **^{13}C NMR** (150 MHz, $CDCl_3$, TMS): δ 18.7, 23.5, 24.6, 28.5, 38.0, 77.5, 82.5, 128.0, 128.5, 128.6, 129.2, 132.1, 133.0, 136.0, 137.0, 200.0. **IR** (EtOH): ν 2973, 2883, 2870, 2217, 1654, 1424, 1380, 1328, 1087, 1045, 880, 737, 634 cm^{-1}. **HRMS** (EI) m/z: $[M]^+$ Calcd. for $C_{20}H_{18}OCl$ 309.1041; found 309.1036.

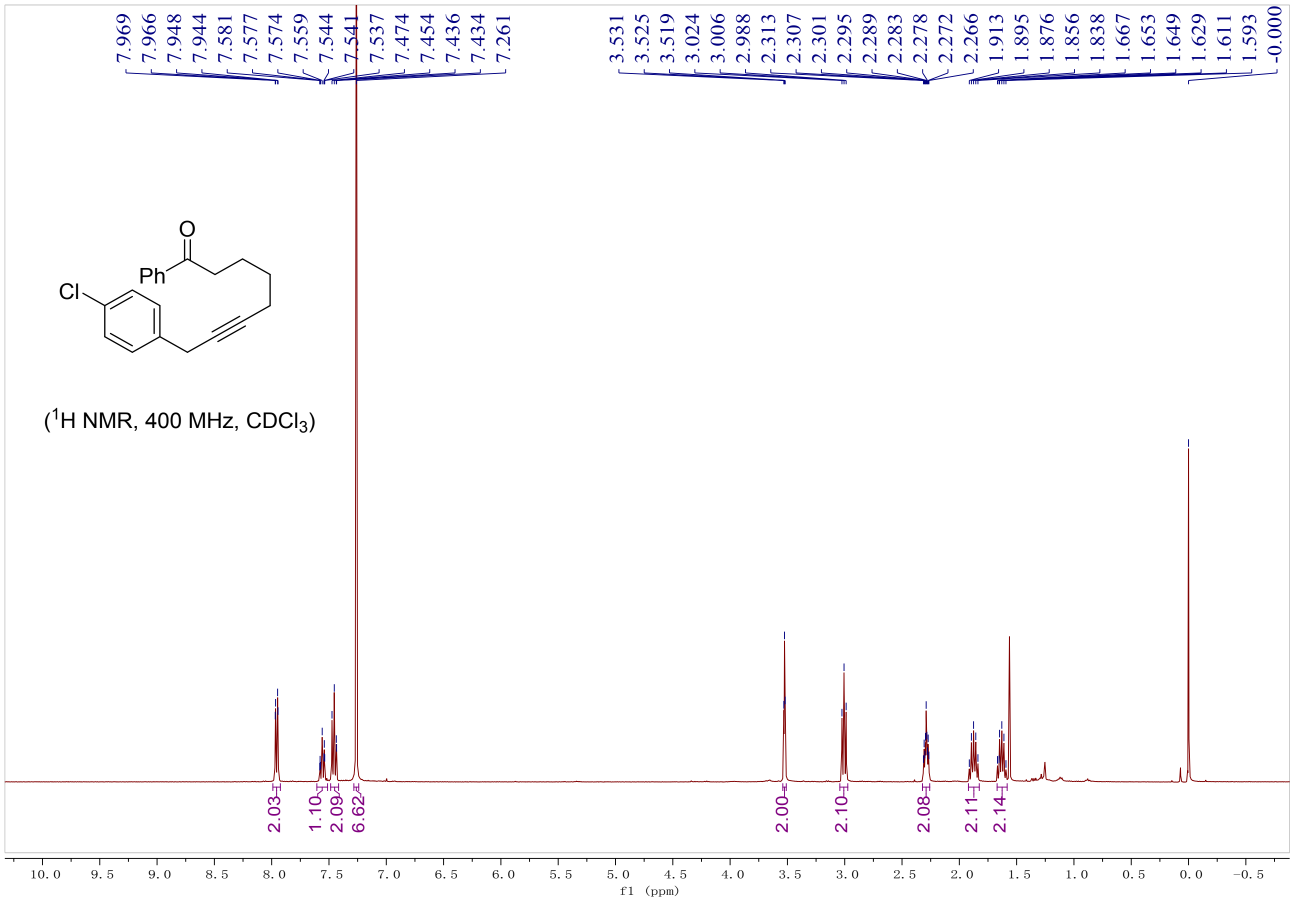
7.969
7.966
7.948
7.944
7.581
7.577
7.574
7.559
7.544
7.541
7.537
7.474
7.454
7.436
7.434
7.261
3.531
3.525
3.519
3.024
3.006
2.988
2.313
2.307
2.301
2.295
2.289
2.283
2.278
2.272
2.266
1.913
1.895
1.876
1.856
1.838
1.667
1.653
1.649
1.629
1.611
1.593
-0.000
O
Ph
Cl
(¹H NMR, 400 MHz, CDCl3)
2.03
1.10
2.09
6.62
2.00
2.10
2.08
2.11
2.14
10.0
9.5
9.0
8.5
8.0
7.5
7.0
6.5
6.0
5.5
5.0
4.5
4.0
3.5
3.0
2.5
2.0
1.5
1.0
0.5
0.0
-0.5
f1 (ppm)

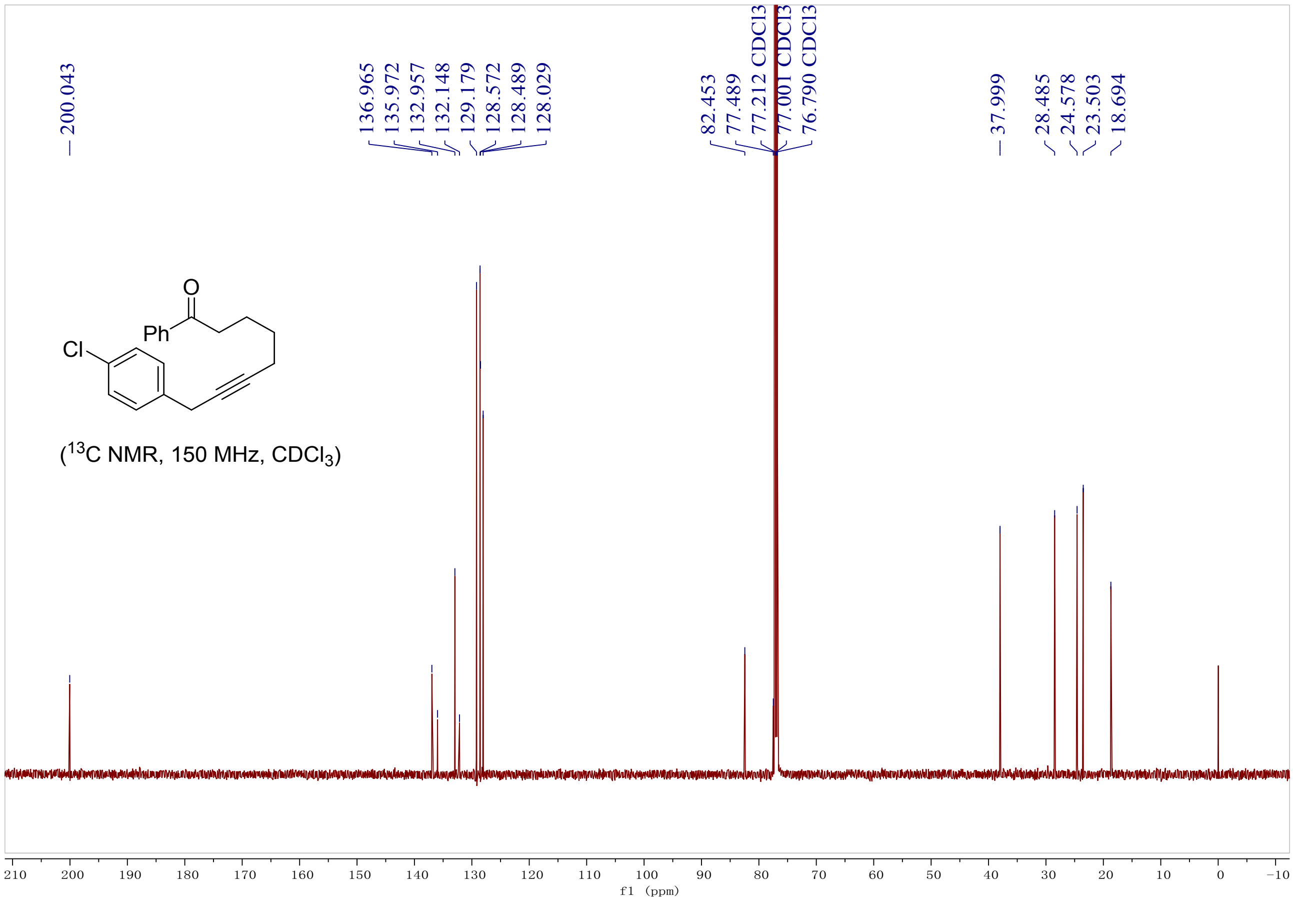
200.043
136.965
135.972
132.957
132.148
129.179
128.572
128.489
128.029
82.453
77.489
77.212 CDCl3
77.001 CDCl3
76.790 CDCl3
37.999
28.485
24.578
23.503
18.694
O
Ph
Cl
(¹³C NMR, 150 MHz, CDCl3)
210
200
190
180
170
160
150
140
130
120
110
100
90
80
70
60
50
40
30
20
10
0
-10
f1 (ppm)

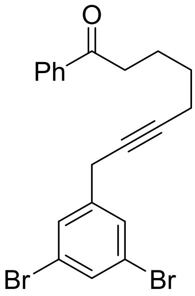

8-(3,5-dibromophenyl)-1-phenyloct-6-yn-1-one (3ha): colorless oil, 77 mg, 89%, Eluent: PE/EA = 20/1.

^{1}H NMR (600 MHz, $CDCl_3$, TMS): δ 7.98-7.93 (m, 2H), 7.57-7.54 (m, 1H), 7.52 (t, *J* = 2.0 Hz, 1H), 7.47-7.44 (m, 2H), 7.42 (dd, *J* = 1.8, 0.9 Hz, 2H), 3.51 (t, *J* = 2.6 Hz, 2H), 3.02 (t, *J* = 7.2 Hz, 2H), 2.30 (tt, *J* = 7.0, 2.4 Hz, 2H), 1.88 (tt, *J* = 7.6, 6.4 Hz, 2H), 1.68-1.61 (m, 2H). **^{13}C NMR** (150 MHz, $CDCl_3$, TMS): δ 18.7, 22.7, 24.6, 28.4, 38.0, 76.2, 83.5, 122.8, 128.0, 128.6, 129.8, 132.2, 133.0, 137.0, 141.4, 200.0. **IR** (EtOH): ν 3026, 2942, 2862, 2115, 1682, 1597, 1448, 1367, 1223, 1180, 1002, 988, 880 cm^{-1}. **HRMS** (ESI) m/z: $[M+Na]^+$ Calcd. for $C_{20}H_{18}OBr_2Na$ 456.9596; found 456.9605.

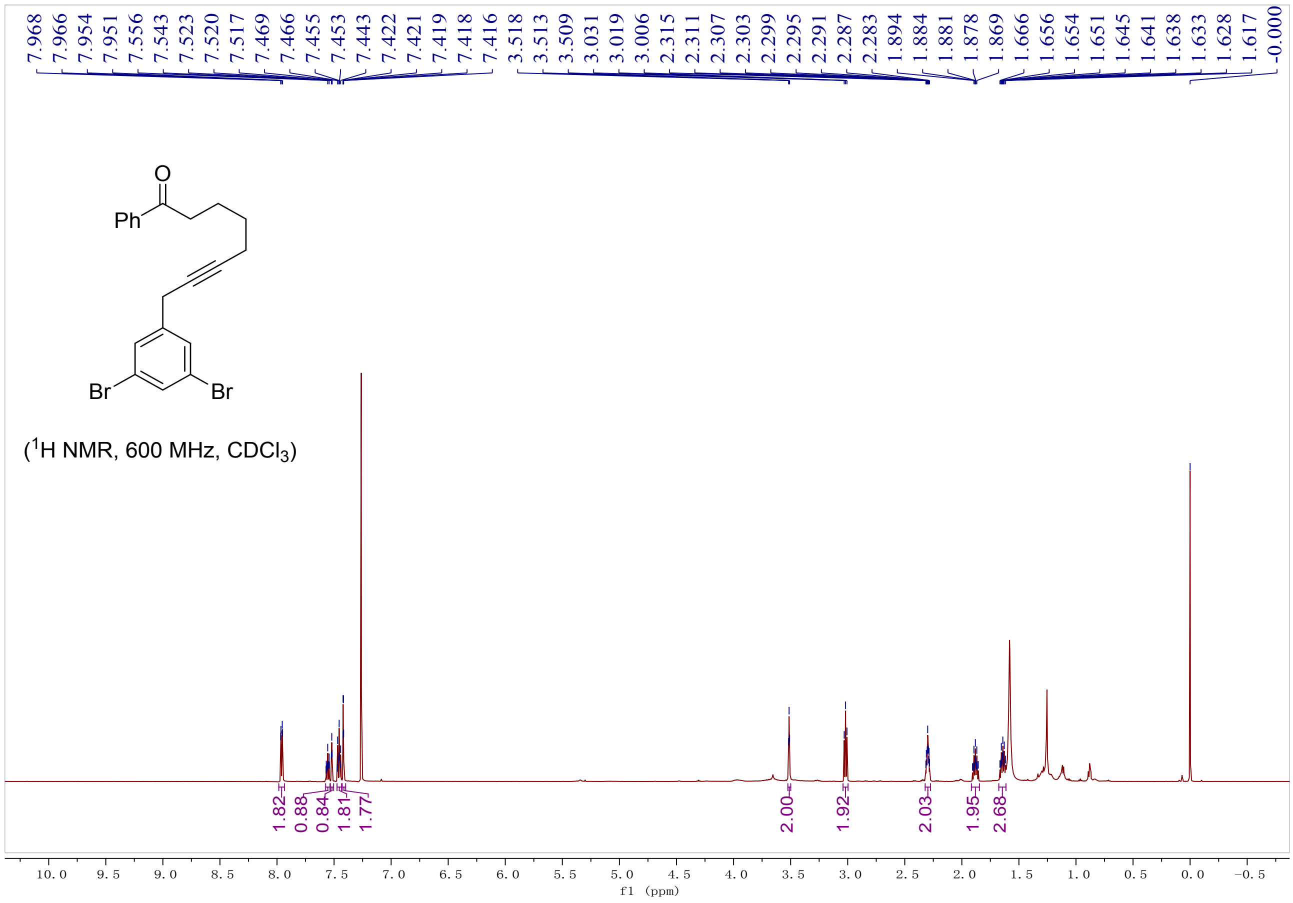

7.968 7.966 7.954 7.951 7.556 7.543 7.523 7.520 7.517 7.469 7.466 7.455 7.453 7.443 7.422 7.421 7.419 7.418 7.416 3.518 3.513 3.509 3.031 3.019 3.006 2.315 2.311 2.307 2.303 2.299 2.295 2.291 2.287 2.283 1.894 1.884 1.881 1.878 1.869 1.666 1.656 1.654 1.651 1.645 1.641 1.638 1.633 1.628 1.617 -0.000
O
Ph
Br
Br
(1H NMR, 600 MHz, CDCl3)
1.82 0.88 0.84 1.81 1.77 2.00 1.92 2.03 1.95 2.68
10.0 9.5 9.0 8.5 8.0 7.5 7.0 6.5 6.0 5.5 5.0 4.5 4.0 3.5 3.0 2.5 2.0 1.5 1.0 0.5 0.0 -0.5
f1 (ppm)

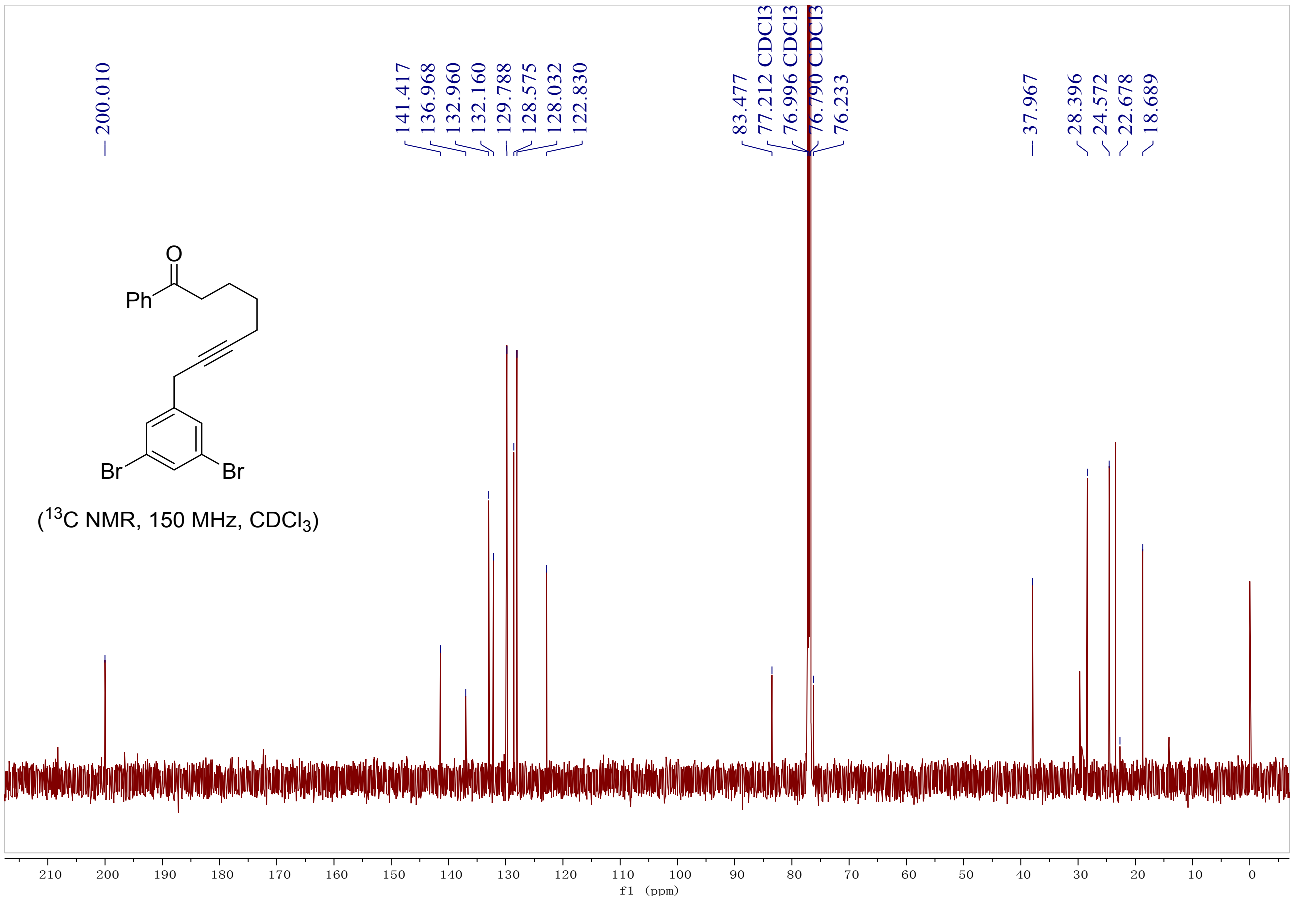

200.010
141.417 136.968 132.960 132.160 129.788 128.575 128.032 122.830
83.477 77.212 CDCl3 76.996 CDCl3 76.790 CDCl3 76.233
37.967
28.396 24.572 22.678 18.689
O
Ph
Br
Br
(13C NMR, 150 MHz, CDCl3)
210 200 190 180 170 160 150 140 130 120 110 100 90 80 70 60 50 40 30 20 10 0
f1 (ppm)

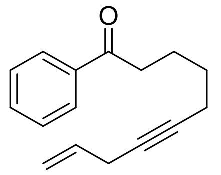

1-phenyldec-9-en-6-yn-1-one (3ia): colorless oil, 36 mg, 80%, Eluent: PE/EA = 20/1.

^{1}H NMR (400 MHz, $CDCl_3$, TMS): δ 8.00-7.93 (m, 2H), 7.59-7.53 (m, 1H), 7.49-7.42 (m, 2H), 5.81 (ddt, *J* = 15.6, 10.2, 5.2 Hz, 1H), 5.30 (dt, *J* = 17.0, 2.0 Hz, 1H), 5.08 (dd, *J* = 10.0, 2.0 Hz, 1H), 3.04-2.88 (m, 4H), 2.26 (t, *J* = 7.0 Hz, 2H), 1.87 (p, *J* = 7.6 Hz, 2H), 1.68-1.56 (m, 2H). **^{13}C NMR** (150 MHz, $CDCl_3$, TMS): δ 18.6, 23.1, 23.5, 28.6, 38.0, 77.0, 82.2, 115.6, 128.0, 128.6, 132.9, 133.3, 137.0, 200.1. **IR** (EtOH): ν 2973, 2918, 2890, 2128, 1920, 1655, 1450, 1379, 1323, 1087, 1045, 880, 799, 702, 689 cm^{-1}. **HRMS** (ESI) m/z: $[M+H]^+$ Calcd. for $C_{16}H_{19}O$ 227.1430; found 227.1431.

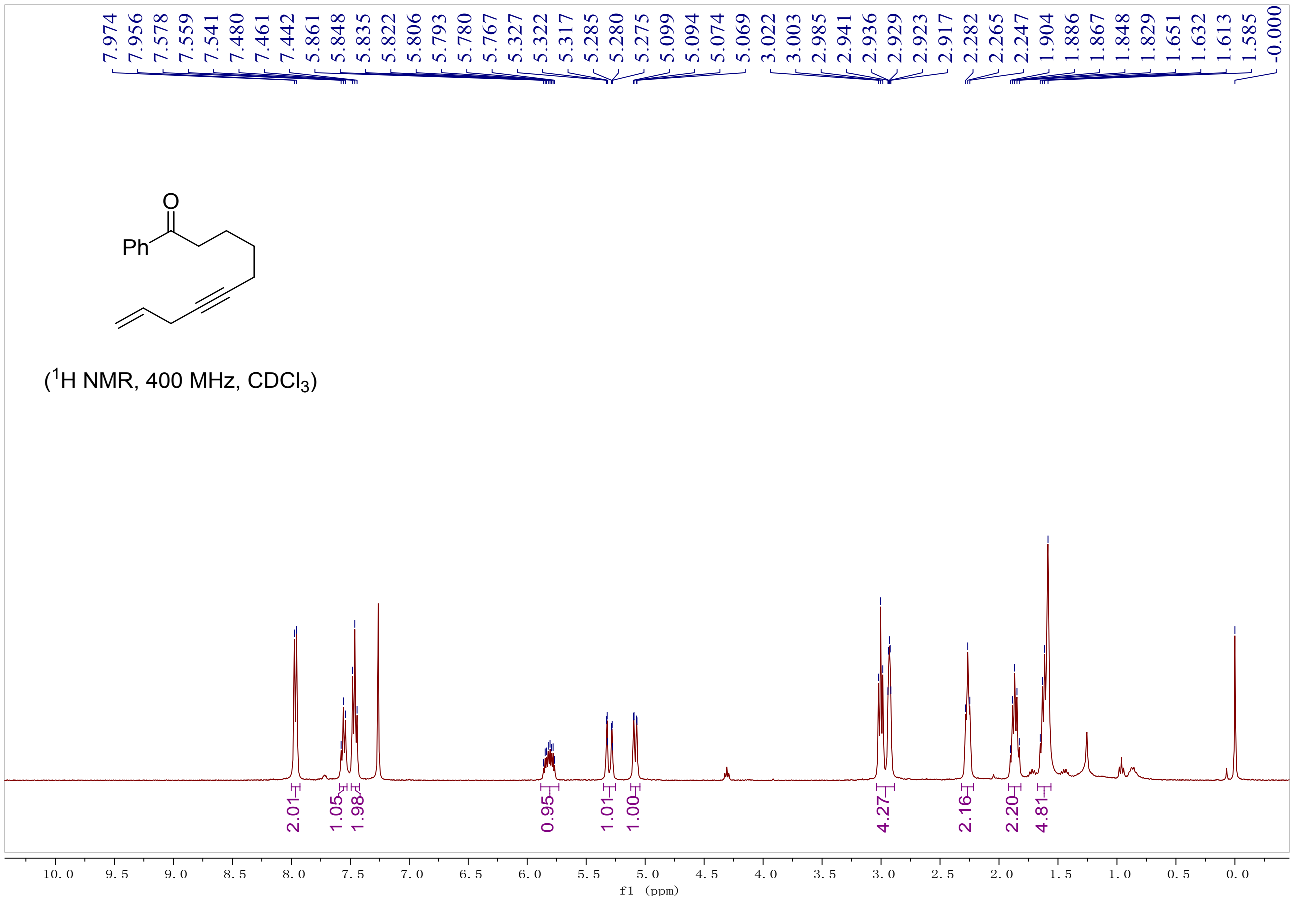
7.974
7.956
7.578
7.559
7.541
7.480
7.461
7.442
5.861
5.848
5.835
5.822
5.806
5.793
5.780
5.767
5.327
5.322
5.317
5.285
5.280
5.275
5.099
5.094
5.074
5.069
3.022
3.003
2.985
2.941
2.936
2.929
2.923
2.917
2.282
2.265
2.247
1.904
1.886
1.867
1.848
1.829
1.651
1.632
1.613
1.585
-0.000
O
Ph
(¹H NMR, 400 MHz, CDCl3)
2.01
1.05
1.98
0.95
1.01
1.00
4.27
2.16
2.20
4.81
f1 (ppm)

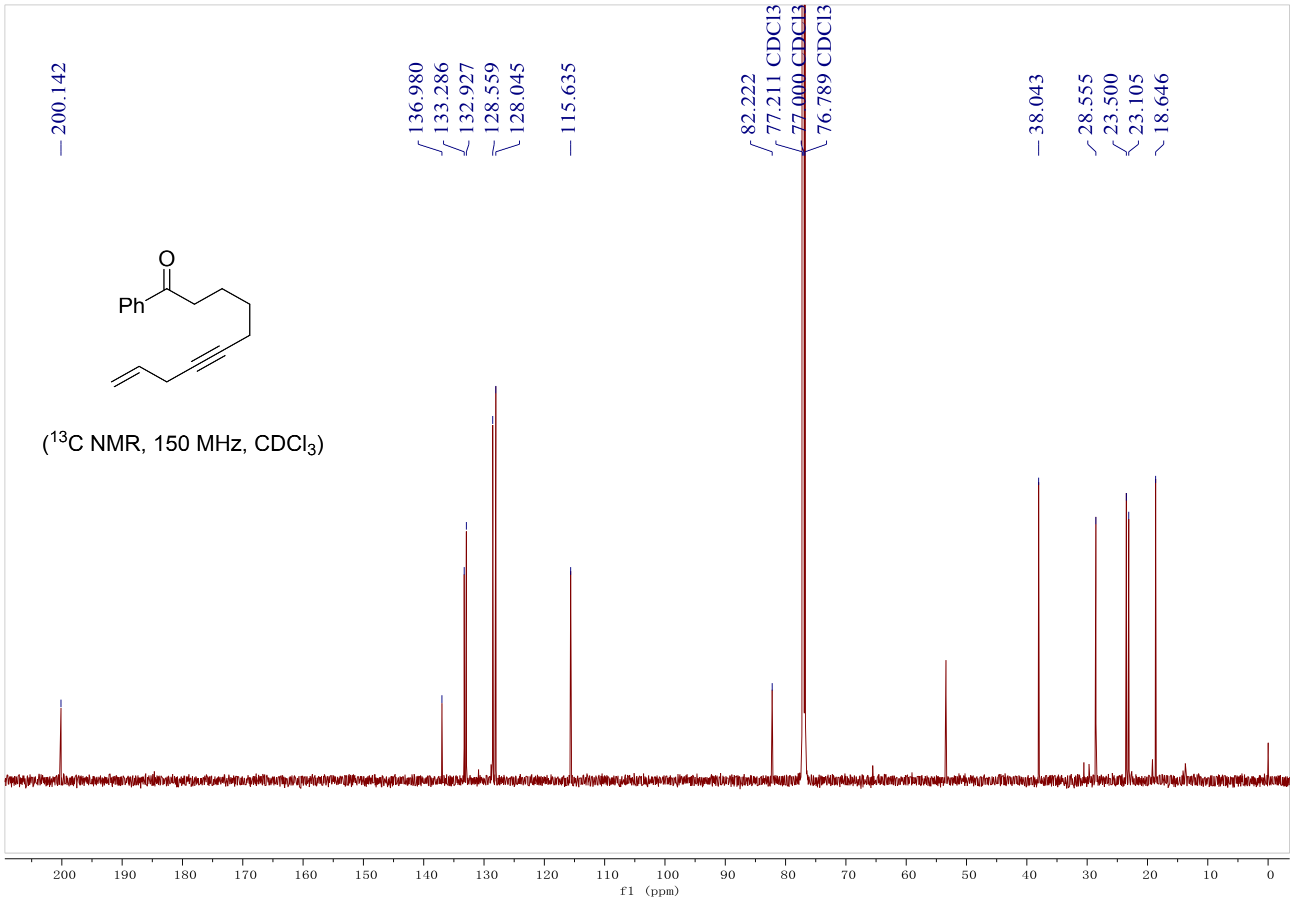
200.142
136.980
133.286
132.927
128.559
128.045
115.635
82.222
77.211 CDCl3
77.000 CDCl3
76.789 CDCl3
38.043
28.555
23.500
23.105
18.646
O
Ph
(13C NMR, 150 MHz, CDCl3)
f1 (ppm)

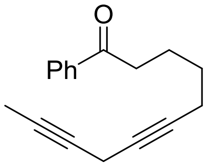

1-phenylundeca-6,9-diyn-1-one (3ja): colorless oil, 39 mg, 82%, Eluent: PE/EA = 20/1.

^{1}H NMR (400 MHz, $CDCl_3$, TMS): δ 7.98-7.93 (m, 2H), 7.59-7.53 (m, 1H), 7.49-7.43 (m, 2H), 3.11-3.05 (m, 2H), 2.99 (t, J = 7.4 Hz, 2H), 2.23 (tt, J = 7.2, 2.4 Hz, 2H), 1.89-1.80 (m, 2H), 1.78 (t, J = 2.4 Hz, 3H), 1.64-1.55 (m, 2H). **^{13}C NMR** (100 MHz, $CDCl_3$, TMS): δ 3.5, 9.6, 18.6, 23.4, 28.2, 38.0, 73.5, 74.8, 75.9, 79.9, 128.0, 128.5, 132.9, 136.9, 200.1. **IR** (EtOH): ν 2972, 2925, 2870, 2201, 1678, 1597, 1449, 1376, 1223, 1085, 1045, 879, 751, 691 cm^{-1}. **HRMS** (EI) m/z: $[M]^+$ Calcd. for $C_{17}H_{17}O$ 237.1274; found 237.1266.

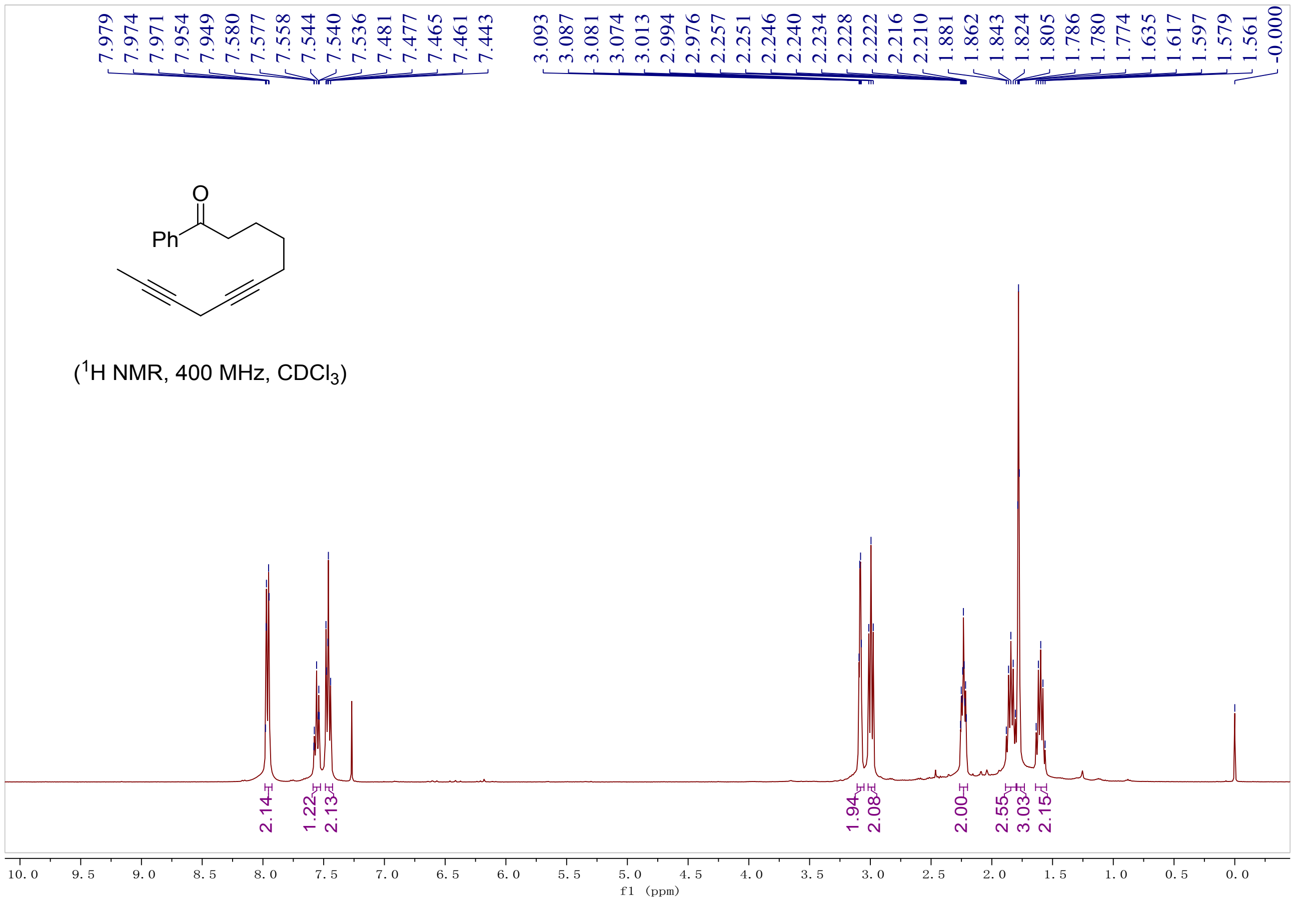
7.979 7.974 7.971 7.954 7.949 7.580 7.577 7.558 7.544 7.540 7.536 7.481 7.477 7.465 7.461 7.443
3.093 3.087 3.081 3.074 3.013 2.994 2.976 2.257 2.251 2.246 2.240 2.234 2.228 2.222 2.216 2.210 1.881 1.862 1.843 1.824 1.805 1.786 1.780 1.774 1.635 1.617 1.597 1.579 1.561 -0.000
O
Ph
(1H NMR, 400 MHz, CDCl3)
2.14 1.22 2.13 1.94 2.08 2.00 2.55 3.03 2.15
f1 (ppm)

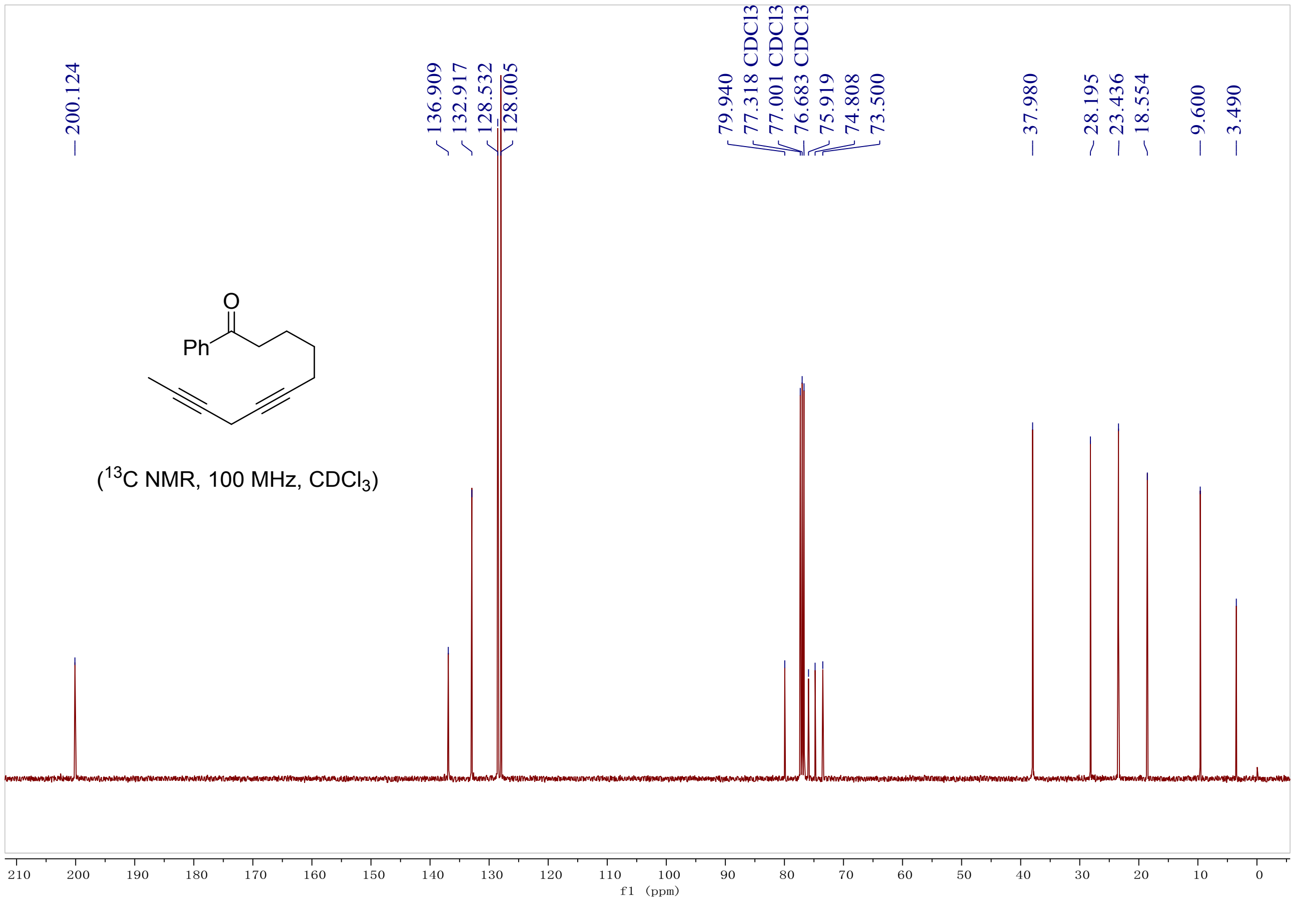
200.124
136.909 132.917 128.532 128.005
79.940 77.318 CDCl3 77.001 CDCl3 76.683 CDCl3 75.919 74.808 73.500
37.980 28.195 23.436 18.554 9.600 3.490
O
Ph
(13C NMR, 100 MHz, CDCl3)
f1 (ppm)

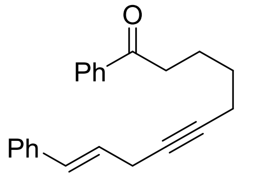

(*E*)-1,9-diphenylnon-8-en-6-yn-1-one (3ka): colorless oil, 54 mg, 94%, Eluent: PE/EA = 20/1.

^{1}H NMR (400 MHz, $CDCl_3$, TMS): δ 7.99-7.94 (m, 2H), 7.58-7.52 (m, 1H), 7.47-7.42 (m, 2H), 7.37-7.33 (m, 2H), 7.32-7.27 (m, 2H), 7.23-7.18 (m, 1H), 6.62 (dt, *J* = 15.8, 2.0 Hz, 1H), 6.17 (dt, *J* = 15.8, 5.6 Hz, 1H), 3.10 (dq, *J* = 4.4, 2.2 Hz, 2H), 3.02 (t, *J* = 7.2 Hz, 2H), 2.30 (tt, *J* = 7.2, 2.4 Hz, 2H), 1.94-1.85 (m, 2H), 1.69-1.60 (m, 2H). **^{13}C NMR** (150 MHz, $CDCl_3$, TMS): δ 18.7, 22.4, 23.5, 28.5, 38.0, 82.3, 125.2, 126.2, 127.2, 128.0, 128.5, 128.6, 130.9, 132.9, 137.0, 137.2, 200.1. **IR** (EtOH): ν 2924, 2890, 2199, 1681, 1595, 1449, 1368, 1284, 1221, 1004, 965, 749, 693 cm^{-1}. **HRMS** (ESI) m/z: $[M+H]^+$ Calcd. for $C_{21}H_{21}O$ 289.1587; found 289.1589.

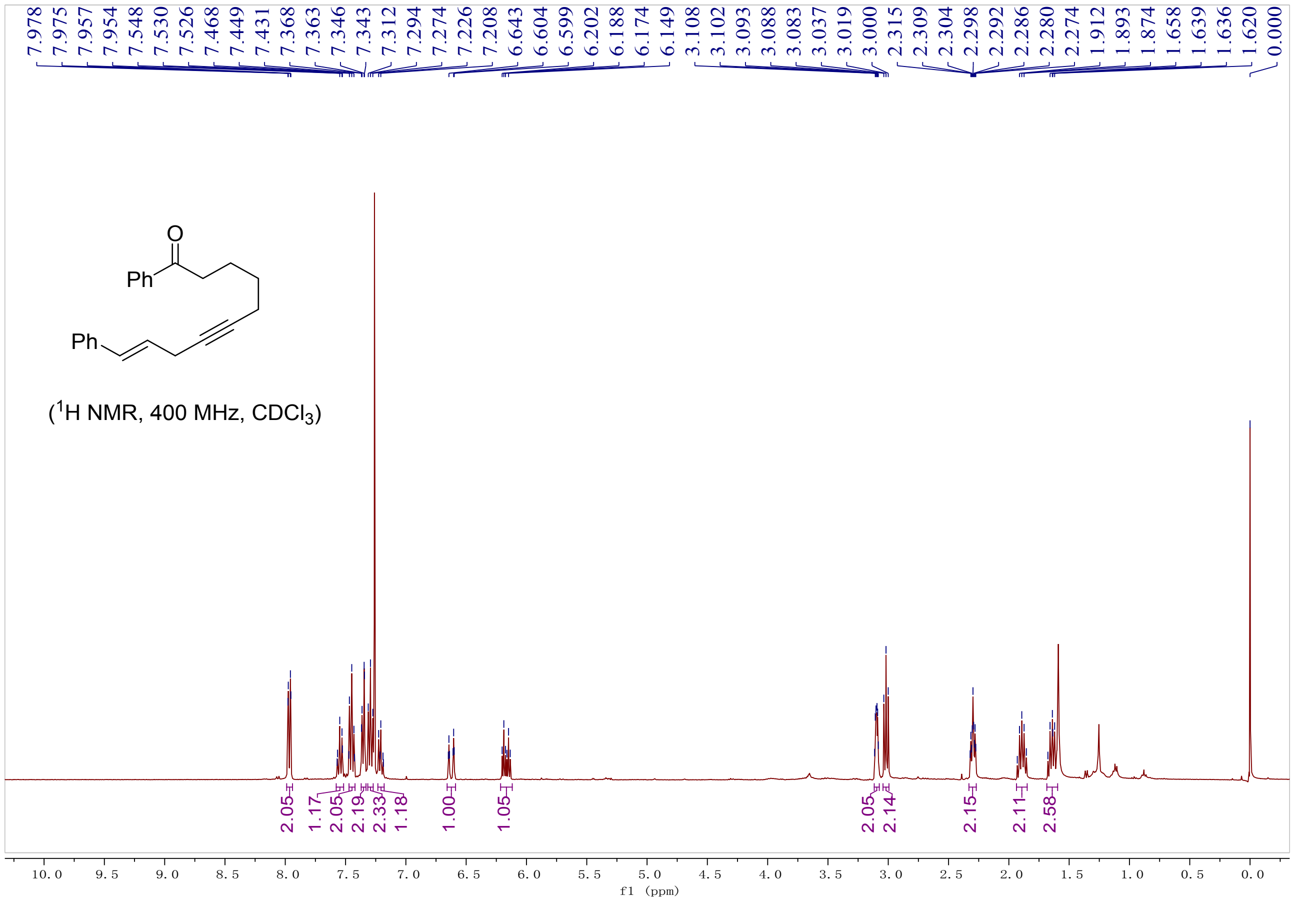
7.978
7.975
7.957
7.954
7.548
7.530
7.526
7.468
7.449
7.431
7.368
7.363
7.346
7.343
7.312
7.294
7.274
7.226
7.208
6.643
6.604
6.599
6.202
6.188
6.174
6.149
3.108
3.102
3.093
3.088
3.083
3.037
3.019
3.000
2.315
2.309
2.304
2.298
2.292
2.286
2.280
2.274
1.912
1.893
1.874
1.658
1.639
1.636
1.620
0.000
O
Ph
Ph
(1H NMR, 400 MHz, CDCl3)
2.05
1.17
2.05
2.19
2.33
1.18
1.00
1.05
2.05
2.14
2.15
2.11
2.58
10.0 9.5 9.0 8.5 8.0 7.5 7.0 6.5 6.0 5.5 5.0 4.5 4.0 3.5 3.0 2.5 2.0 1.5 1.0 0.5 0.0
f1 (ppm)

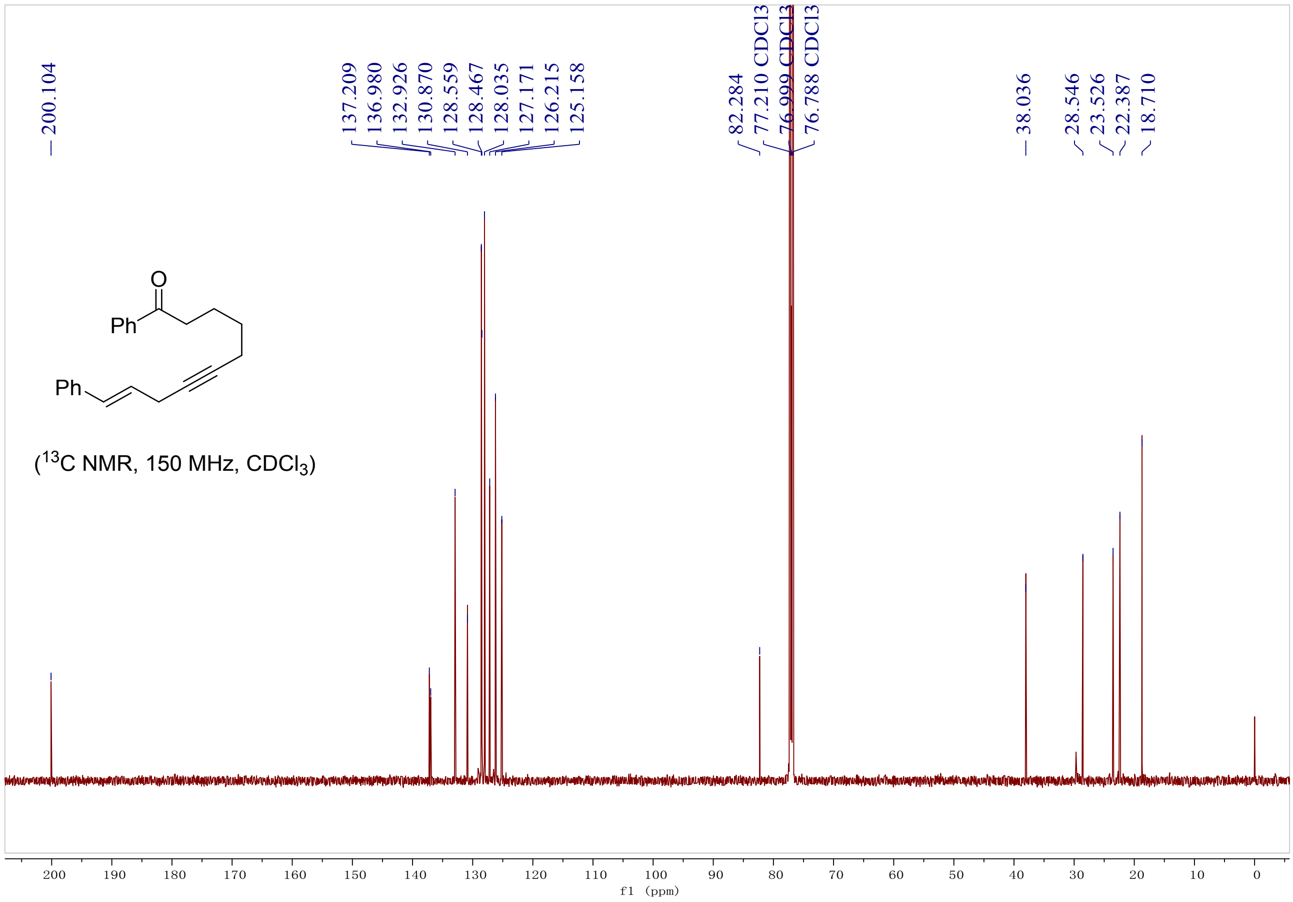
200.104
137.209
136.980
132.926
130.870
128.559
128.467
128.035
127.171
126.215
125.158
82.284
77.210 CDCl3
76.999 CDCl3
76.788 CDCl3
38.036
28.546
23.526
22.387
18.710
O
Ph
Ph
(13C NMR, 150 MHz, CDCl3)
200 190 180 170 160 150 140 130 120 110 100 90 80 70 60 50 40 30 20 10 0
f1 (ppm)

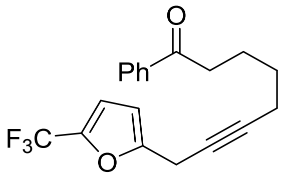

1-phenyl-8-(5-(trifluoromethyl)furan-2-yl)oct-6-yn-1-one (3la): colorless oil, 57 mg, 85%, Eluent: PE/EA = 20/1.

^{1}H NMR (600 MHz, $CDCl_3$, TMS): δ 7.98-7.93 (m, 2H), 7.59-7.54 (m, 1H), 7.48-7.43 (m, 2H), 6.70 (dd, *J* = 3.2, 1.2 Hz, 1H), 6.28 (dd, *J* = 3.2, 1.2 Hz, 1H), 3.57 (s, 2H), 3.01 (t, *J* = 7.2 Hz, 2H), 2.27 (tt, *J* = 7.0, 2.4 Hz, 2H), 1.87 (tt, *J* = 7.6, 6.4 Hz, 2H), 1.65-1.60 (m, 2H). **^{13}C NMR** (150 MHz, $CDCl_3$, TMS): δ 18.6, 18.9, 23.4, 28.3, 37.9, 73.8, 82.4, 107.1, 112.5, 119.1 (q, *J* = 220.6 Hz), 128.0, 128.6, 133.0, 137.0, 140.8 (q, *J* = 35.6 Hz), 154.5, 200.0. ^{19}F NMR (564 MHz, $CDCl_3$): δ -64.0; **IR** (EtOH): ν 2975, 2925, 2895, 2201, 1651, 1457, 1382, 1327, 1086, 1044, 879, 788, 632 cm^{-1}. **HRMS** (EI) m/z: $[M]^+$ Calcd. for $C_{19}H_{17}O_2F_3$ 334.1175; found 334.1165.

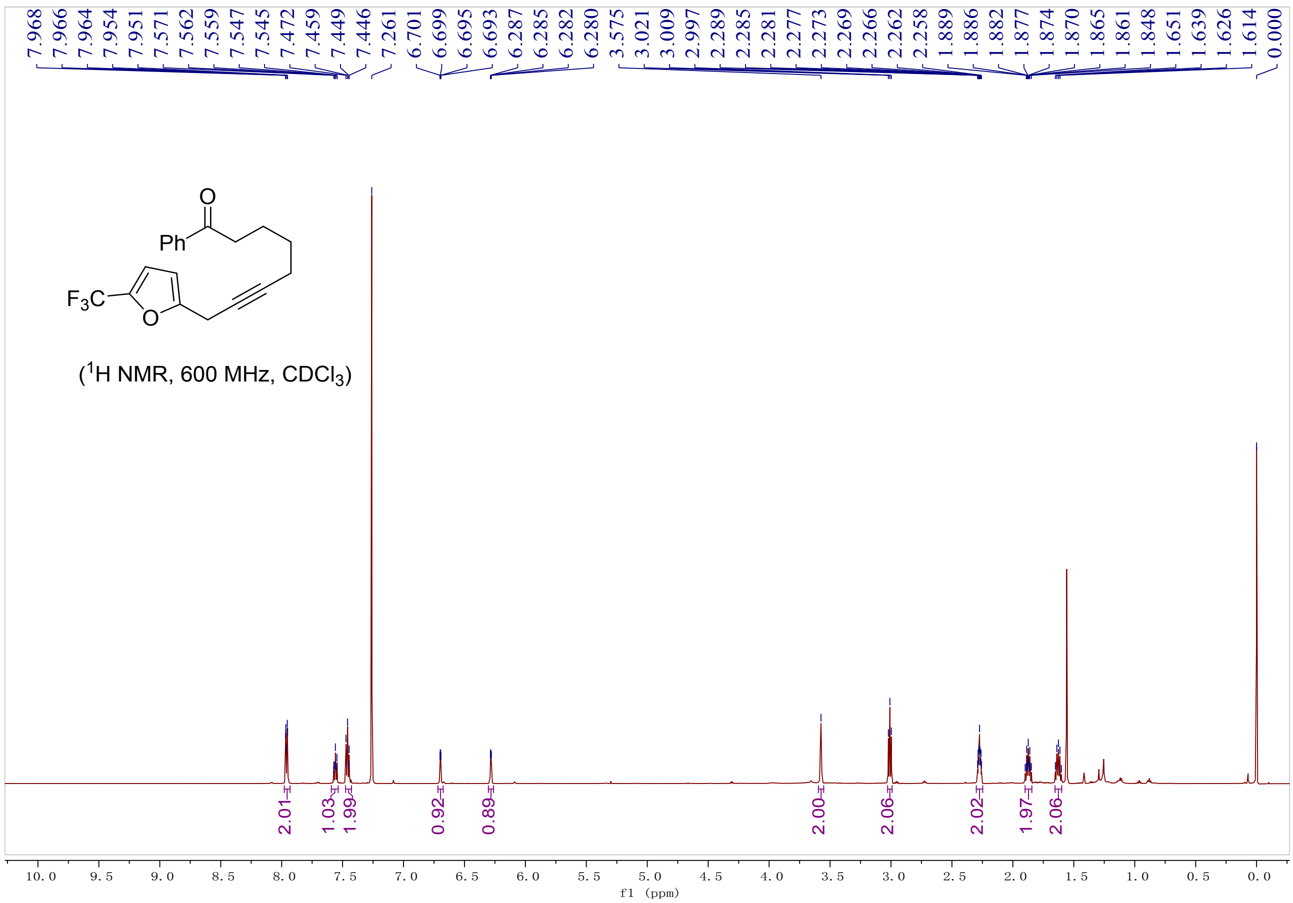
7.968 7.966 7.964 7.954 7.951 7.571 7.562 7.559 7.547 7.545 7.472 7.459 7.449 7.446 7.261 6.701 6.699 6.695 6.693 6.287 6.285 6.282 6.280 3.575 3.021 3.009 2.997 2.289 2.285 2.281 2.277 2.273 2.269 2.266 2.262 2.258 1.889 1.886 1.882 1.877 1.874 1.870 1.865 1.861 1.848 1.651 1.639 1.626 1.614 0.000
O
Ph
F_3C
O
(^{1}H NMR, 600 MHz, $CDCl_3$)
2.01 1.03 1.99 0.92 0.89 2.00 2.06 2.02 1.97 2.06
10.0 9.5 9.0 8.5 8.0 7.5 7.0 6.5 6.0 5.5 5.0 4.5 4.0 3.5 3.0 2.5 2.0 1.5 1.0 0.5 0.0
f1 (ppm)

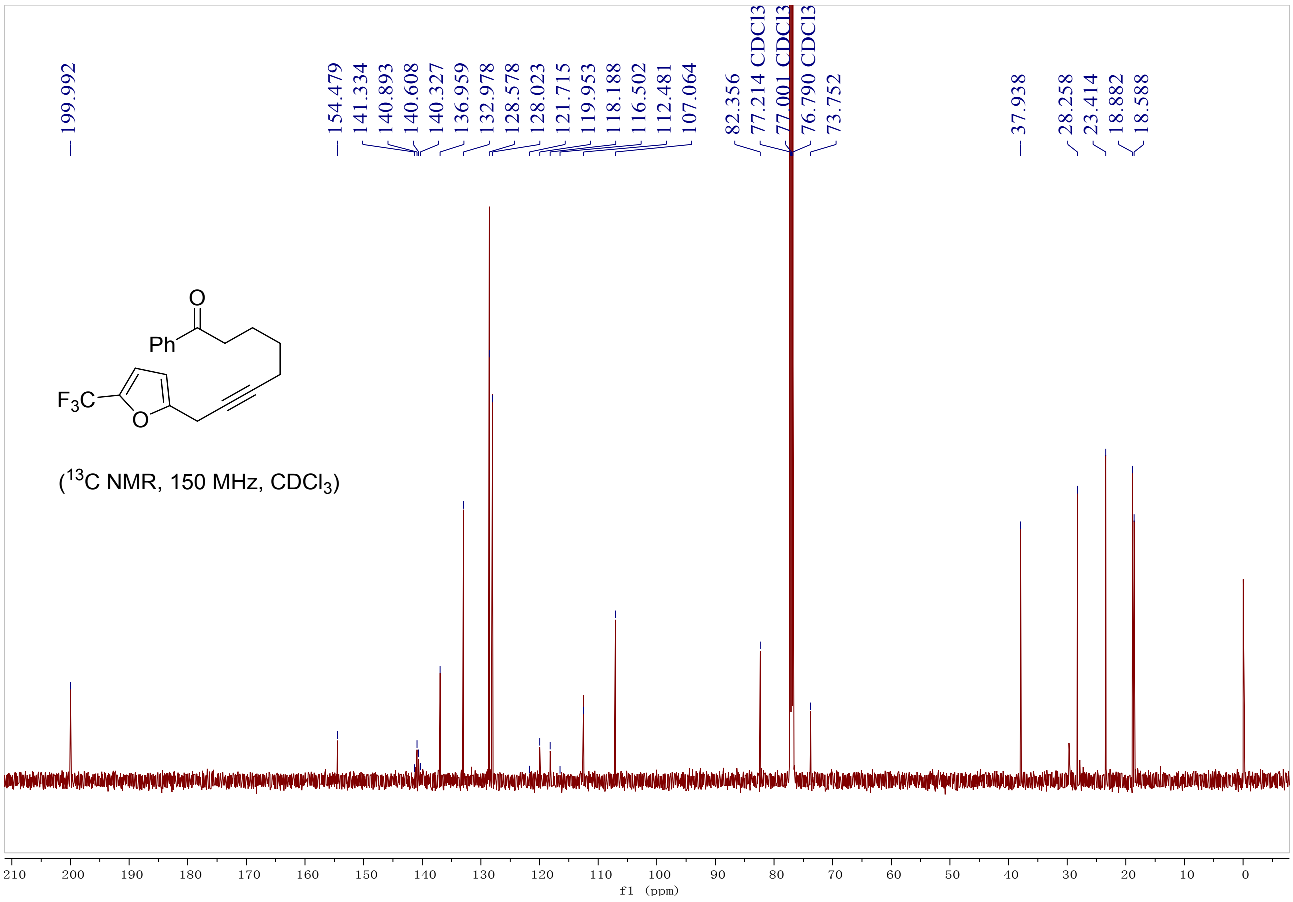
199.992
154.479 141.334 140.893 140.608 140.327 136.959 132.978 128.578 128.023 121.715 119.953 118.188 116.502 112.481 107.064
82.356 77.214 CDCl3 77.001 CDCl3 76.790 CDCl3 73.752
37.938 28.258 23.414 18.882 18.588
O
Ph
F_3C
O
(^{13}C NMR, 150 MHz, $CDCl_3$)
210 200 190 180 170 160 150 140 130 120 110 100 90 80 70 60 50 40 30 20 10 0
f1 (ppm)

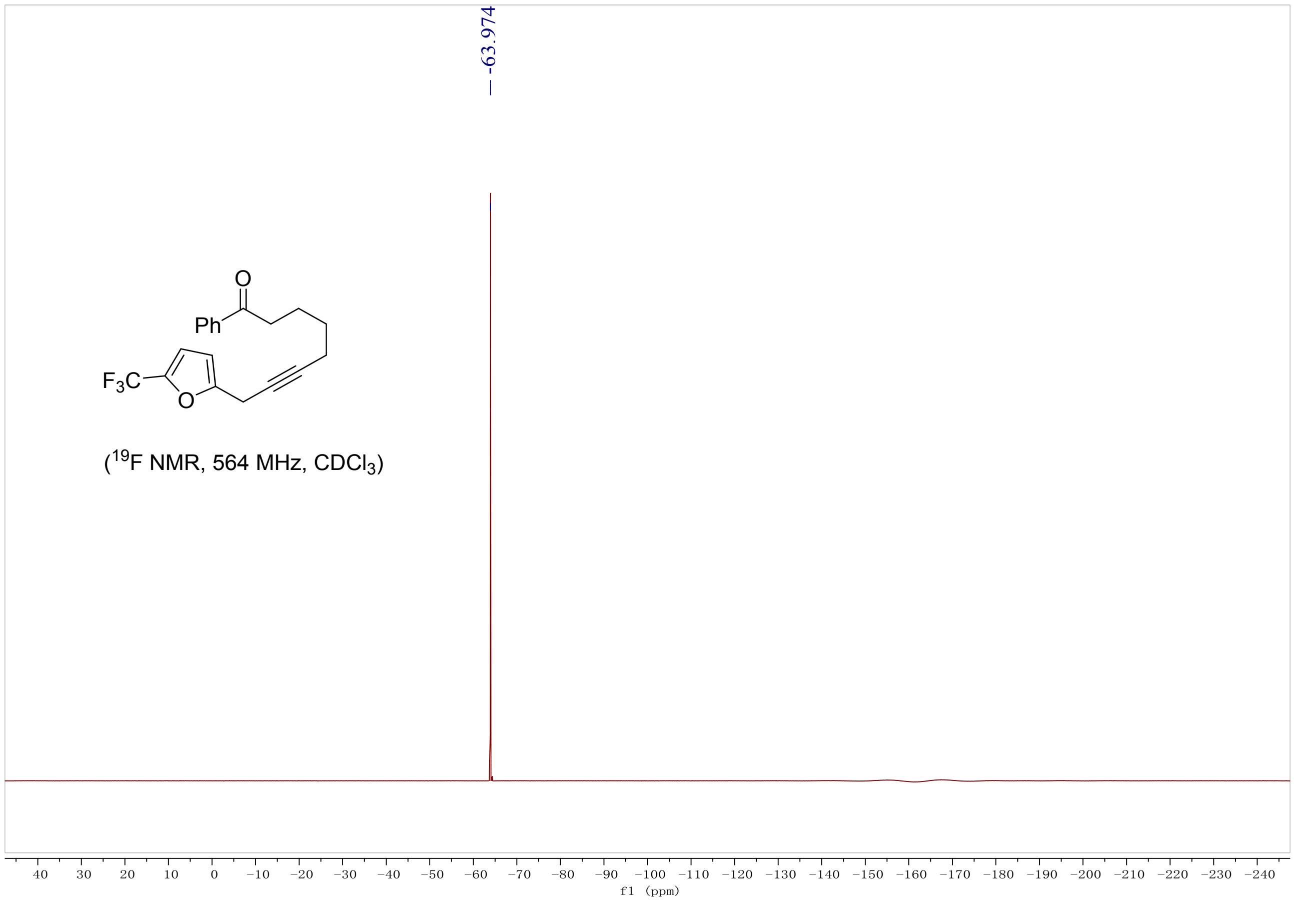

-63.974
O
Ph
F_3C
O
(^{19}F NMR, 564 MHz, $CDCl_3$)
40 30 20 10 0 −10 −20 −30 −40 −50 −60 −70 −80 −90 −100 −110 −120 −130 −140 −150 −160 −170 −180 −190 −200 −210 −220 −230 −240
f1 (ppm)

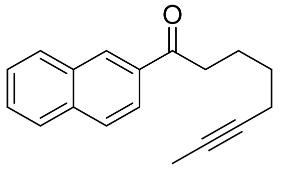

1-(naphthalen-2-yl)oct-6-yn-1-one (3bb): colorless solid, m.p. 165-167 °C, 43 mg, 87%, Eluent: PE/EA = 20/1.

^{1}H NMR (400 MHz, $CDCl_3$, TMS): δ 8.48 (s, 1H), 8.04 (dd, *J* = 8.6, 1.6 Hz, 1H), 7.97 (d, *J* = 8.0 Hz, 1H), 7.92-7.86 (m, 2H), 7.64-7.53 (m, 2H), 3.13 (t, *J* = 7.4 Hz, 2H), 2.27-2.17 (m, 2H), 1.96-1.86 (m, 2H), 1.77 (t, *J* = 2.4 Hz, 3H), 1.67-1.60 (m, 2H). **^{13}C NMR** (150 MHz, $CDCl_3$, TMS): δ 3.5, 18.6, 23.7, 28.6, 38.1, 75.9, 78.8, 123.9, 126.7, 127.8, 128.36, 128.41, 129.5, 129.6, 132.5, 134.3, 135.5, 200.2. **IR** (EtOH): ν 2939, 2872, 2200, 1682, 1597, 1448, 1223, 1180, 851, 751, 732, 690 cm^{-1}. **HRMS** (ESI) m/z: $[M+Na]^+$ Calcd. for $C_{18}H_{18}ONa$ 273.1250; found 273.1258.

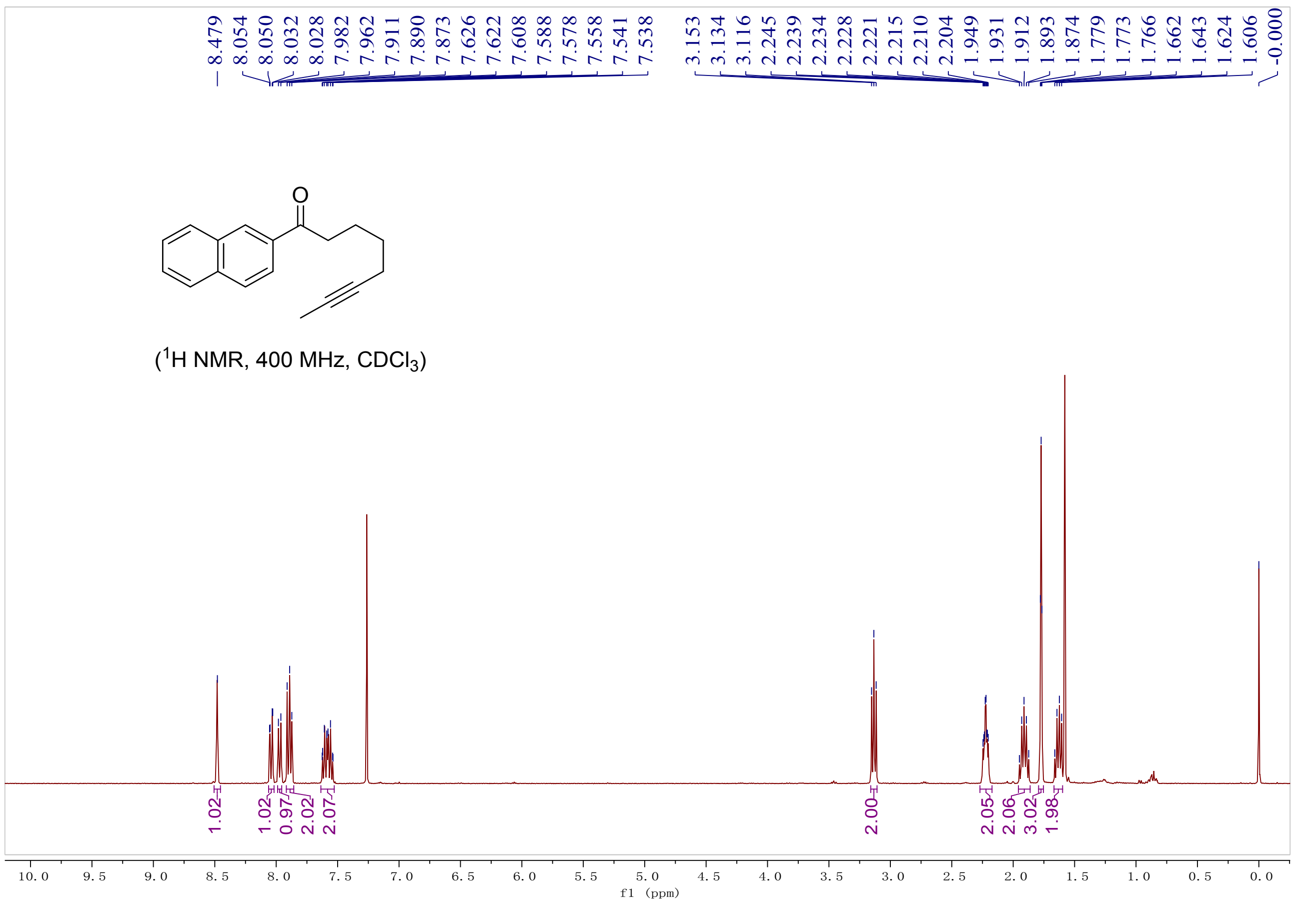
8.479
8.054
8.050
8.032
8.028
7.982
7.962
7.911
7.890
7.873
7.626
7.622
7.608
7.588
7.578
7.558
7.541
7.538
3.153
3.134
3.116
2.245
2.239
2.234
2.228
2.221
2.215
2.210
2.204
1.949
1.931
1.912
1.893
1.874
1.779
1.773
1.766
1.662
1.643
1.624
1.606
-0.000
O
(1H NMR, 400 MHz, CDCl3)
1.02
1.02
0.97
2.02
2.07
2.00
2.05
2.06
3.02
1.98
f1 (ppm)

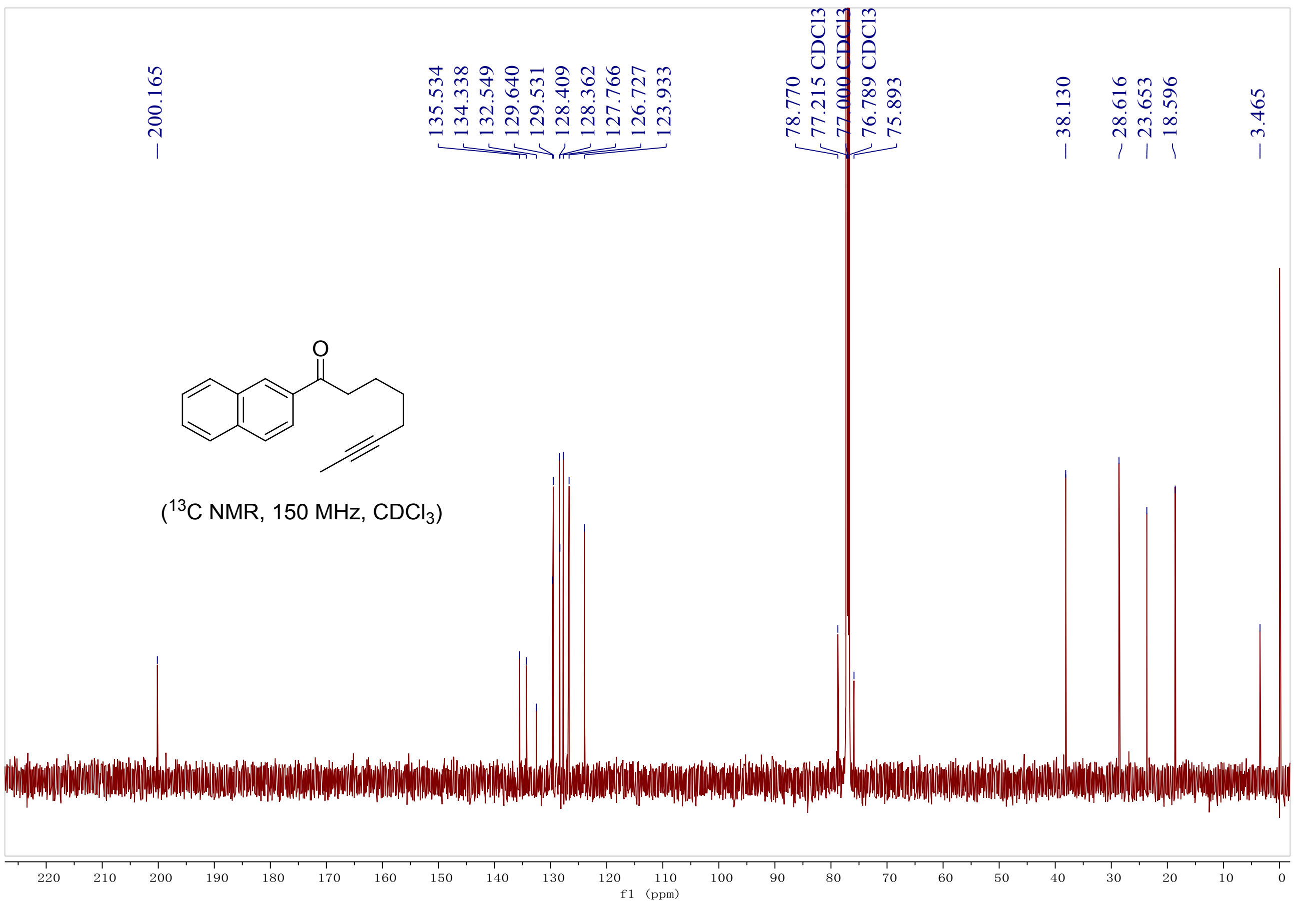
200.165
135.534
134.338
132.549
129.640
129.531
128.409
128.362
127.766
126.727
123.933
78.770
77.215 CDCl3
77.000 CDCl3
76.789 CDCl3
75.893
38.130
28.616
23.653
18.596
3.465
O
(13C NMR, 150 MHz, CDCl3)
f1 (ppm)

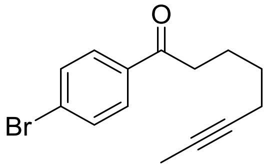

1-(4-bromophenyl)oct-6-yn-1-one (3bc) colorless oil, 51 mg, 92%, Eluent: PE/EA = 20/1.

^{1}H NMR (400 MHz, $CDCl_3$, TMS): δ 7.83 (d, *J* = 8.6 Hz, 2H), 7.60 (d, *J* = 8.6 Hz, 2H), 2.95 (t, *J* = 7.4 Hz, 2H), 2.24-2.14 (m, 2H), 1.87-1.80 (m, 2H), 1.77 (t, *J* = 2.6 Hz, 3H), 1.60-1.53 (m, 2H). **^{13}C NMR** (100 MHz, $CDCl_3$, TMS): δ 3.4, 18.5, 23.4, 28.5, 38.0, 75.9, 78.6, 128.1, 129.6, 131.9, 135.7, 199.1. **IR** (EtOH): ν 2973, 2883, 2219, 1653, 1534, 1379, 1086, 1045, 880, 801, 627 cm^{-1}. **HRMS** (EI) m/z: $[M]^+$ Calcd. for $C_{14}H_{14}OBr$ 277.0223; found 277.0212.

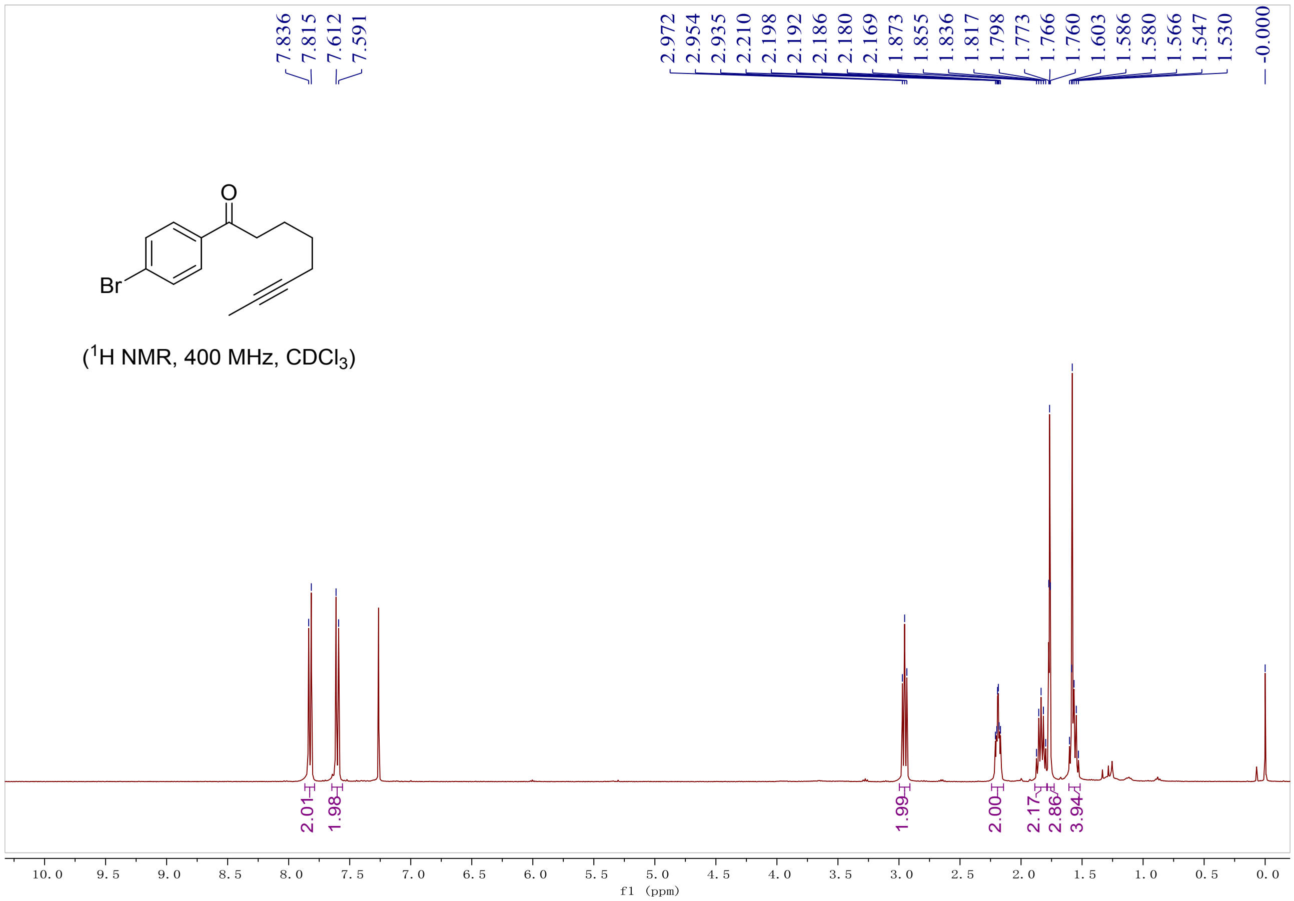

7.836
7.815
7.612
7.591
2.972
2.954
2.935
2.210
2.198
2.192
2.186
2.180
2.169
1.873
1.855
1.836
1.817
1.798
1.773
1.766
1.760
1.603
1.586
1.580
1.566
1.547
1.530
-0.000
O
Br
(¹H NMR, 400 MHz, CDCl₃)
2.01
1.98
1.99
2.00
2.17
2.86
3.94
f1 (ppm)

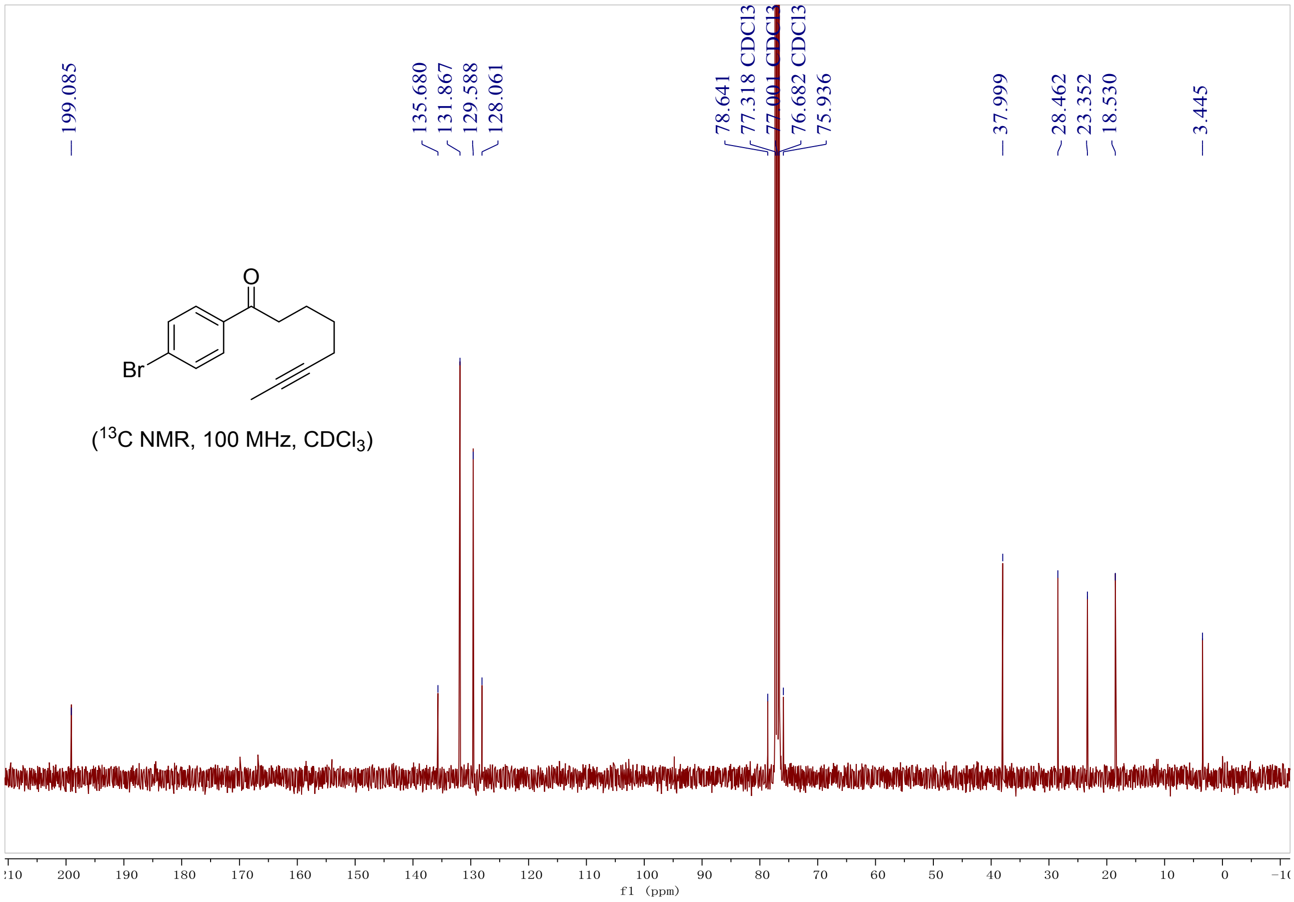

199.085
135.680
131.867
129.588
128.061
78.641
77.318 CDCl3
77.001 CDCl3
76.682 CDCl3
75.936
37.999
28.462
23.352
18.530
3.445
O
Br
(¹³C NMR, 100 MHz, CDCl₃)
f1 (ppm)

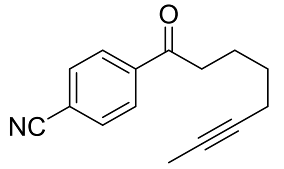

4-(oct-6-ynoyl)benzonitrile (3bd): colorless oil, 36 mg, 79%, Eluent: PE/EA = 20/1.

^{1}H NMR (600 MHz, $CDCl_3$, TMS): δ 8.04 (d, J = 8.6 Hz, 2H), 7.77 (d, J = 8.6 Hz, 2H), 3.01 (t, J = 7.2 Hz, 2H), 2.20 (tq, J = 7.2, 2.6 Hz, 2H), 1.90-1.82 (m, 2H), 1.76 (t, J = 2.4 Hz, 3H), 1.60-1.55 (m, 2H). **^{13}C NMR** (150 MHz, $CDCl_3$, TMS): δ 3.4, 18.5, 23.1, 28.3, 38.3, 76.0, 78.5, 116.2, 118.0, 128.4, 132.5, 139.9, 198.6. **IR** (EtOH): ν 2974, 2921, 2893, 2211, 1645, 1380, 1327, 1086, 1044, 879, 737, 653 cm^{-1}. **HRMS** (EI) m/z: $[M]^+$ Calcd. for $C_{15}H_{14}ON$ 224.1070; found 224.1061.

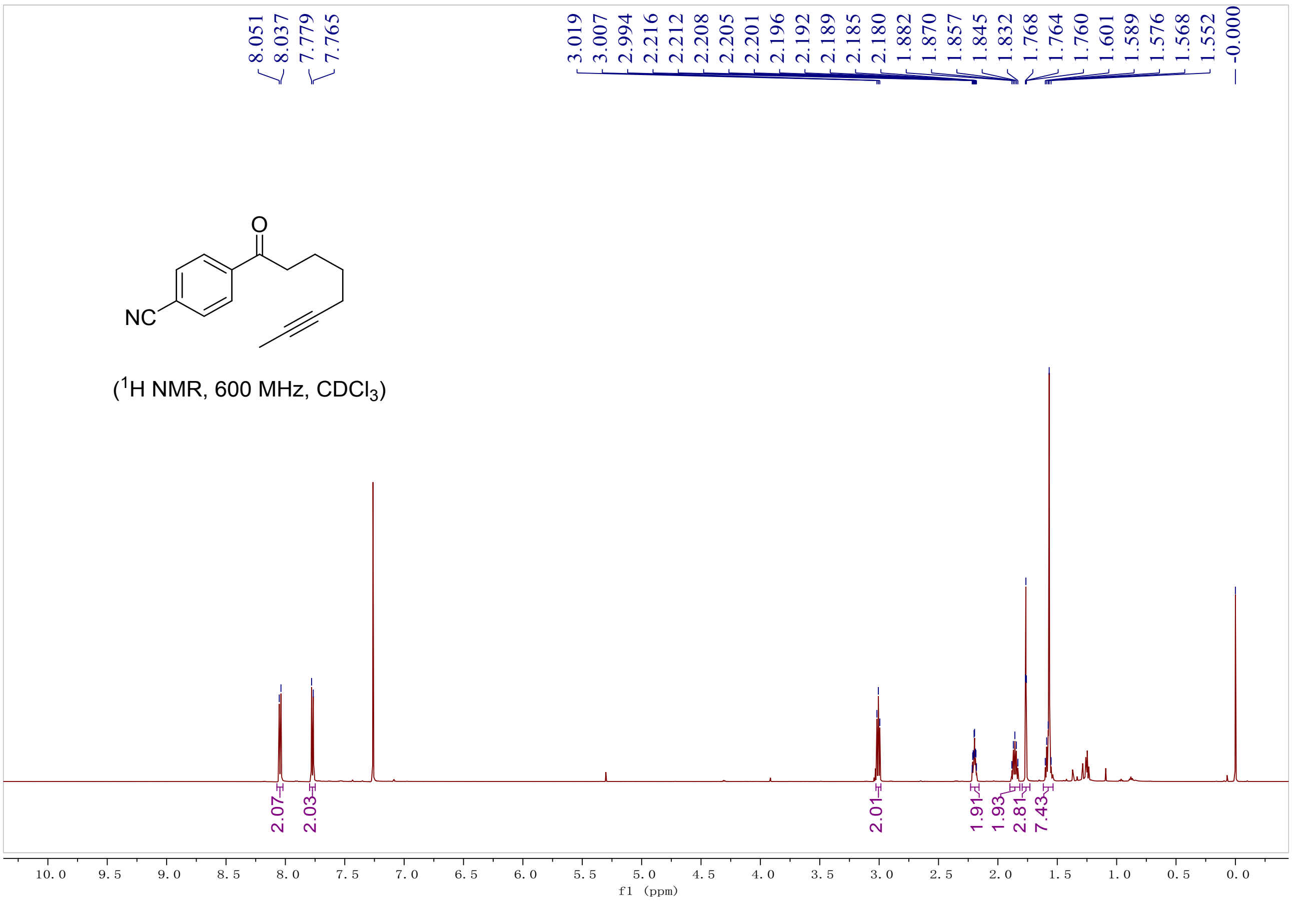
8.051
8.037
7.779
7.765
3.019
3.007
2.994
2.216
2.212
2.208
2.205
2.201
2.196
2.192
2.189
2.185
2.180
1.882
1.870
1.857
1.845
1.832
1.768
1.764
1.760
1.601
1.589
1.576
1.568
1.552
-0.000
O
NC
(1H NMR, 600 MHz, CDCl3)
2.07
2.03
2.01
1.91
1.93
2.81
7.43
f1 (ppm)

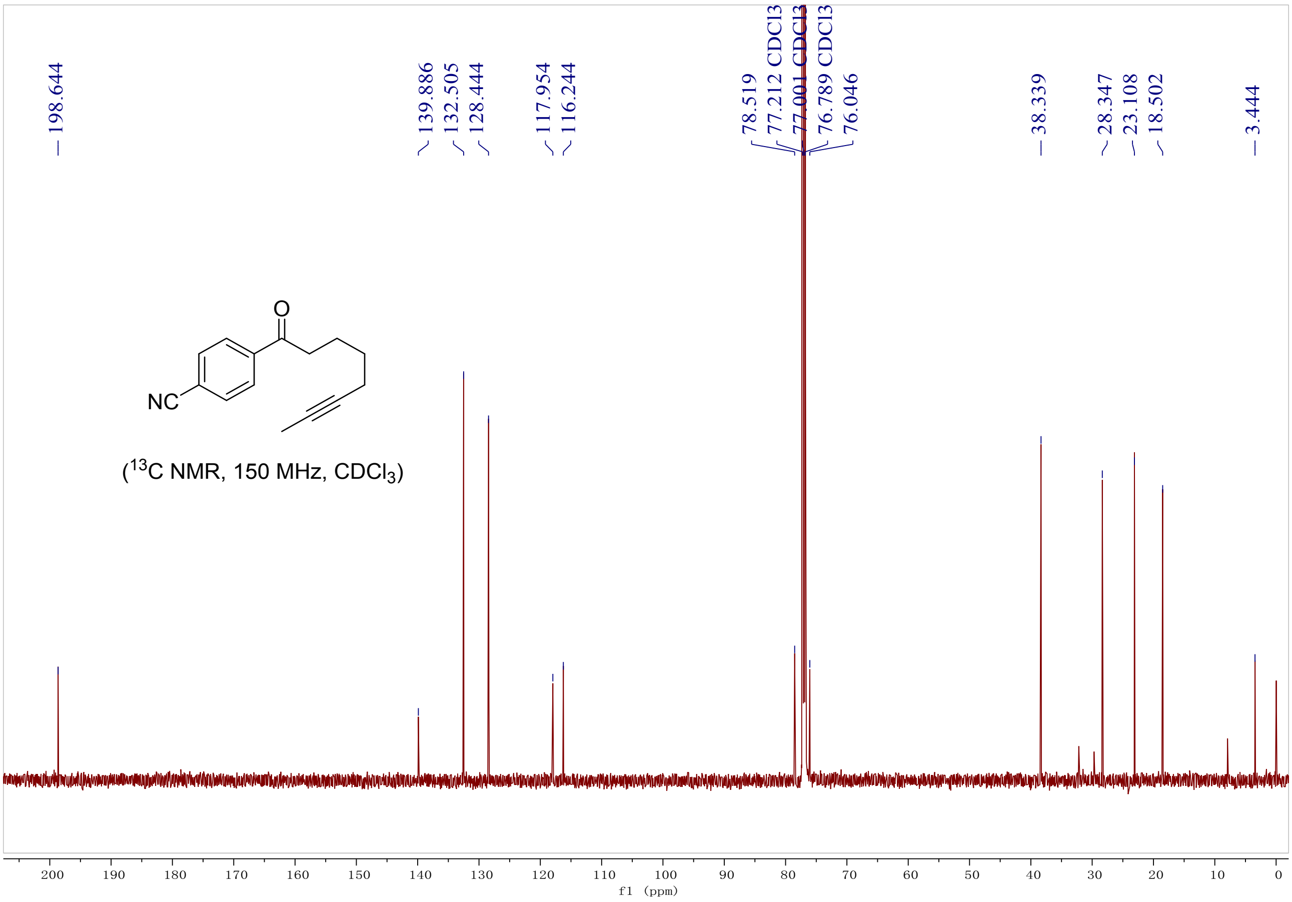
198.644
139.886
132.505
128.444
117.954
116.244
78.519
77.212 CDCl3
77.001 CDCl3
76.789 CDCl3
76.046
38.339
28.347
23.108
18.502
3.444
O
NC
(13C NMR, 150 MHz, CDCl3)
f1 (ppm)

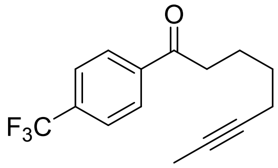

1-(4-(trifluoromethyl)phenyl)oct-6-yn-1-one (3be): colorless oil, 40 mg, 75%, Eluent: PE/EA = 20/1.

^{1}H NMR (400 MHz, $CDCl_3$, TMS): δ 8.06 (d, *J* = 8.0 Hz, 2H), 7.73 (d, *J* = 8.0 Hz, 2H), 3.02 (t, *J* = 7.2 Hz, 2H), 2.20 (tq, *J* = 7.2, 2.6 Hz, 2H), 1.91-1.82 (m, 2H), 1.77 (t, *J* = 2.6 Hz, 3H), 1.62-1.57 (m, 2H). **^{13}C NMR** (150 MHz, $CDCl_3$, TMS): δ 3.4, 18.5, 23.2, 28.4, 38.3, 76.0, 78.6, 123.6 (q, *J* = 225.3 Hz), 125.7 (q, *J* = 3.4 Hz), 128.4, 134.3 (q, *J* = 26.5 Hz), 139.6, 199.1. ^{19}F NMR (376 MHz, $CDCl_3$): δ -63.1; **IR** (EtOH): ν 2940, 2910, 2882, 2115, 1672, 1576, 1448, 1367, 1020, 1009, 971, 738, 663 cm^{-1}. **HRMS** (EI) m/z: $[M]^+$ Calcd. for $C_{15}H_{15}OF_3$ 268.1075; found 268.1070.

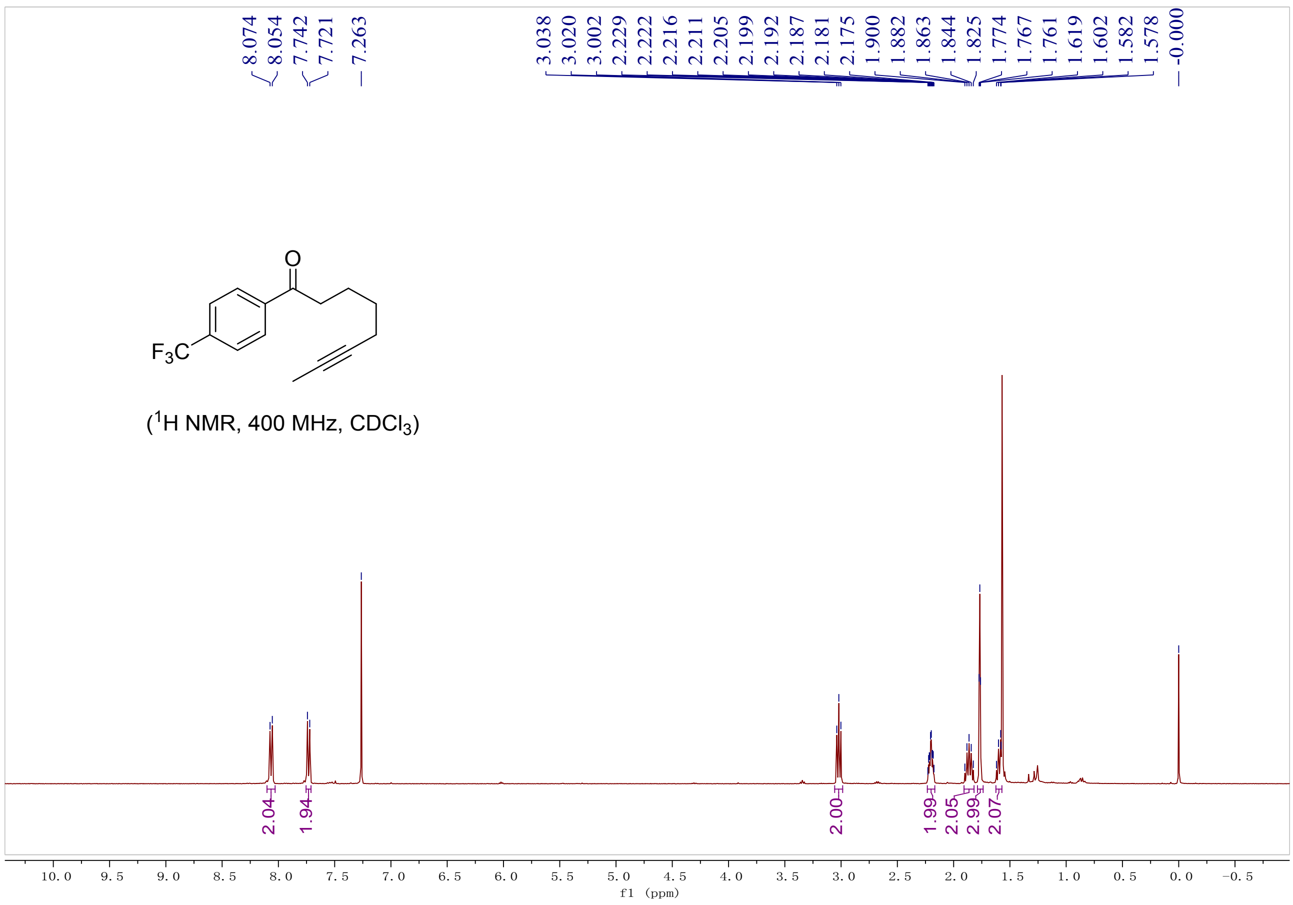
8.074
8.054
7.742
7.721
7.263
3.038
3.020
3.002
2.229
2.222
2.216
2.211
2.205
2.199
2.192
2.187
2.181
2.175
1.900
1.882
1.863
1.844
1.825
1.774
1.767
1.761
1.619
1.602
1.582
1.578
-0.000
O
F3C
(1H NMR, 400 MHz, CDCl3)
2.04
1.94
2.00
1.99
2.05
2.99
2.07
f1 (ppm)

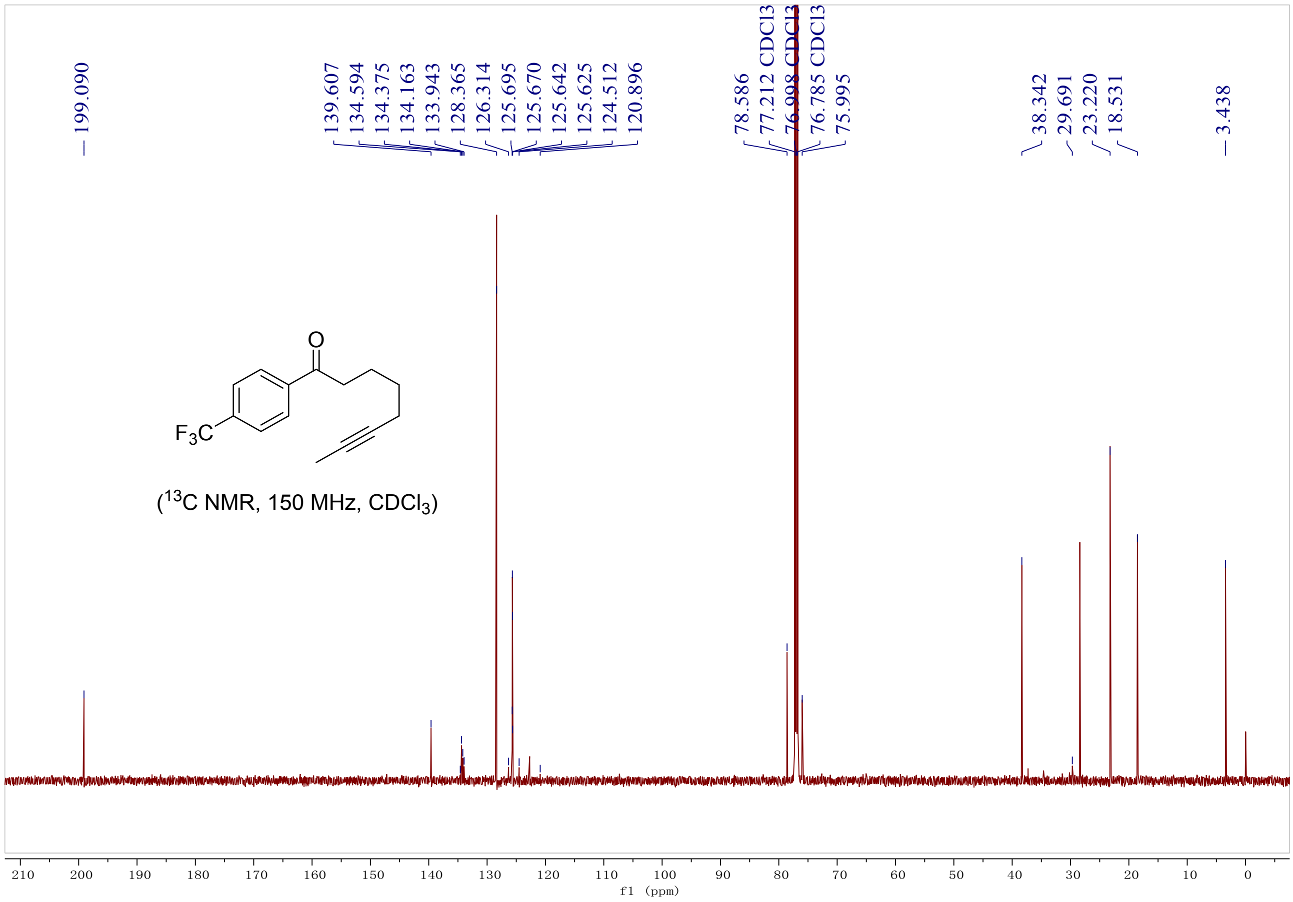
199.090
139.607
134.594
134.375
134.163
133.943
128.365
126.314
125.695
125.670
125.642
125.625
124.512
120.896
78.586
77.212 CDCl3
76.998 CDCl3
76.785 CDCl3
75.995
38.342
29.691
23.220
18.531
3.438
O
F3C
(13C NMR, 150 MHz, CDCl3)
f1 (ppm)

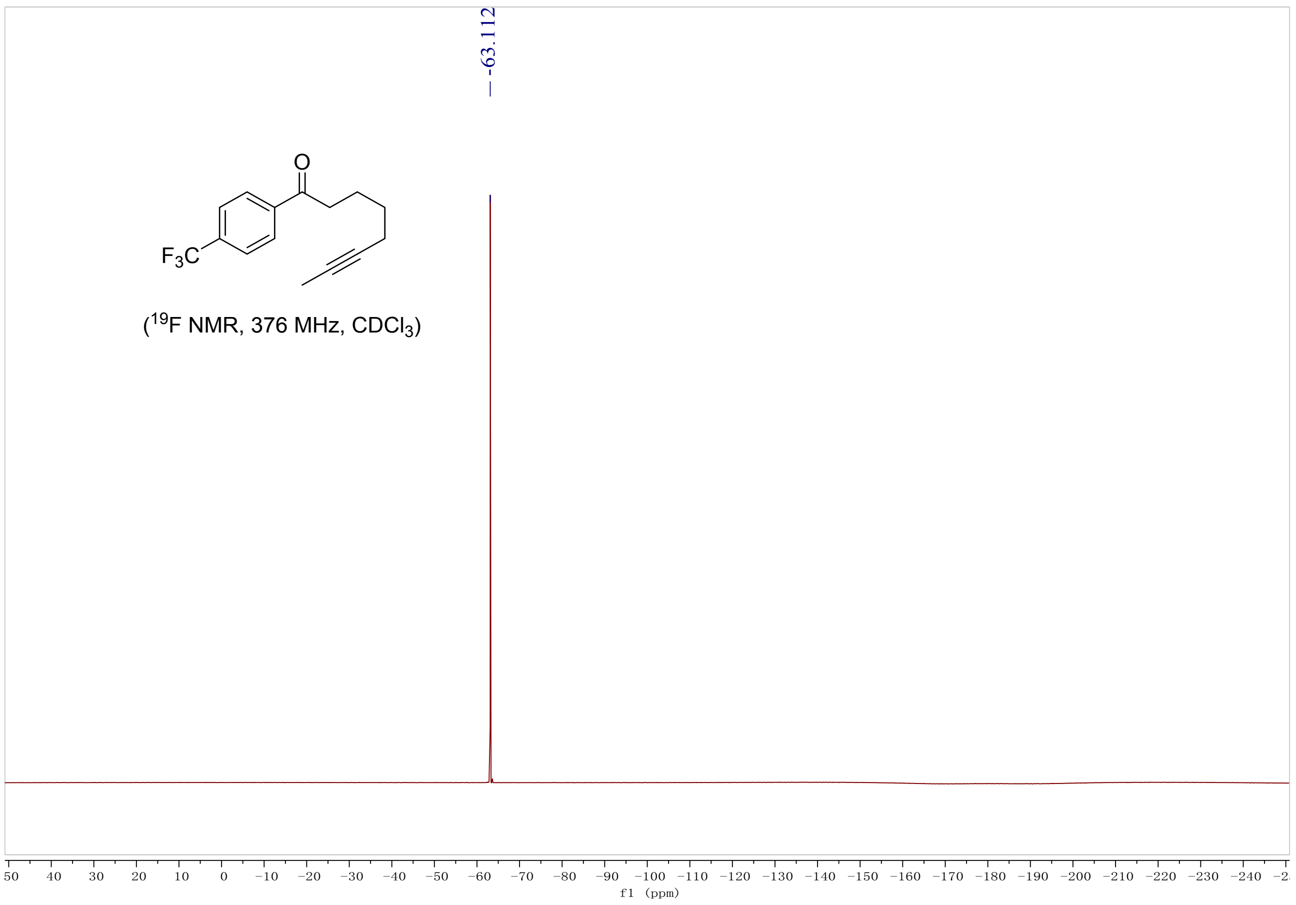

-63.112
F3C
O
(19F NMR, 376 MHz, CDCl3)
50 40 30 20 10 0 −10 −20 −30 −40 −50 −60 −70 −80 −90 −100 −110 −120 −130 −140 −150 −160 −170 −180 −190 −200 −210 −220 −230 −240
f1 (ppm)

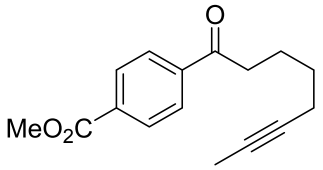

Methyl 4-(oct-6-ynoyl)benzoate (3bf): colorless oil, 44 mg, 84%, Eluent: PE/EA = 20/1.

^{1}H NMR (400 MHz, $CDCl_3$, TMS): δ 8.12 (d, *J* = 8.4 Hz, 2H), 8.01 (d, *J* = 8.4 Hz, 2H), 3.95 (s, 3H), 3.02 (t, *J* = 7.4 Hz, 2H), 2.20 (tq, *J* = 7.2, 2.6 Hz, 2H), 1.90-1.83 (m, 2H), 1.77 (t, *J* = 2.4 Hz, 3H), 1.64-1.52 (m, 2H). **^{13}C NMR** (100 MHz, $CDCl_3$, TMS): δ 3.5, 18.5, 23.3, 28.4, 38.4, 52.5, 76.0, 78.6, 127.9, 129.8, 133.7, 140.2, 166.3, 199.6. **IR** (EtOH): ν 2978, 2945, 2218, 1671, 1607, 1507, 1357, 1269, 1229, 1087, 995, 855, 724, 652 cm^{-1}. **HRMS** (ESI) m/z: $[M+Na]^+$ Calcd. for $C_{16}H_{18}O_3Na$ 281.1148; found 281.1153.

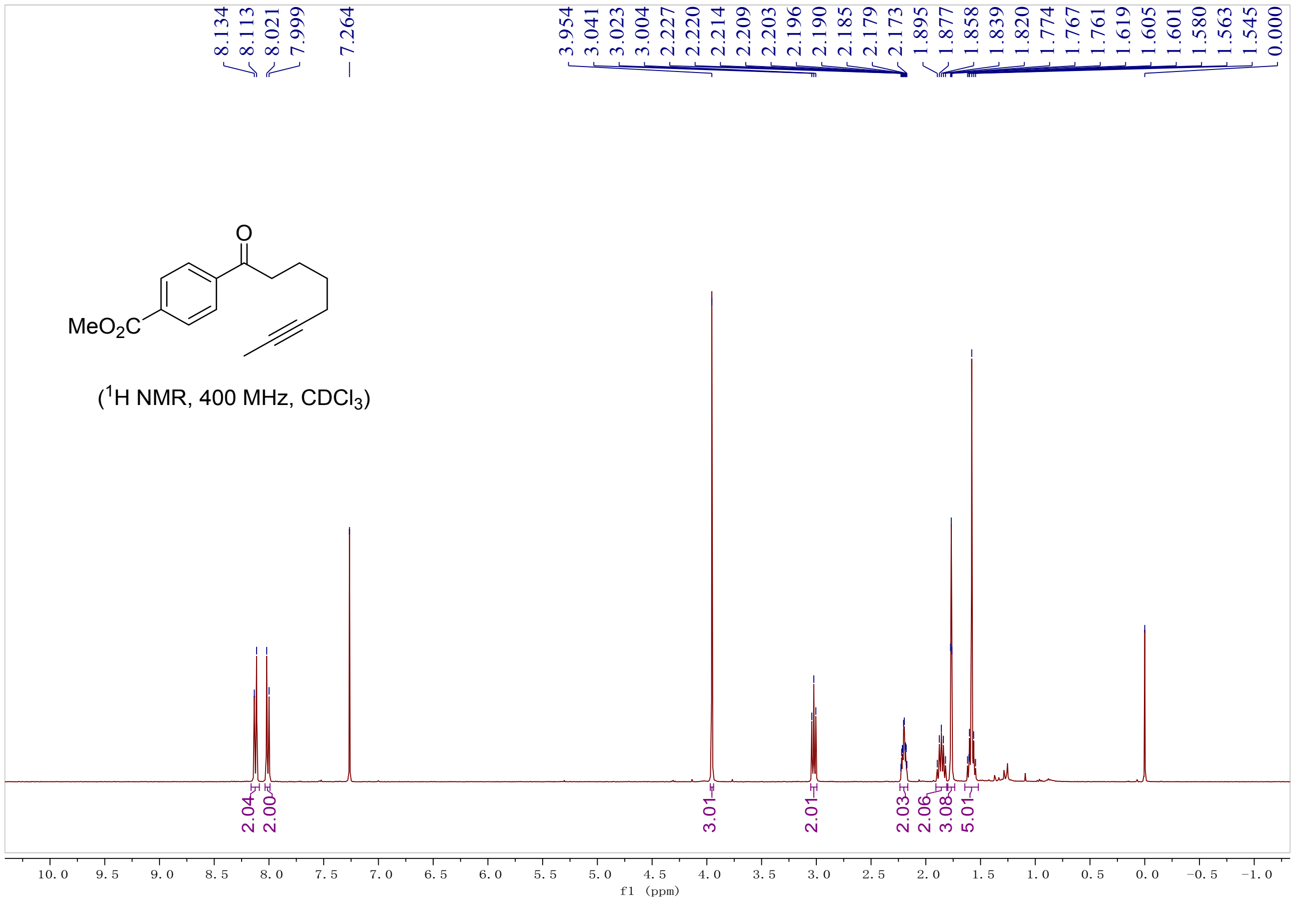

8.134
8.113
8.021
7.999
7.264
3.954
3.041
3.023
3.004
2.227
2.220
2.214
2.209
2.203
2.196
2.190
2.185
2.179
2.173
1.895
1.877
1.858
1.839
1.820
1.774
1.767
1.761
1.619
1.605
1.601
1.580
1.563
1.545
0.000
O
MeO2C
(1H NMR, 400 MHz, CDCl3)
2.04
2.00
3.01
2.01
2.03
2.06
3.08
5.01
f1 (ppm)

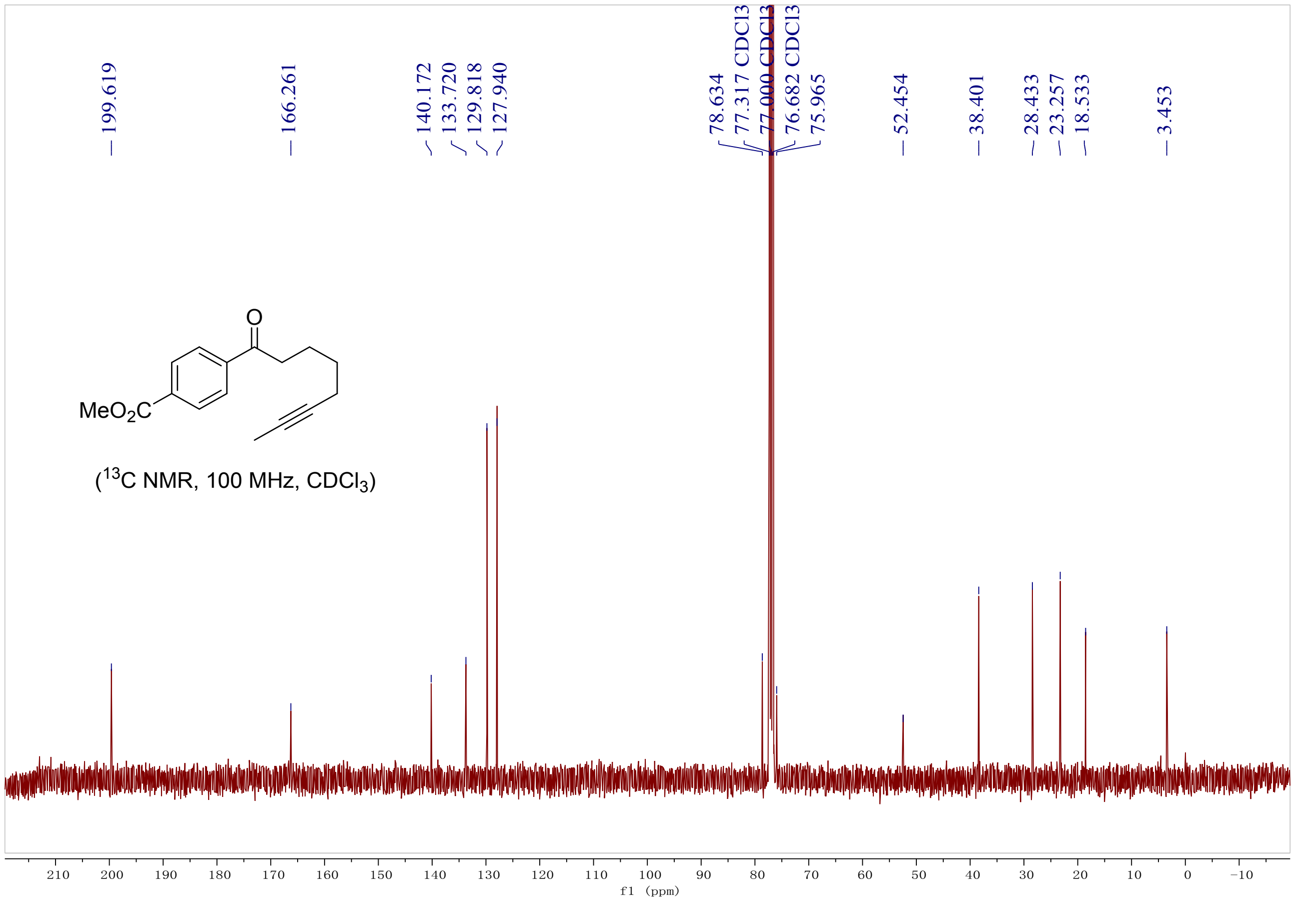

199.619
166.261
140.172
133.720
129.818
127.940
78.634
77.317 CDCl3
77.000 CDCl3
76.682 CDCl3
75.965
52.454
38.401
28.433
23.257
18.533
3.453
O
MeO2C
(13C NMR, 100 MHz, CDCl3)
f1 (ppm)

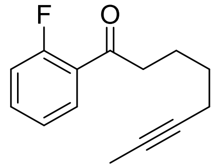

1-(2-fluorophenyl)oct-6-yn-1-one (3bg): colorless oil, 34 mg, 79%, Eluent: PE/EA = 20/1.

^{1}H NMR (600 MHz, $CDCl_3$, TMS): δ 7.85 (td, *J* = 7.6, 2.0 Hz, 1H), 7.54-7.47 (m, 1H), 7.25-7.19 (m, 1H), 7.15-7.09 (m, 1H), 3.02-2.98 (m, 2H), 2.18 (tq, *J* = 7.2, 2.6 Hz, 2H), 1.85-1.79 (m, 2H), 1.76 (t, *J* = 2.4 Hz, 3H), 1.59-1.53 (m, 2H). **^{13}C NMR** (150 MHz, $CDCl_3$, TMS): δ 3.4, 18.6, 23.2, 28.5, 43.1, 75.8, 78.8, 116.6 (d, *J* = 23.6 Hz), 124.4 (d, *J* = 3.9 Hz), 125.9 (d, *J* = 13.7 Hz), 130.6, 134.3 (d, *J* = 9.6 Hz), 162.4 (d, *J* = 253.5 Hz), 198.6. ^{19}F NMR (564 MHz, $CDCl_3$): δ -109.6; **IR** (EtOH): ν 2939, 2866, 2214, 1682, 1597, 1458, 1213, 1002, 808, 752, 732, 690 cm^{-1}. **HRMS** (ESI) m/z: $[M+Na]^+$ Calcd. for $C_{14}H_{15}OFNa$ 241.0999; found 241.1006.

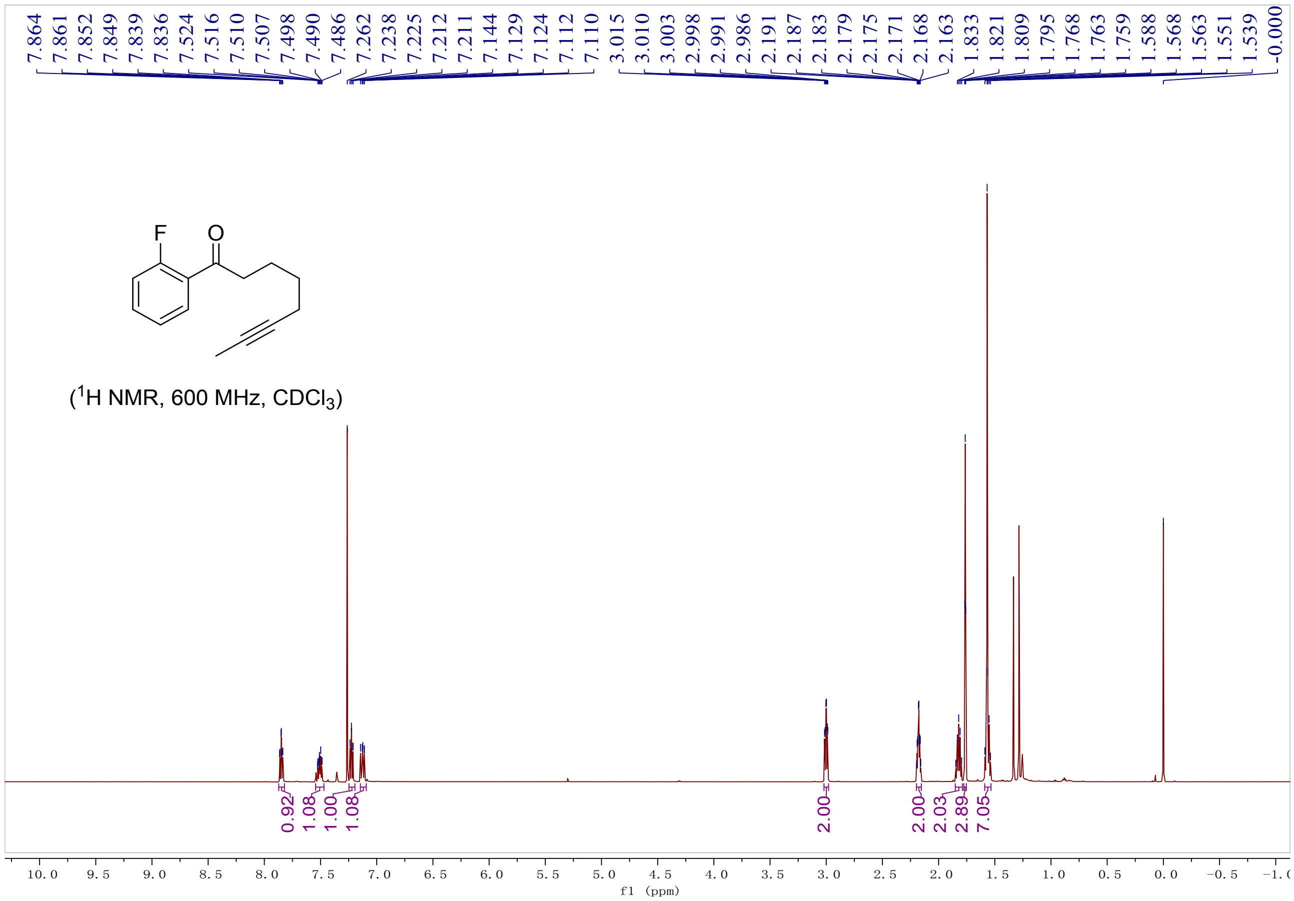
7.864
7.861
7.852
7.849
7.839
7.836
7.524
7.516
7.510
7.507
7.498
7.490
7.486
7.262
7.238
7.225
7.212
7.211
7.144
7.129
7.124
7.112
7.110
3.015
3.010
3.003
2.998
2.991
2.986
2.191
2.187
2.183
2.179
2.175
2.171
2.168
2.163
1.833
1.821
1.809
1.795
1.768
1.763
1.759
1.588
1.568
1.563
1.551
1.539
-0.000
F
O
(1H NMR, 600 MHz, CDCl3)
0.92
1.08
1.00
1.08
2.00
2.00
2.03
2.89
7.05
f1 (ppm)

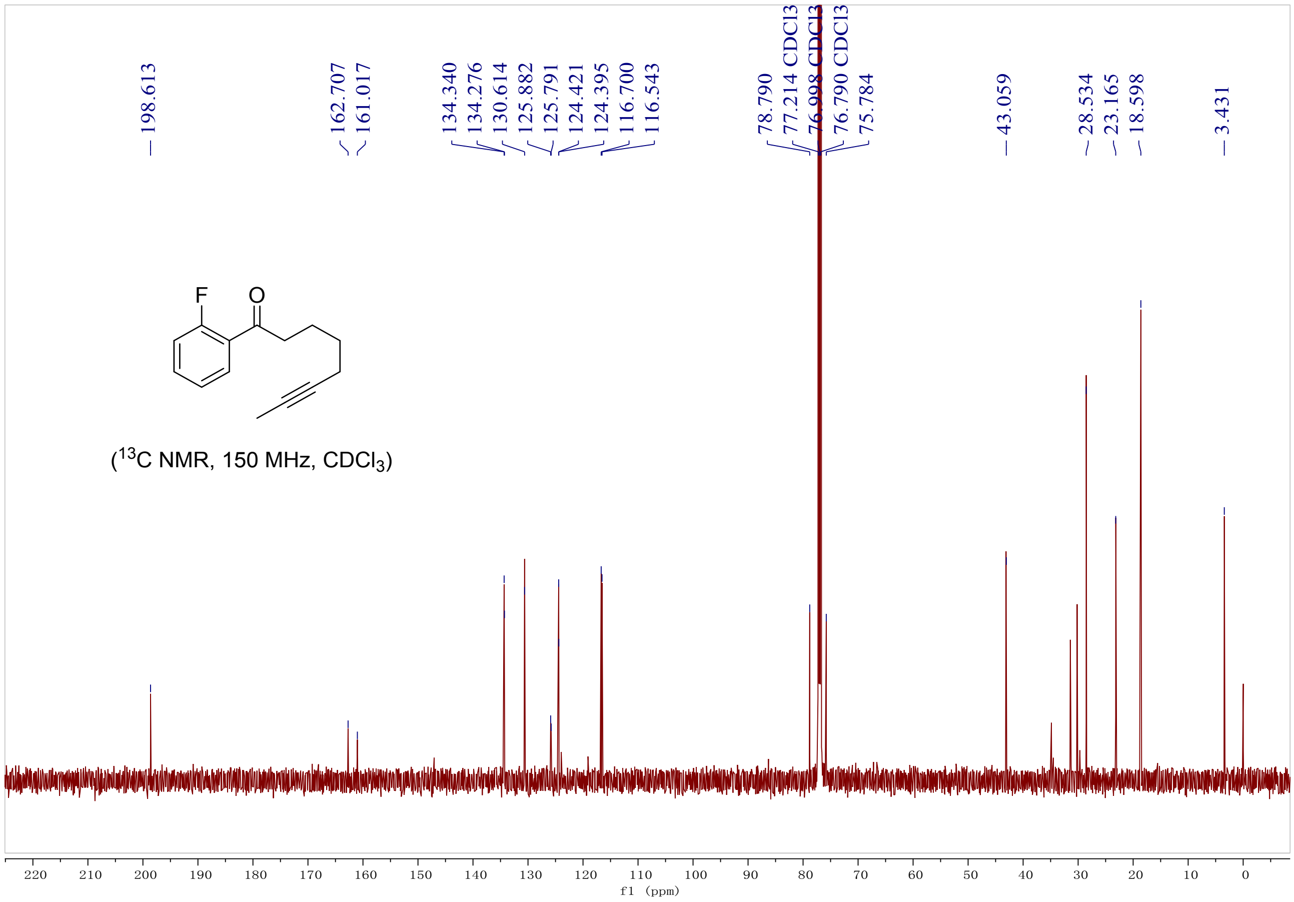
198.613
162.707
161.017
134.340
134.276
130.614
125.882
125.791
124.421
124.395
116.700
116.543
78.790
77.214 CDCl3
76.998 CDCl3
76.790 CDCl3
75.784
43.059
28.534
23.165
18.598
3.431
F
O
(13C NMR, 150 MHz, CDCl3)
f1 (ppm)

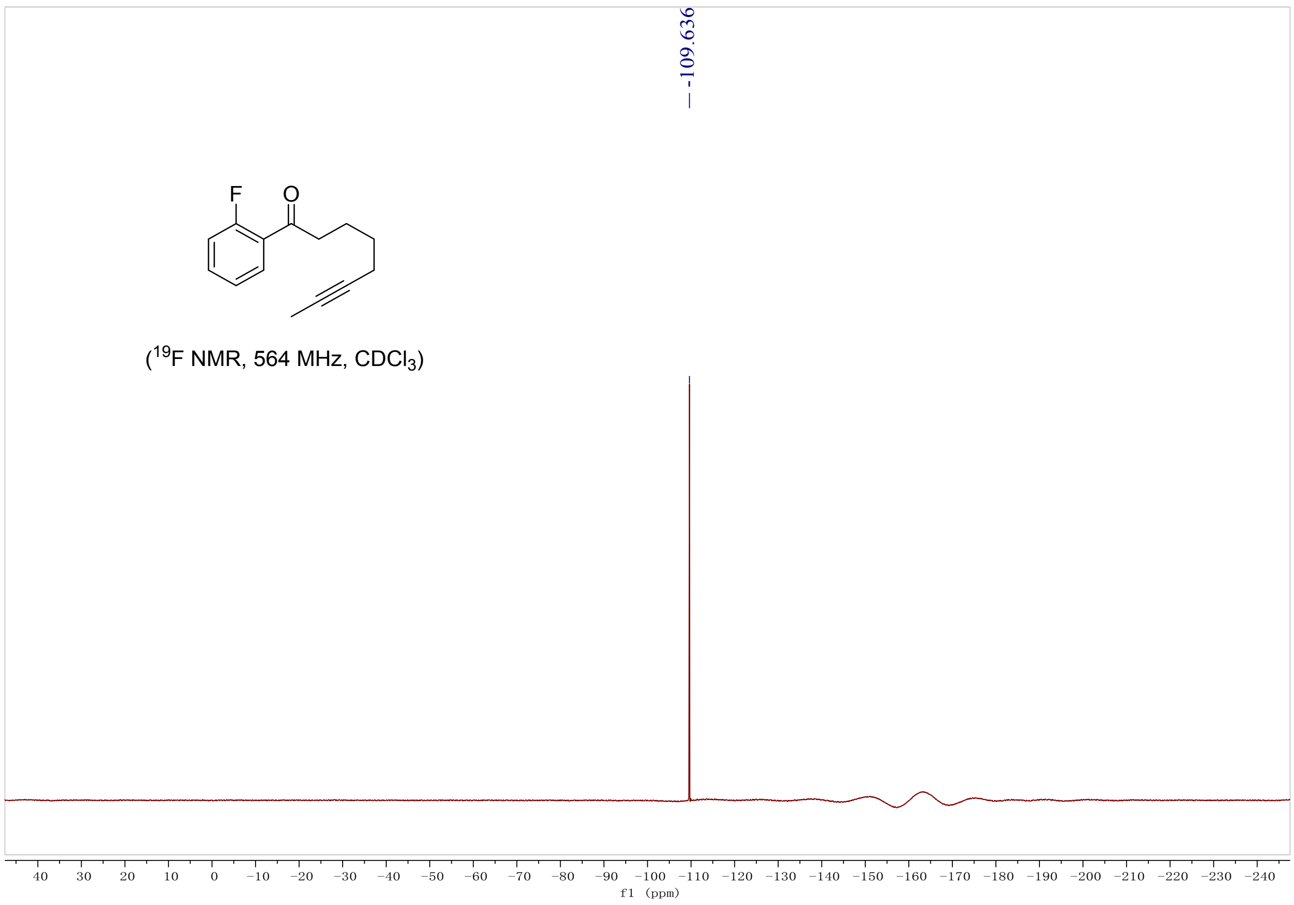

-109.636
F
O
(19F NMR, 564 MHz, CDCl3)
40 30 20 10 0 −10 −20 −30 −40 −50 −60 −70 −80 −90 −100 −110 −120 −130 −140 −150 −160 −170 −180 −190 −200 −210 −220 −230 −240
f1 (ppm)

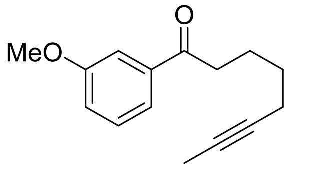

1-(3-methoxyphenyl)oct-6-yn-1-one (3bh): colorless oil, 42 mg, 92%, Eluent: PE/EA = 20/1.

^{1}H NMR (400 MHz, $CDCl_3$, TMS): δ 7.54 (d, *J* = 7.8 Hz, 1H), 7.51-7.46 (m, 1H), 7.36 (t, *J* = 7.8 Hz, 1H), 7.10 (ddd, *J* = 8.2, 2.8, 1.0 Hz, 1H), 3.85 (s, 3H), 2.97 (t, *J* = 7.4 Hz, 2H), 2.19 (tq, *J* = 7.2, 2.6 Hz, 2H), 1.88-1.80 (m, 2H), 1.77 (t, *J* = 2.6 Hz, 3H), 1.61-1.53 (m, 2H). **^{13}C NMR** (100 MHz, $CDCl_3$, TMS): δ 3.4, 18.5, 23.5, 28.5, 38.1, 55.3, 75.8, 78.7, 112.3, 119.3, 120.6, 129.5, 138.3, 159.8, 199.9. **IR** (EtOH): ν 2941, 2925, 2199, 1681, 1600, 1450, 1221, 1194, 1080, 1002, 841, 776, 737, 682 cm^{-1}. **HRMS** (DART) m/z: $[M+H]^+$ Calcd. for $C_{15}H_{19}O_2$ 231.1380; found 231.1378.

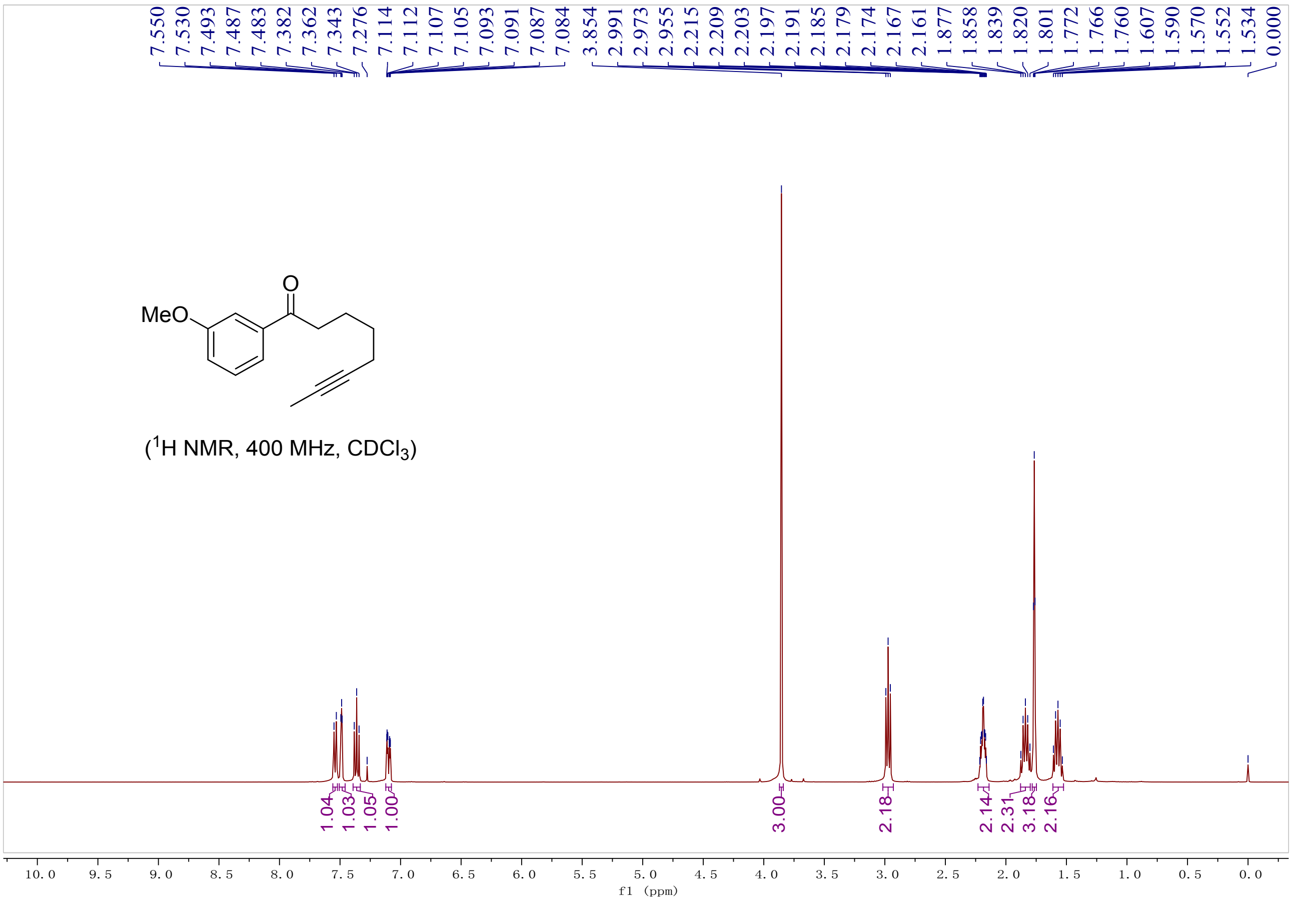

7.550
7.530
7.493
7.487
7.483
7.382
7.362
7.343
7.276
7.114
7.112
7.107
7.105
7.093
7.091
7.087
7.084
3.854
2.991
2.973
2.955
2.215
2.209
2.203
2.197
2.191
2.185
2.179
2.174
2.167
2.161
1.877
1.858
1.839
1.820
1.801
1.772
1.766
1.760
1.607
1.590
1.570
1.552
1.534
0.000
MeO
O
(^{1}H NMR, 400 MHz, CDCl$_3$)
1.04
1.03
1.05
1.00
3.00
2.18
2.14
2.31
3.18
2.16
10.0 9.5 9.0 8.5 8.0 7.5 7.0 6.5 6.0 5.5 5.0 4.5 4.0 3.5 3.0 2.5 2.0 1.5 1.0 0.5 0.0
f1 (ppm)

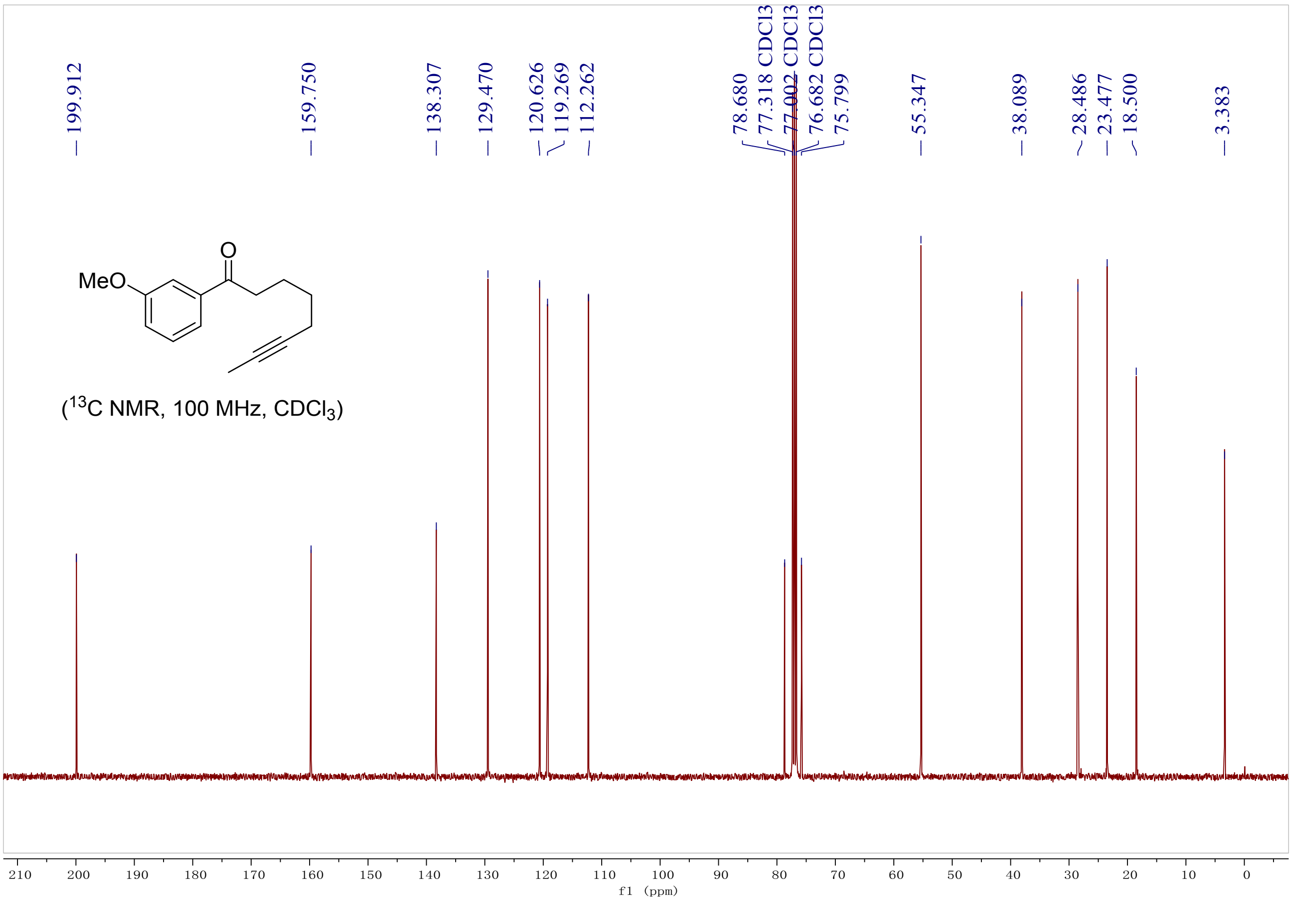

199.912
159.750
138.307
129.470
120.626
119.269
112.262
78.680
77.318 CDCl3
77.002 CDCl3
76.682 CDCl3
75.799
55.347
38.089
28.486
23.477
18.500
3.383
MeO
O
(^{13}C NMR, 100 MHz, CDCl$_3$)
210 200 190 180 170 160 150 140 130 120 110 100 90 80 70 60 50 40 30 20 10 0
f1 (ppm)

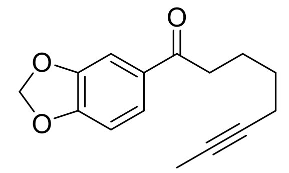

1-phenyloct-6-yn-1-one (3bi): colorless oil, 40 mg, 81%, Eluent: PE/EA = 20/1.

^{1}H NMR (400 MHz, $CDCl_3$, TMS): δ 7.57 (dd, J = 8.2, 1.8 Hz, 1H), 7.44 (d, J = 1.8 Hz, 1H), 6.85 (d, J = 8.2 Hz, 1H), 6.04 (s, 2H), 2.91 (t, J = 7.2 Hz, 2H), 2.19 (tq, J = 7.2, 2.6 Hz, 2H), 1.86-1.75 (m, 5H), 1.59-1.51 (m, 2H). **^{13}C NMR** (100 MHz, $CDCl_3$, TMS): δ 3.4, 18.5, 23.7, 28.6, 37.8, 75.9, 78.7, 101.8, 107.8, 107.9, 124.2, 131.9, 148.1, 151.6, 198.3. **IR** (EtOH): ν 2936, 2920, 2199, 2866, 1682, 1599, 1278, 1257, 1223, 1194, 1180, 1000, 789, 752, 700, 663 cm^{-1}. **HRMS** (ESI) m/z: $[M+Na]^+$ Calcd. for $C_{15}H_{16}O_3Na$ 267.0992; found 267.0998.

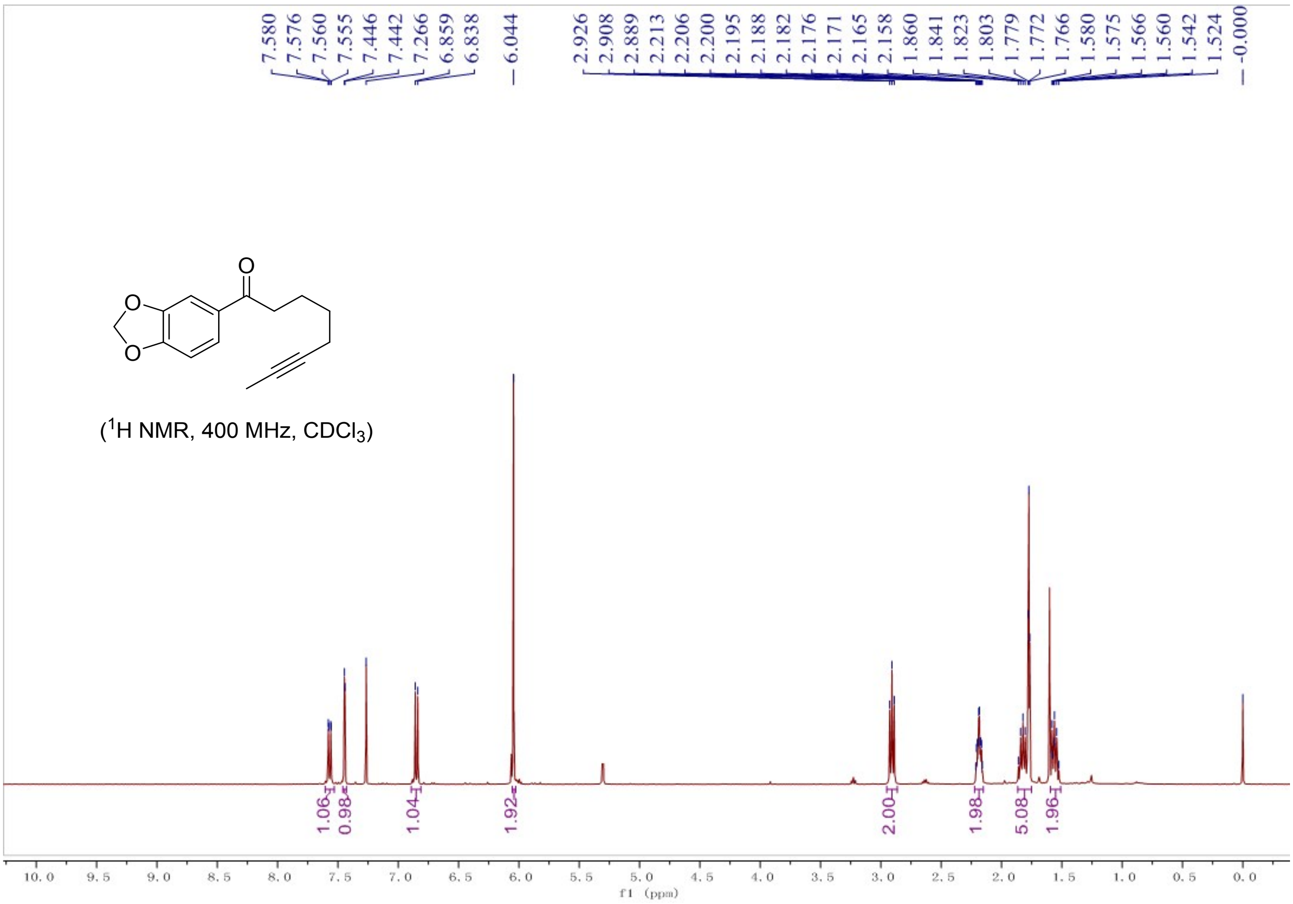
7.580
7.576
7.560
7.555
7.446
7.442
7.266
6.859
6.838
6.044
2.926
2.908
2.889
2.213
2.206
2.200
2.195
2.188
2.182
2.176
2.171
2.165
2.158
1.860
1.841
1.823
1.803
1.779
1.772
1.766
1.580
1.575
1.566
1.560
1.542
1.524
-0.000
O
O
O
(1H NMR, 400 MHz, CDCl3)
1.06
0.98
1.04
1.92
2.00
1.98
5.08
1.96
f1 (ppm)

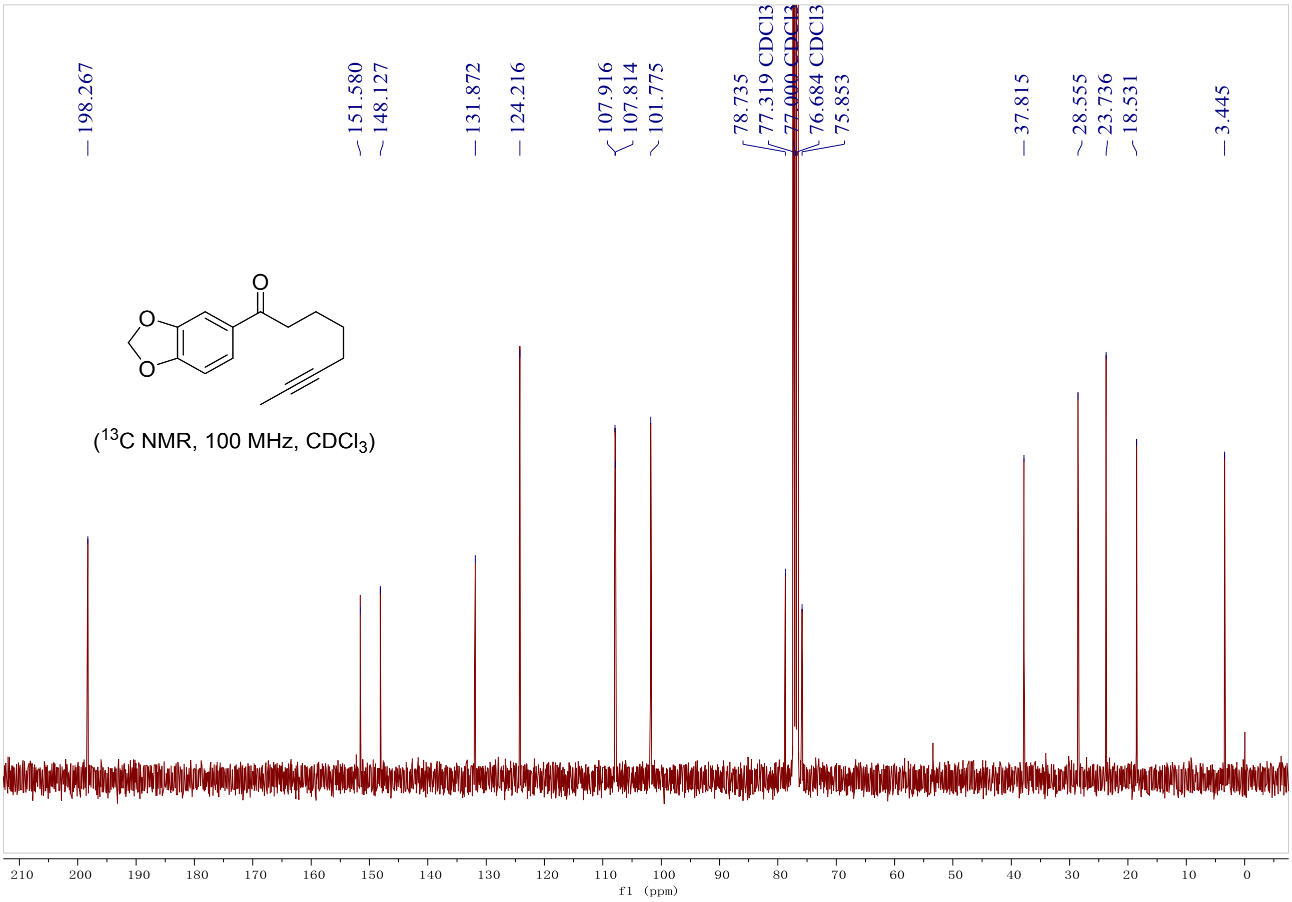
198.267
151.580
148.127
131.872
124.216
107.916
107.814
101.775
78.735
77.319 CDCl3
77.000 CDCl3
76.684 CDCl3
75.853
37.815
28.555
23.736
18.531
3.445
O
O
O
(13C NMR, 100 MHz, CDCl3)
f1 (ppm)

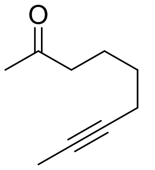

Non-7-yn-2-one (3bj): colorless oil, 12 mg, 43%, Eluent: PE/EA = 20/1.

^{1}H NMR (400 MHz, $CDCl_3$, TMS): δ 2.38 (t, *J* = 7.4 Hz, 2H), 2.13-2.01 (m, 5H), 1.71 (s, 3H), 1.65-1.55 (m, 2H), 1.47-1.34 (m, 2H). **^{13}C NMR** (150MHz, $CDCl_3$, TMS): δ 3.3, 18.4, 22.9, 28.3, 29.7, 43.1, 75.7, 78.6, 208.8. **IR** (EtOH): ν 2973, 2881, 2872, 2209, 1710, 1399, 1380, 1087, 1045, 880 cm^{-1}. **HRMS** (DART) m/z: $[M+H]^+$ Calcd. for $C_9H_{15}O$ 139.1117; found 139.1119.

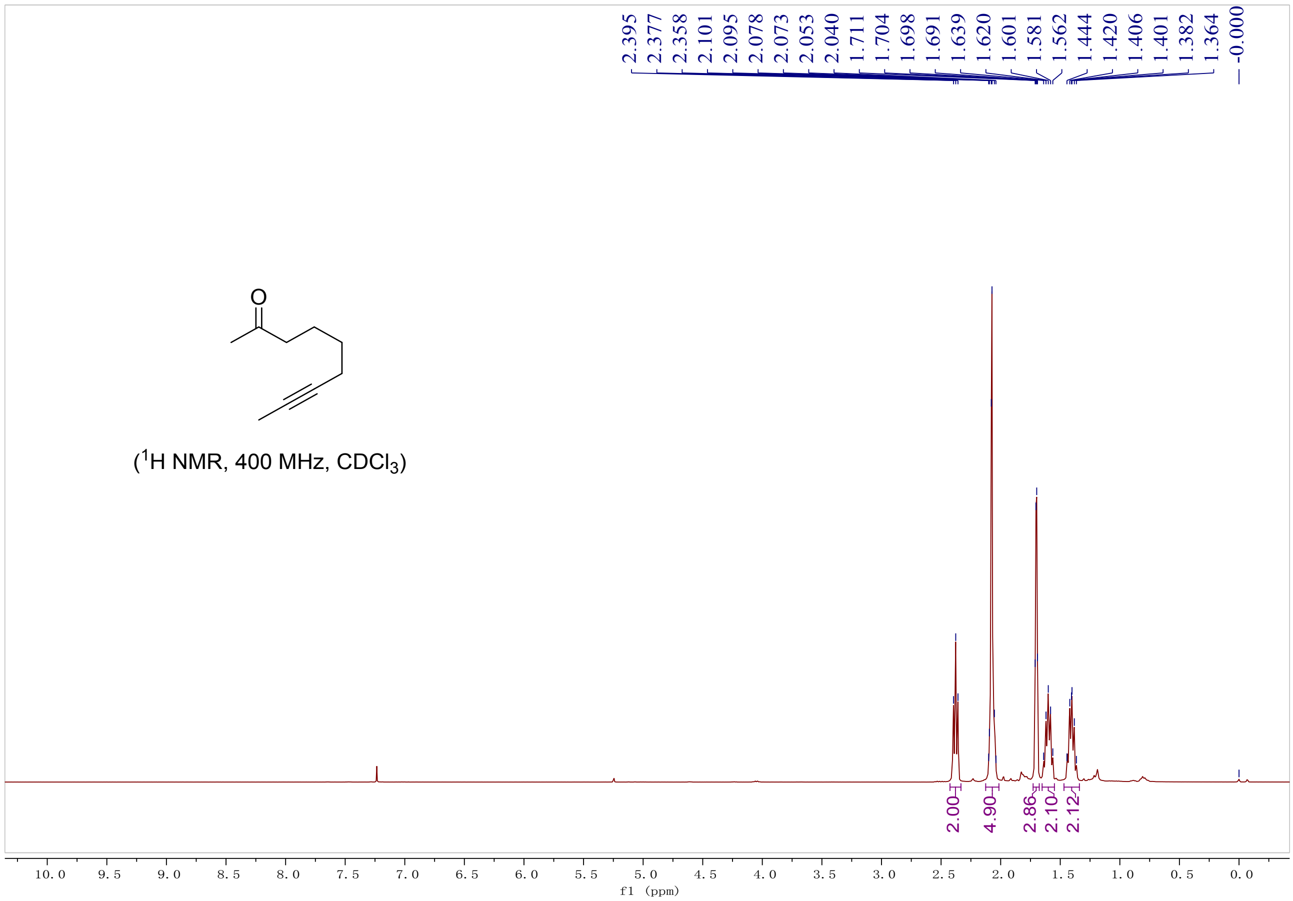
2.395 2.377 2.358 2.101 2.095 2.078 2.073 2.053 2.040 1.711 1.704 1.698 1.691 1.639 1.620 1.601 1.581 1.562 1.444 1.420 1.406 1.401 1.382 1.364 -0.000
O
(1H NMR, 400 MHz, CDCl3)
2.00 4.90 2.86 2.10 2.12
10.0 9.5 9.0 8.5 8.0 7.5 7.0 6.5 6.0 5.5 5.0 4.5 4.0 3.5 3.0 2.5 2.0 1.5 1.0 0.5 0.0
f1 (ppm)

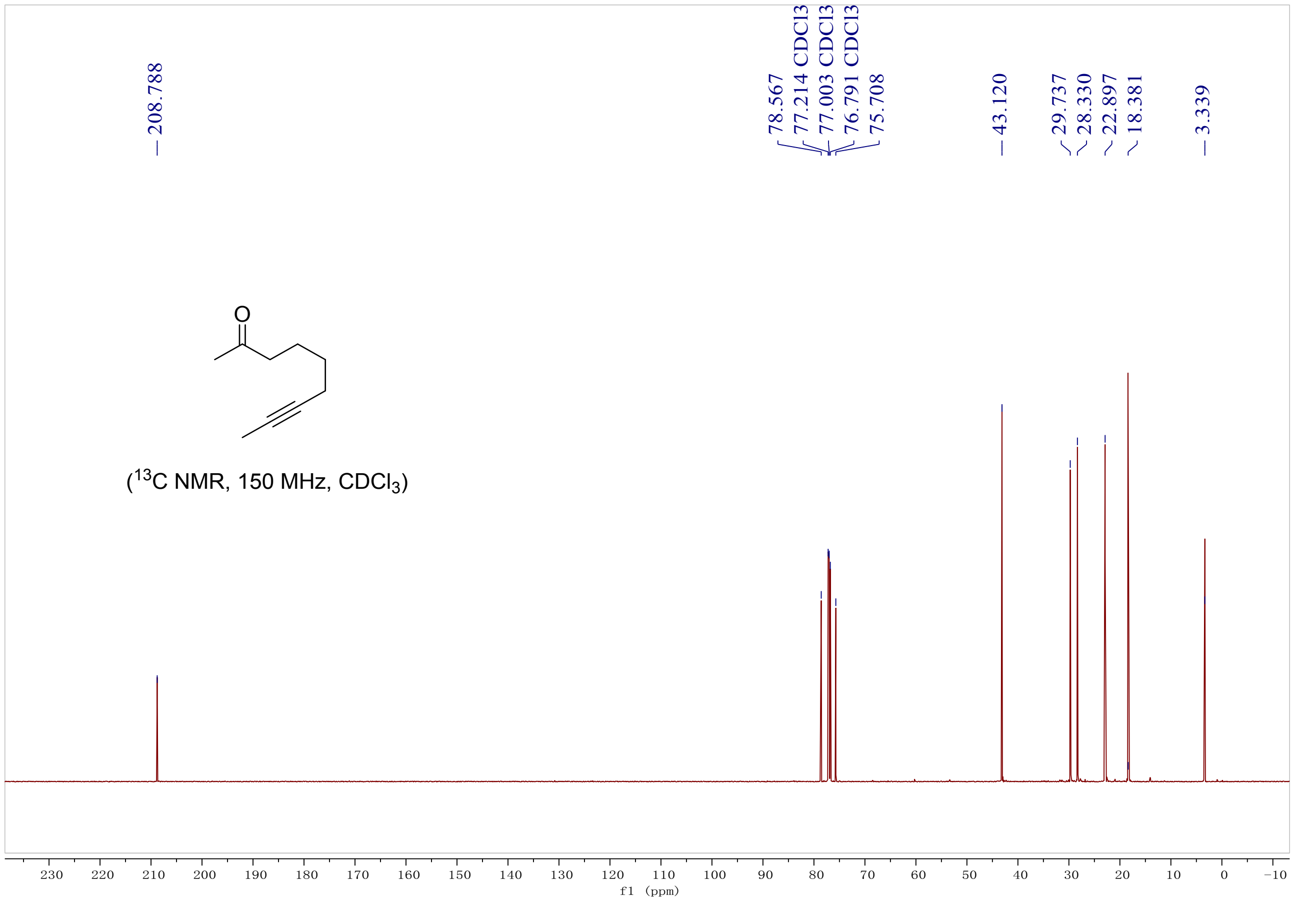
208.788
78.567 77.214 CDCl3 77.003 CDCl3 76.791 CDCl3 75.708
43.120
29.737 28.330 22.897 18.381
3.339
O
(13C NMR, 150 MHz, CDCl3)
230 220 210 200 190 180 170 160 150 140 130 120 110 100 90 80 70 60 50 40 30 20 10 0 -10
f1 (ppm)

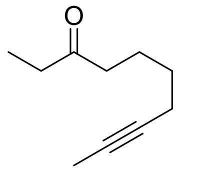

Dec-8-yn-3-one (3bk): colorless oil, 12 mg, 40%, Eluent: PE/EA = 20/1.

^{1}H NMR (600 MHz, $CDCl_3$, TMS): δ 2.42-2.36 (m, 4H), 2.10 (tq, J = 7.2, 2.6 Hz, 2H), 1.73 (t, J = 2.6 Hz, 3H), 1.65-1.60 (m, 2H), 1.47-1.39 (m, 2H), 1.01 (t, J = 7.2 Hz, 3H). **^{13}C NMR** (150 MHz, $CDCl_3$, TMS): δ 3.4, 7.7, 18.5, 23.0, 28.5, 35.8, 41.8, 75.7, 78.6, 211.5. **IR** (EtOH): ν 2950, 2938, 2859, 2199, 1713, 1458, 1375, 1114, 899 cm^{-1}. **HRMS** (DART) m/z: $[M+H]^+$ Calcd. for $C_{10}H_{17}O$ 153.1274; found 153.1274.

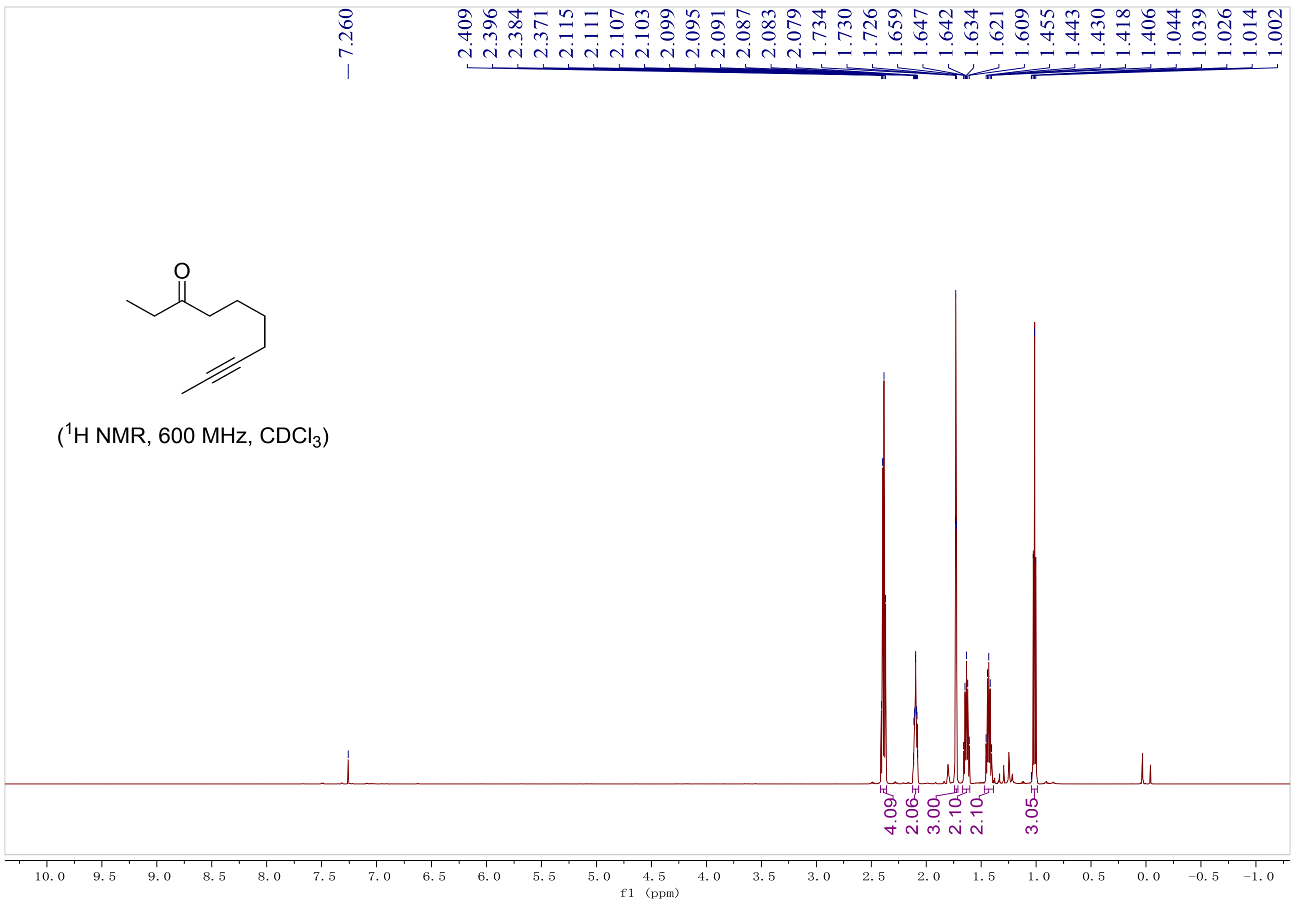
7.260
2.409
2.396
2.384
2.371
2.115
2.111
2.107
2.103
2.099
2.095
2.091
2.087
2.083
2.079
1.734
1.730
1.726
1.659
1.647
1.642
1.634
1.621
1.609
1.455
1.443
1.430
1.418
1.406
1.044
1.039
1.026
1.014
1.002
O
(1H NMR, 600 MHz, CDCl3)
4.09
2.06
3.00
2.10
2.10
3.05
f1 (ppm)

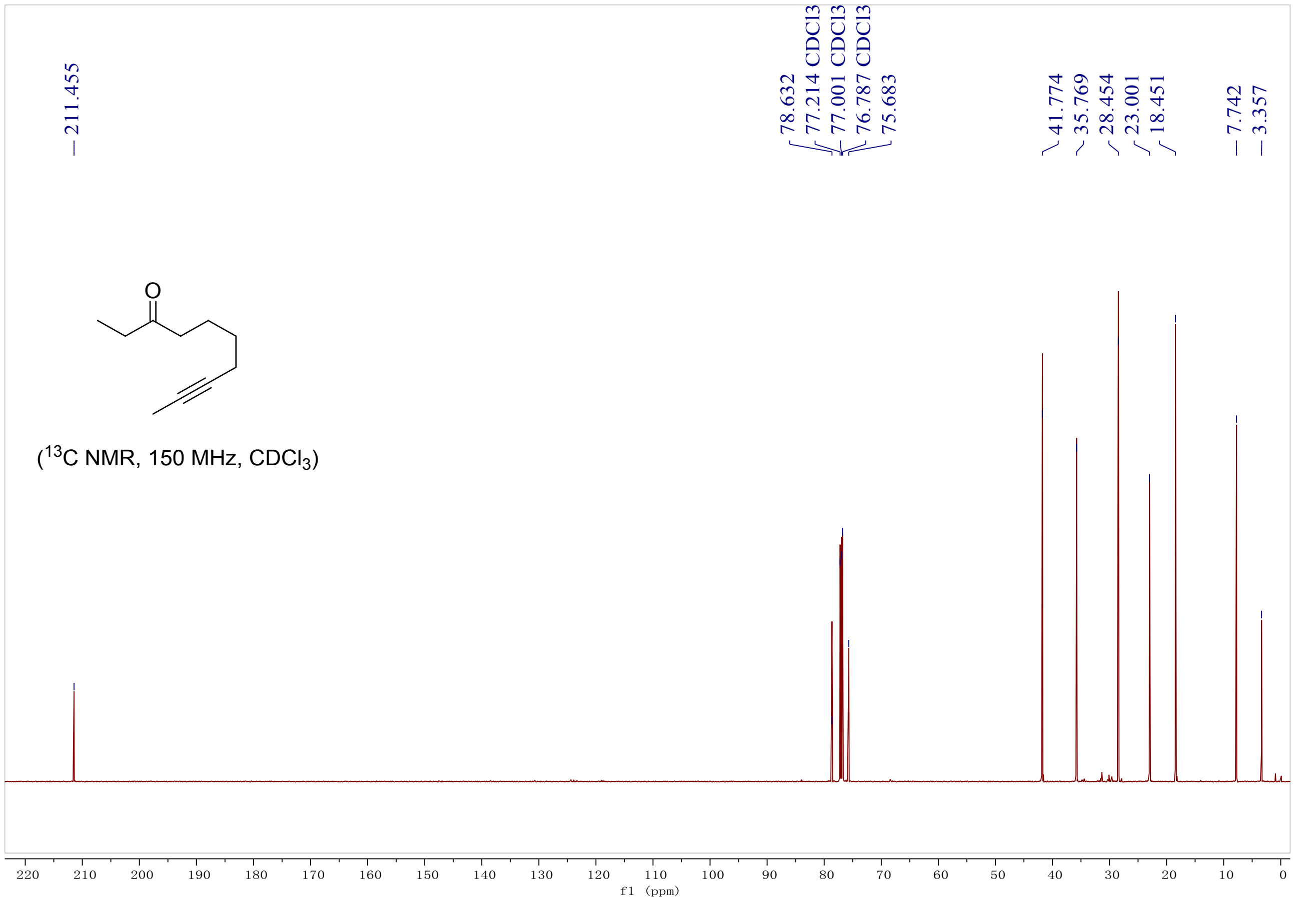
211.455
78.632
77.214 CDCl3
77.001 CDCl3
76.787 CDCl3
75.683
41.774
35.769
28.454
23.001
18.451
7.742
3.357
O
(13C NMR, 150 MHz, CDCl3)
f1 (ppm)

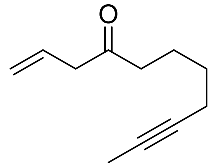

Undec-1-en-9-yn-4-one (3bl): colorless oil, 13 mg, 38%, Eluent: PE/EA = 20/1.

^{1}H NMR (400 MHz, $CDCl_3$, TMS): δ 5.91 (ddt, *J* = 17.2, 10.2, 7.0 Hz, 1H), 5.22-5.08 (m, 2H), 3.17 (d, *J* = 7.0 Hz, 2H), 2.45 (t, *J* = 7.4 Hz, 2H), 2.13 (tq, *J* = 7.2, 2.6 Hz, 2H), 1.76 (t, *J* = 2.6 Hz, 3H), 1.73-1.62 (m, 2H), 1.50-1.42 (m, 2H). **^{13}C NMR** (150 MHz, $CDCl_3$, TMS): δ 3.4, 18.5, 22.8, 28.4, 41.8, 47.7, 75.8, 78.7, 118.8, 130.6, 208.6. **IR** (EtOH): ν 2954, 2920, 2853, 2223, 1709, 1459, 1377, 1260, 1079, 1018, 967, 797 cm^{-1}. **HRMS** (DART) m/z: $[M+H]^+$ Calcd. for $C_{11}H_{17}O$ 165.1274; found 165.1275.

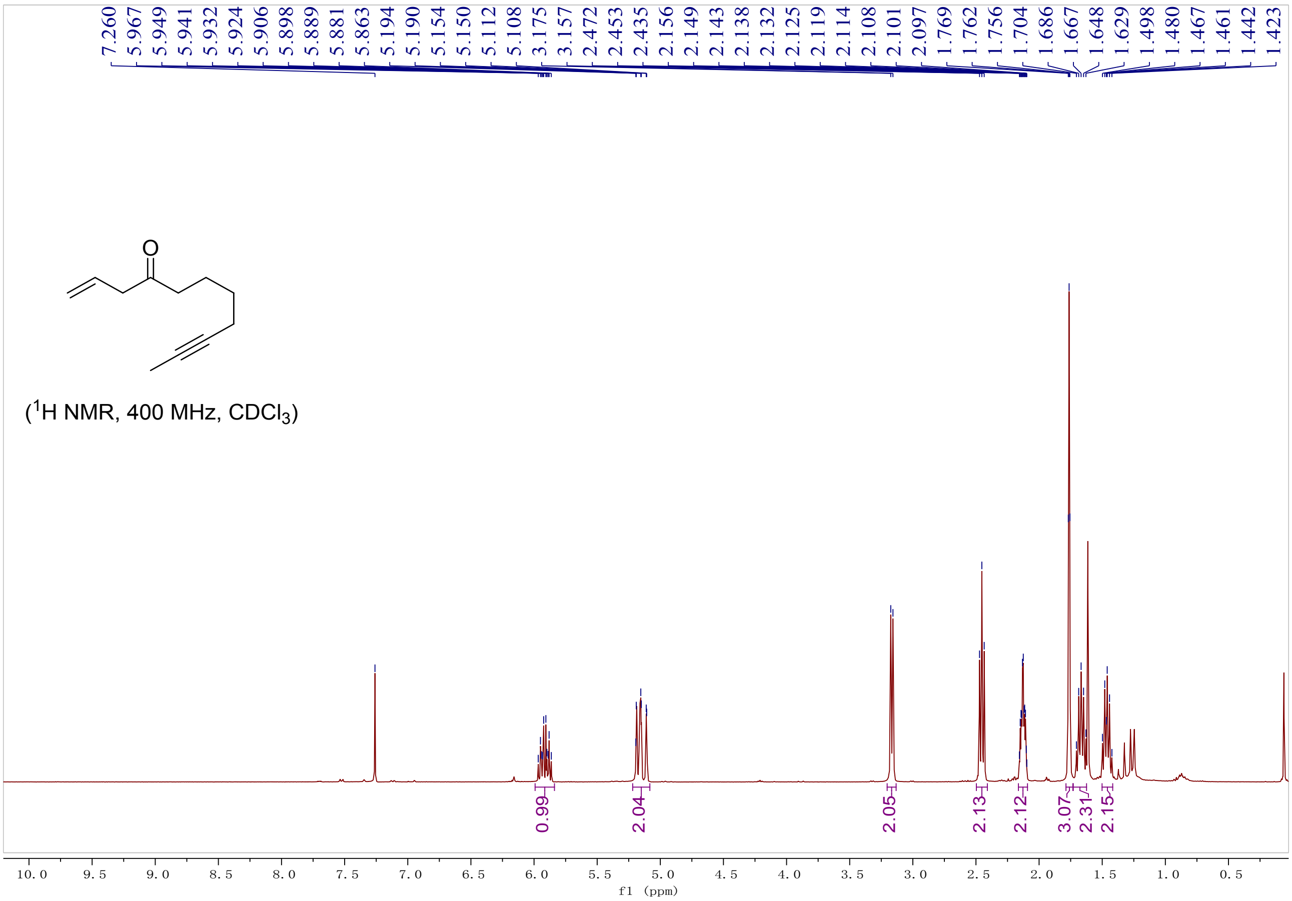
(1H NMR, 400 MHz, CDCl3)
f1 (ppm)

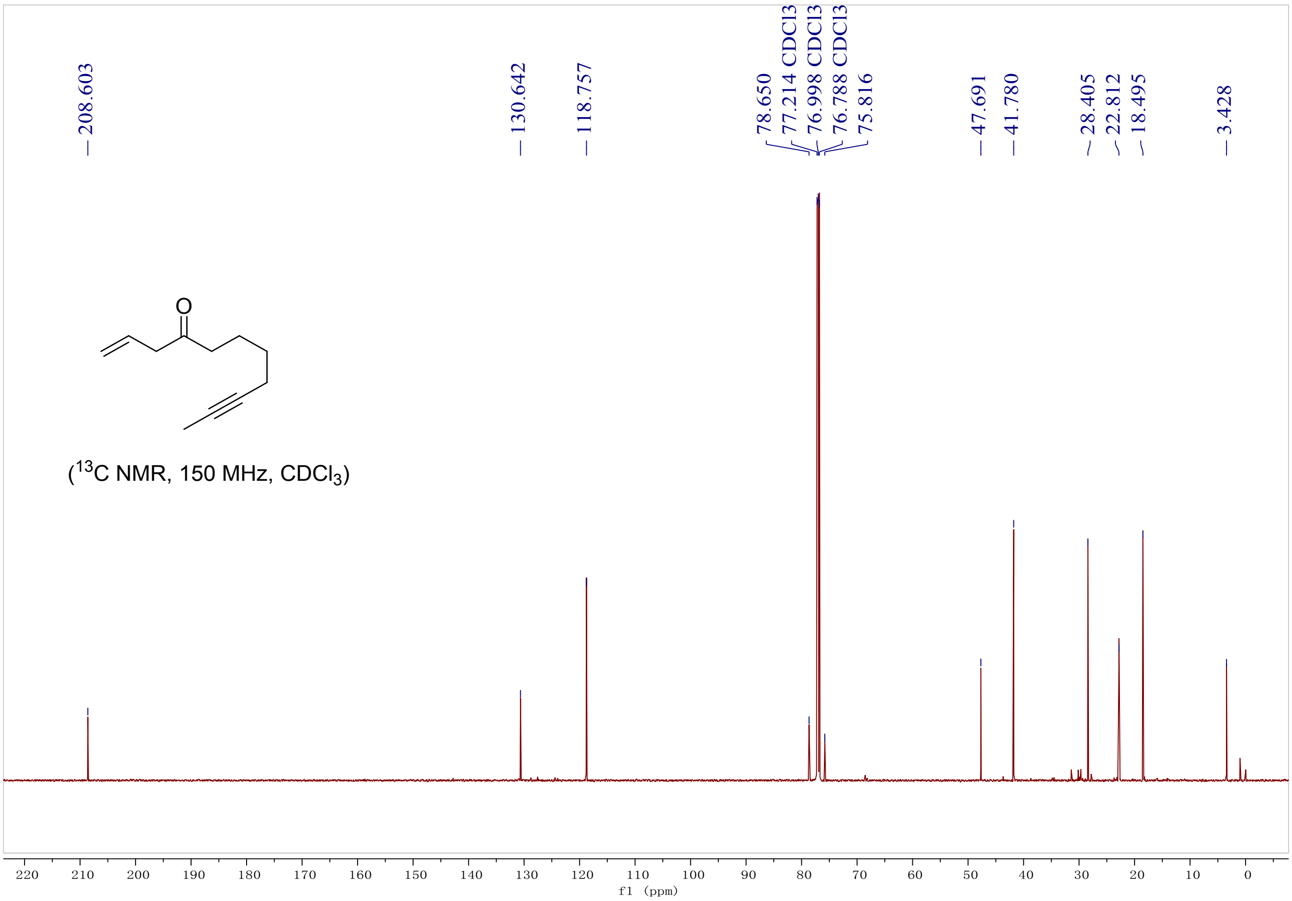
(13C NMR, 150 MHz, CDCl3)
f1 (ppm)

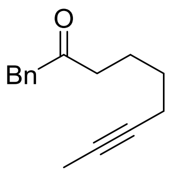

1-phenylnon-7-yn-2-one (3bm): colorless oil, 15 mg, 36%, Eluent: PE/EA = 20/1.

^{1}H NMR (400 MHz, $CDCl_3$, TMS): δ 7.35-7.30 (m, 2H), 7.29-7.23 (m, 1H), 7.22-7.16 (m, 2H), 3.68 (s, 2H), 2.46 (t, J = 7.4 Hz, 2H), 2.09 (tq, J = 7.2, 2.6 Hz, 2H), 1.76 (t, J = 2.6 Hz, 3H), 1.69-1.61 (m, 2H), 1.46-1.37 (m, 2H). **^{13}C NMR** (100 MHz, $CDCl_3$, TMS): δ 3.4, 18.4, 22.8, 28.3, 41.4, 50.1, 75.8, 78.7, 127.0, 128.7, 129.4, 134.3, 208.2. **IR** (EtOH): ν 2972, 2939, 2855, 2218, 1713, 1458, 1354, 1114, 1005, 955, 845, 767, 699 cm^{-1}. **HRMS** (DART) m/z: $[M+H]^+$ Calcd. for $C_{15}H_{19}O$ 215.1430; found 215.1430.

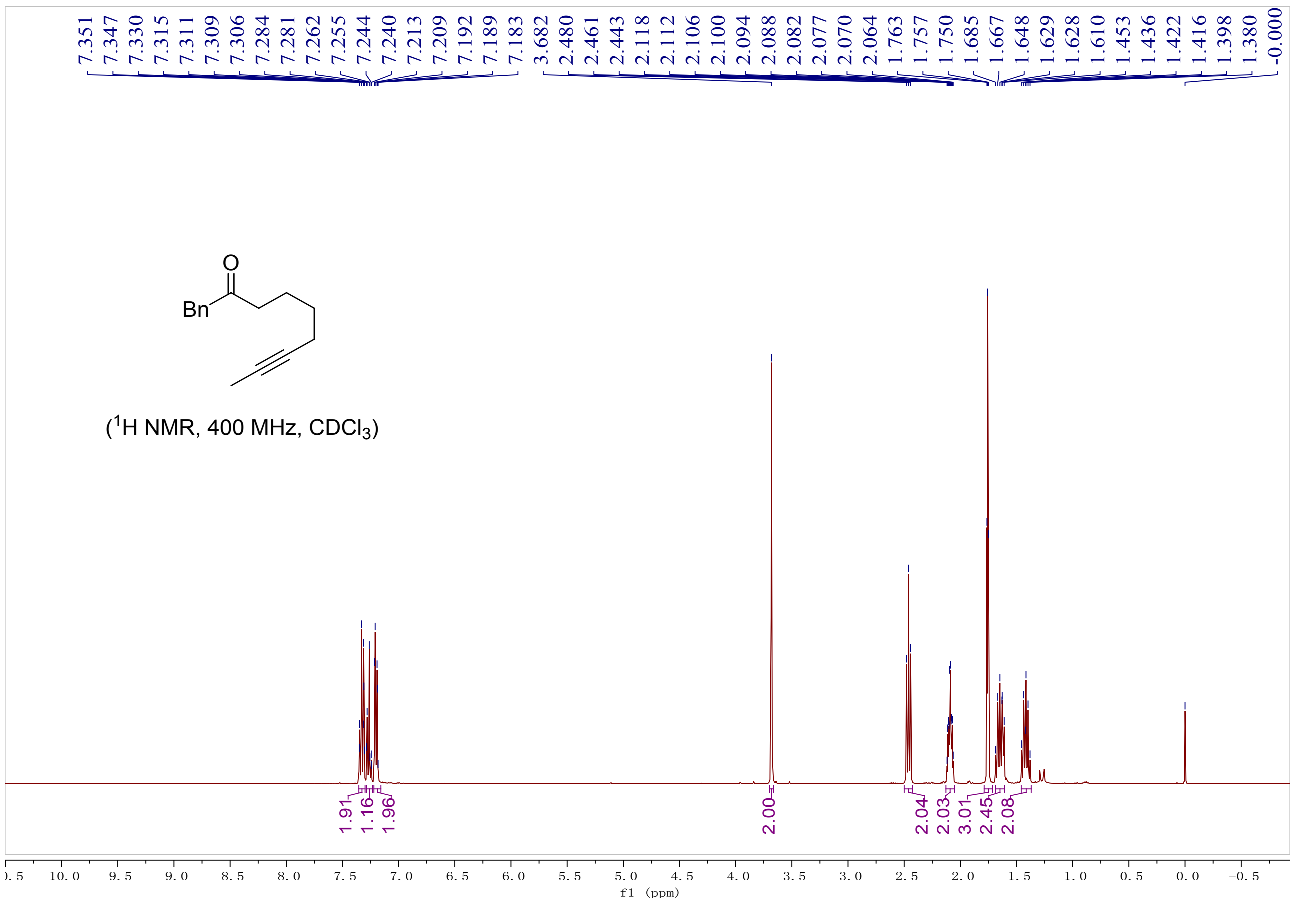
7.351 7.347 7.330 7.315 7.311 7.309 7.306 7.284 7.281 7.262 7.255 7.244 7.240 7.213 7.209 7.192 7.189 7.183 3.682 2.480 2.461 2.443 2.118 2.112 2.106 2.100 2.094 2.088 2.082 2.077 2.070 2.064 1.763 1.757 1.750 1.685 1.667 1.648 1.629 1.628 1.610 1.453 1.436 1.422 1.416 1.398 1.380 -0.000
O
Bn
(¹H NMR, 400 MHz, CDCl3)
1.91 1.16 1.96 2.00 2.04 2.03 3.01 2.45 2.08
f1 (ppm)
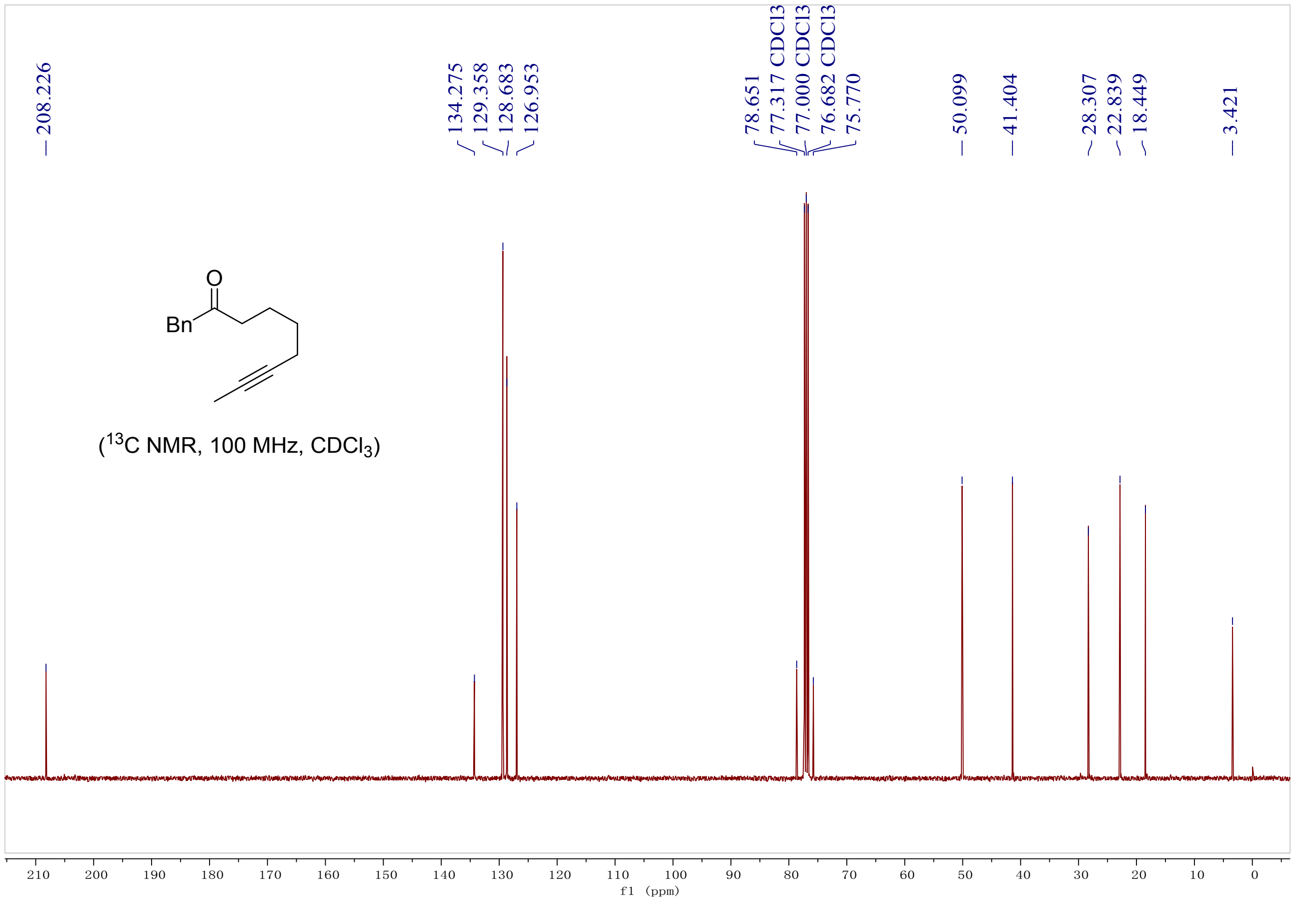
208.226
134.275 129.358 128.683 126.953
78.651 77.317 CDCl3 77.000 CDCl3 76.682 CDCl3 75.770
50.099 41.404 28.307 22.839 18.449 3.421
O
Bn
(¹³C NMR, 100 MHz, CDCl3)
f1 (ppm)

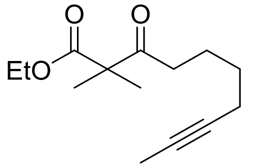

Ethyl 2,2-dimethyl-3-oxodec-8-ynoate (3bn): colorless oil, 19 mg, 40%, Eluent: PE/EA = 20/1. **^{1}H NMR** (400 MHz, $CDCl_3$, TMS): δ 4.11 (q, J = 7.2 Hz, 2H), 2.40 (t, J = 7.2 Hz, 2H), 2.05 (tq, J = 7.2, 2.6 Hz, 2H), 1.68 (t, J = 2.6 Hz, 3H), 1.64-1.55 (m, 2H), 1.44-1.33 (m, 2H), 1.28 (s, 6H), 1.19 (t, J = 7.2 Hz, 3H). **^{13}C NMR** (100 MHz, $CDCl_3$, TMS): δ 3.2, 13.8, 18.3, 21.6, 22.9, 28.2, 37.2, 55.3, 61.0, 75.5, 78.4, 173.4, 207.5. **IR** (EtOH): ν 2980, 2938, 2865, 2216, 1713, 1640, 1459, 1375, 1114 cm^{-1}. **HRMS** (DART) m/z: $[M+H]^+$ Calcd. for $C_{14}H_{23}O_3$ 239.1642; found 239.1641.

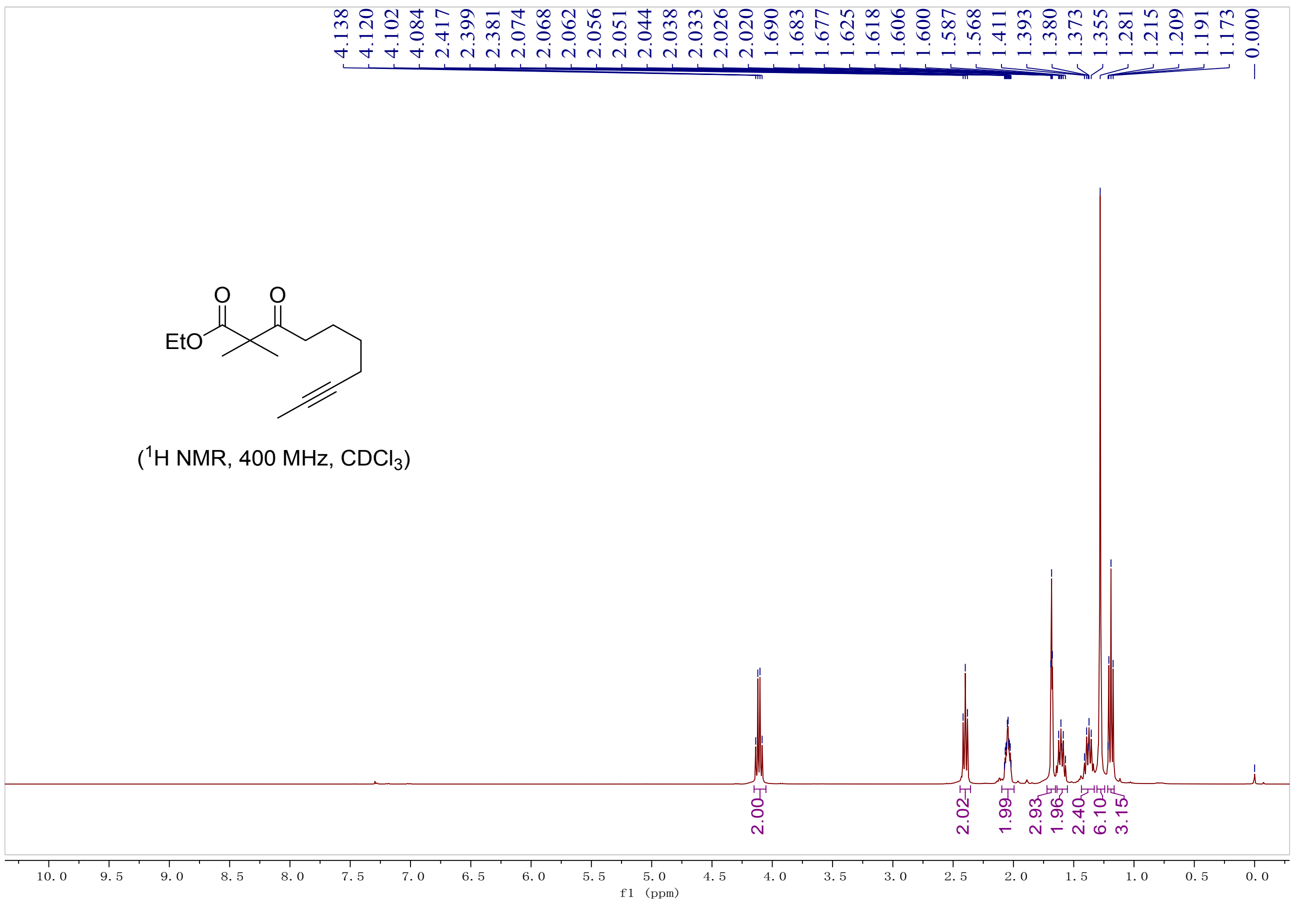
4.138
4.120
4.102
4.084
2.417
2.399
2.381
2.074
2.068
2.062
2.056
2.051
2.044
2.038
2.033
2.026
2.020
1.690
1.683
1.677
1.625
1.618
1.606
1.600
1.587
1.568
1.411
1.393
1.380
1.373
1.355
1.281
1.215
1.209
1.191
1.173
0.000
EtO
O
O
(^{1}H NMR, 400 MHz, $CDCl_3$)
2.00
2.02
1.99
2.93
1.96
2.40
6.10
3.15
f1 (ppm)

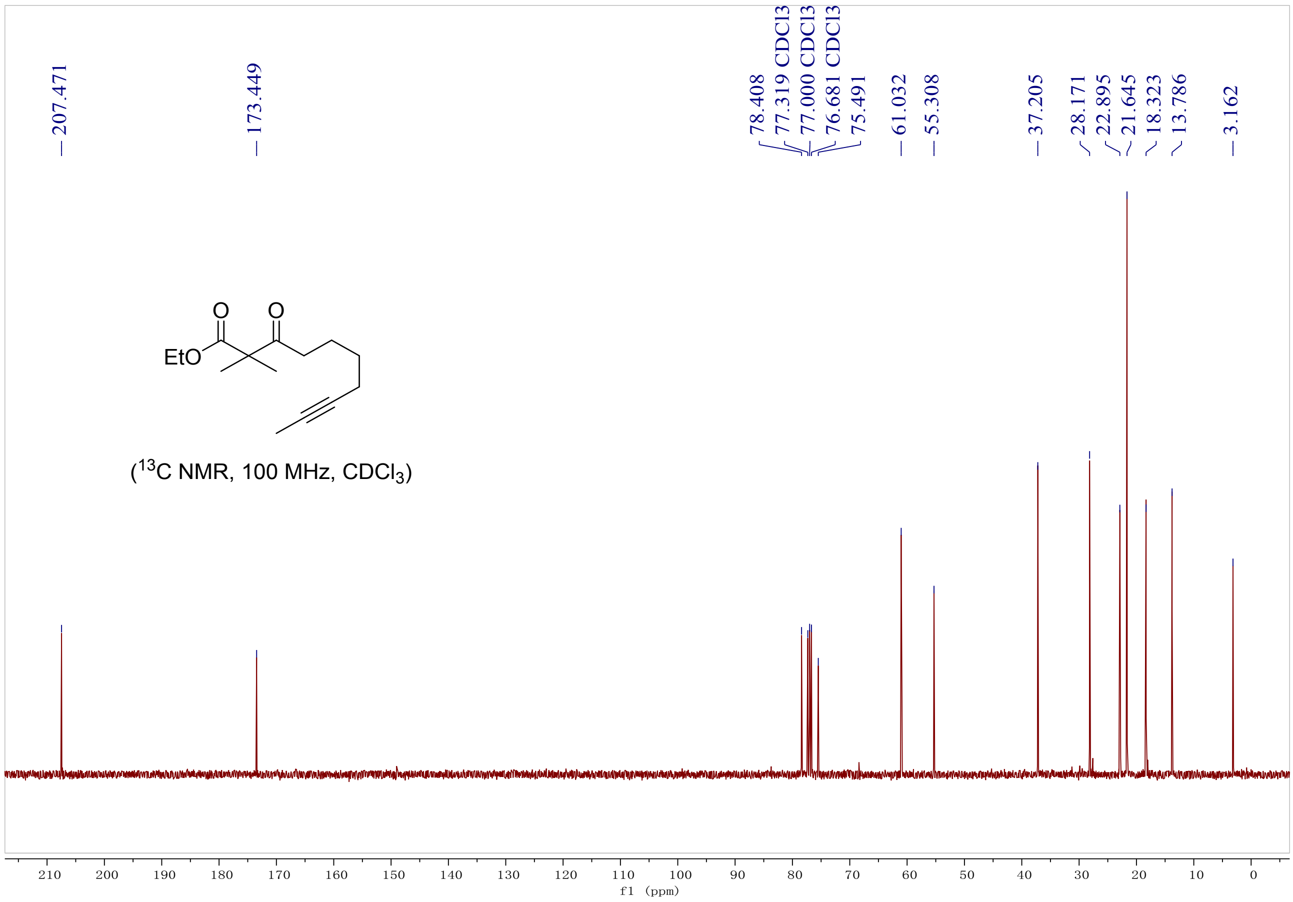
207.471
173.449
78.408
77.319 CDCl3
77.000 CDCl3
76.681 CDCl3
75.491
61.032
55.308
37.205
28.171
22.895
21.645
18.323
13.786
3.162
EtO
O
O
(^{13}C NMR, 100 MHz, $CDCl_3$)
f1 (ppm)

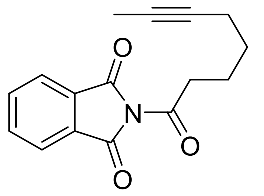

2-(oct-6-ynoyl)isoindoline-1,3-dione (3bo): colorless oil, 17 mg, 33%, Eluent: PE/EA = 5/1.

^{1}H NMR (600 MHz, $CDCl_3$, TMS): δ 7.89 (dd, *J* = 5.4, 3.1 Hz, 2H), 7.79 (dd, *J* = 5.4, 3.1 Hz, 2H), 2.70 (t, *J* = 7.5 Hz, 2H), 2.24-2.18 (m, 2H), 1.90 (p, *J* = 7.6 Hz, 2H), 1.79 (t, *J* = 2.6 Hz, 3H), 1.66-1.60 (m, 2H). **^{13}C NMR** (150 MHz, $CDCl_3$, TMS): δ 3.5, 23.7, 27.9, 30.5, 76.2, 78.2, 123.9, 128.9, 134.7, 162.0, 169.4. **IR** (EtOH): ν 3128, 2949, 2861, 2216, 1787, 1706, 1607, 1463, 1383, 1176, 1134, 1118, 973, 880, 781, 694 cm^{-1}. **HRMS** (ESI) m/z: $[M+H]^+$ Calcd. for $C_{16}H_{16}NO_3$ 270.1125; found 270.1122.

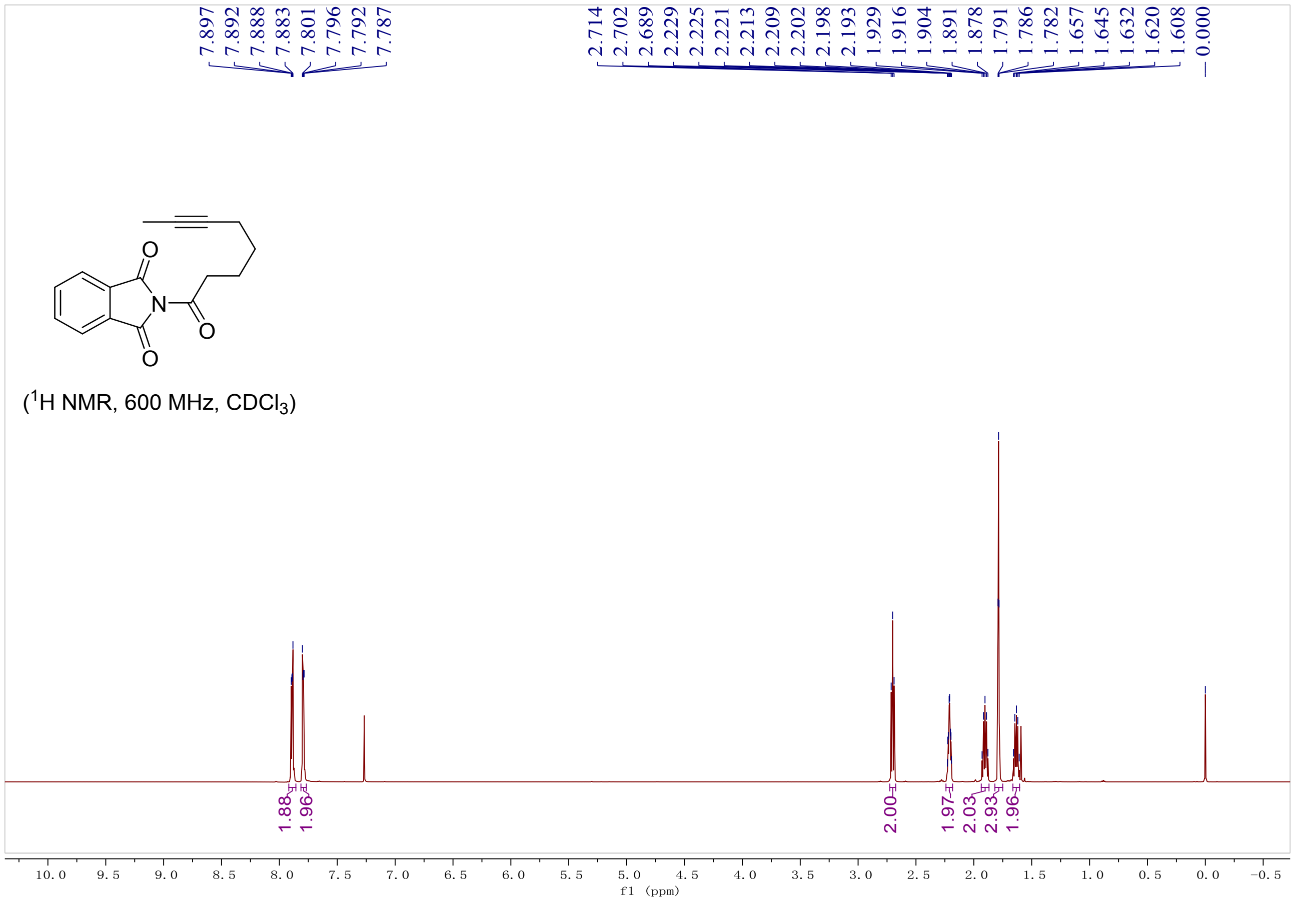

(¹H NMR, 600 MHz, CDCl3)
f1 (ppm)

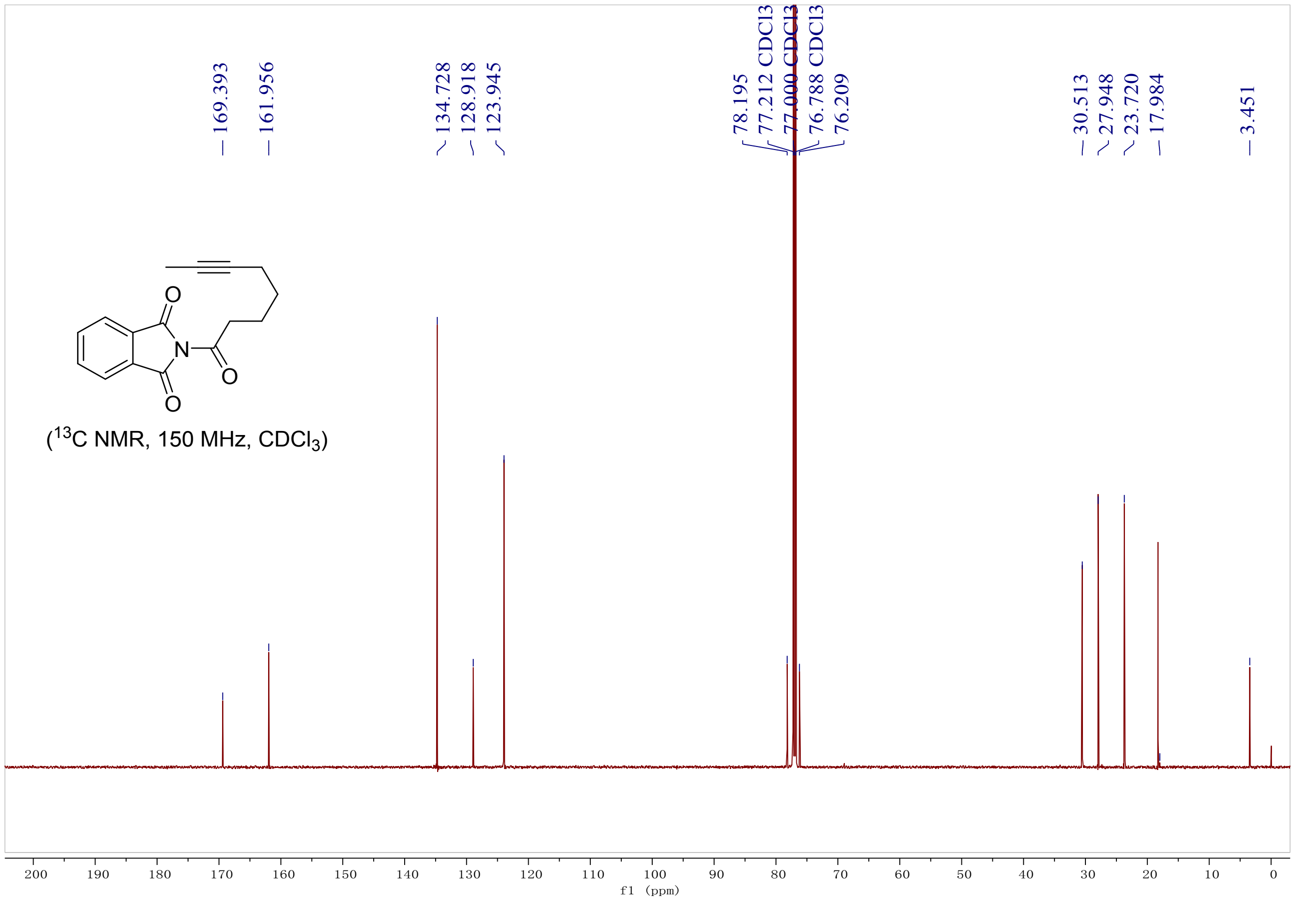

(¹³C NMR, 150 MHz, CDCl3)
f1 (ppm)

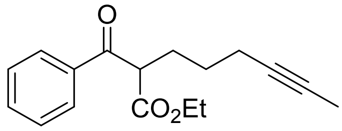

1-phenyloct-6-yn-1-one (3bp): colorless oil, 34 mg, 87%, Eluent: PE/EA = 20/1.

^{1}H NMR (400 MHz, $CDCl_3$, TMS): δ 8.03-7.97 (m, 2H), 7.59 (t, *J* = 7.4 Hz, 1H), 7.52-7.44 (m, 2H), 4.34 (t, *J* = 7.2 Hz, 1H), 4.15 (q, *J* = 7.2 Hz, 2H), 2.19 (tq, *J* = 7.2, 2.4 Hz, 2H), 2.14-2.09 (m, 2H), 1.74 (t, *J* = 2.4 Hz, 3H), 1.58-1.48 (m, 3H), 1.17 (t, *J* = 7.2 Hz, 3H). **^{13}C NMR** (100 MHz, $CDCl_3$, TMS): δ 3.4, 14.0, 18.5, 26.8, 28.1, 53.8, 61.4, 76.2, 78.3, 128.6, 128.7, 133.5, 136.2, 169.9, 195.1. **IR** (EtOH): ν 2975, 2895, 2230, 1648, 1599, 1462, 1381, 1307, 1086, 1044, 879, 739, 693 cm^{-1}. **HRMS** (EI) m/z: $[M]^+$ Calcd. for $C_{17}H_{18}O_3$ 270.1250; found 270.1248.

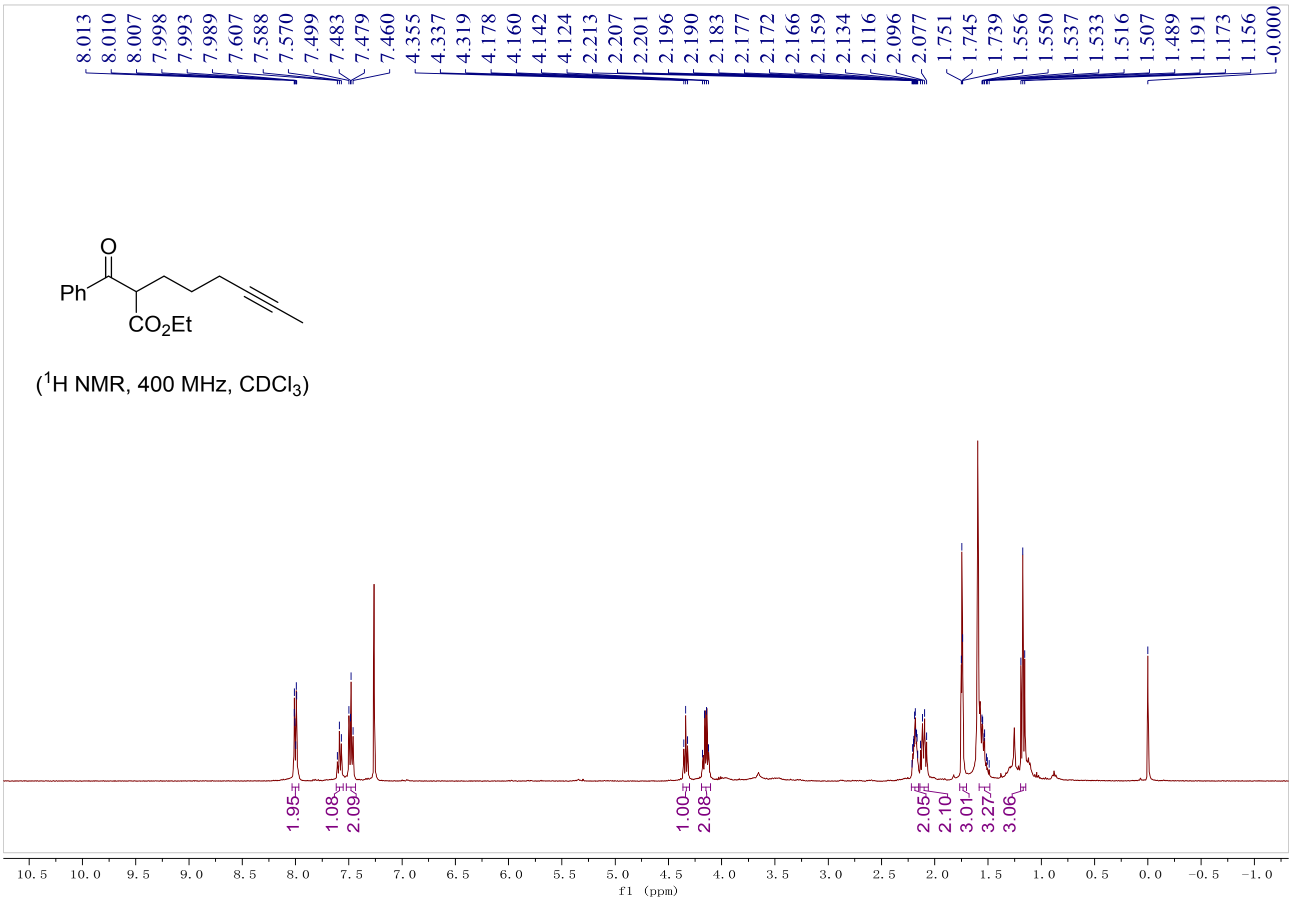
8.013
8.010
8.007
7.998
7.993
7.989
7.607
7.588
7.570
7.499
7.483
7.479
7.460
4.355
4.337
4.319
4.178
4.160
4.142
4.124
2.213
2.207
2.201
2.196
2.190
2.183
2.177
2.172
2.166
2.159
2.134
2.116
2.096
2.077
1.751
1.745
1.739
1.556
1.550
1.537
1.533
1.516
1.507
1.489
1.191
1.173
1.156
-0.000
Ph
O
CO2Et
(1H NMR, 400 MHz, CDCl3)
1.95
1.08
2.09
1.00
2.08
2.05
2.10
3.01
3.27
3.06
f1 (ppm)

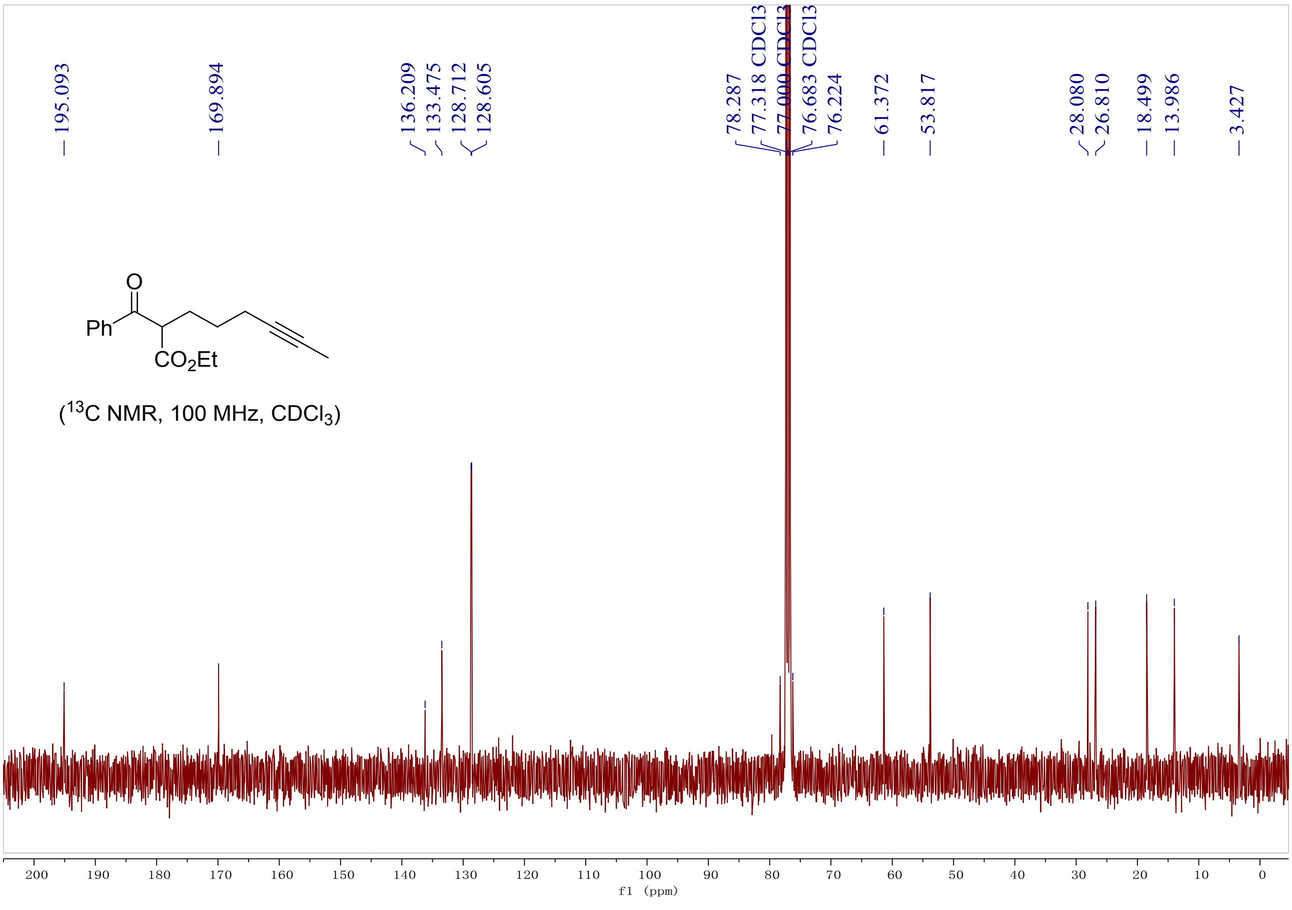
195.093
169.894
136.209
133.475
128.712
128.605
78.287
77.318 CDCl3
77.000 CDCl3
76.683 CDCl3
76.224
61.372
53.817
28.080
26.810
18.499
13.986
3.427
Ph
O
CO2Et
(13C NMR, 100 MHz, CDCl3)
f1 (ppm)

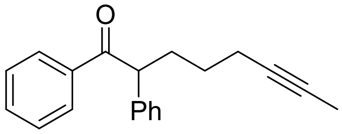

1,2-diphenyloct-6-yn-1-one (3bq): colorless oil, 45 mg, 81%, Eluent: PE/EA = 20/1.

^{1}H NMR (400 MHz, $CDCl_3$, TMS): δ 8.01-7.93 (m, 2H), 7.51-7.44 (m, 1H), 7.41-7.35 (m, 2H), 7.34-7.25 (m, 4H), 7.23-7.17 (m, 1H), 4.58 (t, *J* = 7.2 Hz, 1H), 2.31-2.21 (m, 1H), 2.18-2.10 (m, 2H), 1.99-1.88 (m, 1H), 1.74 (t, *J* = 2.4 Hz, 3H), 1.54-1.37 (m, 2H). **^{13}C NMR** (100 MHz, $CDCl_3$, TMS): δ 3.4, 18.7, 26.9, 33.1, 53.1, 75.9, 78.7, 127.0, 128.2, 128.5, 128.6, 128.9, 132.8, 136.8, 139.5, 199.8. **IR** (EtOH): ν 2976, 2945, 2897, 2211, 1647, 1524, 1472, 1381, 1086, 1044, 1005, 879, 800, 724, 699, 638 cm^{-1}. **HRMS** (EI) m/z: $[M]^+$ Calcd. for $C_{20}H_{20}O$ 276.1509; found 276.1501.

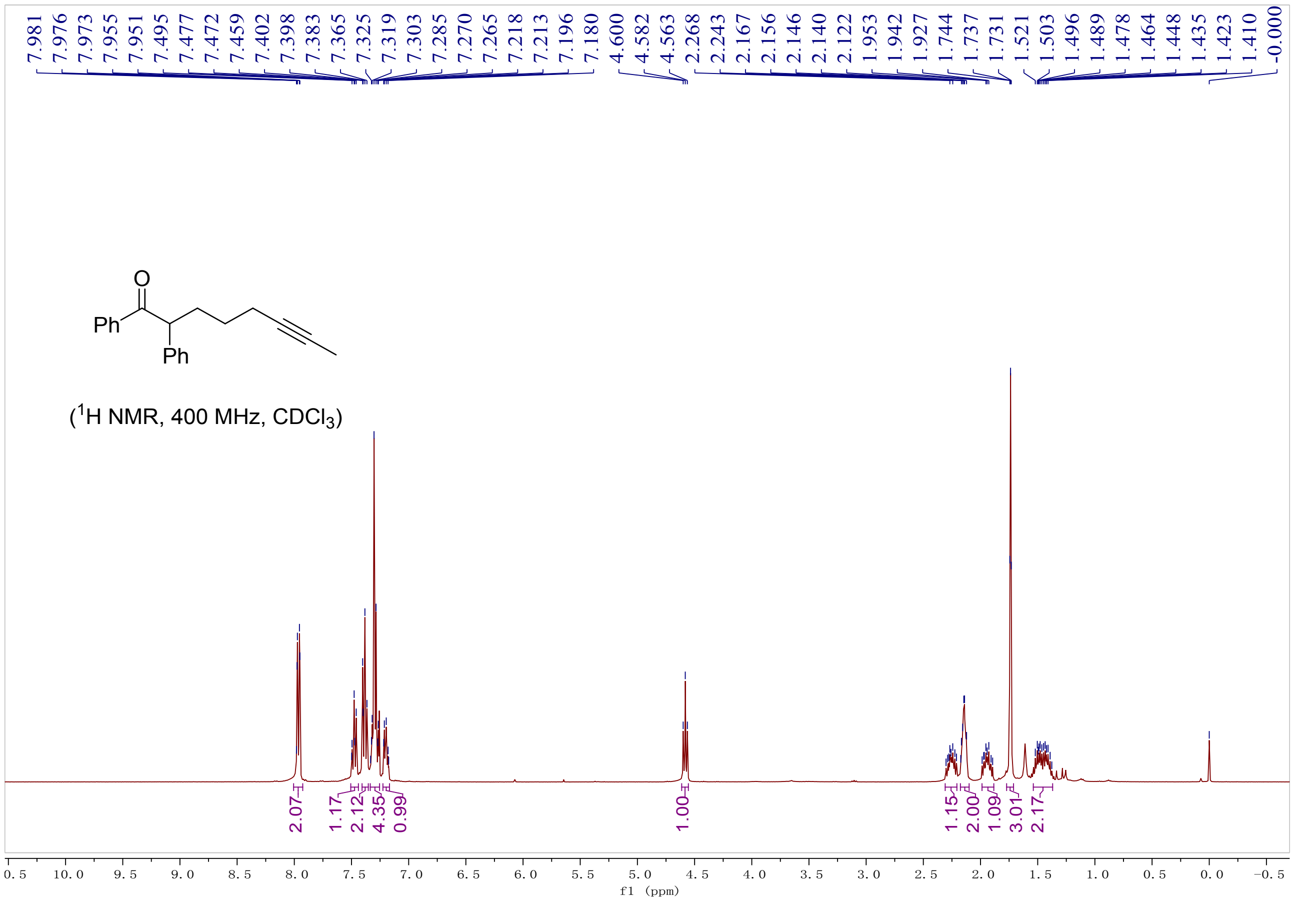
7.981
7.976
7.973
7.955
7.951
7.495
7.477
7.472
7.459
7.402
7.398
7.383
7.365
7.325
7.319
7.303
7.285
7.270
7.265
7.218
7.213
7.196
7.180
4.600
4.582
4.563
2.268
2.243
2.167
2.156
2.146
2.140
2.122
1.953
1.942
1.927
1.744
1.737
1.731
1.521
1.503
1.496
1.489
1.478
1.464
1.448
1.435
1.423
1.410
-0.000
Ph
O
Ph
(1H NMR, 400 MHz, CDCl3)
2.07
1.17
2.12
4.35
0.99
1.00
1.15
2.00
1.09
3.01
2.17
f1 (ppm)

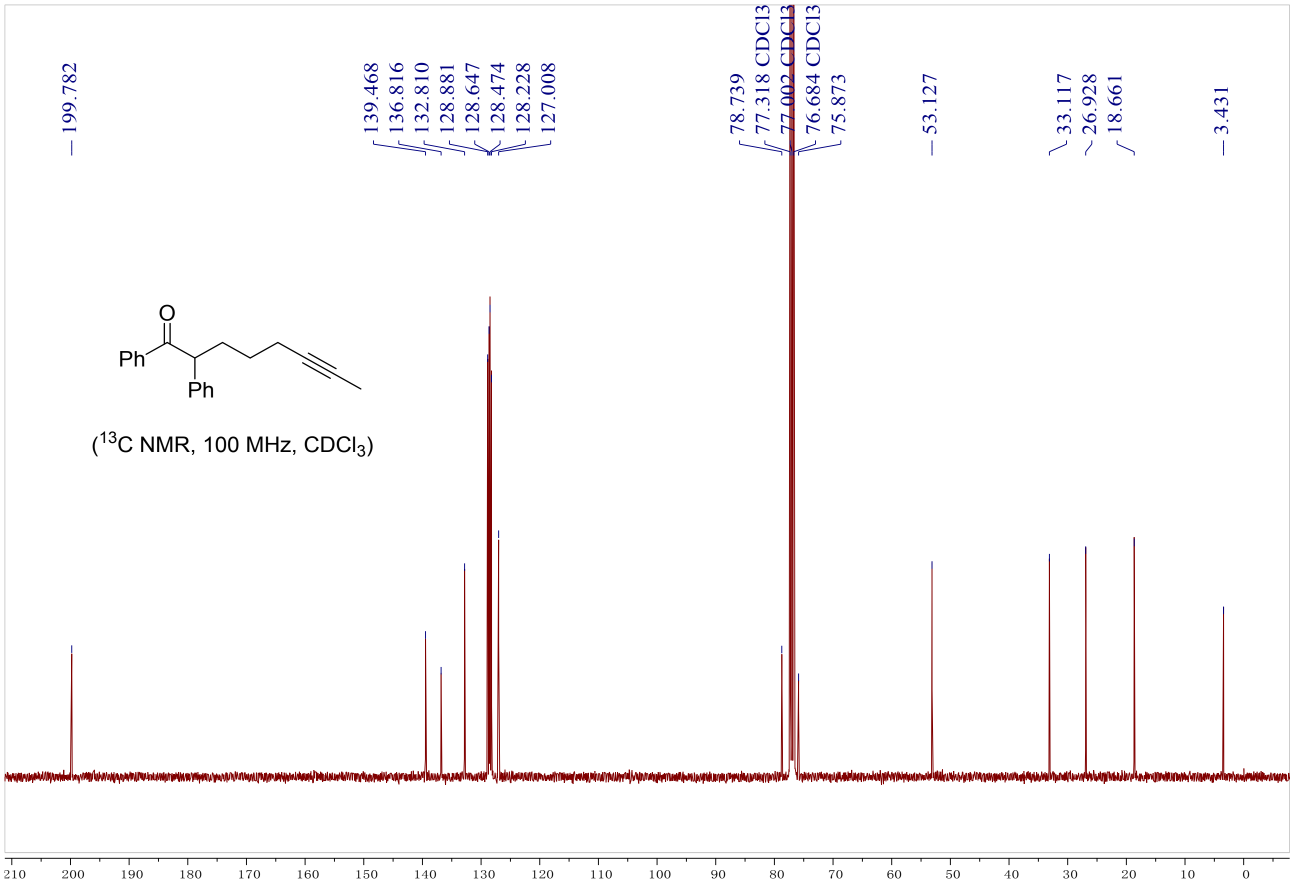
199.782
139.468
136.816
132.810
128.881
128.647
128.474
128.228
127.008
78.739
77.318 CDCl3
77.002 CDCl3
76.684 CDCl3
75.873
53.127
33.117
26.928
18.661
3.431
Ph
O
Ph
(13C NMR, 100 MHz, CDCl3)
f1 (ppm)

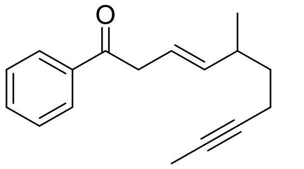

(*E*)-5-Methyl-1-phenyldec-3-en-8-yn-1-one (3br): colorless oil, 44 mg, 92%, Eluent: PE/EA = 20/1. **(instable)**

^{1}H NMR (400 MHz, $CDCl_3$, TMS): δ 7.99-7.95 (m, 2H), 7.59-7.54 (m, 1H), 7.50-7.44 (m, 2H), 5.74-5.62 (m, 1H), 5.44 (dd, *J* = 15.6, 7.8 Hz, 1H), 3.69 (dd, *J* = 6.8, 1.4 Hz, 2H), 2.33-2.24 (m, 1H), 2.13-2.04 (m, 2H), 1.76 (t, *J* = 2.6 Hz, 3H), 1.49-1.42 (m, 2H), 0.99 (d, *J* = 6.8 Hz, 3H). **^{13}C NMR** (100 MHz, $CDCl_3$, TMS): δ 3.5, 16.6, 20.1, 35.9, 36.0, 42.5, 75.5, 79.1, 121.4, 128.3, 128.6, 133.0, 136.6, 139.7, 198.6. **IR** (EtOH): ν 2930, 2921, 2852, 2200, 1666, 1628, 1450, 1272, 1181, 1079, 973, 759, 694 cm^{-1}. **HRMS** (EI) m/z: $[M]^+$ Calcd. for $C_{17}H_{18}O$ 238.1352; found 238.1349.

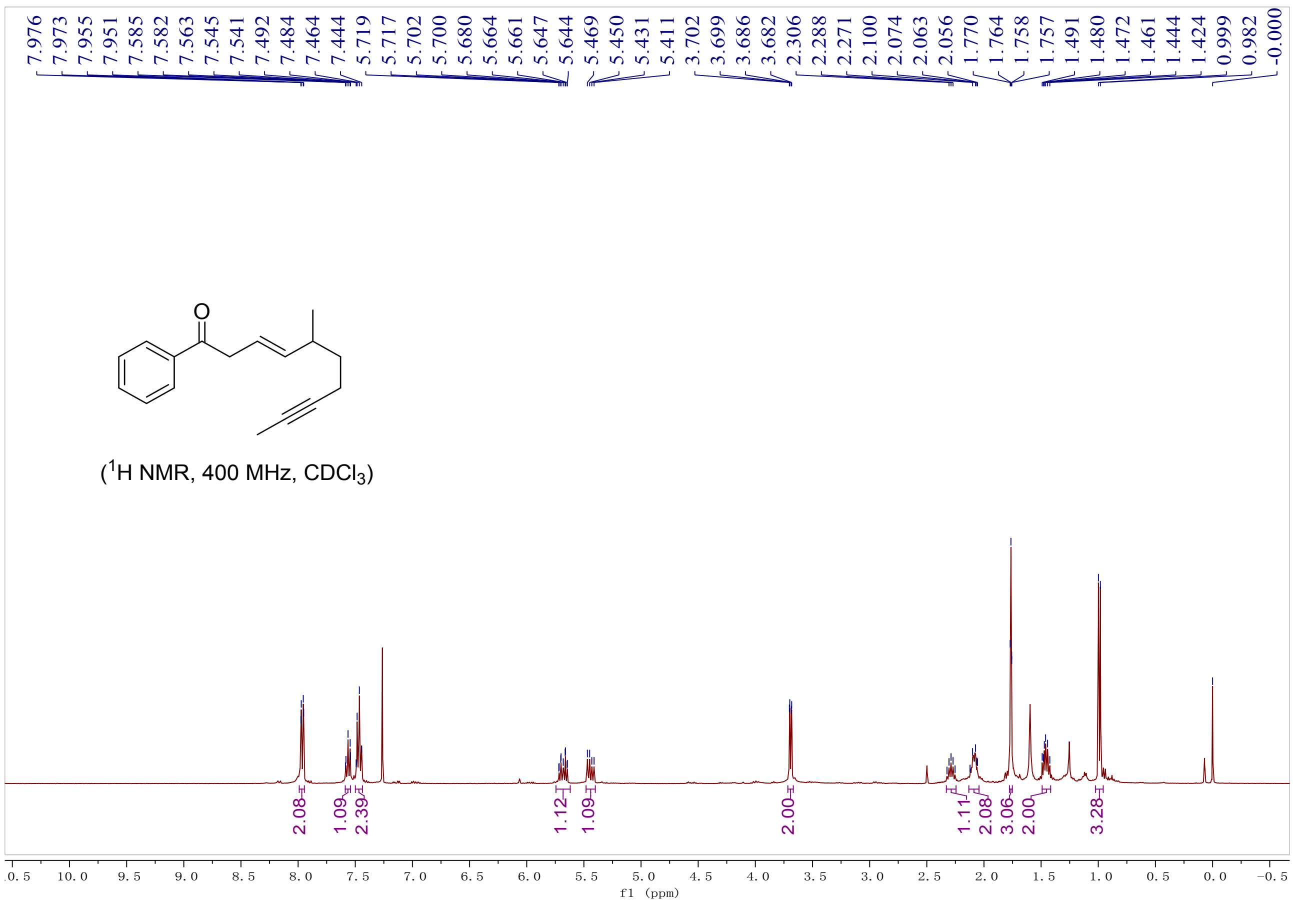
7.976 7.973 7.955 7.951 7.585 7.582 7.563 7.545 7.541 7.492 7.484 7.464 7.444 5.719 5.717 5.702 5.700 5.680 5.664 5.661 5.647 5.644 5.469 5.450 5.431 5.411 3.702 3.699 3.686 3.682 2.306 2.288 2.271 2.100 2.074 2.063 2.056 1.770 1.764 1.758 1.757 1.491 1.480 1.472 1.461 1.444 1.424 0.999 0.982 -0.000
O
(1H NMR, 400 MHz, CDCl3)
2.08 1.09 2.39 1.12 1.09 2.00 1.11 2.08 3.06 2.00 3.28
f1 (ppm)

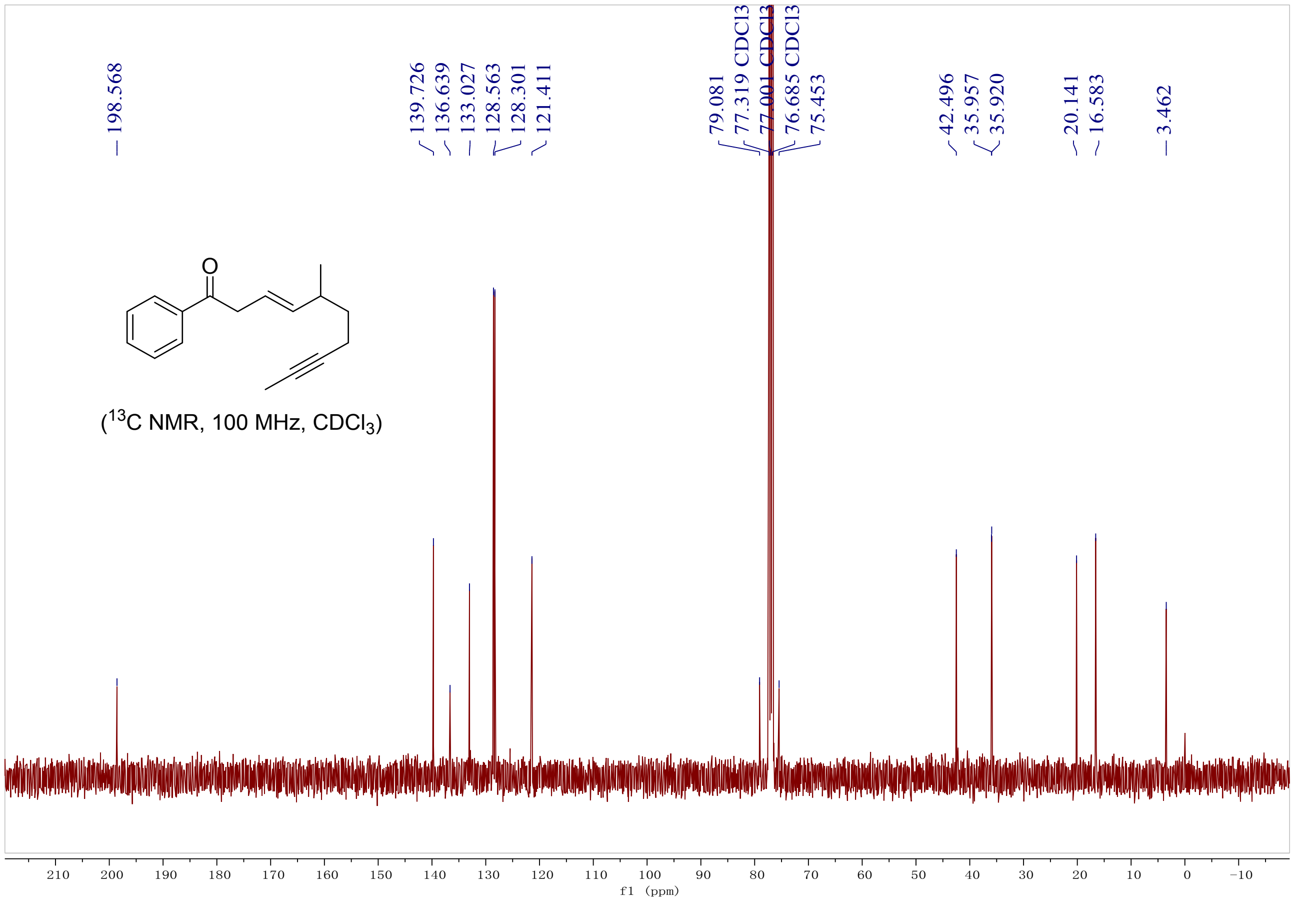
198.568
139.726 136.639 133.027 128.563 128.301 121.411
79.081 77.319 CDCl3 77.001 CDCl3 76.685 CDCl3 75.453
42.496 35.957 35.920
20.141 16.583
3.462
O
(13C NMR, 100 MHz, CDCl3)
f1 (ppm)

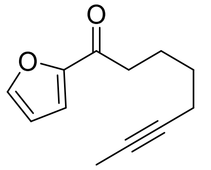

1-(furan-2-yl)oct-6-yn-1-one (3bs): colorless oil, 26 mg, 67%, Eluent: PE/EA = 20/1. **(instable)**

^{1}H NMR (600 MHz, $CDCl_3$, TMS): δ 7.58 (s, 1H), 7.18 (d, *J* = 3.6 Hz, 1H), 6.53 (d, *J* = 3.6 Hz, 1H), 2.84 (t, *J* = 7.6 Hz, 2H), 2.21-2.16 (m, 2H), 1.85-1.75 (m, 5H), 1.61-1.53 (m, 2H). **^{13}C NMR** (150 MHz, $CDCl_3$, TMS): δ 3.4, 18.5, 23.4, 28.5, 37.9, 75.9, 78.7, 112.1, 116.8, 146.2, 152.8, 189.4. **IR** (EtOH): ν 2946, 2920, 2199, 1669, 1524, 1399, 1289, 1203, 1003, 862, 755, 725 cm^{-1}. **HRMS** (ESI) m/z: $[M+Na]^+$ Calcd. for $C_{12}H_{14}O_2Na$ 213.0886; found 213.0889.

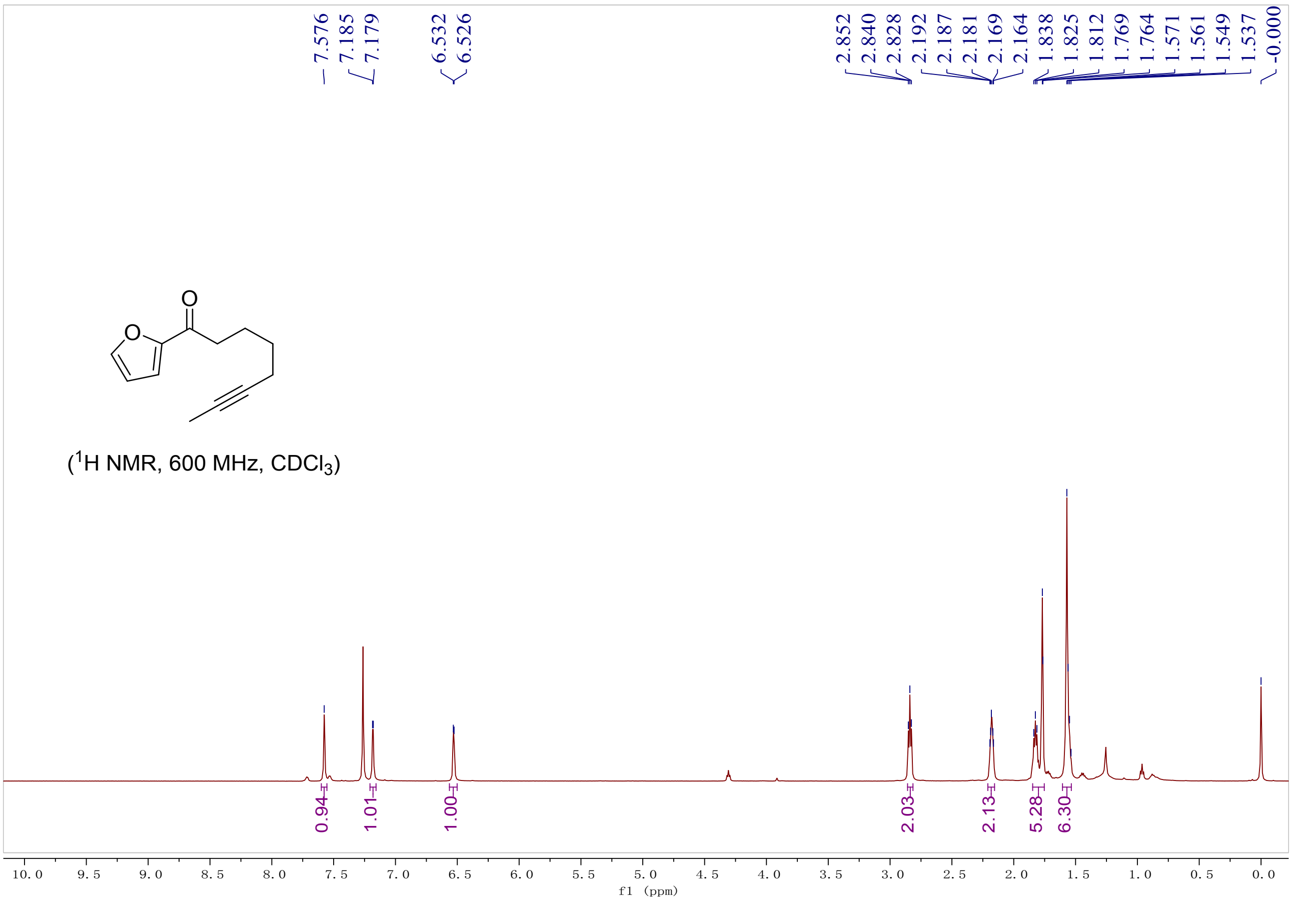
7.576
7.185
7.179
6.532
6.526
2.852
2.840
2.828
2.192
2.187
2.181
2.169
2.164
1.838
1.825
1.812
1.769
1.764
1.571
1.561
1.549
1.537
-0.000
O
O
(1H NMR, 600 MHz, CDCl3)
0.94
1.01
1.00
2.03
2.13
5.28
6.30
f1 (ppm)

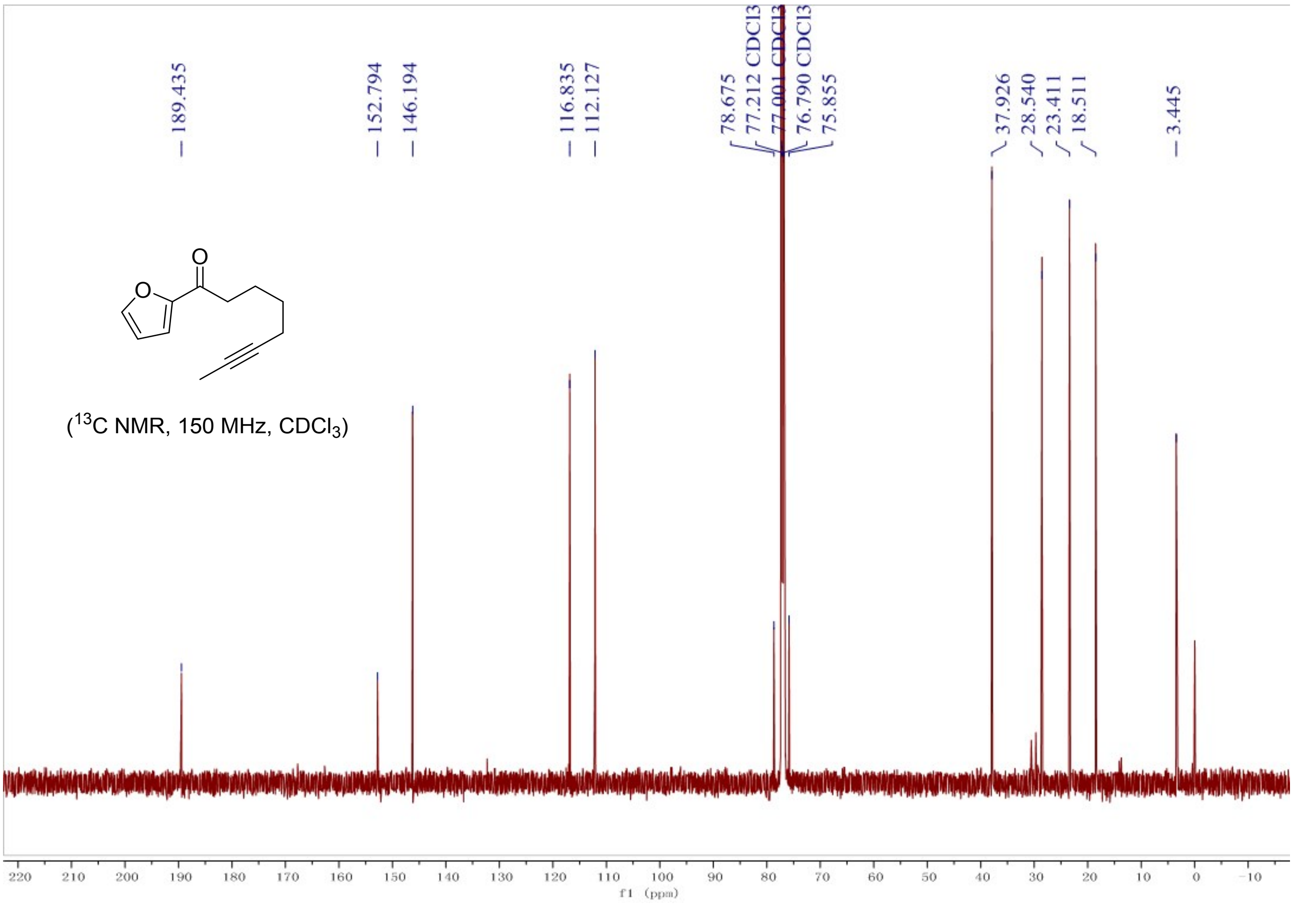
189.435
152.794
146.194
116.835
112.127
78.675
77.212 CDCl3
77.001 CDCl3
76.790 CDCl3
75.855
37.926
28.540
23.411
18.511
3.445
O
O
(13C NMR, 150 MHz, CDCl3)
f1 (ppm)

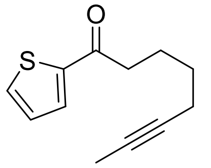

1-(thiophen-2-yl)oct-6-yn-1-one (3bt): colorless oil, 36 mg, 87%, Eluent: PE/EA = 20/1.

^{1}H NMR (400 MHz, $CDCl_3$, TMS): δ 7.72 (dd, *J* = 3.8, 1.2 Hz, 1H), 7.63 (dd, *J* = 5.0, 1.2 Hz, 1H), 7.13 (dd, *J* = 5.0, 3.8 Hz, 1H), 2.95-2.89 (m, 2H), 2.19 (tq, *J* = 7.2, 2.6 Hz, 2H), 1.91-1.81 (m, 2H), 1.77 (t, *J* = 2.6 Hz, 3H), 1.62-1.52 (m, 2H). **^{13}C NMR** (150 MHz, $CDCl_3$, TMS): δ 3.4, 18.5, 23.9, 28.5, 38.8, 75.9, 78.7, 128.0, 131.7, 133.4, 144.4, 193.1. **IR** (EtOH): ν 2960, 2899, 2209, 1662, 1600, 1544, 1385, 1377, 1279, 1065, 857, 735 cm^{-1}. **HRMS** (EI) m/z: $[M]^+$ Calcd. for $C_{12}H_{13}OS$ 205.0682; found 205.0675.

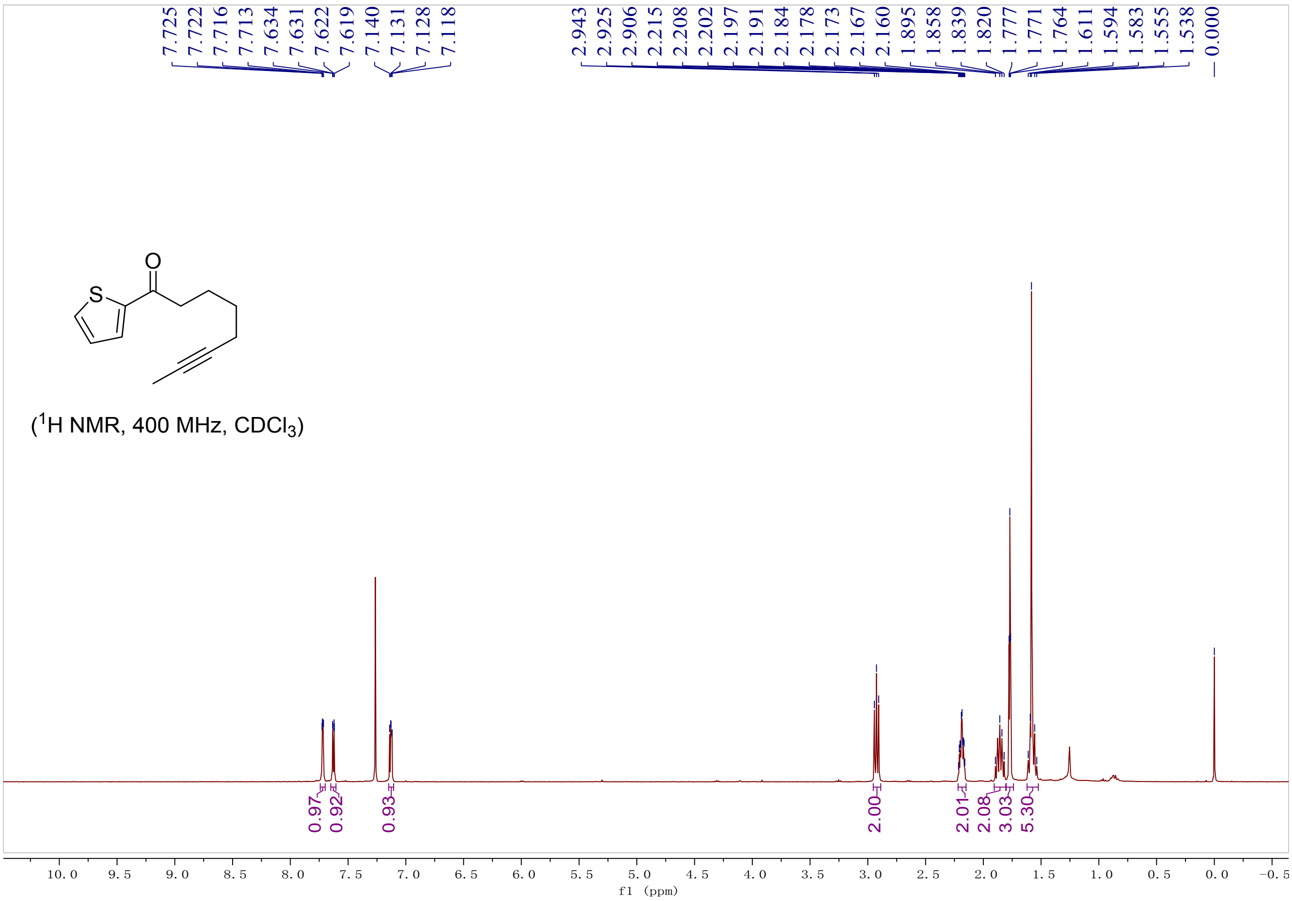
7.725
7.722
7.716
7.713
7.634
7.631
7.622
7.619
7.140
7.131
7.128
7.118
2.943
2.925
2.906
2.215
2.208
2.202
2.197
2.191
2.184
2.178
2.173
2.167
2.160
1.895
1.858
1.839
1.820
1.777
1.771
1.764
1.611
1.594
1.583
1.555
1.538
0.000
O
S
(1H NMR, 400 MHz, CDCl3)
0.97
0.92
0.93
2.00
2.01
2.08
3.03
5.30
f1 (ppm)

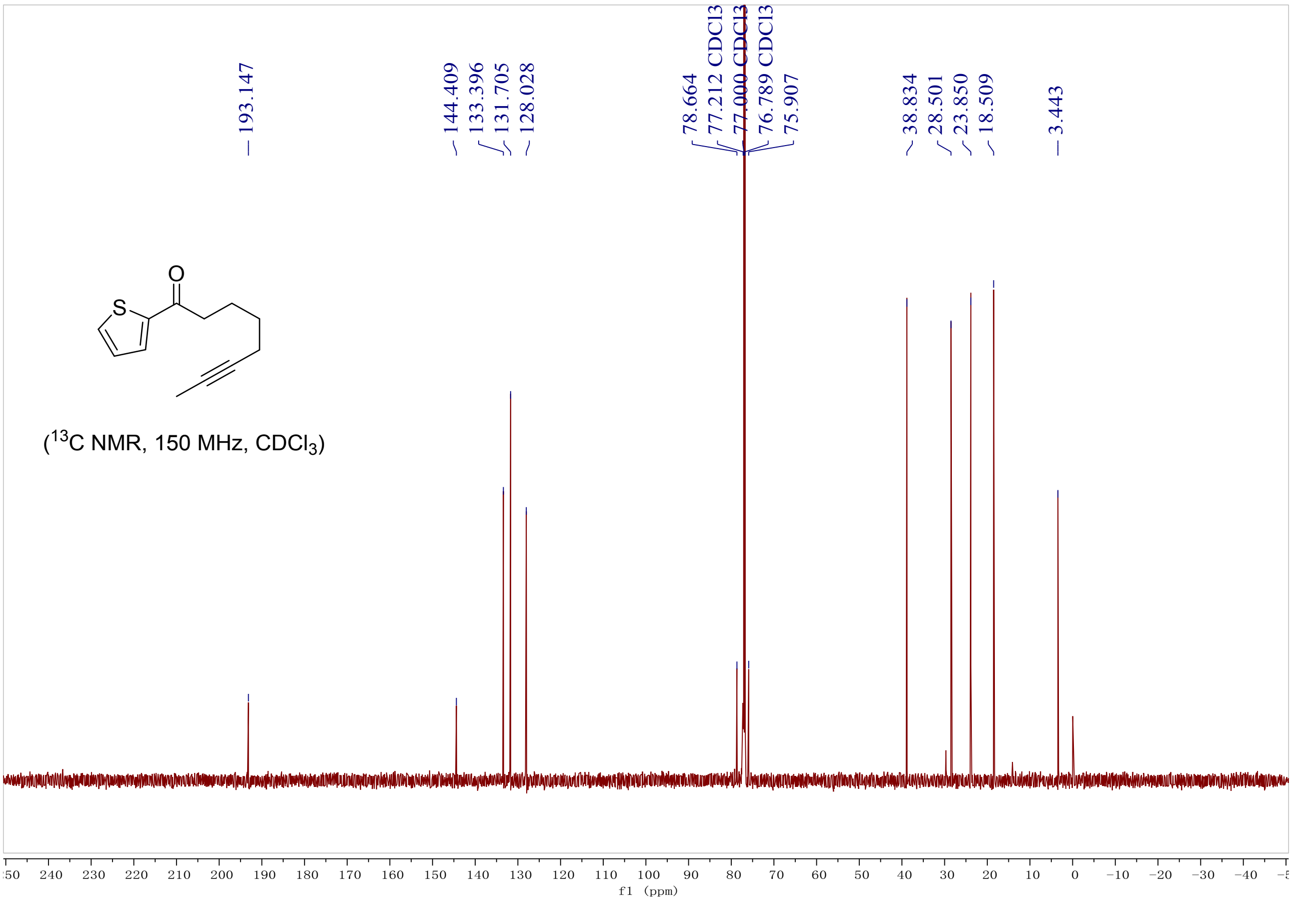
193.147
144.409
133.396
131.705
128.028
78.664
77.212 CDCl3
77.000 CDCl3
76.789 CDCl3
75.907
38.834
28.501
23.850
18.509
3.443
O
S
(13C NMR, 150 MHz, CDCl3)
f1 (ppm)

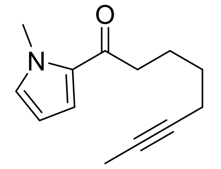

1-(1-methyl-1*H*-pyrrol-2-yl)oct-6-yn-1-one (3bu): colorless oil, 38 mg, 93%, Eluent: PE/EA = 20/1.

^{1}H NMR (400 MHz, $CDCl_3$, TMS): δ 6.96 (dd, *J* = 4.0, 1.6 Hz, 1H), 6.79 (t, *J* = 2.0 Hz, 1H), 6.12 (dd, *J* = 4.0, 2.5 Hz, 1H), 3.94 (s, 3H), 2.78 (t, *J* = 7.6 Hz, 2H), 2.17 (tq, *J* = 7.6, 2.6 Hz, 2H), 1.85-1.73 (m, 5H), 1.59-1.50 (m, 2H). **^{13}C NMR** (150 MHz, $CDCl_3$, TMS): δ 3.5, 18.6, 24.4, 28.7, 37.7, 38.5, 75.7, 78.8, 107.8, 118.9, 130.8, 191.3. **IR** (EtOH): ν 2973, 2915, 2892, 2219, 1672, 1554, 1482, 1325, 1234, 1185, 1032, 757, 699 cm^{-1}. **HRMS** (ESI) m/z: $[M+H]^+$ Calcd. for $C_{13}H_{18}ON$ 204.1383; found 204.1391.

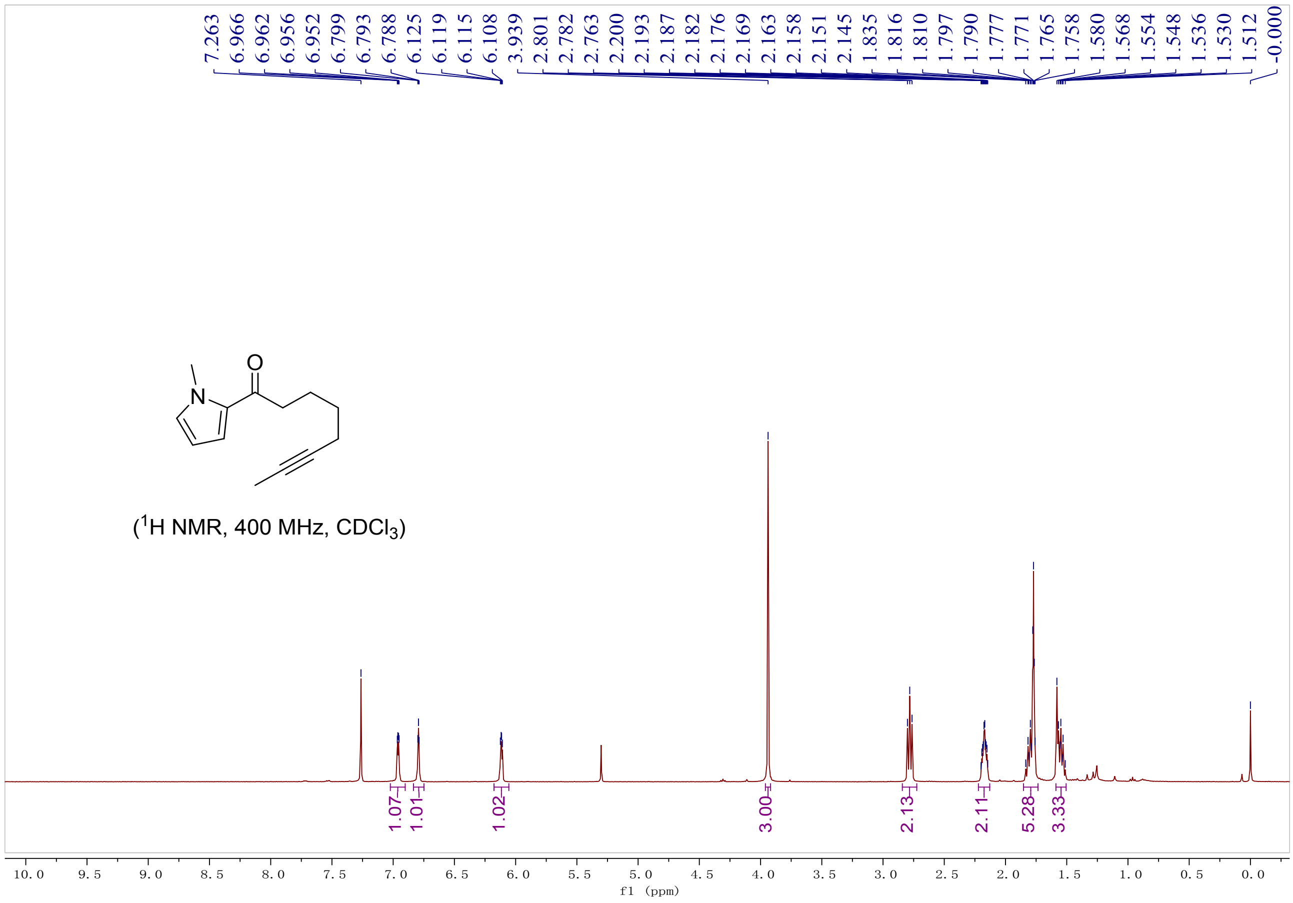

7.263 6.966 6.962 6.956 6.952 6.799 6.793 6.788 6.125 6.119 6.115 6.108 3.939 2.801 2.782 2.763 2.200 2.193 2.187 2.182 2.176 2.169 2.163 2.158 2.151 2.145 1.835 1.816 1.810 1.797 1.790 1.777 1.771 1.765 1.758 1.580 1.568 1.554 1.548 1.536 1.530 1.512 -0.000
O
N
(1H NMR, 400 MHz, CDCl3)
1.07 1.01 1.02 3.00 2.13 2.11 5.28 3.33
f1 (ppm)

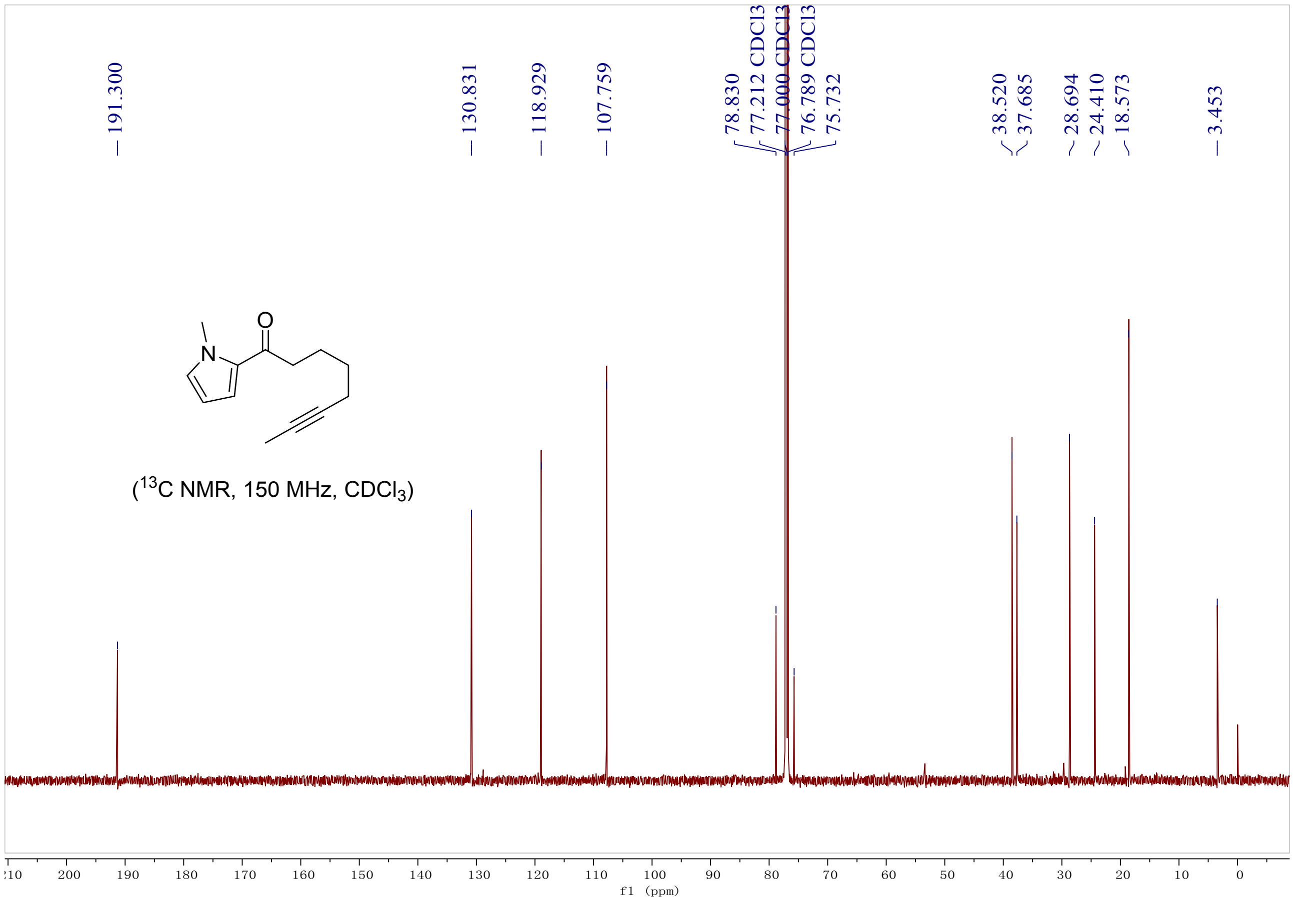

191.300 130.831 118.929 107.759 78.830 77.212 CDCl3 77.000 CDCl3 76.789 CDCl3 75.732 38.520 37.685 28.694 24.410 18.573 3.453
O
N
(13C NMR, 150 MHz, CDCl3)
f1 (ppm)

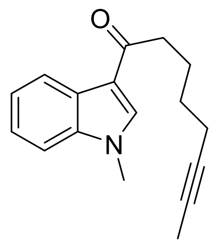

1-(1-methyl-1*H*-indol-3-yl)oct-6-yn-1-one (3bv): colorless oil, 45 mg, 88%, Eluent: PE/EA = 20/1.

^{1}H NMR (600 MHz, $CDCl_3$, TMS): δ 8.41-8.36 (m, 1H), 7.74 (s, 1H), 7.38-7.27 (m, 3H), 3.86 (s, 3H), 2.86 (t, *J* = 7.6 Hz, 2H), 2.20 (tq, *J* = 7.2, 2.6 Hz, 2H), 1.91-1.86 (m, 2H), 1.77 (t, *J* = 2.6 Hz, 3H), 1.63-1.59 (m, 2H). **^{13}C NMR** (150 MHz, $CDCl_3$, TMS): δ 3.5, 18.6, 24.4, 28.8, 33.5, 39.4, 75.7, 78.9, 109.6, 116.6, 122.5, 122.6, 123.3, 126.3, 135.2, 137.5, 195.6. **IR** (EtOH): ν 2946, 2929, 2220, 1678, 1554, 1449, 1239, 1165, 1002, 851, 757, 690 cm^{-1}. **HRMS** (ESI) m/z: $[M+Na]^+$ Calcd. for $C_{17}H_{19}ONNa$ 276.1359; found 276.1365.

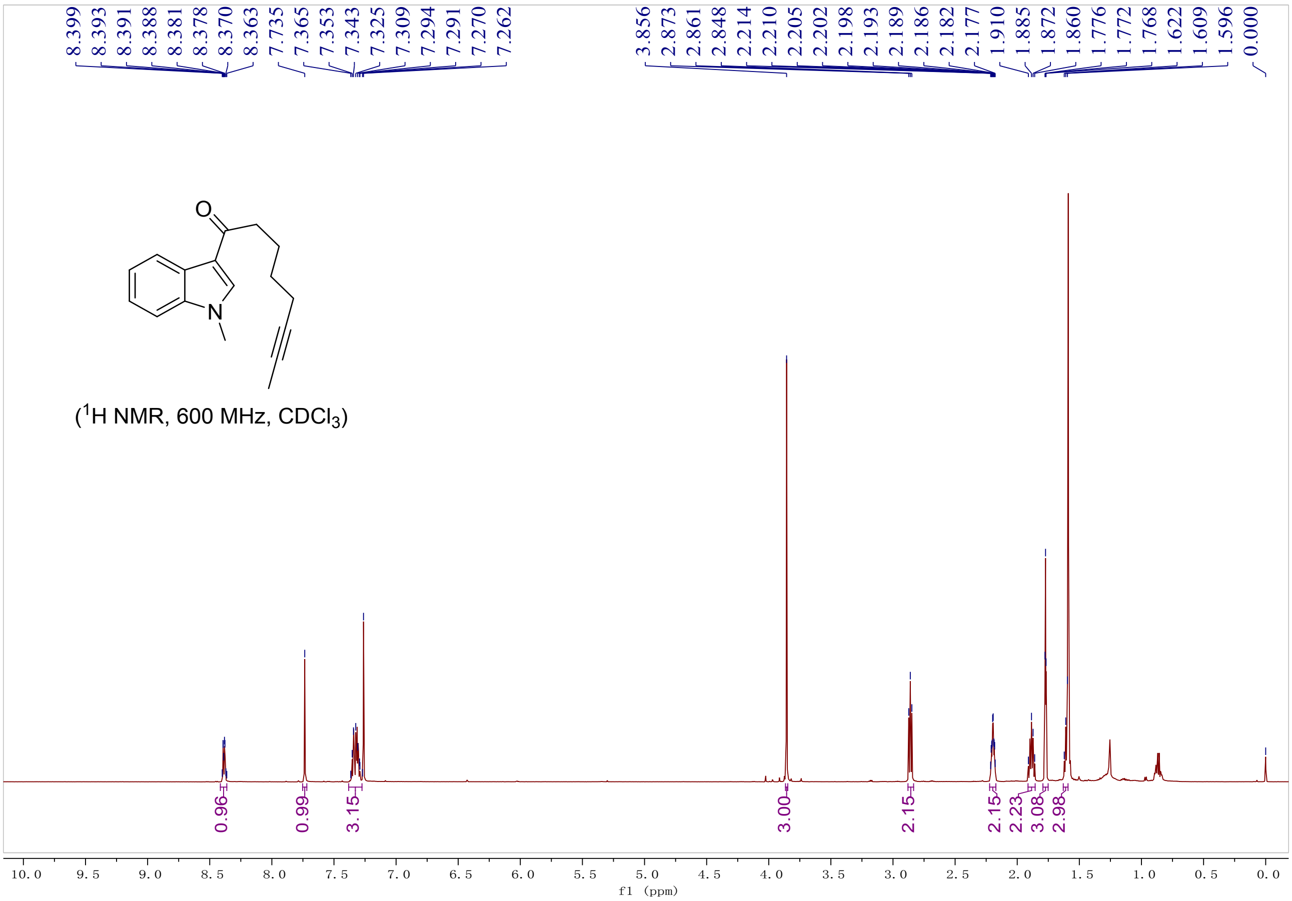

8.399
8.393
8.391
8.388
8.381
8.378
8.370
8.363
7.735
7.365
7.353
7.343
7.325
7.309
7.294
7.291
7.270
7.262
3.856
2.873
2.861
2.848
2.214
2.210
2.205
2.202
2.198
2.193
2.189
2.186
2.182
2.177
1.910
1.885
1.872
1.860
1.776
1.772
1.768
1.622
1.609
1.596
0.000
O
N
(1H NMR, 600 MHz, CDCl3)
0.96
0.99
3.15
3.00
2.15
2.15
2.23
3.08
2.98
f1 (ppm)

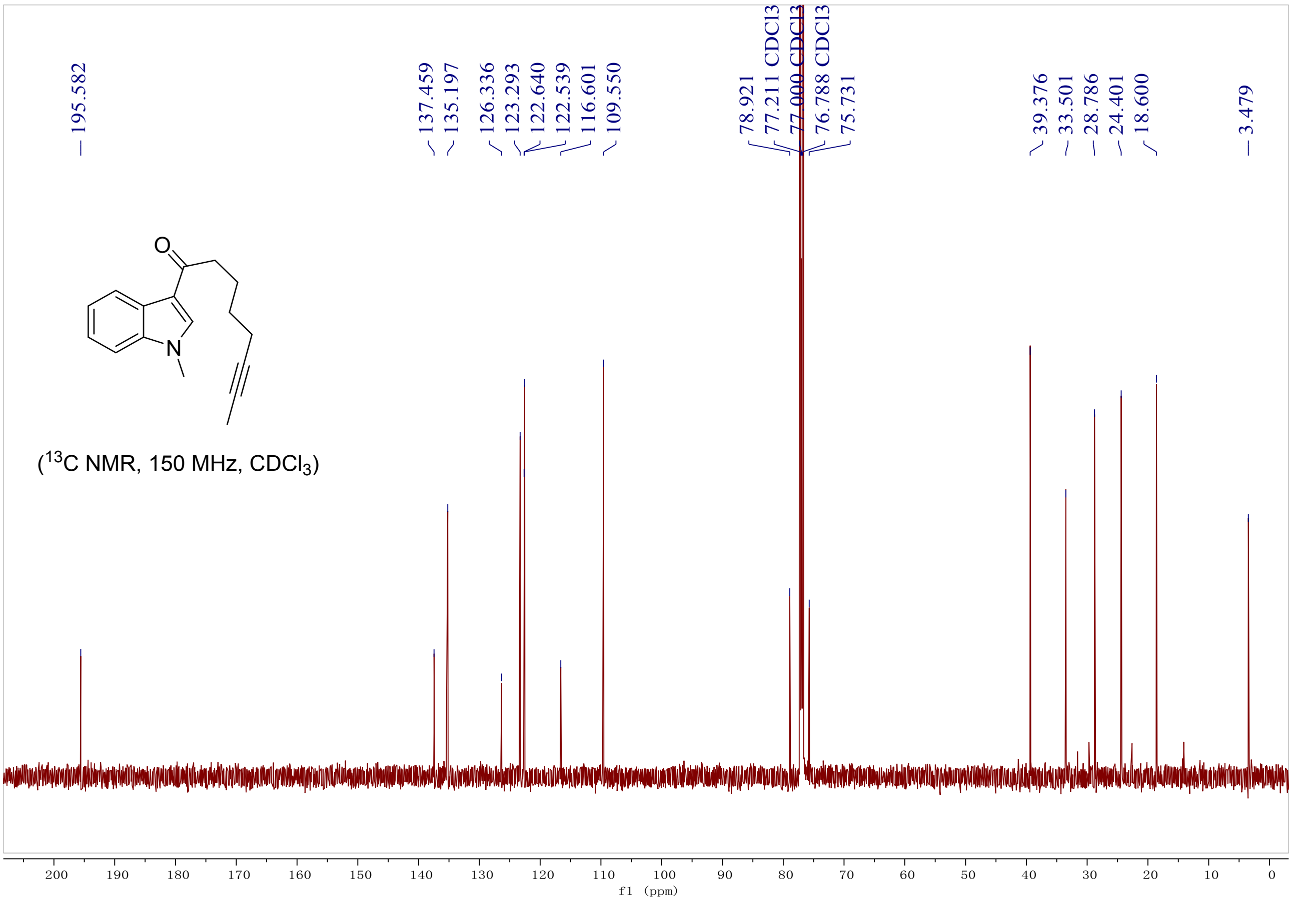

195.582
137.459
135.197
126.336
123.293
122.640
122.539
116.601
109.550
78.921
77.211 CDCl3
77.000 CDCl3
76.788 CDCl3
75.731
39.376
33.501
28.786
24.401
18.600
3.479
O
N
(13C NMR, 150 MHz, CDCl3)
f1 (ppm)

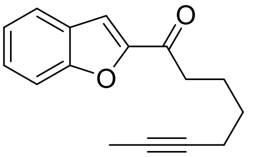

1-(benzofuran-2-yl)oct-6-yn-1-one (3bw): colorless oil, 42 mg, 88%, Eluent: PE/EA = 20/1.

^{1}H NMR (600 MHz, $CDCl_3$, TMS): δ 7.71 (dt, *J* = 8.0, 1.0 Hz, 1H), 7.58 (dd, *J* = 8.4, 1.0 Hz, 1H), 7.51 (d, *J* = 1.0 Hz, 1H), 7.51-7.45 (m, 1H), 7.34-7.28 (m, 1H), 2.99 (t, *J* = 7.2 Hz, 2H), 2.21 (tq, *J* = 7.2, 2.6 Hz, 2H), 1.92-1.86 (m, 2H), 1.77 (t, *J* = 2.6 Hz, 3H), 1.63-1.58 (m, 2H). **^{13}C NMR** (150 MHz, $CDCl_3$, TMS): δ 3.5, 18.5, 23.4, 28.5, 38.4, 76.0, 78.6, 112.5, 112.6, 123.2, 123.9, 127.1, 128.1, 152.6, 155.6, 191.3. **IR** (EtOH): ν 2940, 2912, 2854, 2215, 1672, 1499, 1321, 1254, 1132, 854, 777, 765, 702, 689 cm^{-1}. **HRMS** (ESI) m/z: $[M+H]^+$ Calcd. for $C_{16}H_{17}O_2$ 241.1223; found 241.1230.

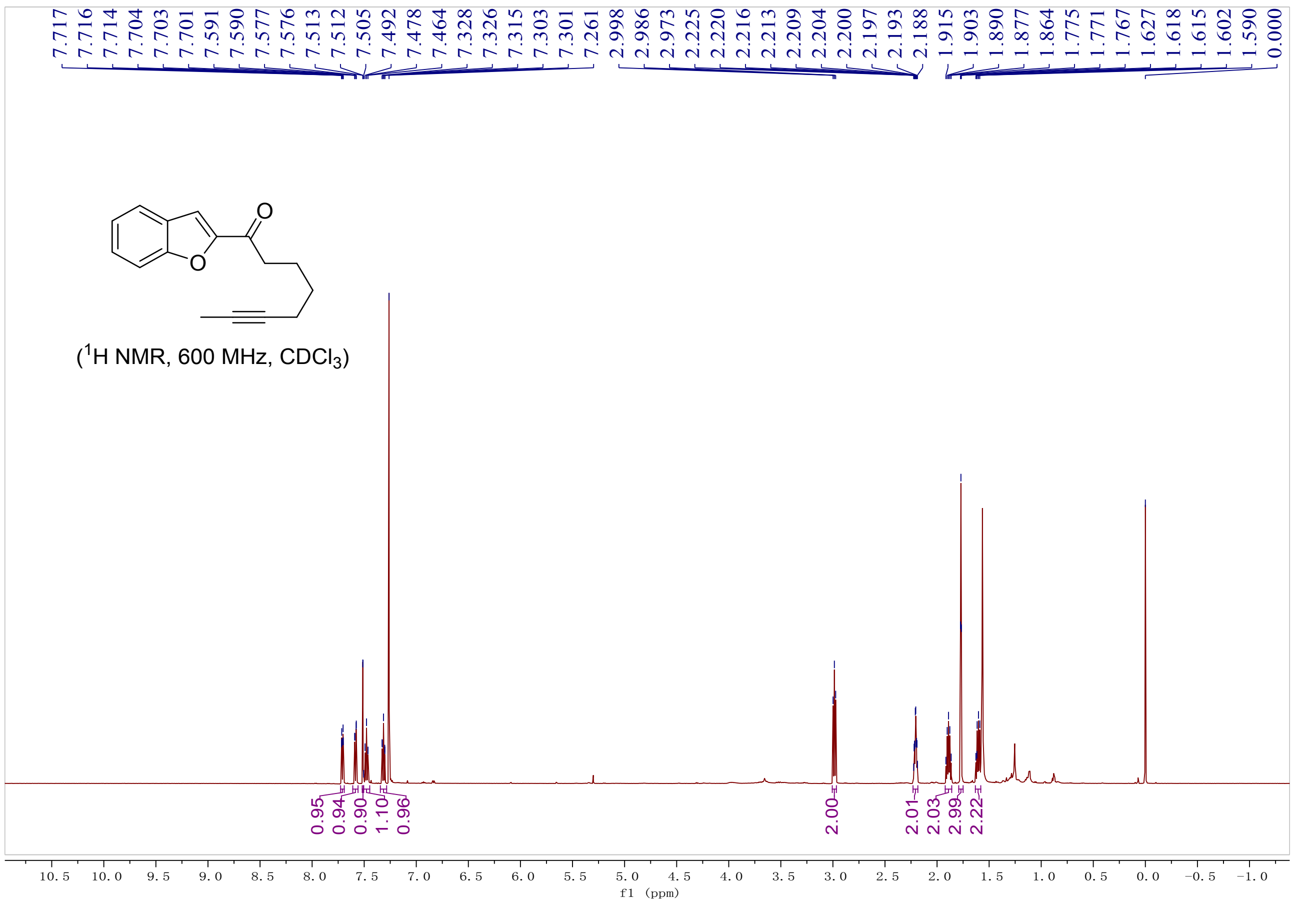
7.717
7.716
7.714
7.704
7.703
7.701
7.591
7.590
7.577
7.576
7.513
7.512
7.505
7.492
7.478
7.464
7.328
7.326
7.315
7.303
7.301
7.261
2.998
2.986
2.973
2.225
2.220
2.216
2.213
2.209
2.204
2.200
2.197
2.193
2.188
1.915
1.903
1.890
1.877
1.864
1.775
1.771
1.767
1.627
1.618
1.615
1.602
1.590
0.000
O
O
(^{1}H NMR, 600 MHz, CDCl$_3$)
0.95
0.94
0.90
1.10
0.96
2.00
2.01
2.03
2.99
2.22
f1 (ppm)

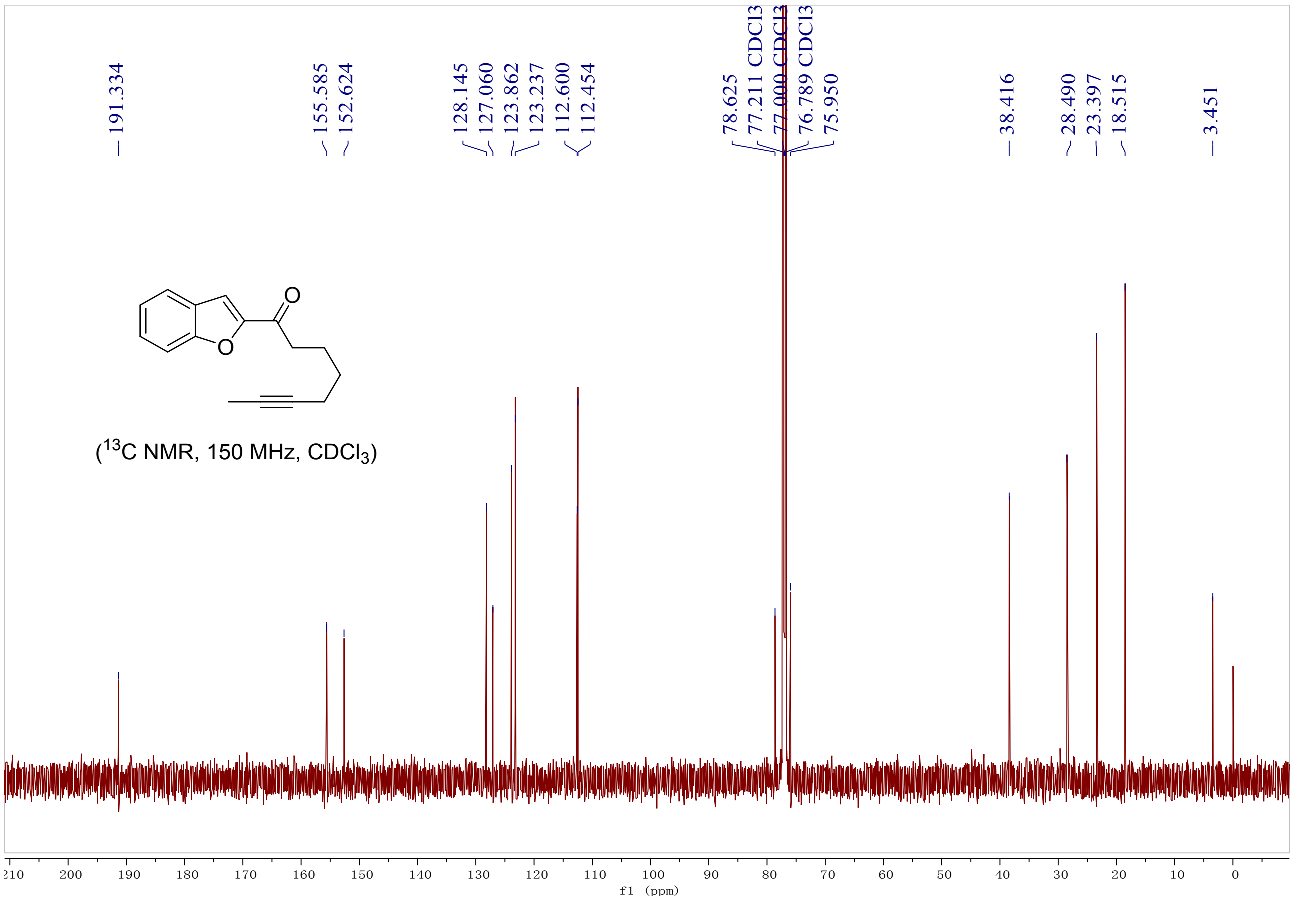
191.334
155.585
152.624
128.145
127.060
123.862
123.237
112.600
112.454
78.625
77.211 CDCl3
77.000 CDCl3
76.789 CDCl3
75.950
38.416
28.490
23.397
18.515
3.451
O
O
(^{13}C NMR, 150 MHz, CDCl$_3$)
f1 (ppm)

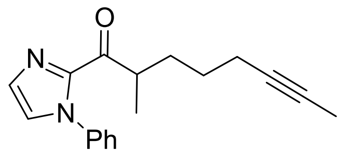

2-Methyl-1-(1-phenyl-1*H*-imidazol-2-yl)oct-6-yn-1-one (3bx): colorless oil, 56 mg, 89%, Eluent: PE/EA = 20/1.

^{1}H NMR (600 MHz, $CDCl_3$, TMS): δ 7.47-7.44 (m, 3H), 7.30-7.23 (m, 4H), 3.92-3.87 (m, 1H), 2.17-2.06 (m, 2H), 1.85-1.78 (m, 1H), 1.74 (t, J = 2.6 Hz, 3H), 1.56-1.45 (m, 3H), 1.18 (d, J = 6.8 Hz, 3H). **^{13}C NMR** (150 MHz, $CDCl_3$, TMS): δ 3.4, 17.0, 18.8, 26.8, 32.4, 41.0, 76.8, 78.9, 125.7, 127.1, 128.6, 128.9, 129.5, 138.5, 142.7, 195.2. **IR** (EtOH): ν 2973, 2925, 2872, 2210, 1654, 1534, 1444, 1380, 13028, 1087, 1045, 1009, 851, 767, 700, 653 cm^{-1}. **HRMS** (EI) m/z: $[M]^+$ Calcd. for $C_{18}H_{19}ON_2$ 279.1492; found 279.1487.

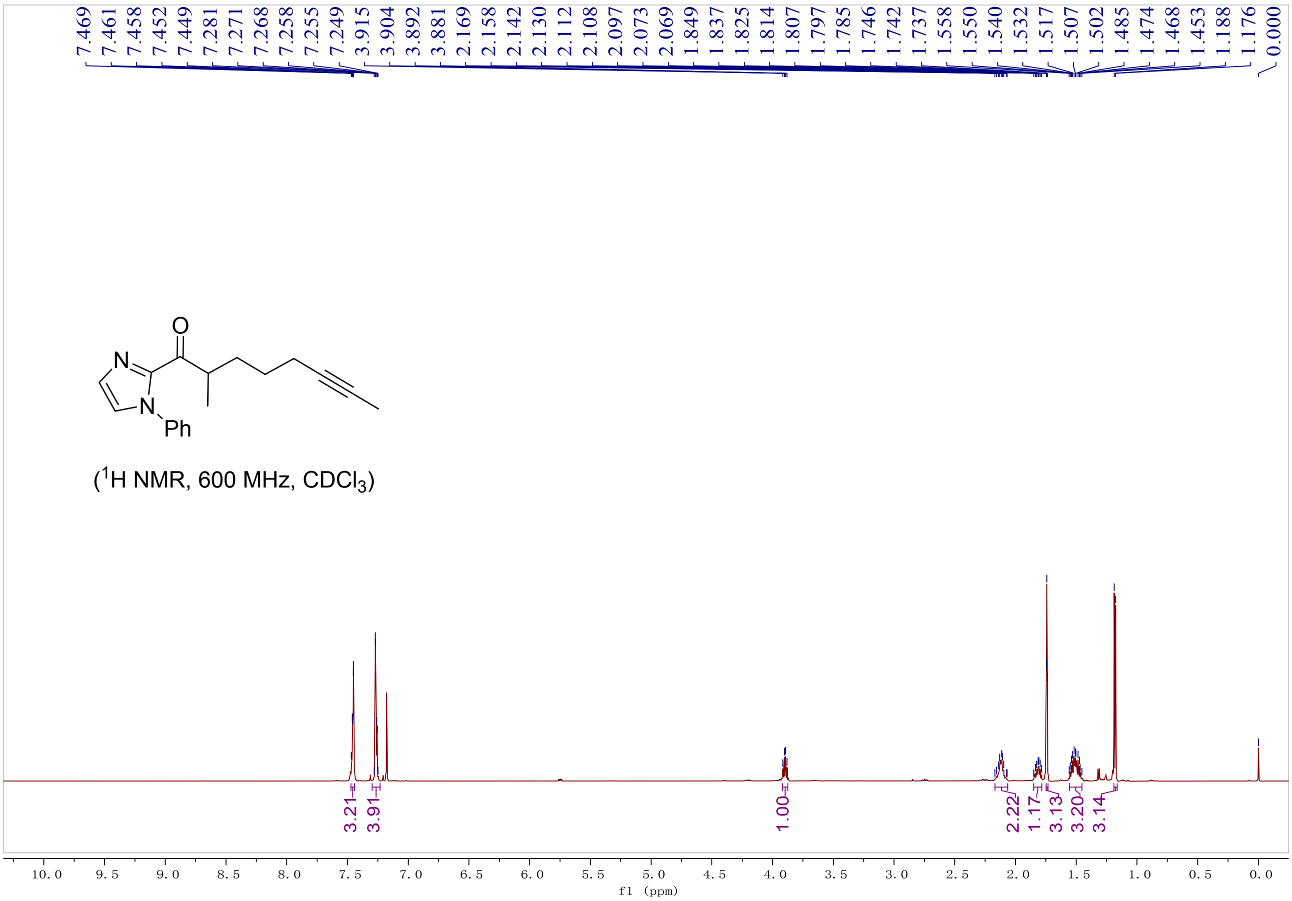
7.469
7.461
7.458
7.452
7.449
7.281
7.271
7.268
7.258
7.255
7.249
3.915
3.904
3.892
3.881
2.169
2.158
2.142
2.130
2.112
2.108
2.097
2.073
2.069
1.849
1.837
1.825
1.814
1.807
1.797
1.785
1.746
1.742
1.737
1.558
1.550
1.540
1.532
1.517
1.507
1.502
1.485
1.474
1.468
1.453
1.188
1.176
0.000
O
N
N
Ph
(^{1}H NMR, 600 MHz, $CDCl_3$)
3.21
3.91
1.00
2.22
1.17
3.13
3.20
3.14
10.0 9.5 9.0 8.5 8.0 7.5 7.0 6.5 6.0 5.5 5.0 4.5 4.0 3.5 3.0 2.5 2.0 1.5 1.0 0.5 0.0
f1 (ppm)

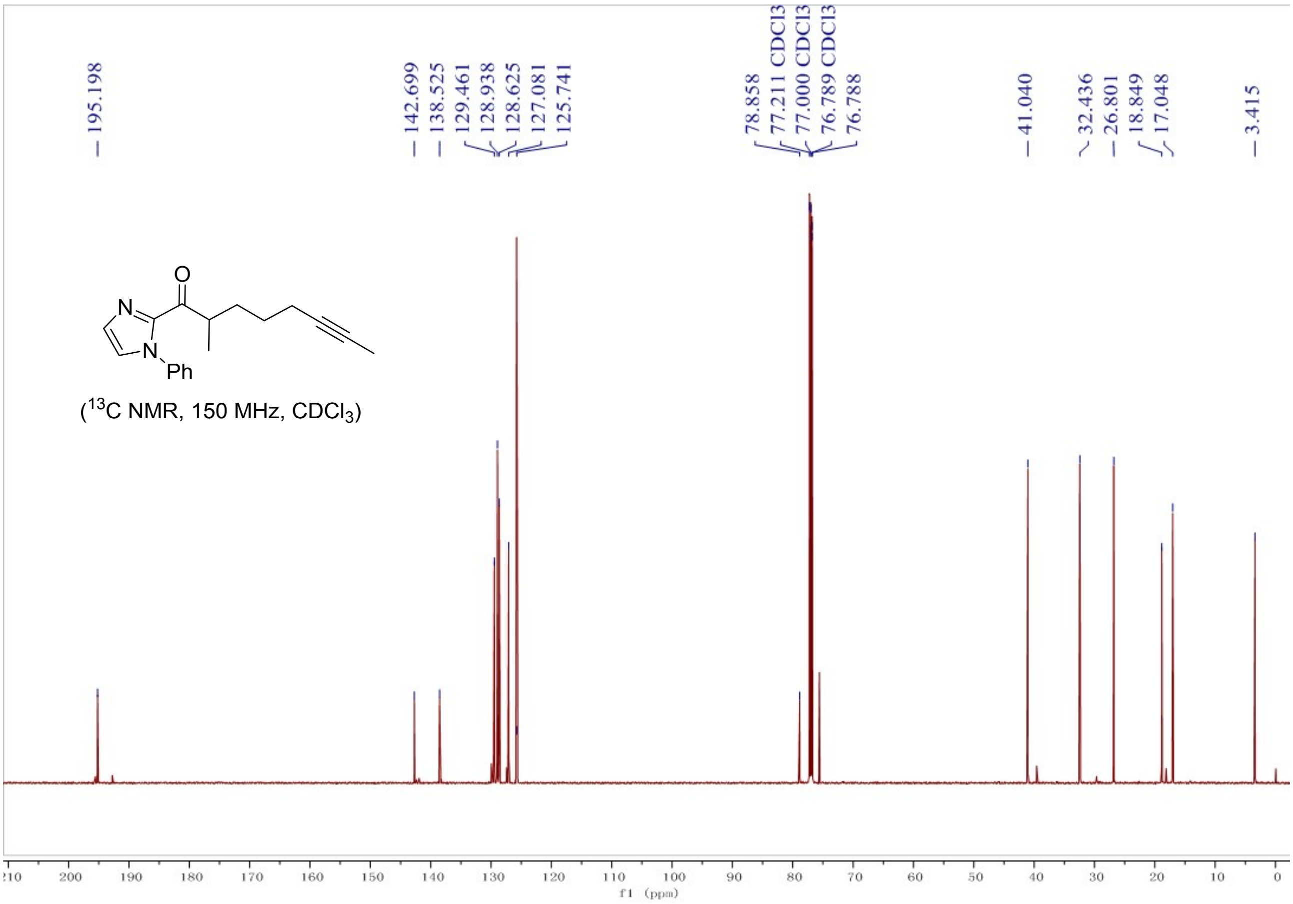
195.198
142.699
138.525
129.461
128.938
128.625
127.081
125.741
78.858
77.211 CDCl3
77.000 CDCl3
76.789 CDCl3
76.788
41.040
32.436
26.801
18.849
17.048
3.415
O
N
N
Ph
(^{13}C NMR, 150 MHz, $CDCl_3$)
210 200 190 180 170 160 150 140 130 120 110 100 90 80 70 60 50 40 30 20 10 0
f1 (ppm)

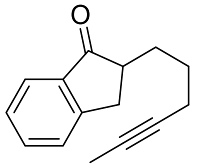

2-(hex-4-yn-1-yl)-2,3-dihydro-1*H*-inden-1-one (3by): colorless oil, 33 mg, 78%, Eluent: PE/EA = 20/1.

^{1}H NMR (600 MHz, $CDCl_3$, TMS): δ 7.76 (d, *J* = 7.6 Hz, 1H), 7.62-7.56 (m, 1H), 7.46 (d, *J* = 7.6 Hz, 1H), 7.40-7.34 (m, 1H), 3.34 (dd, *J* = 17.2, 7.8 Hz, 1H), 2.83 (dd, *J* = 17.2, 4.0 Hz, 1H), 2.68 (tt, *J* = 8.4, 4.2 Hz, 1H), 2.25-2.14 (m, 2H), 2.09-2.00 (m, 1H), 1.77 (t, *J* = 2.4 Hz, 3H), 1.66-1.57 (m, 2H). **^{13}C NMR** (150 MHz, $CDCl_3$, TMS): δ 3.5, 18.8, 26.9, 30.7, 32.9, 47.1, 75.9, 78.7, 123.9, 126.5, 127.4, 134.7, 136.8, 153.7, 208.7. **IR** (EtOH): ν 2973, 2883, 2210, 1653, 1399, 1087, 1045, 880, 852, 747, 627 cm^{-1}. **HRMS** (EI) m/z: $[M]^+$ Calcd. for $C_{15}H_{16}O$ 212.1196; found 212.1194.

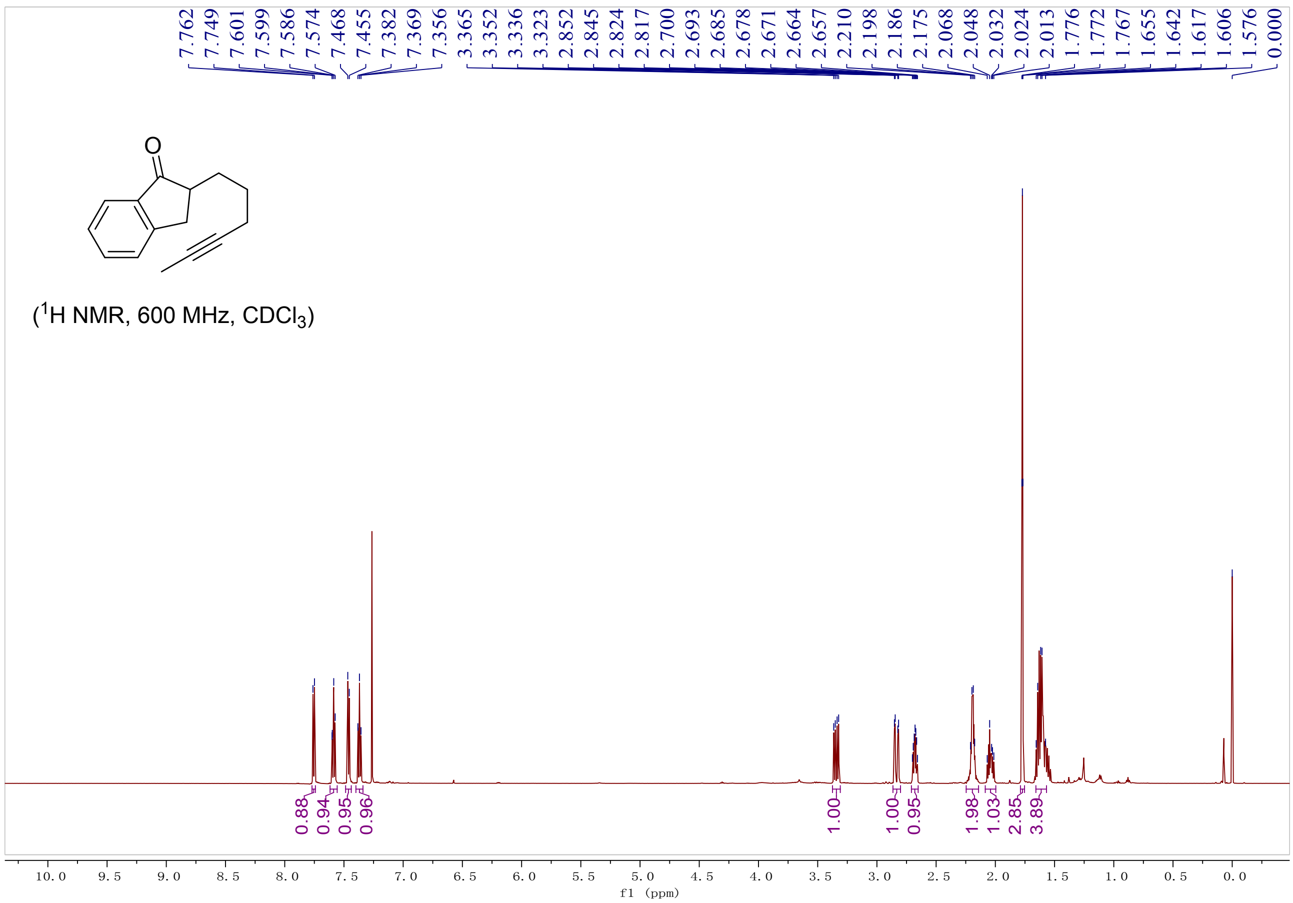

O
(^{1}H NMR, 600 MHz, $CDCl_3$)
7.762 7.749 7.601 7.599 7.586 7.574 7.468 7.455 7.382 7.369 7.356 3.365 3.352 3.336 3.323 2.852 2.845 2.824 2.817 2.700 2.693 2.685 2.678 2.671 2.664 2.657 2.210 2.198 2.186 2.175 2.068 2.048 2.032 2.024 2.013 1.776 1.772 1.767 1.655 1.642 1.617 1.606 1.576 0.000
0.88 0.94 0.95 0.96 1.00 1.00 0.95 1.98 1.03 2.85 3.89
10.0 9.5 9.0 8.5 8.0 7.5 7.0 6.5 6.0 5.5 5.0 4.5 4.0 3.5 3.0 2.5 2.0 1.5 1.0 0.5 0.0
f1 (ppm)

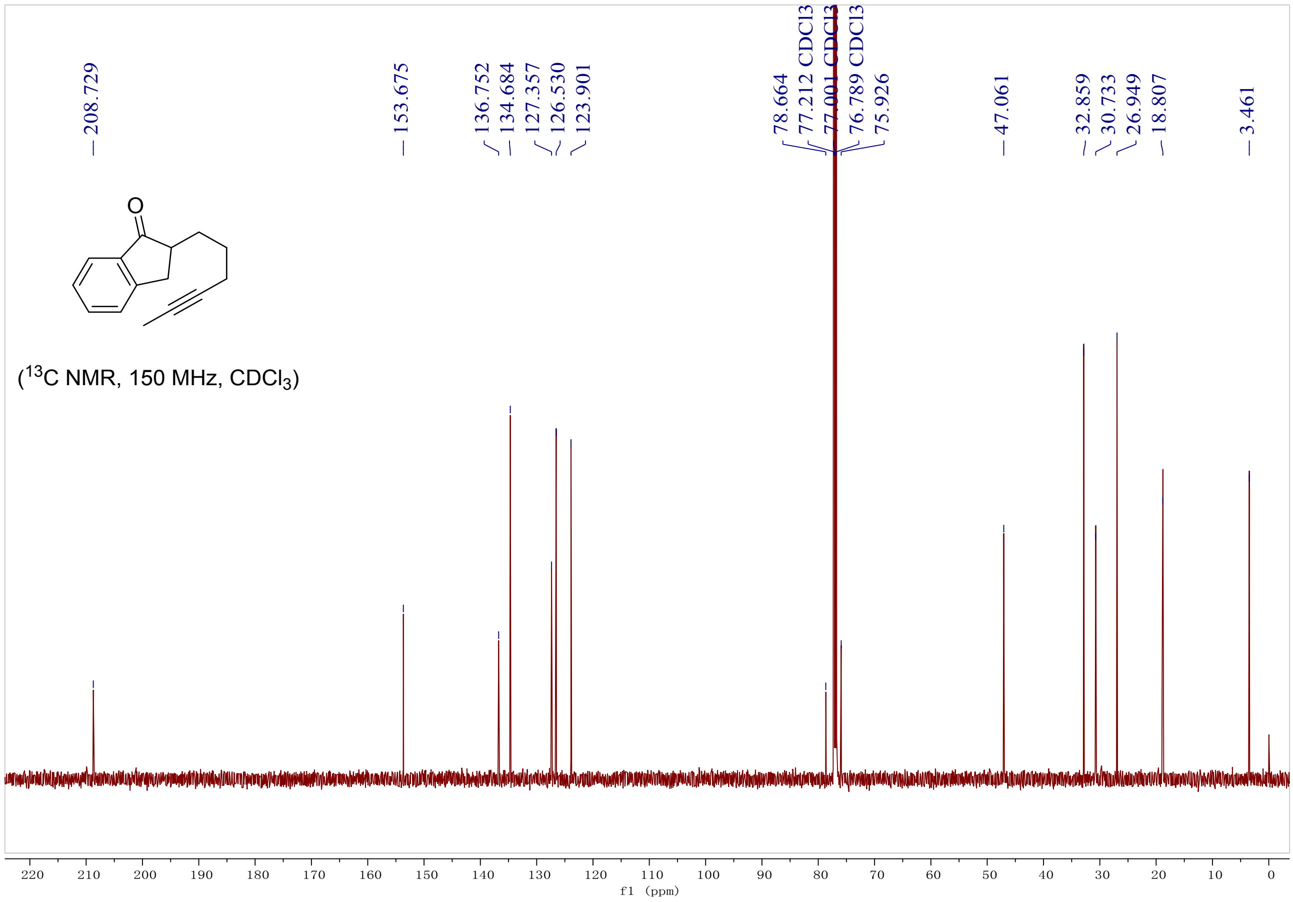

O
(^{13}C NMR, 150 MHz, $CDCl_3$)
208.729 153.675 136.752 134.684 127.357 126.530 123.901 78.664 77.212 CDCl3 77.001 CDCl3 76.789 CDCl3 75.926 47.061 32.859 30.733 26.949 18.807 3.461
220 210 200 190 180 170 160 150 140 130 120 110 100 90 80 70 60 50 40 30 20 10 0
f1 (ppm)

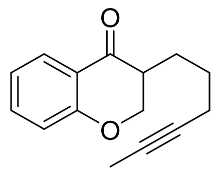

3-(hex-4-yn-1-yl)chroman-4-one (3bz): colorless oil, 36 mg, 78%, Eluent: PE/EA = 20/1.

^{1}H NMR (600 MHz, $CDCl_3$, TMS): δ 7.89 (dd, J = 7.8, 1.8 Hz, 1H), 7.50-7.42 (m, 1H), 7.02 (t, J = 7.6 Hz, 1H), 6.96 (d, J = 8.4 Hz, 1H), 4.53 (dd, J = 11.4, 4.3 Hz, 1H), 4.32-4.26 (m, 1H), 2.72-2.65 (m, 1H), 2.23-2.15 (m, 2H), 2.01-1.93 (m, 1H), 1.77 (t, J = 2.6 Hz, 3H), 1.64-1.60 (m, 3H). **^{13}C NMR** (150 MHz, $CDCl_3$, TMS): δ 3.4, 18.7, 25.6, 26.4, 45.5, 70.5, 76.1, 78.4, 117.7, 120.6, 121.4, 127.4, 135.7, 161.4, 194.4. **IR** (EtOH): ν 2944, 2935, 2842, 2214, 1682, 1544, 1470, 1355, 1297, 1233, 1154, 1085, 1002, 844, 747, 693, 632 cm^{-1}. **HRMS** (ESI) m/z: $[M+H]^+$ Calcd. for $C_{15}H_{17}O_2$ 229.1223; found 229.1225.

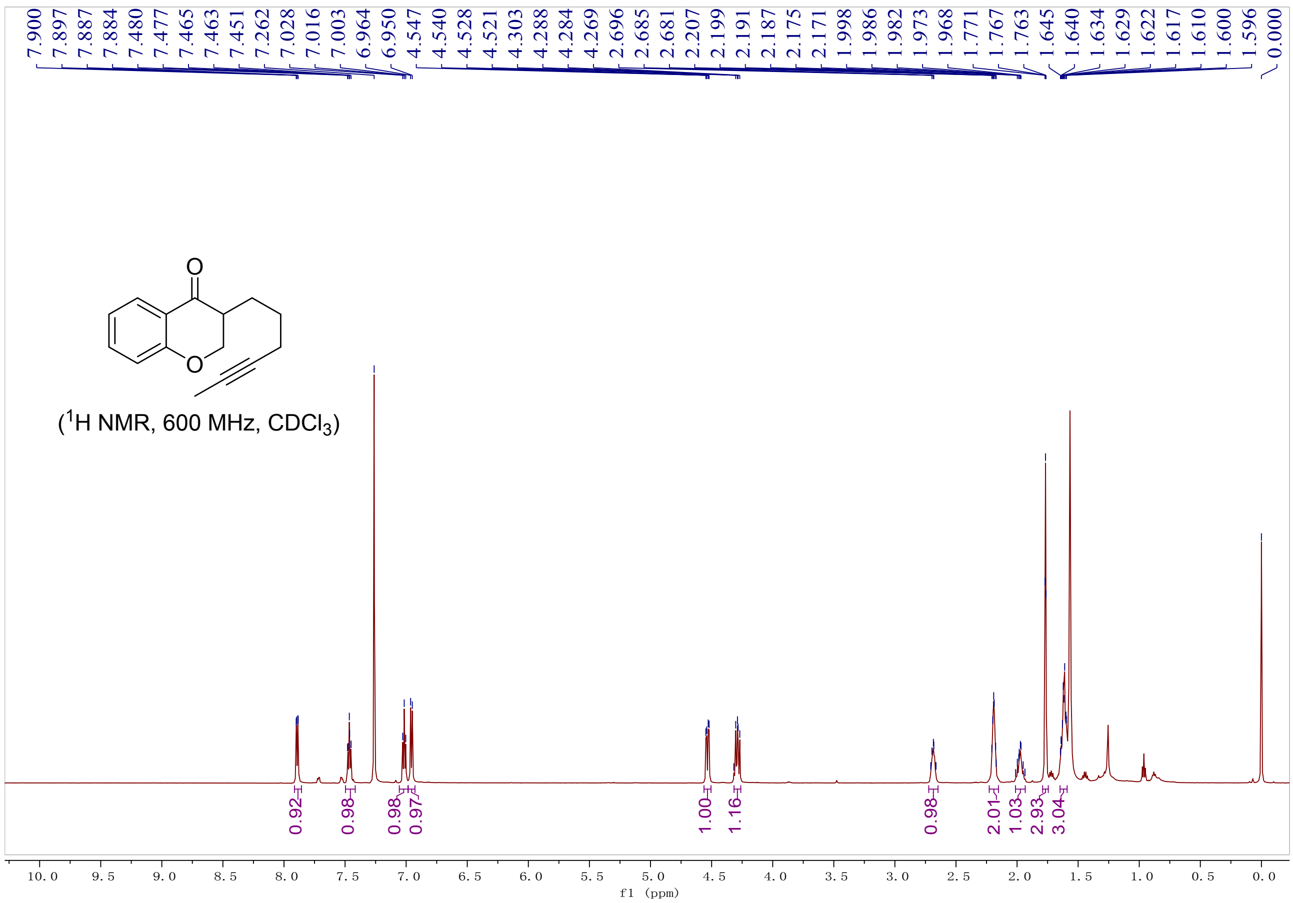

(1H NMR, 600 MHz, CDCl3)
7.900 7.897 7.887 7.884 7.480 7.477 7.465 7.463 7.451 7.262 7.028 7.016 7.003 6.964 6.950 4.547 4.540 4.528 4.521 4.303 4.288 4.284 4.269 2.696 2.685 2.681 2.207 2.199 2.191 2.187 2.175 2.171 1.998 1.986 1.982 1.973 1.968 1.771 1.767 1.763 1.645 1.640 1.634 1.629 1.622 1.617 1.610 1.600 1.596 0.000
0.92 0.98 0.98 0.97 1.00 1.16 0.98 2.01 1.03 2.93 3.04
f1 (ppm)

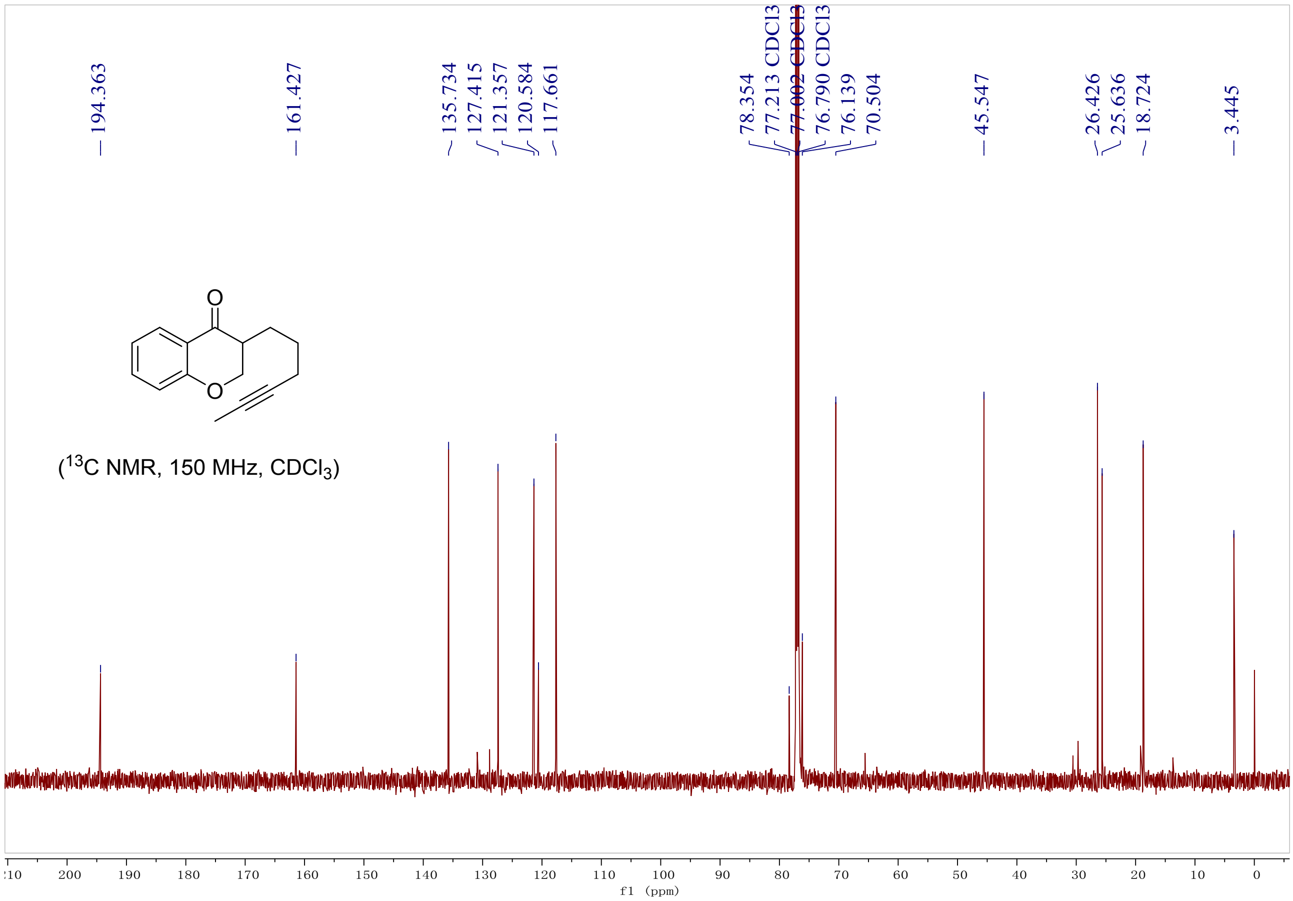

(13C NMR, 150 MHz, CDCl3)
194.363 161.427 135.734 127.415 121.357 120.584 117.661 78.354 77.213 CDCl3 77.002 CDCl3 76.790 CDCl3 76.139 70.504 45.547 26.426 25.636 18.724 3.445
f1 (ppm)

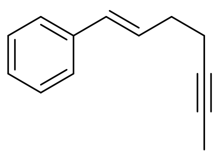

(*E*)-Hept-1-en-5-yn-1-ylbenzene (3baa): colorless oil, 33 mg, 97%, Eluent: PE/EA = 20/1.

^{1}H NMR (400 MHz, $CDCl_3$, TMS): δ 7.32-7.26 (m, 2H), 7.23 (t, *J* = 7.6 Hz, 2H), 7.16-7.10 (m, 1H), 6.36 (d, *J* = 16.0 Hz, 1H), 6.19 (dt, *J* = 16.0, 6.8 Hz, 1H), 2.38-2.27 (m, 2H), 2.28-2.17 (m, 2H), 1.73 (t, *J* = 2.6 Hz, 3H). **^{13}C NMR** (100 MHz, $CDCl_3$, TMS): δ 3.5, 19.0, 32.6, 76.1, 78.5, 126.0, 127.0, 128.5, 129.1, 130.7, 137.6. **IR** (EtOH): ν 2958, 2928, 2870, 2215, 1650, 1586, 1452, 1215, 1166, 914, 872, 780, 701 cm^{-1}. **HRMS** (ESI) m/z: $[M+H]^+$ Calcd. for $C_{13}H_{15}$ 171.1168; found 171.1170.

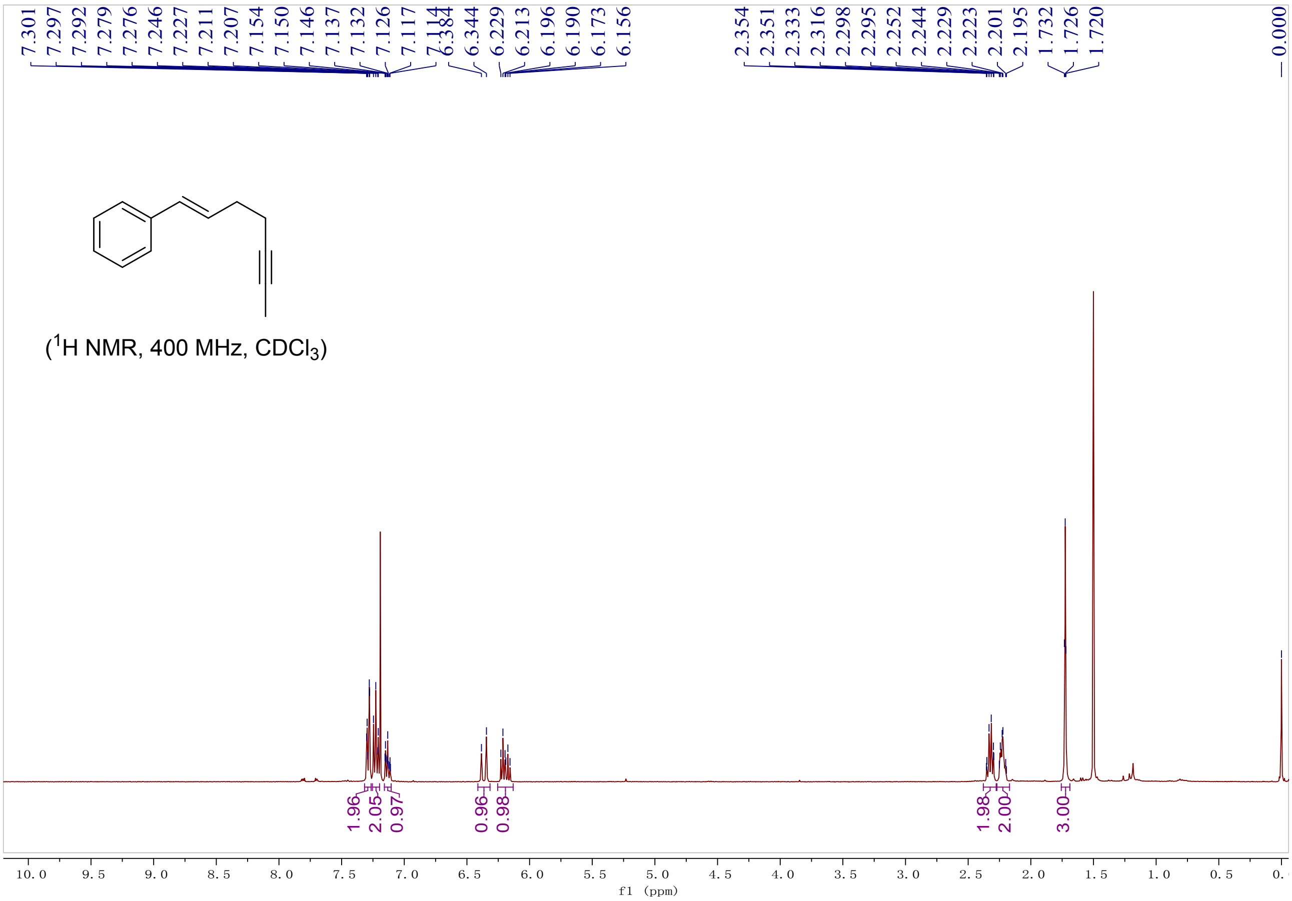

7.301
7.297
7.292
7.279
7.276
7.246
7.227
7.211
7.207
7.154
7.150
7.146
7.137
7.132
7.126
7.117
7.114
6.384
6.344
6.229
6.213
6.196
6.190
6.173
6.156
2.354
2.351
2.333
2.316
2.298
2.295
2.252
2.244
2.229
2.223
2.201
2.195
1.732
1.726
1.720
0.000
(1H NMR, 400 MHz, CDCl3)
1.96
2.05
0.97
0.96
0.98
1.98
2.00
3.00
f1 (ppm)

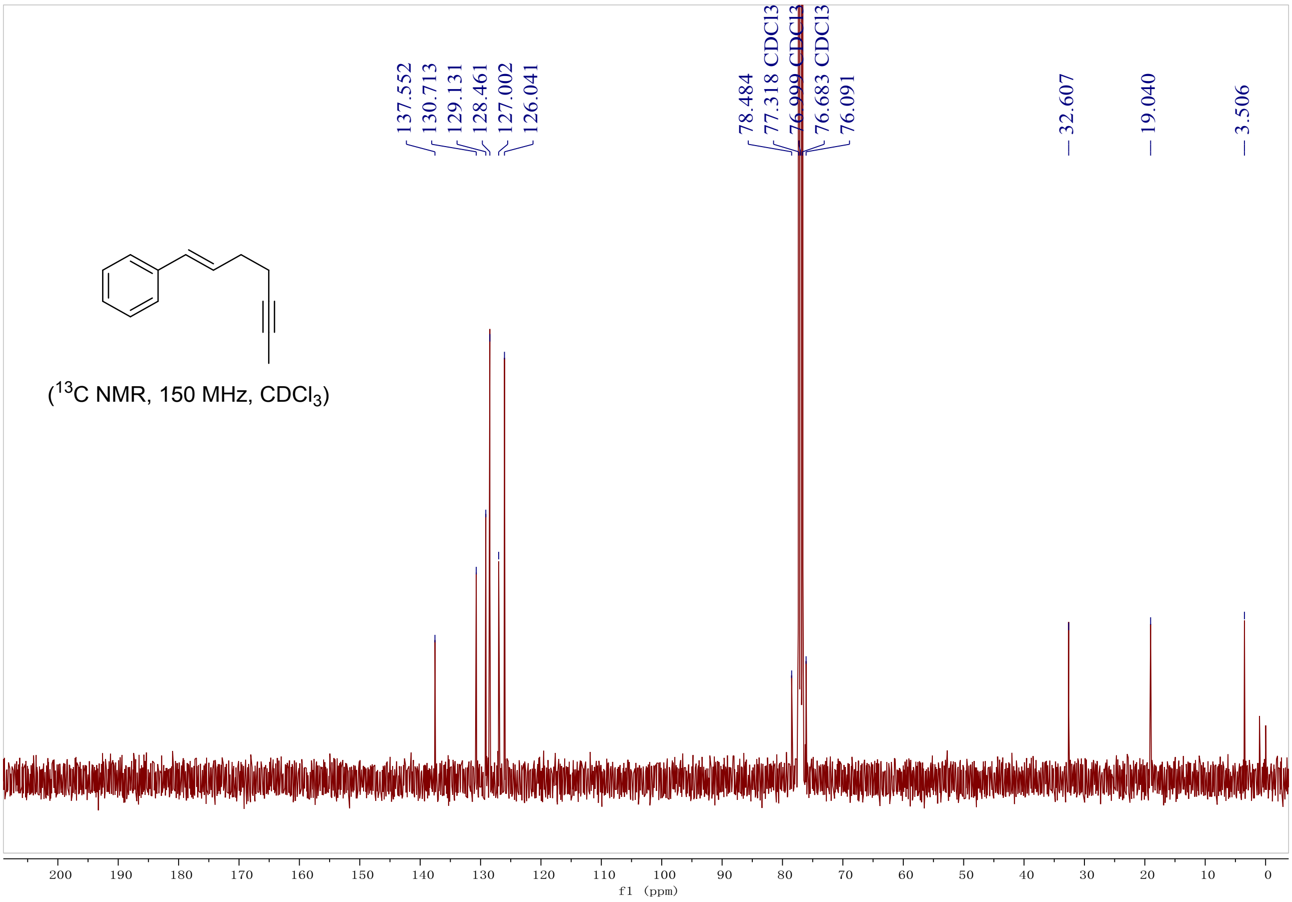

137.552
130.713
129.131
128.461
127.002
126.041
78.484
77.318 CDCl3
76.999 CDCl3
76.683 CDCl3
76.091
32.607
19.040
3.506
(13C NMR, 150 MHz, CDCl3)
f1 (ppm)

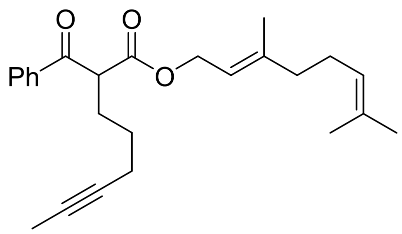

(*E*)-3,7-Dimethylocta-2,6-dien-1-yl 2-benzoyloct-6-ynoate (3bab): colorless oil, 67 mg, 88%, Eluent: PE/EA = 20/1.

^{1}H NMR (600 MHz, $CDCl_3$, TMS): δ 8.02-7.97 (m, 2H), 7.58 (t, *J* = 7.4 Hz, 1H), 7.50-7.44 (m, 2H), 5.23 (td, *J* = 7.2, 1.4 Hz, 1H), 5.05 (tt, *J* = 6.8, 1.4 Hz, 1H), 4.60 (dd, *J* = 7.2, 3.4 Hz, 2H), 4.33 (t, *J* = 7.2 Hz, 1H), 2.18 (tq, *J* = 7.2, 2.4 Hz, 2H), 2.14-2.07 (m, 2H), 2.05-2.01 (m, 2H), 1.99-1.95 (m, 2H), 1.74 (t, *J* = 2.4 Hz, 3H), 1.67 (s, 3H), 1.61 (s, 3H), 1.57-1.53 (m, 2H). **^{13}C NMR** (150 MHz, $CDCl_3$, TMS): δ 3.4, 16.4, 17.7, 18.5, 25.6, 26.2, 26.8, 28.1, 39.4, 53.9, 62.2, 76.2, 78.3, 117.7, 123.7, 128.7, 131.8, 133.4, 136.2, 143.0, 169.9, 194.9. **IR** (EtOH): ν 3002, 2975, 2935, 2882, 2220, 1682, 1648, 1599, 1544, 1482, 1382, 1327, 1086, 1044, 1009, 879, 737, 634 cm^{-1}. **HRMS** (ESI) m/z: $[M+Na]^+$ Calcd. for $C_{25}H_{32}O_3Na$ 403.2244; found 403.2250.

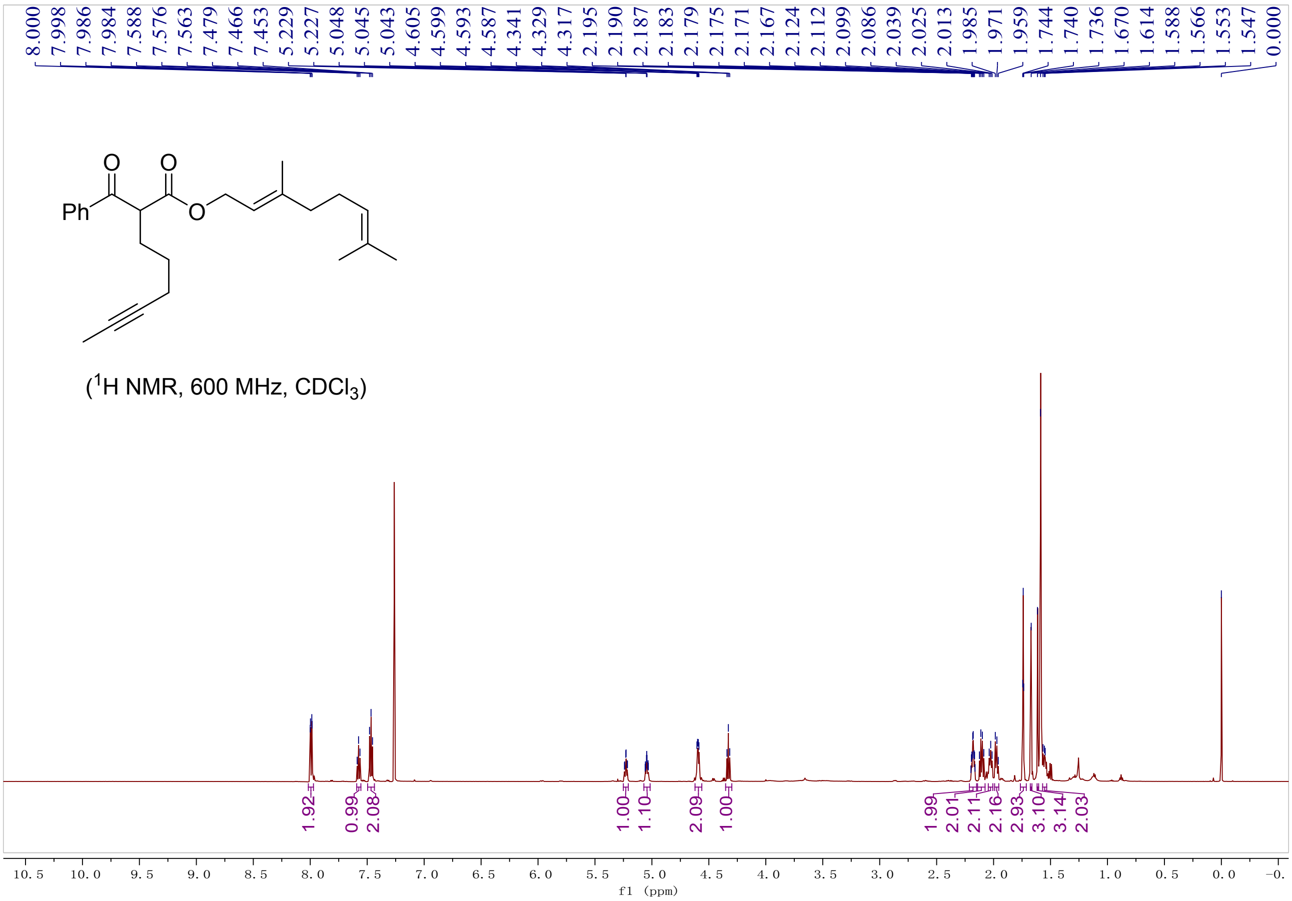
8.000
7.998
7.986
7.984
7.588
7.576
7.563
7.479
7.466
7.453
5.229
5.227
5.048
5.045
5.043
4.605
4.599
4.593
4.587
4.341
4.329
4.317
2.195
2.190
2.187
2.183
2.179
2.175
2.171
2.167
2.124
2.112
2.099
2.086
2.039
2.025
2.013
1.985
1.971
1.959
1.744
1.740
1.736
1.670
1.614
1.588
1.566
1.553
1.547
0.000
Ph
O
O
O
(^{1}H NMR, 600 MHz, $CDCl_3$)
1.92
0.99
2.08
1.00
1.10
2.09
1.00
1.99
2.01
2.11
2.16
2.93
3.10
3.14
2.03
f1 (ppm)

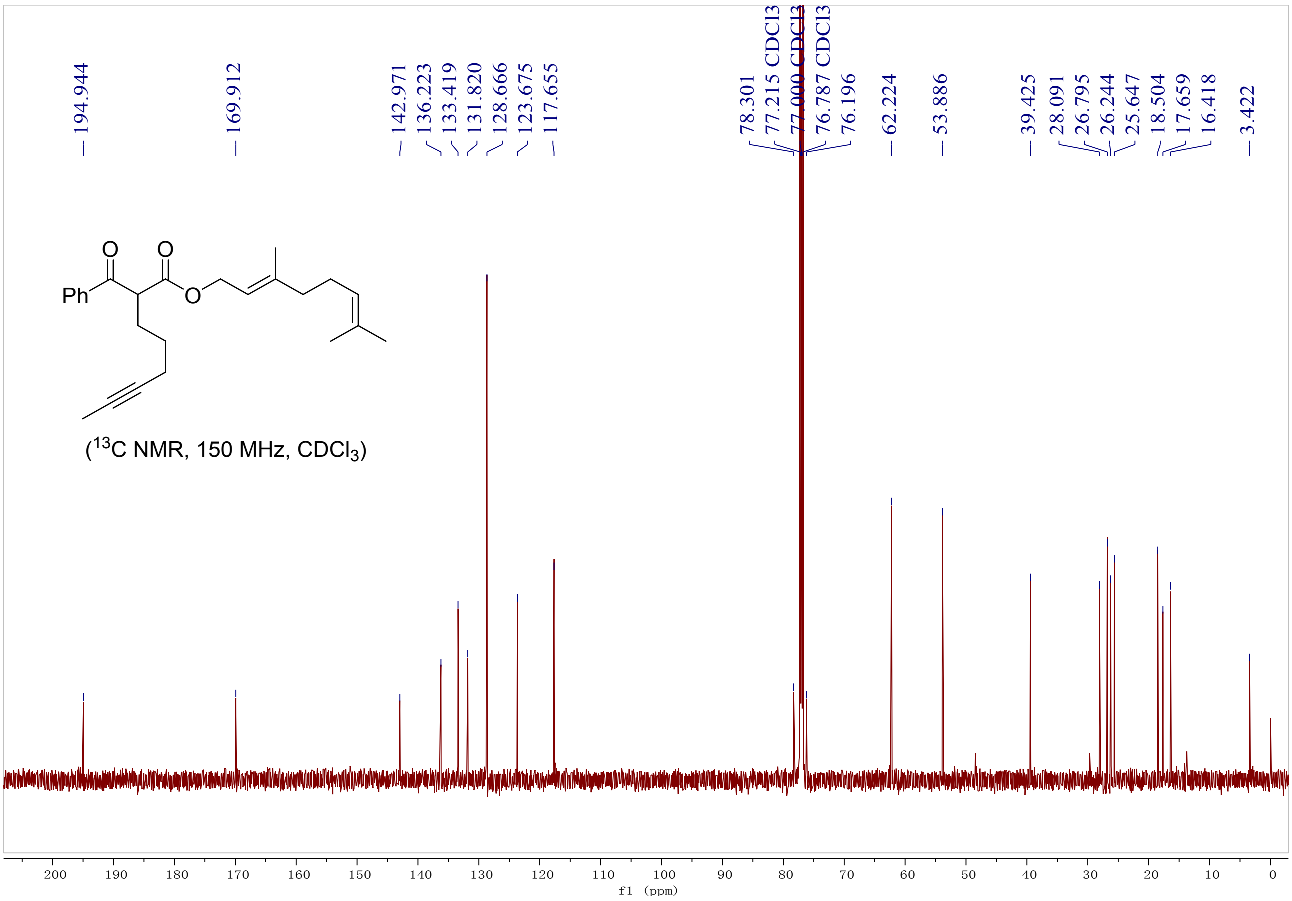
194.944
169.912
142.971
136.223
133.419
131.820
128.666
123.675
117.655
78.301
77.215 CDCl3
77.000 CDCl3
76.787 CDCl3
76.196
62.224
53.886
39.425
28.091
26.795
26.244
25.647
18.504
17.659
16.418
3.422
Ph
O
O
O
(^{13}C NMR, 150 MHz, $CDCl_3$)
f1 (ppm)

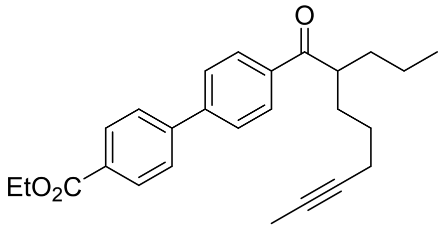

Ethyl 4'-(2-propyloct-6-ynoyl)-[1,1'-biphenyl]-4-carboxylate (3bac): colorless oil, 78 mg, 83%, Eluent: PE/EA = 20/1.

^{1}H NMR (400 MHz, $CDCl_3$, TMS): δ 8.15 (d, J = 8.4 Hz, 2H), 8.06 (d, J = 8.4 Hz, 2H), 7.76-7.66 (m, 4H), 4.42 (q, J = 7.2 Hz, 2H), 3.50 (tt, J = 7.8, 5.4 Hz, 1H), 2.17-2.06 (m, 2H), 1.92-1.73 (m, 5H), 1.67-1.59 (m, 2H), 1.54-1.40 (m, 6H), 1.35-1.28 (m, 2H), 0.90 (t, J = 7.2 Hz, 3H). **^{13}C NMR** (150 MHz, $CDCl_3$, TMS): δ 3.4, 14.26, 14.34, 18.8, 20.8, 26.9, 31.5, 34.7, 45.5, 61.1, 75.9, 78.7, 127.2, 127.5, 128.9, 130.2, 136.9, 144.1, 144.3, 166.3, 203.9. **IR** (EtOH): ν 2977, 2898, 2219, 1683, 1644, 1531, 1476, 1382, 1352, 1223, 1086, 1044, 878, 777, 682 cm^{-1}. **HRMS** (ESI) m/z: $[M+Na]^+$ Calcd. for $C_{26}H_{30}O_3Na$ 413.2087; found 413.2094.

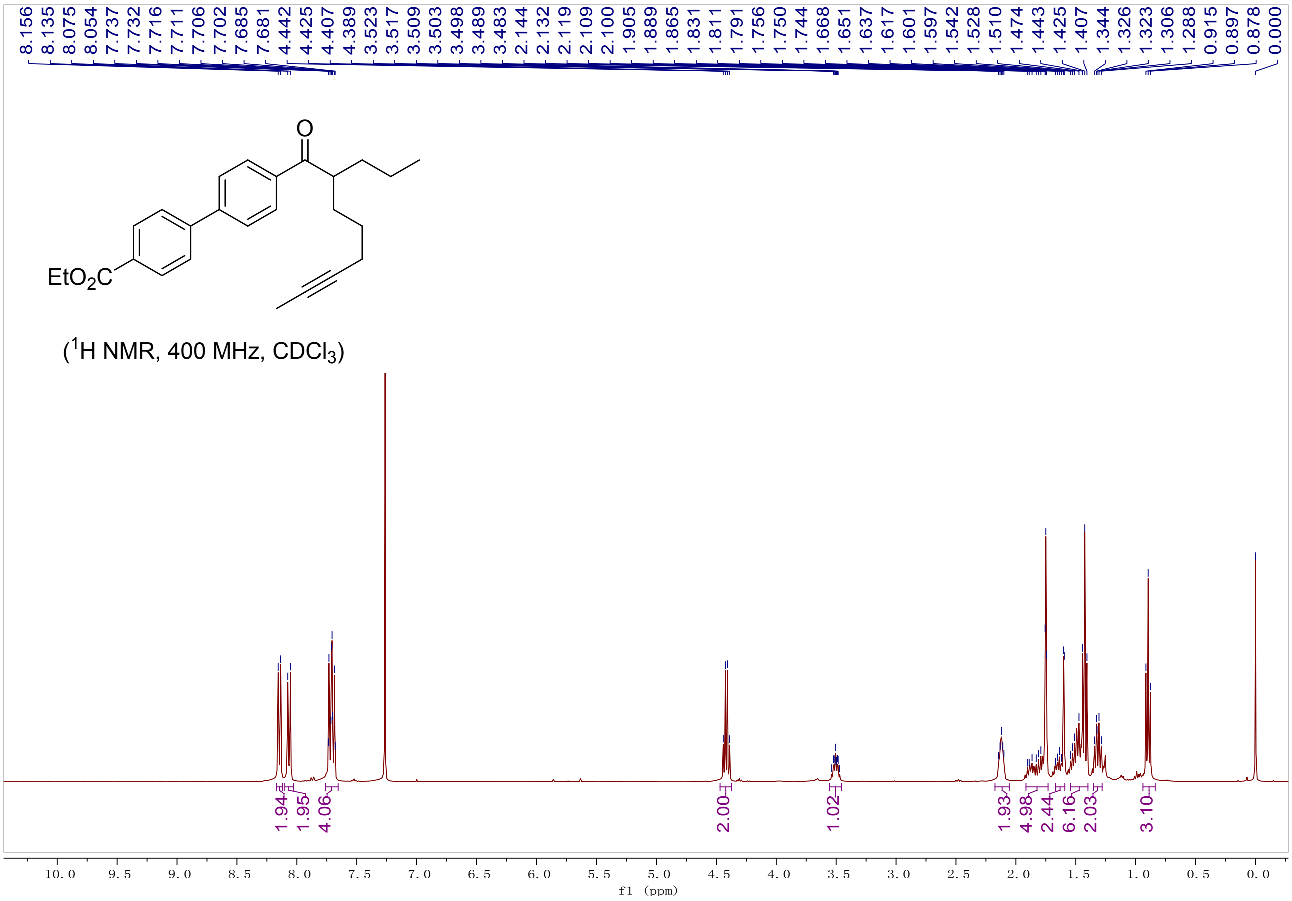
8.156 8.135 8.075 8.054 7.737 7.732 7.716 7.711 7.706 7.702 7.685 7.681 4.442 4.425 4.407 4.389 3.523 3.517 3.509 3.503 3.498 3.489 3.483 2.144 2.132 2.119 2.109 2.100 1.905 1.889 1.865 1.831 1.811 1.791 1.756 1.750 1.744 1.668 1.651 1.637 1.617 1.601 1.597 1.542 1.528 1.510 1.474 1.443 1.425 1.407 1.344 1.326 1.323 1.306 1.288 0.915 0.897 0.878 0.000
EtO2C
O
(1H NMR, 400 MHz, CDCl3)
1.94 1.95 4.06 2.00 1.02 1.93 4.98 2.44 6.16 2.03 3.10
10.0 9.5 9.0 8.5 8.0 7.5 7.0 6.5 6.0 5.5 5.0 4.5 4.0 3.5 3.0 2.5 2.0 1.5 1.0 0.5 0.0
f1 (ppm)

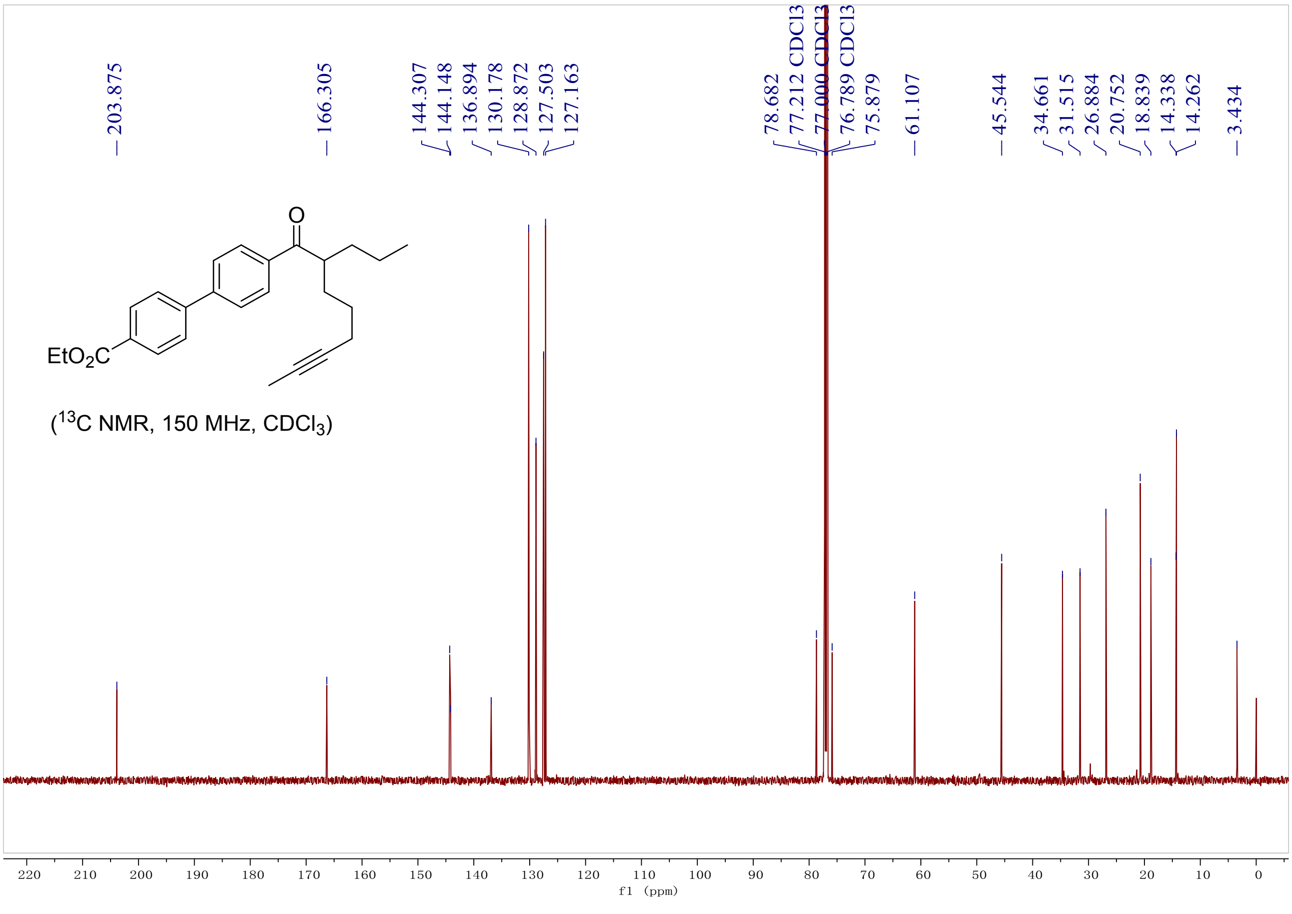
203.875
166.305
144.307 144.148 136.894 130.178 128.872 127.503 127.163
78.682 77.212 CDCl3 77.000 CDCl3 76.789 CDCl3 75.879
61.107
45.544
34.661 31.515 26.884 20.752 18.839 14.338 14.262
3.434
EtO2C
O
(13C NMR, 150 MHz, CDCl3)
220 210 200 190 180 170 160 150 140 130 120 110 100 90 80 70 60 50 40 30 20 10 0
f1 (ppm)

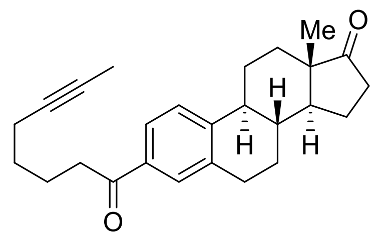

(8*R*,9*S*,13*S*,14*S*)-13-methyl-3-(oct-6-ynoyl)-6,7,8,9,11,12,13,14,15,16-decahydro-17*H*-cyclopenta[*a*]phenanthren-17-one (3bad): colorless solid, m.p. 226-228 °C, 59 mg, 78%, Eluent: PE/EA = 20/1.

^{1}H NMR (400 MHz, $CDCl_3$, TMS): δ 7.77-7.69 (m, 2H), 7.38 (d, *J* = 8.2 Hz, 1H), 3.03-2.92 (m, 4H), 2.56-2.44 (m, 2H), 2.38-2.30 (m, 1H), 2.24-2.14 (m, 3H), 2.12-1.96 (m, 4H), 1.87-1.76 (m, 5H), 1.71-1.44 (m, 7H), 0.92 (s, 3H). **^{13}C NMR** (150 MHz, $CDCl_3$, TMS): δ 3.5, 13.8, 18.6, 21.6, 23.6, 25.6, 26.3, 28.6, 29.3, 31.5, 35.8, 37.8, 38.0, 44.7, 47.9, 50.5, 75.8, 78.8, 125.5, 128.7, 134.7, 136.9, 145.2, 200.1, 220.6. **IR** (EtOH): ν 2938, 2928, 2856, 2231, 1684, 1584, 1486, 1459, 1430, 1283, 1256, 1168, 1044, 867, 785, 686 cm^{-1}. **HRMS** (ESI) m/z: $[M+Na]^+$ Calcd. for $C_{26}H_{32}O_2Na$ 399.2295; found 399.2301.

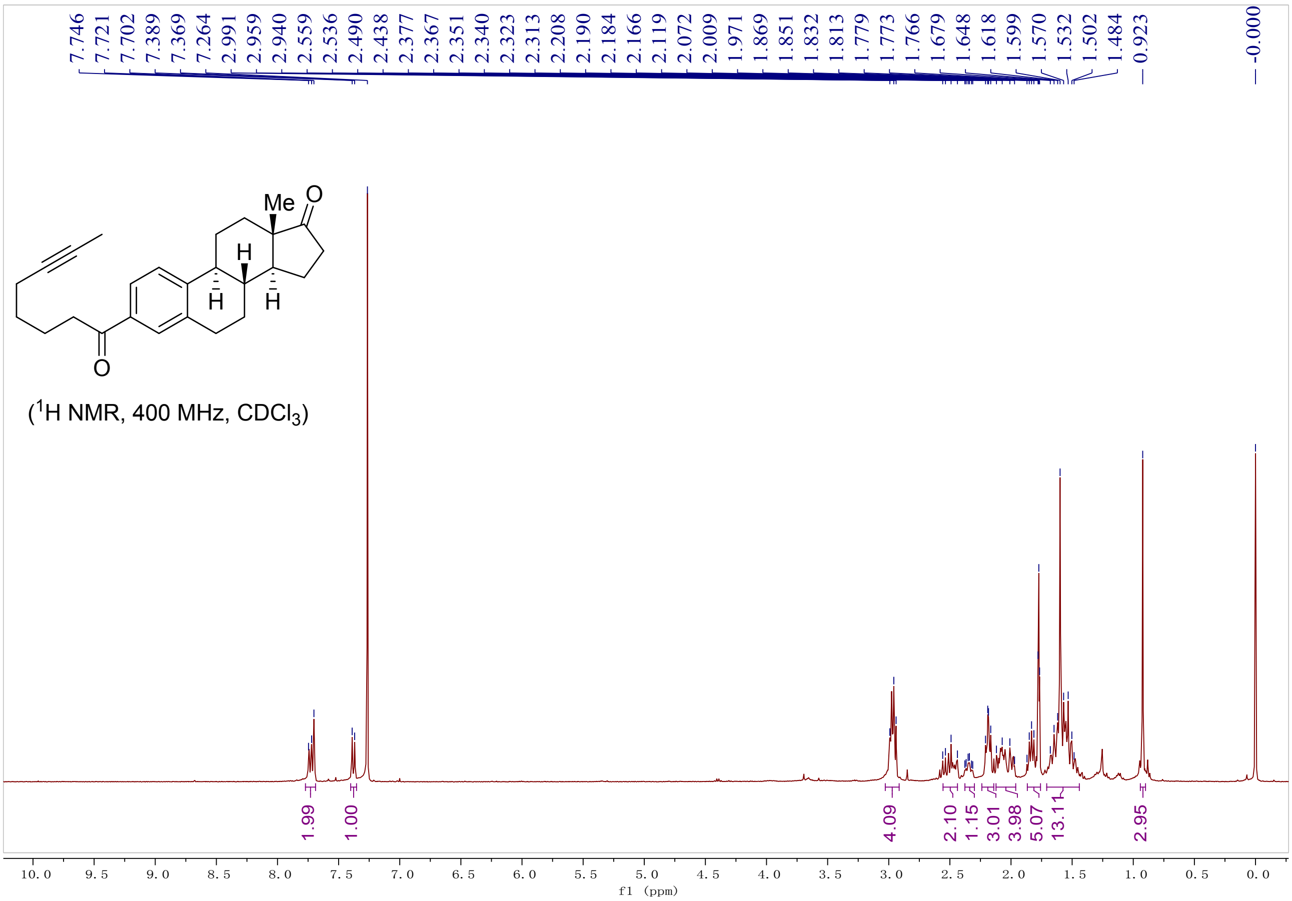
7.746
7.721
7.702
7.389
7.369
7.264
2.991
2.959
2.940
2.559
2.536
2.490
2.438
2.377
2.367
2.351
2.340
2.323
2.313
2.208
2.190
2.184
2.166
2.119
2.072
2.009
1.971
1.869
1.851
1.832
1.813
1.779
1.773
1.766
1.679
1.648
1.618
1.599
1.570
1.532
1.502
1.484
0.923
-0.000
Me
O
H
H
H
O
(1H NMR, 400 MHz, CDCl3)
1.99
1.00
4.09
2.10
1.15
3.01
3.98
5.07
13.11
2.95
f1 (ppm)

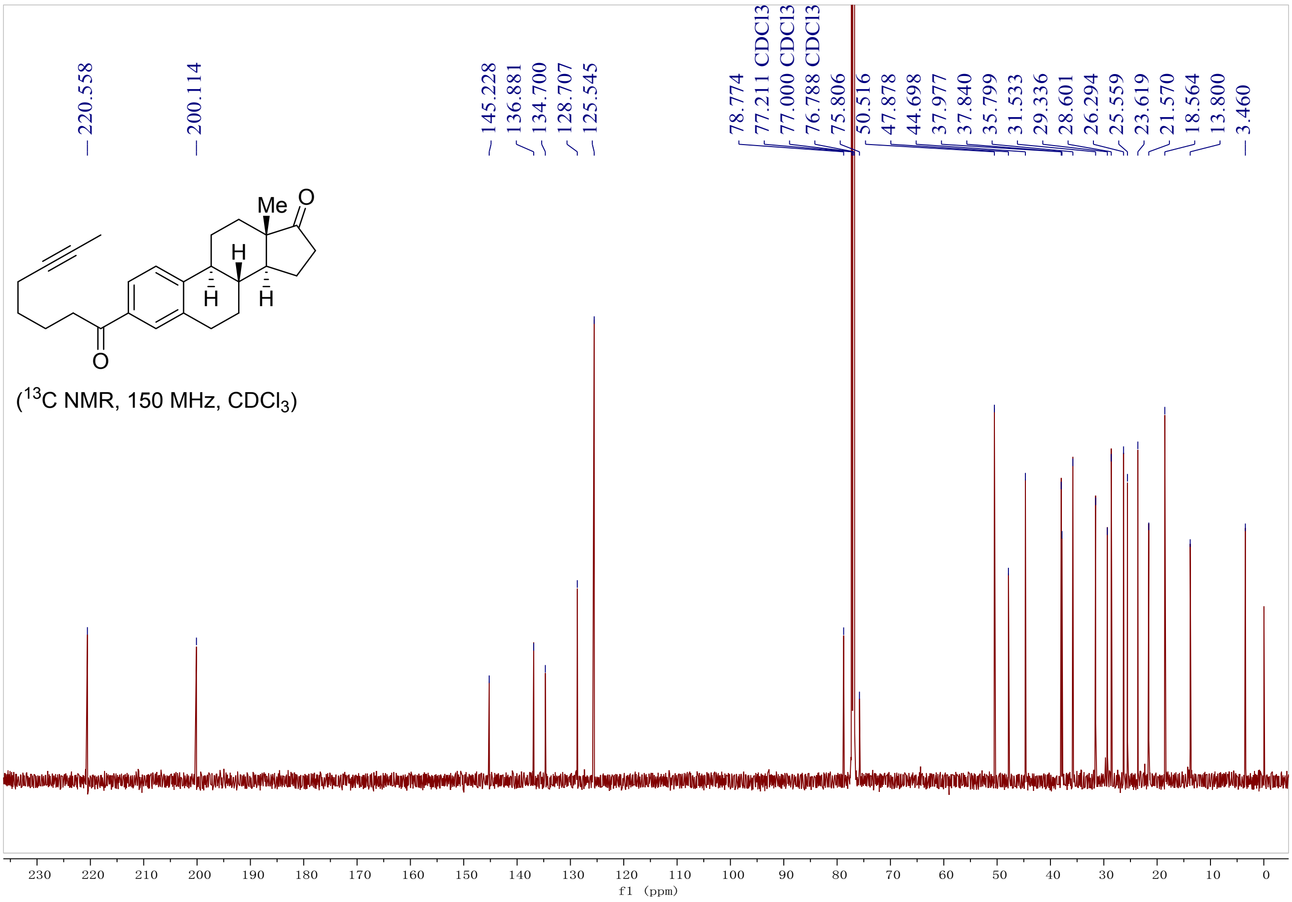
220.558
200.114
145.228
136.881
134.700
128.707
125.545
78.774
77.211 CDCl3
77.000 CDCl3
76.788 CDCl3
75.806
50.516
47.878
44.698
37.977
37.840
35.799
31.533
29.336
28.601
26.294
25.559
23.619
21.570
18.564
13.800
3.460
Me
O
H
H
H
O
(13C NMR, 150 MHz, CDCl3)
f1 (ppm)

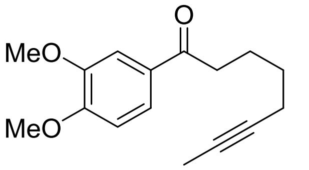

1-(3,4-dimethoxyphenyl)oct-6-yn-1-one (3bae): colorless oil, 46 mg, 88%, Eluent: PE/EA = 20/1. **^{1}H NMR** (400 MHz, $CDCl_3$, TMS): δ 7.62-7.52 (m, 2H), 6.89 (d, *J* = 8.4 Hz, 1H), 3.95 (s, 6H), 2.95 (t, *J* = 7.4 Hz, 2H), 2.19 (tq, *J* = 7.2, 2.6 Hz, 2H), 1.88-1.79 (m, 2H), 1.77 (t, *J* = 2.6 Hz, 3H), 1.60-1.54 (m, 2H). **^{13}C NMR** (100 MHz, $CDCl_3$, TMS): δ 3.5, 18.6, 23.8, 28.6, 37.6, 55.95, 56.04, 75.8, 78.8, 109.9, 110.1, 122.7, 130.2, 149.0, 153.1, 198.9. **IR** (EtOH): ν 2973, 2875, 2200, 1677, 1591, 1514, 1416, 1266, 1086, 1045, 1001, 879, 765, 623 cm^{-1}. **HRMS** (EI) m/z: $[M]^+$ Calcd. for $C_{16}H_{30}O_3$ 260.1407; found 260.1401.

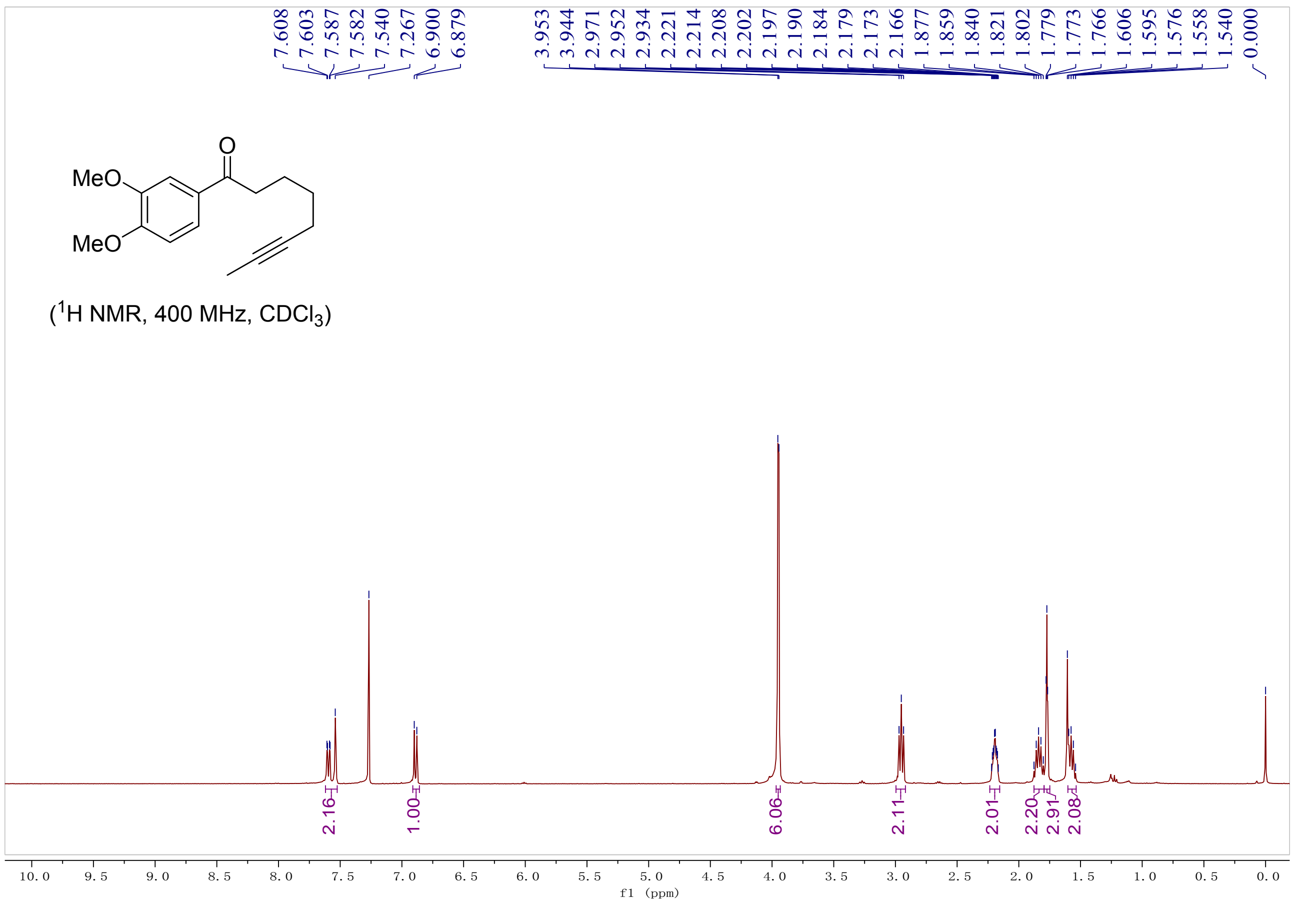
7.608 7.603 7.587 7.582 7.540 7.267 6.900 6.879
3.953 3.944 2.971 2.952 2.934 2.221 2.214 2.208 2.202 2.197 2.190 2.184 2.179 2.173 2.166 1.877 1.859 1.840 1.821 1.802 1.779 1.773 1.766 1.606 1.595 1.576 1.558 1.540 0.000
MeO
MeO
O
(1H NMR, 400 MHz, CDCl3)
2.16
1.00
6.06
2.11
2.01
2.20
2.91
2.08
10.0 9.5 9.0 8.5 8.0 7.5 7.0 6.5 6.0 5.5 5.0 4.5 4.0 3.5 3.0 2.5 2.0 1.5 1.0 0.5 0.0
f1 (ppm)

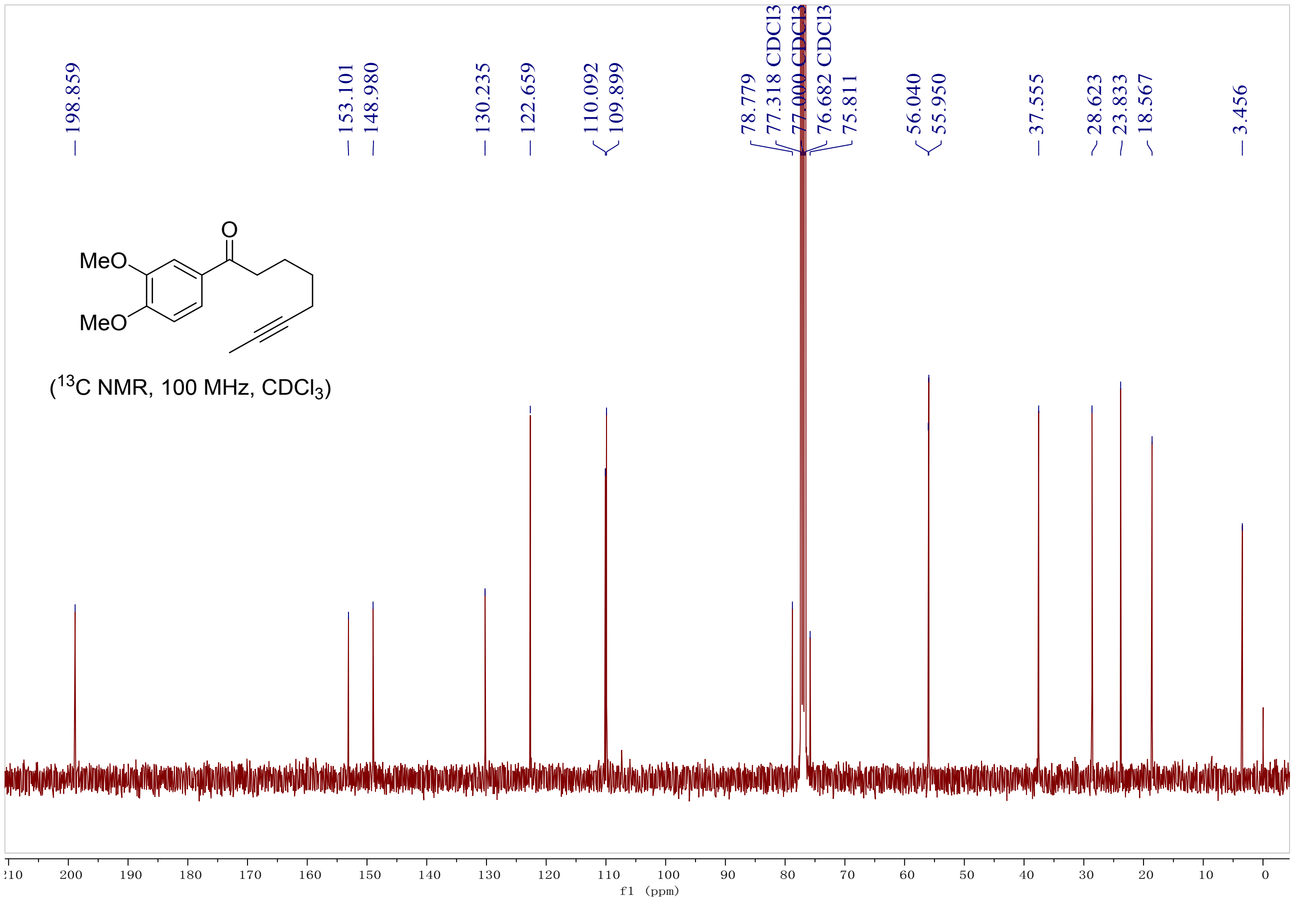
198.859
153.101 148.980
130.235 122.659
110.092 109.899
78.779 77.318 CDCl3 77.000 CDCl3 76.682 CDCl3 75.811
56.040 55.950
37.555
28.623 23.833 18.567
3.456
MeO
MeO
O
(13C NMR, 100 MHz, CDCl3)
210 200 190 180 170 160 150 140 130 120 110 100 90 80 70 60 50 40 30 20 10 0
f1 (ppm)

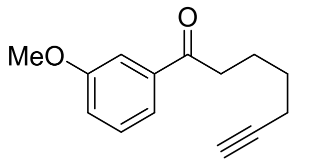

1-(3-methoxyphenyl)hept-6-yn-1-one (3baf): colorless oil, 149 mg, 69%, Eluent: PE/EA = 20/1. **^{1}H NMR** (600 MHz, $CDCl_3$, TMS): δ 7.54 (dt, J = 7.6, 1.2 Hz, 1H), 7.49 (dd, J = 2.6, 1.6 Hz, 1H), 7.37 (t, J = 8.0 Hz, 1H), 7.11 (dd, J = 8.2, 2.6 Hz, 1H), 3.86 (s, 3H), 2.99 (t, J = 7.2 Hz, 2H), 2.26 (td, J = 7.2, 2.8 Hz, 2H), 1.96 (t, J = 2.6 Hz, 1H), 1.90-1.84 (m, 2H), 1.67-1.60 (m, 2H). **^{13}C NMR** (150 MHz, $CDCl_3$, TMS): δ 18.3, 23.4, 28.0, 38.0, 55.4, 68.6, 84.1, 112.3, 119.4, 120.7, 129.6, 138.3, 159.8, 199.8. **IR** (EtOH): ν 3291, 2940, 2837, 2115, 1680, 1583, 1455, 1429, 1254, 1166, 1043, 870, 781, 749, 683, 631 cm^{-1}. **HRMS** (EI) m/z: $[M]^+$ Calcd. for $C_{14}H_{16}O_2$ 216.1145; found 216.1144.

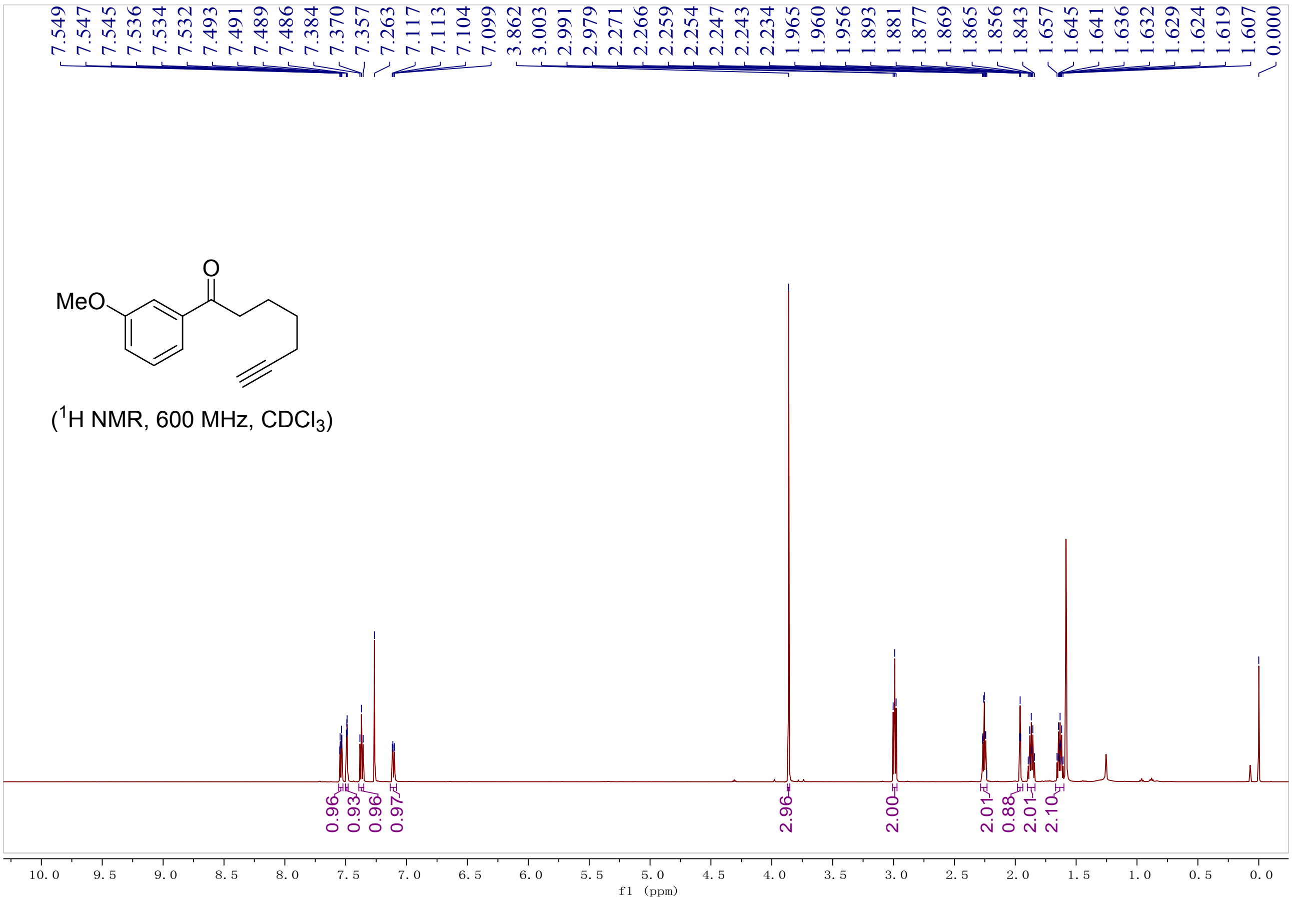
7.549
7.547
7.545
7.536
7.534
7.532
7.493
7.491
7.489
7.486
7.384
7.370
7.357
7.263
7.117
7.113
7.104
7.099
3.862
3.003
2.991
2.979
2.271
2.266
2.259
2.254
2.247
2.243
2.234
1.965
1.960
1.956
1.893
1.881
1.877
1.869
1.865
1.856
1.843
1.657
1.645
1.641
1.636
1.632
1.629
1.624
1.619
1.607
0.000
MeO
O
(1H NMR, 600 MHz, CDCl3)
0.96
0.93
0.96
0.97
2.96
2.00
2.01
0.88
2.01
2.10
10.0
9.5
9.0
8.5
8.0
7.5
7.0
6.5
6.0
5.5
5.0
4.5
4.0
3.5
3.0
2.5
2.0
1.5
1.0
0.5
0.0
f1 (ppm)

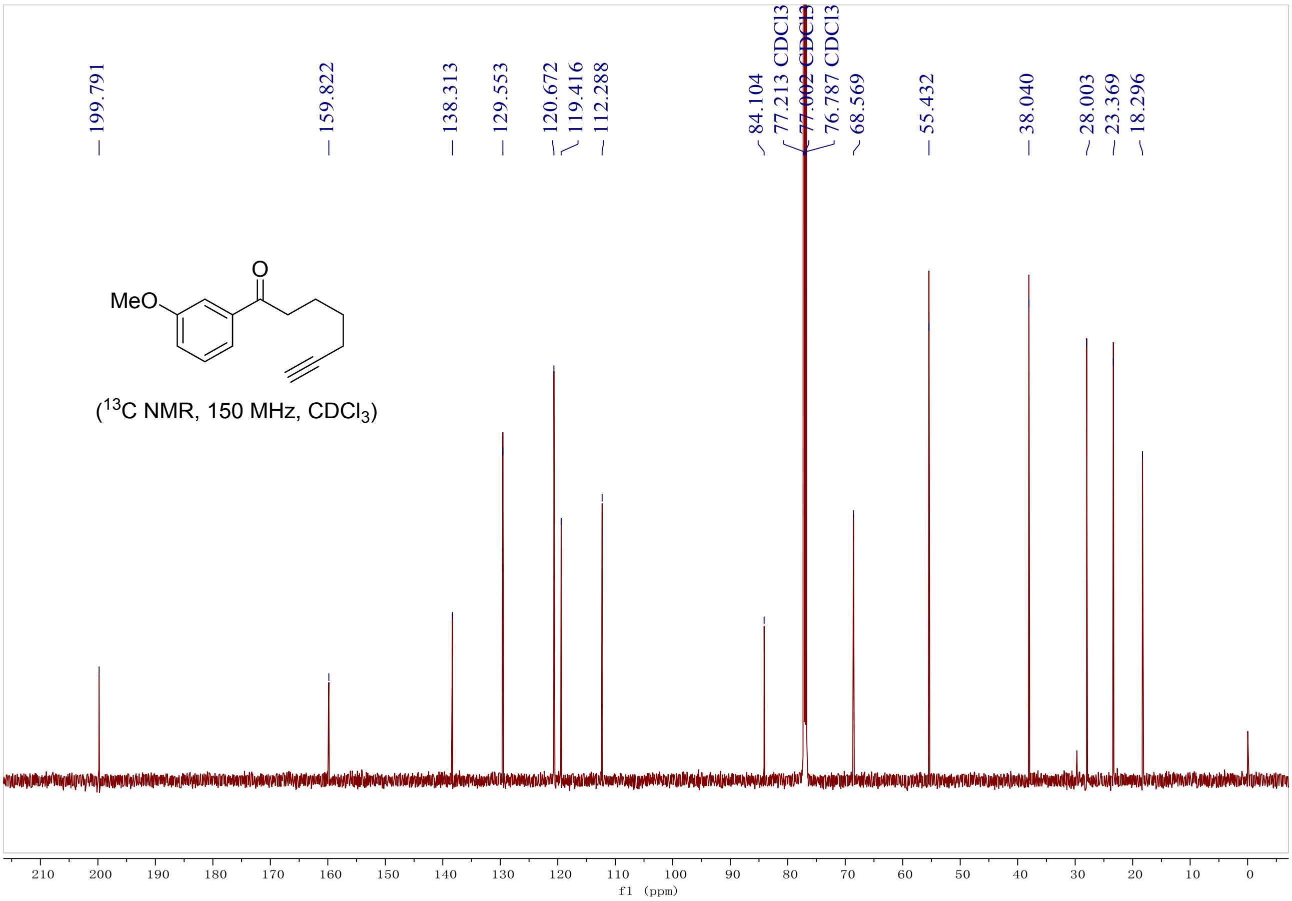
199.791
159.822
138.313
129.553
120.672
119.416
112.288
84.104
77.213 CDCl3
77.002 CDCl3
76.787 CDCl3
68.569
55.432
38.040
28.003
23.369
18.296
MeO
O
(13C NMR, 150 MHz, CDCl3)
210
200
190
180
170
160
150
140
130
120
110
100
90
80
70
60
50
40
30
20
10
0
f1 (ppm)

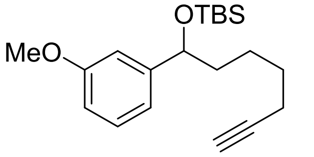

Tert-Butyl((1-(3-methoxyphenyl)hept-6-yn-1-yl)oxy)dimethylsilane (S3): colorless oil, 239 mg, 90%, Eluent: PE/EA = 20/1.

^{1}H NMR (400 MHz, $CDCl_3$, TMS): δ 7.20 (t, *J* = 7.8 Hz, 1H), 6.90-6.81 (m, 2H), 6.79-6.73 (m, 1H), 4.61 (dd, *J* = 7.4, 4.8 Hz, 1H), 3.80 (s, 3H), 2.20-2.12 (m, 2H), 1.92 (t, *J* = 2.6 Hz, 1H), 1.71-1.34 (m, 7H), 0.89 (s, 9H), 0.03 (s, 3H), -0.13 (s, 3H). **^{13}C NMR** (100 MHz, $CDCl_3$, TMS): δ -5.0, -4.6, 18.2, 18.4, 24.8, 25.8, 28.4, 40.4, 55.1, 68.2, 74.7, 84.5, 111.2, 112.3, 118.2, 128.9, 147.5, 159.4. **IR** (EtOH): ν 3310, 2974, 2925, 2892, 2214, 1652, 1380, 1346, 1307, 1224, 1150, 1005, 876, 799, 654 cm^{-1}. **HRMS** (EI) m/z: $[M+H]^+$ Calcd. for $C_{20}H_{33}O_2Si$ 333.2244; found 333.2242.

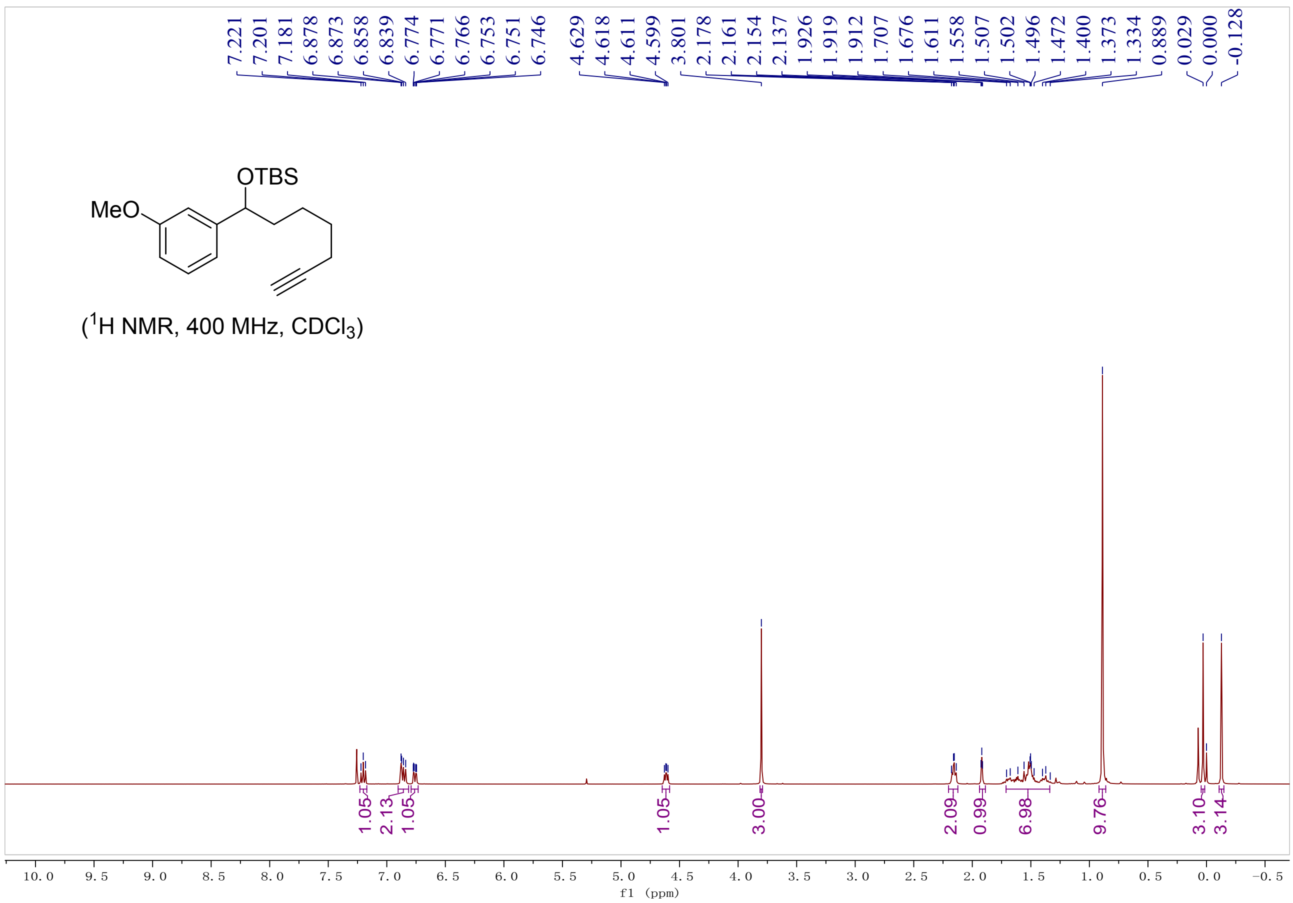

7.221 7.201 7.181 6.878 6.873 6.858 6.839 6.774 6.771 6.766 6.753 6.751 6.746 4.629 4.618 4.611 4.599 3.801 2.178 2.161 2.154 2.137 1.926 1.919 1.912 1.707 1.676 1.611 1.558 1.507 1.502 1.496 1.472 1.400 1.373 1.334 0.889 0.029 0.000 -0.128
OTBS
MeO
(^{1}H NMR, 400 MHz, $CDCl_3$)
1.05 2.13 1.05 1.05 3.00 2.09 0.99 6.98 9.76 3.10 3.14
f1 (ppm)

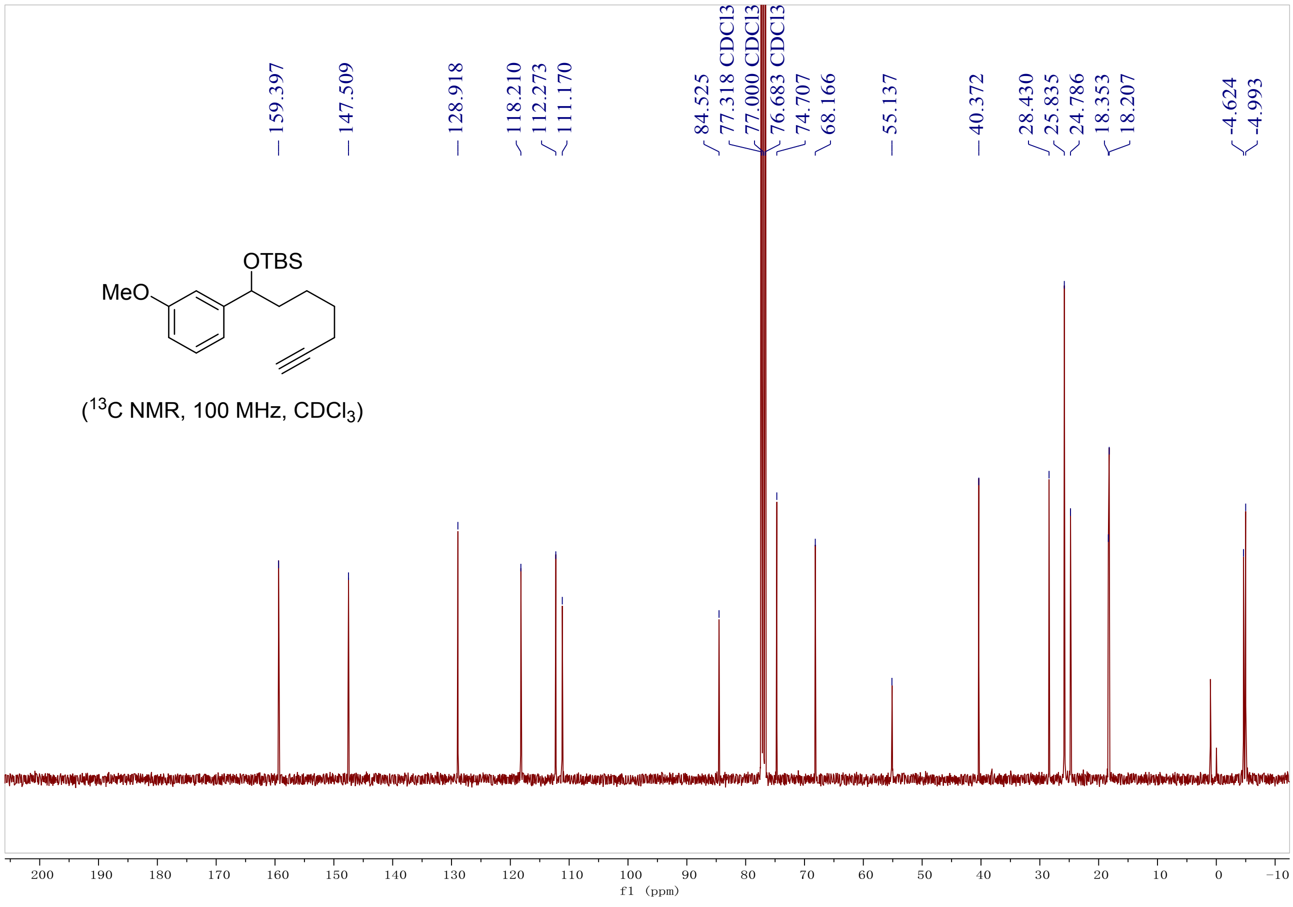

159.397 147.509 128.918 118.210 112.273 111.170 84.525 77.318 CDCl3 77.000 CDCl3 76.683 CDCl3 74.707 68.166 55.137 40.372 28.430 25.835 24.786 18.353 18.207 -4.624 -4.993
OTBS
MeO
(^{13}C NMR, 100 MHz, $CDCl_3$)
f1 (ppm)

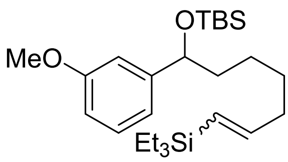

Tert-Butyl((1-(3-methoxyphenyl)-7-(triethylsilyl)hept-6-en-1-yl)oxy)dimethylsilane (4): colorless oil, 117 mg, *E*: *Z* = 2.5: 1, 87%, Eluent: PE/EA = 20/1.

^{1}H NMR (400 MHz, $CDCl_3$, TMS): δ 7.27-7.15 (m, 1H), 6.90-6.81 (m, 2H), 6.77-6.72 (m, 1H), 6.34 (dt, *J* = 14.2, 7.2 Hz, 0.13H), 5.99 (dt, *J* = 18.6, 6.4 Hz, 0.72H), 5.60 (dt, *J* = 3.2, 1.6 Hz, 0.1H), 5.51 (d, *J* = 18.6 Hz, 0.72H), 5.38 (d, *J* = 14.2 Hz, 0.13H), 5.27 (d, *J* = 2.8 Hz, 0.1H), 4.64-4.56 (m, 1H), 3.80 (s, 3H), 2.15-2.01 (m, 2H), 1.75-1.56 (m, 2H), 1.43-1.23 (m, 4H), 0.97-0.85 (m, 18H), 0.60-0.52 (m, 6H), 0.02 (s, 3H), -0.13 (s, 3H). **^{13}C NMR** (100 MHz, $CDCl_3$, TMS): δ -5.0, -4.6, 3.5, 4.7, 7.4, 7.5, 18.2, 25.1, 25.3, 25.8, 25.9, 28.8, 28.9, 36.2, 37.0, 40.8, 40.9, 55.06, 55.11, 74.8, 74.9, 111.2, 112.19, 112.24, 118.2, 125.0, 125.6, 128.9, 147.7, 148.5, 149.0, 150.1, 159.4. **IR** (EtOH): ν 2943, 2910, 2861, 1650, 1544, 1355, 1306, 1287, 1234, 1149, 1065, 1008, 886, 802, 683, 654. **HRMS** (ESI) m/z: $[M+Na]^+$ Calcd. for $C_{26}H_{48}O_2Si_2Na$ 471.3091; found 471.3092.

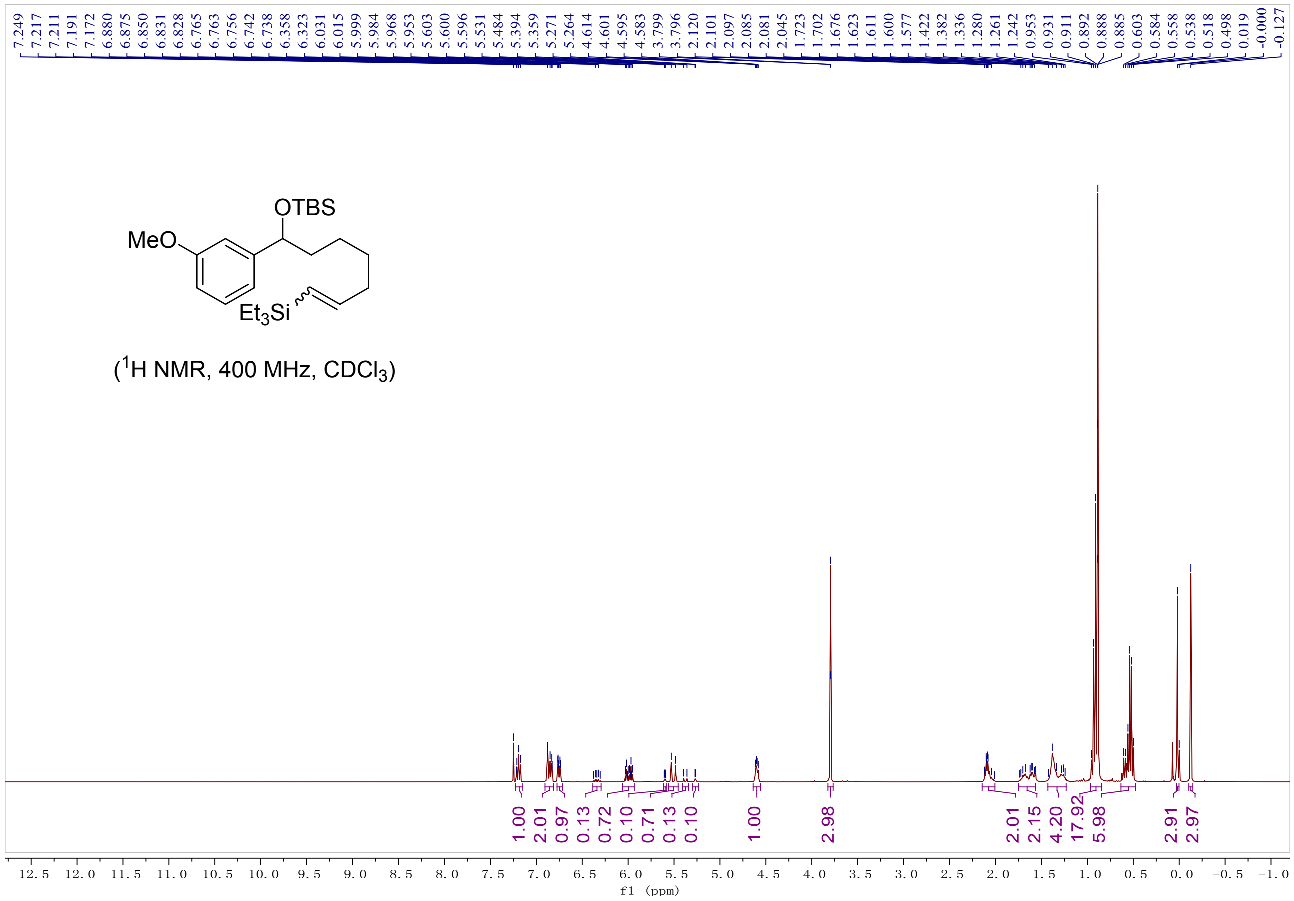
OTBS
MeO
Et3Si
(1H NMR, 400 MHz, CDCl3)
f1 (ppm)

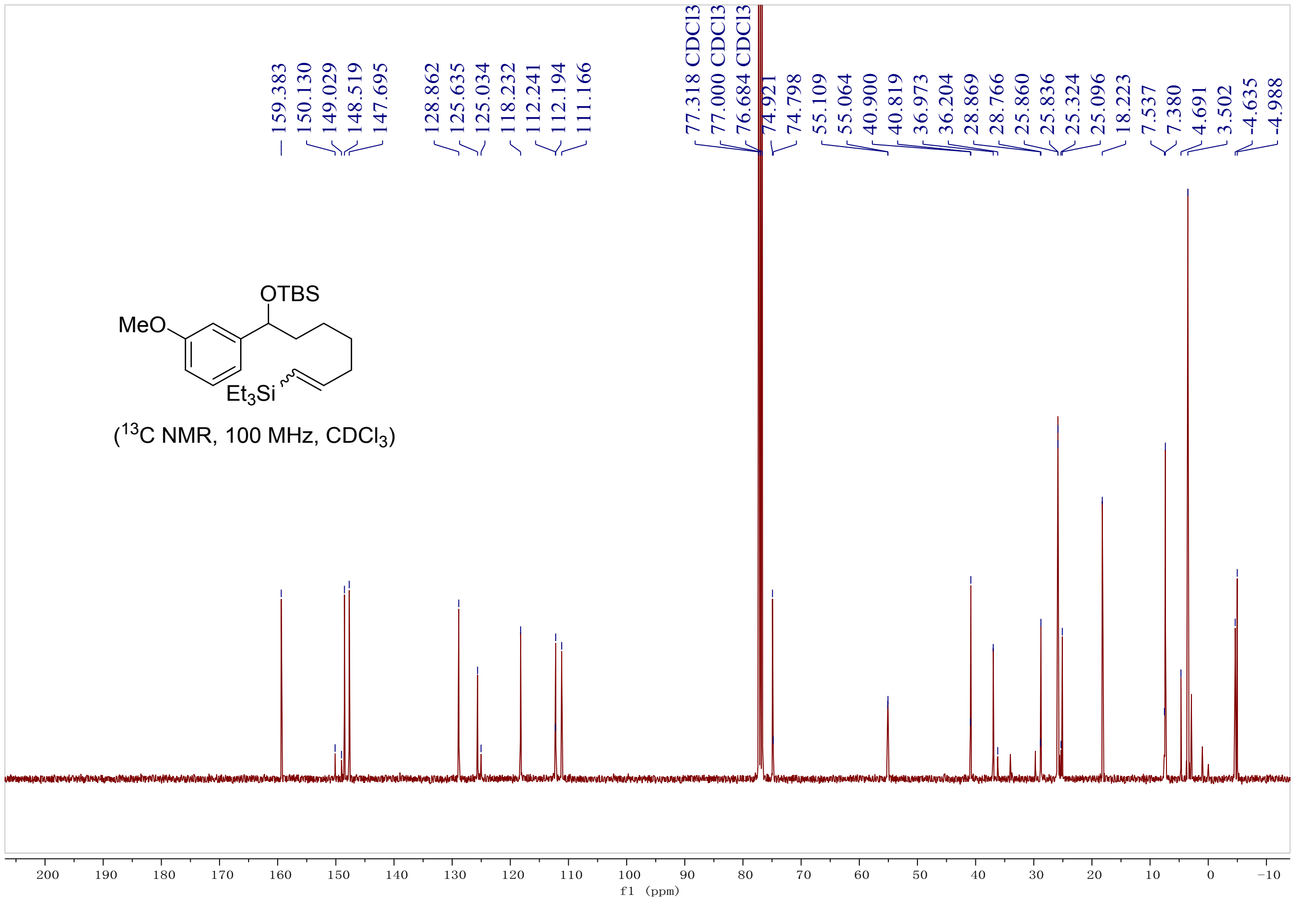
OTBS
MeO
Et3Si
(13C NMR, 100 MHz, CDCl3)
f1 (ppm)

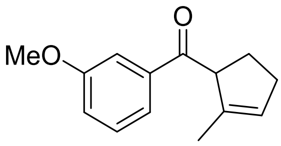

(3-methoxyphenyl)(2-methylcyclopent-2-en-1-yl)methanone (5): colorless oil, 58 mg, 90%, Eluent: PE/EA = 20/1.

^{1}H NMR (400 MHz, $CDCl_3$, TMS): δ 7.22 (t, *J* = 7.8 Hz, 1H), 6.95 (d, *J* = 7.8 Hz, 1H), 6.92 (t, *J* = 2.1 Hz, 1H), 6.79 (dd, *J* = 8.4, 2.6 Hz, 1H), 6.40-6.39 (m, 1H), 4.02-3.95 (m, 1H), 3.80 (s, 3H), 2.67-2.63 (m, 1H), 2.43-2.31 (m, 1H), 2.13-2.05 (m, 1H), 2.03 (s, 3H). **^{13}C NMR** (150 MHz, $CDCl_3$, TMS): δ 26.4, 28.1, 32.5, 55.2, 60.0, 111.4, 113.0, 118.3, 129.6, 130.8, 136.8, 141.6, 159.7, 211.1. **IR** (EtOH): ν 2963, 2935, 2857, 1688, 1660, 1499, 1337, 1233, 1176, 1010, 852, 745, 685, 652 cm^{-1}. **HRMS** (EI) m/z: $[M]^+$ Calcd. for $C_{14}H_{16}O_2$ 216.1145; found 216.1143.

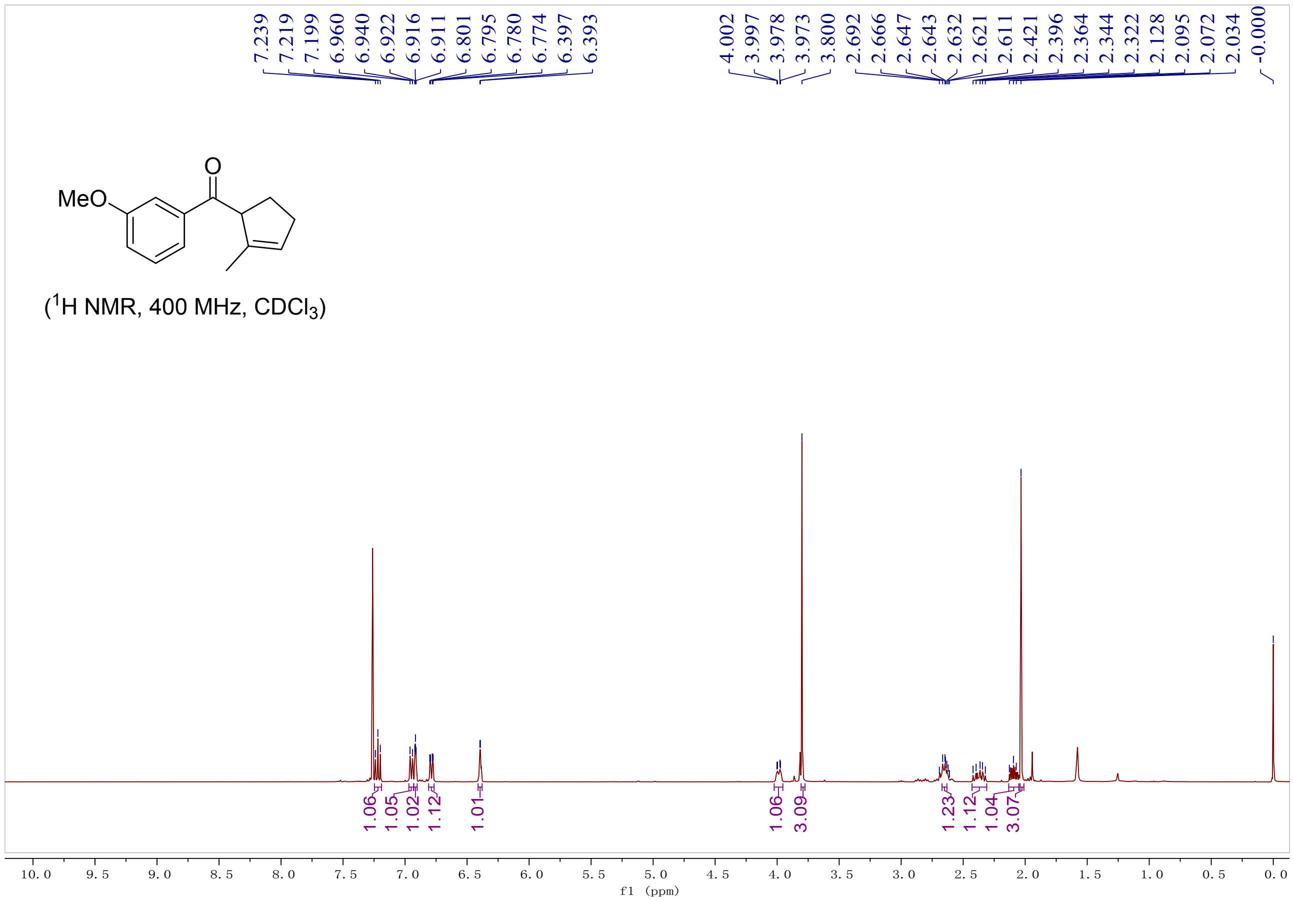

7.239
7.219
7.199
6.960
6.940
6.922
6.916
6.911
6.801
6.795
6.780
6.774
6.397
6.393
4.002
3.997
3.978
3.973
3.800
2.692
2.666
2.647
2.643
2.632
2.621
2.611
2.421
2.396
2.364
2.344
2.322
2.128
2.095
2.072
2.034
-0.000
MeO
O
(1H NMR, 400 MHz, CDCl3)
1.06
1.05
1.02
1.12
1.01
1.06
3.09
1.23
1.12
1.04
3.07
f1 (ppm)

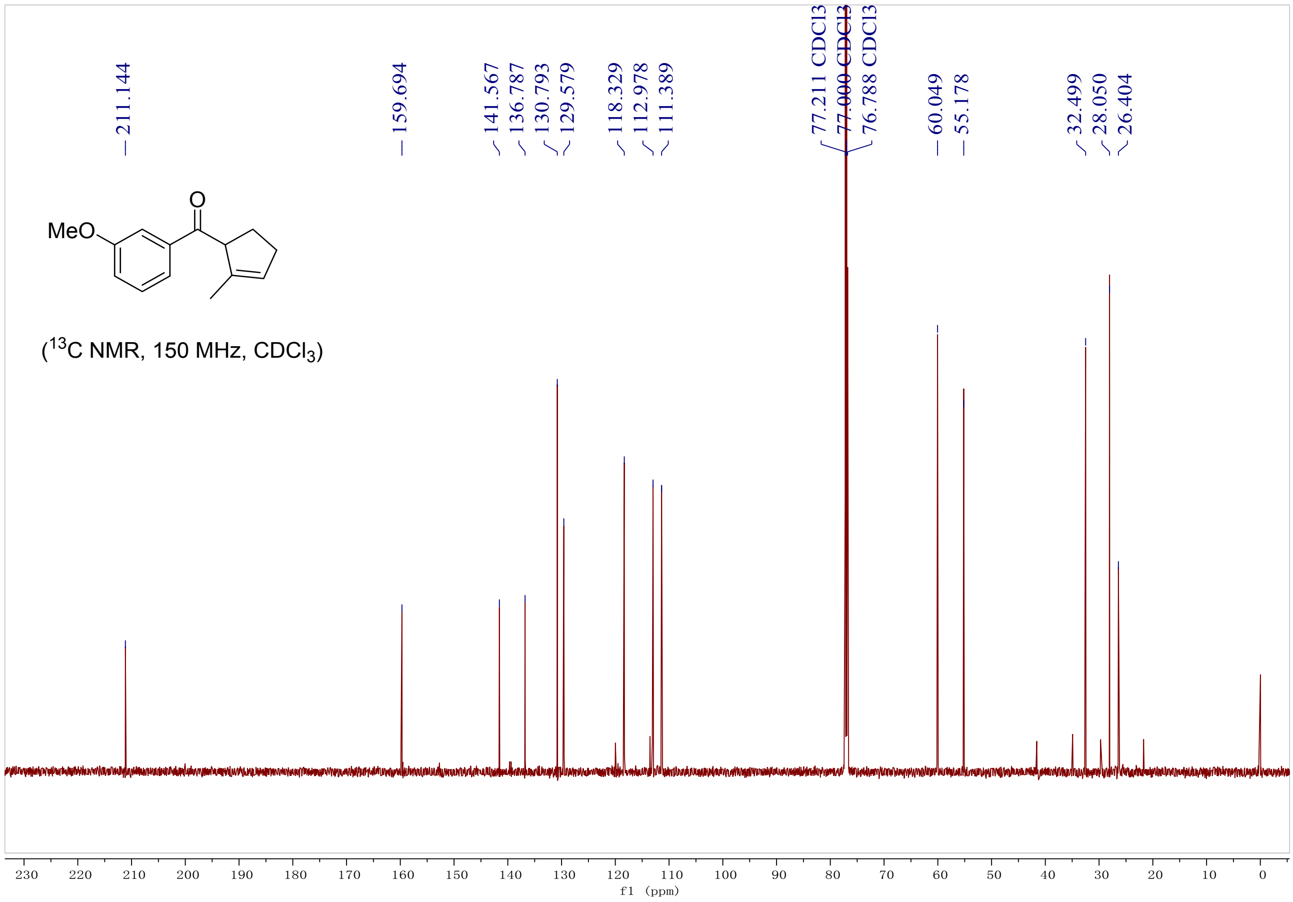

211.144
159.694
141.567
136.787
130.793
129.579
118.329
112.978
111.389
77.211 CDCl3
77.000 CDCl3
76.788 CDCl3
60.049
55.178
32.499
28.050
26.404
MeO
O
(13C NMR, 150 MHz, CDCl3)
f1 (ppm)

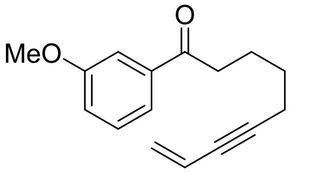

1-(3-methoxyphenyl)non-8-en-6-yn-1-one (6): colorless oil, 72 mg, 99%, Eluent: PE/EA = 20/1.

^{1}H NMR (400 MHz, $CDCl_3$, TMS): δ 7.58-7.50 (m, 1H), 7.52-7.46 (m, 1H), 7.36 (t, *J* = 8.0 Hz, 1H), 7.10 (dd, *J* = 8.0, 2.8 Hz, 1H), 5.77 (ddt, *J* = 17.6, 11.0, 2.2 Hz, 1H), 5.54 (dd, *J* = 17.6, 2.2 Hz, 1H), 5.37 (dd, *J* = 11.0, 2.2 Hz, 1H), 3.85 (s, 3H), 2.99 (t, *J* = 7.2 Hz, 2H), 2.37 (td, *J* = 7.0, 2.1 Hz, 2H), 1.91-1.81 (m, 2H), 1.68-1.58 (m, 2H). **^{13}C NMR** (100 MHz, $CDCl_3$, TMS): δ 19.1, 23.5, 28.1, 38.0, 55.4, 79.7, 90.5, 112.2, 117.5, 119.3, 120.6, 125.6, 129.5, 138.3, 159.8, 199.8. **IR** (EtOH): ν 3074, 2934, 2271, 1682, 1640, 1599, 1456, 1429, 1345, 1254, 1042, 992, 909, 872, 780, 685, 564 cm^{-1}. **HRMS** (ESI) m/z: $[M+Na]^+$ Calcd. for $C_{16}H_{18}NaO_2$ 265.1199; found 216.1205.

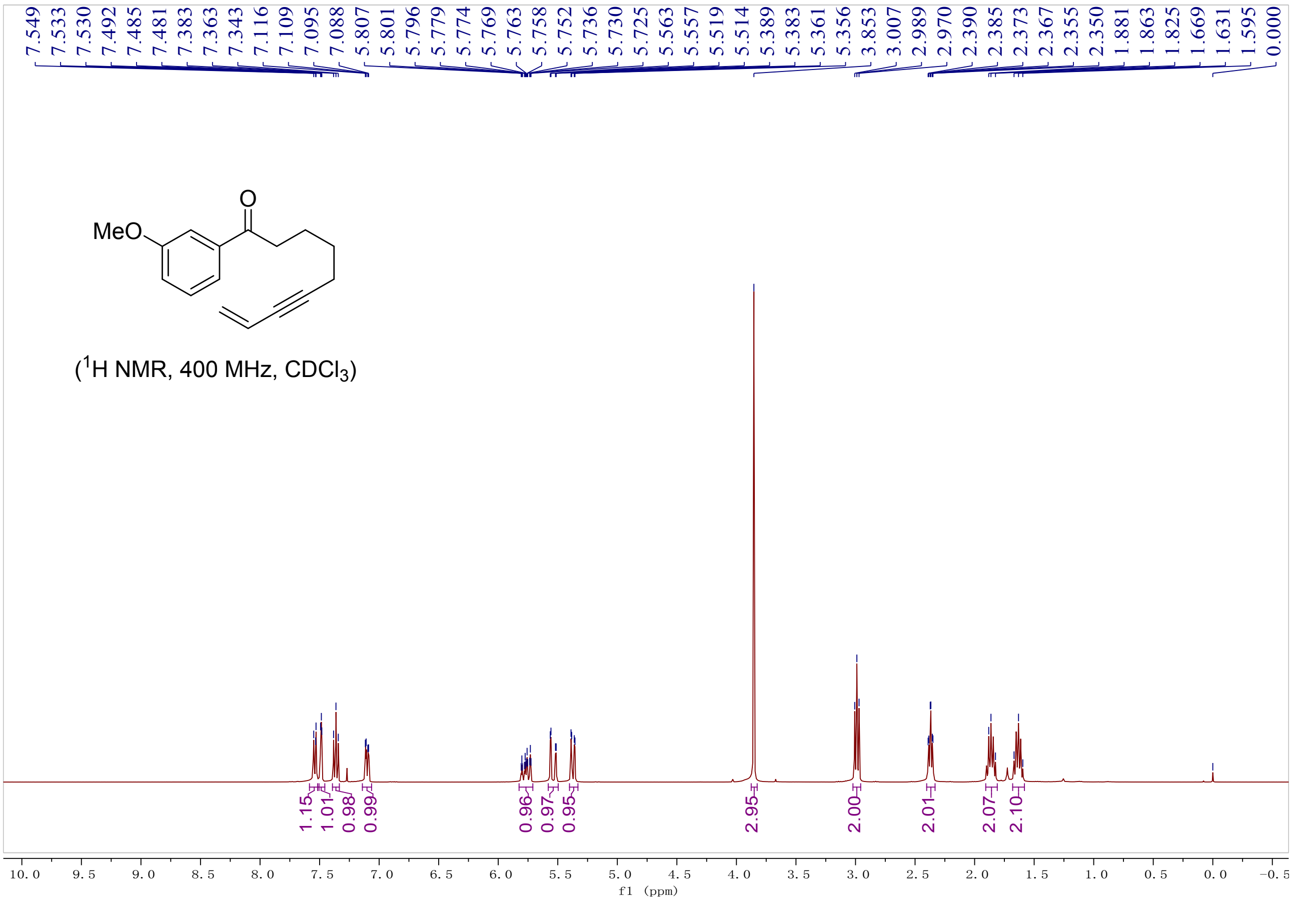
7.549
7.533
7.530
7.492
7.485
7.481
7.383
7.363
7.343
7.116
7.109
7.095
7.088
5.807
5.801
5.796
5.779
5.774
5.769
5.763
5.758
5.752
5.736
5.730
5.725
5.563
5.557
5.519
5.514
5.389
5.383
5.361
5.356
3.853
3.007
2.989
2.970
2.390
2.385
2.373
2.367
2.355
2.350
1.881
1.863
1.825
1.669
1.631
1.595
0.000
MeO
O
(¹H NMR, 400 MHz, CDCl₃)
1.15
1.01
0.98
0.99
0.96
0.97
0.95
2.95
2.00
2.01
2.07
2.10
f1 (ppm)

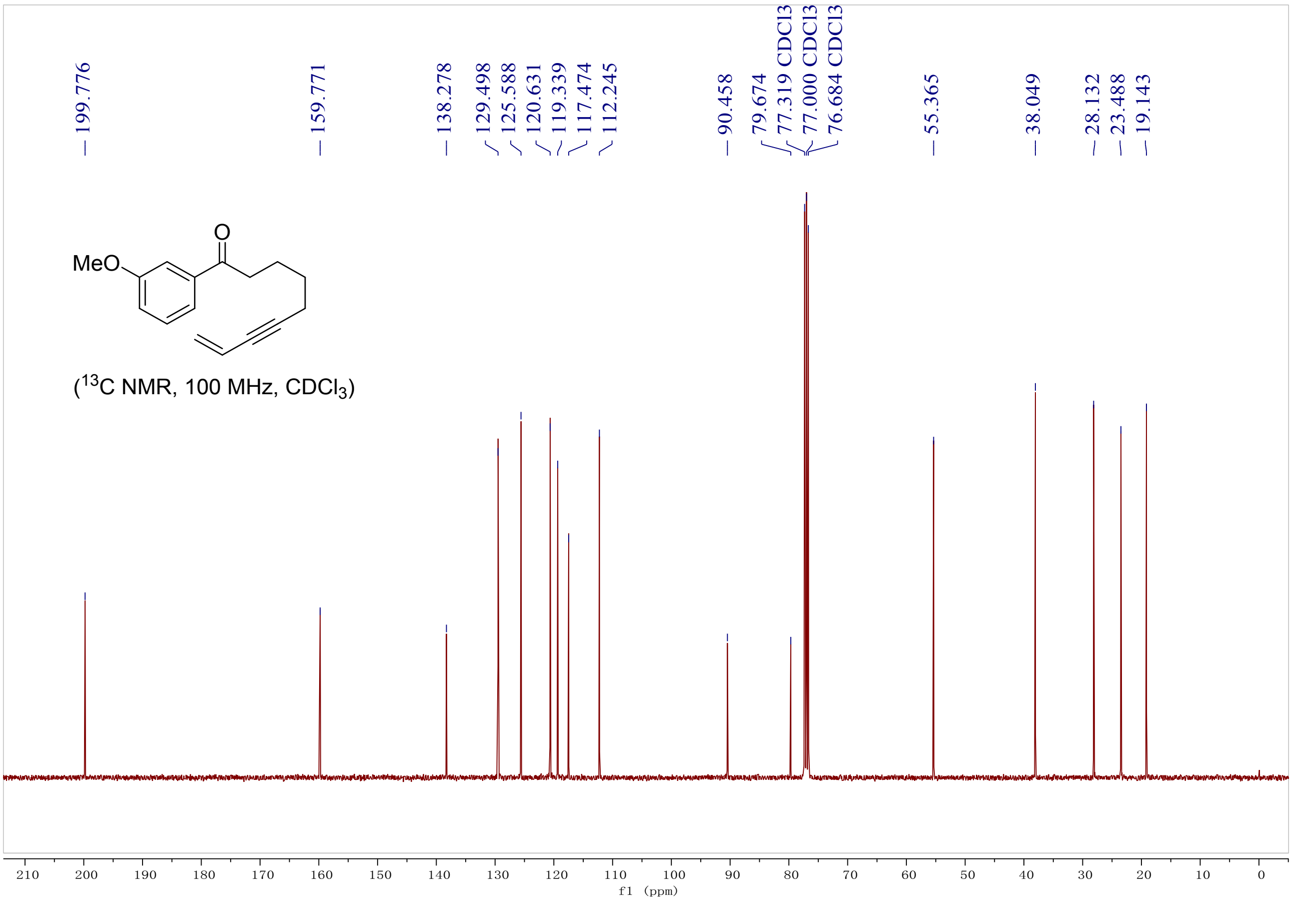
199.776
159.771
138.278
129.498
125.588
120.631
119.339
117.474
112.245
90.458
79.674
77.319 CDCl3
77.000 CDCl3
76.684 CDCl3
55.365
38.049
28.132
23.488
19.143
MeO
O
(¹³C NMR, 100 MHz, CDCl₃)
f1 (ppm)

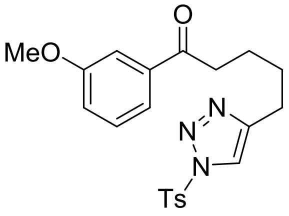

1-(3-methoxyphenyl)-5-(1-tosyl-1*H*-1,2,3-triazol-4-yl)pentan-1-one (7): white solid, m.p. 156-158 °C, 196 mg, 95%, Eluent: PE/EA = 20/1.

^{1}H NMR (400 MHz, $CDCl_3$, TMS): δ 7.98 (d, *J* = 8.4 Hz, 2H), 7.89 (s, 1H), 7.36 (t, *J* = 7.8 Hz, 3H), 7.11 (dd, *J* = 8.2, 2.8 Hz, 1H), 3.86 (s, 3H), 2.98 (t, *J* = 6.6 Hz, 2H), 2.77 (t, *J* = 7.0 Hz, 2H), 2.44 (s, 3H), 1.83-1.72 (m, 4H). **^{13}C NMR** (150 MHz, $CDCl_3$, TMS): δ 21.8, 23.6, 25.2, 28.4, 38.1, 55.4, 112.3, 119.4, 120.4, 120.6, 128.5, 129.6, 130.4, 133.2, 138.2, 147.0, 147.7, 159.8, 199.7. **IR** (EtOH): ν 2940, 2915, 2543, 2021, 1678, 1597, 1582, 1486, 1452, 1257, 1165, 1121, 1033, 1009, 817, 784, 682 cm^{-1}. **HRMS** (DART) m/z: $[M+H]^+$ Calcd. for $C_{21}H_{24}N_3O_4S$ 414.1482; found 414.1479.

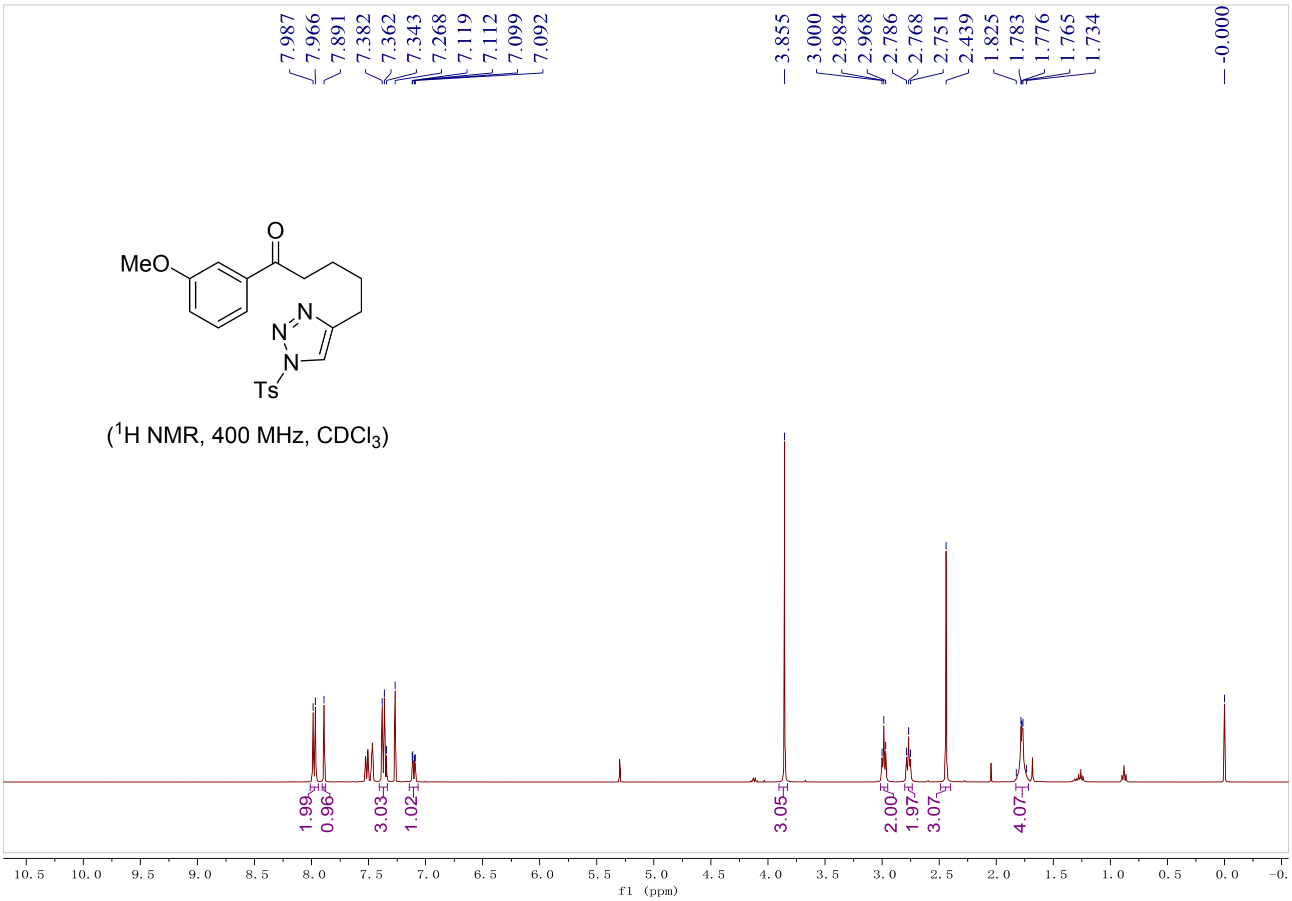

7.987
7.966
7.891
7.382
7.362
7.343
7.268
7.119
7.112
7.099
7.092
3.855
3.000
2.984
2.968
2.786
2.768
2.751
2.439
1.825
1.783
1.776
1.765
1.734
-0.000
MeO
O
N
N
N
Ts
(1H NMR, 400 MHz, CDCl3)
1.99
0.96
3.03
1.02
3.05
2.00
1.97
3.07
4.07
f1 (ppm)

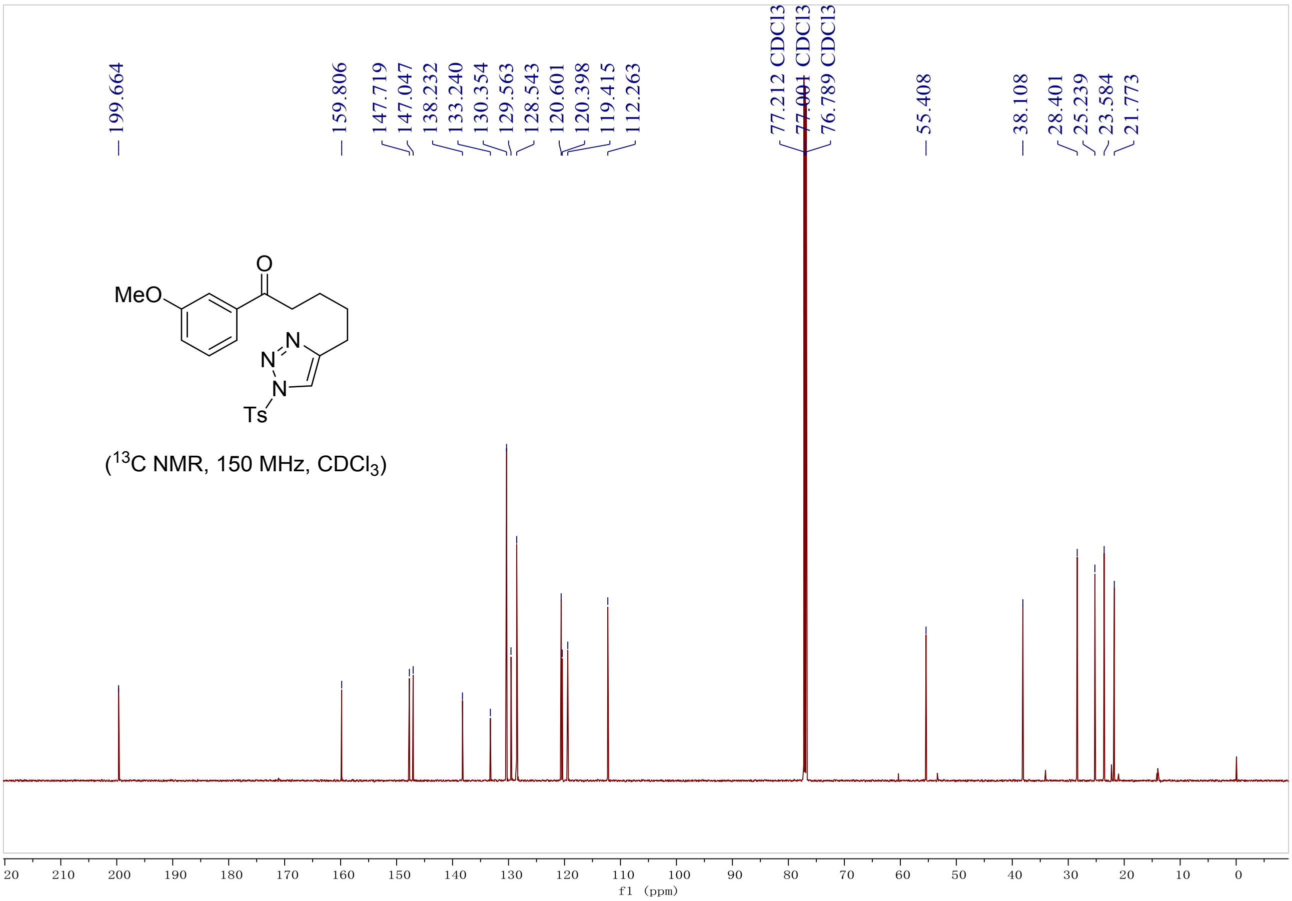

199.664
159.806
147.719
147.047
138.232
133.240
130.354
129.563
128.543
120.601
120.398
119.415
112.263
77.212 CDCl3
77.001 CDCl3
76.789 CDCl3
55.408
38.108
28.401
25.239
23.584
21.773
MeO
O
N
N
N
Ts
(13C NMR, 150 MHz, CDCl3)
f1 (ppm)

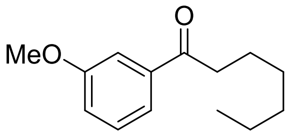

1-(3-methoxyphenyl)heptan-1-one (8): colorless oil, 65 mg, 98%, Eluent: PE/EA = 20/1.

^{1}H NMR (400 MHz, $CDCl_3$, TMS): δ 7.54 (dd, *J* = 7.8, 1.2 Hz, 1H), 7.52-7.46 (m, 1H), 7.36 (t, *J* = 7.8 Hz, 1H), 7.09 (dd, *J* = 8.4, 2.4 Hz, 1H), 3.85 (s, 3H), 2.94 (t, *J* = 7.4 Hz, 2H), 1.79-1.67 (m, 2H), 1.41-1.27 (m, 6H), 0.94-0.85 (m, 3H). **^{13}C NMR** (100 MHz, $CDCl_3$, TMS): δ 14.0, 22.5, 24.4, 29.0, 31.6, 38.7, 55.4, 112.3, 119.2, 120.7, 129.5, 138.5, 159.8, 200.4. **IR** (EtOH): ν 2998, 2967, 2934, 2870, 1681, 1514, 1429, 1264, 1167, 870, 753, 680 cm^{-1}. **HRMS** (EI) m/z: $[M]^+$ Calcd. for $C_{14}H_{20}O_2$ 220.1458; found 220.1456.

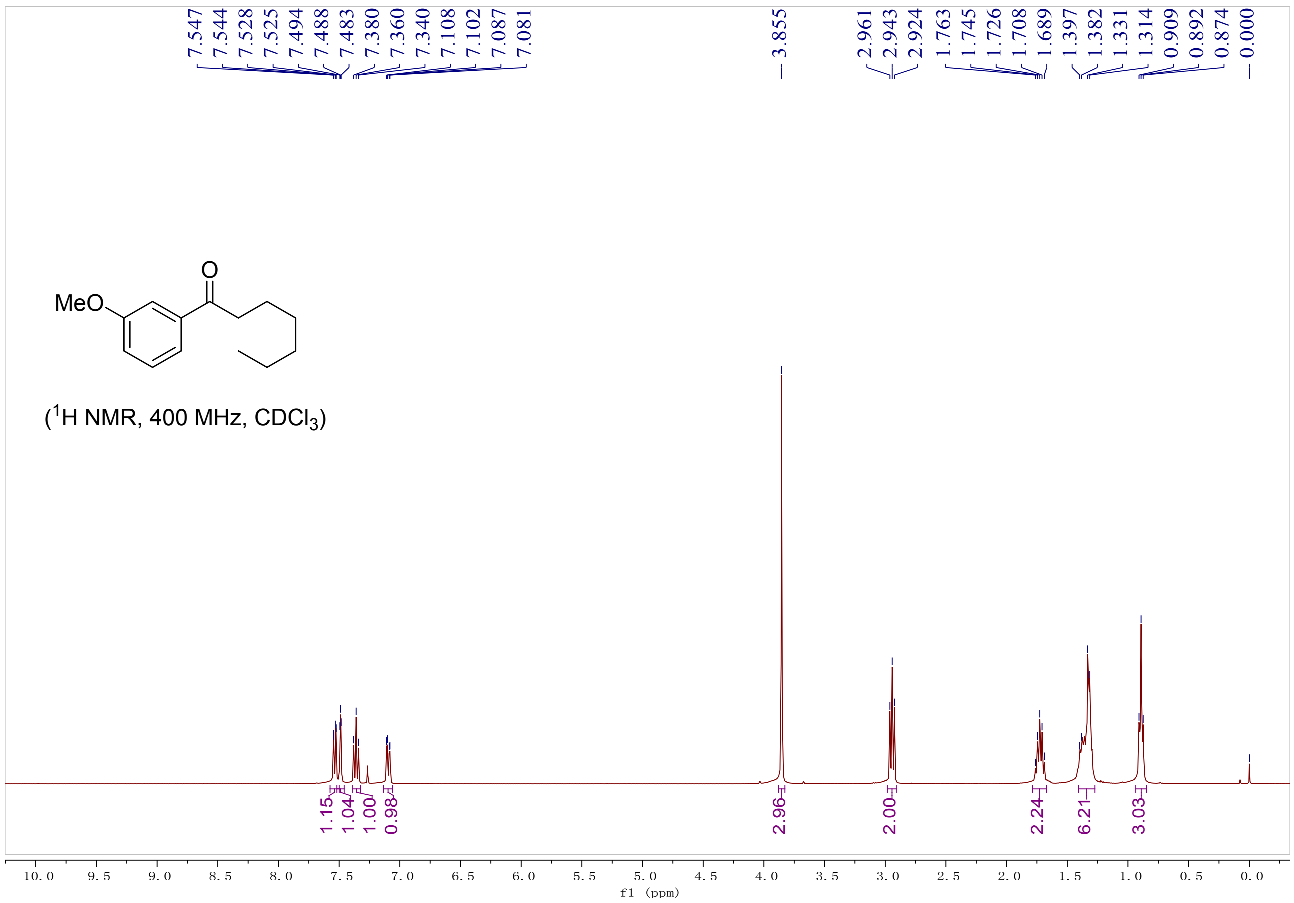
7.547
7.544
7.528
7.525
7.494
7.488
7.483
7.380
7.360
7.340
7.108
7.102
7.087
7.081
3.855
2.961
2.943
2.924
1.763
1.745
1.726
1.708
1.689
1.397
1.382
1.331
1.314
0.909
0.892
0.874
0.000
MeO
O
(1H NMR, 400 MHz, CDCl3)
1.15
1.04
1.00
0.98
2.96
2.00
2.24
6.21
3.03
f1 (ppm)

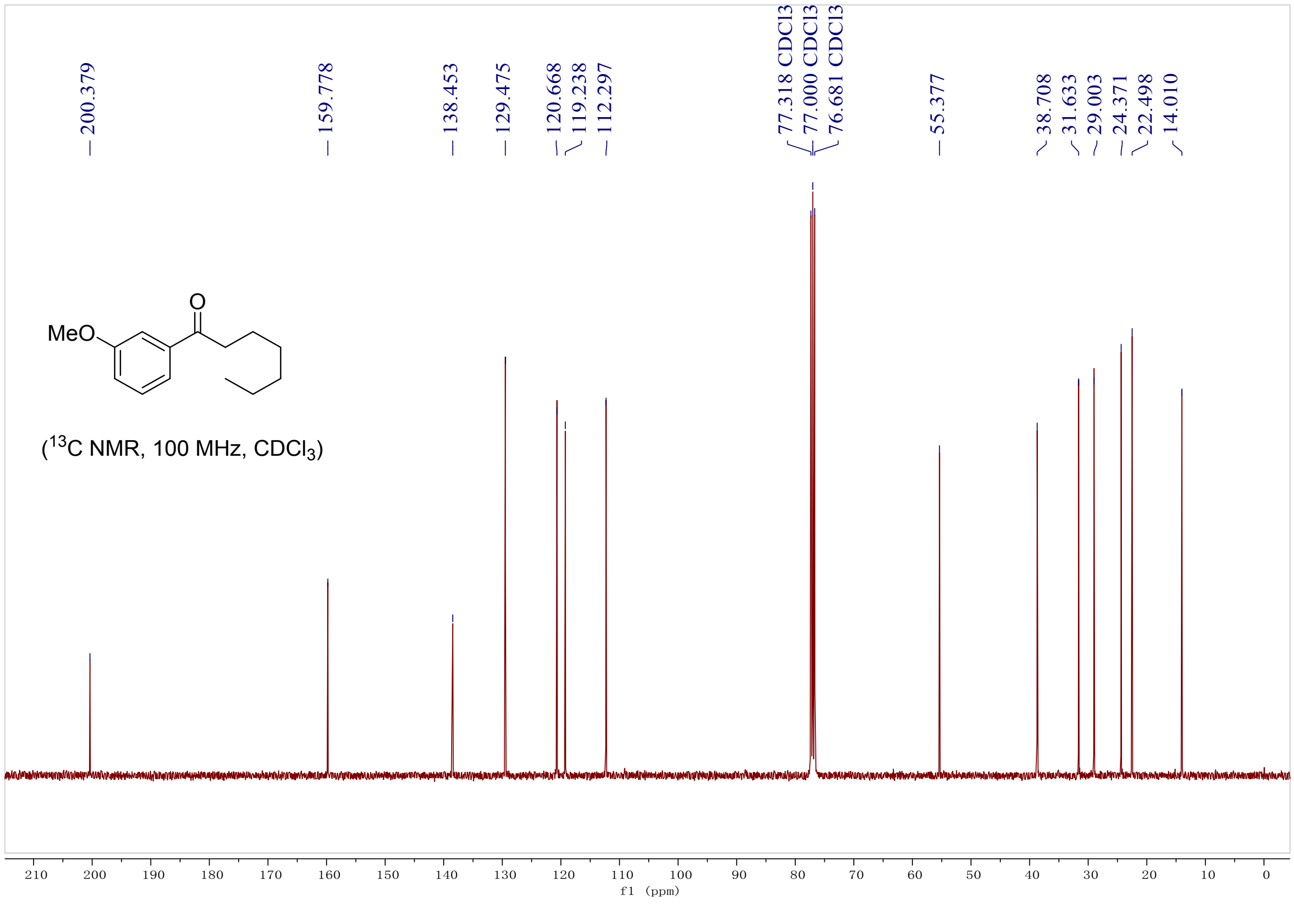
200.379
159.778
138.453
129.475
120.668
119.238
112.297
77.318 CDCl3
77.000 CDCl3
76.681 CDCl3
55.377
38.708
31.633
29.003
24.371
22.498
14.010
MeO
O
(13C NMR, 100 MHz, CDCl3)
f1 (ppm)

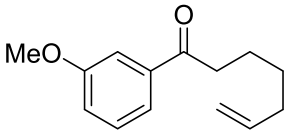

1-(3-methoxyphenyl)hept-6-en-1-one (9): colorless oil, 62 mg, 95%, Eluent: PE/EA = 20/1.

^{1}H NMR (400 MHz, $CDCl_3$, TMS): δ 7.53 (dt, *J* = 7.8, 1.2 Hz, 1H), 7.49 (dd, *J* = 2.8, 1.6 Hz, 1H), 7.36 (t, *J* = 8.0 Hz, 1H), 7.10 (dd, *J* = 8.2, 2.8 Hz, 1H), 5.82 (ddt, *J* = 16.8, 10.2, 6.8 Hz, 1H), 5.07-4.93 (m, 2H), 3.85 (s, 3H), 2.96 (t, *J* = 7.4 Hz, 2H), 2.16-2.05 (m, 2H), 1.80-1.70 (m, 2H), 1.53-1.43 (m, 2H). **^{13}C NMR** (100 MHz, $CDCl_3$, TMS): δ 23.8, 28.5, 33.5, 38.5, 55.4, 112.3, 114.6, 119.3, 120.6, 129.5, 138.4, 138.5, 159.8, 200.1. **IR** (EtOH): ν 3056, 2935, 2892, 2845, 1679, 1640, 1584, 1485, 1324, 1256, 1042, 1002, 998, 867, 780, 667 cm^{-1}. **HRMS** (EI) m/z: $[M]^+$ Calcd. for $C_{14}H_{18}O_2$ 218.1301; found 218.1299.

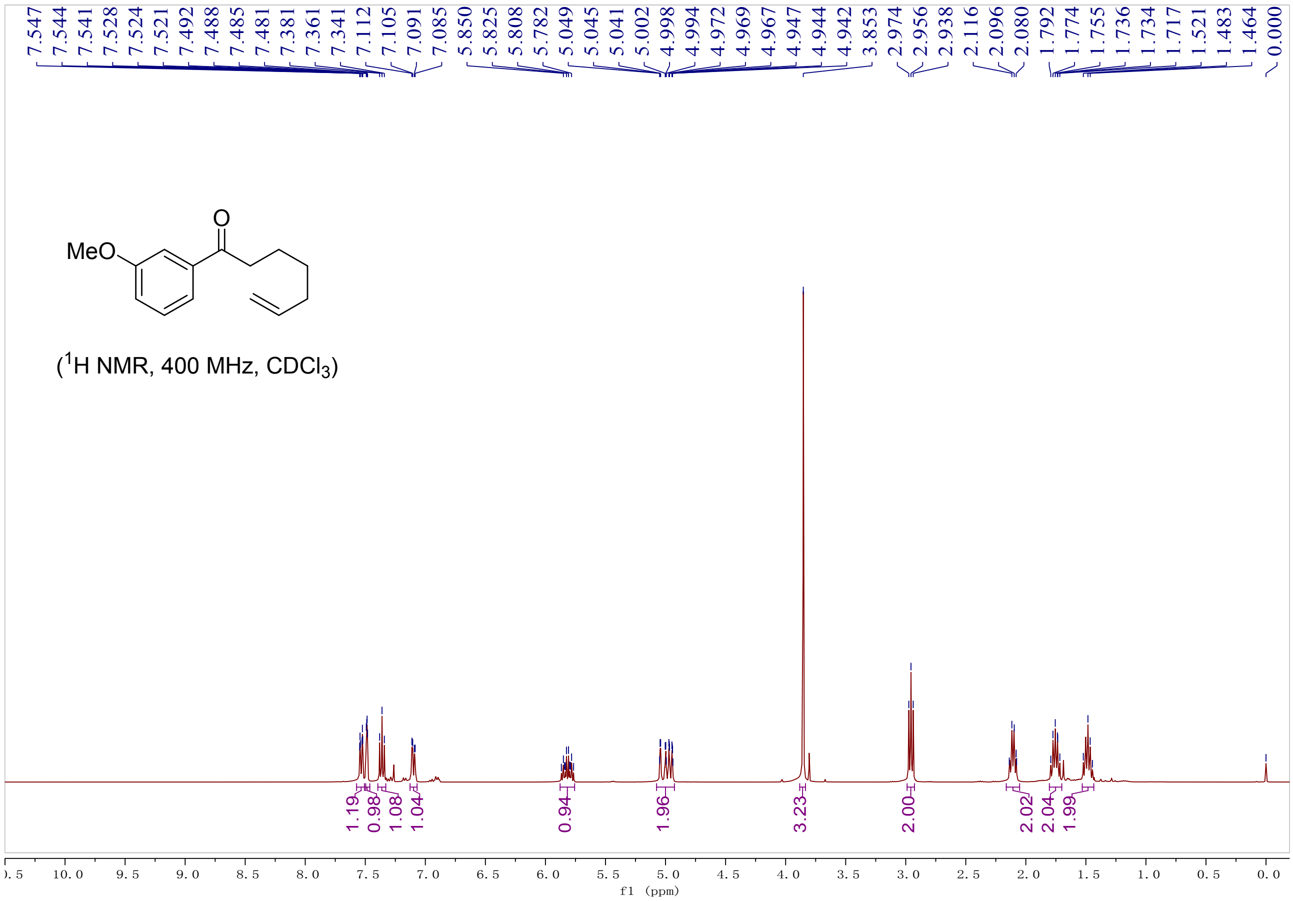
7.547 7.544 7.541 7.528 7.524 7.521 7.492 7.488 7.485 7.481 7.381 7.361 7.341 7.112 7.105 7.091 7.085 5.850 5.825 5.808 5.782 5.049 5.045 5.041 5.002 4.998 4.994 4.972 4.969 4.967 4.947 4.944 4.942 3.853 2.974 2.956 2.938 2.116 2.096 2.080 1.792 1.774 1.755 1.736 1.734 1.717 1.521 1.483 1.464 0.000
MeO
O
(^{1}H NMR, 400 MHz, CDCl$_3$)
1.19 0.98 1.08 1.04 0.94 1.96 3.23 2.00 2.02 2.04 1.99
f1 (ppm)

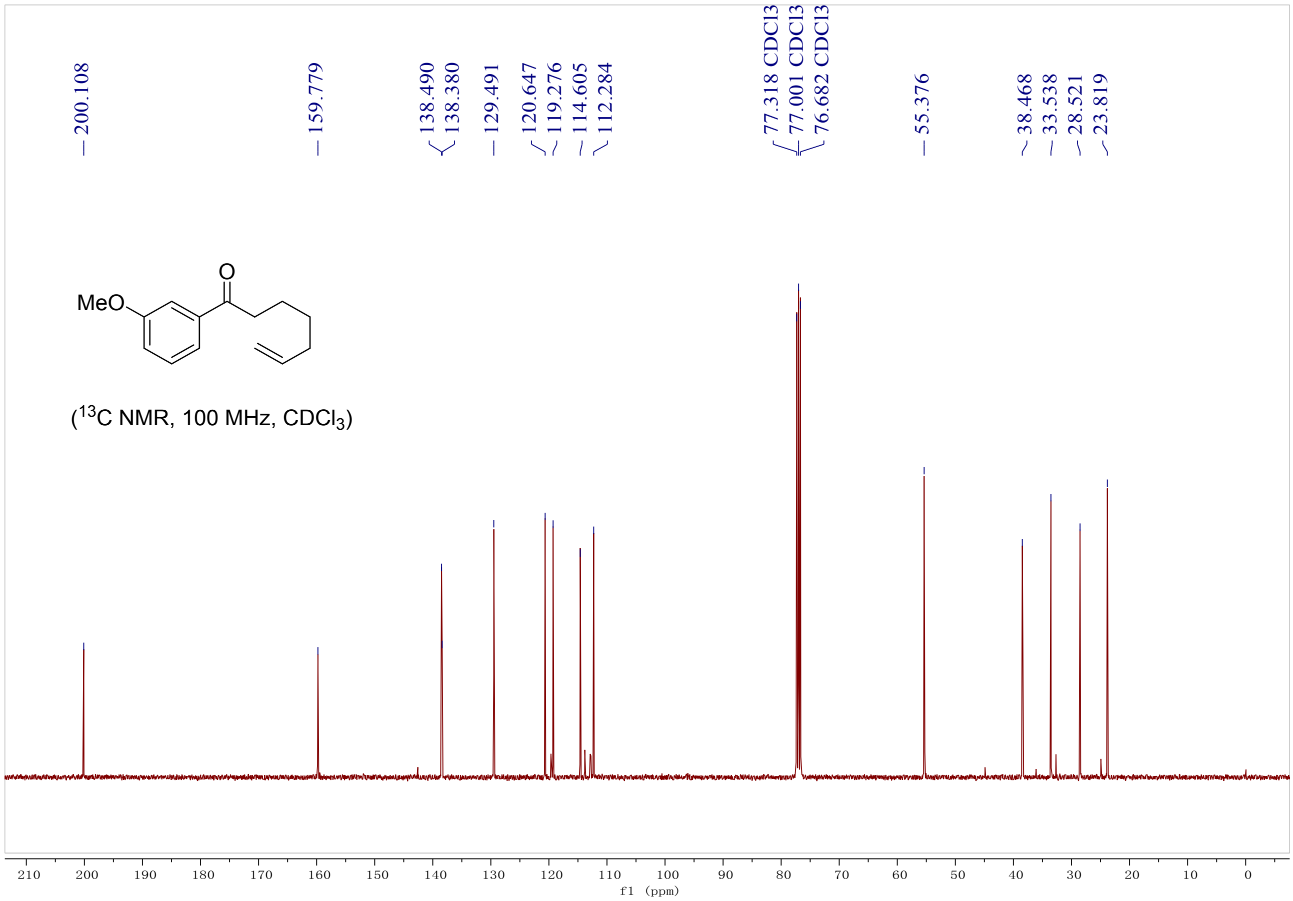
200.108 159.779 138.490 138.380 129.491 120.647 119.276 114.605 112.284 77.318 CDCl3 77.001 CDCl3 76.682 CDCl3 55.376 38.468 33.538 28.521 23.819
MeO
O
(^{13}C NMR, 100 MHz, CDCl$_3$)
f1 (ppm)

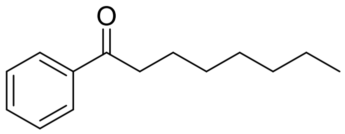

1-phenyloctan-1-one (S4): colorless oil, 1.5 g, 98%, Eluent: PE/EA = 20/1.

^{1}H NMR (400 MHz, $CDCl_3$, TMS): δ 7.98-7.94 (m, 2H), 7.57-7.51 (m, 1H), 7.49-7.40 (m, 2H), 2.95 (t, *J* = 7.4 Hz, 2H), 1.77-1.69 (m, 2H), 1.43-1.22 (m, 8H), 0.87 (t, *J* = 6.4 Hz, 3H). **^{13}C NMR** (100 MHz, $CDCl_3$, TMS): δ 14.0, 22.6, 24.3, 29.1, 29.3, 31.7, 38.5, 128.0, 128.5, 132.8, 137.0, 200.5. **IR** (EtOH): ν 2925, 2855, 1682, 1598, 1450, 1221, 1086, 1045, 880, 750, 689 cm^{-1}. **HRMS** (EI) m/z: $[M]^+$ Calcd. for $C_{14}H_{20}O$ 204.1509; found 204.1506.

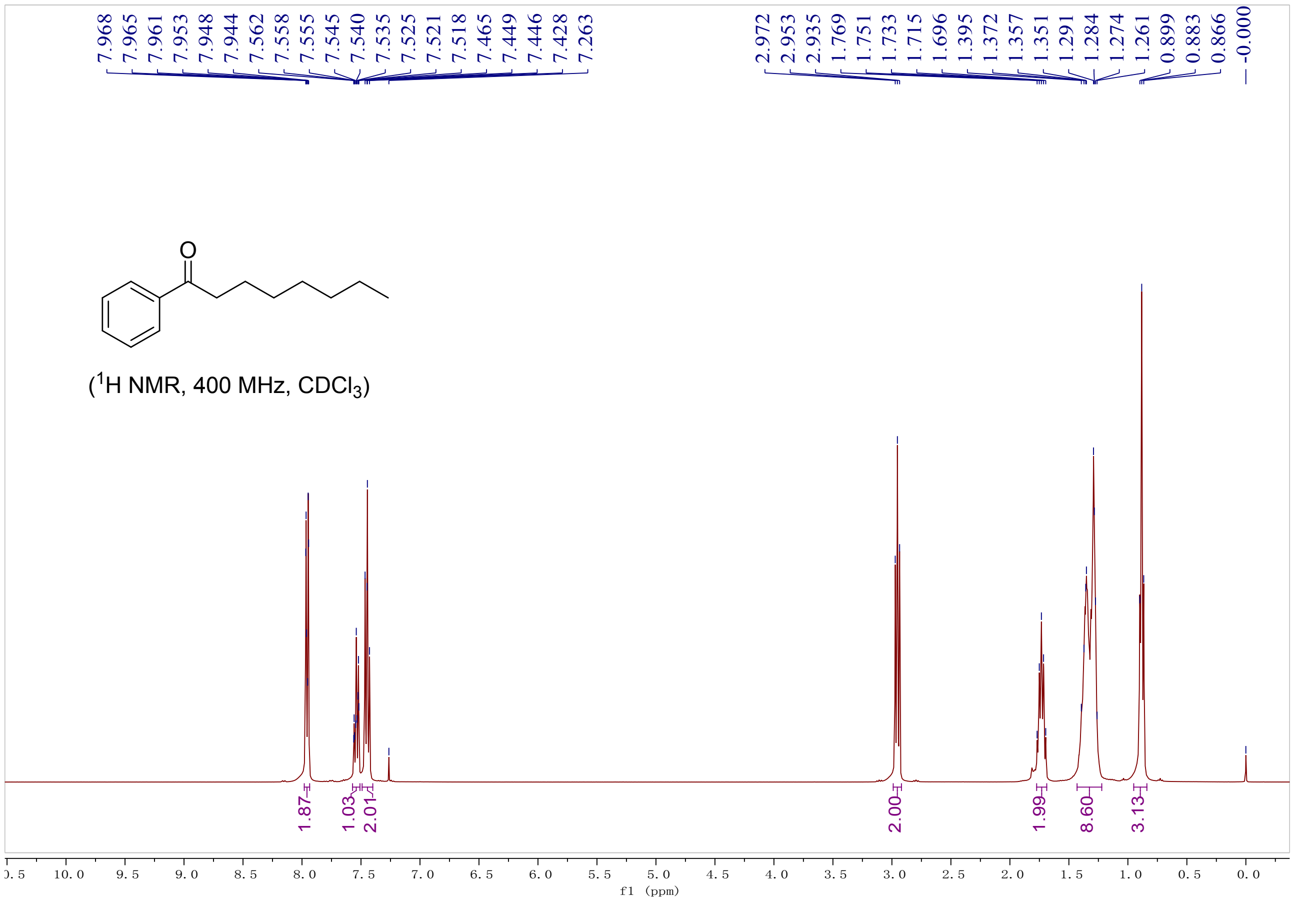
7.968 7.965 7.961 7.953 7.948 7.944 7.562 7.558 7.555 7.545 7.540 7.535 7.525 7.521 7.518 7.465 7.449 7.446 7.428 7.263
2.972 2.953 2.935 1.769 1.751 1.733 1.715 1.696 1.395 1.372 1.357 1.351 1.291 1.284 1.274 1.261 0.899 0.883 0.866 -0.000
O
(1H NMR, 400 MHz, CDCl3)
1.87 1.03 2.01 2.00 1.99 8.60 3.13
f1 (ppm)

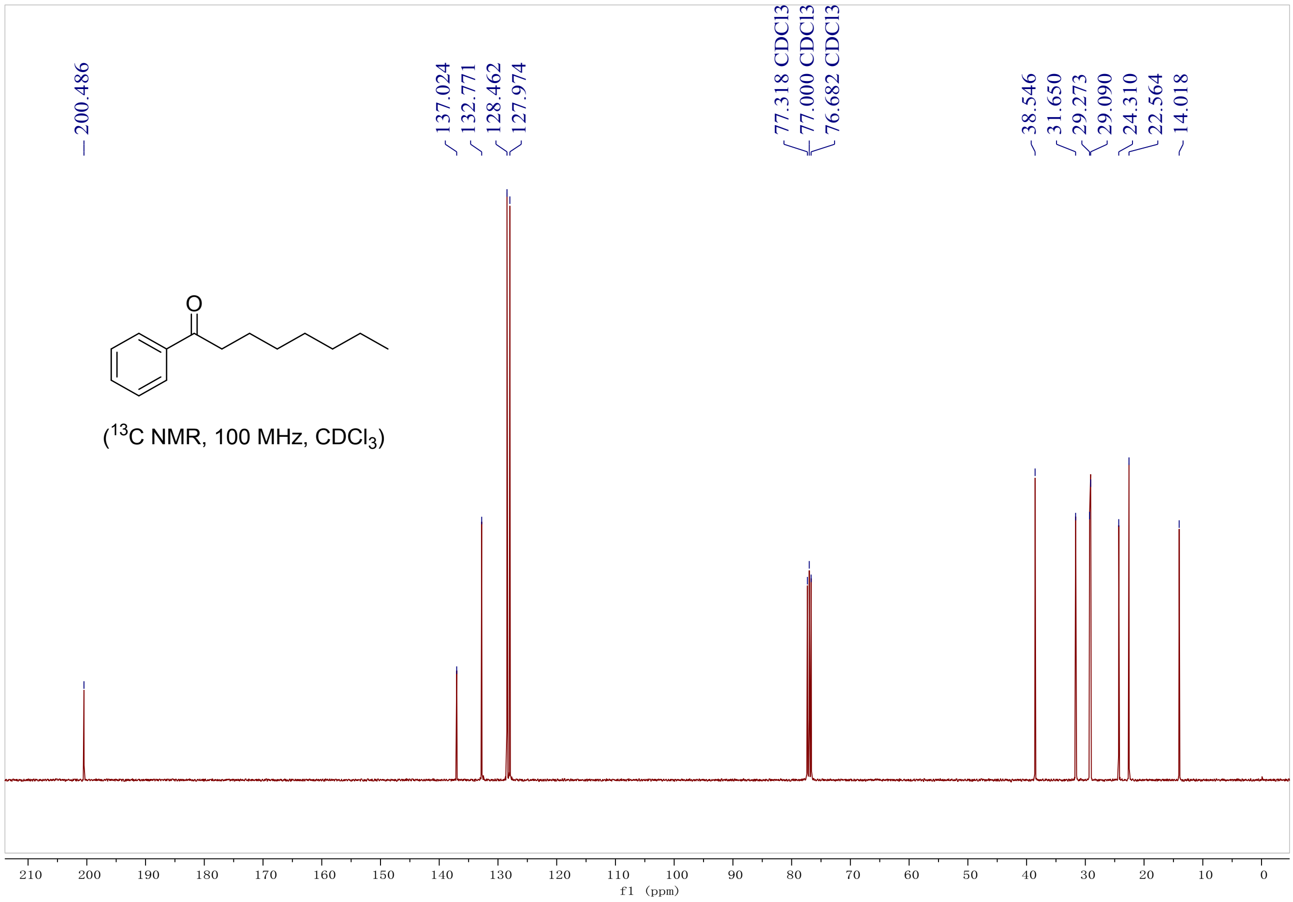
200.486
137.024 132.771 128.462 127.974
77.318 CDCl3 77.000 CDCl3 76.682 CDCl3
38.546 31.650 29.273 29.090 24.310 22.564 14.018
O
(13C NMR, 100 MHz, CDCl3)
f1 (ppm)

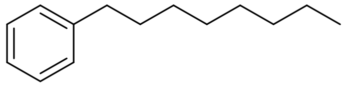

Octylbenzene (10): colorless oil, 910 mg, 65%, Eluent: PE/EA = 20/1.

^{1}H NMR (600 MHz, $CDCl_3$, TMS): δ 7.29-7.25 (m, 2H), 7.20-7.13 (m, 3H), 2.60 (t, *J* = 7.2 Hz, 2H), 1.65-1.57 (m, 2H), 1.37-1.21 (m, 10H), 0.88 (t, *J* = 7.0 Hz, 3H). **^{13}C NMR** (150 MHz, $CDCl_3$, TMS): δ 14.1, 22.7, 29.3, 29.4, 29.5, 31.5, 31.9, 36.0, 125.5, 128.2, 128.4, 143.0. **IR** (EtOH): ν 3027, 2922, 2908, 2853, 1495, 1457, 1376, 743, 696 cm^{-1}. **HRMS** (EI) m/z: $[M]^+$ Calcd. for $C_{14}H_{22}$ 190.1716; found 190.1714.

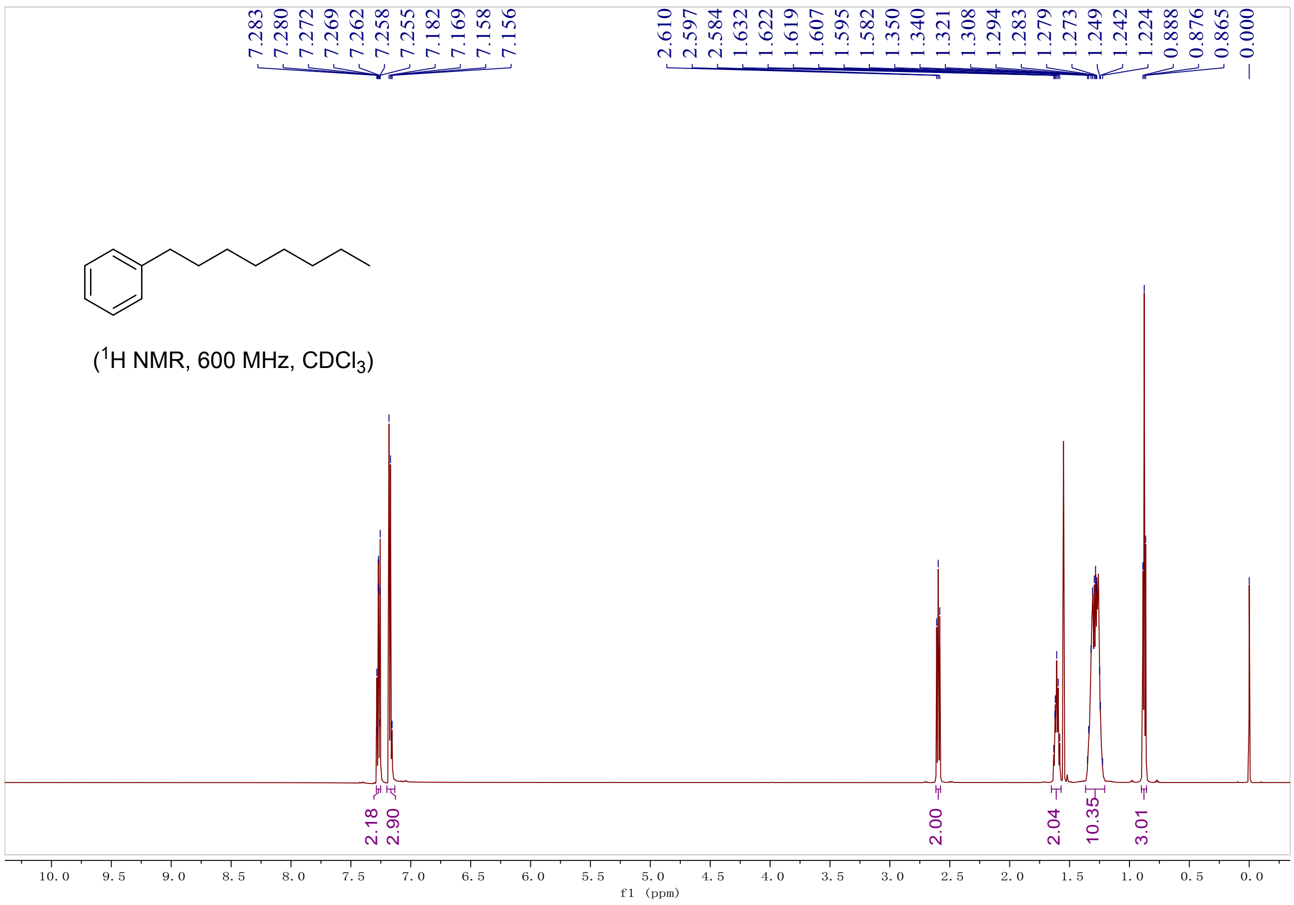
7.283
7.280
7.272
7.269
7.262
7.258
7.255
7.182
7.169
7.158
7.156
2.610
2.597
2.584
1.632
1.622
1.619
1.607
1.595
1.582
1.350
1.340
1.321
1.308
1.294
1.283
1.279
1.273
1.249
1.242
1.224
0.888
0.876
0.865
0.000
(¹H NMR, 600 MHz, CDCl3)
2.18
2.90
2.00
2.04
10.35
3.01
f1 (ppm)

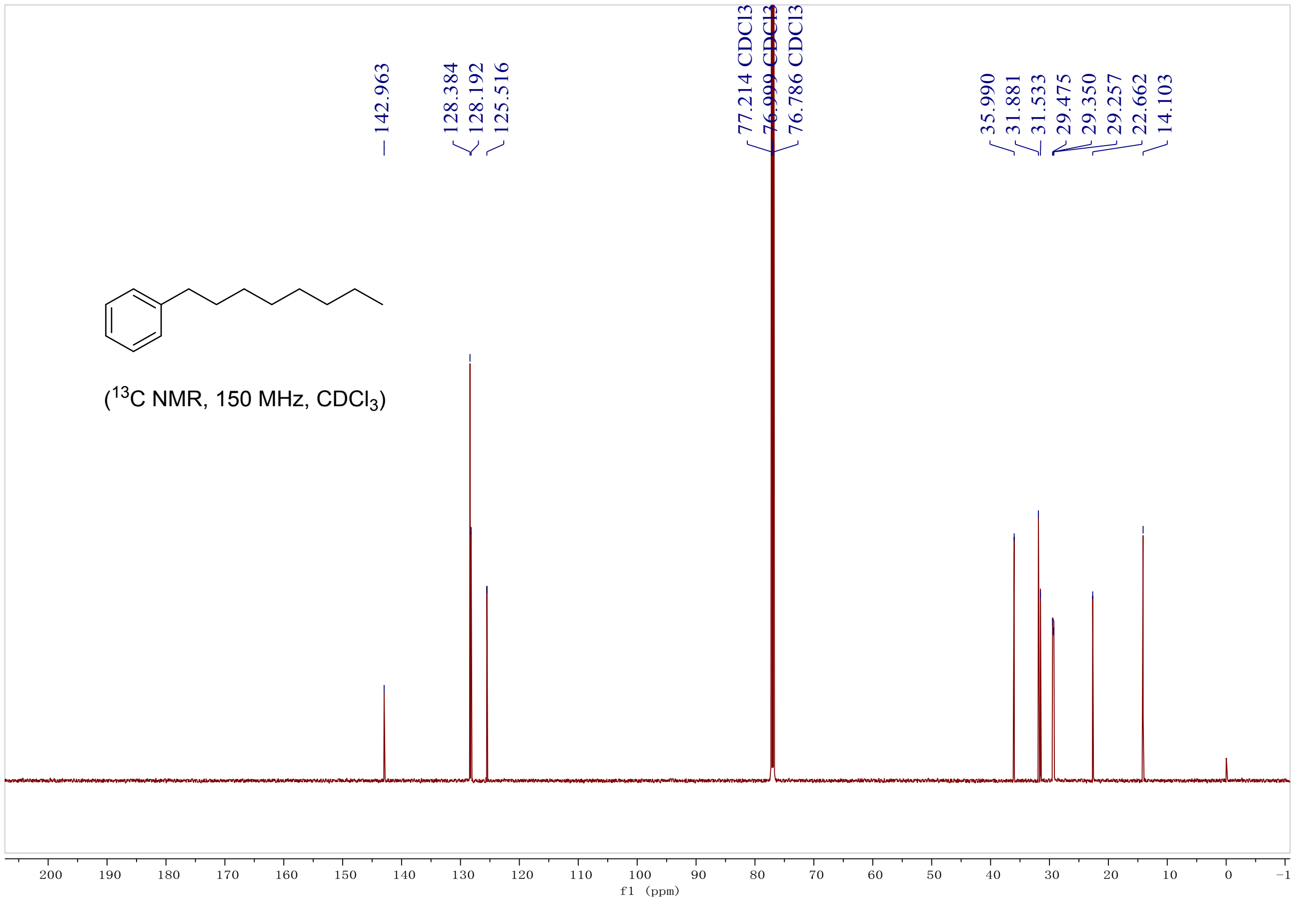
142.963
128.384
128.192
125.516
77.214 CDCl3
76.999 CDCl3
76.786 CDCl3
35.990
31.881
31.533
29.475
29.350
29.257
22.662
14.103
(¹³C NMR, 150 MHz, CDCl3)
f1 (ppm)

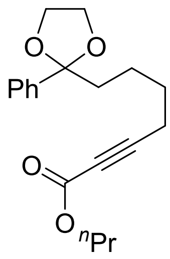

Propyl 7-(2-phenyl-1,3-dioxolan-2-yl)hept-2-ynoate (S5): colorless oil, 1.3 g, 91%, Eluent: PE/EA = 20/1.

^{1}H NMR (400 MHz, $CDCl_3$, TMS): δ 7.48-7.39 (m, 2H), 7.37-7.27 (m, 3H), 4.10 (t, *J* = 6.7 Hz, 2H), 4.05-3.97 (m, 2H), 3.81-3.72 (m, 2H), 2.28 (t, *J* = 7.2 Hz, 2H), 1.94-1.86 (m, 2H), 1.69 (h, *J* = 7.2 Hz, 2H), 1.60-1.53 (m, 3H), 1.50-1.41 (m, 2H), 0.96 (t, *J* = 7.4 Hz, 3H). **^{13}C NMR** (100 MHz, $CDCl_3$, TMS): δ 10.3, 18.7, 21.8, 23.0, 27.6, 39.8, 64.5, 67.3, 73.1, 89.2, 110.2, 125.6, 127.8, 128.1, 142.4, 154.0. **IR** (EtOH): ν 2952, 2923, 2234, 1707, 1463, 1390, 1244, 1075, 1042, 958, 766, 751, 703 cm^{-1}. **HRMS** (ESI) m/z: $[M+Na]^+$ Calcd. for $C_{19}H_{24}O_4Na$ 339.1567; found 339.1569.

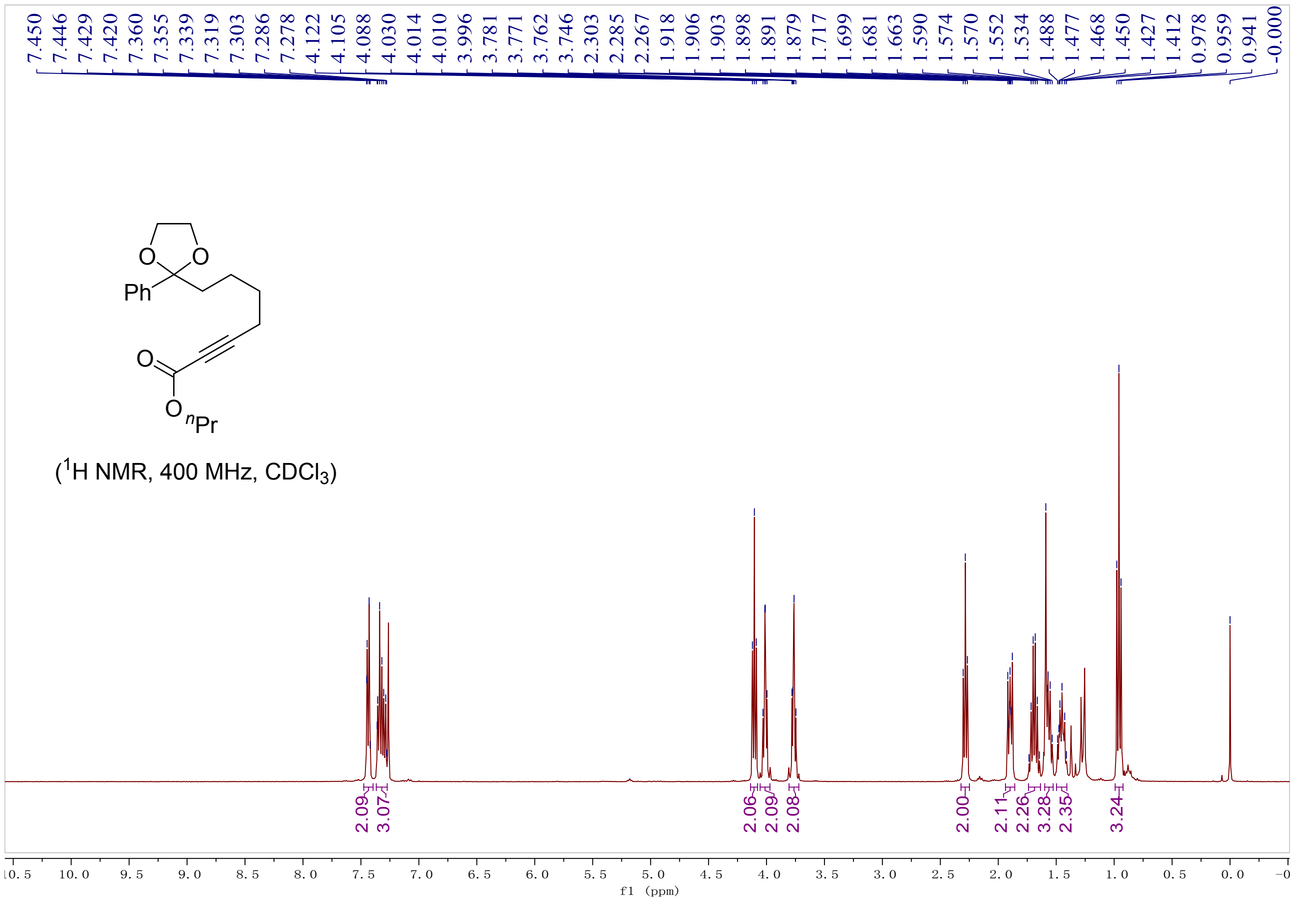

7.450 7.446 7.429 7.420 7.360 7.355 7.339 7.319 7.303 7.286 7.278 4.122 4.105 4.088 4.030 4.014 4.010 3.996 3.781 3.771 3.762 3.746 2.303 2.285 2.267 1.918 1.906 1.903 1.898 1.891 1.879 1.717 1.699 1.681 1.663 1.590 1.574 1.570 1.552 1.534 1.488 1.477 1.468 1.450 1.427 1.412 0.978 0.959 0.941 -0.000
O O
Ph
O
O nPr
(1H NMR, 400 MHz, CDCl3)
2.09 3.07 2.06 2.09 2.08 2.00 2.11 2.26 3.28 2.35 3.24
f1 (ppm)

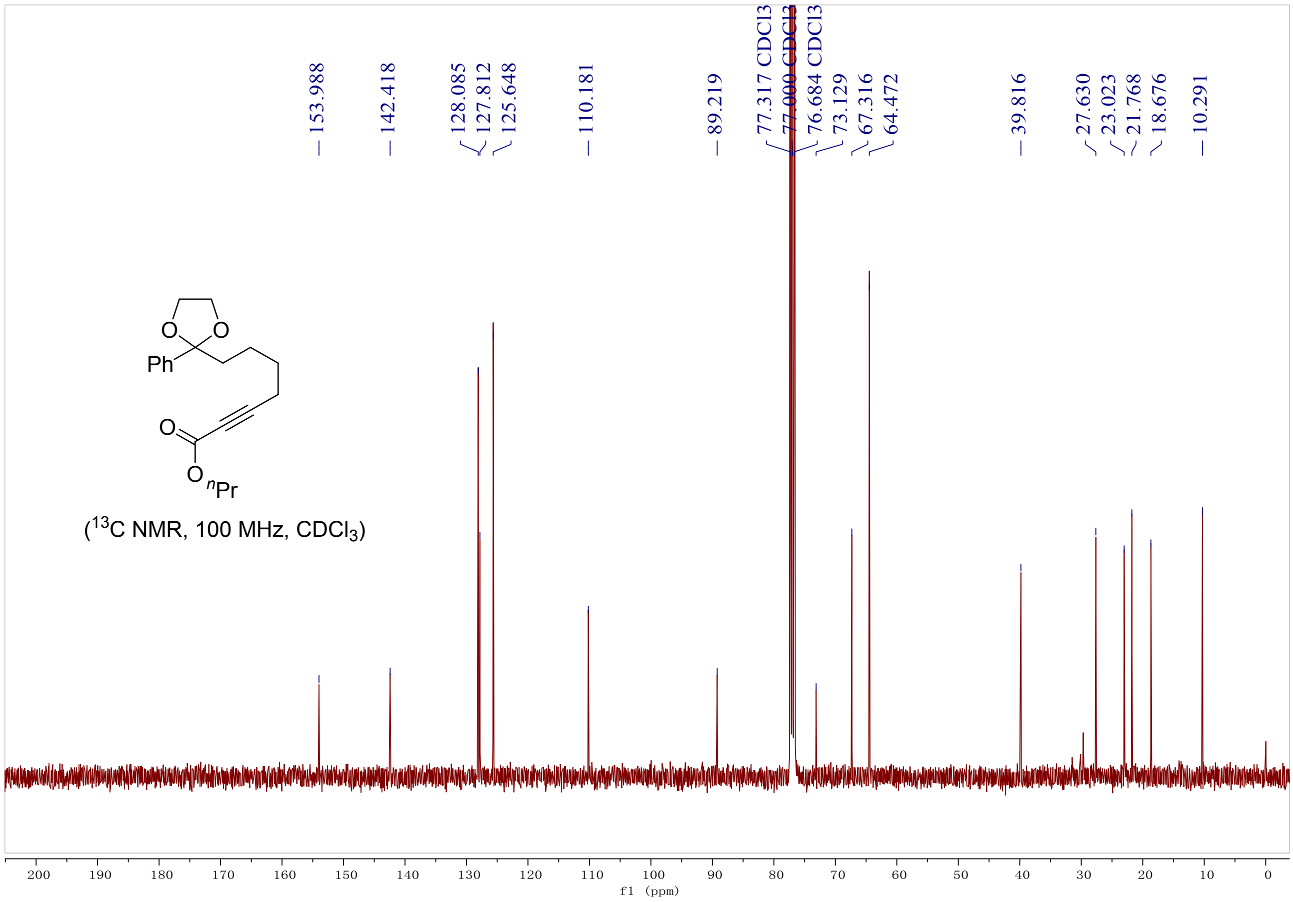

153.988 142.418 128.085 127.812 125.648 110.181 89.219 77.317 CDCl3 77.000 CDCl3 76.684 CDCl3 73.129 67.316 64.472 39.816 27.630 23.023 21.768 18.676 10.291
O O
Ph
O
O nPr
(13C NMR, 100 MHz, CDCl3)
f1 (ppm)

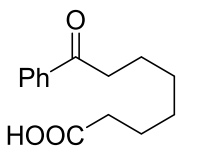

8-Oxo-8-phenyloctanoic acid (11): colorless solid, m.p. 147-148 ℃, 560 mg, 46%, Eluent: PE/EA = 1/1.

^{1}H NMR (400 MHz, $CDCl_3$, TMS): δ 7.99-7.93 (m, 2H), 7.59-7.52 (m, 1H), 7.46 (t, J = 7.6 Hz, 2H), 2.97 (t, J = 7.3 Hz, 2H), 2.36 (t, J = 7.5 Hz, 2H), 1.81-1.60 (m, 4H), 1.41 (p, J = 3.6 Hz, 4H). **^{13}C NMR** (150 MHz, $CDCl_3$, TMS): δ 24.1, 24.5, 28.92, 28.86, 33.8, 38.4, 128.1, 128.6, 132.9, 137.0, 179.0, 200.4. **IR** (EtOH): ν 3292, 2923, 2853, 1711, 1676, 1636, 1447, 1408, 1244, 1188, 1008, 846, 723, 689, 669 cm^{-1}. **HRMS** (ESI) m/z: $[M+Na]^+$ Calcd. for $C_{14}H_{18}O_3Na$ 257.1148; found 257.1156.

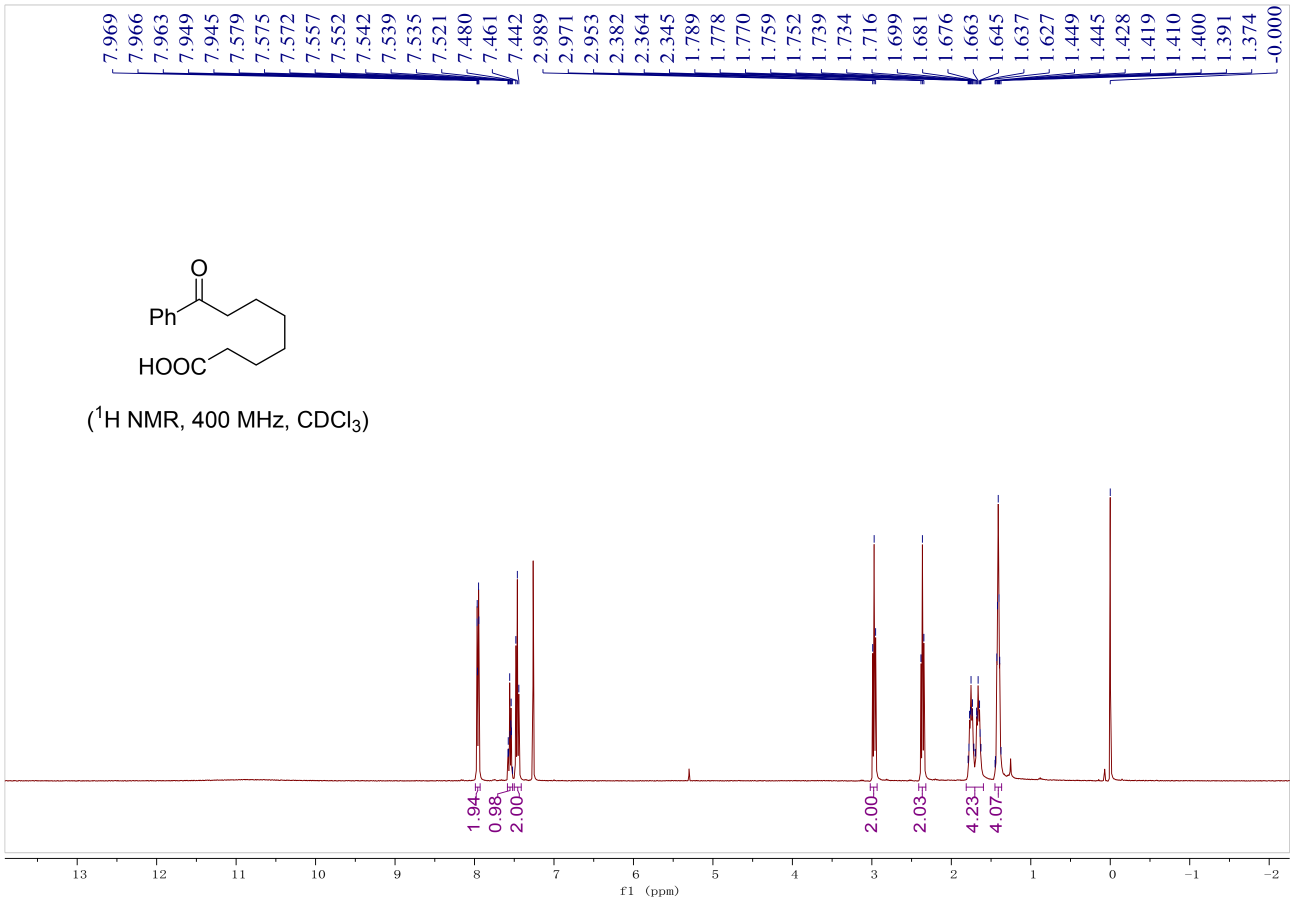
7.969
7.966
7.963
7.949
7.945
7.579
7.575
7.572
7.557
7.552
7.542
7.539
7.535
7.521
7.480
7.461
7.442
2.989
2.971
2.953
2.382
2.364
2.345
1.789
1.778
1.770
1.759
1.752
1.739
1.734
1.716
1.699
1.681
1.676
1.663
1.645
1.637
1.627
1.449
1.445
1.428
1.419
1.410
1.400
1.391
1.374
-0.000
O
Ph
HOOC
(1H NMR, 400 MHz, CDCl3)
1.94
0.98
2.00
2.00
2.03
4.23
4.07
f1 (ppm)

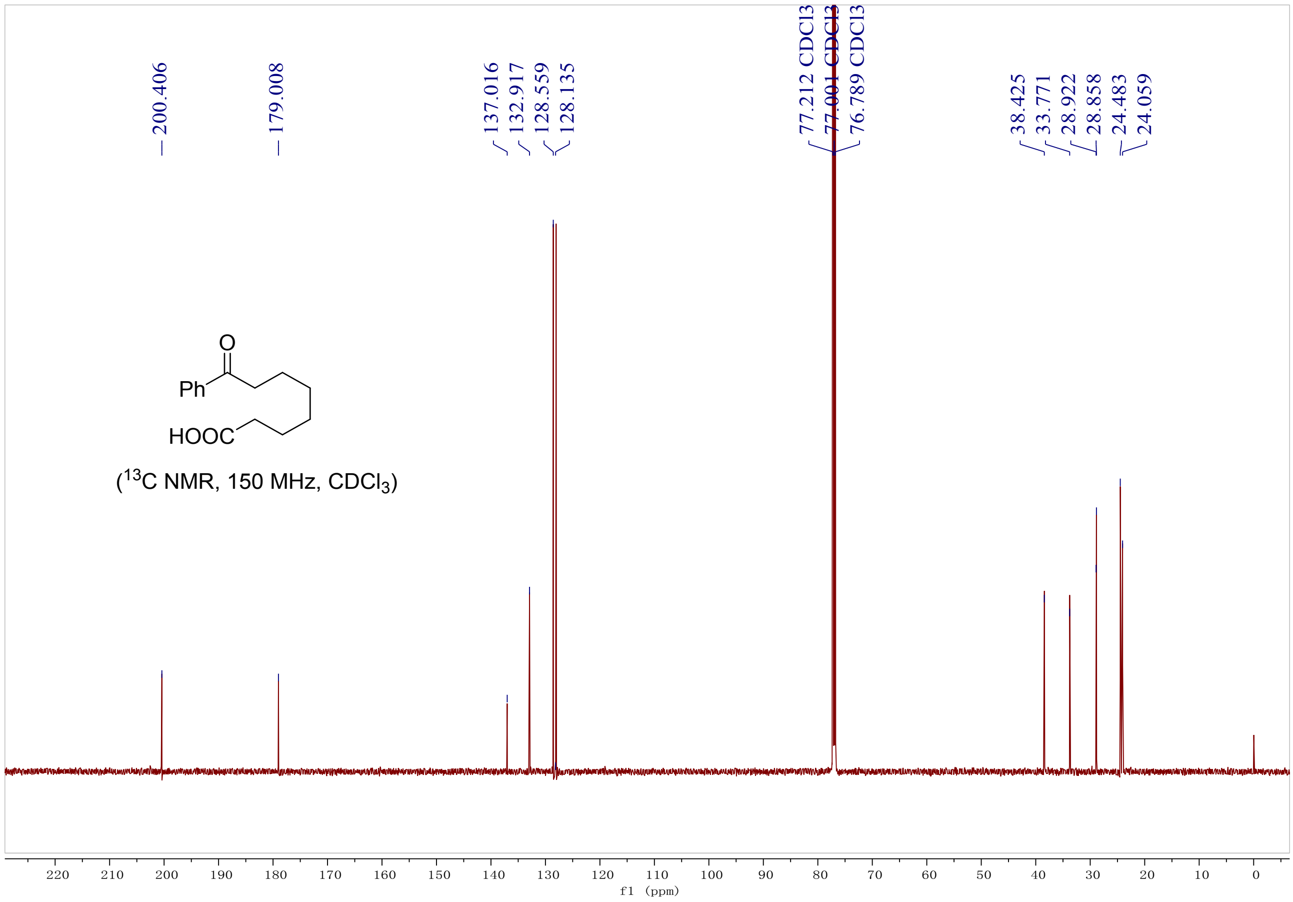
200.406
179.008
137.016
132.917
128.559
128.135
77.212 CDCl3
77.001 CDCl3
76.789 CDCl3
38.425
33.771
28.922
28.858
24.483
24.059
O
Ph
HOOC
(13C NMR, 150 MHz, CDCl3)
f1 (ppm)

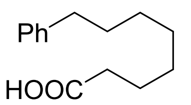

8-Phenyloctanoic acid (12) colorless oil, 492 mg, 43%, Eluent: PE/EA = 1/1.

^{1}H NMR (600 MHz, $CDCl_3$, TMS): δ 7.29-7.24 (m, 2H), 7.17 (dd, J = 6.6, 2.7 Hz, 3H), 2.60 (t, J = 7.7 Hz, 2H), 2.34 (t, J = 7.5 Hz, 2H), 1.67-1.57 (m, 4H), 1.37-1.31 (m, 6H). **^{13}C NMR** (150 MHz, $CDCl_3$, TMS): δ 24.6, 28.9, 29.06, 29.07, 31.4, 34.0, 35.9, 125.6, 128.2, 128.4, 142.8, 180.1. **IR** (EtOH): ν 3026, 2926, 2855, 1705, 1454, 1411, 1284, 1154, 1064, 935, 746, 697 cm^{-1}. **HRMS** (EI) m/z: $[M]^+$ Calcd. for $C_{14}H_{20}O_2$ 220.1458; found 220.1454.

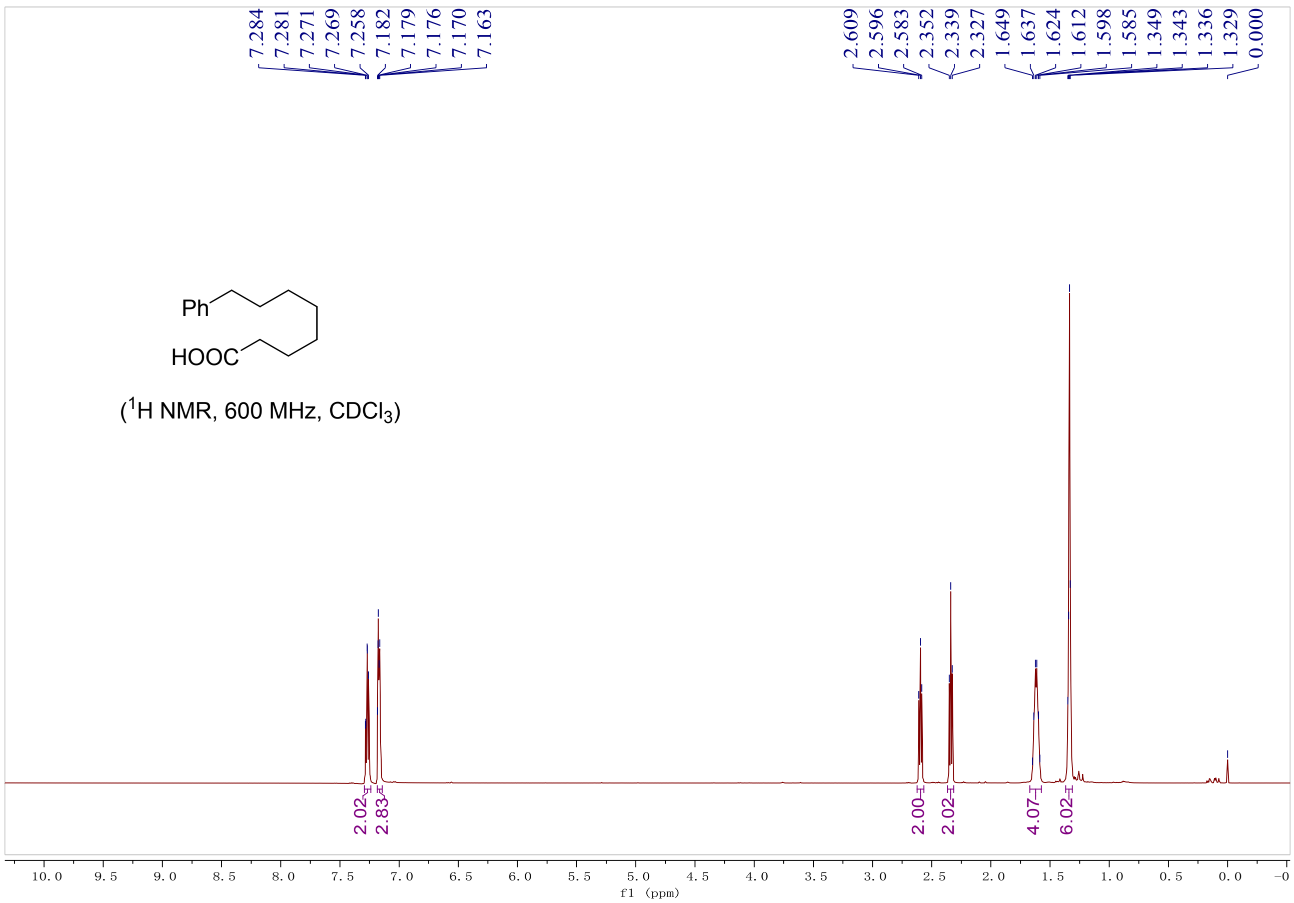
7.284
7.281
7.271
7.269
7.258
7.182
7.179
7.176
7.170
7.163
2.609
2.596
2.583
2.352
2.339
2.327
1.649
1.637
1.624
1.612
1.598
1.585
1.349
1.343
1.336
1.329
0.000
Ph
HOOC
(1H NMR, 600 MHz, CDCl3)
2.02
2.83
2.00
2.02
4.07
6.02
f1 (ppm)

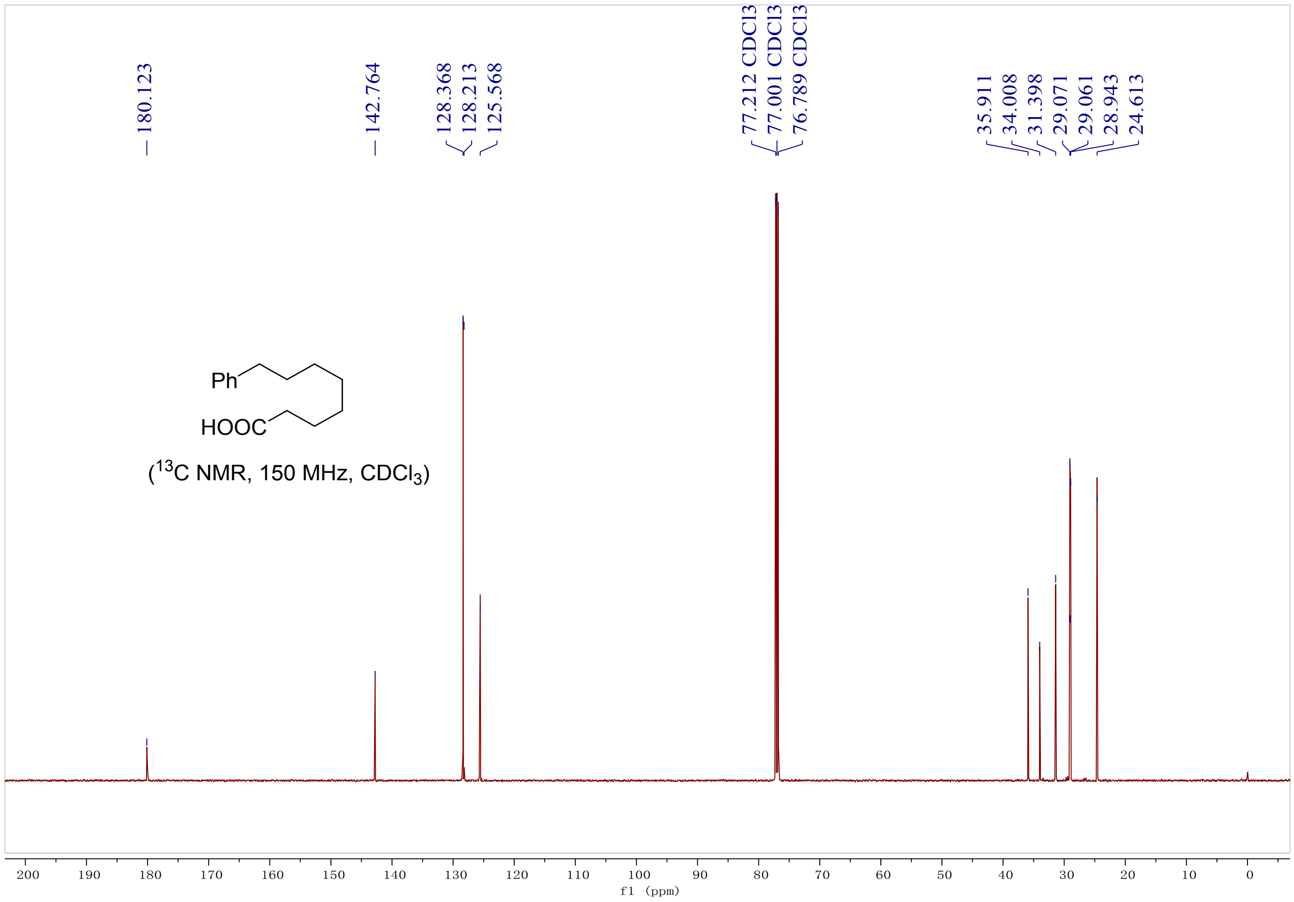
180.123
142.764
128.368
128.213
125.568
77.212 CDCl3
77.001 CDCl3
76.789 CDCl3
35.911
34.008
31.398
29.071
29.061
28.943
24.613
Ph
HOOC
(13C NMR, 150 MHz, CDCl3)
f1 (ppm)

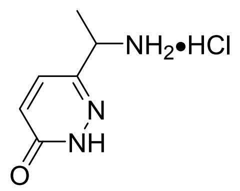

6-(1-aminoethyl)pyridazin-3(2*H*)-one (S6): grey solid, m.p. 267-269 °C, 1.8 g, Recrystallization. **^{1}H NMR** (600 MHz, DMSO-d_6, TMS): δ 13.22 (s, 1H), 8.56 (s, 3H), 7.65 (d, J = 9.8 Hz, 1H), 6.99 (d, J = 9.8 Hz, 1H), 4.47-4.34 (m, 1H), 1.48 (d, J = 6.9 Hz, 3H). **^{13}C NMR** (150 MHz, DMSO-d_6, TMS): δ 17.7, 47.5, 129.9, 131.4, 143.9, 159.7. **IR** (EtOH): ν 3390, 3100, 2833, 2510, 1643, 1585, 1524, 1433, 1259, 1124, 1078, 1008, 843, 724, 668 cm^{-1}. **HRMS** (ESI) m/z: [M]$^+$ Calcd. for $C_6H_{10}N_3O$ 140.0818; found 140.0814.

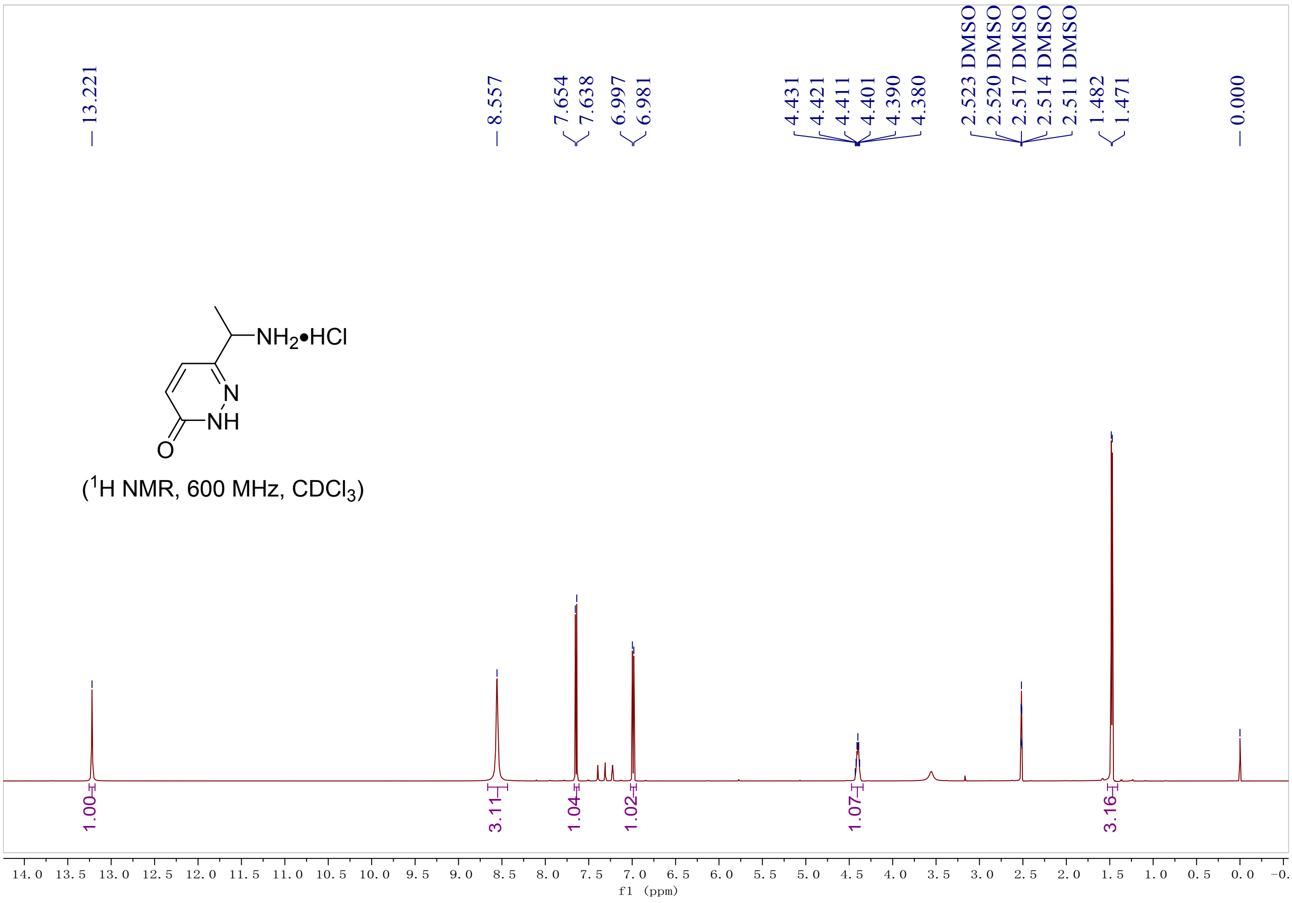
13.221
8.557
7.654
7.638
6.997
6.981
4.431
4.421
4.411
4.401
4.390
4.380
2.523 DMSO
2.520 DMSO
2.517 DMSO
2.514 DMSO
2.511 DMSO
1.482
1.471
0.000
NH2•HCl
N
NH
O
(1H NMR, 600 MHz, CDCl3)
1.00
3.11
1.04
1.02
1.07
3.16
f1 (ppm)

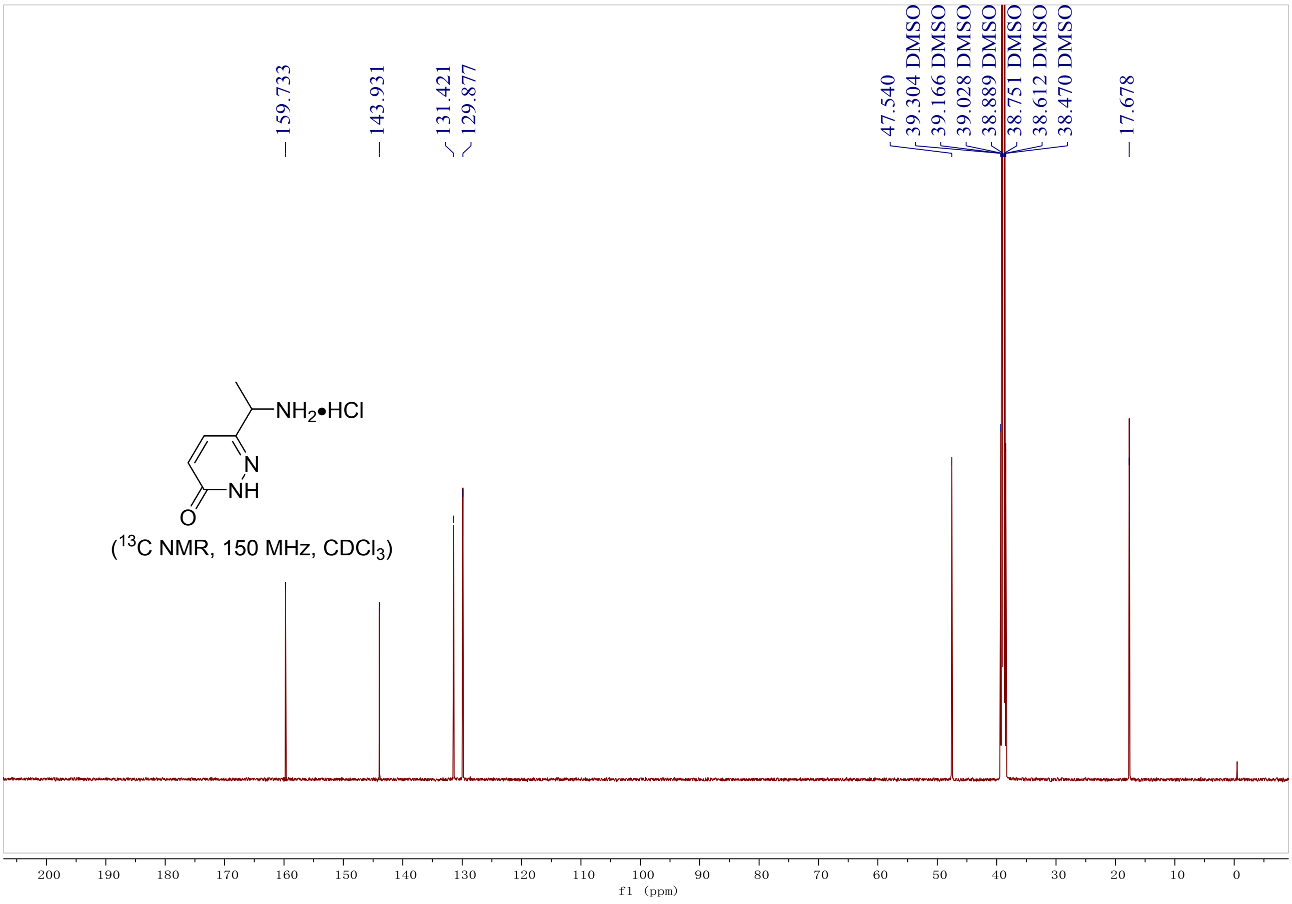
159.733
143.931
131.421
129.877
47.540
39.304 DMSO
39.166 DMSO
39.028 DMSO
38.889 DMSO
38.751 DMSO
38.612 DMSO
38.470 DMSO
17.678
NH2•HCl
N
NH
O
(13C NMR, 150 MHz, CDCl3)
f1 (ppm)

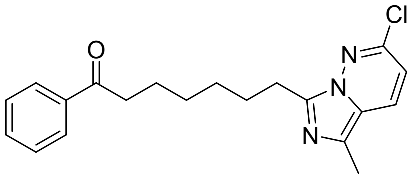

7-(2-chloro-5-methylimidazo[1,5-*b*]pyridazin-7-yl)-1-phenylheptan-1-one (13): yellow oil, 678 mg, 64%, Eluent: PE/EA = 1/1.

^{1}H NMR (600 MHz, $CDCl_3$, TMS): δ 7.97-7.93 (m, 2H), 7.61 (d, *J* = 9.3 Hz, 1H), 7.58-7.51 (m, 1H), 7.45 (t, *J* = 7.8 Hz, 2H), 6.35 (d, *J* = 9.4 Hz, 1H), 3.11-3.01 (m, 2H), 2.96 (t, *J* = 7.4 Hz, 2H), 2.47 (s, 3H), 1.88-1.82 (m, 2H), 1.79-1.71 (m, 3H), 1.45 (p, *J* = 3.6 Hz, 3H). **^{13}C NMR** (150 MHz, $CDCl_3$, TMS): δ 12.9, 24.2, 25.7, 26.9, 29.0, 29.1, 38.5, 110.5, 119.4, 128.0, 128.5, 129.3, 132.8, 137.0, 142.2, 147.9, 200.5. **IR** (EtOH): ν 2924, 2854, 1684, 1612, 1478, 1449, 1408, 1295, 1098, 968, 777, 750, 731, 697 cm^{-1}. **HRMS** (ESI) m/z: $[M+H]^+$ Calcd. for $C_{20}H_{23}N_3OCl$ 356.1524; found 356.1522.

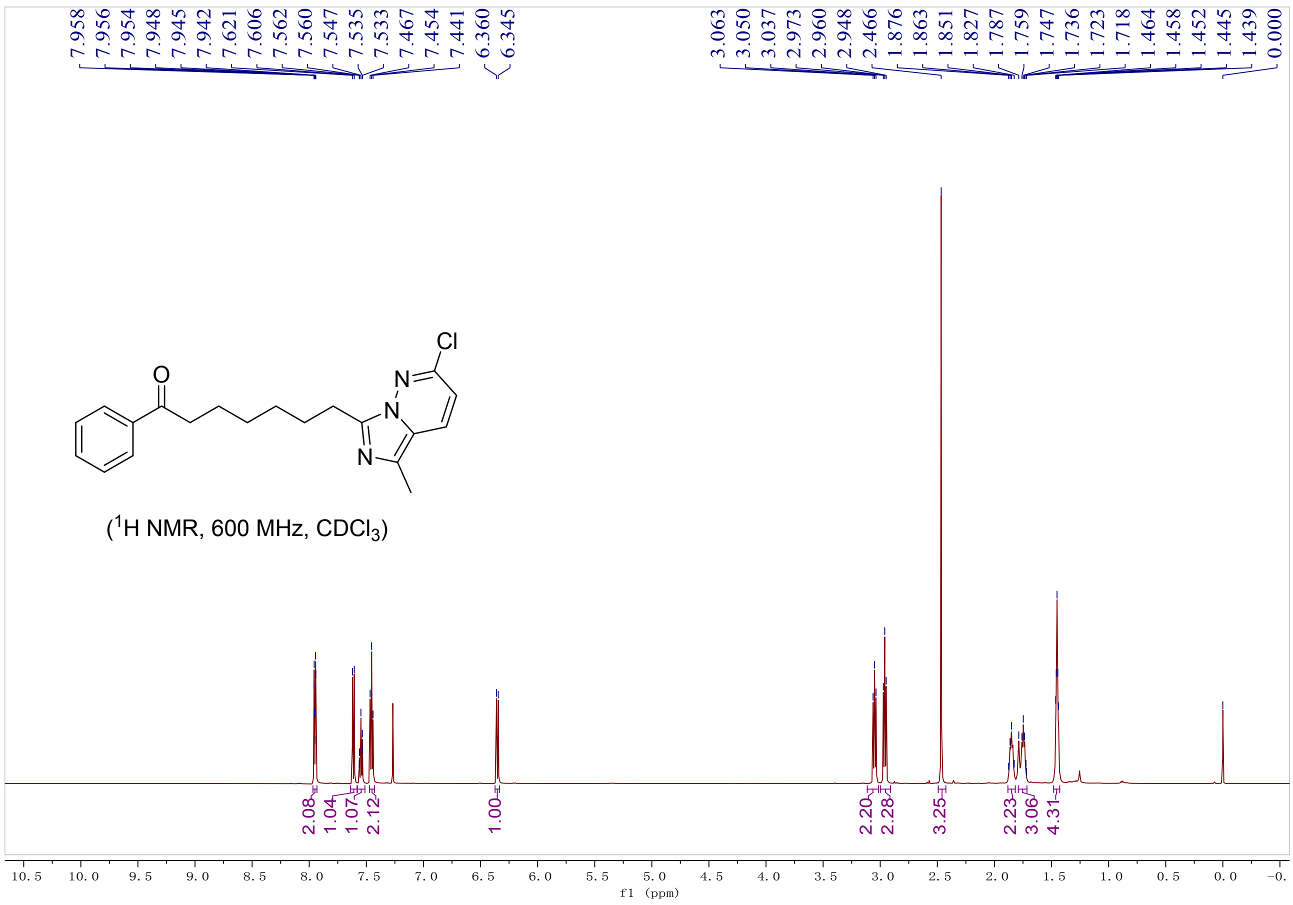
7.958
7.956
7.954
7.948
7.945
7.942
7.621
7.606
7.562
7.560
7.547
7.535
7.533
7.467
7.454
7.441
6.360
6.345
3.063
3.050
3.037
2.973
2.960
2.948
2.466
1.876
1.863
1.851
1.827
1.787
1.759
1.747
1.736
1.723
1.718
1.464
1.458
1.452
1.445
1.439
0.000
Cl
N
N
O
N
(1H NMR, 600 MHz, CDCl3)
2.08
1.04
1.07
2.12
1.00
2.20
2.28
3.25
2.23
3.06
4.31
f1 (ppm)

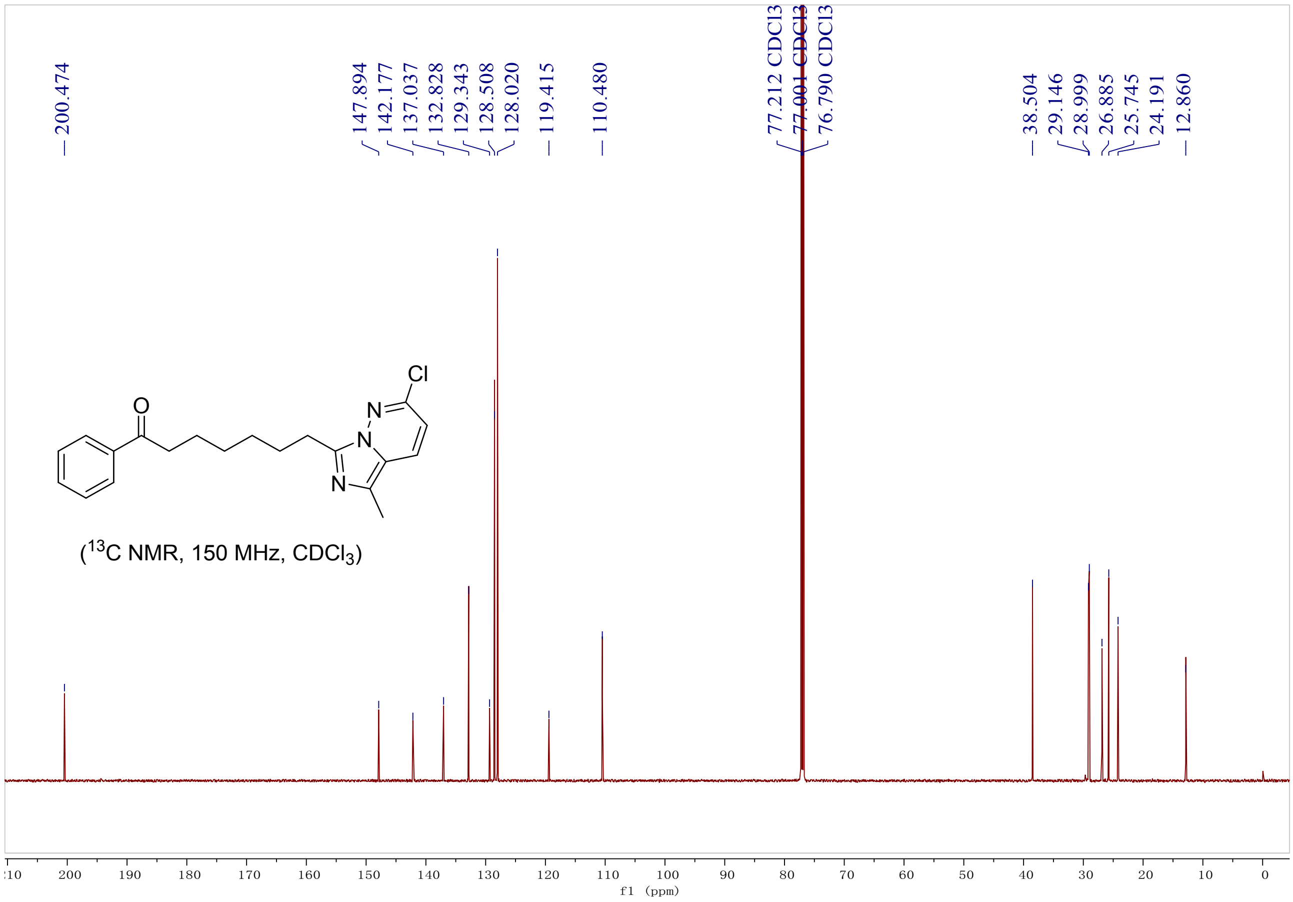
200.474
147.894
142.177
137.037
132.828
129.343
128.508
128.020
119.415
110.480
77.212 CDCl3
77.001 CDCl3
76.790 CDCl3
38.504
29.146
28.999
26.885
25.745
24.191
12.860
Cl
N
N
O
N
(13C NMR, 150 MHz, CDCl3)
f1 (ppm)

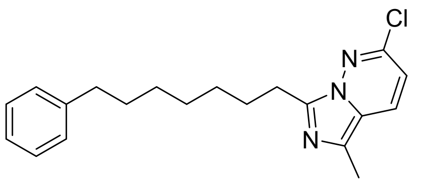

2-chloro-5-methyl-7-(7-phenylheptyl)imidazo[1,5-b]pyridazine (14): yellow oil, 636 mg, 62%, Eluent: PE/EA = 1/1.

^{1}H NMR (600 MHz, $CDCl_3$, TMS): δ 7.61 (d, J = 9.3 Hz, 1H), 7.29-7.23 (m, 2H), 7.17 (d, J = 6.2 Hz, 3H), 6.35 (d, J = 9.3 Hz, 1H), 3.05-3.01 (m, 2H), 2.62-2.57 (m, 2H), 2.47 (s, 3H), 1.84-1.79 (m, 2H), 1.63-1.57 (m, 4H), 1.40-1.32 (m, 4H). **^{13}C NMR** (150 MHz, $CDCl_3$, TMS): δ 12.9, 25.8, 27.0, 29.1, 29.2, 29.3, 31.5, 35.9, 110.5, 119.4, 125.5, 128.0, 128.2, 128.4, 129.4, 142.4, 142.9, 147.9. **IR** (EtOH): ν 2913, 2850, 1612, 1478, 1454, 1295, 1183, 1098, 967, 870, 776, 749, 698 cm^{-1}. **HRMS** (ESI) m/z: $[M+H]^+$ Calcd. for $C_{20}H_{25}N_3Cl$ 342.1732; found 342.1733.

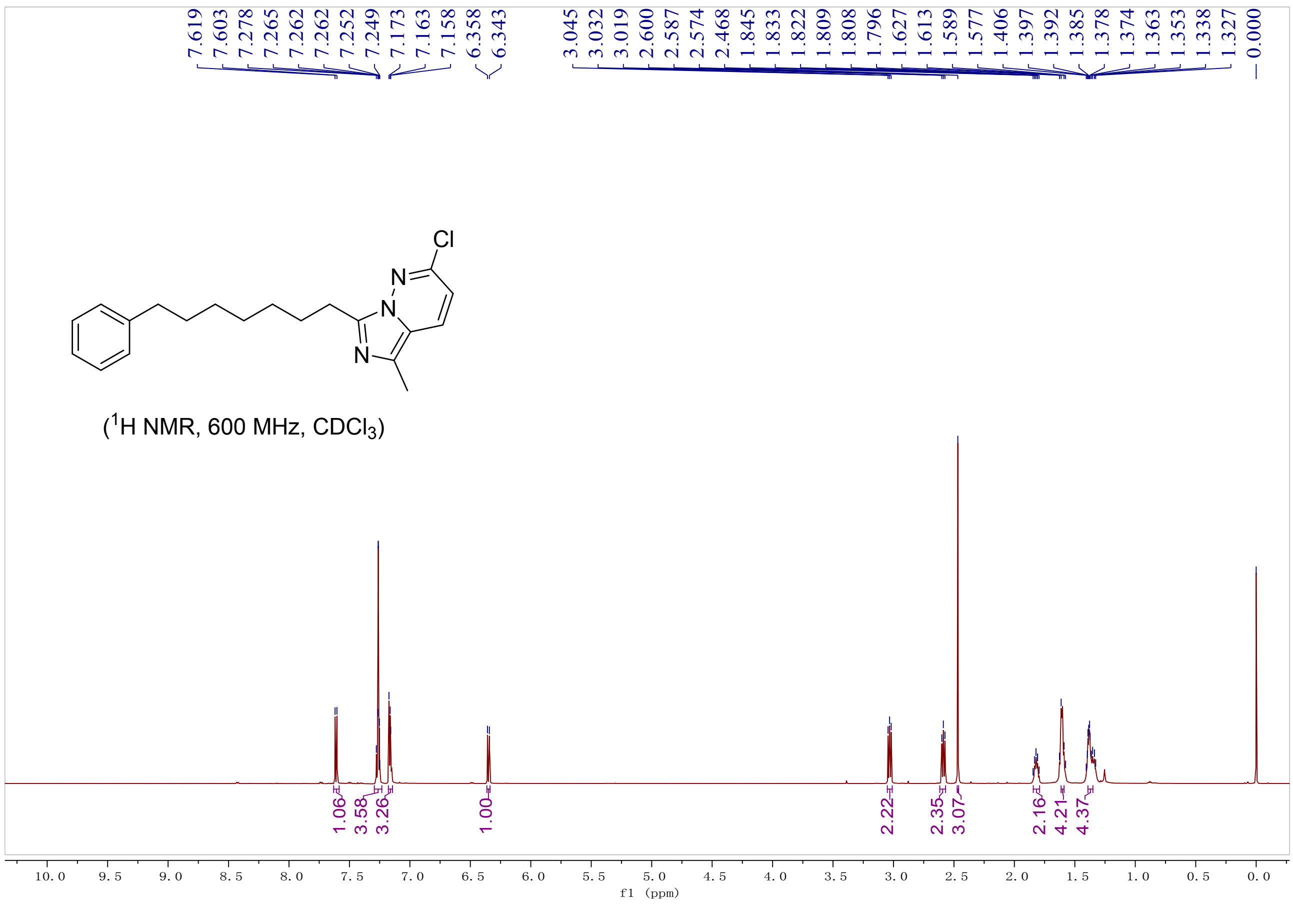
7.619
7.603
7.278
7.265
7.262
7.262
7.252
7.249
7.173
7.163
7.158
6.358
6.343
3.045
3.032
3.019
2.600
2.587
2.574
2.468
1.845
1.833
1.822
1.809
1.808
1.796
1.627
1.613
1.589
1.577
1.406
1.397
1.392
1.385
1.378
1.374
1.363
1.353
1.338
1.327
0.000
Cl
N
N
N
(^{1}H NMR, 600 MHz, $CDCl_3$)
1.06
3.58
3.26
1.00
2.22
2.35
3.07
2.16
4.21
4.37
f1 (ppm)

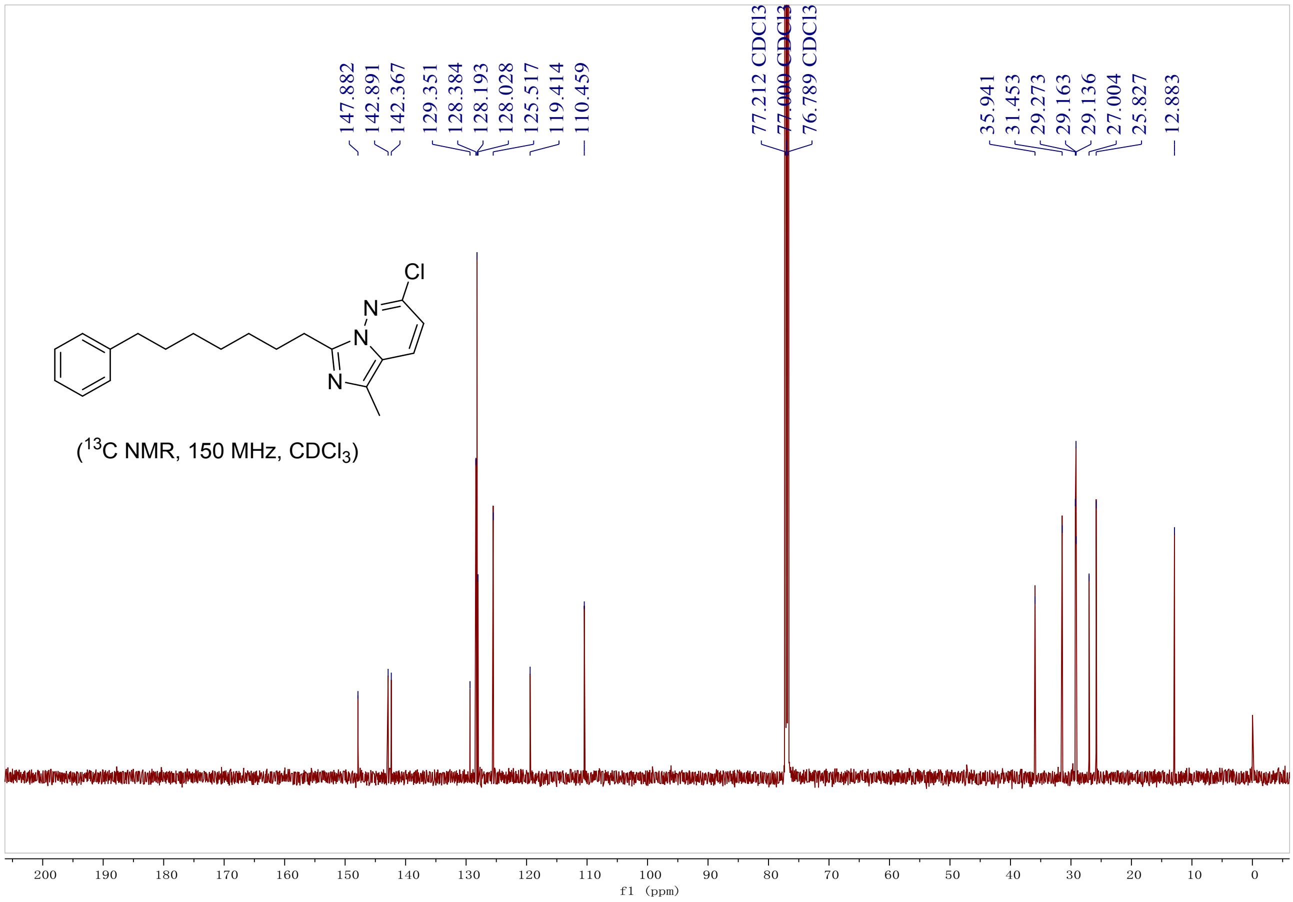
147.882
142.891
142.367
129.351
128.384
128.193
128.028
125.517
119.414
110.459
77.212 CDCl3
77.000 CDCl3
76.789 CDCl3
35.941
31.453
29.273
29.163
29.136
27.004
25.827
12.883
Cl
N
N
N
(^{13}C NMR, 150 MHz, $CDCl_3$)
f1 (ppm)

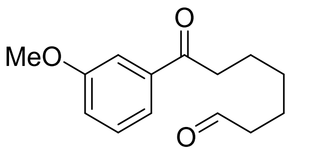

7-(3-methoxyphenyl)-7-oxoheptanal (15): colorless oil, 690 mg, 59%, Eluent: PE/EA = 20/1.

^{1}H NMR (400 MHz, $CDCl_3$, TMS): δ 9.77 (s, 1H), 7.57-7.45 (m, 2H), 7.37 (t, *J* = 8.0 Hz, 1H), 7.10 (dd, *J* = 8.2, 2.6 Hz, 1H), 3.85 (s, 3H), 2.97 (t, *J* = 7.2 Hz, 2H), 2.52-2.42 (m, 2H), 1.81-1.64 (m, 4H), 1.47-1.37 (m, 2H). **^{13}C NMR** (100 MHz, $CDCl_3$, TMS): δ 21.8, 23.9, 28.7, 38.2, 43.6, 55.3, 112.2, 119.3, 120.6, 129.5, 138.3, 159.7, 199.8, 202.5. **IR** (EtOH): ν 3098, 2967, 2940, 1748, 1682, 1587, 1467, 1267, 1189, 1060, 867, 789, 689, 668 cm^{-1}. **HRMS** (EI) m/z: $[M]^+$ Calcd. for $C_{14}H_{18}O_3$ 234.1250; found 234.1248.

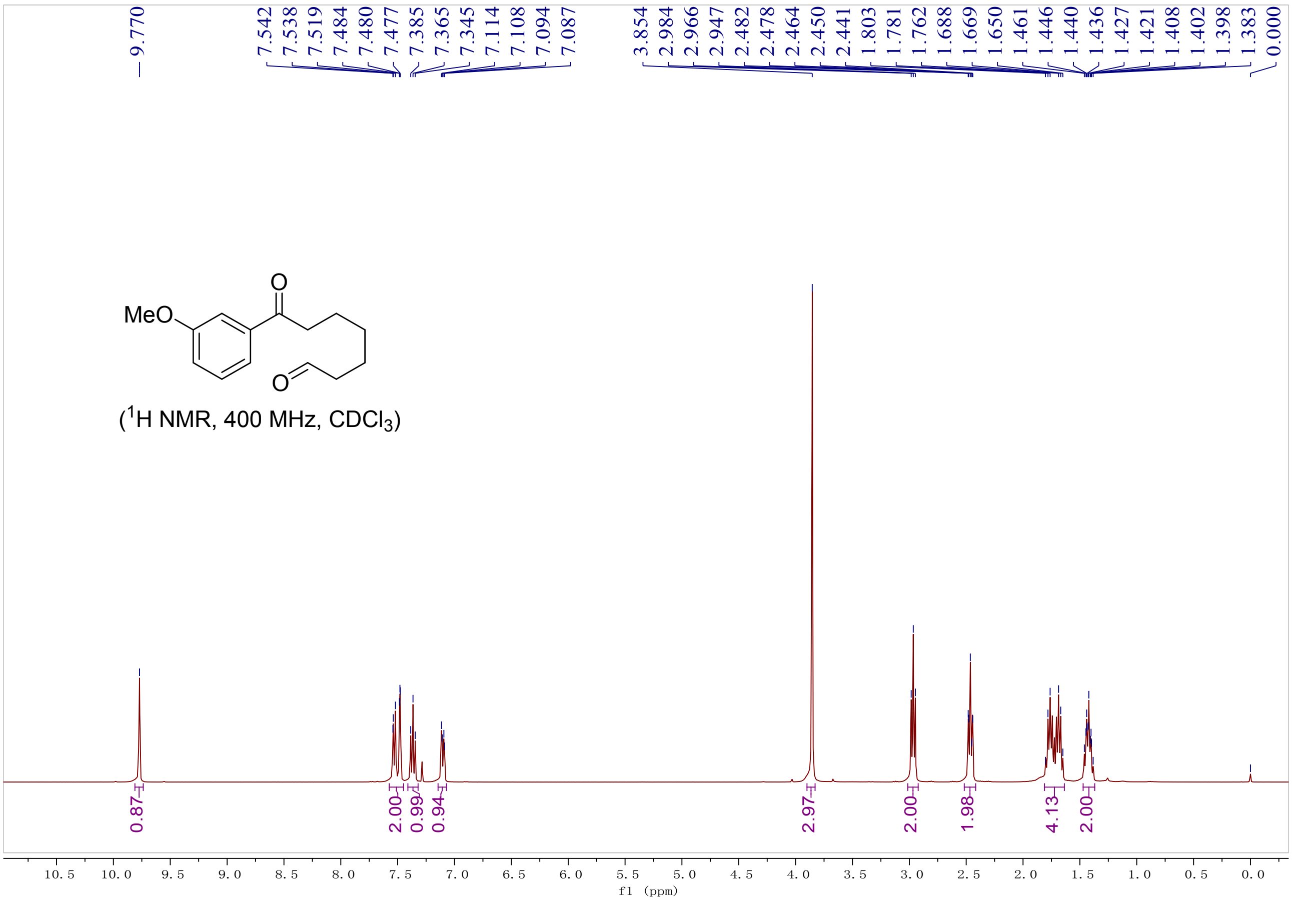
9.770
7.542
7.538
7.519
7.484
7.480
7.477
7.385
7.365
7.345
7.114
7.108
7.094
7.087
3.854
2.984
2.966
2.947
2.482
2.478
2.464
2.450
2.441
1.803
1.781
1.762
1.688
1.669
1.650
1.461
1.446
1.440
1.436
1.427
1.421
1.408
1.402
1.398
1.383
0.000
MeO
O
O
(1H NMR, 400 MHz, CDCl3)
0.87
2.00
0.99
0.94
2.97
2.00
1.98
4.13
2.00
10.5 10.0 9.5 9.0 8.5 8.0 7.5 7.0 6.5 6.0 5.5 5.0 4.5 4.0 3.5 3.0 2.5 2.0 1.5 1.0 0.5 0.0
f1 (ppm)

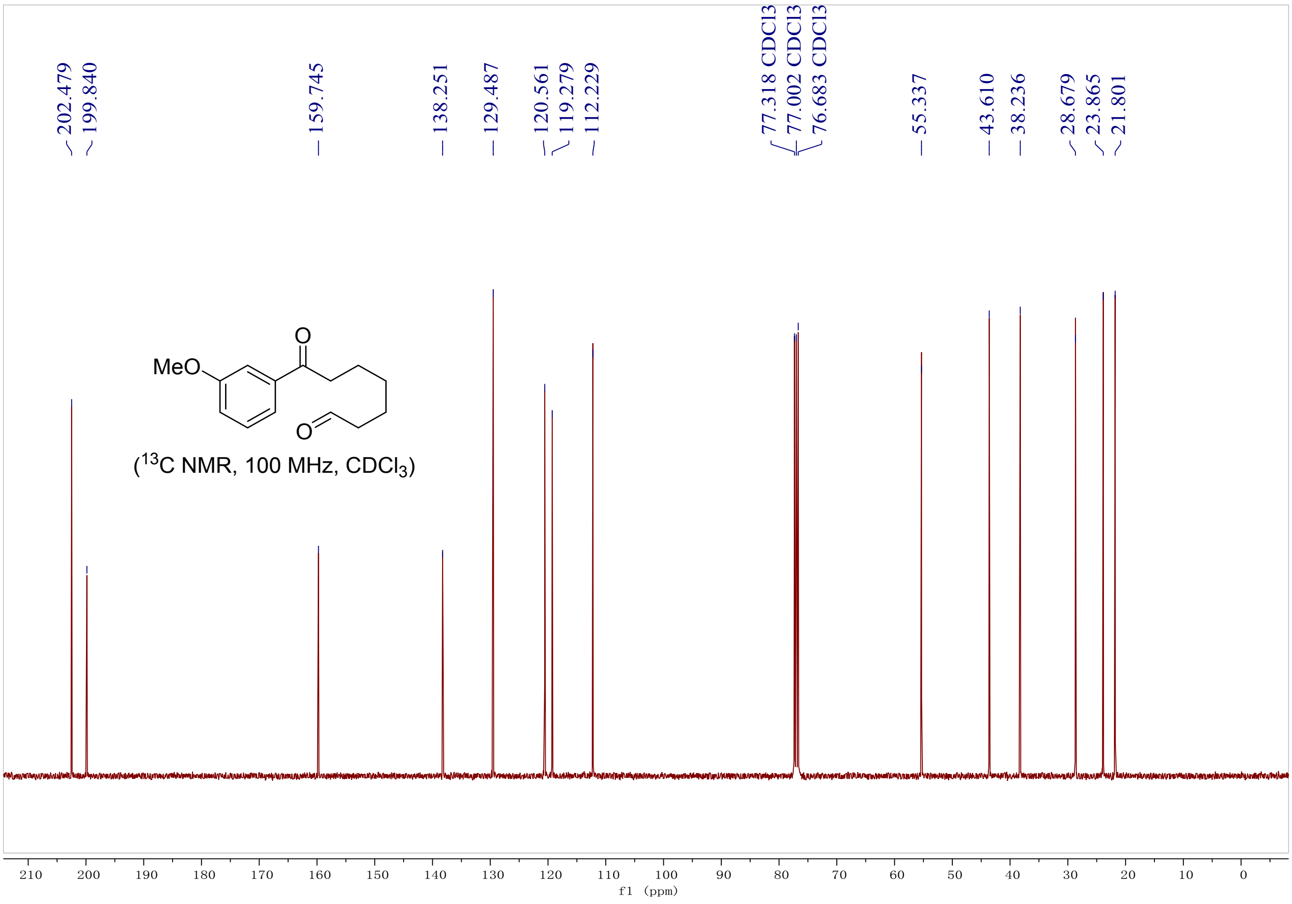
202.479
199.840
159.745
138.251
129.487
120.561
119.279
112.229
77.318 CDCl3
77.002 CDCl3
76.683 CDCl3
55.337
43.610
38.236
28.679
23.865
21.801
MeO
O
O
(13C NMR, 100 MHz, CDCl3)
210 200 190 180 170 160 150 140 130 120 110 100 90 80 70 60 50 40 30 20 10 0
f1 (ppm)

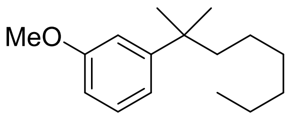

1-Methoxy-3-(2-methyloctan-2-yl)benzene (S7): colorless oil, 1.0 g, 90%, Eluent: PE/EA = 20/1. **^{1}H NMR** (400 MHz, $CDCl_3$, TMS): δ 7.22 (t, *J* = 8.0 Hz, 1H), 6.96-6.85 (m, 2H), 6.71 (dd, *J* = 8.2, 2.6 Hz, 1H), 3.80 (s, 3H), 1.60-1.54 (m, 2H), 1.27 (s, 6H), 1.24-1.15 (m, 6H), 1.05 (dt, *J* = 8.8, 4.8 Hz, 2H), 0.84 (t, *J* = 6.8 Hz, 3H). **^{13}C NMR** (150 MHz, $CDCl_3$, TMS): δ 14.1, 22.7, 24.6, 29.0, 30.0, 31.8, 37.7, 44.6, 55.1, 109.7, 112.6, 118.4, 128.8, 151.7, 159.3. **IR** (EtOH): ν 2988, 2975, 2842, 1507, 1454, 1380, 1277, 1087, 1045, 880, 759, 683, 665 cm^{-1}. **HRMS** (EI) m/z: $[M]^+$ Calcd. for $C_{16}H_{26}O$ 234.1978; found 234.1980.

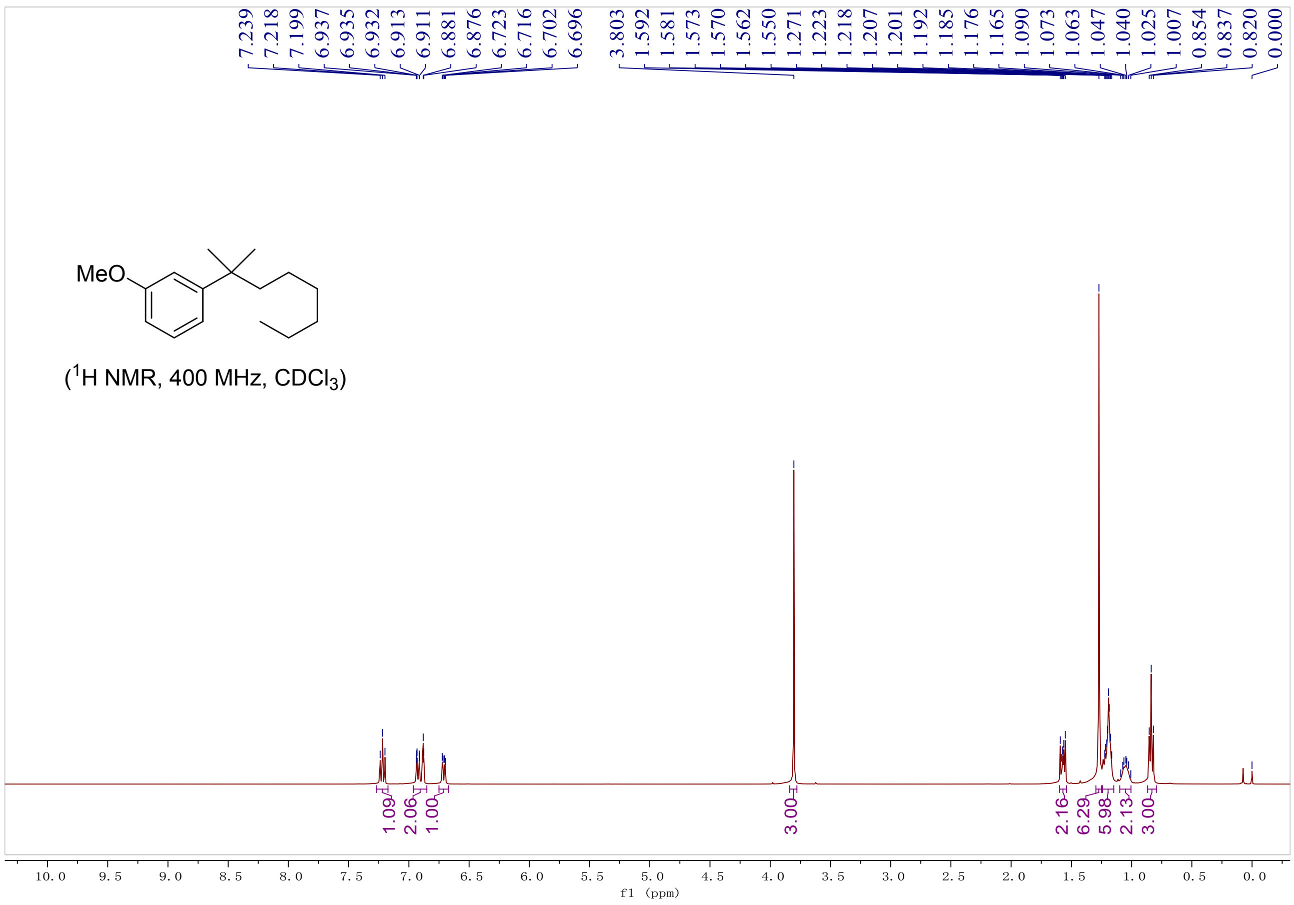
7.239
7.218
7.199
6.937
6.935
6.932
6.913
6.911
6.881
6.876
6.723
6.716
6.702
6.696
3.803
1.592
1.581
1.573
1.570
1.562
1.550
1.271
1.223
1.218
1.207
1.201
1.192
1.185
1.176
1.165
1.090
1.073
1.063
1.047
1.040
1.025
1.007
0.854
0.837
0.820
0.000
MeO
(1H NMR, 400 MHz, CDCl3)
1.09
2.06
1.00
3.00
2.16
6.29
5.98
2.13
3.00
f1 (ppm)

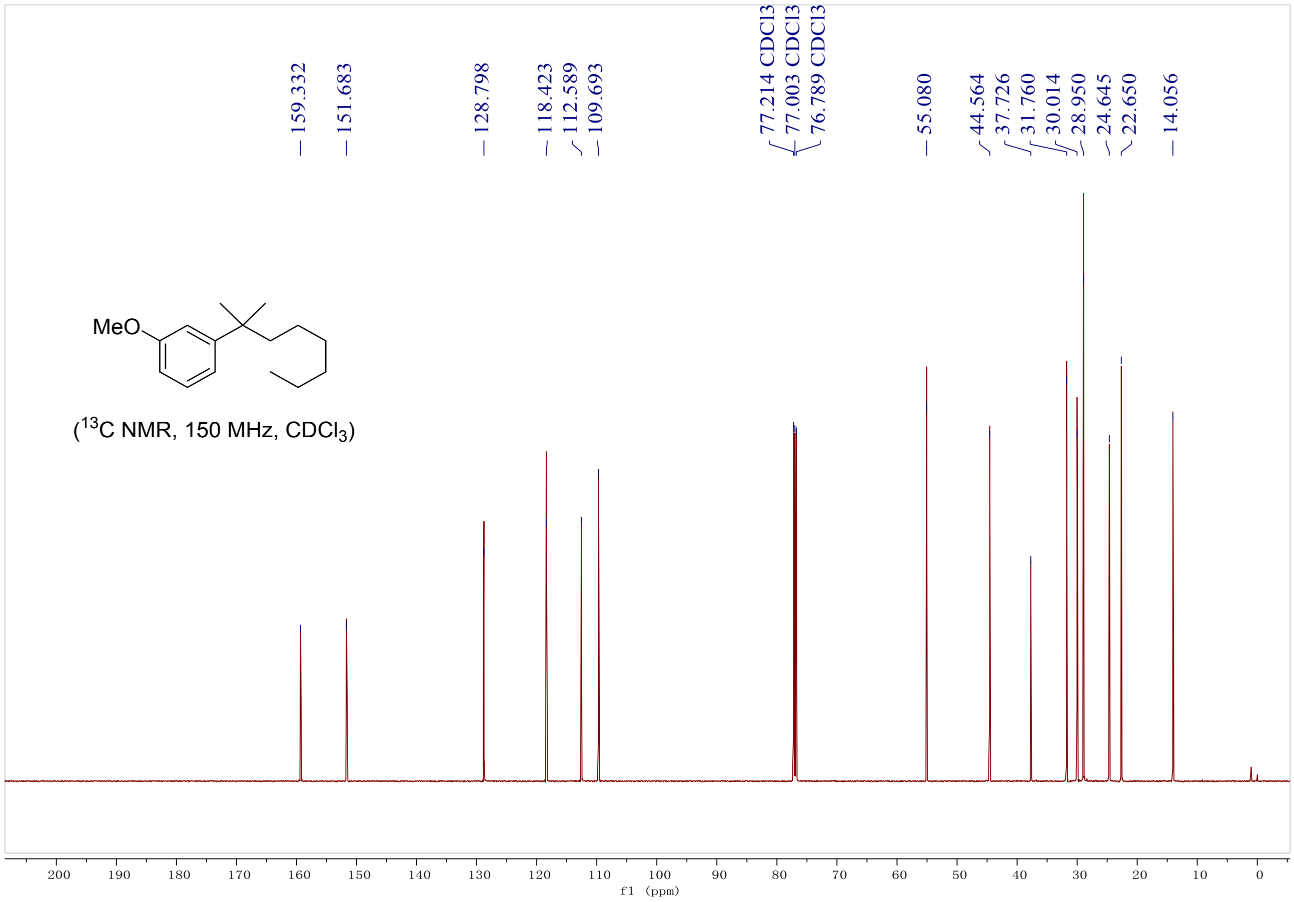
159.332
151.683
128.798
118.423
112.589
109.693
77.214 CDCl3
77.003 CDCl3
76.789 CDCl3
55.080
44.564
37.726
31.760
30.014
28.950
24.645
22.650
14.056
MeO
(13C NMR, 150 MHz, CDCl3)
f1 (ppm)

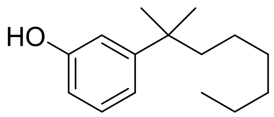

3-(2-methyloctan-2-yl)phenol (16): colorless oil, 627 mg, 79%, Eluent: PE/EA = 4/1.

^{1}H NMR (400 MHz, $CDCl_3$, TMS): δ 7.15 (t, *J* = 8.0 Hz, 1H), 6.93-6.86 (m, 1H), 6.81 (t, *J* = 2.2 Hz, 1H), 6.63 (dd, *J* = 8.0, 2.6 Hz, 1H), 3.96 (s, 1H), 1.60-1.50 (m, 2H), 1.28-1.16 (m, 12H), 1.08-0.99 (m, 2H), 0.84 (t, *J* = 6.8 Hz, 3H). **^{13}C NMR** (150 MHz, $CDCl_3$, TMS): δ 14.0, 22.6, 24.6, 28.9, 30.0, 31.8, 37.6, 44.5, 112.1, 113.1, 118.4, 129.0, 152.1, 155.2. **IR** (EtOH): ν 3330, 2957, 2928, 2870, 1587, 1452, 1215, 1166, 1078, 914, 872, 780, 701 cm^{-1}. **HRMS** (ESI) m/z: $[M]^+$ Calcd. for $C_{15}H_{23}O$ 219.1749; found 219.1748.

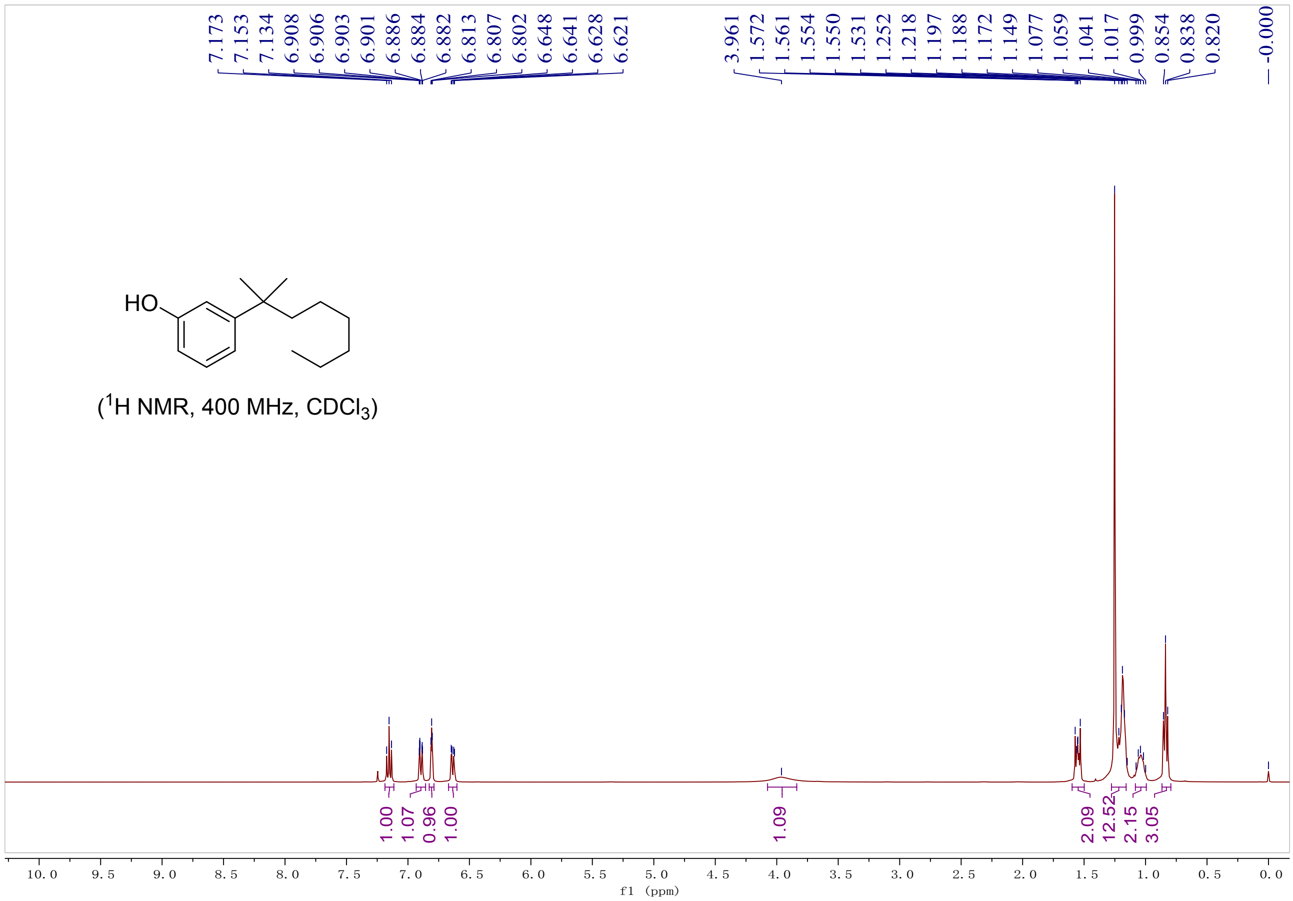

7.173
7.153
7.134
6.908
6.906
6.903
6.901
6.886
6.884
6.882
6.813
6.807
6.802
6.648
6.641
6.628
6.621
3.961
1.572
1.561
1.554
1.550
1.531
1.252
1.218
1.197
1.188
1.172
1.149
1.077
1.059
1.041
1.017
0.999
0.854
0.838
0.820
-0.000
HO
(^{1}H NMR, 400 MHz, $CDCl_3$)
1.00
1.07
0.96
1.00
1.09
2.09
12.52
2.15
3.05
f1 (ppm)

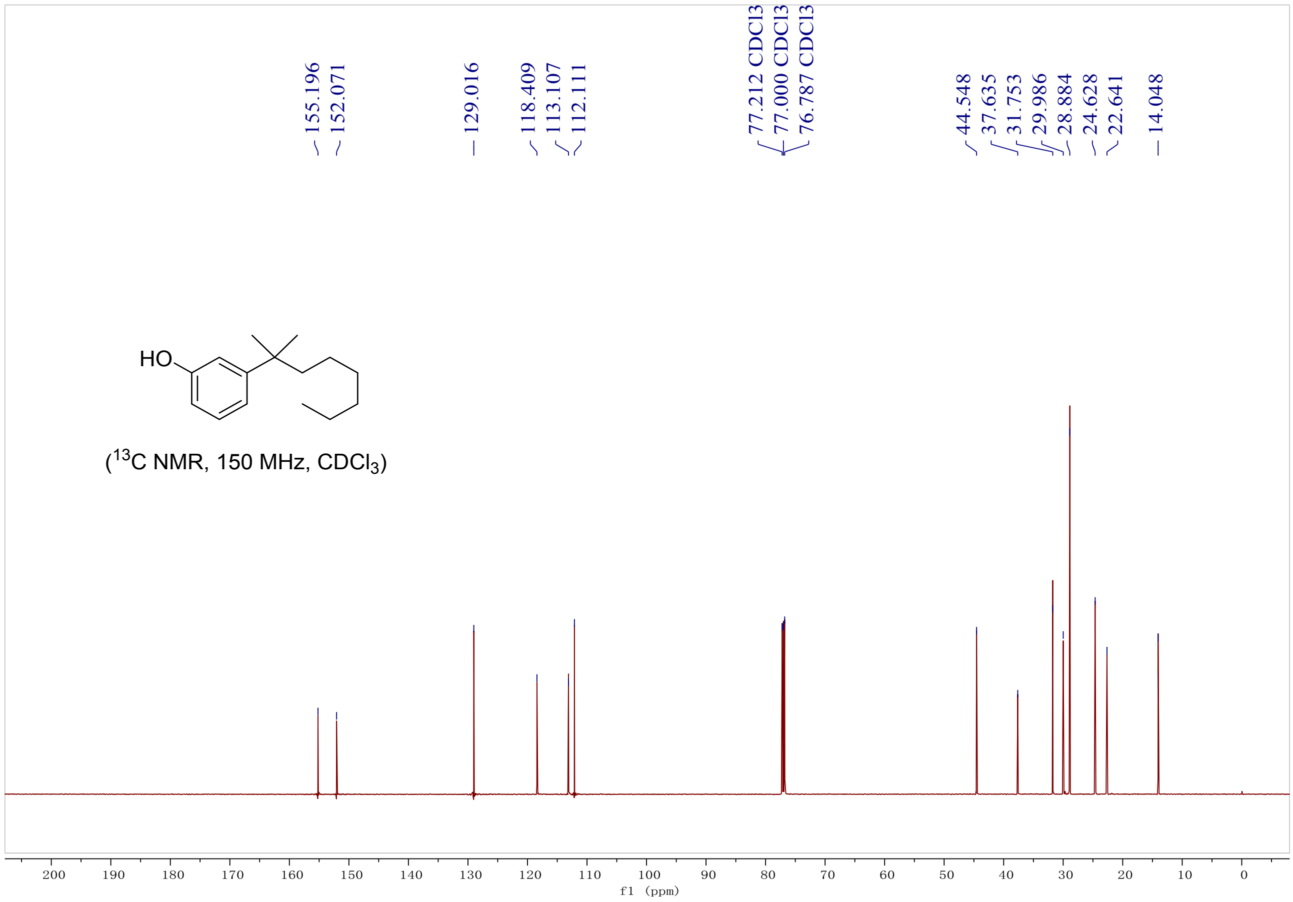

155.196
152.071
129.016
118.409
113.107
112.111
77.212 CDCl3
77.000 CDCl3
76.787 CDCl3
44.548
37.635
31.753
29.986
28.884
24.628
22.641
14.048
HO
(^{13}C NMR, 150 MHz, $CDCl_3$)
f1 (ppm)

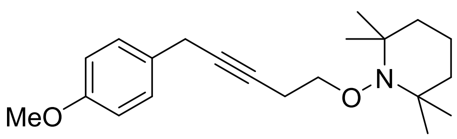

1-((5-(4-methoxyphenyl)pent-3-yn-1-yl)oxy)-2,2,6,6-tetramethylpiperidine (17): colorless oil, 16 mg, 49%, Eluent: PE/EA = 20/1.

^{1}H NMR (600 MHz, $CDCl_3$, TMS): δ 7.25 (d, *J* = 8.6 Hz, 3H), 6.84 (d, *J* = 8.6 Hz, 2H), 3.85 (t, *J* = 7.2 Hz, 2H), 3.79 (s, 3H), 3.51 (t, *J* = 2.4 Hz, 2H), 2.43 (tt, *J* = 7.2, 2.4 Hz, 2H), 1.58-1.50 (m, 1H), 1.48-1.42 (m, 4H), 1.33-1.27 (m, 1H), 1.17 (s, 6H), 1.10 (s, 6H). **^{13}C NMR** (150 MHz, $CDCl_3$, TMS): δ 17.1, 18.8, 20.1, 24.2, 33.0, 39.6, 55.3, 59.8, 74.9, 78.6, 79.4, 113.8, 128.8, 129.4, 158.2. **IR** (EtOH): ν 2960, 2927, 2857, 2823, 2218, 1587, 1488, 1452, 1217, 1167, 1046, 911, 861, 783, 673, 666 cm^{-1}. **HRMS** (ESI) m/z: $[M+H]^+$ Calcd. for $C_{21}H_{32}O_2N$ 330.2428; found 330.2427.

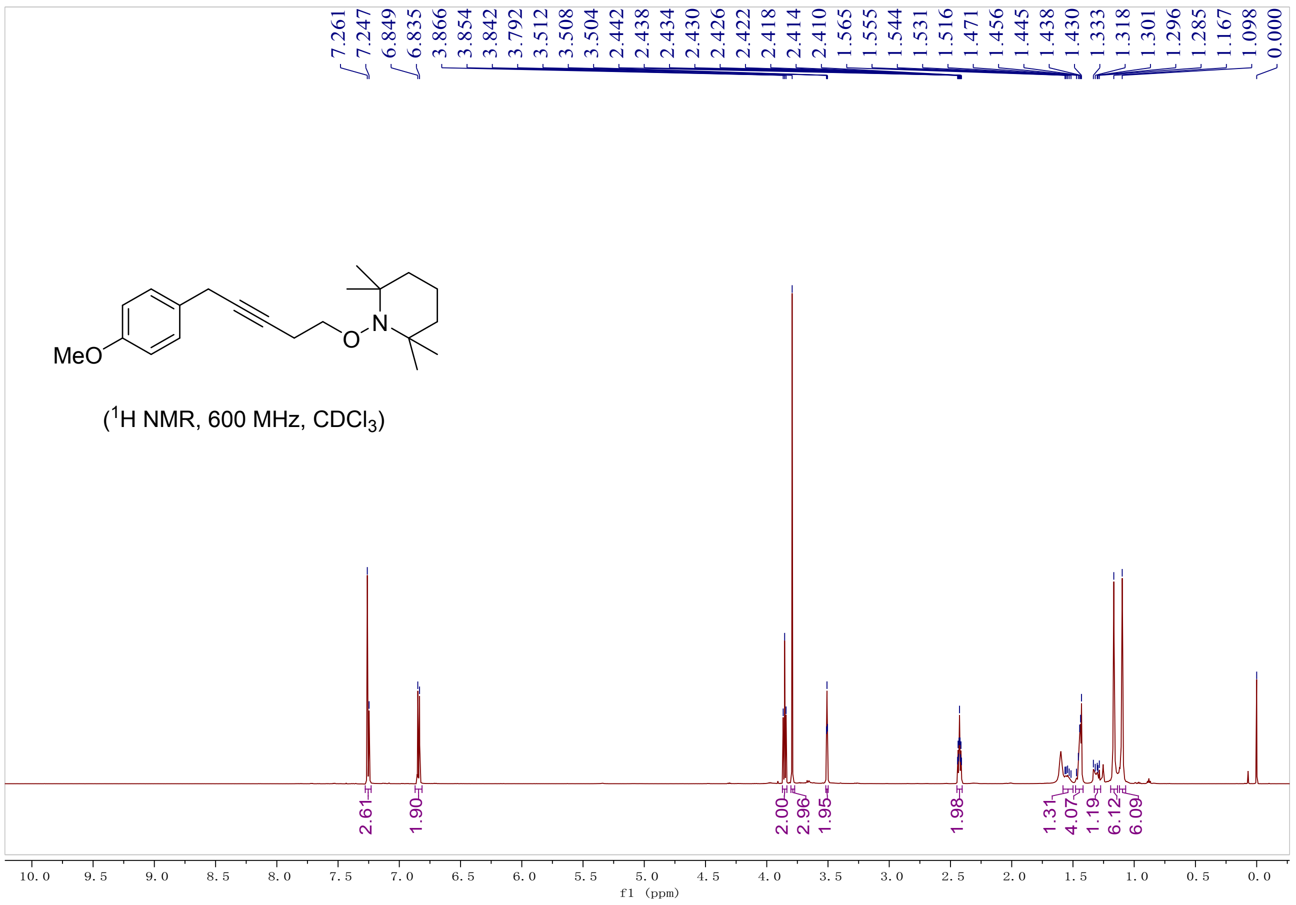

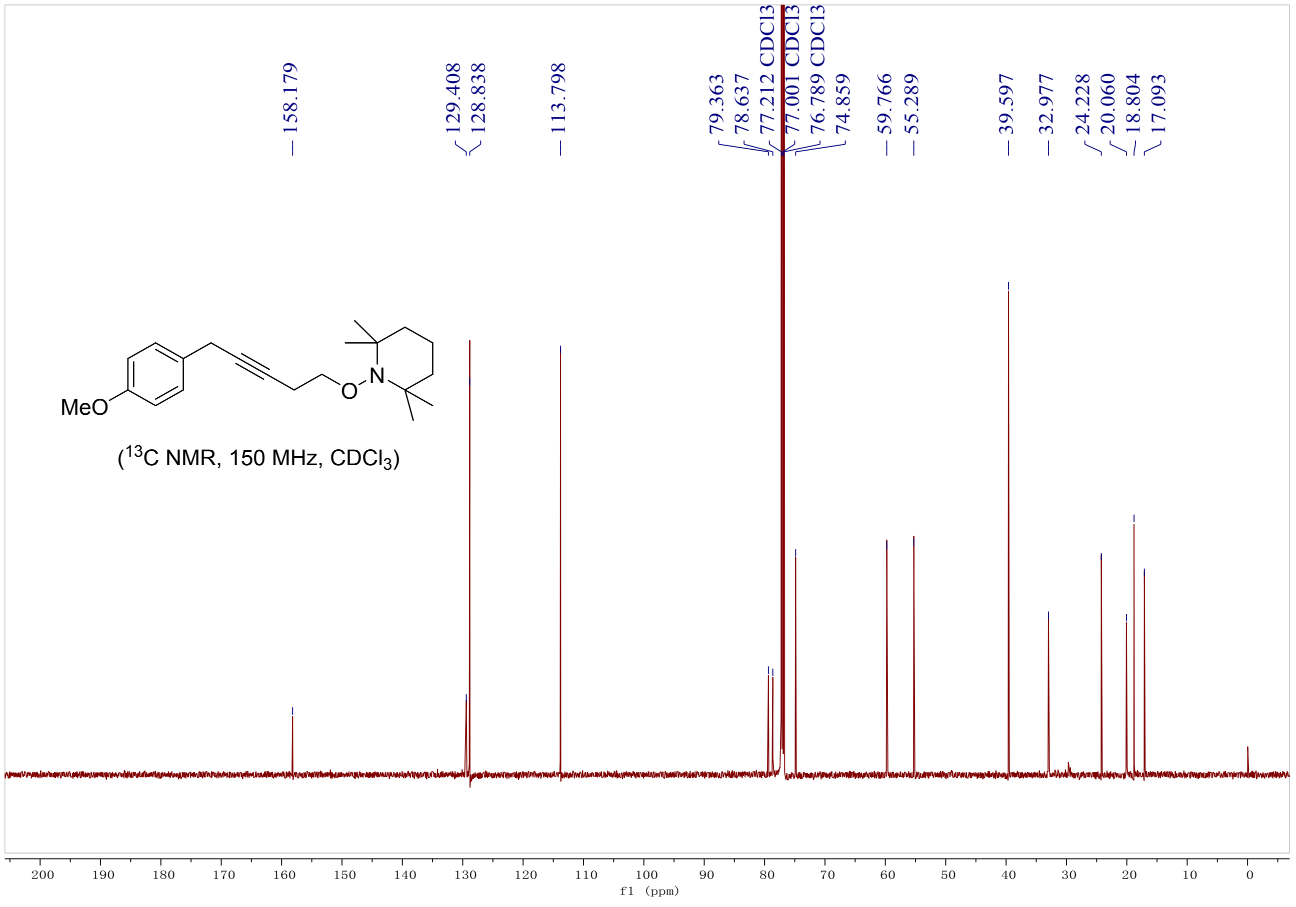

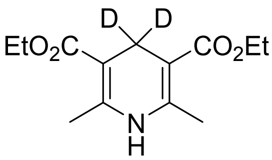

Diethyl 2,6-dimethyl-1,4-dihydropyridine-3,5-dicarboxylate-4,4-d_2 (2af): yellow solid, 408 mg, 50%.

^{1}H NMR (400 MHz, DMSO-d_6, TMS): δ 8.28 (s, 1H), 4.06 (q, J = 7.2 Hz, 4H), 2.12 (s, 6H), 1.19 (t, J = 7.2 Hz, 6H).

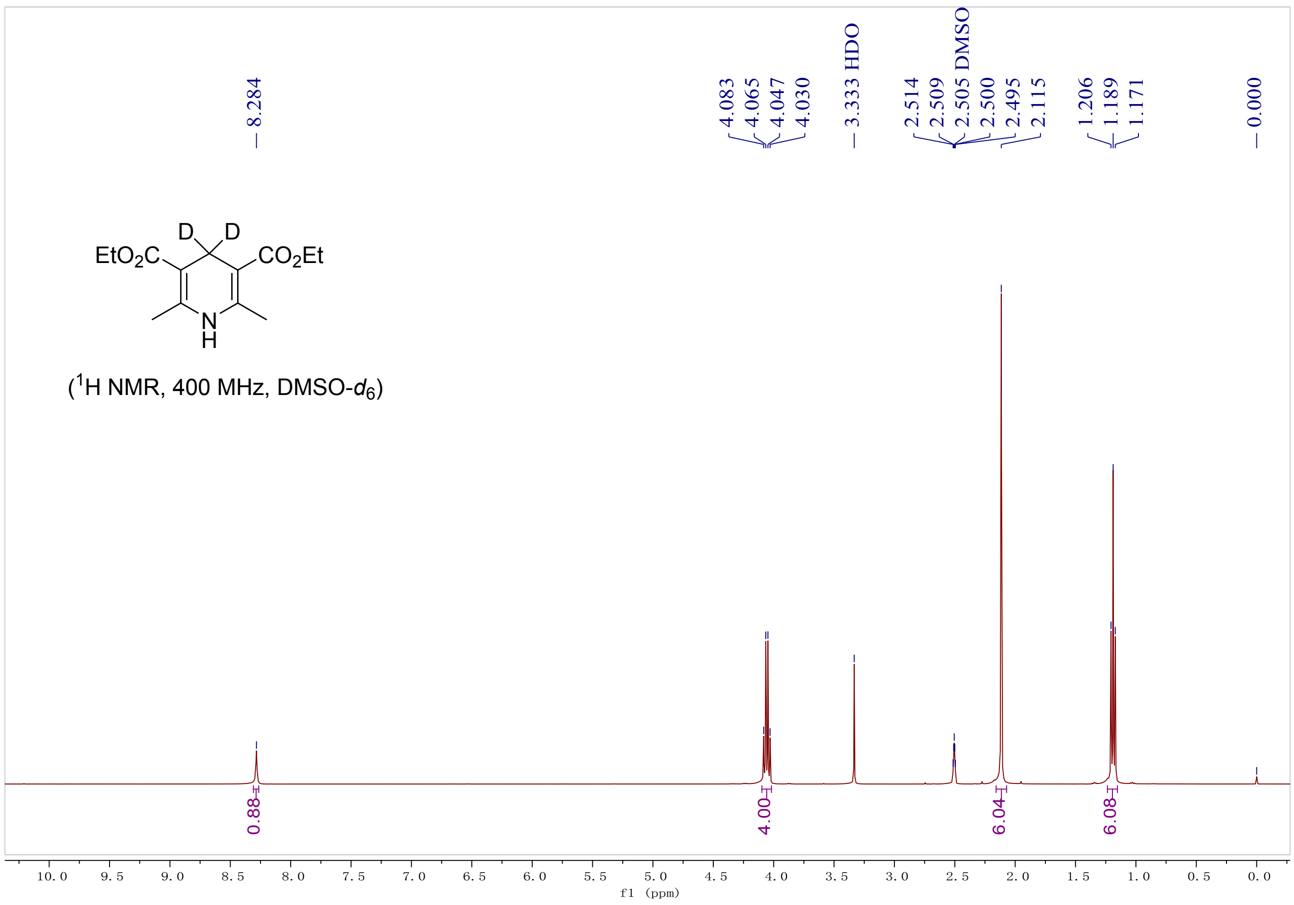

8.284
4.083
4.065
4.047
4.030
3.333 HDO
2.514
2.509
2.505 DMSO
2.500
2.495
2.115
1.206
1.189
1.171
0.000
D
D
EtO2C
CO2Et
N
H
(1H NMR, 400 MHz, DMSO-d6)
0.88
4.00
6.04
6.08
10.0
9.5
9.0
8.5
8.0
7.5
7.0
6.5
6.0
5.5
5.0
4.5
4.0
3.5
3.0
2.5
2.0
1.5
1.0
0.5
0.0
f1 (ppm)

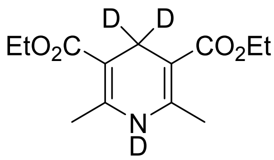

Diethyl 2,6-dimethyl-1,4-dihydropyridine-3,5-dicarboxylate-1,4,4-d_3 (2ag): pale green solid, 93 mg, 95%.

^{1}H NMR (400 MHz, $CDCl_3$, TMS): δ 4.17 (q, J = 7.2 Hz, 3H), 2.19 (s, 6H), 1.29 (t, J = 7.2 Hz, 6H).

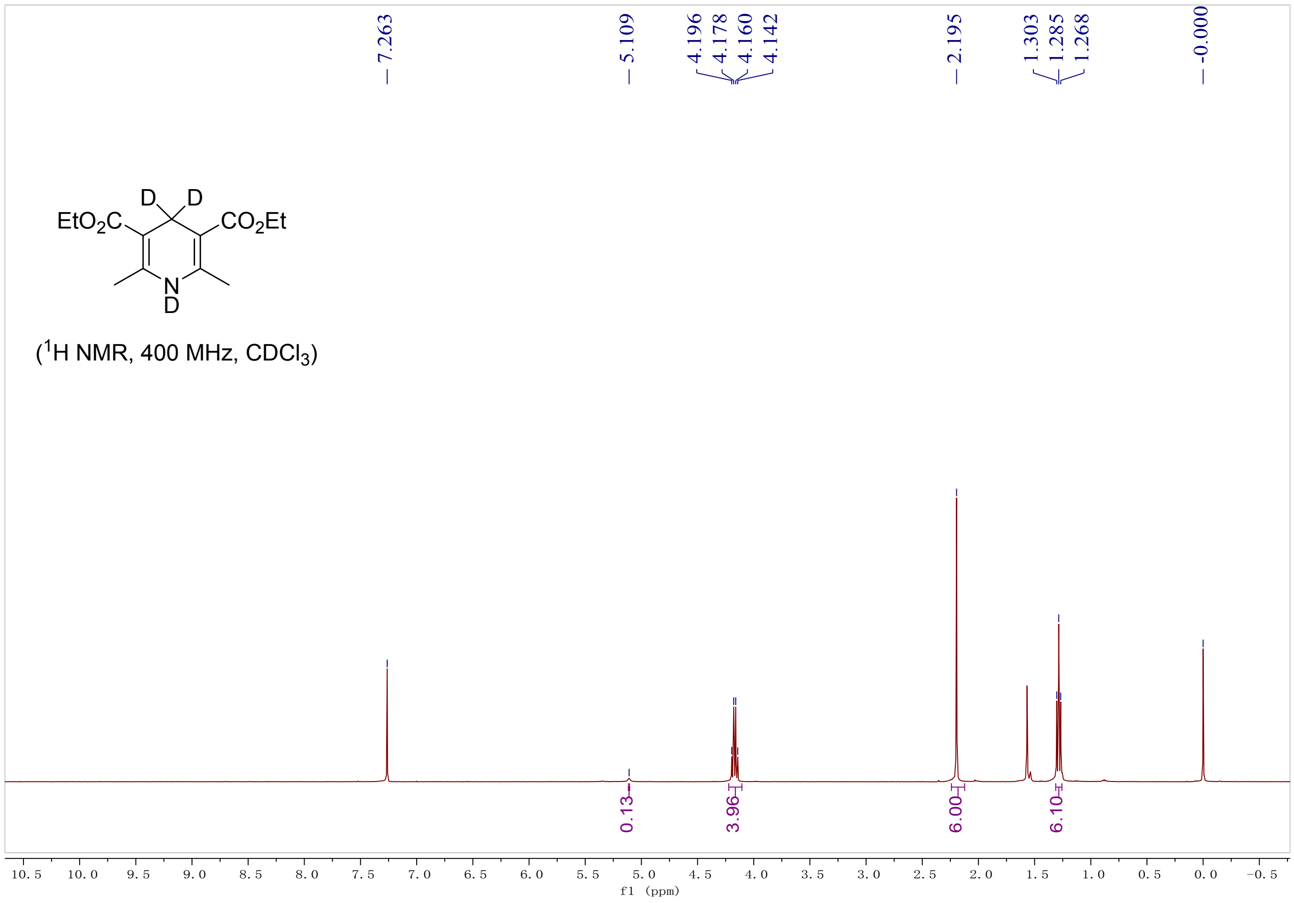

7.263
5.109
4.196
4.178
4.160
4.142
2.195
1.303
1.285
1.268
-0.000
EtO2C
CO2Et
D
N
(1H NMR, 400 MHz, CDCl3)
0.13
3.96
6.00
6.10
10.5 10.0 9.5 9.0 8.5 8.0 7.5 7.0 6.5 6.0 5.5 5.0 4.5 4.0 3.5 3.0 2.5 2.0 1.5 1.0 0.5 0.0 -0.5
f1 (ppm)

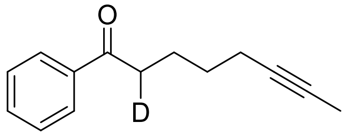

1-phenyloct-6-yn-1-one-2-d (*d*$_1$-3ba): colorless oil, 16 mg, 80%, Eluent: PE/EA = 20/1.

^{1}H NMR (400 MHz, $CDCl_3$, TMS): δ 8.00-7.94 (m, 2H), 7.59-7.53 (m, 1H), 7.51-7.42 (m, 2H), 3.00 (t, J = 7.4 Hz, 1.18H), 2.19 (tq, J = 7.2, 2.6 Hz, 2H), 1.90-1.80 (m, 2H), 1.77 (t, J = 2.6 Hz, 3H), 1.63-1.54 (m, 2H).

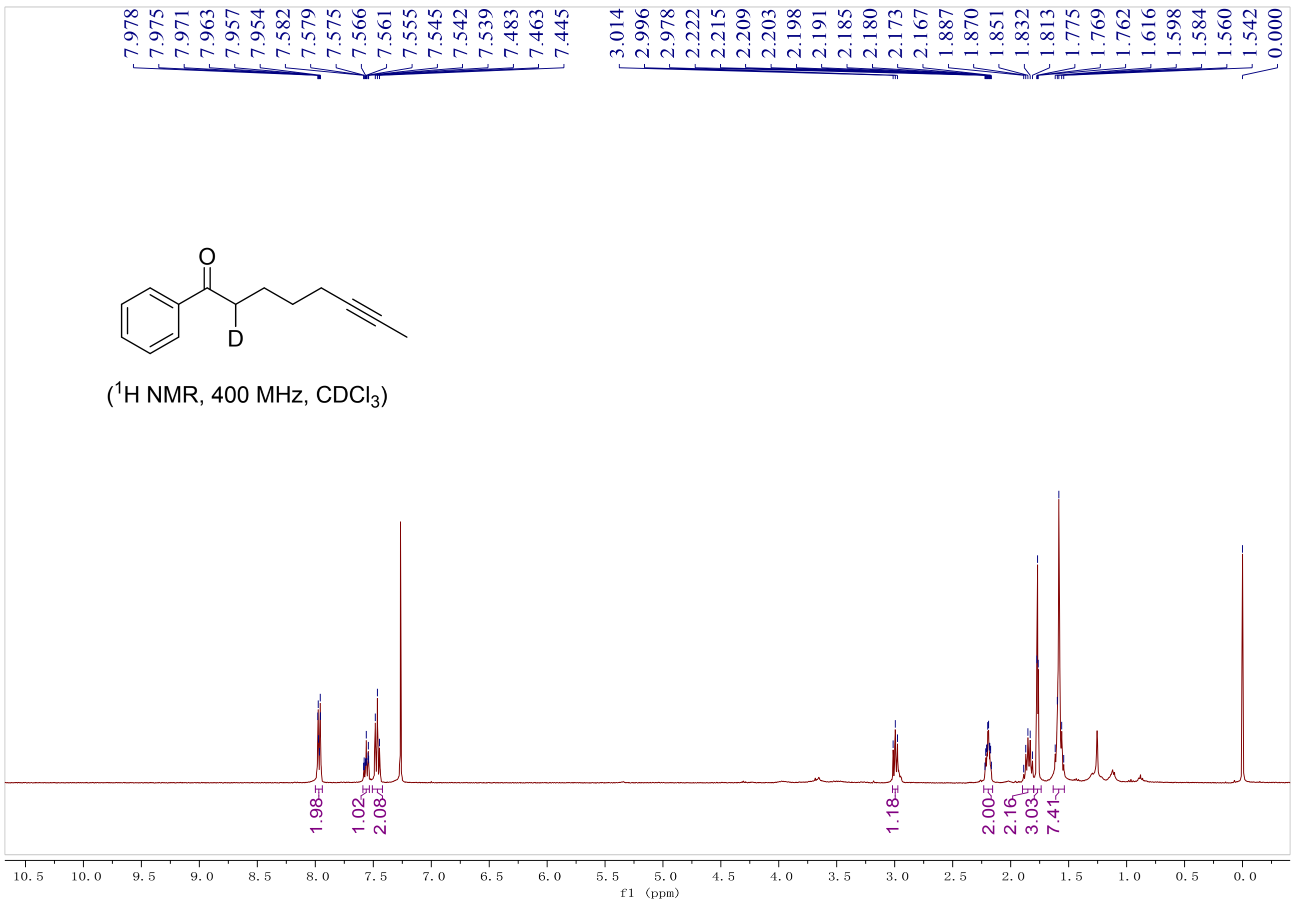

7.978 7.975 7.971 7.963 7.957 7.954 7.582 7.579 7.575 7.566 7.561 7.555 7.545 7.542 7.539 7.483 7.463 7.445
3.014 2.996 2.978 2.222 2.215 2.209 2.203 2.198 2.191 2.185 2.180 2.173 2.167 1.887 1.870 1.851 1.832 1.813 1.775 1.769 1.762 1.616 1.598 1.584 1.560 1.542 0.000
O
D
(1H NMR, 400 MHz, CDCl3)
1.98
1.02
2.08
1.18
2.00
2.16
3.03
7.41
10.5 10.0 9.5 9.0 8.5 8.0 7.5 7.0 6.5 6.0 5.5 5.0 4.5 4.0 3.5 3.0 2.5 2.0 1.5 1.0 0.5 0.0
f1 (ppm)

8. References

1. Lan, Y., Yang, C., Xu, Y.-H. & Loh, T.-P. Direct coupling of sp^3 carbon of alkanes with α,β-unsaturated carbonyl compounds using a copper/hydroperoxide system. *Org. Chem. Front.* **4**, 1411-1415 (2017). doi:10.1039/c7qo00149e
2. Zhao, Q.-Q., Zhou, X.-S., Xu, S.-H., Wu, Y.-L., Xiao, W.-J. & Chen, J.-R. Visible-light-driven nitrogen radical-catalyzed [3+2] cyclization of vinylcyclopropanes and *N*-tosyl vinylaziridines with alkenes. *Org. Lett.* **22**, 2470-2475 (2020). doi:10.1021/acs.orglett.0c00712
3. Nanteuil, F. de. & Waser, J. Synthesis of aminocyclobutanes by iron-catalyzed [2+2] cycloaddition. *Angew. Chem. Int. Ed.* **52**, 9009-9013 (2013). doi:10.1002/anie.201303803
4. Zhang, X., Jones‐Mensah, E., Deobald, J. & Magolan, J. Alkylation of indoles with α,β-unsaturated ketones using alumina in hexanes. *Adv. Syn. Catal.* **361**, 5548-5551 (2019). doi:10.1002/adsc.201901037
5. Luo, Y., Roy, I. D., Madec, A. G. & Lam, H. W. Enantioselective synthesis of allylboronates and allylic alcohols by copper-catalyzed 1,6-boration. *Angew. Chem. Int. Ed.* **53**, 4186-4190 (2014). doi:10.1002/anie.201310380
6. Zhang, Y.-H., Zhang, W.-W., Zhang, Z.-Y., Zhao, K. & Loh, T.-P. Manganese-catalyzed ring-opening coupling reactions of cyclopropanols with enones. *Org. Lett.* **21**, 5101-5105 (2019). doi:10.1021/acs.orglett.9b01703
7. Allais, C., Liéby-Muller, F., Rodriguez, J. & Constantieux, T. Metal-free michael-addition-initiated three-component reaction for the regioselective synthesis of highly functionalized pyridines: scope, mechanistic investigations and applications. *Eur. J. Org. Chem.* **2013**, 4131-4145 (2013). doi:10.1002/ejoc.201300246
8. Bugarin, A., Jones, K.-D. & Connell, B.-T. Efficient, direct alpha-methylenation of carbonyls mediated by diisopropylammonium trifluoroacetate. *Chem. Commun.* **46**, 1715-1717 (2010). doi:10.1039/b924577d
9. Dong, X.-Y., Zhang, Y.-F., Ma, C.-L., Gu, Q.-S., Wang, F.-L., Li, Z.-L., Jiang, S.-P. & Liu, X.-Y. A general asymmetric copper-catalysed Sonogashira C(sp(3))-C(sp) coupling. *Nat. Chem.* **11**, 1158-1166 (2019). doi:10.1038/s41557-019-0346-2
10. Xia, Y., Wang, J. & Dong, G. Suzuki-miyaura coupling of simple ketones via activation of unstrained carbon-carbon bonds. *J. Am. Chem. Soc.* **140**, 5347-5351 (2018).

doi:10.1021/jacs.8b02462

11. Henderson, J.-R., Parvez, M. & Keay, B.-A. Diastereoselective diels-alder reactions of a novel cyclopropenyl-containing chiral auxiliary. *Org. Lett.* **9**, 5167-5170 (2007). doi: 10.1021/ol702280q
12. Spitzner, D. & Swoboda, H., Akzeptoren für die doppelte Michael-reaktion, eine verbesserte synthese von cyclopropylidencarbonsäureestern und deren reaktivität gegenüber cyclohexadienolaten. *Tetrahedron Letters* **27**, 1281-1284 (1986). doi: 10.1016/S0040-4039(00)84238-0
13. Scholz, D., Weber-Roth, S., Macoratti, E. & Francotte, E. Expedient synthesis of α-substituted α,β-unsaturated γ-amino acids (dipeptide mimetics); witting reaction of α-amino aldehydes with α-substituted alkoxycarbonyl phosphoranes. *Syn. Commun.* **29***,* 1143-1155 (1999). doi: 10.1080/00397919908086084
14. Trowbridge, A., Reich, D. & Gaunt, M.-J. Multicomponent synthesis of tertiary alkylamines by photocatalytic olefin-hydroaminoalkylation. *Nature* **561**, 522-527 (2018). doi: 10.1038/s41586-018-0537-9
15. Das, M., Vu, M.-D., Zhang, Q. & Liu, X.-W. Metal-free visible light photoredox enables generation of carbyne equivalents via phosphonium ylide C-H activation. *Chem. Sci.* **10**, 1687-1691 (2019). doi: 10.1039/c8sc04195d
